# Diverse saturated heterocycles from a hydroacylation/conjugate addition cascade†

**DOI:** 10.1039/d1sc06900d

**Published:** 2022-01-19

**Authors:** Ndidi U. N. Iwumene, Daniel. F. Moseley, Robert D. C. Pullin, Michael C. Willis

**Affiliations:** Department of Chemistry, University of Oxford, Chemistry Research Laboratory Mansfield Road Oxford OX1 3TA UK michael.willis@chem.ox.ac.uk; Vertex Pharmaceuticals (Europe) Ltd 86-88 Jubilee Avenue, Milton Park Abingdon OX14 4RW UK

## Abstract

Rhodium-catalyzed hydroacylation using alkynes substituted with pendant nucleophiles, delivers linear α,β-unsaturated enone intermediates with excellent regioselectivity. These adducts are used to construct a broad range of diversely substituted, saturated O-, N- and S-heterocycles in a one-pot process. Judicious choice of cyclisation conditions enabled isolation of O-heterocycles with high levels of diastereoselectivity. A variety of derivatisation reactions are also performed, generating functionalised hydroacylation products. This sequence serves as a general approach for the synthesis of fully saturated heterocycles.

## Abstract 2

We demonstrate a one-pot hydroacylation/intramolecular conjugate-addition sequence to access a series of complex stereodefined heterocycles. Subsequent diversification of products is achieved, furnishing functionalized sp^3^-rich fragments.

## Graphical abstract

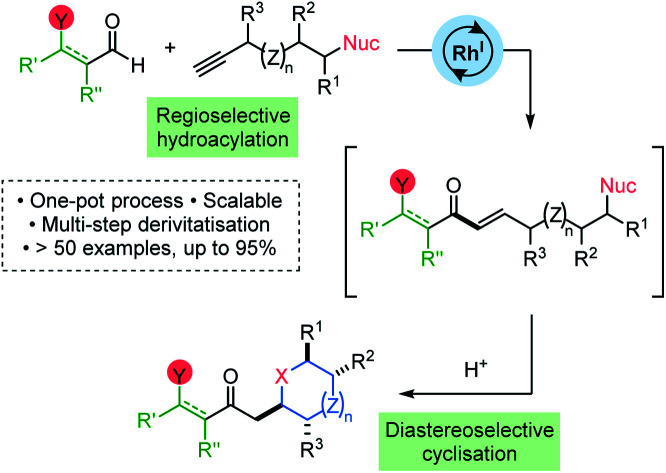

## Introduction

**Fig. 1:**
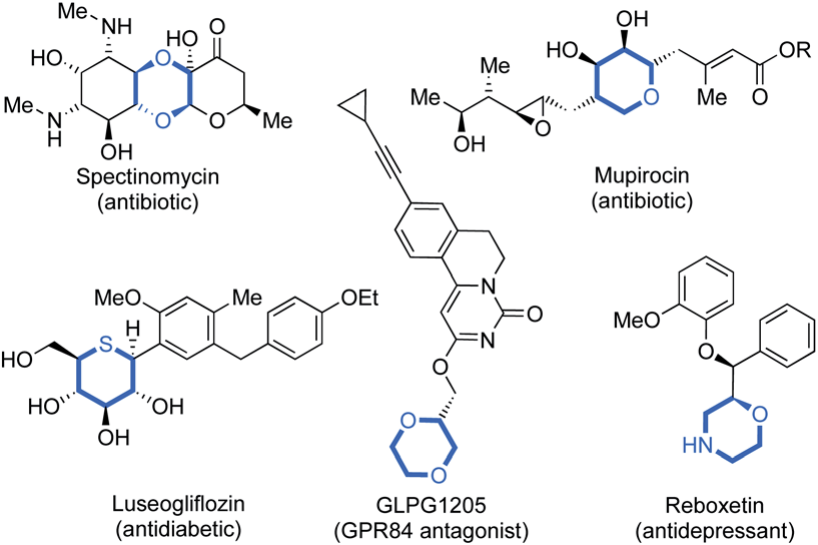
Examples of biologically active compounds containing saturated N-, O- and S-heterocycles.

Since Lovering's seminal ‘flatland’ epiphany,^1^ more emphasis lies on increasing saturation within therapeutics.^1,2^ This increased three-dimensionality results in the improved aqueous solubility,^1,2*g*,3^ and target selectivity of drug candidates.^4^ Saturated N-, O- and S-heterocycles are ubiquitous in natural and pharmaceutical products which display an extensive range of biological activities (Fig. 1).^5^ Thus, the efficient synthesis of saturated heterocyclic fragments is an important application of new synthetic methodology.

Recently, rhodium(i)-catalysed intermolecular hydroacylation,^6^ formally the atom-economic addition of a formyl C–H bond across a C–C π-bond,^7^ has been validated as a powerful C–C bond forming tool in heterocycle synthesis.^7*b*,*c*,8^ Of the variety of catalysts known to promote hydroacylation,^9^ rhodium(i)bisphosphines have proved to be highly effective.^10^ These catalysts have previously been exploited in intramolecular alkene hydroacylation approaches to the synthesis of 7- and 8-membered N-,^11^ S- and O-heterocycles,^7*a*^ and in the preparation 5,5- and 5,6-polycyclic nitrogen heterocycles.^12^

**Scheme 1:**
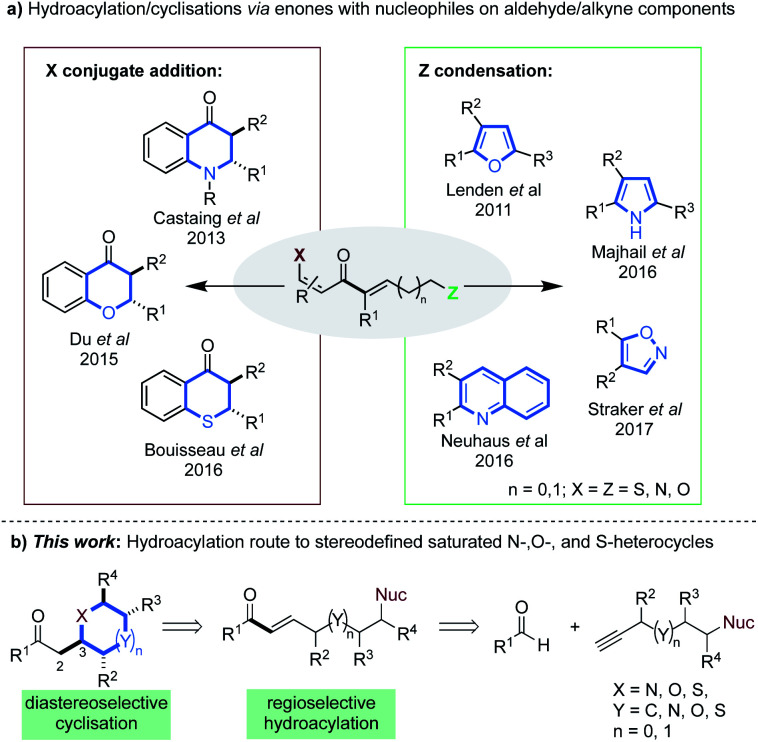
(a) Hydroacylation methods to various heterocycles *via* α,β-unsaturated enones; (b) our proposed alkyne hydroacylation route to stereodefined saturated heterocycles.

Intermolecular alkyne hydroacylation methods have emerged as a modular alternative to intramolecular cyclisation. However, these reactions can be plagued by deleterious decarbonylation pathways.^13^ To date, the most successful remedy has been chelation control;^14^ where a directing group, most often positioned beta to the aldehyde, forms a stable 5-membered metallacycle.^14*c*,15^ Methodologies that employ low catalyst loadings and aldehydes containing P-, O-, N-, or S-coordinating groups, have been reported.^16,10*c*,17,18,19^ Elegant disconnections of certain heterocyclic scaffolds, typically those that incorporate partial or full unsaturation, have been realized through tandem hydroacylation cyclisation sequences.^7*b*,*c*,8*a*–*e*^ The latter exploit highly electrophilic α,β-unsaturated enone intermediates (Scheme 1a), that contain aforementioned coordinating groups on the aldehyde (X) and/or nucleophilic groups on the alkyne components (Z).

Our laboratory has shown that β-heteroatoms on the aldehyde component can be exploited in conjugate–addition processes, yielding dihydroquinolones^8*b*^ and thiochroman-4-ones.^8*a*^ The Stanley group have also described the formation of 2,3-disubstituted chroman-4-ones *via* an analogous pathway, using O-coordinating aldehydes.^20^ The said enone intermediates, can also partake in condensation reactions with nucleophiles appended on the alkyne segment (Scheme 1a). This has allowed the synthesis of substituted furans,^8*c*^ pyrroles,^7*c*^ quinolines^7*b*^ and isoxazoles.^8*e*^ The above examples constitute encouraging precedent, however, most are either limited to specific substrate classes,^7*b*,8*a*–*c*,20^ rely on forcing conditions,^8*a*–*c*,8*e*,20^ or are not one–pot processes.^8*b*,*d*,*e*^ Most importantly, no previous work achieves regio- and diastereo-defined syntheses of saturated heterocycles.

We envisioned that diversely substituted saturated heterocycles of varied ring-size could be disconnected to α,β-unsaturated enones bearing pendant N-, O- or S-nucleophiles (Scheme 1b). In turn, these intermediates may be prepared by the hydroacylative union of appropriately substituted aldehydes and terminal alkynes. Such a route has the potential to generate stereodefined structures, provided that stereo-control can be achieved at the C3 position of the products (Scheme 1b).

In this article we describe a one-pot hydroacylation/diastereoselective conjugate-addition sequence, that delivers an exceptional scope of saturated N-, O- and S-heterocycles from simple unactivated substrates. We also demonstrate the utility of this method through product derivatization; achieving the modular assembly of complex scaffolds.

## Results and discussion

### O- and N-heterocycles

**Scheme 2:**
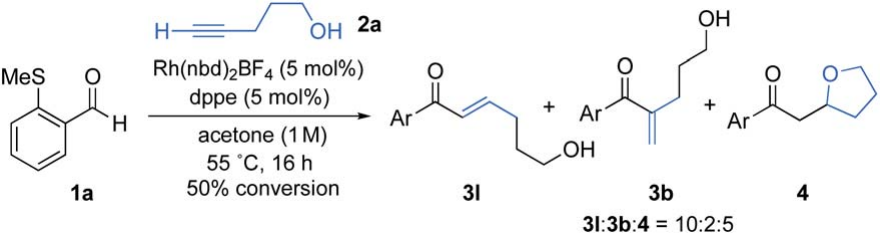
Intermolecular hydroacylation of aldehyde 1a and 4-pentyn-1-ol (2a).

We began our investigation by determining the tolerance of alkynols and alkynamines in hydroacylation reactions. We have previously shown that Rh(i)-catalysts that include small-bite-angle diphosphine ligands produce efficient and selective hydroacylation reactions. When a similar catalyst system was applied to the targeted reaction, the conversion was initially low (Scheme 2). Nonetheless, selectivity for the linear hydroacylation adduct was high.^21^ Conversions were dramatically improved when employing 1,2-dichloroethane (DCE) solvent and 1,2 bis(dicyclohexylphosphino)ethane (dcpe) as a ligand (see the ESI† for further details). Under these optimal conditions, a significant proportion of the tetrahydrofuran product was detected, suggesting the intermediate enone undergoes intramolecular conjugate-addition spontaneously. This observation is consistent with a serendipitous finding of a previous study from our laboratory.^22^ The successful hydroacylation reactions with alkynol 2a confirmed the feasibility of a tandem hydroacylation/cyclisation protocol. Accordingly, we then set out to construct substituted alkynols, with the aim of achieving a diastereoselective cyclisation.

A one-pot strategy was developed using alkynol 2b as a test substrate. Initially, hydroacylation of aryl sulfide 1a with 1.2 equiv. of alkynol, yielded dioxane 5a in a modest 4 : 1 dr and 86% total yield (Table 1, entry 1). Performing the cyclisation at room temperature was less efficient (entry 2). We then sought to improve the diastereoselectivity by inducing epimerisation of the forming stereocentre in product 5a. Stoichiometric quantities of the Brønsted acids, *p*-TsOH and MsOH increased selectivity to 11 : 1 and >20 : 1 dr, respectively (entries 3 and 4), but depreciated the overall yield. Dropping the loading of these acids to 20 mol% delivered a smaller increase in dr, without an overall reduction in yield (entries 5 and 6). When evaluating sub-stoichiometric quantities of Lewis acids at 55 °C (entries 7 to 10), the diastereoselectivity remained low. Conversely when BCl_3_ was used (entry 11), a dr of 7.3 : 1 was obtained, with no yield degeneration. On addition of BCl_3_ to the reaction vial, fuming HCl was observed. Inspired by this, an organic solution of stoichiometric HCl (entry 12) was tested, providing the desired 1,4-dioxane ring in 13 : 1 dr and 87% isolated yield. Attempts to extend the duration of epimerisation, or increase the equivalents of HCl, failed to produce a further increase in dr, without the accompaniment of product degradation (see the ESI† for further details).

**Table 1:** Optimisation of cyclisation diastereoselectivity using alkynol 2b

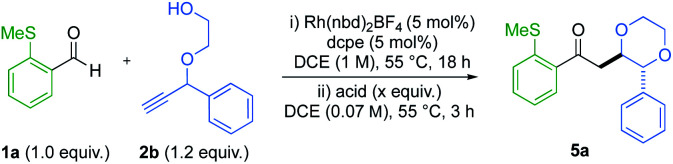			
Entry	Acid (equiv.)	Yield^a^ (%)	dr^b^
1	—	86^d^	4 : 1
2^c^	—	64^e^	3 : 1
3	*p*-TsOH (2.0)	62	11 : 1
4	MsOH (2.0)	33	>20 : 1
5	*p*-TsOH (0.2)	87	6.8 : 1
6	MsOH (0.2)	98	4.4 : 1
7	Sc(OTf)_3_ (0.2)	78	4.9 : 1
8	Ti(*i*-PrO)_4_ (0.2)	96	4.2 : 1
9	AlCl_3_ (0.2)	87	4.4 : 1
10	BF_3_·OEt_2_	76	4.4 : 1
11	BCl_3_ (0.2)	83	7.3 : 1
12	HCl in dioxane (2.0)	95(87)	13 : 1

a Determined using ^1^H NMR spectroscopy with methyl-3,5-dinitrobenzoate as an internal standard. Isolated yield in parentheses.

b Measured by ^1^NMR spectroscopy on the unpurified reaction mixture.

c Reaction at room temperature.

d >20 : 1 l : b regioselectivity, and >20 : 1 5a : 1a determined by ^1^H NMR spectroscopy.

e 13.9 : 1 l : b regioselectivity, and 6.2 : 1 5a : 1a determined by ^1^H NMR spectroscopy.

**Scheme 3:**
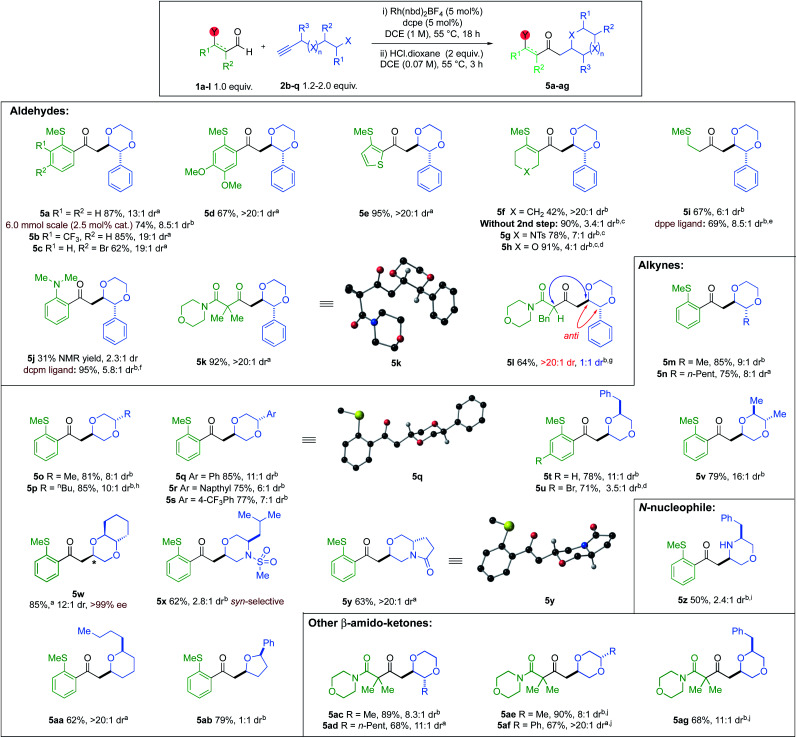
Aldehyde and alkyne scope; diastereomeric ratios were determined by ^1^H NMR spectroscopic analysis of the crude reaction mixture; ^a^isolated yield of a single major diastereoisomer; ^b^total isolated yield of both diastereoisomers; ^c^reactions with step (i) only; ^d^reactions carried out on 0.2 mmol scale; ^e^step (i) conditions: alkyne (2.0 equiv.), Rh(nbd)_2_BF_4_ (5 mol%), dppe (5 mol%), acetone (0.15 M), 55 °C, 2 h; ^f^step (i) conditions: Rh(nbd)_2_BF_4_ (10 mol%), dcpm (10 mol%), acetone (0.15 M), 55 °C, 4 h; ^g^>20 : 1 dr with respect to ring closure, 1 : 1 dr with respect to the aldehyde stereocentre; ^h^1.33 equiv. alkyne used; ^i^*p*-TsOH used in cyclisation step instead of HCl in dioxane; ^j^5 mol% DPEPhos used as ligand instead of dcpe.

With the optimised conditions in hand, we explored the reaction substrate scope (Scheme 3). Electron-withdrawing CF_3_-substituent (5b) and donating OMe-substituents (5d) provided good yields and high selectivity. An aryl bromide (5c) was also compatible. Clean conversion of a thiophene-derived substrate, afforded the dioxane 5e in 95% yield and >20 : 1 dr. Vinyl sulfide 5f decomposed under the acidic epimerisation conditions, resulting in a 42% yield with a >20 : 1 dr. However, without the acid step, the yield increased to 90%, but the dr was reduced. Other heterocyclic vinyl sulfides (X = NTs, O) provided similar results in the absence of HCl (5g,h). Alkyl sulfide 5i and 2-aminobenzaldehyde-derived product 5j, were obtained with good to moderate dr, but required alternative Rh-catalysts.^8*b*,23^ Di- and Mono-substituted β-ketoamides 5k and 5l, both displayed excellent ring-closing diastereoselectivity, with yields of 92% and 64%, respectively. Exploration of the alkyne scope proved that good yields and selectivity could be obtained for all substitution patterns on the 1,4-dioxane ring (5m to 5y). An enantiopure alkynol was also compatible, delivering *trans*-6,6-bicyclic species 5w in 85% yield and 12 : 1 dr, with retention of ee. A (*rac*)-leucine-derived, monocyclic morpholine 5x, was isolated in 62% yield, but with modest dr. A 6,5-pyrrolo-1,4-oxazine 5y could also be accessed in 63% yield with a >20 : 1 dr. Tetrahydropyran 5aa and tetrahydrofuran 5ab, were also isolated in 62% and 79% yields, respectively. The diastereoselectivity of 5-*exo*-trig ring closure within tetrahydrofuran 5ab, could not be improved (see ESI, Section 4†).^24^ A (*rac*)-phenylalanine-derived free morpholine heterocycle (5z) could be accessed from an N-nucleophilic conjugate addition, in good yield and moderate dr, using *p*-TsOH for the cyclisation and *N*-Boc deprotection. The scope was extended to β-amido substrates; good yields and selectivities were obtained for the desired 1,4-dioxane products 5ac to 5ag. We were pleased to find that the reaction could be performed on a gram-scale (6 mmol) with half the catalyst loading (2.5 mol%), furnishing 5a in 74% isolated yield and 8.5 : 1 dr.

The relative configurations of the major diastereoisomers of products 5k, 5q and oxazine 5y were unambiguously determined by X-ray crystallography to be *anti*.^25^ For heterocycle 5x, the relative configuration was determined to be *syn* through NOESY analysis (see ESI, Section 6.3†), and is consistent with earlier studies.^26^

### *S*-Heterocycles

**Scheme 4:**
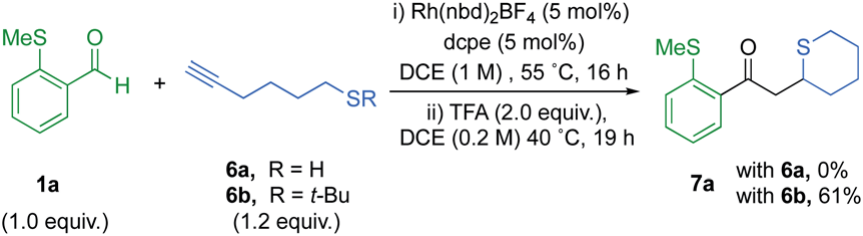
Tandem hydroacylation/*S*-conjugate addition to access thiane 7a.

We next targeted the synthesis of sulfur containing heterocycles,^27^ and for our initial investigation aldehyde 1a was paired with alkanethiol 6a under our previously optimized hydroacylation conditions (Scheme 4). However, after 16 hours the enone product did not form. It is probable that the free thiol chelates to an intermediary rhodium species and prevents catalysis. The latter mandated the use of a protecting group in the hydroacylation step.

We therefore selected *t*-Bu sulfide substituted-alkynes as suitable substrates. We were pleased to find that the coupling between aldehyde 1a and alkyne 6b proceeded to full conversion, with high selectivity for the linear regioisomer (14 : 1). Subsequent treatment with 2.0 equivalents of TFA, afforded thiane 7a in 61% yield (Scheme 4). Encouraged by this result, the cyclisation step was further optimized (Table 2). It became apparent that higher equivalents of acid and longer reaction times depreciated the yield (Table 2, entries 1–3). Using a shorter reaction time had minimal effect on yield (entry 4). However, diluting the reaction favoured ring-formation (entries 5 and 6), ultimately delivering the thiane in 82% yield. Further dilution did not significantly affect the yield (entry 7).

**Table 2:** Optimisation of one-pot hydroacylation/*S*-conjugate addition with *t*-Bu sulfide alkyne 6b^a^

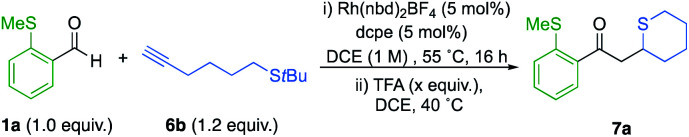				
Entry	Equiv. TFA	Time/h	Conc./M	Yield^b^ (%)
1	2	19	0.2	61
2	5	19	0.2	54
3	10	19	0.2	55
4	2	6	0.2	57
5	2	4	0.5	64
6	2	4	0.1	74(82)
7	2	4	0.025	70

a 100% conversion for step (i) based on analysis by ^1^H NMR spectroscopy.

b Yields determined using ^1^H NMR spectroscopy with 1,3,5-trimethoxybenzene as internal standard; isolated yields in parentheses.

**Scheme 5:**
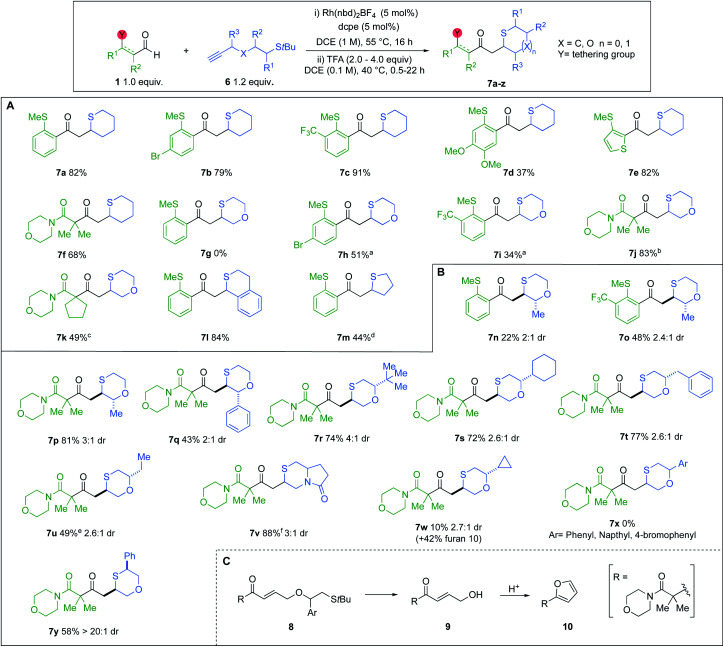
(A and B) Tandem hydroacylation/*S*-conjugate addition: scope of aldehydes and unsubstituted *t*-Bu sulfide alkynes; ^a^16 h; ^b^22 h, 4.0 equiv. TFA; ^c^step (i) 74% conversion based on analysis of ^1^H NMR; ^d^2 h, 1.0 equiv. TFA; ^e^step (i) 89% conversion based on analysis of ^1^H NMR; ^f^10 equiv. of TFA; (C) possible mechanism of competing pathway.

Having established an efficient one-pot protocol, the scope of the reaction was explored (Scheme 5A). Aldehydes bearing electron-withdrawing groups were well tolerated (7b and 7c). However, when OMe substituents were present, a lower yield of cyclic sulfide 7d was obtained. Heteroaromatic and β-amido aldehydes were also compatible, yielding thianes 7e and 7f in 82% and 68%, respectively. The transformation was also successfully extended to *t*-Bu sulfide alkynes possessing an oxygen atom linker. However, when *o*-SMe-benzaldehydes were used with these alkynes, the ensuing α,β-unsaturated enones underwent a competitive 6-*endo*-trig cyclisation, delivering thiochroman-4-ones.^8*a*^ Consequently, 1,4-oxathiane 7g could not be isolated. Pleasingly, incorporating electron-withdrawing groups on the *o*-SMe-benzaldehydes inhibited the competing pathway, providing improved yields of the desired oxathianes 7h and 7i. Employing β-amido chelating aldehydes eliminated the aforesaid competing cyclisation (7j and 7k). The reaction was also successfully applied to the synthesis of thiochromane derivative 7l, as well as tetrahydrothiophene 7m.

We next explored the scope of substituted *t*-Bu sulfide alkynes (Scheme 5B). Variation of groups at the R^3^ position was tolerated. Pairing methyl-substituted alkynes with *o*-SMe-aldehydes furnished lower yields of the desired cyclic sulfides (7n and 7o), due to the aforementioned competing 6-*endo*-trig ring closure. However, exploiting a dicarbonyl aldehyde enabled the synthesis of 1,4-oxathiane 7p in good yield. For the above cases, the crude diastereoselectivity did not exceed 3 : 1. Attempts to increase the selectivity through treatment of the diastereoisomeric mixture with weak base, resulted in epimerization to a 1 : 1 mixture. Incorporating a phenyl R^3^ substituent did not enhance the dr, but instead impacted the yield of cyclised product 7q. Employing acyclic and aliphatic R^2^ groups gave rise to diversely substituted saturated *S*-heterocycles, in moderate to high yields (7r–w). Notable among these compounds is 6,5-pyrrolo-1,4-thiazinone 7v, which has the potential to serve as a versatile scaffold in drug discovery.^28^ Alkynes featuring R^2^ groups with sp^2^ character (8) displayed alternate reactivity to give furan 10, as opposed to oxathianes 7x, under the cyclisation conditions (Scheme 5C). Cyclopropyl-derivative 7w shows intermediate reactivity, with a low yield of the oxothiane being achieved, along with furan 10. Moderate drs were also obtained with the R^2^ substituted alkynes, however, we were pleased to achieve excellent selectivity, when using a phenyl R^1^ substituent, delivering cyclic sulfide 7y as a single diastereoisomer.

**Scheme 6:**
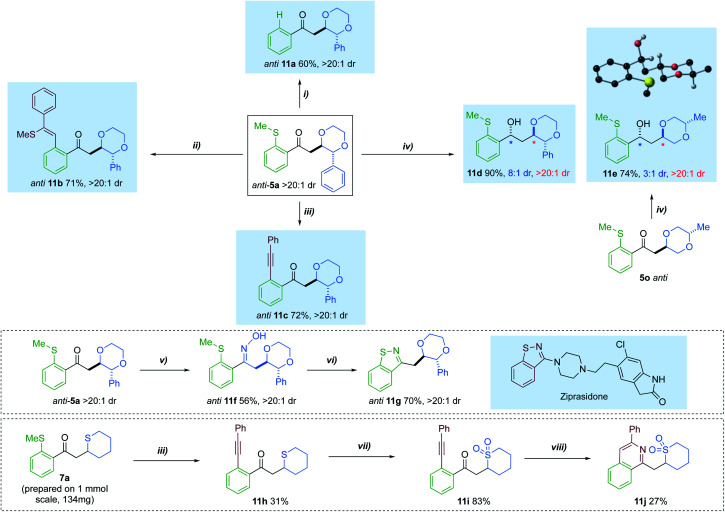
Transformations of saturated heterocycle hydroacylation adducts: (i) Rh(nbd)_2_BF_4_ (5 mol%), dcpm (5 mol%), (EtO)_3_SiH (10 equiv.), CH_2_Cl_2_ (0.15 M), r.t, 24 h; (ii) Rh(nbd)_2_BF_4_ (5 mol%), Xantphos (5 mol%), phenylacetylene (2.0 equiv.), DCE, 100–120 °C, 21 h; (iii) Rh(nbd)_2_BF_4_ (5 mol%), dcpe (5 mol%), CuBr (2.0 equiv.), Ag_2_CO_3_ (1.0 equiv.), phenylacetylene (2.0 equiv.), DCE (0.075 M), 80 °C, 16 h; (iv) Eu(OTf)_3_, LiBH_4_, −78 °C, Et_2_O, 2–3 h; (v) NH_2_OH·HCl (4.0 equiv.), pyridine (4.0 equiv.), MeOH, 60 °C, 20 h; (vi) Ac_2_O (4.0 equiv.), pyridine, 120 °C, 20 h; (vii) *m*-CPBA (3.0 equiv.), DCM (0.16 M), r.t, 3h; (viii) NH_4_OAc (5.5 equiv.), AgNO_3_ (0.8 equiv.), *t*-BuOH, 60 °C, 16 h.

Having accessed a series of diversely substituted saturated heterocycles, we sought to extend the utility of the method by investigating a series of transformations to further functionalise the products (Scheme 6). For example, the directing SMe group could be removed using a silane-mediated desulfurization to give phenyl ketone 11a.^14*c*^ Additionally, the thiomethyl tether was exploited as a ‘late-stage’ lynchpin in a rhodium-catalysed carbothiolation, giving product 11b in 70% yield.^29^ A Sonogashira-type coupling could also be performed on the *anti*-isomer of 5a, affording cross-coupled adduct 11c 72% yield.^30^ The ketone group of 5a could also be interconverted. A diastereoselective reduction with Eu(OTf)_3_ and LiBH_4,_ afforded alcohol 11d in 90% yield and 8 : 1 dr.^31^ When the same conditions were applied to dioxane 5o, with a distal Me-ring substituent, moderate diastereoselectivity could still be achieved. The *anti*-adduct was preferentially formed in these reductions.

We additionally carried out two multi-step sequences to access *bis*-heterocyclic motifs with relevance to the pharmaceutical industry. Condensation of ketone 5a, with hydroxylamine hydrochloride, provided oxime 11f in 56% yield.^32^ This was further activated using acetic anhydride and base, resulting in the formation of benzoisothiazole 11g in 70% yield.^33^ Such a motif has relevance in a variety of FDA approved drug scaffolds,^34^ one of which being ziprasidone.^35^ Furthermore, cyclic sulfide 7a could also be coupled with phenylacetylene, delivering *o*-(1-alkynyl)phenylketone 11h. Treatment of 11h with *m*-CPBA resulted in clean conversion to the sulfone 11i. After modifying literature conditions,^36^ direct construction of isoquinoline 11j was achieved *via* annulation of 11i with ammonium acetate.

## Conclusions

In summary, we have developed a one-pot tandem hydroacylation/conjugate-addition sequence that delivers a diverse array of fully saturated O, N, and S-heterocycles in good to excellent yields. Likewise, the robustness of a rhodium(i)/dcpe catalyst system is demonstrated through exemplary functional group tolerance and high regioselectivity. For oxygen ring-closure, significant enhancement of diastereoselectivity has been achieved, allowing access to single diastereomers (>20 : 1 dr) of O-heterocycles. The cyclised products have also been exploited in a series of derivatisation reactions, generating synthetically attractive and pharmaceutically intriguing molecules.

## Data availability

Full experimental and characterisation data are provided as part of the ESI.†

## Author contributions

N. U. N. I. and D. F. M. performed the experiments and analysed the results. All authors designed the project and wrote the manuscript. All authors discussed the results and commented on the manuscript.

## Conflicts of interest

There are no conflicts to declare.

## Supplementary Material

SC-013-D1SC06900D-s001

SC-013-D1SC06900D-s002
