# Supplementary material for: Diverse saturated heterocycles from a hydroacylation/conjugate addition cascade
Source: Chem Sci. 2022 Jan 19;13(5):1504–11. doi: 10.1039/d1sc06900d (PMC8809418; doi:10.1039/d1sc06900d)
Supplement: SC-013-D1SC06900D-s001 [file SC-013-D1SC06900D-s001.pdf]

Electronic Supplementary Material (ESI) for Chemical Science.
This journal is © The Royal Society of Chemistry 2022

Diverse Saturated Heterocycles from a Hydroacylation/Conjugate Addition Cascade

Ndidi U. N. Iwumene,[a+] Daniel. F. Moseley,[a+] Robert D. C. Pullin[b] and Michael C. Willis[a*]

[a] Chemistry Research Laboratory, University of Oxford, Mansfield Road, Oxford, OX1 3TA, UK

[b] Vertex Pharmaceuticals (Europe) Ltd, 86-88 Jubilee Avenue, Milton Park, Abingdon, Oxfordshire, OX14 4RW

[+]These authors contributed equally

michael.willis@chem.ox.ac.uk

Supporting Information

Table of Contents

1. General Information 3

2. General Procedures 4

3. Substrate Synthesis 9

3.1 Aldehyde substrates 9

3.2 Alkyne substrates 18

4. Reaction Optimisation 41

5. Assignment of Relative Configurations of cyclised products 45

5.1 *O*- and *N*-heterocycles 45

5.1.1 R^3 substituents 45

5.1.2 R^2 substituents 47

5.1.3 R^1 substituents 49

5.2 *S*-heterocycles 45

5.2.1 R^3 substituents 54

5.2.2 R^2 substituents 55

5.2.3 R^1 substituents 55

6. *O*- and *N*-heterocycles Substrate Scope 57

6.1 Scope reaction procedures 57

6.2 Aldehyde scope compounds 59

6.3 Alkyne scope compounds 72

6.4 Alkyne scope with beta-amido aldehyde 96

6.5 Product derivatisations 100

7. Synthesis of *S*-heterocycles 108

7.1 Substrate reaction procedures 113

7.2 Aldehyde and alkyne substrates 113

7.3 Scope reaction procedures 137

7.4 *S*-heterocycle compounds 138

7.5 Product derivatisations 160

8. References 162

9. NMR spectra of Novel Compounds 165

1. General Information

All reactions were performed in oven-dried glassware, under argon, using standard Schlenk techniques. Unless otherwise stated, solvents and reagents were supplied by Sigma Aldrich, FluoroChem, Alfa Aesar or Acros Organics. Acetone and 1,2-DCE were dried over Drierite™ for 16 hours, distilled at atmospheric pressure and degassed using freeze-pump-thawed method with argon before use. Anhydrous THF and DMF were obtained by filtration through anhydrous alumina columns, using an Innovative Technology Inc. PS-400-7 solvent purification system. "Petrol" refers to the fraction of petroleum ether that boils between 40 and 60 °C. Reactions were monitored by analytical Thin Layer Chromatography (TLC), using silica gel F254 aluminium plates. "Brine" refers to a saturated aqueous solution of sodium choride. Spots were visualised under UV light (254 nm) or stained with a vanillin or potassium permanganate solution. Flash column chromatography was carried out with matrix 60 silica supplied by Sigma Aldrich. All infrared data were run neat on a Bruker Tensor 27 FT-IR spectrometer, with adsorptions reported in wavenumbers (cm^{-1}). Melting points were recorded on a Stuart Scientific Melting Point Apparatus SMP1. ^{1}H and ^{13}C NMR spectra were obtained on a Bruker AVIII400 (400 MHz) using the residual solvent signal as an internal standard ($CDCl_3$: $\boldsymbol{\delta_H}$ = 7.26 ppm, $\boldsymbol{\delta_C}$ = 77.16 ppm). Chemical shifts (δ) are quoted in ppm with multiplicities reported as follows: s, singlet; d, doublet; t, triplet; q, quartet; quin., quintet; m, multiplet; br, broad; bs, broad singlet. All coupling constants (*J* values) were reported in Hertz (Hz). High and Low resolution ESI mass spectra were recorded on a Waters LCT Premier high resolution mass spectrometer. For chiral analysis, the enantiomeric ratio (er) was determined by chiral stationary phase HPLC in a Dionex P680 chromatogram with a Dionex UVD170U detector (λ_{max} = 225 nm) using a flow rate of 1.0 mL min-1 with either a Daicel Chiralpack OJ-H column. The eluent used and retention times (τ_{major} and τ_{minor}) are described in the compound

assignment. Where diastereoisomeric products have been isolated as an inseparable mixture, the data for the ^{1}H and ^{13}C NMR major diastereoisomer is reported.

2. General Procedures

General Procedure A: Nucleophilic Substitution of 2-haloaldehydes with NaSMe

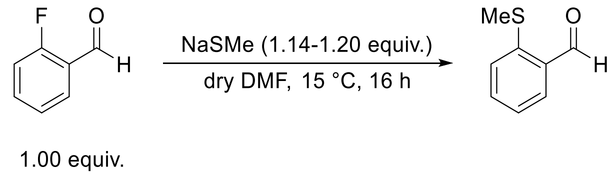

Prepared according to literature procedure.[1] NaSMe (1.14-1.20 equiv.) was added to a round-bottom flask and sparged with argon gas for 15 min. Anhydrous DMF was added and a cooling bath (ca. 15 °C) was applied. To the resulting suspension, a solution of the corresponding 2-haloaldehyde (1.00 equiv.) in DMF was added and the mixture stirred overnight (16 h). Once the reaction was complete as monitored by TLC analysis, the resulting mixture was poured into H_2O and the organic layer was separated. The remaining aqueous layer was extracted with EtOAc (× 3) and the combined organic extracts were washed with H_2O, brine, dried with $MgSO_4$, filtered and concentrated *in vacuo*. The crude residue was then purified by column chromatography to yield the desired product(s).

General Procedure B: Amide Couplings

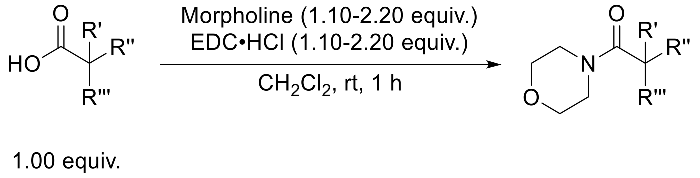

To a solution of the corresponding acid (1.00 equiv.) in CH_2Cl_2 was added morpholine (1.10-2.20 equiv.) and EDC·HCl (1.10-2.20 equiv.) and the mixture stirred for 1 h at room temperature. Once complete by TLC analysis, the mixture was quenched with sat. aq. $NaHCO_3$

and the layers were separated. The organic phase was then washed with brine, dried over $MgSO_4$, filtered and concentrated *in vacuo*. Purification by column chromatography yielded the corresponding amides.

General Procedure C: Alkylation of Propargyl Alcohols with ethyl 2-bromoacetate

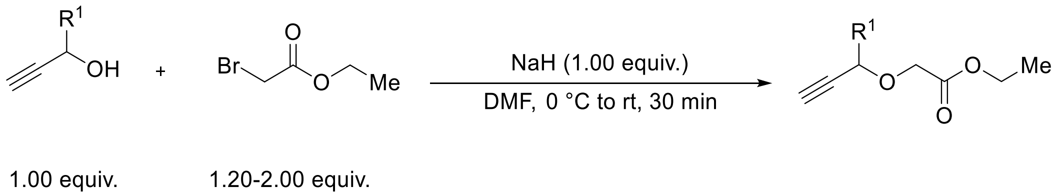

Prepared according to a modified literature procedure.[2] To a stirred suspension of NaH (60% in mineral oil, 1.00 equiv.) in DMF under N_2 at 0 °C was added a solution of the corresponding propargyl alcohol (80% w/w in toluene, 1.00 equiv.) in DMF. The resulting slurry was stirred at 0 °C for 30 min before a solution of ethyl-2-bromoacetate (1.20-2.00 equiv.) in DMF, was added dropwise at 0 °C. The mixture was then warmed to room temperature (unless otherwise stated) and stirred for a further 30 min (unless otherwise stated). After completion (as monitored by TLC analysis), the reaction mixture was quenched by addition of sat. aq. NH_4Cl at 0 °C. The mixture was then extracted with EtOAc (× 3) and the combined organic extracts were washed with 5% aq. LiCl, dried over $MgSO_4$, filtered and concentrated *in vacuo*. Purification of the crude residue by column chromatography yielded the corresponding propargyl ether.

General Procedure D: Propargylation of alpha-hydroxyesters and *N*Boc phenylalaninol

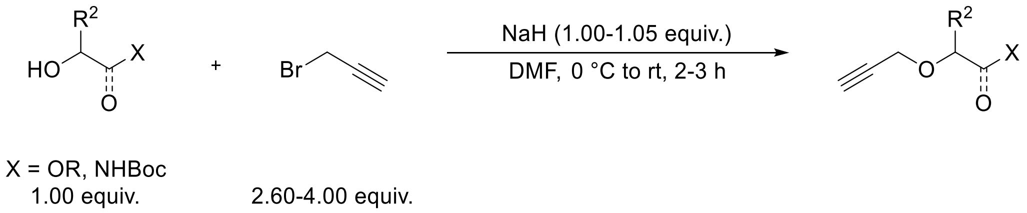

Prepared according to a literature procedure.[3] The desired alpha-hydroxyester (1.00 equiv.) was dissolved in anhydrous DMF and cooled to 0 °C. NaH (60% in mineral oil, 1.00-1.05 equiv.) was then added portion wise and the resulting solution was stirred for 1 h at 0 °C. Propargyl bromide (80% in toluene, 2.60-4.00 equiv.) was added dropwise at this temperature before warming to room temperature and stirring for 2-3 h (unless otherwise stated). Once complete, the reaction was quenched with sat. aq. NH_4Cl and the resulting mixture was extracted with EtOAc (× 3). The combined organic extracts were dried over $MgSO_4$, filtered and concentrated *in vacuo*. Purification of the crude residue by column chromatography yielded the corresponding propargyl ether.

General Procedure E: Ethyl Ester Reductions with $LiAlH_4$

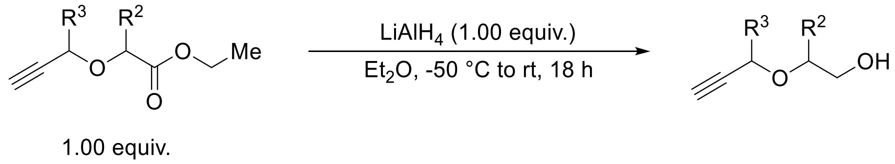

Prepared according to a literature procedure.[4] A solution of $LiAlH_4$ (4 M in Et_2O, 1.00 equiv.) was added to a two-neck flask containing Et_2O, at –50 °C under N_2. A solution of ethyl 2-(but-3-yn-2-yloxy)acetate (1.00 equiv.) in Et_2O, was then added dropwise over 1 h at –50 °C with stirring. The resulting mixture was then stirred for 90 min at –30 °C before being warmed to room temperature and stirred overnight (16 h). The mixture was then quenched by addition of H_2O (1 mL), NaOH (3.80 M, 1 mL), and then H_2O (1 mL), followed by filtration through celite. The filter cake was washed with Et_2O (2 × 10 mL) and the combined filtrate was dried over

Na_2SO_4, filtered and concentrated *in vacuo*. Purification by column chromatography yielded the corresponding alcohol.

General Procedure F: Yb(OTf)$_3$ Catalysed Epoxide Ring Openings

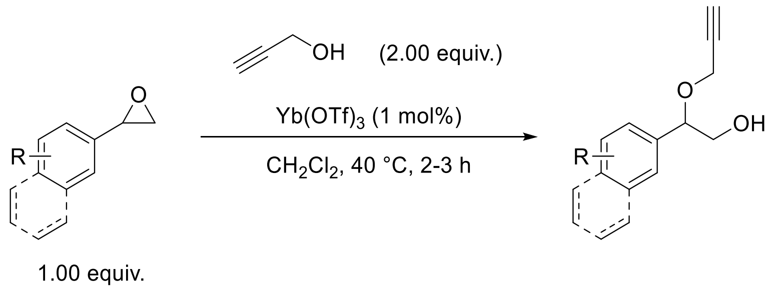

Prepared according to a modified literature procedure.[5] A two-neck flask containing anhydrous Yb(III)OTf (0.010 equiv.) was evacuated and backfilled 3-times with N_2 before anhydrous CH_2Cl_2 and the corresponding epoxide (1.00 equiv.) and were added sequentially. Propargyl alcohol (2.00 equiv.) was then added and the mixture was stirred at reflux (ca. 40 °C, unless otherwise stated) for 2-3 h. Once the reaction was complete by TLC analysis, the crude mixture was poured into H_2O and organic layer was separated. The aqueous layer was extracted with CH_2Cl_2 (×3) and the combined organic extracts were dried over Na_2SO_4, filtered and concentrated *in vacuo*. Purification of the crude residue by column chromatography to yield the corresponding ring-opened product.

General Procedure G: Methyl Ester Reductions with LiBH$_4$

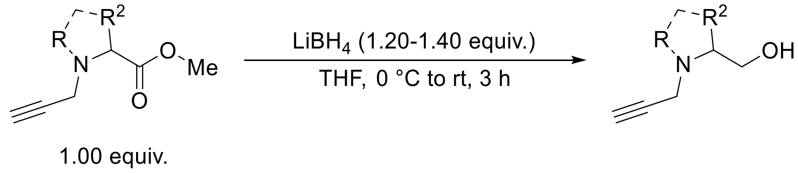

Prepared according to a literature procedure.[6] To solution of methyl ester (1.00 equiv.) in anhydrous THF under argon at 0 °C was added dropwise $LiBH_4$ solution (2 M in THF, 1.20-1.40 equiv.). The mixture was allowed to warm to room temperature and stirred for 3 h ***(Unless stated otherwise)***. Once the reaction was complete by TLC analysis, the mixture was

concentrated *in vacuo*. H_2O was added to the resulting residue, and the mixture was extracted with EtOAc (×3). The combined organic extracts were dried over Na_2SO_4, filtered and concentrated *in vacuo*. Purification of the crude residue by column chromatography yielded the corresponding alcohol.

3. Substrate Synthesis

3.1 Aldehyde substrates

2-(Methylthio)benzaldehyde (1a)

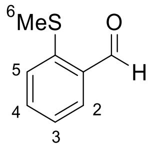

Prepared according to **general procedure A**, using NaSMe (1.80 g, 25.7 mmol, 1.14 equiv.) in DMF (7.5 mL), then 2-fluorobenzaldehyde (2.4 mL, 22.5 mmol, 1.00 equiv.) in DMF (15 mL). Work-up using H_2O (2 × 50 mL), EtOAc (3 × 35 mL) and brine (50 mL). Purification by column chromatography (6:1 to 4:1 Petrol:EtOAc) yielded 2-(methylthio)benzaldehyde **1a** (3.24 g, 21.3 mmol, 95%) as a yellow oil.

δ_H (400 MHz, $CDCl_3$) 10.27 (1H, s, C(1)*H*=O), 7.81 (1H, dd, *J* = 7.7, 1.6 Hz, *Ar*), 7.53 (1H, ddd, *J* = 8.1, 7.3, 1.6 Hz, *Ar*), 7.37-7.32 (1H, m, *Ar*), 7.28 (1H, dd, *J* = 8.2, 7.1 Hz, *Ar*), 2.50 (3H, s, SC(6)*H*$_3$); **δ_C** (101 MHz, $CDCl_3$) 191.5, 143.5, 134.1, 133.4, 133.0, 125.6, 124.5, 15.6; **ν_{max}**/ cm^{-1} (neat) 2920, 2840, 2739, 1617, 1586, 1558, 1260, 1195, 843, 750. *Data is consistent with literature*[1]

2-(Methylthio)-3-(trifluoromethyl)benzaldehyde (1b)

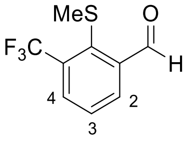

Prepared according to **general procedure A**, using NaSMe (290 mg, 4.14 mmol, 1.14 equiv.) in DMF (2.5 mL), then 2-fluoro-3-(trifluoromethyl)benzaldehyde (0.5 mL, 3.63 mmol, 1.00 equiv.) in DMF (7.5 mL). Work-up using H_2O (2 × 10 mL), EtOAc (3 × 10 mL) and brine (10

mL). Purification by column chromatography (3:97 EtOAc:Petrol) 3,4-dimethoxy-2-(methylthio)benzaldehyde **1b** (689 mg, 3.13 mmol, 86%) as an off-white solid.

m.p. (CH_2Cl_2) 34–36 °C; (*Lit:* 36–38 °C);[7] **δ_F** (377 MHz $CDCl_3$) -59.52; **δ_H** (400 MHz, $CDCl_3$) 10.77 (1H, d, J = 0.8 Hz, C(1)*H*=O), 8.04 (1H, ddd, J = 7.8, 1.0 Hz, *Ar*), 7.91 (1H, ddd, J = 7.8, 1.0 Hz, *Ar*), 7.56 (1H, app t, J = 7.7 Hz, *Ar*), 2.38 (3H, s, C(6)H_3); **δ_C** (101 MHz, $CDCl_3$) 191.9, 139.9, 139.6, 135.4 (q, J_{CF} = 29.3 Hz), 132.2, 131.7 (q, J_{CF} = 5.6 Hz), 129.3, 123.4 (q, J_{CF} = 274.0 Hz), 23.0; **m/z HRMS** (ESI^+) $[M + H]^+$ $C_9H_8OF_3{}^{32}S^+$ calc. 221.0243, found 221.0245. *Data is consistent with literature*[7]

4-Bromo-2-(methylthio)benzaldehyde (1c)

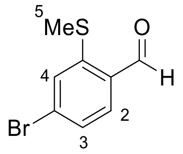

Prepared according to a modified procedure.[8] To a stirred solution of NaSMe (340 mg, 4.90 mmol, 1.13 equiv.), in DMF (25 mL) was added 4-bromo-2-fluorobenzaldehyde (1.00 g, 4.30 mmol, 1.00 equiv.) over 30 mins at –45 °C. The mixture was stirred at this temperature for 3 h before warming to room temperature overnight (16 h). The mixture was then quenched with H_2O (5 mL) and the resulting white precipitate was collected by Büchner filtration to yield 4-bromo-2-(methylthio)benzaldehyde **1c** (761 mg, 3.31 mmol, 78%) as a white solid.

m.p. (DMF) 80–83 °C; (*Lit:* 78 °C);[9] **δ_H** (400 MHz, $CDCl_3$) 10.17 (1H, s, C(1)*H*=O), 7.63 (1H, d, J = 8.1 Hz, C(2)*H*), 7.45-7.34 (2H, m, C(3 and 4)*H*), 2.49 (3H, s, SC(6)H_3); **δ_C** (101 MHz, $CDCl_3$) 190.3, 145.6, 134.4, 131.6, 129.8, 128.1, 127.7, 15.6; **m/z HRMS** (ESI^+) $[M + H]^+$ $C_8H_8O^{79}Br^{32}S^+$ calc. 230.9474, found 230.9476, $C_8H_8O^{81}Br^{32}S^+$ calc. 232.9453, found 232.9456. *Data is consistent with literature*[8]

3,4-Dimethoxy-2-(methylthio)benzaldehyde (1d)

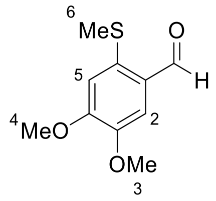

Prepared according to a modified procedure.[7] NaSMe (600 mg, 8.56 mmol, 1.20 equiv.) was added to a solution of 2-bromo-4,5-dimethoxybenzaldehyde (1.74 g, 7.13 mmol, 1.00 equiv.) in DMF (27 mL) at 0 °C, and the resulting solution stirred overnight (16 h) at 70 °C. After this time, H_2O (15 mL) was added and the mixture obtained extracted with Et_2O (3 × 15 mL), washed with brine (3 × 30 mL), dried over $MgSO_4$, filtered and concentrated *in vacuo*. Purification by column chromatography (1:19 to 1:4 EtOAc:Petrol) yielded 3,4-dimethoxy-2-(methylthio)benzaldehyde **1d** (1.03 g, 4.85 mmol, 68%) as an off-white solid.

m.p. (CH_2Cl_2) 97–99 °C; (*Lit:* 106–111 °C);[10] **δ_H** (400 MHz, $CDCl_3$) 10.38 (1H, s, C(1)*H*=O), 7.38 (1H, s, *Ar*), 6.93 (1H, s, *Ar*), 4.01 (3H, s, C(3 or 4)H_3), 3.94 (3H, s, C(3 or 4)H_3), 2.53 (3H, s, SC(6)H_3); **δ_C** (101 MHz, $CDCl_3$) 189.6, 154.0, 147.8, 136.6, 127.8, 111.7, 111.6, 56.1, 56.1, 18.2; **m/z HRMS** (ESI^+) $C_{10}H_{12}O_3{}^{23}Na^{32}S^+$ $[M + Na]^+$ calc. 235.0399, found 235.0402. *Data is consistent with literature[7]*

3-(Methylthio)thiophene-2-carbaldehyde (1e)

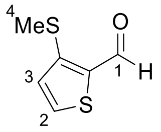

Prepared according to **general procedure A**, using NaSMe (650 mg, 9.34 mmol, 1.20 equiv.) in DMF (2.5 mL), then 3-bromothiophene-2-carbaldehyde (0.7 mL, 7.78 mmol, 1.00 equiv.) in DMF (7.5 mL). Work-up using H_2O (2 × 20 mL), EtOAc (3 × 20 mL) and brine (20 mL). Purification by column chromatography (1:49 to 1:9 EtOAc:Petrol) yielded 3-(methylthio)thiophene-2-carbaldehyde **1e** (693 mg, 4.39 mmol, 56%) as an off-white solid.

m.p. (CH_2Cl_2) 34–37 °C; **δ_H** (400 MHz, $CDCl_3$) 10.01 (1H, d, *J* = 1.1 Hz, C(1)*H*=O), 7.71 (1H, dd, *J* = 5.1, 1.1 Hz, *Ar*), 7.08 (1H, d, *J* = 5.1 Hz, *Ar*), 2.57 (3H, s, SC(4)*H*$_3$); **δ_C** (101 MHz, $CDCl_3$) 181.6, 146.1, 135.2, 134.7, 128.2, 17.2; **m/z HRMS** (ESI^+) $[M + H]^+$ $C_6H_7O^{32}S_2^+$ calc. 158.9933, found 158.9933. *Data is consistent with literature*[11]

2-Bromocyclohex-1-ene-1-carbaldehyde (S1) and 2-(methylthio)cyclohex-1-ene-1-carbaldehyde (1f)

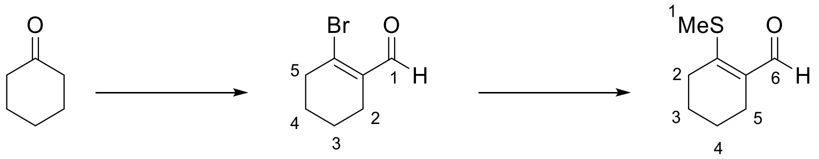

S1 **1f**

2-Bromocyclohex-1-ene-1-carbaldehyde (S1): Prepared according to a modified procedure.[12] To a solution of DMF (4.6 mL, 60.0 mmol, 3.00 equiv.) in CH_2Cl_2 (25 mL) was added PBr_3 (5.1 mL, 54.0 mmol, 2.70 equiv.) dropwise at 0 °C. The mixture was then stirred at this temperature for 1 h before cyclohexanone (2.1 mL, 20.0 mmol, 1.00 equiv.) was added at 0 °C. The mixture was then warmed to room temperature and stirred overnight (16 h). Once completion was shown by TLC analysis, the reaction mixture was poured into H_2O (75 mL), the layers were separated, and the aqueous layer was extracted with CH_2Cl_2 (3 × 25 mL). The

combined organic layers were washed with sat. aq. $NaHCO_3$ (75 mL), brine (75 mL), dried over $MgSO_4$, filtered and concentrated *in vacuo*. Purification by column chromatography (3:97 to 1:9 EtOAc:Petrol) yielded 2-bromocyclohex-1-ene-1-carbaldehyde **S1** (1.40 g, 7.45 mmol, 37%) as a colourless oil.

δ_H (400 MHz, $CDCl_3$) 9.95 (1H, s, C(1)*H*=O), 2.71-2.65 (2H, m, C(2 or 5)H_2), 2.24-2.18 (2H, m, C(2 or 5)H_2), 1.75-1.66 (2H, m, C(3 or 4)H_2), 1.66-1.57 (2H, m, C(3 or 4)H_2); **δ_C** (101 MHz, $CDCl_3$) 193.5, 143.5, 135.3, 38.8, 25.0, 24.3, 21.1; **m/z HRMS** (ESI^+) $[M + H]^+$ $C_7H_{10}O^{79}Br^+$ calc. 188.9910, found 188.9911, $C_7H_{10}O^{81}Br^+$ calc. 190.9889, found 190.9891. *Data is consistent with literature*[13]

2-(Methylthio)cyclohex-1-ene-1-carbaldehyde (1f): Prepared according to **general procedure A**, using NaSMe (320 mg, 4.48 mmol, 1.20 equiv.) in DMF (10 mL), then 2-bromocyclohex-1-ene-1-carbaldehyde **S1** (700 mg, 3.71 mmol, 1.00 equiv.) in DMF (10 mL). Work-up using H_2O (2 × 10 mL), EtOAc (3 × 10 mL) and brine (10 mL). Purification by column chromatography (1:19 Et_2O:Petrol) yielded 2-(methylthio)cyclohex-1-ene-1-carbaldehyde **1f** (383 mg, 2.43 mmol, 46%) as a yellow solid.

m.p. (CH_2Cl_2) 48–50 °C; (*Lit:* 45–47 °C);[7] **δ_H** (400 MHz, $CDCl_3$) 10.26 (1H, s, C(6)*H*=O), 2.55-2.48 (2H, m, C(2 or 5)H_2), 2.33 (3H, s, C(1)H_3), 2.29-2.24 (2H, m, C(2 or 5)H_2), 1.77-1.69 (2H, m, C(3 or 4)H_2), 1.66-1.57 (2H, m, C(3 or 4)H_2); **δ_C** (101 MHz, $CDCl_3$) 189.8, 156.9, 135.1, 30.9, 24.1, 23.1, 21.4, 14.2; **m/z HRMS** (ESI^+) $[M + H]^+$ $C_8H_{13}O^{32}S^+$ calc. 157.0682, found 157.0684. *Data is consistent with literature*[7]

2-(Dimethylamino)benzaldehyde (1j)

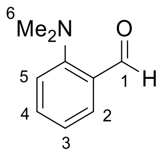

Prepared according to a modified procedure.[14] To a mixture of 2-fluorobenzaldehyde (1.1 mL, 10.0 mmol, 1.00 equiv.) and K_2CO_3 (1.38 g, 10.0 mmol, 1.00 equiv.) in anhydrous DMF (20 mL) at room temperature was added Me_2NH solution (5.60 M in EtOH, 2.4 mL, 13.4 mmol, 1.34 equiv.) The resulting mixture was stirred and heated to 110 °C for 20 h. After this time, the reaction was cooled to room temperature and H_2O (40 mL) was added. The organic layer was separated and the aqueous layer was extracted with EtOAc (3 × 40 mL). The combined organic extracts were then washed with a 5% w/w aq. LiCl solution (40 mL), dried over $MgSO_4$, filtered and concentrated *in vacuo*. Purification by column chromatography (97:3 to 19:1 Petrol:EtOAc) yielded 2-(dimethylamino)benzaldehyde **1j** (1.26 g, 8.45 mmol, 85%) as a yellow oil.

δ_H (400 MHz, $CDCl_3$) 10.22 (1H, s, C(1)*H*), 7.76 (1H, dd, *J* = 7.7, 1.8 Hz, *Ar*), 7.46 (1H, ddd, *J* = 8.3, 7.2, 1.8 Hz, *Ar*), 7.09-6.96 (2H, m, *Ar*), 2.92 (6H, s, C(6)H_3); **δ_C** (101 MHz, $CDCl_3$) 191.3, 155.9, 134.7, 131.1, 127.1, 120.7, 117.7, 45.7; **ν_{max}**/ cm^{-1} (neat) 2981, 2868, 2839, 2794, 1680, 1658, 1596, 1487, 1455, 1433, 1386, 1326, 1279, 1187, 1151, 1137, 1051, 949, 845, 831, 762, 641; **m/z HRMS** (ESI^+) $[M + H]^+$ $C_9H_{12}ON^+$ calc. 150.0913, found 150.0912. *Data is consistent with literature*[14]

3-Hydroxy-2,2-dimethyl-1-morpholinopropan-1-one (S2) and 2,2-dimethyl-3-morpholino-3-oxopropanal (1k)

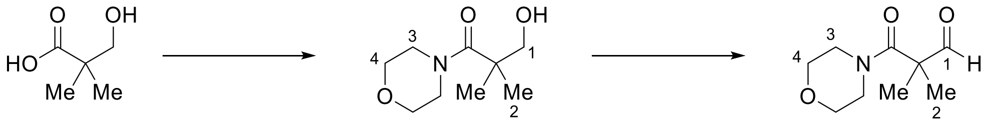

S2 1k

3-Hydroxy-2,2-dimethyl-1-morpholinopropan-1-one (S2): Prepared according to **general procedure B** using 2-hydroxypivalic acid (3.00 g, 25.4 mmol, 1.00 equiv.), CH_2Cl_2 (130 mL), morpholine (2.4 mL, 28.0 mmol, 1.10 equiv.) and EDC.HCl (5.37 g, 28.0 mmol, 1.10 equiv.). Quench using sat. aq. $NaHCO_3$ (70 mL). Work-up using brine (70 mL). Purification by column chromatography (EtOAc) yielded 3-hydroxy-2,2-dimethyl-1-morpholinopropan-1-one **S2** (2.33 g, 12.5 mmol, 49%) as a white solid.

m.p. (CH_2Cl_2) 45–48 °C; $\boldsymbol{\delta_H}$ (400 MHz, $CDCl_3$) 3.71-3.63 (8H, m, C(3 and 4)H_2), 3.61-3.56 (1H, m, O*H*), 3.51-3.49 (2H, m, C(1)H_2), 1.27 (6H, s, C(2)H_3); $\boldsymbol{\delta_C}$ (101 MHz, $CDCl_3$) 176.1, 72.1, 66.5, 45.1, 42.8, 21.7; $\boldsymbol{\nu_{max}}$/ cm^{-1} (neat) 3458, 2863, 2358, 1608, 1425, 1268, 1159, 1115, 1031, 850; **m/z HRMS** (ESI^+) $[M + H]^+$ $C_9H_{18}O_3N^+$ calc. 188.1281, found 188.1281.

2,2-Dimethyl-3-morpholino-3-oxopropanal (1k): Prepared according to a modified literature procedure.[15] CH_2Cl_2 (10 mL) and oxalyl chloride (0.59 mL, 6.82 mmol, 1.10 equiv.) were added to an argon flushed three-neck flask fitted with a thermometer and cooled to –78 °C. In a separate flask under argon, dimethyl sulfoxide (0.88 mL, 12.4 mmol, 2.00 equiv.) was dissolved in CH_2Cl_2 (10 mL). This mixture was added dropwise to the oxalyl chloride solution, ensuring that the temperature did not exceed –65 °C. After addition was complete, the reaction mixture was stirred at this temperature for 5 min. In another flask under argon, 3-hydroxy-2,2-dimethyl-1-morpholinopropan-1-one **S2** (1.16 g, 6.20 mmol, 1.00 equiv.) was dissolved in CH_2Cl_2 (20 mL). This solution was then added dropwise to the first solution, and the mixture

was stirred for 30 min at –78 °C before Et_3N (4.3 mL, 31.0 mmol, 5.00 equiv.) was added dropwise. The mixture was then allowed to warm to room temperature over 30 min before being washed with 2 M $HCl_{(aq)}$ (5.6 mL), sat. aq. $NaHCO_3$ (5.6 mL) and brine (5.6 mL) before being dried over $MgSO_4$, filtered and concentrated *in vacuo*. Purification of the crude residue by column chromatography (1:1 EtOAc:Petrol) yielded 2,2-dimethyl-3-morpholino-3-oxopropanal **1k** (1.09 g, 5.89 mmol, 95%) as a colourless oil.

δ_H (400 MHz, $CDCl_3$) 9.61 (1H, s, C(1)*H*=O), 3.71-3.25 (8H, m, C(3 and 4)*H_2*), 1.37 (6H, s, C(2)*H_3*); **δ_C** (101 MHz, $CDCl_3$) 199.9, 170.0, 66.8, 54.1, 45.0 (bs), 20.6; **ν_{max}**/ cm^{-1} (neat) 2859, 1722, 1638, 1429, 1263, 1115, 1034; **m/z HRMS** (ESI^+) $[M + H]^+$ $C_9H_{16}O_3N^+$ calc. 186.1125, found 186.1126. *Data is consistent with literature*[16]

2-Benzylmalonic acid (S3), 2-benzyl-1,3-dimorpholinopropane-1,3-dione (S4) and 2-benzyl-3-morpholino-3-oxopropanal (1l)

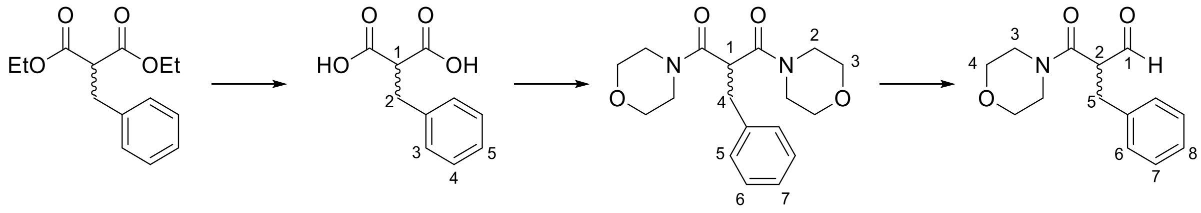

S3 **S4** **1l**

2-Benzylmalonic acid (S3): NaOH pellets (5.00 g, 125 mmol, 2.09 equiv.) was ground with a pestle and mortar and added to a solution of 2-benzyl diethyl malonate (14.1 mL, 59.9 mmol, 1.00 equiv.) in H_2O (300 mL) and stirred over 48 h. Once the reaction was complete by TLC analysis, the mixture was washed with EtOAc (100 mL) to remove any remaining ester. The aqueous layer was then acidified by dropwise addition of 37% w/w aq. HCl was then undertaken until the mixture turned litmus paper red (pH 3). The acidified aqueous layer was then extracted with EtOAc (3 × 100 mL) and the combined organic extracts concentrated *in vacuo* to yield 2-benzylmalonic acid **S3** (11.6 g, 59.4 mmol, 99%) as a white powder.

m.p. (CH_2Cl_2) 108–111 °C; (*Lit:* 110–114 °C);[17] **δ_H** (400 MHz, $CDCl_3$) 7.35-7.20 (5H, m, *Ar*), 3.77 (1H, t, *J* = 7.5 Hz, C(1)*H*), 3.28 (2H, d, *J* = 7.5 Hz, C(2)*H_2*); **δ_C** (101 MHz, MeOD) 172.5, 139.5, 139.5, 129.7, 129.4, 127.5, 54.9, 35.7, 35.6; **ν_{max}**/ cm^{-1} (neat) 3263, 1760, 1661, 1230, 1082; **m/z HRMS** (ESI^-) $[M - H]^-$ $C_{10}H_9O_4^-$ calc. 193.0506, found 193.0504. *Data is consistent with literature[18]*

2-Benzyl-1,3-dimorpholinopropane-1,3-dione (S4): Prepared according to **general procedure B** using di-acid **S3** (1.94 g, 10.0 mmol, 1.00 equiv.), CH_2Cl_2 (50 mL), morpholine (1.90 mL, 22.0 mmol, 2.20 equiv.) and EDC.HCl (4.22 g, 22.0 mmol, 2.20 equiv.). Quench using sat. aq. $NaHCO_3$ (30 mL). Work-up using brine (30 mL). Purification by column chromatography (100:0 to 99:1 EtOAc:Methanol) yielded 2-benzyl-1,3-dimorpholinopropane-1,3-dione **S4** (2.11 g, 6.35 mmol, 64%) as a white solid.

m.p. (CH_2Cl_2) 144–146 °C; **δ_H** (400 MHz, $CDCl_3$) 7.33-7.20 (5H, m, *Ar*), 3.89 (1H, t, *J* = 7.3 Hz, C(1)*H*), 3.73-3.17 (18H, m, C(2 and 3)*H_2* and C(4)*H_2*); **δ_C** (101 MHz, $CDCl_3$) 167.8, 138.8, 129.3, 128.8, 127.0, 66.8, 66.3, 50.5, 46.0, 42.8, 35.9; **ν_{max}**/ cm^{-1} (neat) 3517, 2857, 2362, 1643, 1434, 1237; **m/z HRMS** (ESI^+) $[M + H]^+$ $C_{18}H_{25}O_4N_2^+$ calc. 333.1809, found 333.1809.

2-Benzyl-3-morpholino-3-oxopropanal (1l): A solution of DIBAL-H (1.00 M in CH_2Cl_2, 1.50 mL, 1.50 mmol, 1.00 equiv.) was added dropwise to a solution of bisamide **S4** (500 mg, 1.50 mmol, 1.00 equiv.) in CH_2Cl_2 (20 mL) at -78 ˚C. The mixture was stirred for 2 h at -78 ˚C before being quenched by a sat. aqueous solution of Rochelle's Salt (20 mL). The resulting mixture was allowed to warm to room temperature with stirring for 2 h until a biphasic mixture could be distinguished. The layers were separated and the aqueous solution was extracted with CH_2Cl_2 (3 × 10 mL). The combined organic extracts were dried over $MgSO_4$, filtered and concentrated *in vacuo*. Purification by column chromatography (1:1 EtOAc:Petrol) yielded 2-benzyl-3-morpholino-3-oxopropanal **1l** (320 mg, 1.29 mmol, 86%) as a viscous colourless oil.

δ_H (400 MHz, $CDCl_3$) 9.71 (1H, s, C(1)*H*=O), 7.34-7.16 (5H, m, *Ar*), 3.77-2.86 (11H, m, C(2)*H* and C(3, 4 and 5)H_2); **δ_C** (101 MHz, $CDCl_3$) 198.4, 167.2, 137.5, 129.1, 128.9, 127.2, 66.6, 66.2, 56.6, 46.3, 42.3, 34.5; **ν_{max}**/ cm^{-1} (neat) 3382, 2974, 2923, 2860, 1614, 1447, 1234, 1114, 753, 702; **m/z LRMS** (ESI^+) 270.0 $[M + Na]^+$. *Data is consistent with literature*[16]

3.2 Alkyne substrates

2-((1-Phenylprop-2-yn-1-yl)oxy)ethan-1-ol (2b)

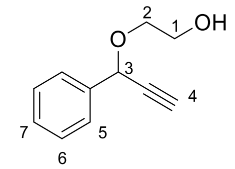

Prepared according to a literature procedure.[19] To a round-bottomed flask was added 1-phenyl-2-propyn-1-ol (0.62 mL, 5.00 mmol, 1.00 equiv.), ethylene glycol (5 mL) and Amberlyst® 15 (1.50 g). The resulting suspension was heated to 80 °C and stirred for 2 h. Once the reaction was complete as monitored by TLC analysis, the suspension was diluted with H_2O (10 mL) and extracted with CH_2Cl_2 (3 × 10 mL). The combined organic extracts were dried over $MgSO_4$, filtered, and concentrated *in vacuo*. The crude residue was then purified by column chromatography (20:1 to 5:1 Petrol:Et_2O) to afford 2-((1-phenylprop-2-yn-1-yl)oxy)ethan-1-ol **2b** (687 mg, 3.90 mmol, 78%) as a pale-yellow oil.

δ_H (400 MHz, $CDCl_3$) 7.55-7.49 (2H, m, *Ar*), 7.43-7.29 (3H, m, *Ar*), 5.23 (1H, d, *J* = 2.2 Hz, C(3)*H*), 3.86-3.56 (4H, m, C(1)H_2 and C(2)H_2), 2.68 (1H, d, *J* = 2.2 Hz, C(4)*H*), 1.91 (1H, br, O*H*); **δ_C** (101 MHz, $CDCl_3$) 137.9, 128.8, 128.7, 127.5, 81.4, 76.2, 71.9, 69.7, 61.9; **ν_{max}**/ cm^{-1} (neat) 3419, 3286, 2931, 2869, 1453, 1059, 1025, 754, 697, 650; **m/z HRMS** (ESI^+) $[M + Na]^+$ $C_{11}H_{12}O_2{}^{23}Na^+$ calc. 199.0730, found 199.0732. *Data is consistent with literature*[19]

Ethyl 2-(but-3-yn-2-yloxy)acetate (S5) and 2-(but-3-yn-2-yloxy)ethan-1-ol (2c)

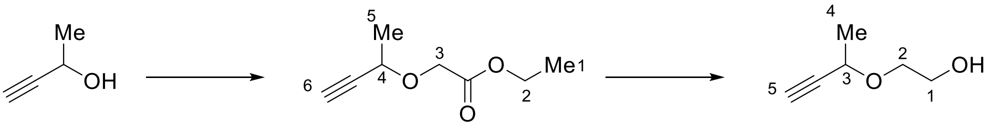

S5 **2c**

Ethyl 2-(but-3-yn-2-yloxy)acetate (S5): Prepared according to **general procedure C** using NaH (60% in mineral oil, 2.04 g, 51.0 mmol, 1.00 equiv.), but-3-yn-2-ol (4.0 mL, 51.0 mmol, 1.00 equiv.), ethyl-2-bromoacetate (6.8 mL, 61.2 mmol, 1.20 equiv.) and DMF (3 × 60 mL). Quench using sat. aq. NH_4Cl (140 mL). Work-up using EtOAc (3 × 100 mL) and 5% aq. LiCl (100 mL). Purification by column chromatography (1:30 to 1:15 Et_2O:Petrol) yielded ethyl 2-(but-3-yn-2-yloxy)acetate **S5** (3.30 g, 21.1 mmol, 41%) as colourless oil.

δ_H (400 MHz, $CDCl_3$) 4.34 (1H, qd, J = 6.8, 2.1 Hz, C(4)H), 4.25-4.08 (4H, m, C(2)H_2 and C(3)H_2), 2.43 (1H, d, J = 2.1 Hz, C(6)H), 1.45 (3H, d, J = 6.6 Hz, C(5)H_3), 1.24 (3H, t, J = 7.1 Hz, C(1)H_3); **δ_C** (101 MHz, $CDCl_3$) 170.2, 82.6, 74.1, 65.5, 65.5, 60.9, 21.9, 14.2; **ν_{max}**/ cm^{-1} (neat) 3296, 1749, 1205, 1118, 1035; **m/z HRMS** (ESI^+) $[M + H]^+$ $C_8H_{13}O_3^+$ calc. 157.0859, found 157.0864. *Data is consistent with literature*[2]

2-(But-3-yn-2-yloxy)ethan-1-ol (2c): Prepared according to **general procedure E** using $LiAlH_4$ (4 M in Et_2O, 2.24 mL, 8.96 mmol, 1.00 equiv.) in Et_2O (10 mL), then 2-(but-3-yn-2-yloxy)acetate **S5** (1.40 g, 8.96 mmol, 1.00 equiv.) in Et_2O (31 mL). Purification by column chromatography (1:9 to 1:1 Et_2O:Petrol) yielded 2-(but-3-yn-2-yloxy)ethan-1-ol **2c** (809 mg, 7.09 mmol, 79%) as a colourless oil.

δ_H (400 MHz, $CDCl_3$) 4.21 (1H, qd, J = 6.6, 2.0 Hz, C(3)H), 3.86-3.80 (1H, m, C(1 or 2)H), 3.78-3.73 (2H, m, C(1 or 2)H_2), 3.56-3.50 (1H, m, C(1 or 2)H), 2.44 (1H, d, J = 2.0 Hz, C(5)H), 2.03 (1H, t, J = 6.2 Hz, OH), 1.46 (3H, d, J = 6.7 Hz, C(4)H_3); **δ_C** (101 MHz, $CDCl_3$) 83.7, 73.3, 70.0, 65.7, 62.0, 22.1; **ν_{max}**/ cm^{-1} (neat) 3379, 3294, 2981, 2361, 2341, 1637, 1460, 1374,

1326, 1252, 1110, 1091, 1058, 948, 890, 850, 802; **m/z HRMS** (ESI^+) $[M + Na]^+$ $C_6H_{10}O_2{}^{23}Na^+$ calc. 137.0573, found 137.0572.

Ethyl 2-(oct-1-yn-3-yloxy)acetate (S6) and 2-(oct-1-yn-3-yloxy)ethan-1-ol (2d)

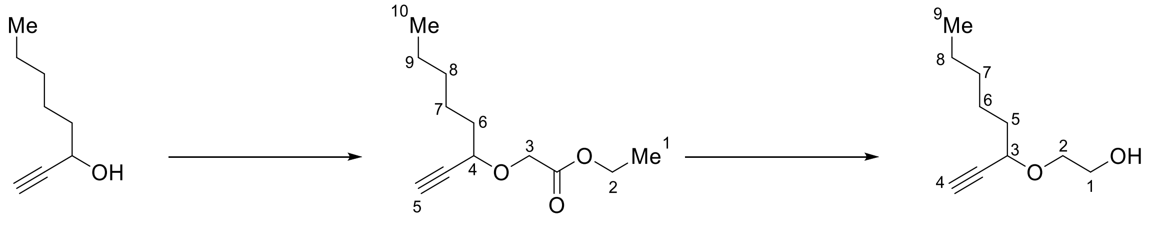

S6 **2d**

Ethyl 2-(oct-1-yn-3-yloxy)acetate (S6): Prepared according to **general procedure C** using NaH (60% in mineral oil, 820 mg, 20.6 mmol, 1.00 equiv.), oct-1-yn-3-ol (3.0 mL, 20.6 mmol, 1.00 equiv.), ethyl-2-bromoacetate (7.6 mL, 68.5 mmol, 2.00 equiv.) and DMF (3 × 50 mL) ***with stirring at 50 °C, overnight (18 h)***. Quench using sat. aq. NH_4Cl (100 mL). Work-up using EtOAc (3 × 50 mL) and 5% aq. LiCl (50 mL). Purification by column chromatography (1:99 to 1:19 Et_2O:Petrol) yielded ethyl 2-(oct-1-yn-3-yloxy)acetate **S6** (1.54 g, 7.26 mmol, 35%) as a colourless oil.

δ_H (400 MHz, $CDCl_3$) 4.29-4.12 (5H, m, C(2)H_2, C(3)H_2 and C(4)H), 2.44 (1H, d, J = 2.1 Hz, C(5)H), 1.84-1.66 (2H, m, C(6)H_2), 1.52-1.43 (2H, m, C(7)H_2), 1.36-1.26 (4H, m, C(8 and 9)H_2), 1.27 (3H, t, J = 7.2 Hz, C(1)H_3), 0.93-0.84 (3H, t, J = 7.0 Hz, C(10)H_3); δ_C (101 MHz, $CDCl_3$) 170.4, 82.0, 74.7, 69.9, 65.6, 60.9, 35.5, 31.6, 24.8, 22.6, 14.3, 14.1; ν_{max}/ cm^{-1} (neat) 3272, 2957, 1752, 1204, 1120, 1029; **m/z HRMS** (ESI^+) $[M + H]^+$ $C_{12}H_{21}O_3{}^+$ calc. 213.1485, found 213.1490.

2-(Oct-1-yn-3-yloxy)ethan-1-ol (2d): Prepared according to **general procedure E** using $LiAlH_4$ (4 M in Et_2O, 0.90 mL, 3.63 mmol, 1.00 equiv.) in Et_2O (10 mL), then 2-(oct-1-yn-3-yloxy)acetate **S6** (770 mg, 3.63 mmol, 1.00 equiv.) in Et_2O (10 mL). Purification by column

chromatography (1:9 to 1:1 Et_2O:Petrol) yielded 2-(oct-1-yn-3-yloxy)ethan-1-ol **2d** (611 mg, 3.59 mmol, 99%) as a colourless oil.

$δ_H$ (400 MHz, $CDCl_3$) 4.06 (1H, td, *J* = 6.6, 2.1 Hz, C(3)*H*), 3.87-3.81 (1H, m, C(2)*H*H), 3.79-3.71 (2H, m, C(1)H_2), 3.52 (1H, m, C(2)H*H*), 2.44 (1H, d, *J* = 2.0 Hz, C(4)*H*), 2.05 (1H, t, *J* = 5.8 Hz, O*H*), 1.82-1.64 (2H, m, C(5)H_2), 1.51-1.39 (2H, m, C(6)H_2), 1.37-1.24 (4H, m, C(7 and 8)H_2), 0.89 (3H, t, *J* = 6.8 Hz, C(9)H_3); **$δ_C$** (101 MHz, $CDCl_3$) 83.0, 74.0, 70.1, 70.1, 62.0, 35.7, 31.6, 25.0, 22.7, 14.1; **$ν_{max}$**/ cm^{-1} (neat) 3426, 3272, 2957, 2112, 1466, 1379, 1274, 1204, 1121, 1029, 944, 668; **m/z HRMS** (ESI^+) $[M + Na]^+$ $C_{10}H_{18}O_2{}^{23}Na^+$ calc. 193.1199, found 193.1201.

Ethyl 2-(prop-2-yn-1-yloxy)propanoate (S7) and 2-(prop-2-yn-1-yloxy)propan-1-ol (2e)

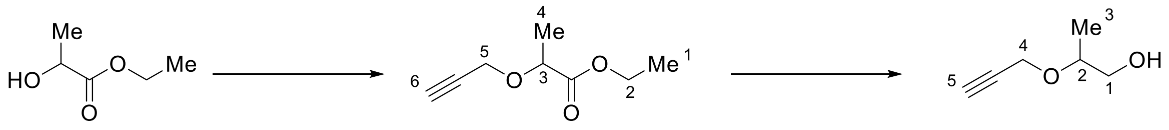

S7 **2e**

Ethyl 2-(prop-2-yn-1-yloxy)propanoate (S7): Prepared according to **general procedure D** using ethyl lactate (4.0 mL, 34.9 mmol, 1.00 equiv.), DMF (190 mL), NaH (60% in mineral oil, 1.46 g, 36.6 mmol, 1.05 equiv.) and propargyl bromide (80% w/w in toluene, 15.5 mL, 139 mmol, 4.00 equiv.) with stirring for 2.5 h. Quench using sat. aq. NH_4Cl (190 mL). Work-up using EtOAc (3 × 190 mL) and 5% aq. LiCl (190 mL). Purification by column chromatography (98:2 to 19:1 Petrol:EtOAc) yielded ethyl 2-(prop-2-yn-1-yloxy)propanoate **S7** (2.10 g, 13.45 mmol, 39%) as a pale-yellow oil.

$δ_H$ (400 MHz, $CDCl_3$) 4.39-4.10 (5H, m, C(2)H_2, C(3)*H* and C(5)H_2), 2.44 (1H, t, *J* = 2.4 Hz, C(6)*H*), 1.43 (3H, d, *J* = 6.9 Hz, C(4)H_3), 1.29 (3H, t, *J* = 7.1 Hz, C(1)H_3); **$δ_C$** (101 MHz, $CDCl_3$) 172.8, 79.2, 75.1, 73.3, 61.1, 57.2, 18.6, 14.3; **$ν_{max}$**/ cm^{-1} (neat) 2955, 2859, 1742, 1468,

1377, 1254, 1145, 1093, 1093, 1049, 981, 832, 775; **m/z HRMS** (ESI^+) $[M + Na]^+$ $C_8H_{12}O_3{}^{23}Na^+$ calc. 179.0679, found 179.0679. *Data is consistent with literature*[20]

2-(Prop-2-yn-1-yloxy)propan-1-ol (2e): Prepared according to **general procedure E** using $LiAlH_4$ (4M in Et_2O, 1.6 mL, 6.40 mmol, 1.00 equiv.) in Et_2O (10 mL), then ethyl 2-(prop-2-yn-1-yloxy)propanoate **S7** (1.00 g, 6.40 mmol, 1.00 equiv.) in Et_2O (22 mL). Purification by column chromatography (9:1 to 1:1 Petrol:Et_2O) yielded 2-(prop-2-yn-1-yloxy)propan-1-ol **2e** (263 mg, 2.31 mmol, 36%) as a pale-yellow oil.

$\boldsymbol{\delta_H}$ (400 MHz, $CDCl_3$) 4.22 (1H, dd, *J* = 15.9, 2.4 Hz, C(4)*H*H), 4.11 (1H, dd, *J* = 15.9, 2.4 Hz, C(4)H*H*), 3.78-3.64 (1H, m, C(2)*H*), 3.56 (1H, ddd, *J* = 11.3, 7.9, 3.2 Hz, C(1)*H*H), 3.43 (1H, ddd, *J* = 11.6, 7.1, 4.3 Hz, C(1)H*H*), 2.38 (1H, t, *J* = 2.4 Hz, C(5)*H*), 1.96 (1H, dd, *J* = 8.0, 4.6 Hz, O*H*), 1.09 (3H, d, *J* = 6.3 Hz, C(3)H_3); $\boldsymbol{\delta_C}$ (101 MHz, $CDCl_3$) 80.2, 75.7, 74.4, 66.4, 56.3, 15.7; $\boldsymbol{\nu_{max}}$/ cm^{-1} (neat) 3288, 2977, 2888, 1456, 1380, 1065, 664; **m/z HRMS** (ESI^+) $[M + Na]^+$ $C_6H_{10}O_2{}^{23}Na^+$ clac. 137.0573, found 137.0574. *Data is consistent with literature*[21]

Ethyl 2-(prop-2-yn-1-yloxy)hexanoate (S8) and 2-(prop-2-yn-1-yloxy)hexan-1-ol (2f)

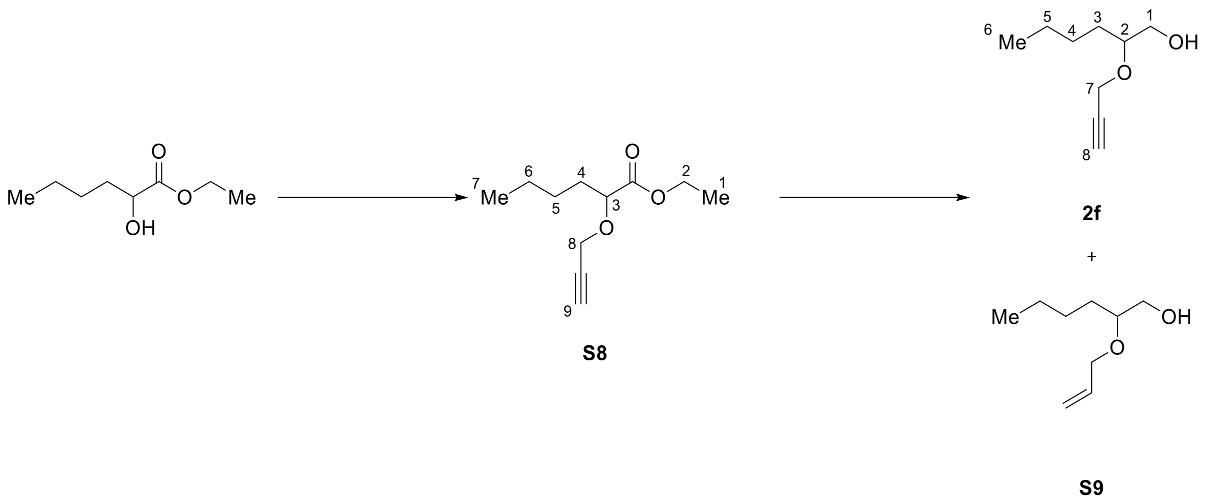

Ethyl 2-(prop-2-yn-1-yloxy)hexanoate (S8): Prepared according to **general procedure D** using ethyl 2-hydroxyhexanoate (2.1 mL, 12.5 mmol, 1.00 equiv.), DMF (70 mL), NaH (60% in mineral oil, 524 mg, 13.1 mmol, 1.05 equiv.) and propargyl bromide (80% w/w in toluene, 5.6 mL, 50.0 mmol, 4.00 equiv.) ***with stirring overnight (18 h)***. Quench using sat. aq. NH_4Cl (70 mL). Work-up using EtOAc (3 × 70 mL) and 5% aq. LiCl (70 mL). Purification by column chromatography (1:99 to 1:49 EtOAc:Petrol) yielded ethyl 2-(prop-2-yn-1-yloxy)hexanoate **S8** (700 mg, 3.53 mmol, 28%), as a pale-yellow oil.

$\boldsymbol{\delta_H}$ (400 MHz, $CDCl_3$) 4.32 (1H, dd, *J* = 16.0, 2.4 Hz, C(8)*H*H), 4.24-4.16 (2H, qd, *J* = 7.1, 3.5 Hz, C(2)H_2), 4.14 (1H, dd, *J* = 16.0, 2.3 Hz, C(8)H*H*) 4.10 (1H, dd, *J* = 6.5, 5.2 Hz, C(3)*H*), 2.42 (1H, t, *J* = 2.4 Hz, C(9)*H*), 1.79-1.68 (2H, m, C(4)H_2), 1.44-1.25 (4H, m, C(5 and 6)H_2), 1.28 (3H, t, *J* = 7.1 Hz, C(1)H_3), 0.88 (3H, t, *J* = 7.1 Hz, C(7)H_3); $\boldsymbol{\delta_C}$ (101 MHz, $CDCl_3$) 172.6, 79.2, 77.2, 75.0, 61.0, 57.4, 32.6, 27.3, 22.5, 14.4, 14.0; $\boldsymbol{\nu_{max}}$/ cm^{-1} (neat) 3274, 2958, 2361, 1745, 1457, 1195, 1123, 1100, 1026; **m/z HRMS** (ESI^+) $[M + H]^+$ $C_{11}H_{19}O_3^+$ calc. 199.1329, found 199.1330.

2-(Prop-2-yn-1-yloxy)hexan-1-ol (2f): Prepared according to **general procedure E** using $LiAlH_4$ (4M in Et_2O, 0.88 mL, 3.53 mmol, 1.00 equiv.) in Et_2O (6 mL), then ethyl 2-(prop-2-

yn-1-yloxy)hexanoate **S8** (700 mg, 3.53 mmol, 1.00 equiv.) in Et_2O (6 mL). Purification by column chromatography (7:3 Petrol:Et_2O) yielded 2-(prop-2-yn-1-yloxy)hexan-1-ol **2f** as a 9:1 inseparable mixture with 2-(allyloxy)hexan-1-ol side product **S9** (374 mg, 2.40 mmol, 68%) as a pale-yellow oil.

δ_H (400 MHz, $CDCl_3$) 4.26 (1H, dd, *J* = 15.9, 2.4 Hz, C(7)*H*H), 4.18 (1H, dd, *J* = 15.9, 2.4 Hz, C(7)H*H*), 3.70-3.63 (1H, m, C(1)*H*H), 3.66-3.42 (2H, m, C(1)H*H* and C(2)*H*), 2.43 (1H, t, *J* = 2.4 Hz, C(8)*H*), 2.19 (1H, dd, *J* = 7.0, 5.1 Hz, O*H*), 1.60-1.49 (1H, m, C(3)*H*H), 1.49-1.39 (1H, m, C(3)H*H*), 1.38-1.20 (4H, m, C(4 and 5)H_2), 0.95-0.81 (3H, m, C(6)H_3); **δ_C** (101 MHz, $CDCl_3$) 80.4, 80.0, 74.3, 64.4, 57.0, 30.4, 27.6, 22.9, 14.0; **ν_{max}**/ cm^{-1} (neat) 3406, 3309, 2932, 2862, 2361, 1458, 1345, 1082, 919, 667; **m/z HRMS** (ESI^+) $[M + Na]^+$ $C_9H_{16}O_2{}^{23}Na^+$ calc. 179.1048, found 179.1044.

2-Phenyl-2-(prop-2-yn-1-yloxy)ethan-1-ol (2g)

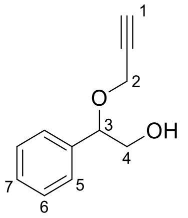

Prepared according to **general procedure F**, using styrene oxide (1.4 mL, 12.5 mmol, 1.00 equiv.), propargyl alcohol (1.4 mL, 25.0 mmol, 2.00 equiv.), $Yb(OTf)_3$ (78.0 mg, 0.130 mmol, 0.010 equiv.) and CH_2Cl_2 (87 mL). Workup with H_2O (100 mL) and CH_2Cl_2 (3 × 50 mL). Purification by column chromatography (1:49 to 1:5 EtOAc:Petrol) yielded 2-phenyl-2-(prop-2-yn-1-yloxy)ethan-1-ol **2g** (442 mg, 2.51 mmol, 20%) as a yellow oil.

δ_H (400 MHz, $CDCl_3$) 7.35-7.28 (2H, m, *Ar*), 7.37-7.21 (3H, m, *Ar*), 4.68 (1H, dd, *J* = 8.4, 3.7 Hz, C(3)H), 4.20 (1H, dd, *J* = 15.7, 2.3 Hz, C(2)*H*H), 3.96 (1H, dd, *J* = 15.7, 2.4 Hz, C(2)H*H*), 3.79-3.71 (1H, m, C(4)*H*H), 3.68-3.60 (1H, m, C(4)H*H*), 2.61-2.54 (1H, m, O*H*), 2.45 (1H, t, *J* = 2.4 Hz, C(1)*H*); **δ_C** (101 MHz, $CDCl_3$) 137.4, 128.7, 128.5, 127.2, 81.8, 79.6, 74.8, 67.1,

56.1; **ν_{max}**/ cm^{-1} (neat) 3304, 1086, 1065, 1041, 906, 727, 701, 647, **m/z HRMS** (ESI^+) [M + $Na]^+$, $C_{11}H_{12}O_2{}^{23}Na^+$ calc. 199.0730, found 199.0732.

2-Vinylnapthalene oxide (S10) and 2-(naphthalen-2-yl)-2-(prop-2-yn-1-yloxy)ethan-1-ol (2h)

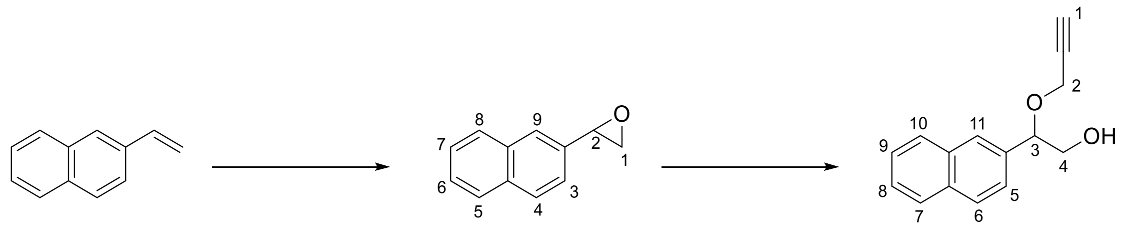

S10 **2h**

2-Vinylnapthalene oxide (S10): Prepared according to a literature procedure.[22] 2-vinylnapthalene (2.00 g, 13.0 mmol, 1.00 equiv.), and $NaHCO_3$ (1.14 g, 13.6 mmol, 1.15 equiv.) were dissolved in CH_2Cl_2 (65 mL) at 0 °C, and *m*-CPBA (75% w/w, 3.45 g, 15.0 mmol, 1.15 equiv.) was added portion-wise. The resulting mixture was warmed to room temperature and stirred under N_2 for 48 h. The solution was then washed with 5% w/w aq. $NaHSO_3$ solution (2 × 50 mL) and sat. aq. $NaHCO_3$ (4 × 40 mL), dried over $MgSO_4$, filtered and concentrated *in vacuo*. Purification of the crude residue by column chromatography (1:19 EtOAc:Petrol) yielded 2-vinylnapthalene oxide **S10** (1.23 g, 7.28 mmol, 56%) as a white powdered solid.

m.p. (CH_2Cl_2) 56–58 °C; (*Lit:* 57–58 °C);[23] **δ_H** (400 MHz, $CDCl_3$) 7.88-7.78 (4H, m, *Ar*), 7.54-7.44 (2H, m, *Ar*), 7.34 (1H, dd, *J* = 8.5, 1.8 Hz, *Ar*), 4.04 (1H, dd, *J* = 4.1, 2.5 Hz, C(2)*H*), 3.23 (1H, dd, *J* = 5.4, 4.1 Hz, C(1)*H*H), 2.92 (1H, dd, *J* = 5.5, 2.6 Hz, C(1)H*H*); **δ_C** (101 MHz, $CDCl_3$) 135.2, 133.5, 133.3, 128.5, 127.9, 126.5, 126.2, 125.3, 122.8, 52.7, 51.4. *Data is consistent with literature[22]*

2-(Naphthalen-2-yl)-2-(prop-2-yn-1-yloxy)ethan-1-ol (2h): Prepared according to **general procedure F**, using 2-vinylnapthalene oxide **S10** (750 mg, 4.40 mmol, 1.00 equiv.), propargyl alcohol (0.5 mL, 8.80 mmol, 2.00 equiv.), $Yb(OTf)_3$ (27.0 mg, 0.040 mmol, 0.010 equiv.) and

CH_2Cl_2 (30 mL), ***with stirring at 30 °C***. Workup with H_2O (40 mL) and CH_2Cl_2 (3 × 20 mL). Purification by column chromatography (1:9 to 1:7 EtOAc:Petrol) yielded 2-(naphthalen-2-yl)-2-(prop-2-yn-1-yloxy)ethan-1-ol **2h** (530 mg, 2.35 mmol, 53%) as a white powdered solid.

m.p. (CH_2Cl_2) 58–60 °C; **δ_H** (400 MHz, $CDCl_3$) 7.94-7.77 (4H, m, *Ar*), 7.58-7.40 (3H, m, *Ar*), 4.85 (1H, dd, *J* = 8.2, 3.9 Hz, C(3)*H*), 4.26 (1H, dd, *J* = 15.7, 2.4 Hz, C(2)*H*H), 4.01 (1H, dd, *J* = 15.7, 2.4 Hz, C(2)H*H*), 3.94-3.65 (2H, m, C(4)H_2), 2.46 (1H, t, *J* = 2.4 Hz, C(1)*H*), 2.25 (1H, dd, *J* = 9.3, 3.9 Hz, O*H*); **δ_C** (101 MHz, $CDCl_3$) 134.9, 133.6, 133.3, 128.8, 128.1, 127.9, 126.8, 126.5, 126.4, 124.6, 82.0, 79.7, 74.9, 67.1, 56.3; **ν_{max}**/ cm^{-1} (neat) 3413, 3289, 3055, 2860, 2100, 1601, 1508, 1442, 1361, 1328, 1271, 1097, 1075, 1043, 859, 821, 748, 673; **m/z HRMS** (ESI^+) $[M + Na]^+$ $C_{15}H_{14}O_2{}^{23}Na^+$ calc. 249.0886, found 249.0887.

2-(prop-2-yn-1-yloxy)-2-(4-(trifluoromethyl)phenyl)ethan-1-ol (2i)

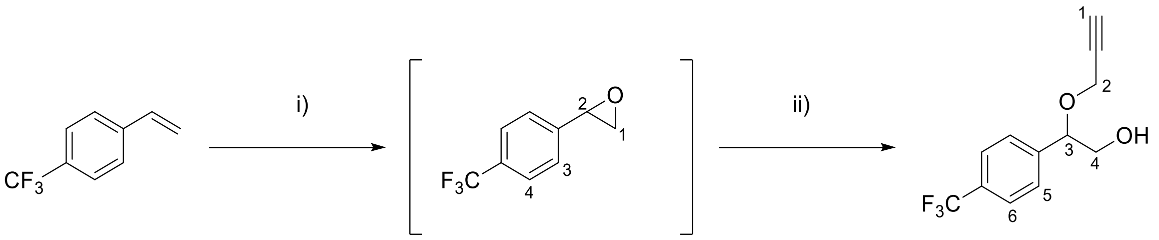

2i

Step i): Prepared according to a literature procedure.[24] To a solution of trifluoromethyl styrene (0.7 mL, 4.74 mmol, 1.00 equiv.) in CH_2Cl_2 (21 mL), was added *m*-CPBA (75% w/w, 1.64 g, 7.11 mmol, 1.50 equiv.) at 0 °C. The reaction mixture was then warmed to room temperature and stirred under N_2 for 18 h. Once complete by TLC analysis, the reaction mixture was quenched with sat. aq. $NaHSO_3$ (20 mL) and the layers were separated. The aqueous phase was then extracted with CH_2Cl_2 (3 × 20 mL). The combined organic extracts were washed with sat. aq. $NaHCO_3$ (20 mL), dried over $MgSO_4$, filtered and concentrated *in vacuo* to yield crude 2-(4-(trifluoromethyl)phenyl)oxirane (874 mg) as a colourless oil. **Step ii):** Prepared according to **general procedure F**, using crude 2-(4-(trifluoromethyl)phenyl)oxirane (874 mg, 4.65 mmol, 1.00 equiv.), propargyl alcohol (0.57 mL, 9.30 mmol, 2.00 equiv.), $Yb(OTf)_3$ (29 mg,

0.050 mmol, 0.010 equiv.) and CH_2Cl_2 (30 mL). Workup with H_2O (40 mL) and CH_2Cl_2 (3 × 20 mL). Purification by column chromatography (1:49 to 1:9 EtOAc:Petrol) yielded 2-(prop-2-yn-1-yloxy)-2-(4-(trifluoromethyl)phenyl)ethan-1-ol **2i** (455 mg, 1.86 mmol, 39% over two steps) as a yellow oil.

δ_F (377 MHz, $CDCl_3$) -62.64; **δ_H** (400 MHz, $CDCl_3$) 7.63 (2H, d, J = 8.0 Hz, C(6)H), 7.46 (2H, d, J = 8.0 Hz, C(5)H), 4.74 (1H, dd, J = 7.8, 3.9 Hz, C(3)H), 4.23 (1H, dd, J = 15.8, 2.4 Hz, C(2)HH), 3.98 (1H, dd, J = 15.7, 2.4 Hz, C(2)HH), 3.78-3.60 (2H, m, C(4)H_2), 2.53 (1H, br, OH), 2.46 (1H, t, J = 2.4 Hz, C(1)H); **δ_C** (101 MHz, $CDCl_3$) 141.8, 130.8 (q, J_{CF} = 32.5 Hz), 127.6, 125.8 (q, J_{CF} = 3.8 Hz), 124.1 (q, J_{CF} = 272.4 Hz), 81.3, 79.2, 75.3, 67.0, 56.6; **ν_{max}**/ cm^{-1} (neat) 3307, 2923, 2360, 1620, 1420, 1326, 1166, 1125, 1067, 1019, 841, 669; **m/z HRMS** (ESI^+) $[M + Na]^+$, $C_{12}H_{11}O_2F_3{}^{23}Na^+$ calc. 267.0603, found 267.0604.

(3-Phenyloxiran-2-yl)methanol (S11), 2-phenyl-3-((prop-2-yn-1-yloxy)methyl)oxirane (S12), 1-phenyl-3-(prop-2-yn-1-yloxy)propan-2-one (S13), 1-phenyl-3-(prop-2-yn-1-yloxy)propan-2-ol (2j)

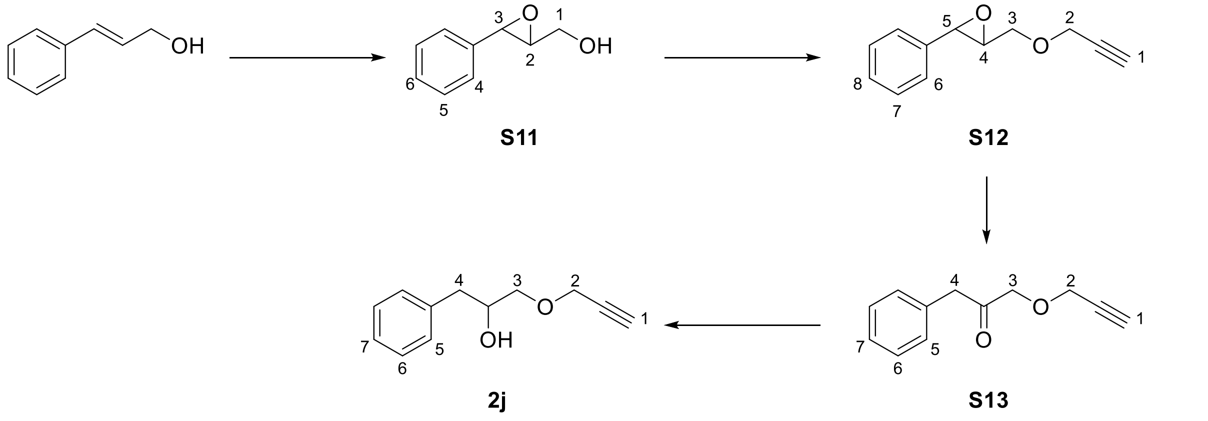

(3-Phenyloxiran-2-yl)methanol (S11): Prepared according to a literature procedure.[25] *m*-CPBA (75% w/w, 20.0 g, 89.4 mmol, 1.50 equiv.) was added to a stirred solution of (*E*)-cinnamyl alcohol (7.7 mL, 59.6 mmol, 1.00 equiv.) in CH_2Cl_2 (300 mL) at 0 °C. The reaction was stirred at room temperature for 2 h. Once complete by TLC analysis, sat. aq. Na_2SO_3 (100

mL) was added to quench excess *m*-CPBA. Additional CH_2Cl_2 (100 mL) was added to the reaction mixture and the two layers were separated. The organic layer was washed with sat. aq. $NaHCO_3$ (100 mL), brine (100 mL), dried over Na_2SO_4, filtered and concentrated *in vacuo*. Purification of the crude residue by column chromatography (1:9 to 3:2 EtOAc:Petrol) yielded (3-phenyloxiran-2-yl)methanol **S11** (6.71 g, 44.7 mmol, 75%), as a colourless oil.

δ_H (400 MHz, $CDCl_3$) 7.43-7.25 (5H, m, *Ar*), 4.05 (1H, ddd, *J* = 12.8, 5.2, 2.4 Hz, C(1)*H*H), 3.93 (1H, d, *J* = 2.2 Hz, C(3)*H*), 3.81 (1H, ddd, *J* = 12.7, 7.7, 3.8 Hz, C(1)H*H*), 3.24-3.21 (1H, m, C(2)*H*), 1.94 (1H, br, O*H*); **δ_C** (101 MHz, $CDCl_3$) 136.8, 128.6, 128.5, 125.9, 62.6, 61.3, 55.7; **ν_{max}**/ cm^{-1} (neat) 3400, 1459, 1074, 1025, 874, 759, 698; **m/z HRMS** (ESI^+) $[M + Na]^+$ $C_9H_{10}O_2{}^{23}Na^+$ calc. 173.0573, found 173.0576. *Data is consistent with literature[25]*

2-Phenyl-3-((prop-2-yn-1-yloxy)methyl)oxirane (S12): Prepared according to a literature procedure.[25] NaH (60% dispersion in mineral oil, 1.40 g, 35.0 mmol, 1.50 equiv) was added to a dry two-neck round bottomed flask under argon, followed by anhydrous THF (65 mL). The reaction flask was then cooled to 0 °C. To this, a solution of (3-phenyloxiran-2-yl)methanol **S11** (3.50 g, 23.3 mmol, 1.00 equiv.) in THF (65 mL) was added dropwise. The reaction mixture was allowed to stir for 30 min at 0 °C, and then propargyl bromide (80% w/w in toluene, 3.1 mL, 28.0 mmol, 1.20 equiv.) in THF (6 mL) was added dropwise. The reaction mixture was warmed to room temperature and stirred for 2 h. Once the reaction was complete by TLC analysis, the excess NaH was quenched by adding EtOAc (10 mL). The solvent was removed from the reaction mixture, and the residue was dissolved in EtOAc (150 mL). The solution was washed with H_2O (150 mL) and brine (150 mL), dried over anhydrous Na_2SO_4, filtered and concentrated *in vacuo*. Purification of the crude residue by column chromatography (3:97 to 1:24 EtOAc:Petrol) yielded 2-phenyl-3-((prop-2-yn-1-yloxy)methyl)oxirane **S12** (4.28 g, 22.9 mmol, 98%) as a colourless oil.

δ_H (400 MHz, $CDCl_3$) 7.39-7.25 (5H, m, *Ar*), 4.26 (2H, d, *J* = 2.3 Hz, C(2)*H_2*), 3.92 (1H, dd, *J* = 11.4, 3.1 Hz, C(3)*H*H), 3.82 (1H, d, *J* = 2.0 Hz, C(5)*H*), 3.70 (1H, dd, *J* = 11.4, 5.3 Hz, C(3)H*H*), 3.24 (1H, ddd, *J* = 5.2, 3.1, 2.1 Hz, C(4)*H*), 2.48 (1H, t, *J* = 2.4 Hz, C(1)*H*); **δ_C** (101 MHz, $CDCl_3$) 136.8, 128.6, 128.5, 125.8, 79.3, 75.1, 69.4, 60.8, 58.6, 56.0; **ν_{max}**/ cm^{-1} (neat) 3286, 2100, 1456, 1357, 1100, 876, 758, 692; **m/z HRMS** (ESI^+) $[M + Na]^+$ $C_{12}H_{12}O_2{}^{23}Na^+$ calc. 211.0730, found 211.0730. *Data is consistent with literature[25]*

1-Phenyl-3-(prop-2-yn-1-yloxy)propan-2-one (S13): Prepared according to a modified procedure.[25] $Cu(OTf)_2$ (77.0 mg, 0.210 mmol, 0.020 equiv.) was added to a solution of 2-phenyl-3-((prop-2-yn-1-yloxy)methyl)oxirane **S12** (2.00 g, 10.6 mmol, 1.00 equiv.) in CH_2Cl_2 (70 mL) and the reaction mixture was stirred for 2 h at room temperature. Once complete by TLC analysis, the solvent was removed *in vacuo*. Purification of the crude residue by column chromatography (1:9 EtOAc:Petrol) yielded 1-phenyl-3-(prop-2-yn-1-yloxy)propan-2-one **S13** (1.06 g, 5.63 mmol, 53%), as a colourless oil.

δ_H (400 MHz, $CDCl_3$) 7.37-7.20 (5H, m, *Ar*), 4.24 (2H, d, *J* = 2.4 Hz, C(2)*H_2*), 4.22 (2H, s, C(3 or 4)*H_2*), 3.78 (2H, s, C(3 or 4)*H_2*), 2.46 (1H, t, *J* = 2.4 Hz, C(1)*H*); **δ_C** (101 MHz, $CDCl_3$) 205.4, 133.3, 129.6, 128.9, 127.3, 78.6, 75.8, 73.6, 58.5, 46.4; **m/z HRMS** (ESI^+) $[M + Na]^+$ $C_{12}H_{12}O_2{}^{23}Na^+$ calc. 211.0730, found 211.0729. *Data is consistent with literature[25]*

1-Phenyl-3-(prop-2-yn-1-yloxy)propan-2-ol (2j): Prepared according to a modified procedure.[26] To a solution of 1-phenyl-3-(prop-2-yn-1-yloxy)propan-2-one **S13** (400 mg, 2.13 mmol, 1.00 equiv.) in anhydrous THF (25 mL) was added $NaBH_4$ (240 mg, 6.40 mmol, 3.00 equiv.) at 0 °C. The reaction mixture was then warmed to room temperature and stirred for 2 h. Once complete by TLC analysis, H_2O (25 mL) was added and the layers were separated. The aqueous phase was then extracted with CH_2Cl_2 (3 × 25 mL). The combined organic extracts were washed with brine (25 mL), dried over Na_2SO_4, filtered and concentrated *in*

vacuo. Purification of the crude residue by column chromatography (1:19 EtOAc:Petrol) yielded 1-phenyl-3-(prop-2-yn-1-yloxy)propan-2-ol **2j** (262 mg, 1.38 mmol, 65%) as a colourless oil.

δ_H (400 MHz, $CDCl_3$) 7.35-7.21 (5H, m, *Ar*), 4.19 (2H, d, *J* = 2.4 Hz, C(2)H_2), 4.10-4.00 (1H, m, C(4)*H*), 3.58 (1H, dd, *J* = 9.4, 3.4 Hz, C(3 or 5)*H*H), 3.45 (1H, dd, *J* = 9.4, 6.9 Hz, C(3 or 5)H*H*), 2.82 (2H, d, *J* = 6.7 Hz, C(3 or 5)H_2), 2.45 (1H, t, *J* = 2.4 Hz, C(1)*H*), 2.32 (1H, d, *J* = 4.0 Hz, O*H*); **δ_C** (101 MHz, $CDCl_3$) 137.9, 129.5, 128.6, 126.6, 79.6, 74.9, 73.4, 71.4, 58.7, 39.9; **ν_{max}**/ cm^{-1} (neat) 3423, 3288, 3288, 2916, 2100, 1092, 744, 695; **m/z HRMS** (ESI^+) $[M + Na]^+$ $C_{12}H_{14}O_2{}^{23}Na^+$ calc. 213.0886, found 213.0885.

(2*S,3*S**)-3-(Prop-2-yn-1-yloxy)butan-2-ol (2k)**

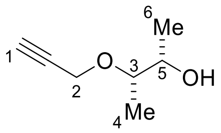

Prepared according to **general procedure D**, using (2*S**,3*S**)-butan-1,2-diol (0.40 mL, 4.45 mmol, 1.00 equiv.) in DMF (20 mL), NaH (60% dispersion in mineral oil, 178 mg, 4.45 mmol, 1.00 equiv.) and propargyl bromide (80% w/w in toluene 1.98 mL, 17.8 mmol, 4.00 equiv. with stirring at room temperature for 2 h. Quench using sat. aq. NH_4Cl (20 mL) work-up using EtOAc (3 × 20 mL), 5% aq. LiCl (20 mL). Purification by column chromatography (1:9 to 1:4 EtOAc:Petrol) yielded (2*S**,3*S**)-3-(prop-2-yn-1-yloxy)butan-2-ol **2k** (209 mg, 1.63 mmol, 37%) as a yellow oil.

δ_H (400 MHz, $CDCl_3$) 4.18 (1H, dd, *J* = 15.9, 2.4 Hz, C(2)*H*H), 4.06 (1H, dd, *J* = 15.9, 2.4 Hz, C(2)H*H*), 3.50 (1H, app p, *J* = 6.4 Hz, C(3 or 5)*H*), 3.38-3.24 (1H, app p, C(3 or 5)*H*), 2.82 (1H, bs, O*H*), 2.38 (1H, t, *J* = 2.4 Hz, C(1)*H*), 1.05 (3H, d, *J* = 6.4 Hz, C(4 or 6)H_3), 1.03 (3H, d, *J* = 6.3 Hz, C(4 or 6)H_3); **δ_C** (101 MHz, $CDCl_3$) 80.0, 79.6, 74.4, 70.7, 56.2, 18.40, 15.0;

ν_{max}/ cm^{-1} (neat) 3418, 3291, 2980, 2889, 2200, 1741 (br), 1522, 1381, 1346, 1263, 1084, 1019, 923, 697, 670, **m/z HRMS** (ESI$^+$) [M + Na]$^+$ $C_7H_{12}O_2{}^{23}Na^+$ calc. 151.0730, found 151.0728.

(1*S*,2*S*)- 2-(Prop-2-yn-1-yloxy)cyclohexan-1-ol (2l)

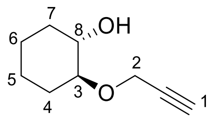

Prepared according to **general procedure D**, using (1*S*,2*S*) -cyclohexane-1,2-diol (300 mg, 2.58 mmol. 1.00 equiv.) in DMF (20 mL), NaH (60%, 103 mg, 2.58 mmol, 1.00 equiv.) and propargyl bromide (80% w/w in toluene, 1.2 mL, 10.3 mmol, 4.00 equiv.) with stirring at room temperature for 2 h. Quench using sat. aq. NH_4Cl (20 mL), and workup using EtOAc (3 × 20 mL), 5% aq. LiCl (20 mL). Purification of the crude residue by column chromatography (1:19 to 1:4 EtOAc:Petrol) yielded (1*S*,2*S*)- 2-(prop-2-yn-1-yloxy)cyclohexan-1-ol **2l** (167 mg, 1.08 mmol, 42%) as a yellow oil.

δ_H (400 MHz, $CDCl_3$) 4.29 (1H, dd, *J* = 15.9, 2.4 Hz, C(2)*H*H), 4.18 (1H, dd, *J* = 15.8, 2.4 Hz, C(2)H*H*), 3.49-3.40 (1H, m, C(3 or 8)*H*), 3.29-3.21 (1H, m, C(3 or 8)*H*), 2.69 (1H, br, O*H*), 2.44 (1H, t, *J* = 2.4 Hz, C(1)*H*), 2.12-2.05 (1H, m, C(4 or 7)*H*H), 2.04-1.98 (1H, m, C(4 or 7)*H*H), 1.77-1.64 (2H, m, C(5 or 6)H_2), 1.35-1.09 (4H, m, C(4 and 7)H*H* and C(5 or 6)H_2); **δ_C** (101 MHz, $CDCl_3$) 83.1, 80.4, 74.4, 73.7, 56.3, 32.3, 29.1, 24.3, 24.0; **ν_{max}**/ cm^{-1} (neat) 3295, 2934, 2862, 1451, 1082, 1015, 847, **m/z HRMS** (ESI$^+$) [M + Na]$^+$ $C_9H_{14}O_2{}^{23}Na^+$ calc. 177.0886, found 177.0887; $[\alpha]_D^{25}$ = +79.1 ° (c = 1.0, $CHCl_3$).

Methyl prop-2-yn-1-ylleucinate (S14), Methyl *N*-(methylsulfonyl)-*N*-(prop-2-yn-1-yl)leucinate (S15) and *N*-(1-hydroxy-4-methylpentan-2-yl)-*N*-(prop-2-yn-1-yl)methanesulfonamide (2m)

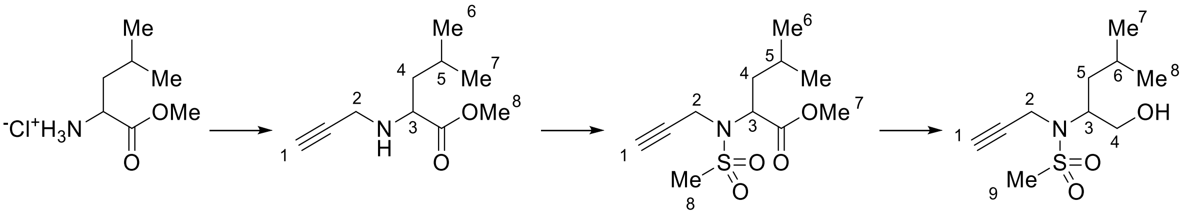

Methyl prop-2-yn-1-ylleucinate (S14): Prepared according to a modified literature procedure.[27] Propargyl bromide (80% in toluene, 2.9 mL, 30.6 mmol, 1.23 equiv.) was added dropwise to a suspension of leucine hydrochloride methyl ester (4.50 g, 24.8 mmol, 1.00 equiv.) and K_2CO_3 (7.20 g, 52.1 mmol, 2.10 equiv.) in anhydrous DMF (60 mL) under N_2. The resulting mixture was left to stir at room temperature for 48 h before H_2O (100 mL) was added. The mixture was extracted with CH_2Cl_2 (3 × 100 mL), and the combined organic extracts were dried over $MgSO_4$, filtered, and concentrated *in vacuo*. Purification of the crude residue by column chromatography (1:9 EtOAc:Petrol) yielded methyl prop-2-yn-1-ylleucinate **S14** (3.39 g, 18.5 mmol, 75%) as a colourless oil.

δ_H (400 MHz, $CDCl_3$) 3.67 (3H, s, OC(8)H_3), 3.44-3.37 (2H, m, C(2)*H*H, C(3)*H*), 3.30 (1H, dd, *J* = 16.8, 2.3 Hz, C(2)H*H*), 2.16 (1H, app t, *J* = 2.5 Hz, C(1)*H*), 1.76-1.62 (2H, m, C(5)*H*, N*H*), 1.50-1.36 (2H, m, C(4)H_2), 0.88 (3H, d, *J* = 3.6 Hz, C(6 or 7)H_3), 0.87 (3H, d, *J* = 3.5 Hz, C(6 or 7)H_3); **δ_C** (101 MHz, $CDCl_3$) 175.9, 81.6, 71.6, 58.6, 51.7, 42.6, 37.0, 24.9, 22.8, 22.2; **ν_{max}**/ cm^{-1} (neat) 3293, 1733, 1435, 1198, 1117, 892, 767, 652; **m/z HRMS** (ESI^+) $[M + H]^+$ $C_{10}H_{18}O_2N^+$ calc. 184.1332, found 184.1332. *Data is consistent with literature*[28]

Methyl N-(methylsulfonyl)-N-(prop-2-yn-1-yl)leucinate (S15): Prepared according to a literature procedure.[29] To a solution of methyl prop-2-yn-1-ylleucinate **S14** (1.00 g, 5.46 mmol, 1.00 equiv.) and Et_3N (0.84 mL, 6.00 mmol, 1.10 equiv.) in CH_2Cl_2 (100 mL) at –20 °C

was added methanesulfonyl chloride (0.51 mL, 6.55 mmol, 1.20 equiv.) dropwise. The mixture was then warmed to room temperature and stirred overnight (18 h). Once complete by TLC analysis, the reaction was quenched by addition of H_2O (20 mL) and the layers were separated. The organic phase was washed with H_2O (20 mL), dried over Na_2SO_4, filtered and concentrated *in vacuo*. Purification of the crude residue by column chromatography (1:9 to 1:4 EtOAc:Petrol) yielded methyl *N*-(methylsulfonyl)-*N*-(prop-2-yn-1-yl)leucinate **S15** (497 mg, 1.90 mmol, 35%) as a colourless oil.

δ_H (400 MHz, $CDCl_3$) 4.61-4.55 (1H, m, C(3)*H*), 4.13 (1H, dd, *J* = 18.8, 2.4 Hz, C(2)*H*H), 4.06 (1H, dd, *J* = 18.8, 2.5 Hz, C(2)H*H*), 3.72 (3H, s, OC(7)H_3), 3.04 (3H, s, C(8)H_3), 2.30 (1H, t, *J* = 2.5 Hz, C(1)*H*), 1.86-1.64 (3H, m, C(4)H_2, C(5)*H*), 0.95 (6H, t, *J* = 6.2 Hz, C(6)H_3); **δ_C** (101 MHz, $CDCl_3$) 171.8, 78.9, 72.9, 57.8, 52.4, 40.7, 38.4, 33.6, 24.6, 23.0, 21.4; **ν_{max}**/ cm^{-1} (neat) 3275, 1737, 1331, 1149, 1056, 993, 970, 903, 871, 773, 664; **m/z HRMS** (ESI^+) $[M + Na]^+$ $C_{11}H_{19}O_4N^{23}Na^{32}S^+$ calc. 284.0927, found 284.0925.

N-(1-hydroxy-4-methylpentan-2-yl)-N-(prop-2-yn-1-yl)methanesulfonamide (2m): Prepared according to **general procedure G**, using methyl *N*-(methylsulfonyl)-*N*-(prop-2-yn-1-yl)leucinate **S15**, (0.450 g, 1.72 mmol, 1.00 equiv.), $LiBH_4$ (2 M in THF, 1.2 mL, 2.41 mmol, 1.40 equiv.) and THF (17 mL) ***with stirring at 60 °C overnight***. Purification by column chromatography (3:7 to 4:6 EtOAc:Petrol) yielded *N*-(1-hydroxy-4-methylpentan-2-yl)-*N*-(prop-2-yn-1-yl)methanesulfonamide **2m** (254 mg, 1.09 mmol, 63%) as a white powder.

m.p. (CH_2Cl_2) 62–64 °C; **δ_H** (400 MHz, $CDCl_3$) 4.18 (1H, dd, *J* = 18.8, 2.5 Hz, C(2)*H*H), 4.07-3.96 (1H, m, C(3)*H*), 3.91 (1H, dd, *J* = 18.8, 2.5 Hz, C(2)H*H*), 3.75-3.65 (1H, m, C(4)*H*H), 3.65-3.55 (1H, m, C(4)H*H*), 3.07 (3H, s, C(9)H_3), 2.38 (1H, t, *J* = 2.5 Hz, C(1)*H*), 2.30 (1H, bs, O*H*), 1.73-1.58 (1H, m, C(6)*H*), 1.44 (1H, ddd, *J* = 14.3, 9.1, 5.2 Hz, C(5)*H*H), 1.25-1.14 (1H, ddd, *J* = 14.3, 8.7, 6.5 Hz, C(5)H*H*), 0.93 (3H, d, *J* = 2.8 Hz, C(7)H_3), 0.91 (3H, d, *J* =

3.0 Hz, C(8)H_3); **δC** (101 MHz, $CDCl_3$) 80.0, 73.0, 63.4, 58.5, 41.3, 38.1, 31.6, 24.6, 23.1, 22.1, **νmax**/ cm^{-1} (neat) 3516, 3275, 1322, 1145, 880, 775; **m/z HRMS** (ESI^+) $[M + Na]^+$ $C_{10}H_{19}O_3N^{23}Na^{32}S^+$ calc. 256.0978, found 256.0978.

Methyl 5-oxo-1-(prop-2-yn-1-yl)pyrrolidine-2-carboxylate (S16) and 5-(hydroxymethyl)-1-(prop-2-yn-1-yl)pyrrolidin-2-one (2n)

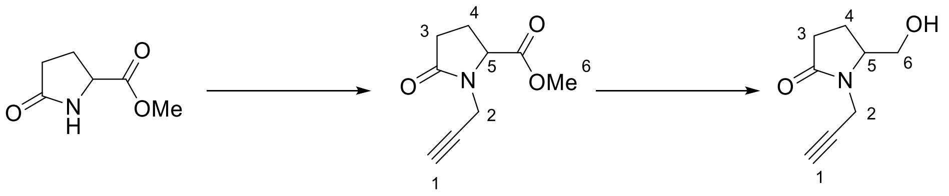

S16 **2n**

Methyl 5-oxo-1-(prop-2-yn-1-yl)pyrrolidine-2-carboxylate (S16): Prepared according to a modified procedure.[30] To a suspension of solid KOH powder (1.85 g, 33.0 mmol, 1.10 equiv.) and tetrabutylammonium bromide (0.480 g, 1.50 mmol, 0.200 equiv.) in anhydrous THF (75 mL) was added dropwise a solution of propargyl bromide (80% w/w in toluene, 5.4 mL, 48.0 mmol, 1.60 equiv.) and methyl 5-oxopyrrolidine-2-carboxylat**e** (3.50 g, 30.0 mmol, 1.00 equiv.), in anhydrous THF (75 mL) at room temperature over 1 h. The mixture was then stirred overnight (18 h) before being concentrated *in vacuo*. The residue was diluted in Et_2O (100 mL) and then washed with H_2O (100 mL), brine (100 mL), dried over Na_2SO_4, filtered and concentrated *in vacuo*. Purification of the crude residue by column chromatography (1:3 to 1:1 EtOAc:Petrol) to yield methyl 5-oxo-1-(prop-2-yn-1-yl)pyrrolidine-2-carboxylate **S16** (1.44 g, 7.95 mmol, 27%) as a colourless oil.

δH (400 MHz, $CDCl_3$) 4.61 (1H, dd, J = 17.7, 2.6 Hz, C(2)*H*H), 4.44-4.40 (1H, m, C(5)*H*), 3.78 (3H, s, C(6)H_3), 3.78 (1H, dd, J = 17.6, 2.7 Hz, C(2)H*H*), 2.56-2.33 (3H, m, C(3)H_2 and C(4)*H*H), 2.23 (1H, t, J = 2.6 Hz, C(1)*H*), 2.15-2.07 (1H, m, C(4)H*H*); **δC** (101 MHz, $CDCl_3$) 174.4, 172.0, 77.2, 73.0, 58.3, 52.5, 31.3, 29.3, 22.7; **νmax**/ cm^{-1} (neat) 2150, 1739, 1689, 1438,

1410, 1348, 1179, 1043, 992, 695; **m/z HRMS** (ESI^+) $[M + H]^+$ $C_9H_{12}O_3N^+$ calc. 182.0812, found 182.0811.

5-(Hydroxymethyl)-1-(prop-2-yn-1-yl)pyrrolidin-2-one (2n): Prepared according to **general procedure G**, using methyl 5-oxo-1-(prop-2-yn-1-yl)pyrrolidine-2-carboxylate **S16** (800 mg, 4.40 mmol, 1.00 equiv.), THF (44 mL) and $LiBH_4$ (2 M in THF, 3.10 mL, 6.30 mmol, 1.20 equiv.). Work-up using H_2O (20 mL) and EtOAc (3 × 20 mL). Purification by column chromatography (100:0 to 9:1 EtOAc:MeOH) yielded 5-(hydroxymethyl)-1-(prop-2-yn-1-yl)pyrrolidin-2-one **2n** (287 mg, 1.89 mmol, 43%) as a white powder.

m.p. (CH_2Cl_2) 43–45 °C; **δ_H** (400 MHz, $CDCl_3$) 4.34 (1H, dd, *J* = 17.6, 2.6 Hz, C(2)*H*H), 3.91 (1H, dd, *J* = 17.6, 2.6 Hz, C(2)H*H*), 3.93-3.86 (1H, m, C(6)*H*H), 3.85-3.78 (1H, m, C(5)*H*), 3.58 (1H, ddd, *J* = 12.1, 7.4, 3.2 Hz, C(6)H*H*), 3.30 (1H, dd, *J* = 7.4, 6.8 Hz, O*H*), 2.41 (1H, dddd, *J* = 17.1, 10.1, 7.0 Hz, C(3)*H*H), 2.26 (1H, ddd, 17.1, 10.1, 5.6 Hz, C(3)H*H*), 2.20 (1H, t, *J* = 2.5 Hz, C(1)*H*), 2.12-2.00 (1H, m, C(4)*H*H), 1.99-1.88 (1H, m, C(4)H*H*); **δ_C** (101 MHz, $CDCl_3$) 175.7, 78.3, 72.1, 62.5, 59.2, 30.6, 30.4, 20.9; **ν_{max}**/ cm^{-1} (neat) 3387, 3282, 1660, 1420, 1348, 1254, 1185, 1058, 652; **m/z HRMS** (ESI^+) $[M + H]^+$ $C_8H_{12}O_2N^+$ calc. 154.0863, found 154.0863.

***tert*-Butyl (1-hydroxy-3-phenylpropan-2-yl)carbamate (S17), *tert*-butyl (1-phenyl-3-(prop-2-yn-1-yloxy)propan-2-yl)carbamate (2o) and *tert*-Butyl (1-phenyl-3-(prop-2-yn-1-yloxy)propan-2-yl)carbamate (S18)**

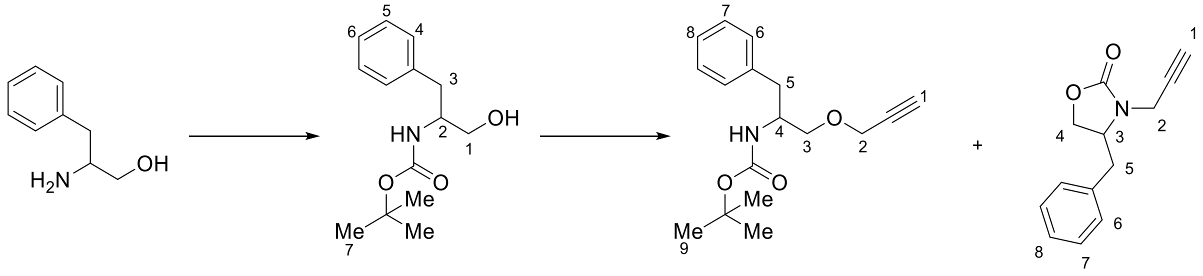

S17 **2o** **S18**

tert-Butyl (1-hydroxy-3-phenylpropan-2-yl)carbamate (S17): Prepared according to a modified procedure.[31] In a round-bottomed flask under N_2, phenylalaninol (1.00 g, 6.60 mmol, 1.00 equiv.) was dissolved in CH_2Cl_2 (20 mL) and cooled to 0 °C. Then, Boc_2O (1.7 mL, 7.59 mmol, 1.15 mmol), was added and the mixture was stirred at 0 °C for 18 h. Once the reaction was complete by TLC analysis, the mixture was washed with 5% aqueous citric acid solution (50 mL) and the layers were separated. The aqueous layer was extracted with CH_2Cl_2 (3 × 10 mL). The combined organic extracts were then washed with brine (25 mL), dried over Na_2SO_4, filtered and concentrated *in vacuo*. The crude residue was then purified by column chromatography (19:1 to 1:1 Petrol:EtOAc) to yield *tert*-butyl (1-hydroxy-3-phenylpropan-2-yl)carbamate **S17** (1.42 g, 5.65 mmol, 86%) as a white powder.

m.p. (CH_2Cl_2) 88–90 °C; (*Lit:* 89–91 °C)[32]; **δ_H** (400 MHz, $CDCl_3$) 7.36-7.16 (5H, m, *Ar*), 4.72 (1H, bs, N*H*), 3.87 (1H, bs, C(2)*H*), 3.71-3.64 (1H, m, C(1)*H*H), 3.59-3.52 (1H, m, C(1)H*H*), 2.84 (2H, d, J = 7.2 Hz, C(3)H_2), 1.41 (9H, s, C(7)H_3); **δ_C** (100 MHz, $CDCl_3$) 156.3, 137.9, 129.4, 128.7, 126.7, 79.9, 64.5, 53.9, 37.6, 28.5; **ν_{max}**/ cm^{-1} (neat) 3355, 2980, 2875, 1686, 1526, 1315, 1268, 1251, 1169, 1005, 700; **m/z LRMS** 274.1 $[M + Na]^+$. *Data is consistent with literature[31]*

tert-Butyl (1-phenyl-3-(prop-2-yn-1-yloxy)propan-2-yl)carbamate (2o): Prepared according to **general procedure D**, using *tert*-butyl (1-hydroxy-3-phenylpropan-2-yl)carbamate **S17** (1.00 g, 3.99 mmol, 1.00 equiv.), NaH (60% in mineral oil, 167 mg, 4.18 mmol, 1.05 equiv.), propargyl bromide (80% in toluene, 1.2 mL, 10.36 mmol, 2.60 equiv.) and DMF (17.5 mL). Quench using sat. aq. NH_4Cl (20 mL). Work-up using EtOAc (3 × 20 mL), 5% aq. LiCl (20 mL). Purification by column chromatography (1:99-3:7 EtOAc:Petrol) yielded *tert*-butyl (1-phenyl-3-(prop-2-yn-1-yloxy)propan-2-yl)carbamate **2o** (423 mg, 1.46 mmol, 37%) and cyclised side adduct 4-benzyl-3-(prop-2-yn-1-yl)oxazolidin-2-one **S18** (307 mg, 1.42 mmol, 36%) as a white powder and pale-yellow oil respectively.

tert-Butyl (1-phenyl-3-(prop-2-yn-1-yloxy)propan-2-yl)carbamate (2o): **m.p.** (CH_2Cl_2) 40–42 °C; **δ_H** (400 MHz, $CDCl_3$) 7.31-7.17 (5H, m, *Ar*), 4.85 (1H, bs, N*H*), 4.14 (2H, dd, *J* = 2.4, 1.1 Hz, C(3)H_2), 3.95 (1H, m, C(4)*H*), 3.43 (2H, dd, *J* = 3.9, 1.6 Hz, C(5)H_2), 2.85 (2H, app hept, *J* = 7.8 Hz, C(2)H_2), 2.42 (1H, t, *J* = 2.4 Hz, C(1)*H*), 1.41 (9H, s, C(9)H_3); **δ_C** (101 MHz, $CDCl_3$) 155.3, 138.1, 129.5, 128.4, 126.4, 79.5, 79.2, 74.8, 69.9, 58.4, 51.5, 37.8, 28.4; **ν_{max}**/ cm^{-1} (neat) 3294, 2976, 1700, 1498, 1453, 1391, 1364, 1246, 1168, 1059, 1029, 742, 701; **m/z HRMS** (ESI^+) $[M + Na]^+$ $C_{17}H_{23}O_3N^{23}Na^+$ calc. 312.1570, found 312.1570.

4-benzyl-3-(prop-2-yn-1-yl)oxazolidin-2-one (S18): **δ_H** (400 MHz, $CDCl_3$) 7.37-7.31 (2H, m, *Ar*), 7.31-7.27 (1H, m, *Ar*), 7.22-7.15 (2H, m, *Ar*), 4.43 (1H, dd, *J* = 17.9, 2.6 Hz, C(2)*H*H), 4.29-4.16 (2H, m, C(3)*H* and C(4)*H*H), 4.09-3.98 (1H, m, C(4)H*H*), 3.83 (1H, dd, *J* = 17.8, 2.5 Hz, C(2)H*H*), 3.24 (1H, dd, *J* = 13.6, 4.2 Hz, C(5)*H*H), 2.70 (1H, dd, *J* = 13.9, 8.6 Hz, C(5)H*H*), 2.34 (1H, app t, *J* = 2.5 Hz, C(1)*H*); **δ_C** (101 MHz, $CDCl_3$) 157.8, 135.4, 129.1, 129.1, 127.4, 77.0, 73.5, 67.3, 55.8, 38.4, 32.6; **ν_{max}**/ cm^{-1} (neat) 3283, 2980, 1750, 1250, 1091; **m/z LRMS** (ESI^+) 238.0 $[M + Na]^+$. *Data is consistent with literature[33]*

Dec-9-yn-5-ol (2p)

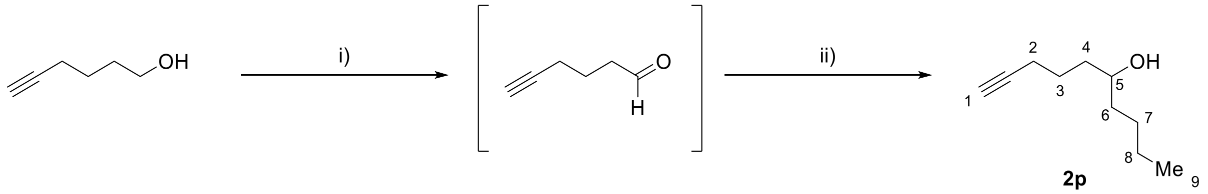

Prepared according to literature procedure.[34] A anhydrous flask under argon was charged with CH_2Cl_2 (42 mL), Et_3N (4.2 mL, 30.2 mmol, 3.28 equiv.), dimethyl sulfoxide (2.1 mL, 29.6 mmol, 3.21 equiv.) and hex-5-yn-1-ol (1.0 mL, 9.22 mmol, 1.00 equiv.). The solution was cooled to 0 °C and stirred for 5 min before pyridine-sulfur trioxide complex (4.42 g, 27.8 mmol, 3.02 equiv.) was added. The mixture was then warmed to room temperature and stirred vigorously for 2 h before being quenched by sat. aq. NH_4Cl (20 mL) at 0 °C. The layers were separated and the aqueous layer was extracted with CH_2Cl_2 (3 × 20 ml). The combined organic extracts were washed with H_2O (20 mL) and brine (20 mL), dried over $MgSO_4$, filtered and concentrated *in vacuo* to yield the crude aldehyde. *n*-Butyllithium (2.15 M in THF, 8.6 mL, 18.44 mmol 2.00 equiv.) was then added to a flask containing anhydrous THF (188 mL) and cooled to –78 °C. The crude aldehyde was then added dropwise to this solution and the resulting mixture was allowed to warm to room temperature over 2 h. After this time, the reaction was quenched with MeOH (5 mL) at 0 °C and concentrated *in vacuo*. The residue was then dissolved in 10% w/w aq. HCl (20 mL) and extracted with CH_2Cl_2 (3 × 20 mL). The combined organic extracts were dried over Na_2SO_4, filtered and concentrated *in vacuo*. Purification of the crude residue by column chromatography (0:100 Petrol to 1:19 Et_2O:Petrol) yielded to dec-9-yn-5-ol **2p** (118 mg, 0.766 mmol, 8% over two steps) as a colourless oil.

δ_H (400 MHz, $CDCl_3$) 3.66-3.57 (1H, m, C(5)*H*), 2.22 (2H, td, *J* = 6.7, 2.6 Hz C(2)H_2), 1.95 (1H, t, *J* = 2.7 Hz, C(1)*H*), 1.75-1.25 (10H, m, C(3, 4, 6, 7 and 8)H_2), 0.90 (3H, t, *J* = 7.0 Hz, C(9)H_3); **δ_C** (101 MHz, CDCl3) 84.5, 71.6, 68.6, 37.4, 36.5, 27.9, 24.7, 22.7, 18.6, 14.2; **ν_{max}**/

cm^{-1} (neat) 3309, 2930, 2861, 1457, 1434, 1127, 1057, 1004, 630. *Data is consistent with literature*[34]

1-Phenylpent-4-yn-1-al (S19) and 1-phenylpent-4-yn-1-ol (2q)

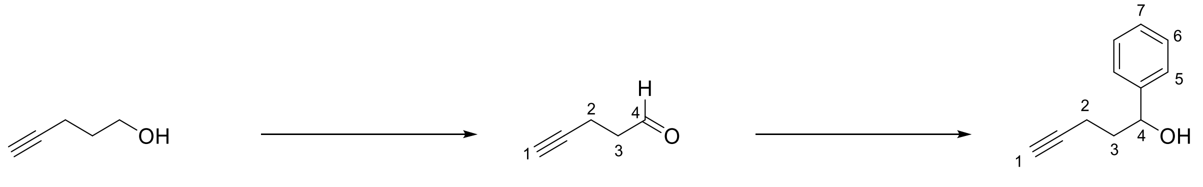

1-Phenylpent-4-yn-1-al (S19): Prepared according to a literature procedure.[35] Oxalyl chloride (2.8 mL, 33.0 mmol), was dissolved in anhydrous CH_2Cl_2 (63 mL) and cooled to –78 °C under N_2. A solution containing dimethyl sulfoxide (4.7 mL, 66.0 mmol, 2 equiv.) in CH_2Cl_2 (5 mL) was added dropwise over 30 min with stirring at –78 °C. A solution of pent-4-yn-1-ol (2.8 mL, 30.0 mmol) in CH_2Cl_2 (5 mL) was then added dropwise and the mixture was stirred for a further 60 min at –78 °C. Et_3N (21.3 mL, 153 mmol) in CH_2Cl_2 (20 mL) was then added dropwise and the solution was allowed to warm to room temperature overnight. After this time, H_2O (100 mL) was then added and the layers were separated. The aqueous layer was acidified with 1 M aq. HCl (75 mL) and extracted with CH_2Cl_2 (3 × 75 mL). The combined organic extracts were washed with 1 M HCl (75 mL), sat. aq. $NaHCO_3$ (75 mL), dried over $MgSO_4$, filtered and concentrated *in vacuo*. Purification of the crude residue by distillation (Lit bp: 127–129 °C at 1 atm)[35] yielded 1-phenylpent-4-yn-1-al **S19** (1.54 g, 18.8 mmol, 63%).

δ_H (400 MHz, $CDCl_3$) 9.80 (1H, t, J = 1.1 Hz C(4)*H*), 2.74-2.66 (2H, m, C(3)H_2), 2.54-2.48 (2H, m, C(2)H_2), 1.99 (1H, t, J = 2.7 Hz, C(1)*H*); **δ_C** (101 MHz, $CDCl_3$) 200.2, 82.4, 69.4, 42.4, 11.7; **ν_{max}**/ cm^{-1} (neat) 3292, 2980, 1723, 1134, 1066, 940, 629. *Data is consistent with literature.*[35]

1-Phenylpent-4-yn-1-ol (2q): Prepared according to a modified procedure.[35] To dry flask under argon containing magnesium turnings (280 mg, 11.6 mmol, 1.90 equiv.) was added dropwise anhydrous Et_2O (8 mL) and the mixture stirred vigorously for 30 min. A solution of bromobenzene (0.92 mL, 8.71 mmol, 1.43 equiv.) in Et_2O (8 mL) was added dropwise. The mixture was then stirred at room temperature for 1 h and heated to reflux for 30 min. The newly formed Grignard reagent was added dropwise to a solution of pent-4-yn-1-ol **S19** (680 mg, 6.09 mmol, 1.00 equiv.) in Et_2O (8 mL) and the mixture was heated to reflux for 2 h. After cooling to room temperature, ice (3 g) was added, and the resulting suspension was dissolved in 1 M aq. HCl (20 mL). The layers were then separated and the organic layer was washed with sat. aq. $NaHSO_3$ (20 mL), sat. aq. $NaHCO_3$ (20 mL), H_2O (20 mL), dried over $MgSO_4$, filtered and concentrated *in vacuo*. Purification of the crude residue by column chromatography (1:9 to 1:4 Et_2O:Petrol) yielded 1-phenylpent-4-yn-1-ol **2q** (488 mg, 3.05 mmol, 50%) as a white solid.

m.p. (CH_2Cl_2) 30–31 °C; $\boldsymbol{\delta_H}$ (400 MHz, $CDCl_3$) 7.39-7.25 (5H, m, *Ar*), 4.86 (1H, ddd, *J* = 8.3, 5.0, 3.3 Hz, C(4)*H*), 2.42-2.31 (1H, bs, O*H*), 2.36-2.27 (1H, m, C(2)*H*H), 2.25-2.15 (1H, m, C(2)H*H*), 2.01 (1H, t, *J* = 2.7 Hz, C(1)*H*), 2.00-1.93 (1H, m, C(3)*H*H), 1.92-1.82 (1H, m, C(3)H*H*); $\boldsymbol{\delta_C}$ (101 MHz, $CDCl_3$) 144.1, 128.5, 127.7, 125.9, 84.0, 73.0, 69.0, 37.4, 15.1; $\boldsymbol{\nu_{max}}$/ cm^{-1} (neat) 3295, 3030, 2980, 1493, 1452, 1431, 1395, 1061, 1025, 942, 915, 763, 700, 630. *Data is consistent with literature*[35]

4. Reaction Optimisation

Table S1: Initial Optimisation of hydroacylation with alkynols and alkynamines[a]

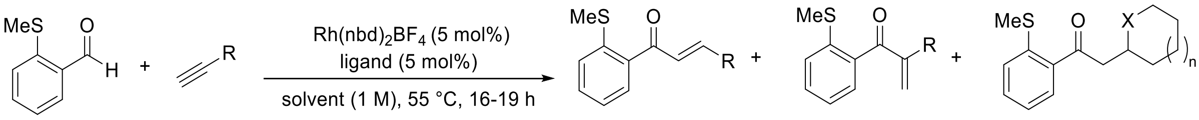

1a **2a** or **2r** **3l** **3b** **4**

X = O, N n = 0, 1

2a R = $CH_2CH_2CH_2OH$

2r R = $CH_2CH_2CH_2CH_2NH(Boc)$

Entry	Alkyne	Solvent	Ligand	Conversion (%)[b]	Ratio (3l:3b:4)
1	**2a**	Acetone	dppe	50	10:2:5
2	**2a**	DCE	dppe	95	10:1:4
3	**2a**	DCE	dcpe	100	10:1:9
4	**2r**	DCE	dppe	85	10:1:2
5	**2r**	DCE	dcpe	100	13:1:2

[a]Reaction conditions: aldehyde (1.0 equiv.), alkyne (1.5 equiv.); nbd, norbornadiene; dppe, 1,2-bis(diphenylphosphino)ethane, dcpe, 1,2-bis(dicyclohexylphosphino)ethane; DCE,1,2-dichloroethane; [b] Determined by ^{1}HNMR spectroscopic analysis of the crude reaction mixture.

Table S2: Condition Optimisation for the Hydroacylation Step with a substituted alkynol

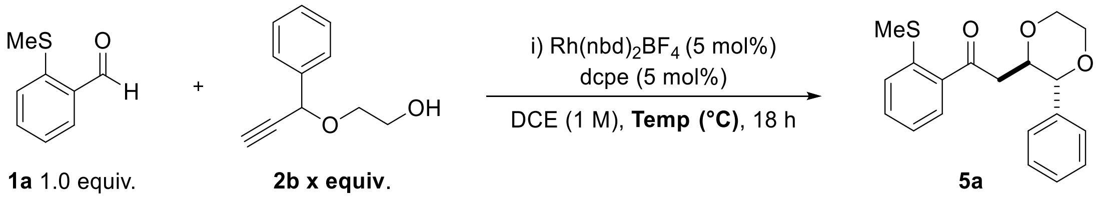

1a 1.0 equiv. **2b x equiv.** **5a**

Entry	Alkyne (equiv.)	Temp (°C)	Total yield (%)[a]	Crude dr[b]
1	1.1	55	75	4.3:1
2	1.2	55	86	4:1
3	1.2	rt	64	3:1
4	1.2	35	88	3.3:1

[a] ^{1}H NMR yields based on ^{1}H NMR standard (methyl-3,5-dinitrobenzoate); [b] dr measured from crude ^{1}H NMR spectroscopy.

Table S3: Acid Screening for the Cyclisation/Epimerisation Step

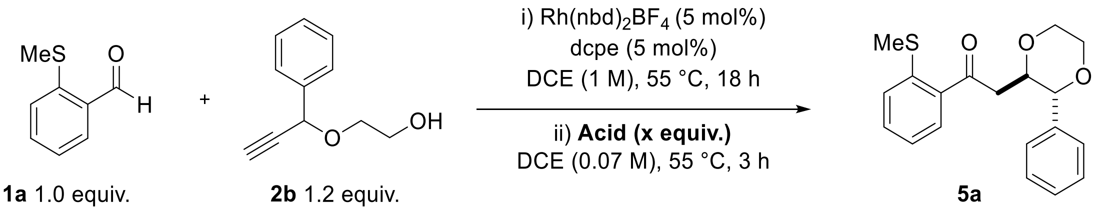

1a 1.0 equiv. **2b** 1.2 equiv. **5a**

Entry	Acid	Loading (equiv.)	Total yield (%)[a]	Crude dr[b]
1	*p*-TsOH	2	62	11:1
2	MsOH	2	33	>20:1
3	*p*-TsOH	0.2	87	6.8:1
4	MsOH	0.2	93	4.4:1
5	$Sc(OTf)_3$	0.2	78	4.5:1
6	$Ti(^iPrO)_4$	0.2	96	4.2:1
7	BF_3OEt_2	0.2	76	4.4:1
8	$AlCl_3$	0.2	87	4.4:1
9	BCl_3	0.2	83	7.3:1
10[c]	SiO_2	-	83	4.3:1
11[d]	Amberlyst 15	-	78	5.3:1

[a] ^{1}H NMR yields based on ^{1}H NMR standard (methyl-3,5-dinitrobenzoate); [b] dr measured from crude ^{1}H NMR spectroscopy; [c] 0.5 g of SiO_2, [d] 200 mg of Amberlyst 15.

Table S4: Room Temperature Reactions and DCE/THF Solvent-Switches for Sulfonic Acid Addition

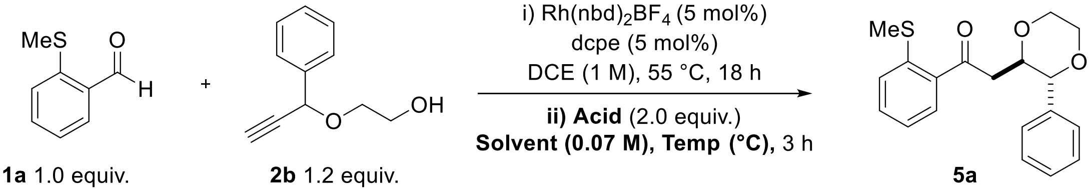

1a 1.0 equiv. **2b** 1.2 equiv. **5a**

Entry	Acid	Solvent	Temp (°C)	Total yield (%)[a]	Crude dr[b]
1	*p*-TsOH	DCE	rt	78	4.5:1
2	MsOH	DCE	rt	69	5.2:1
3	2,4-DNSA	DCE	rt	93	3.8:1
4	*p*-TsOH	THF	rt	94	5.7:1
5	*p*-TsOH	THF	35	82	5.7:1
6	2,4-DNSA	THF	rt	94(80)[c]	5.2:1
7	2,4-DNSA	THF	35	81	4.8:1

[a] ^{1}H NMR yields based on ^{1}H NMR standard (methyl-3,5-dinitrobenzoate); [b] dr measured from crude ^{1}H NMR spectroscopy, [c] Isolated yield of major diastereoisomer (>20:1 dr) in parentheses.

Table S5: Condition Screening using 4 M HCl.dioxane for the Cyclisation/Epimerisation Step

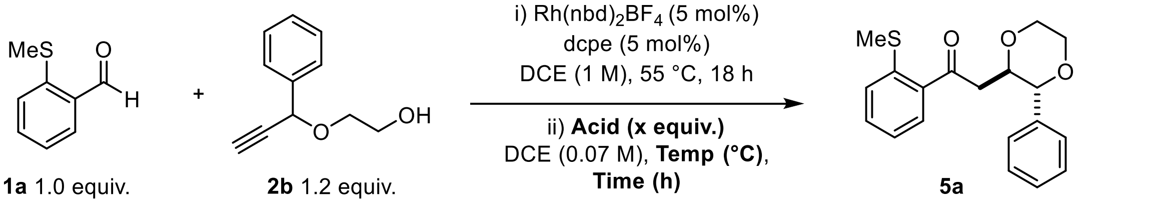

Entry	HCl in dioxane (equiv.)	Time (h)	Temp (°C)	Total yield (%)[a]	Crude dr[b]
1	0.2	3	55	87	5.2:1
2	0.2	3	rt	88	4.9:1
3	2.0	3	55	95(88)[c]	13:1
4	2.0	3	rt	94	5:1
5	2.0	5	55	93	13:1
6	2.0	7	"	95	12:1
7	2.0	24	"	91	11:1
8	3.0	3	55	94(82)[c]	15:1
9	4.0	3	"	95(84)[c]	14:1
10	5.0	3	"	85	12:1
11	10.0	3	"	80	11:1

[a] ^{1}H NMR yields based on ^{1}H NMR standard (methyl-3,5-dinitrobenzoate); [b] dr measured from crude ^{1}H NMR spectroscopy; [c] Isolated yield of major diastereoisomer (>20:1 dr) shown in parentheses.

Table S6: Acid evaluation for the cyclisation to compound **5z:**

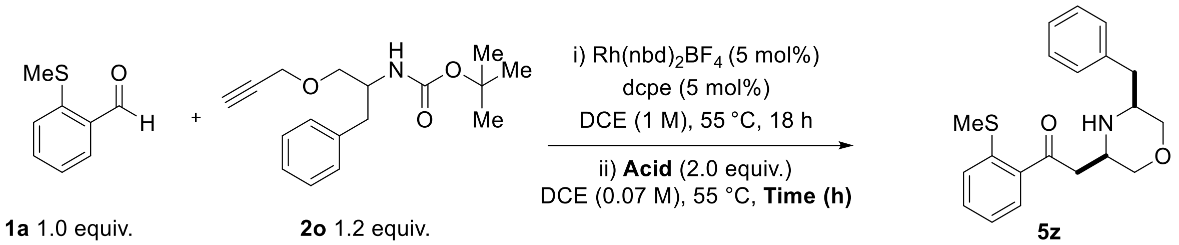

Entry	Additive	Time (h)	Total yield (%)[a]	dr[b]
1	HCl in dioxane	3	17	n/a[c]
2	TFA	36	42	n/a[c]
3	*p*-TSA	3	50	2.4:1

[a] Isolated yields; [b] dr was measured from ^{1}H NMR spectroscopy of the crude reaction mixture; [c] Crude ^{1}H NMR was too complex to give a dr value.

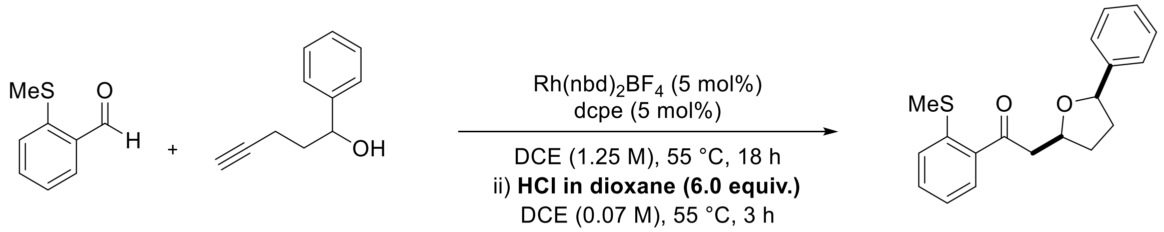

1a (1.0 equiv.) **2q** (1.2 equiv.) **5ab** 83%, 1:1 dr

Scheme S1: Higher acid loading in the cyclisation/epimerisation step towards the formation of diastereoisomeric mixture **5ab**

Table S7: Other attempts to improve the diastereomeric ratio in diastereoisomeric mixture **5ab:**

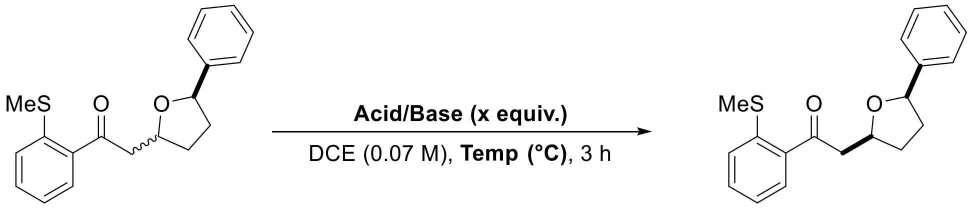

5ab 1:1 dr **5ab** 1:1 dr

Entry	Acid/Base	Loading (equiv.)	Temp (°C)	Crude dr[a]
1	HCl.dioxane	6.0	100	1:1
2	^{t}BuOK	2.0	55	1:1
3	NaH	2.0	55	1:1
4	DBU	2.0	55	1:1
5	TEA	2.0	55	1:1

[a] dr measured from crude ^{1}H NMR spectroscopy

5. Assignment of Relative Configurations of cyclised products

5.1. *O*- and *N*-heterocycles

5.1.1 R^3 substituents

Cyclised products containing R^3 substituents were analysed through $^3J_{HH}$ coupling constants, an example being diastereomeric mixture **5g** detailed below:

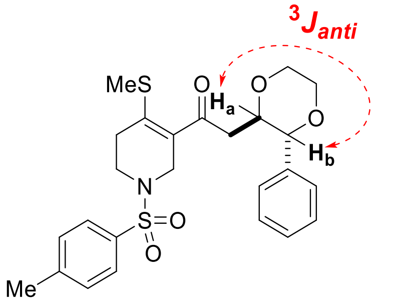

5g

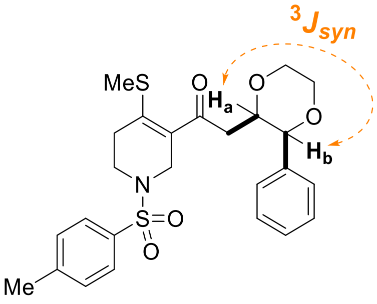

5g'

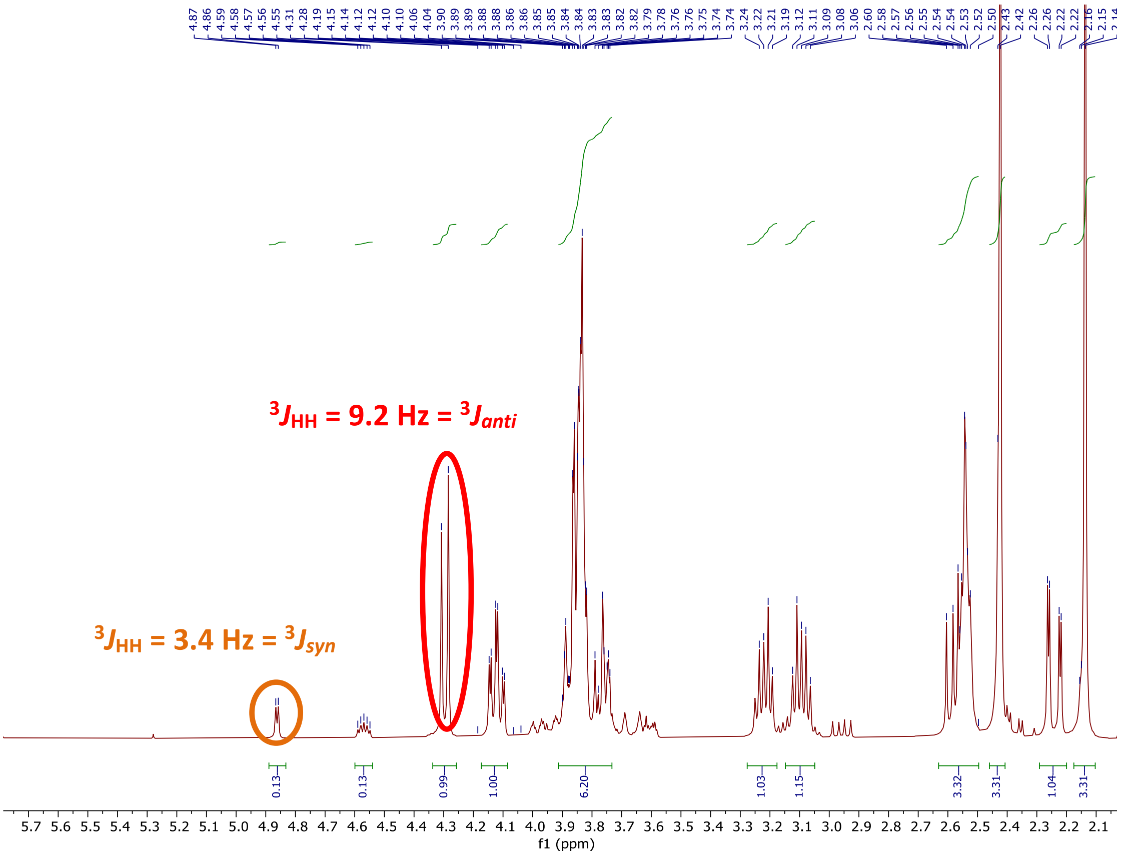

Analogous to diastereoisomeric mixture **5g**, the major diastereomers in all R^3-substituted products display a vicinal $^3J_{HH}$ constant 9.0-10 Hz between protons H_a and H_b. We confirm this to be an *anti*-coupling constant, where the smaller coupling constant in the minor diastereoisomer **5g'** suggests that a *syn*-configuration is present. The relative stereochemistry at this position could also be determined unambiguously by X-ray crystal structure of **5k**, which had a correlating vicinal $^3J_{HH}$ constant of 9.3 Hz.

Products at this position could also be analysed by ^{1}H NOESY data, as exemplified by diastereomeric mixture **5i** below:

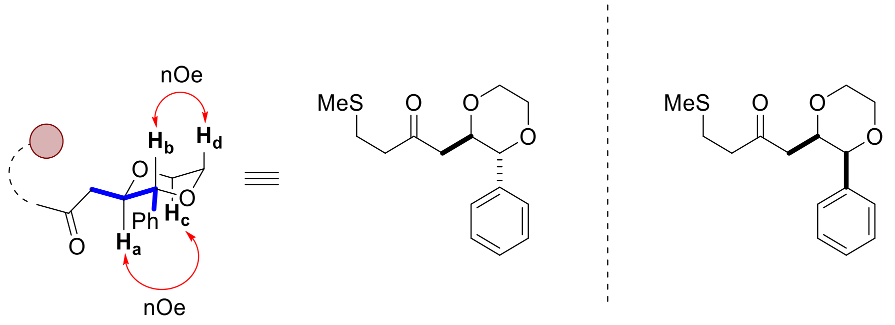

5i **5i'**

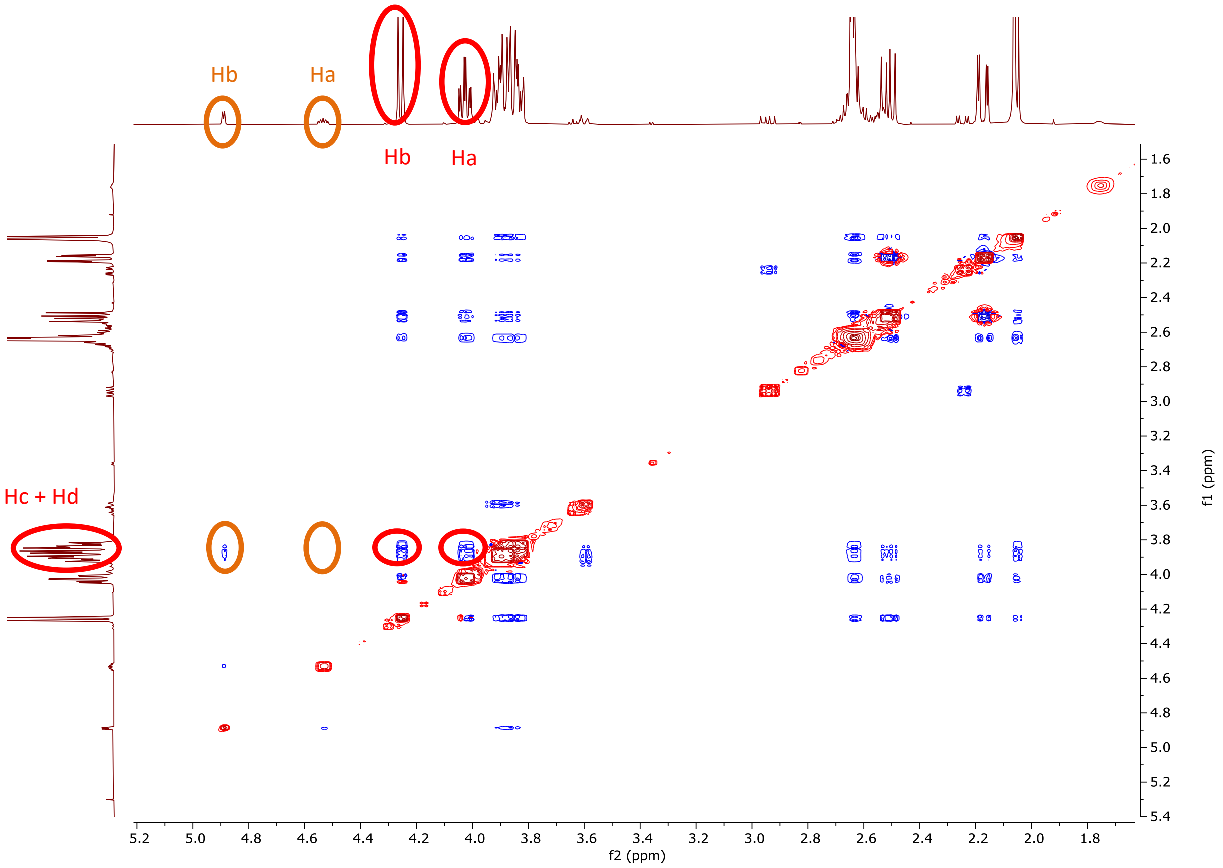

Major diastereoisomer **5i** shows two main NOESY interactions between Ha and Hc and between Hb and Hd environment, suggesting that an *anti*-configuration is present. The minor diastereoisomer **5i'** only shows one key NOESY interaction between Hb and both Hc and Hd, suggesting that the *syn*-configuration is present.

5.1.2 R^2 substituents

The relative stereochemistry at this position could also be determined unambiguously through X-ray crystallography, showing the major diastereoisomer of phenyl substituted product **5q** to be *anti*. Alkyl R^2 substituted products were also identified to be *anti* through ^{1}H NOESY analysis, using an example spectrum of diastereomeric mixture **5o**, detailed below:

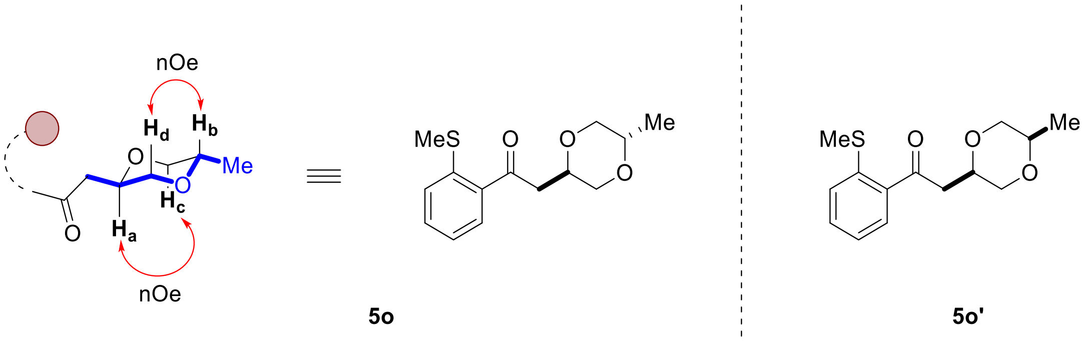

5o **5o'**

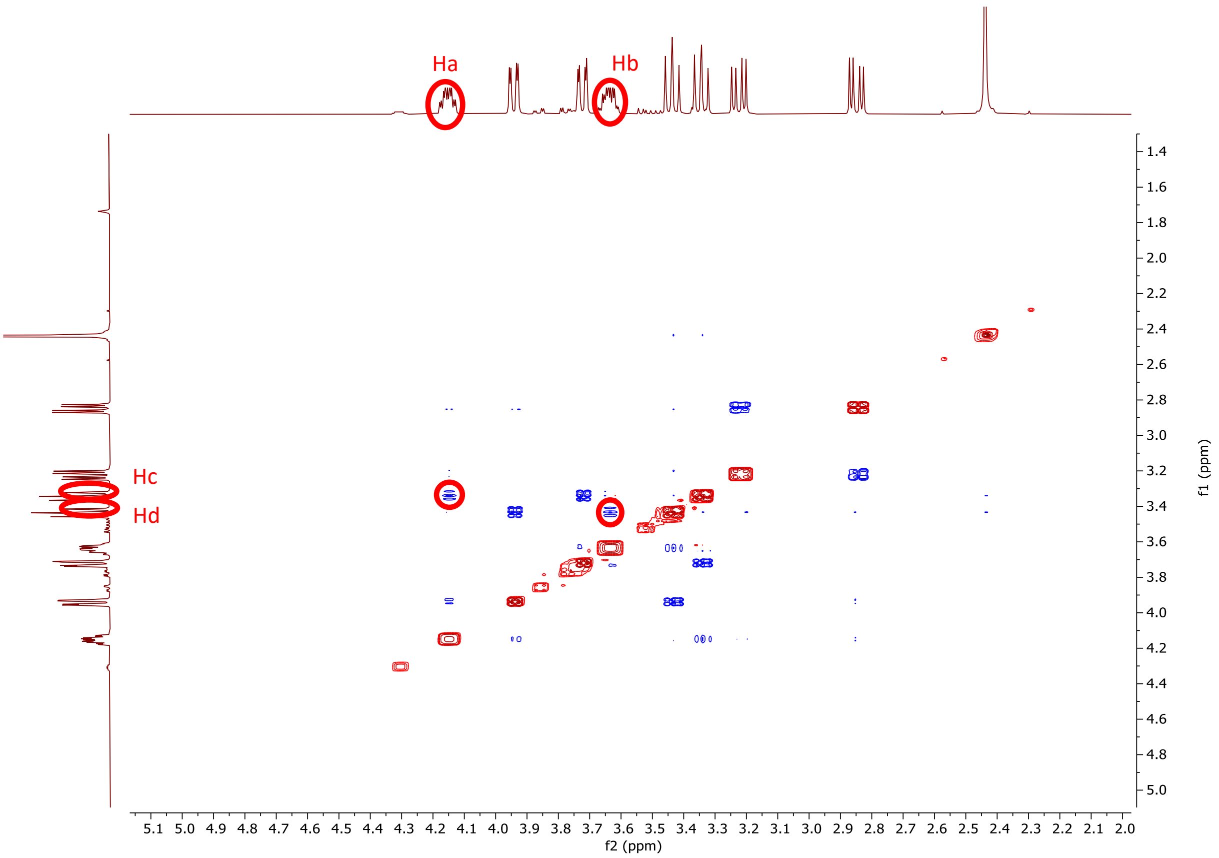

The Major diastereoisomer **5o** shows key NOESY interactions with methine environments Ha and Hb towards axial methylene proton environments Hc and Hd respectively, thus indicating an *anti*-configuration.

Additionally, *anti* vicinal $^3J_{HH}$ couplings observed between Hb and Hc and Ha and Hd help to reveal the equatorial-equatoral relationship between the ring substituents in major diastereoisomer **5o**. Evidence for these *anti* vicinal $^3J_{HH}$ couplings (~10 Hz) is provided in spectra for diastereomeric mixture **5o**, below:

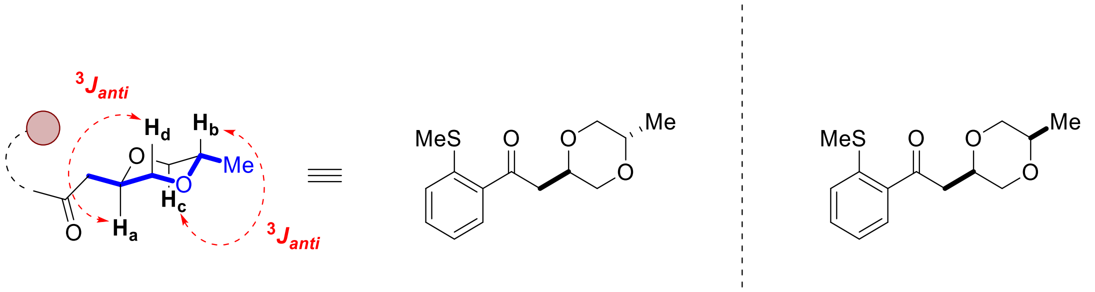

5o **5o'**

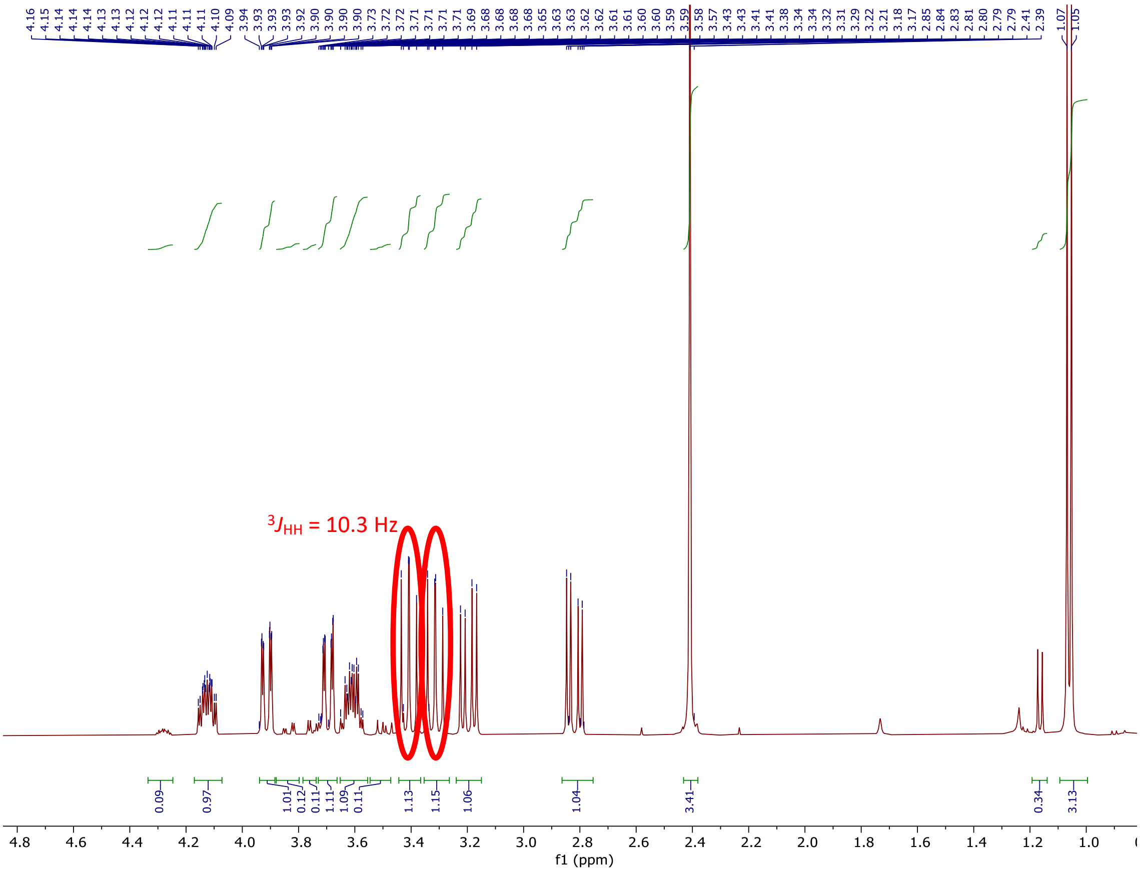

5.1.3 R[1] substituents

The relative configuration of cyclised products with this substitution pattern could be easily identified with ^{1}H NOESY analysis. A primary example, using the spectra from diastereomeric mixture **5ag** is detailed below:

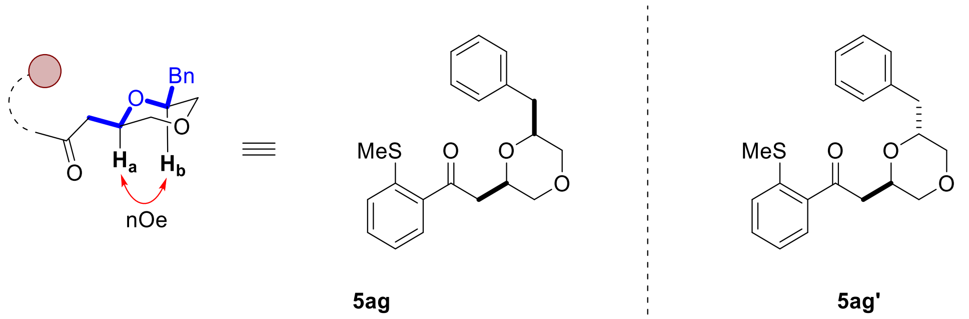

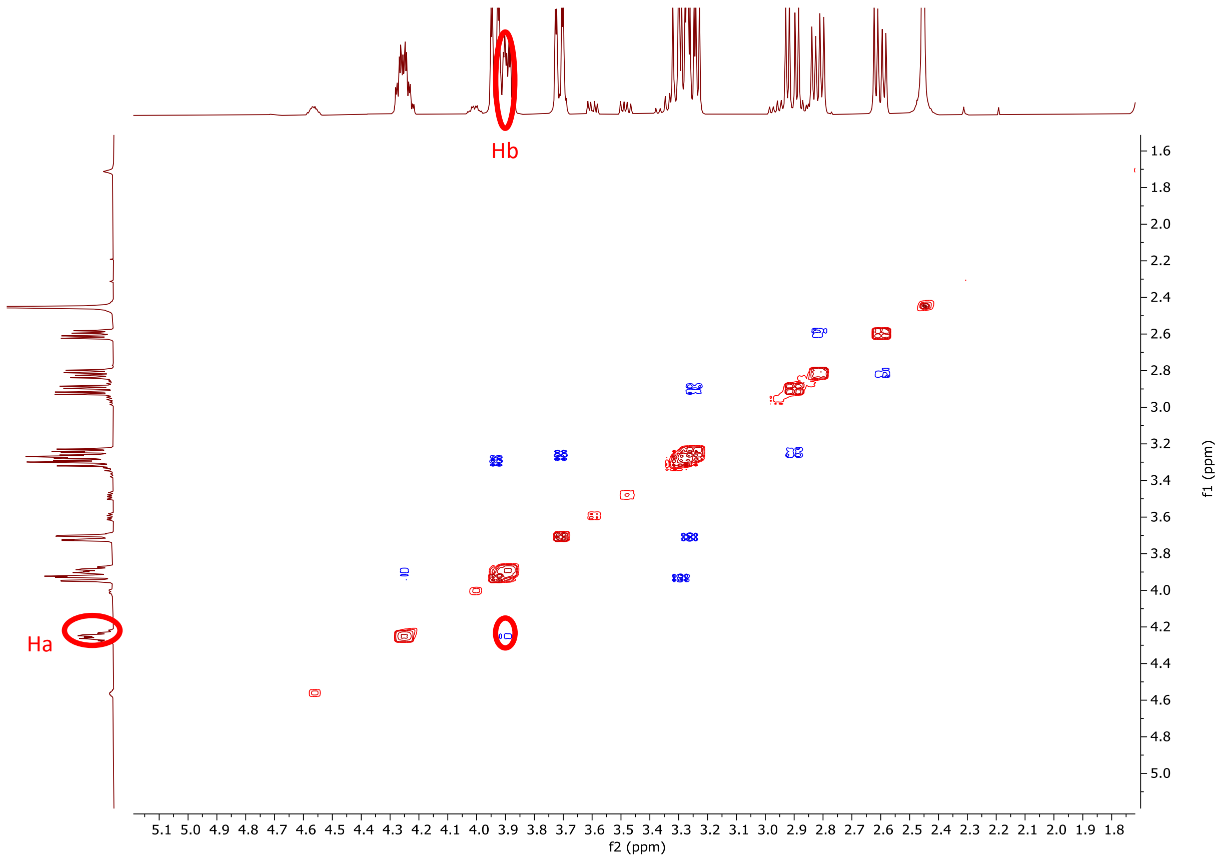

A key NOESY interaction between methine protons Ha and Hb, seen for major diastereoisomer **5ag**, might suggest an axial-axial relationship, thus leading to the *syn*-product. When analysing the same methine proton environments (Ha and Hb) in minor diastereoisomer **5ag’** (see below), a NOESY interaction was absent.

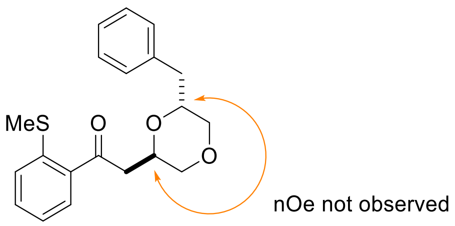

5ag'

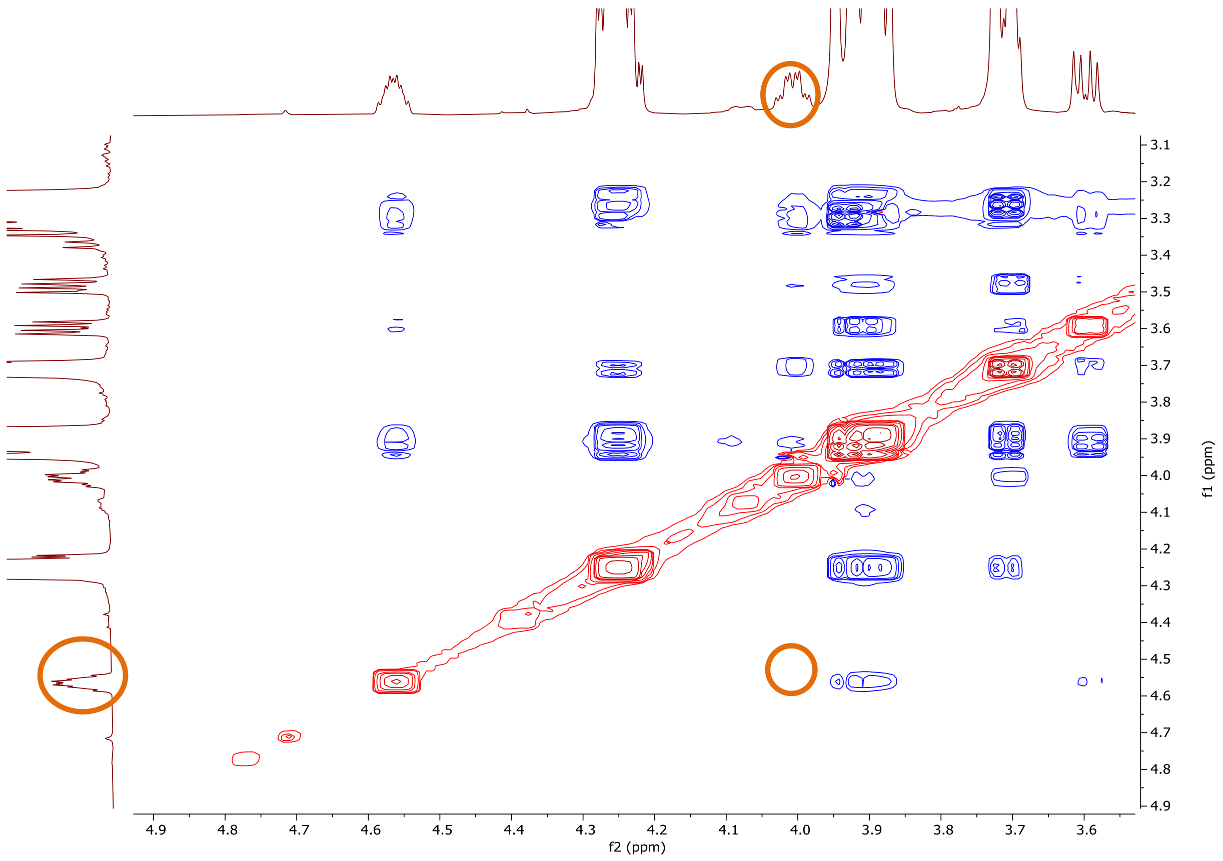

When considering *N*-heterocyclic product **5z**, a *syn* relative configuration in major diastereoisomer **5z** could easily be identified by two key NOESY correlations:

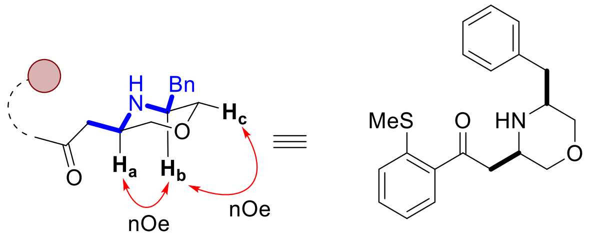

5z

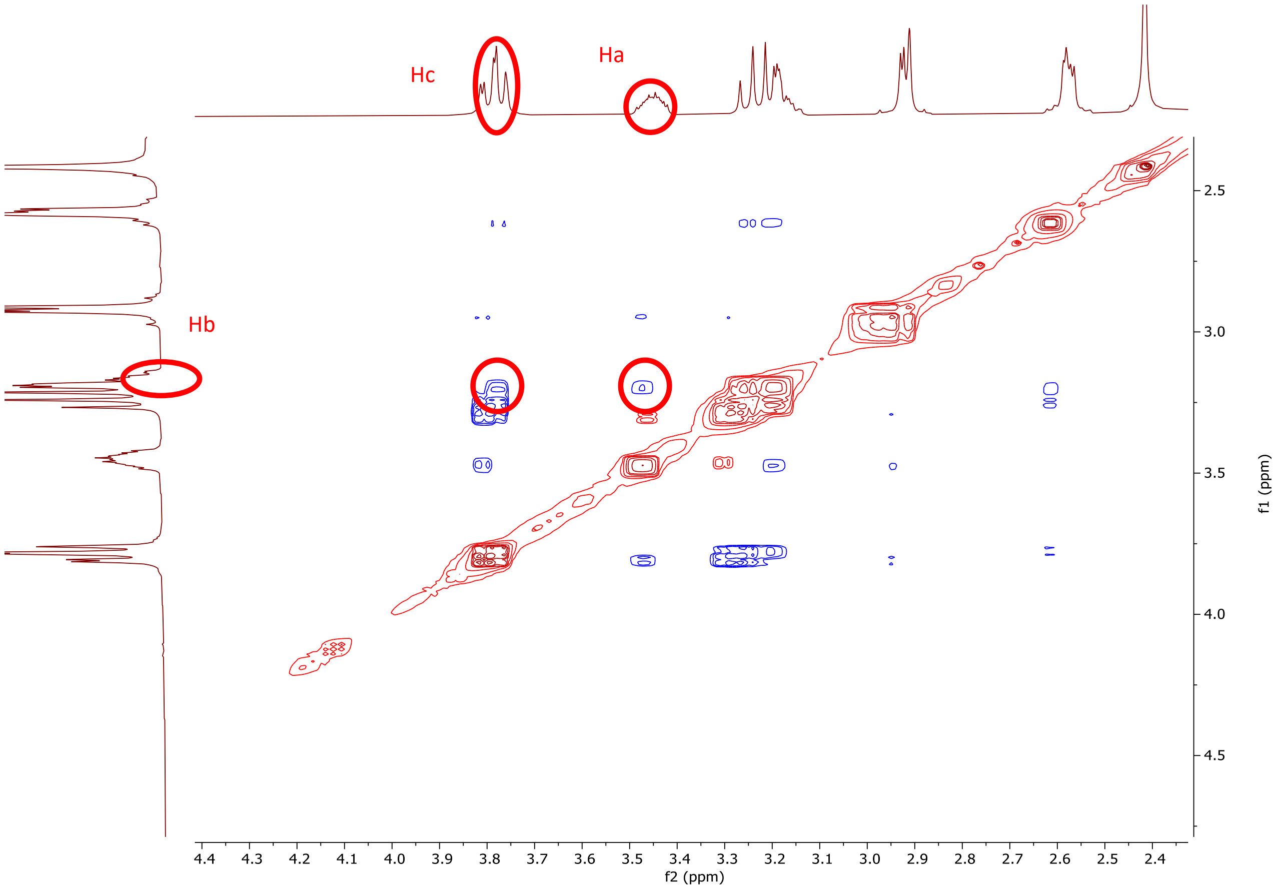

The *anti* relative configuration in minor diastereoisomer **5z’** could also be easily identified by a single NOESY correlation:

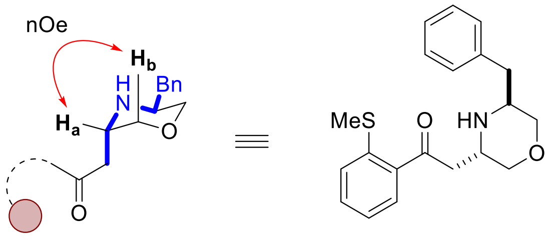

5z'

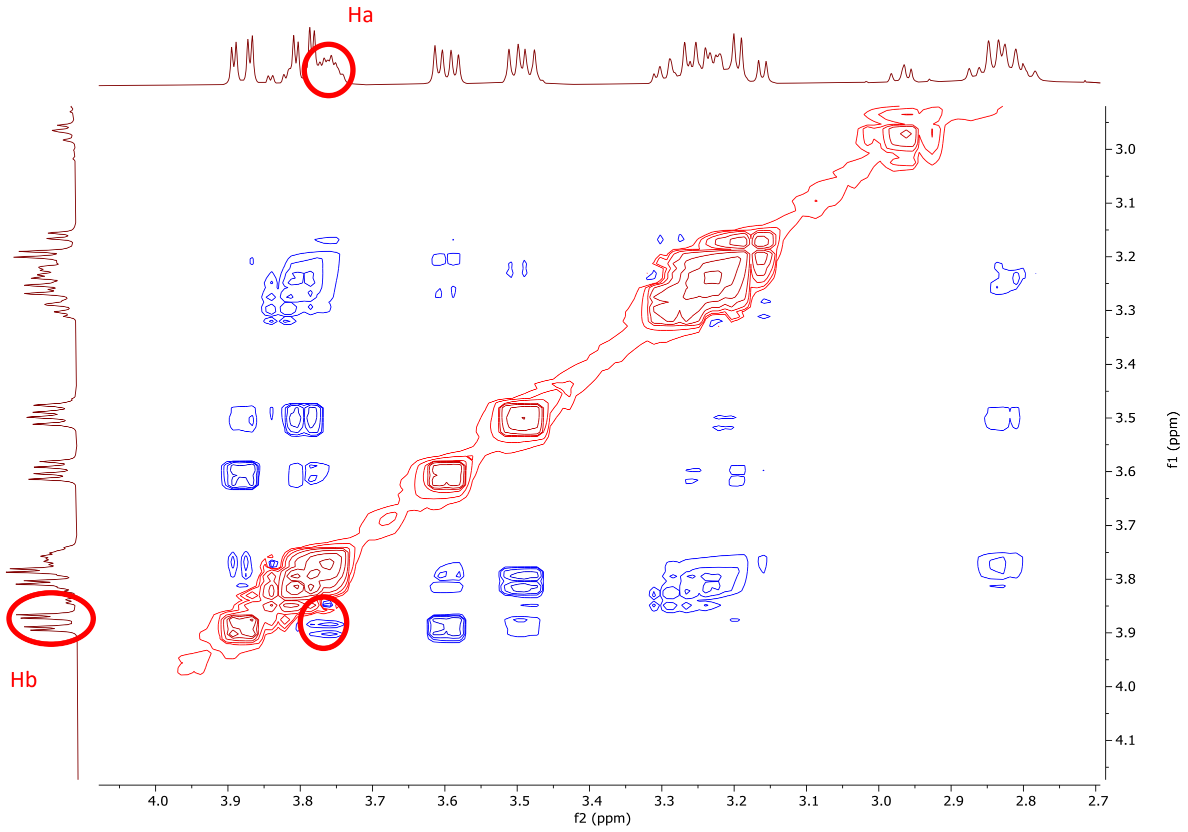

5.2. *S*-heterocycles

5.2.1 R^3 substituents

For the R^3 substituted products formed by *S*-conjugate addition, an analysis of the vicinal $^3J_{HH}$ couplings in product **7q**, was used to confirm the relative configuration. The value of the coupling between protons H_a and H_b has been measured as 9.6 Hz, a similar value to the corresponding $^3J_{anti}$ coupling constant in dioxane product **5g**, suggesting that an *anti* relative configuration is adopted at this position.

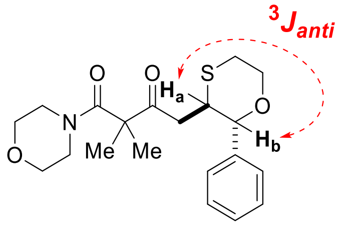

7q

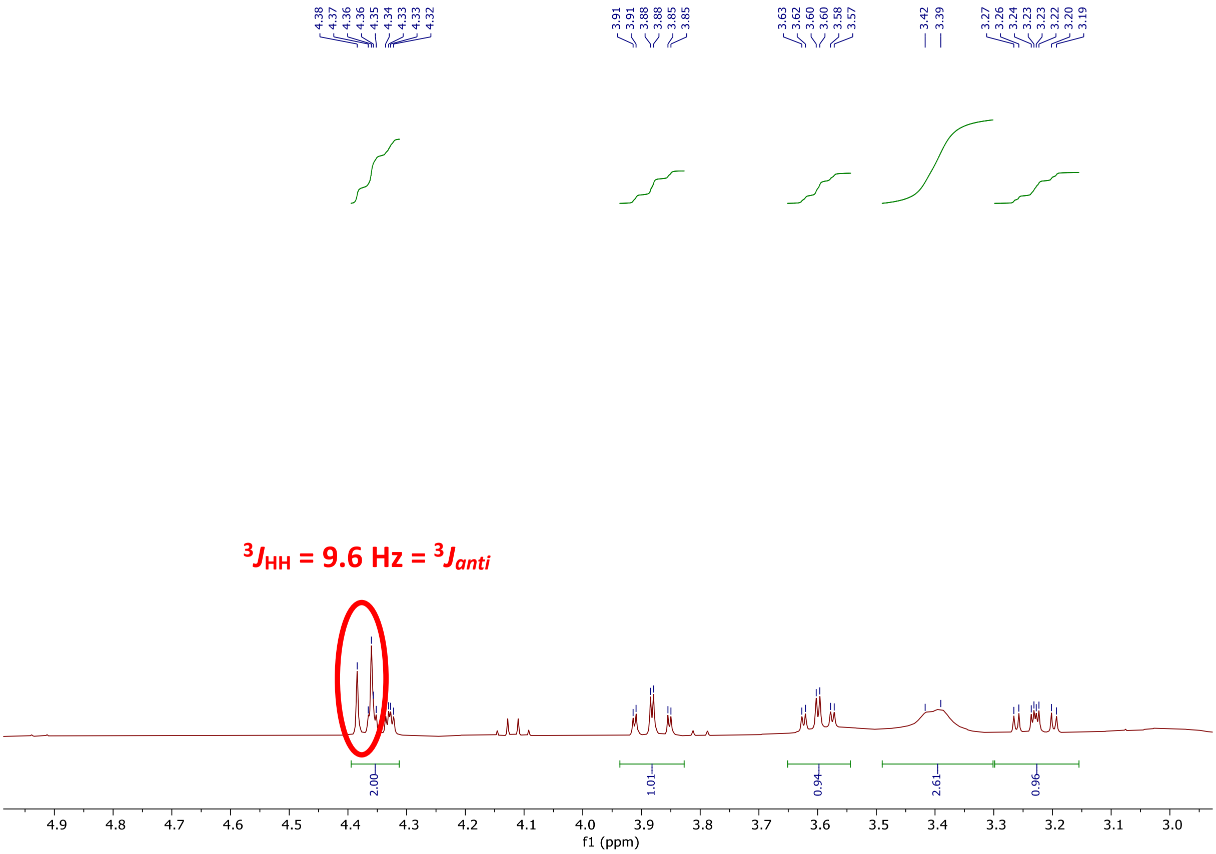

5.2.2 R^2 substituents

The relative configuration for R^2 substituted *S*-heterocycles was determined by the vicinal $^3J_{HH}$ couplings in product **7r**. The coupling value between protons H_a and H_c is 10.6 Hz, and a coupling value of 10.8 Hz is present between protons H_b and H_d. These values correspond with the corresponding $^3J_{HH}$ couplings from R^2 substituted dioxane product **5o**, thus we deduce that an equatorial-equatoral relationship exists between the ring substituents in major diastereoisomer **7r**, leading to and *anti* relative configuration.

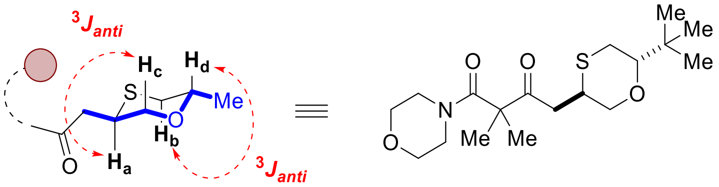

7r

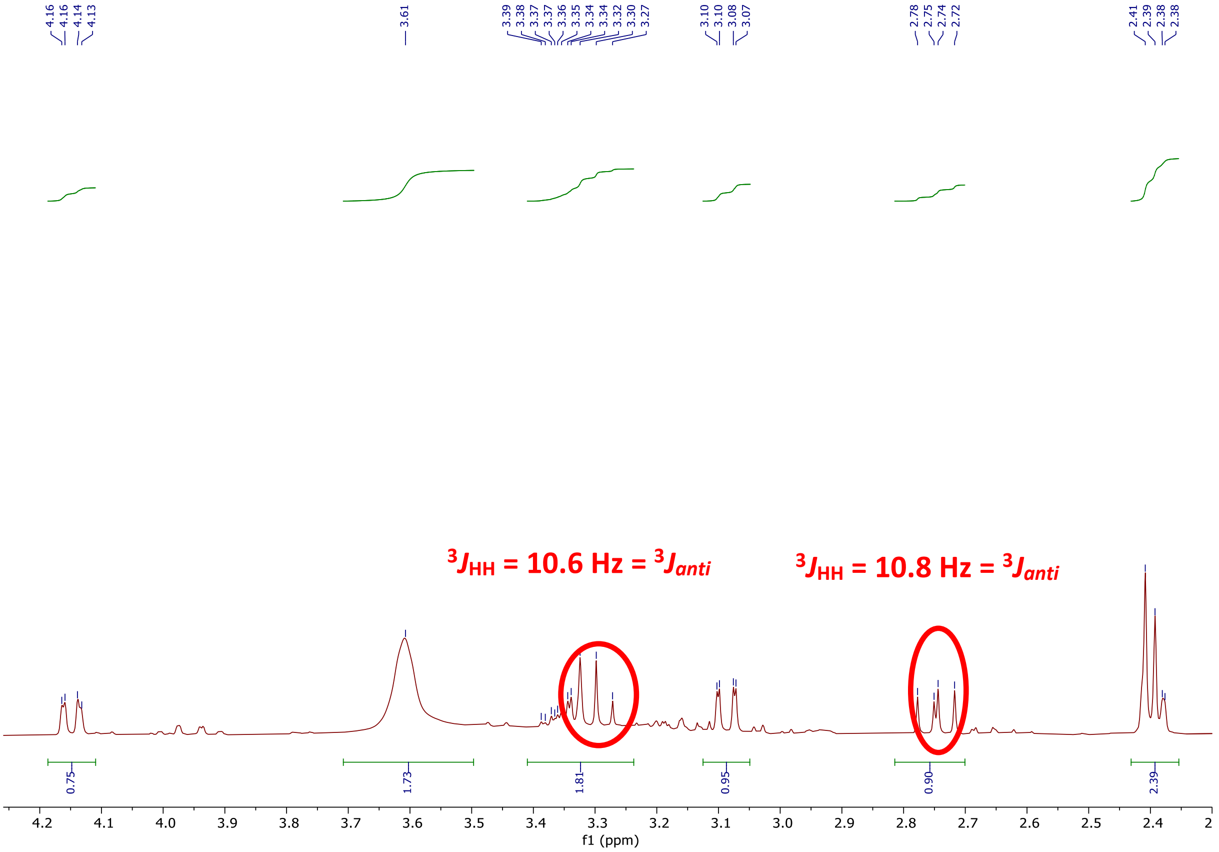

5.2.3 R[1] substituents

For the R[1] substituted *S*-heterocyle **7y**, a $^3J_{HH}$ coupling value between protons H_a and H_c is 11.0 Hz, and a coupling value of 10.5 Hz is present between protons H_b and H_d. The coupling value are similar in magnitude to those of R[2] substituted product **7r**. This suggests that methylene protons H_c and H_d are *anti* to methine protons H_a and H_b, again suggesting that an equatorial-equatoral relationship exists between the ring substituents in major diastereoisomer **7y**, leading to a *syn* relative configuration.

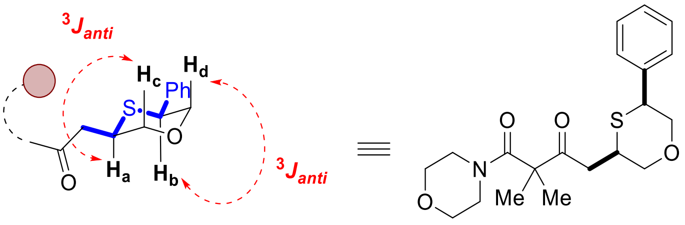

7y

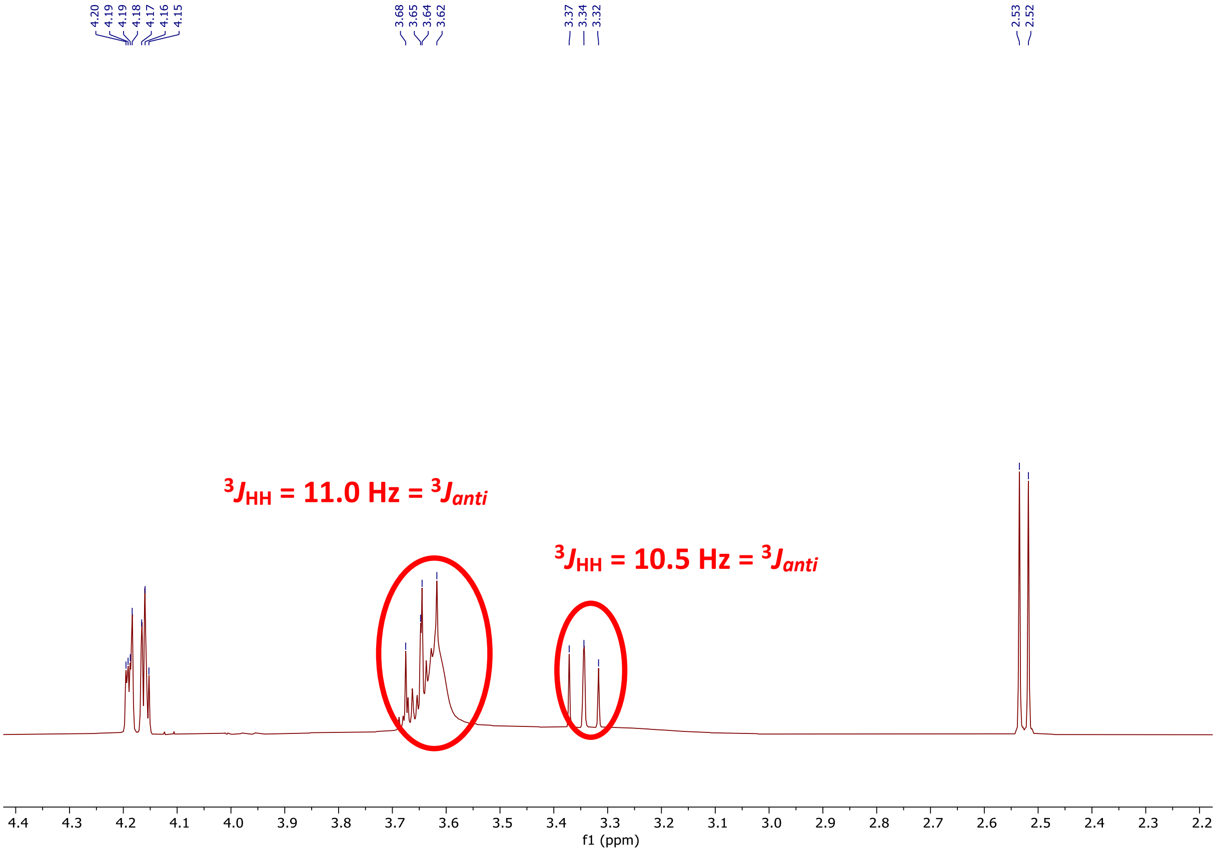

6. *O*- and *N*-heterocycles Substrate Scope

6.1 Scope reaction procedures

General procedure H. $Rh(nbd)_2BF_4$ (2.5-10 mol%) was weighed into a tapered 10 mL microwave vial. Next, ligand (2.5-10 mol%) was introduced and the vial sealed with a suba-seal and parafilmed. The vial was placed under vacuum on a Schlenk line and back-filled with argon. After this, 1.0 mL of distilled solvent (acetone or DCE) was introduced, and the solution sonicated for 30 s. Hydrogen gas was then bubbled through the solution for 1-2 min. The solvent was then removed using a stream of argon and the catalyst system re-dissolved in solvent (0.150 M - 1.00 M). Alkyne (1.20 - 2.00 equiv.) was weighed into a separate concave 10 mL microwave vial along with aldehyde (1.00 equiv.) if a solid. The vial was sealed with a suba-seal/microwave cap and parafilmed (if suba-sealed). It was then placed under vacuum on a Schlenk line, evacuated and back-filled with argon three times. If the aldehyde was a liquid, it was then syringed into the vial post vac-cycle. The catalyst solution was then added to the reactant mixture, and the solution was stirred at 55 °C or rt for 18 h (unless otherwise stated).

Procedures for subsequent steps

General procedure I. A separate oven-dried flask was evacuated and backfilled with argon three-times before adding DCE (4.0 mL/0.3 mmol scale), followed by HCl (4 M in dioxane, 2.00 equiv.). This solution was then added dropwise into the microwave vial containing the crude hydroacylation reaction mixture. The resulting mixture was then stirred at 55 °C for 3 h. If a solvent-switch occurred between **general procedures A and B**, the hydroacylation reaction was cooled to room temperature and concentrated in vacuo. Then a solution of HCl (4 M in dioxane, 2.00 equiv.) in DCE (4.3 mL/0.3 mmol scale) was added to the residue and the mixture was stirred at 55 °C for 3 h. After this time, sat. aq. $NaHCO_3$ (6 mL) was added. The

aqueous layer was extracted with CH_2Cl_2 (3 × 10 mL), dried over anhydrous $MgSO_4$, filtered and concentrated *in vacuo* to yield the crude product.

General procedure J. The crude reaction mixture was diluted with DCE (4.0 mL/0.3 mmol scale), and then *p*-TsOH·H_2O (2.00 equiv.) was added. The resulting mixture was then stirred at 55 °C for 3 h. After this time, sat. aq. $NaHCO_3$ (6 mL) was added. The aqueous layer was exactred with CH_2Cl_2 (3 × 10 mL), dried over anhydrouys $MgSO_4$, filtered and concentrated *in vacuo* to yield the crude product.

6.2 Aldehyde scope compounds

1-(2-(Methylthio)phenyl)-2-((2*R**,3*R**)-3-phenyl-1,4-dioxan-2-yl)ethan-1-one (5a)

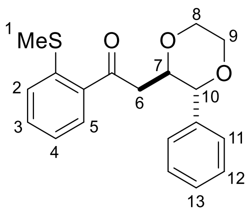

Following **general procedures H** and **I**. **General procedure H** used 2-(methylthio)benzaldehyde **1a** (40 μL, 0.300 mmol, 1.00 equiv.) and 2-((1-phenylprop-2-yn-1-yl)oxy)ethan-1-ol **2b** (63 mg, 0.360 mmol, 1.20 equiv.) with $Rh(nbd)_2BF_4$ (5.6 mg, 0.015 mmol, 0.050 equiv.), dcpe (6.3 mg, 0.015 mmol, 0.050 equiv.) and DCE (0.3 mL). **General procedure I** used HCl in dioxane (4 M in dioxane, 0.15 mL, 0.600 mmol, 2.00 equiv.) and DCE (4.0 mL). Purification of the crude residue (13:1 dr) by column chromatography (1:9 to 1:5 EtOAc:Petrol) yielded the title compound as a single diastereoisomer **5a** (85.9 mg, 0.262 mmol, 87%) as an off-white solid.

m.p. (CH_2Cl_2) 69–71 °C; **δ_H** (400 MHz, $CDCl_3$) 7.65 (1H, dd, *J* = 7.8, 1.5 Hz, *Ar*), 7.46-7.37 (3H, m, *Ar*), 7.38-7.31 (3H, m, *Ar*), 7.31-7.25 (1H, m, *Ar*), 7.12 (1H, td, *J* = 7.6, 1.2 Hz, *Ar*), 4.37 (1H, d, *J* = 9.2 Hz, C(10)*H*), 4.24 (1H, app td, *J* = 9.2, 2.4 Hz, C(7)*H*), 3.95-3.79 (4H, m, C(8 and 9)H_2), 3.11 (1H, dd, *J* = 15.9, 9.2 Hz, C(6)*H*H), 2.61 (1H, dd, *J* = 15.9, 2.4 Hz, C(6)H*H*), 2.38 (3H, s, SC(1)H_3); **δ_C** (101 MHz, $CDCl_3$) 198.4, 142.4, 138.0, 134.9, 132.2, 130.5, 128.8 (2C), 127.9, 125.3, 123.5, 82.7, 77.5, 67.1, 67.1, 41.7, 16.1; **ν_{max}**/ cm^{-1} (neat) 1672, 1433, 1271, 1115, 990, 842, 756, **m/z HRMS** (ESI^+) $[M + H]^+$ $C_{19}H_{21}O_3{}^{32}S^+$ calc. 329.1206, found 329.1203.

Gram-Scale Reaction: Following **general procedures H** and **I**. **General procedure H** used 2-(methylthio)benzaldehyde **1a** (912 mg, 6.00 mmol, 1.00 equiv.) and 2-((1-phenylprop-2-yn-1-yl)oxy)ethan-1-ol **2b** (1.27 g, 7.20 mmol, 1.20 equiv.), with $Rh(nbd)_2BF_4$ (56 mg,

0.150 mmol, 0.025 equiv.), dcpe (63 mg, 0.150 mmol, 0.025 equiv.) and DCE (6.0 mL) in a normal microwave vial. For **general procedure I**, a separate oven-dried round-bottom flask was backfilled under argon three times. The crude reaction mixture from **general procedure H** was transferred over to this flask *via* a syringe. Then, in another oven-dried round-bottom flask, was prepared a degassed (argon) solution of HCl (4 M in dioxane, 3.0 mL, 12.0 mmol, 2.00 equiv.) in DCE (80 mL). This solution was slowly added to the round-bottom flask containing the crude reaction mixture, and the resulting solution was stirred for 3 h at 55 °C (before work-up according to **general procedure B**. Purification of the crude residue (8.5:1 dr) *via* column chromatography (3:17 EtOAc:Petrol), followed by recrystallisation in 1:19 EtOAc:Hexane, yielded title compound **5a** (1.46 g, 5.00 mmol, 74%) as a pale-brown solid. Out of the material obtained, 0.98 g was recrystallised as pure major diastereoisomer, which could be used in subsequent derivatisations (see SI, section **6.5**).

1-(2-(Methylthio)-3-(trifluoromethyl)phenyl)-2-((2*R,3*R**)-3-phenyl-1,4-dioxan-2-yl)ethan-1-one (5b)**

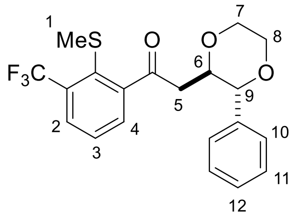

Following **general procedures H** and **I**. **General procedure H** used 2-(methylthio)-3-(trifluoromethyl)benzaldehyde **1b** (66 mg, 0.300 mmol, 1.00 equiv.) and 2-((1-phenylprop-2-yn-1-yl)oxy)ethan-1-ol **2b** (63 mg, 0.360 mmol, 1.20 equiv.) with $Rh(nbd)_2BF_4$ (5.6 mg, 0.015 mmol, 0.050 equiv.), dcpe (6.3 mg, 0.015 mmol, 0.050 equiv.) and DCE (0.3 mL). **General procedure I** used HCl in dioxane (4 M in dioxane, 0.15 mL, 0.600 mmol, 2.00 equiv.) and DCE (4.0 mL). Purification of the crude residue (19:1 dr) by column chromatography (1:9 to

1:5 EtOAc:Petrol) yielded the title compound as a single diastereoisomer **5b** (101 mg, 0.255 mmol, 85%) as a yellow viscous oil.

δ_F (377 MHz, $CDCl_3$) -59.74 (C*F₃*); **δ_H** (400 MHz, $CDCl_3$) 7.73 (1H, dd, *J* = 7.9, 1.5 Hz, *Ar*), 7.49-7.42 (1H, m, *Ar*), 7.38-7.30 (6H, m, *Ar*), 4.29 (1H, d, *J* = 9.1 Hz, C(9)*H*), 4.07 (1H, ddd, *J* = 9.3, 9.1, 2.9 Hz, C(6)*H*), 3.96-3.82 (4H, m, C(7 and 8)H_2), 3.02 (1H, dd, *J* = 16.0, 9.3 Hz, C(5)*H*H), 2.82 (1H, dd, *J* = 16.0, 2.9 Hz, C(5)H*H*), 2.15 (3H, s, SC(1)H_3); **δ_C** (101 MHz, $CDCl_3$) 203.4, 150.9, 137.8, 134.8 (q, J_{CF} = 29.5 Hz), 130.9, 130.1, 129.1, 128.9, 128.8, 127.8 (q, J_{CF} = 5.5 Hz), 127.8, 123.5 (q, J_{CF} = 273.8 Hz), 82.7, 77.5, 67.0 (2C), 45.9, 22.1; **ν_{max}**/cm^{-1} (neat) 2800, 1705, 1317, 1267, 1163, 1114, 910, 731, 700; **m/z HRMS** (ESI^+) $[M + H]^+$ $C_{20}H_{20}O_3F_3{}^{32}S^+$ calc. 397.1080, found 397.1081.

1-(4-Bromo-2-(methylthio)phenyl)-2-((2*R,3*R**)-3-phenyl-1,4-dioxan-2-yl)ethan-1-one (5c)**

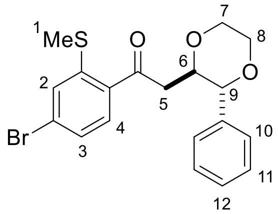

Following **general procedures H** and **I**. **General procedure H** used 4-bromo-2-(methylthio)benzaldehyde **1c** (69 mg, 0.300 mmol, 1.00 equiv.) and 2-((1-phenylprop-2-yn-1-yl)oxy)ethan-1-ol **2b** (63 mg, 0.360 mmol, 1.20 equiv.) with $Rh(nbd)_2BF_4$ (5.6 mg, 0.015 mmol, 0.050 equiv.), dcpe (6.3 mg, 0.015 mmol, 0.050 equiv.) and DCE (0.3 mL). **General rocedure I** used HCl in dioxane (4 M in dioxane, 0.15 mL, 0.600 mmol, 2.00 equiv.) and DCE (4.0 mL). Purification of the crude residue (19:1 dr) by column chromatography (1:12 to 1:7 EtOAc:Petrol) yielded the title compound as a single diastereoisomer **5c** (75.9 mg, 0.187 mmol, 62%) as a pale-brown solid.

m.p. (CH_2Cl_2) 95–96 °C; **δ_H** (400 MHz, $CDCl_3$) 7.49 (1H, d, *J* = 8.4 Hz, *Ar*), 7.42-7.28 (6H, m, *Ar*), 7.24 (1H, dd, *J* = 8.4, 1.8 Hz, *Ar*), 4.35 (1H, d, *J* = 9.1 Hz, C(9)*H*), 4.19 (1H, app td, *J*

= 9.1, 2.5 Hz, C(6)*H*), 3.95-3.76 (4H, m, C(7 and 8)H_2), 3.04 (1H, dd, *J* = 15.7, 9.1 Hz, C(5)*H*H), 2.57 (1H, dd, *J* = 15.7, 2.6 Hz, C(5)H*H*), 2.37 (3H, s, SC(3)H_3); **δ$_C$** (101 MHz, $CDCl_3$) 197.6, 145.2, 138.0, 133.2, 131.8, 128.9, 128.8, 127.9, 127.7, 127.7, 126.5, 82.7, 77.5, 67.1, 67.1, 41.6, 16.2; **ν$_{max}$**/ cm^{-1} (neat) 1673, 1574, 1537, 1437, 1369, 1269, 1114, 990, 942, 799, 726, 648, 624; **m/z HRMS** (ESI$^+$) [M + H]$^+$ $C_{19}H_{20}O_3{}^{79}Br^{32}S^+$ calc. 407.0311, found 407.0311, $C_{19}H_{20}O_3{}^{81}Br^{32}S^+$ calc. 409.0290, found 409.0290.

1-(4,5-Dimethoxy-2-(methylthio)phenyl)-2-((2*R,3*R**)-3-phenyl-1,4-dioxan-2-yl)ethan-1-one (5d)**

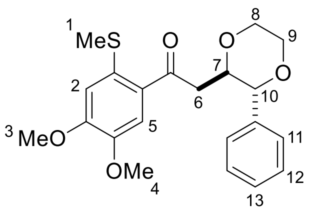

Following **general procedures H** and **I**. **General procedure H** used 4,5-dimethoxy-2-(methylthio)benzaldehyde **1d** (64 mg, 0.300 mmol, 1.00 equiv.) and 2-((1-phenylprop-2-yn-1-yl)oxy)ethan-1-ol **2b** (63 mg, 0.360 mmol, 1.20 equiv.) with $Rh(nbd)_2BF_4$ (5.6 mg, 0.015 mmol, 0.050 equiv.), dcpe (6.3 mg, 0.015 mmol, 0.05 equiv.) and DCE (0.3 mL). **General procedure I** used HCl in dioxane (4 M in dioxane, 0.15 mL, 0.600 mmol, 2.00 equiv.) and DCE (4.0 mL). Purification of the crude residue (>20:1 dr) by column chromatography (1:3 to 2:3 EtOAc:Petrol) yielded the title compound as a single diastereoisomer **5d** (80.0 mg, 0.206 mmol, 67%) as an off-white solid.

m.p. (CH_2Cl_2) 153–155 °C; **δ$_H$** (400 MHz, $CDCl_3$) 7.42-7.39 (2H, m, *Ar*), 7.36-7.29 (3H, m, *Ar*), 7.12 (1H, s, C(2 or 5)*H*), 6.75 (1H, s, C(2 or 5)*H*), 4.36 (1H, d, *J* = 9.1 Hz, C(10)*H*), 4.20 (1H, app td, *J* = 9.2, 2.5 Hz, C(7)*H*), 3.92 (3H, s, C(3 or 4)H_3), 3.91-3.78 (4H, m, C(8 and 9)H_2), 3.81 (3H, s, C(3 or 4)H_3), 3.02 (1H, dd, *J* = 15.5, 9.2 Hz, C(6)*H*H), 2.65 (1H, dd, *J* = 15.5, 2.5 Hz, C(6)H*H*), 2.36 (3H, s, SC(1)H_3); **δ$_C$** (101 MHz, $CDCl_3$) 197.0, 152.4, 145.6, 138.1, 135.9, 128.7 (2C), 128.1, 127.9, 113.8, 108.9, 82.7, 77.6, 67.1, 67.1, 56.4, 56.0, 41.9,

16.9; **ν_{max}**/ cm^{-1} (neat) 2852, 2361, 1660, 1598, 1552, 1502, 1437, 1378, 1336, 1266, 1202, 1167, 1113, 914, 763, 729, 702; **m/z HRMS** (ESI$^+$) [M + H]$^+$ $C_{21}H_{25}O_5{}^{32}S^+$ calc. 389.1417, found 389.1417.

1-(3-(Methylthio)thiophen-2-yl)-2-((2*R,3*R**)-3-phenyl-1,4-dioxan-2-yl)ethan-1-one (5e)**

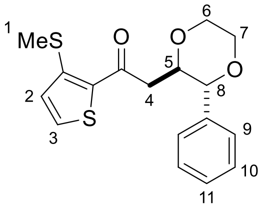

Following **general procedures H** and **I**. **General procedure H** used 3-(methylthio)thiophene-2-carbaldehyde **1e** (48 mg, 0.300 mmol, 1.00 equiv.) and 2-((1-phenylprop-2-yn-1-yl)oxy)ethan-1-ol **2b** (63 mg, 0.360 mmol, 1.20 equiv.) with $Rh(nbd)_2BF_4$ (5.6 mg, 0.015 mmol, 0.050 equiv.), dcpe (6.3 mg, 0.015 mmol, 0.050 equiv.) and DCE (0.3 mL). **General procedure I** used HCl in dioxane (4 M in dioxane, 0.15 mL, 0.600 mmol, 2.00 equiv.) and DCE (4.0 mL). Purification of the crude residue (>20:1 dr) by column chromatography (1:9 to 1:5 EtOAc:Petrol) yielded the title compound as a single diastereoisomer **5e** (95.3 mg, 0.285 mmol, 95%), as an orange/brown solid.

m.p. (CH_2Cl_2) 113–117 °C; **δ_H** (400 MHz, $CDCl_3$) 7.47 (1H, d, *J* = 5.2 Hz, C(3)H), 7.42-7.38 (2H, m, *Ar*), 7.37-7.25 (3H, m, *Ar*), 6.96 (1H, d, *J* = 5.2 Hz, C(2)H), 4.38 (1H, d, *J* = 9.2 Hz, C(8)*H*), 4.25 (1H, td, *J* = 9.0, 2.6 Hz, C(5)*H*), 3.98-3.74 (4H, m, C(6 and 7)H_2), 2.91 (1H, dd, *J* = 15.6, 8.9 Hz, C(4)*H*H), 2.53 (1H, dd, *J* = 15.6, 2.6 Hz, C(4)H*H*), 2.47 (3H, s, SC(1)H_3); **δ_C** (101 MHz, $CDCl_3$) 188.9, 146.3, 137.9, 131.2, 130.9, 128.7, 128.7, 127.8, 126.2, 82.5, 77.2, 67.0 (2C), 42.6, 16.6; **ν_{max}**/ cm^{-1} (neat) 2981, 2856, 1682, 1594, 1490, 1454, 1433, 1378, 1326, 1284, 1269, 1115, 944, 757, 702, 647, 622; **m/z HRMS** (ESI$^+$) $C_{17}H_{18}O_3{}^{23}Na^{32}S_2{}^+$ [M + Na]$^+$ calc. 357.0590, found 357.0590.

1-(2-(Methylthio)cyclohex-1-en-1-yl)-2-((2*R,3*R**)-3-phenyl-1,4-dioxan-2-yl)ethan-1-one (5f)**

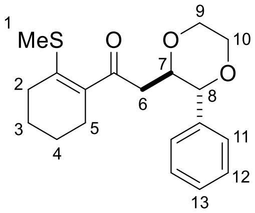

Following **general procedure H** ***(general procedure I was not pursued for this example)***, using 2-(methylthio)cyclohex-1-ene-1-carbaldehyde **1f** (47 mg, 0.300 mmol, 1.00 equiv.) and 2-((1-phenylprop-2-yn-1-yl)oxy)ethan-1-ol **2b** (63 mg, 0.360 mmol, 1.20 equiv.) with $Rh(nbd)_2BF_4$ (5.6 mg, 0.015 mmol, 0.050 equiv.), dcpe (6.3 mg, 0.015 mmol, 0.050 equiv.) and DCE (0.3 mL), purification of the crude residue (3.4:1 dr) by column chromatography (1:9 to 1:4 EtOAc:Petrol) yielded the title compound as an inseparable mixture of diastereoisomers **5f** (90.1 mg, 0.271 mmol, 90%) as a yellow viscous oil.

δ_H (400 MHz, Acetone-d$_6$) (major signals only) 7.46-7.37 (2H, m, *Ar*), 7.41-7.21 (3H, m, *Ar*), 4.30 (1H, d, *J* = 9.1 Hz, C(8)*H*), 4.01 (1H, td, *J* = 9.2, 2.5 Hz, C(7)*H*), 3.86-3.70 (4H, m, C(9 and 10)*H*$_2$), 2.70 (1H, dd, *J* = 15.8, 9.3 Hz, C(6)*H*H), 2.42-2.35 (2H, m, C(2 and 5)*H*$_2$), 2.34-2.14 (3H, m, C(2 and 5)*H*$_2$ and C(6)H*H*), 2.09 (3H, s, C(1)*H*$_3$), 1.69-1.51 (4H, m, C(3 or 4)*H*$_2$); **δ_C** (101 MHz, Acetone-d$_6$) (major signals only) 199.5, 145.4, 139.8, 132.8, 129.1, 129.0, 128.7, 83.2, 78.6, 67.5, 67.5, 42.8, 30.2, 28.5, 23.7, 22.6, 14.5; **ν_{max}**/ cm^{-1} (neat) 2925, 2855, 1700, 1651, 1518, 1449, 1417, 1268, 1246, 1136, 1113, 1011, 985, 943, 894, 760, 701, 650, 624; **m/z HRMS** (ESI$^+$) [M + H]$^+$ $C_{19}H_{25}O_3{}^{32}S^+$ calc. 333.1519, found 333.1520.

1-(4-(Methylthio)-1-tosyl-1,2,5,6-tetrahydropyridin-3-yl)-2-((2*R,3*R**)-3-phenyl-1,4-dioxan-2-yl)ethan-1-one (5g)**

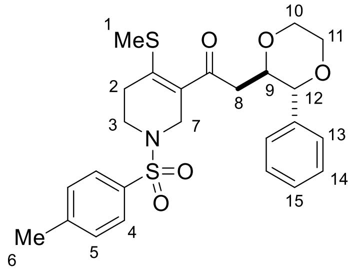

Following **general procedure H** ***(general procedure I was not pursued for this example)***, using 4-(methylthio)-1-tosyl-1,2,5,6-tetrahydropyridine-3-carbaldehyde **1g** (62 mg, 0.200 mmol, 1.00 equiv.) and 2-((1-phenylprop-2-yn-1-yl)oxy)ethan-1-ol **2b** (42 mg, 0.240 mmol, 1.20 equiv.) with $Rh(nbd)_2BF_4$ (3.7 mg, 0.010 mmol, 0.050 equiv.), dcpe (4.2 mg, 0.010 mmol, 0.050 equiv.) and DCE (0.2 mL), purification of the crude residue (7:1 dr) by column chromatography (3:7 EtOAc:Petrol) yielded the title compound as an inseparable mixture of diastereoisomers **5g** (76.3 mg, 0.157 mmol, 78%) as an off-white solid.

m.p. (CH_2Cl_2) 65–71 °C; δ_H (400 MHz, $CDCl_3$) (major signals only) 7.73-7.68 (2H, m, *Ar*), 7.44-7.29 (7H, m, *Ar*), 4.34 (1H, d, *J* = 9.2 Hz, C(12)*H*), 4.16 (1H, app td, *J* = 9.2, 2.6 Hz, C(9)*H*), 3.96-3.75 (6H, m, C(10 and 11)H_2 and C(7)H_2), 3.32-3.22 (1H, m, C(3)*H*H), 3.18-3.09 (1H, m, C(3)H*H*), 2.61 (1H, dd, *J* = 15.9, 9.1 Hz, C(8)*H*H), 2.60-2.55 (2H, m, C(2)H_2), 2.46 (3H, s, C(6)H_3), 2.28 (1H, dd, *J* = 15.8, 2.5 Hz, C(8)H*H*), 2.18 (3H, s, C(1)H_3); δ_C (126 MHz, $CDCl_3$) (major signals only) 195.5, 148.7, 144.1, 137.9, 133.4, 129.9, 128.7, 127.8, 127.8 (2C), 125.7, 82.6, 77.4, 67.0, 67.0, 46.4, 42.6, 41.8, 29.8, 21.6, 14.6; ν_{max}/ cm^{-1} (neat) 3033, 2921, 1652, 1526, 1341, 1164, 1113, 939, 914, 758, 730, 703, 657; **m/z HRMS** (ESI^+) $[M + H]^+$ $C_{25}H_{30}O_5N^{32}S_2^+$ calc. 488.1560, found 488.1558.

1-(4-(Methylthio)-5,6-dihydro-2*H*-pyran-3-yl)-2-((2*R,3*R**)-3-phenyl-1,4-dioxan-2-yl)ethan-1-one (5h)**

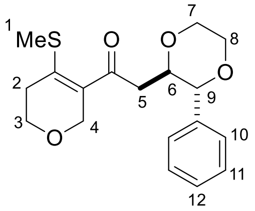

Following **general procedure H** ***(general procedure I was not pursued for this example)***, 4-(methylthio)-5,6-dihydro-2*H*-pyran-3-carbaldehyde **1h** (47 mg, 0.300 mmol, 1.00 equiv.) and 2-((1-phenylprop-2-yn-1-yl)oxy)ethan-1-ol **2b** (63 mg, 0.360 mmol, 1.20 equiv.) with $Rh(nbd)_2BF_4$ (5.6 mg, 0.015 mmol, 0.050 equiv.), dcpe (6.3 mg, 0.015 mmol, 0.050 equiv.) and DCE (0.3 mL), purification of the crude residue (4:1 dr) by column chromatography (1:4 to 1:1 EtOAc:Petrol) yielded the title compound as an inseparable mixture of diastereoisomers **5h** (90.9 mg, 0.272 mmol, 91%) as an orange powdered solid.

m.p. (CH_2Cl_2) 102–104 °C; **δ_H** (400 MHz, $CDCl_3$) (major signals only) 7.38-7.28 (5H, m, *Ar*), 4.42 (1H, app dt, J = 14.7, 2.2 Hz, C(4)*H*H), 4.33-4.26 (2H, m, C(4)H*H* and C(9)*H*), 4.16 (1H, app td, J = 9.0, 2.5 Hz, C(6)*H*), 4.01-3.68 (6H, m, C(3)H_2 and C(7 and 8)H_2), 2.53-2.44 (3H, m, C(2)H_2 and C(5)*H*H), 2.21 (3H, s, SC(1)H_3), 2.17-2.09 (1H, dd, J = 15.9, 2.5 Hz, C(5)H*H*); **δ_C** (126 MHz, $CDCl_3$) (major signals only) 194.3, 150.4, 138.0, 128.7, 127.8, 126.9, 126.3, 82.6, 77.0, 67.0, 66.9, 63.9, 41.1, 29.3, 29.2, 14.1; **ν_{max}**/ cm^{-1} (neat) 2855, 1650, 1528, 1453, 1412, 1351, 1270, 1145, 1112, 1013, 943, 914, 898, 762, 729, 701, 650; **m/z HRMS** (ESI^+) $C_{18}H_{22}O_4{}^{23}Na^{32}S^+$ $[M + Na]^+$ calc. 357.1131, found 357.1132.

4-(Methylthio)-1-((2*R,3*R**)-3-phenyl-1,4-dioxan-2-yl)butan-2-one (5i)**

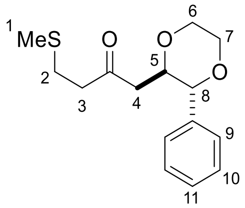

Following **general procedures H** and **I**. **General procedure H** used 3-(methylthio)propanal **1i** (30 μL, 0.300 mmol, 1.00 equiv.) and 2-((1-phenylprop-2-yn-1-yl)oxy)ethan-1-ol **2b** (106 mg, 0.600 mmol, 2.00 equiv.) with Rh(nbd)$_2$BF$_4$ (5.6 mg, 0.015 mmol, 0.050 equiv.) and dppe (6.0 mg, 0.015 mmol, 0.050 equiv.) ***in acetone (2.0 mL), with stirring in procedure A for 2 h before addition of acid. It is important to note that a solvent-switch also occurred between procedures H and I for this example.*** **General procedure H** used HCl in dioxane (4 M in dioxane, 0.15 mL, 0.600 mmol, 2.00 equiv.) and DCE (4.3 mL). Purification of the crude residue (8.5:1 dr) by column chromatography (1:19 to 1:5 EtOAc:Petrol) yielded the title compound as an inseparable mixture of diastereoisomers **5i** (58.2 mg, 0.208 mmol, 69%) as a viscous yellow oil.

δ_H (400 MHz, CDCl$_3$) (major signals only) 7.37-7.29 (5H, m, *Ar*), 4.24 (1H, d, *J* = 9.1 Hz, C(8)*H*), 4.01 (1H, app td, *J* = 9.1, 3.1 Hz, C(5)*H*), 3.93-3.78 (4H, m, C(6 and 7)*H*$_2$), 2.65-2.59 (4H, m, C(2 and 3)*H*$_2$), 2.49 (1H, dd, *J* = 15.5, 9.1 Hz, C(4)*H*H), 2.15 (1H, dd, *J* = 15.5, 3.1 Hz, C(4)H*H*), 2.04 (3H, s, C(1)*H*$_3$); **δ_C** (101 MHz, CDCl$_3$) (major signals only) 206.4, 137.8, 128.8, 128.7, 127.7, 82.7, 77.1, 67.0, 67.0, 44.7, 43.6, 27.6, 15.8; **ν_{max}**/ cm^{-1} (neat) 3657, 2980, 2913, 1715, 1494, 1380, 1268, 1114, 943, 758, 701, 649; **m/z HRMS** $C_{15}H_{20}O_3{}^{23}Na^{32}S^+$ [M + Na]$^+$ calc. 303.1025, found 303.1025.

1-(2-(Dimethylamino)phenyl)-2-((2*R,3*R**)-3-phenyl-1,4-dioxan-2-yl)ethan-1-one (5j)**

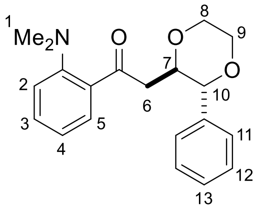

Following **general procedures H** and **I**. **General procedure H** used 2-(dimethylamino)benzaldehyde **1j** (44.7 mg, 0.300 mmol, 1.00 equiv.) and 2-((1-phenylprop-2-yn-1-yl)oxy)ethan-1-ol **2b** (63 mg, 0.360 mmol, 1.20 equiv.) with $Rh(nbd)_2BF_4$ (11.2 mg, 0.030 mmol, 0.100 equiv.) and dcpm (11.5 mg, 0.030 mmol, 0.100 equiv.) ***in acetone (2 mL), with stirring in procedure A for 4 h before addition of acid. It is important to note that a solvent-switch (acetone to DCE) also occurred between procedures H and I for this example.*** **General procedure I** used HCl in dioxane (4 M in dioxane, 0.15 mL, 0.600 mmol, 2.00 equiv.) and DCE (4.3 mL). Purification of the crude residue (5.8:1 dr) by column chromatography (1:19 to 1:5 EtOAc:Petrol) yielded the title compound as an inseparable mixture of diastereoisomers **5j** (92.3 mg, 0.284 mmol, 95%) as an orange/yellow solid.

m.p. (CH_2Cl_2) 36–38 °C; **δ_H** (400 MHz, MeOD) (major signals only) 7.38-7.27 (6H, m, *Ar*), 7.16 (1H, dd, *J* = 5.9, 1.8 Hz, *Ar*), 6.99 (1H, dd, *J* = 8.3, 0.8 Hz, *Ar*), 6.90 (1H, app td, *J* = 7.4, 1.1 Hz, *Ar*), 4.26 (1H, d, *J* = 9.1 Hz, C(10)*H*), 3.97-3.59 (7H, m, C(7)*H*, C(8, 9 and 6)H_2), 2.46 (6H, s, NC(1)H_3); **δ_C** (126 MHz, MeOD) (major signals only) 206.1, 153.1, 139.7, 133.7, 133.1, 130.4, 129.6, 129.5, 129.4, 128.9, 121.8, 118.5, 83.8, 79.0, 68.0, 67.9, 44.7; **ν_{max}**/ cm^{-1} (neat) 2981, 2856, 1682, 1594, 1490, 1454, 1433, 1378, 1284, 1269, 1158, 1115, 970, 945, 757, 702; **m/z HRMS** (ESI^+) $C_{20}H_{24}O_3N^+$ $[M + H]^+$ calc. 326.1751, found 326.1749.

2,2-dimethyl-1-morpholino-4-((2*R,3*R**)-3-phenyl-1,4-dioxan-2-yl)butane-1,3-dione (5k)**

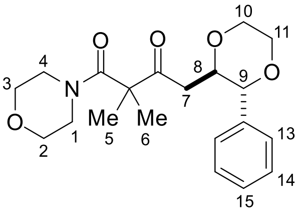

Following **general procedures H** and **I**. **General procedure H** used 2,2-dimethyl-3-morpholino-3-oxopropanal **1k** (55.5 mg, 0.300 mmol, 1.00 equiv.) and 2-((1-phenylprop-2-yn-1-yl)oxy)ethan-1-ol **2b** (63 mg, 0.360 mmol, 1.20 equiv.) with $Rh(nbd)_2BF_4$ (5.6 mg, 0.015 mmol, 0.050 equiv.), dcpe (6.3 mg, 0.015 mmol, 0.050 equiv.) and DCE (0.3 mL). **General procedure I** used HCl in dioxane (4 M in dioxane, 0.15 mL, 0.600 mmol, 2.00 equiv.) and DCE (4.0 mL). Purification of the crude residue (>20:1 dr) by column chromatography (1:4 to 1:1 EtOAc:Petrol) yielded the title compound as a single diastereoisomer **5k** (100 mg, 0.277 mmol, 92%) as a colourless crystalline solid.

m.p. (CH_2Cl_2) 124–126 °C; **δ_H** (400 MHz, $CDCl_3$) 7.32 (5H, s, *Ar*), 4.23 (1H, d, *J* = 9.3 Hz, C(9)*H*), 4.10 (1H, app td, 9.3, 1.9 Hz, C(8)*H*), 3.92-3.73 (4H, m, C(10 and 11)H_2), 3.70-2.80 (8H, br, C(1, 2, 3 and 4)H_2), 2.63 (1H, dd, *J* = 17.4, 9.4 Hz, C(7)*H*H), 2.06 (1H, dd, *J* = 17.4, 1.9 Hz, C(7)H*H*), 1.28 (3H, s, C(5 or 6)H_3), 1.22 ((3H, s, C(5 or 6)H_3); **δ_C** (101 MHz, $CDCl_3$) 207.3, 170.9, 137.9, 129.0, 128.8, 127.8, 82.3, 76.1, 67.1, 66.9, 66.4 (bs, 2C), 55.9, 46.4 (bs), 43.3 (bs), 39.6, 23.3, 23.1; **ν_{max}**/ cm^{-1} (neat) 2856, 1713, 1637, 1423, 1271, 1250, 1115, 1025, 943, 761, 705; **m/z HRMS** (ESI^+) $[M + H]^+$ $C_{20}H_{28}O_5N^+$ calc. 362.1962, found 362.1955.

(*R)-2-Benzyl-1-morpholino-4-((2*R**,3*R**)-3-phenyl-1,4-dioxan-2-yl)butane-1,3-dione (5l) and (*S**)-2-benzyl-1-morpholino-4-((2*R**,3*R**)-3-phenyl-1,4-dioxan-2-yl)butane-1,3-dione (5l')**

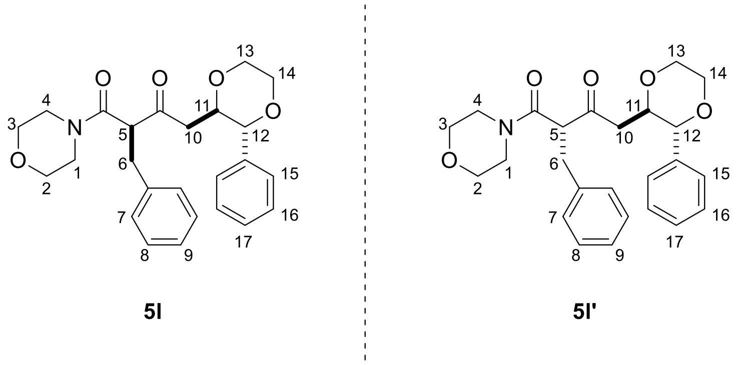

Following **general procedures H** and **I**. **General procedure H** used 2-benzyl-1,3-dimorpholinopropane-1,3-dione **1l** (74 mg, 0.300 mmol, 1.00 equiv.) and 2-((1-phenylprop-2-yn-1-yl)oxy)ethan-1-ol **2b** (63 mg, 0.360 mmol, 1.20 equiv.) with Rh(nbd)$_2$BF$_4$ (5.6 mg, 0.015 mmol, 0.050 equiv.) and dcpe (6.3 mg, 0.015 mmol). **General procedure I** used HCl in dioxane (4 M in dioxane, 0.15 mL, 0.600 mmol, 2.00 equiv.) and DCE (4.0 mL). Purification of the crude residue (1:1 dr) by column chromatography (1:1 to 100:0 EtOAc:Petrol) yielded the title compound as an inseparable mixture of diastereoisomers **5l** and **5l'** (81.1 mg, 0.191 mmol, 64%) as a yellow viscous oil.

δ_H (400 MHz, CDCl$_3$) (1:1 mixture) 7.37-7.29 (9H, m, *Ar*), 7.27-7.16 (7H, m, *Ar*), 7.14-7.05 (4H, m, *Ar*), 4.26 (1H, d, *J* = 9.1 Hz), 4.21 (1H, d, *J* = 9.1 Hz), 4.04-3.72 (12H, m), 3.61-2.91 (20H, m), 2.61 (1H, dd, *J* = 15.6, 10.1 Hz 1H), 2.58 (1H, dd, *J* = 15.9, 8.5 Hz), 2.19 (1H, dd, *J* = 15.8, 3.4 Hz), 2.10 (1H, dd, *J* = 15.4, 2.4 Hz); **δ_C** (101 MHz, CDCl$_3$) (1:1 mixture) 203.0, 202.8, 167.2, 167.2, 138.5, 138.4, 137.7, 137.6, 129.1, 129.0, 128.9, 128.8, 128.8, 128.7, 128.7, 128.7, 127.7, 127.7, 126.8, 126.8, 82.6, 82.3, 77.6, 77.4, 77.0, 67.0, 67.0, 66.9, 66.9, 66.7, 66.6, 66.2, 59.6, 58.6, 46.5, 46.3, 42.6, 42.5, 41.7, 34.6, 34.4; (Proton and Carbon NMR data too

complex to fully assign); **ν_{max}**/ cm^{-1} (neat) 1720, 1635, 1441, 1110, 911, 730; **m/z HRMS** (ESI$^+$) $[M + H]^+$ $C_{25}H_{30}O_5N^+$ calc. 424.2119, found 424.2112.

6.3 Alkyne Scope compounds

2-((2*R,3*R**)-3-Methyl-1,4-dioxan-2-yl)-1-(2-(methylthio)phenyl)ethan-1-one (5m)**

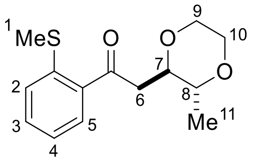

Following **general procedures H** and **I**. **General procedure H** used 2-(methylthio)benzaldehyde **1a** (40 μL, 0.300 mmol, 1.00 equiv.) and 2-(but-3-yn-2-yloxy)ethan-1-ol **2c** (41 mg, 0.360 mmol, 1.20 equiv.) with $Rh(nbd)_2BF_4$ (5.6 mg, 0.015 mmol, 0.050 equiv.), dcpe (6.3 mg, 0.015 mmol, 0.050 equiv.) and DCE (0.3 mL). **General procedure I** used HCl in dioxane (4 M in dioxane, 0.15 mL, 0.600 mmol, 2.00 equiv.) and DCE (4.0 mL). Purification of the crude residue (9:1 dr) by column chromatography (1:12 to 1:5 EtOAc:Petrol) yielded the title compound as an inseparable mixture of diastereoisomers **5m** (67.5 mg, 0.254 mmol, 85%) as a viscous yellow oil.

δ_H (400 MHz, $CDCl_3$) (major signals only) 7.90-7.73 (1H, m, *Ar*), 7.50-7.40 (1H, m, *Ar*), 7.34-7.29 (1H, m, *Ar*), 7.24-7.09 (1H, m, *Ar*), 3.85 (1H, ddd, *J* = 8.9, 8.1, 3.6 Hz, C(7)*H*), 3.75-3.65 (4H, m, C(9 and 10)H_2), 3.46 (1H, dq, *J* = 8.9, 6.3 Hz, C(8)*H*), 3.13 (1H, dd, *J* = 15.8, 8.1 Hz, C(6)*H*H), 2.90 (1H, dd, *J* = 15.8, 3.6 Hz, C(6)H*H*), 2.42 (3H, s, SC(1)H_3), 1.15 (3H, d, *J* = 6.3 Hz, C(11)H_3); **δ_C** (101 MHz, $CDCl_3$) (major signals only) 198.8, 142.4, 135.2, 132.3, 130.6, 125.4, 123.7, 77.9, 75.5, 67.0, 66.9, 42.6, 17.6, 16.2; **ν_{max}**/ cm^{-1} (neat) 2854, 1671, 1587, 1558, 1433, 1355, 1316, 1274, 1199, 1113, 1073, 1050, 1017, 987, 923, 902, 849, 754, 693, 628; **m/z HRMS** (ESI^+) $[M + Na]^+$ $C_{14}H_{18}O_3{}^{23}Na^{32}S^+$ calc. 289.0869, found 289.0868.

1-(2-(Methylthio)phenyl)-2-((2*R,3*R**)-3-pentyl-1,4-dioxan-2-yl)ethan-1-one (5n)**

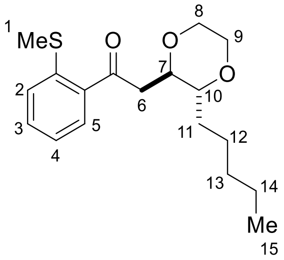

Following **general procedures H** and **I**. **General procedure H** used 2-(methylthio)benzaldehyde **1a** (40 μL, 0.300 mmol, 1.00 equiv.) and 2-(oct-1-yn-3-yloxy)ethan-1-ol **2d** (61 mg, 0.360 mmol, 1.20 equiv.) with $Rh(nbd)_2BF_4$ (5.60 mg, 0.015 mmol, 0.050 equiv.), dcpe (6.30 mg, 0.015 mmol, 0.050 equiv.) and DCE (0.3 mL). **General procedure I** used HCl in dioxane (4 M in dioxane, 0.15 mL, 0.600 mmol, 2.00 equiv.) and DCE (4.0 mL). Purification of the crude residue (8:1 dr) by column chromatography (1:12 to 1:5 EtOAc:Petrol) yielded the title compound as a single diastereoisomer **5n** (72.6 mg, 0.225 mmol, 75%) as a yellow viscous oil.

δ_H (400 MHz, $CDCl_3$) 7.81 (1H, dd, *J* = 7.8, 1.5 Hz, *Ar*), 7.45 (1H, ddd, *J* = 8.1, 7.3, 1.5 Hz, *Ar*), 7.31 (1H, dd, *J* = 8.2, 1.1 Hz, *Ar*), 7.18 (1H, ddd, *J* = 8.0, 7.4, 1.2 Hz, *Ar*), 3.92 (1H, ddd, *J* = 9.0, 8.1, 3.6 Hz, C(7)*H*), 3.76-3.63 (4H, m, C(8)H_2 and C(9)H_2), 3.35-3.25 (1H, ddd, 9.0, 6.7, 4.5 Hz, C(10)*H*), 3.12 (1H, dd, *J* = 15.7, 8.2 Hz, C(6)*H*H), 2.91 (1H, dd, *J* = 15.7, 3.5 Hz, C(6)H*H*), 2.42 (3H, s, SC(1)H_3), 1.61-1.14 (8H, m, C(11, 12, 13 and 14)H_2), 0.87 (3H, t, *J* = 6.9 Hz, C(15)H_3); **δ_C** (101 MHz, $CDCl_3$) 199.0, 142.3, 135.3, 132.2, 130.6, 125.4, 123.7, 79.3, 76.7, 66.9, 66.9, 42.6, 32.0, 31.5, 24.9, 22.7, 16.2, 14.2; **ν_{max}**/ cm^{-1} (neat) 2980, 2956, 2856, 2360, 1673, 1587, 1559, 1463, 1433, 1379, 1273, 1116, 929, 753, 631; **m/z HRMS** (ESI^+) $[M + Na]^+$ $C_{18}H_{26}O_3{}^{23}Na^{32}S^+$ calc. 345.1495, found 345.1496.

2-((2*R,5*S**)-5-Methyl-1,4-dioxan-2-yl)-1-(2-(methylthio)phenyl)ethan-1-one (5o)**

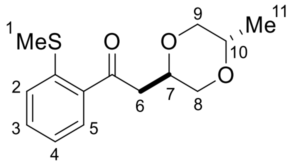

Following **general procedures H** and **I**. **General procedure H** used 2-(methylthio)benzaldehyde **1a** (40 μL, 0.300 mmol, 1.00 equiv.) and 2-(prop-2-yn-1-yloxy)propan-1-ol **2e** (41 mg. 0.360 mmol, 1.20 equiv.) with $Rh(nbd)_2BF_4$ (5.6 mg, 0.015 mmol, 0.050 equiv.), dcpe (6.3 mg, 0.015 mmol, 0.050 equiv.) and DCE (0.3 mL). **General procedure I** used HCl in dioxane (4 M in dioxane, 0.15 mL, 0.600 mmol, 2.00 equiv.) and DCE (4.0 mL). Purification of the crude residue (8:1 dr) by column chromatography (1:5 EtOAc:Petrol) yielded the title compound as an inseparable mixture of diastereoisomers **5o** (64.4 mg, 0.242 mmol, 81%) as an off-white powdered solid.

m.p. (CH_2Cl_2) 77–80 °C; **δ_H** (400 MHz, $CDCl_3$) (major signals only) 7.80 (1H, dd, *J* = 7.9, 1.5 Hz, *Ar*), 7.48-7.41 (1H, m, *Ar*), 7.34-7.28 (1H, m, *Ar*), 7.17 (1H, ddd, *J* = 7.8, 7.2, 1.2 Hz, *Ar*), 4.12 (1H, dddd, *J* = 10.3, 6.6, 5.9, 2.6 Hz, C(7)*H*), 3.91 (1H, dd, *J* = 11.3, 2.6 Hz, C(8)*H*H), 3.70 (1H, dd, *J* = 11.4, 2.6 Hz, C(9)*H*H), 3.66-3.56 (1H, m, C(10)*H*), 3.41 (1H, dd, *J* = 11.4, 10.3 Hz, C(8)H*H*), 3.31 (1H, dd, *J* = 11.5, 10.1 Hz, C(9)H*H*), 3.20 (1H, dd, *J* = 16.3, 6.6 Hz, C(6)*H*H), 2.82 (1H, dd, *J* = 16.3, 5.9 Hz, C(6)H*H*), 2.41 (3H, s, SC(1)H_3), 1.06 (3H, d, *J* = 6.3 Hz, C(11)H_3); **δ_C** (101 MHz, $CDCl_3$) (major signals only) 198.2, 142.6, 134.5, 132.4, 130.5, 125.3, 123.6, 72.6, 71.5, 71.1, 71.0, 42.2, 16.8, 16.1; **ν_{max}**/ cm^{-1} (neat) 2972, 2898, 2361, 1671, 1434, 1276, 1100, 753, 645; **m/z HRMS** (ESI^+) $[M + H]^+$ $C_{14}H_{19}O_3{}^{32}S^+$ calc. 267.1049, found 267.1049.

2-((2*R,5*S**)-5-Butyl-1,4-dioxan-2-yl)-1-(2-(methylthio)phenyl)ethan-1-one (5p)**

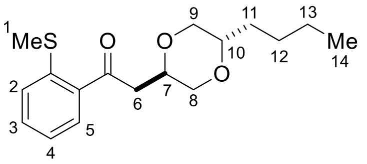

Following **general procedures H** and **I**. **General procedure H** used 2-(methylthio)benzaldehyde **1a** (40 µL, 0.300 mmol, 1.00 equiv.) and the 9:1 inseparable mixture of 2-(prop-2-yn-1-yloxy)hexan-1-ol **2f** and 2-(allyloxy)hexan-1-ol **S9** (62 mg, 0.400 mmol, 1.33 equiv.) with $Rh(nbd)_2BF_4$ (5.6 mg, 0.015 mmol, 0.050 equiv.), dcpe (6.3 mg, 0.015 mmol, 0.050 equiv.) and DCE (0.3 mL). **General procedure I** used HCl in dioxane (4 M in dioxane, 0.15 mL, 0.600 mmol, 2.00 equiv.) and DCE (4.0 mL). Purification of the crude residue (10:1 dr) by column chromatography (1:13 to 1:9 EtOAc:Petrol) yielded the title compound as an inseparable mixture of diastereoisomers **5p** (78.1 mg, 0.253 mmol, 85%) as an off-white powdered solid.

m.p. (CH_2Cl_2) 46–48 °C; **δ_H** (400 MHz, $CDCl_3$) (major signals only) 7.81 (1H, dd, *J* = 7.9, 1.5 Hz, *Ar*), 7.50-7.42 (1H, m, *Ar*), 7.35-7.28 (1H, m, *Ar*), 7.19 (1H, ddd, *J* = 8.3, 7.3, 1.2 Hz, *Ar*), 4.14 (1H, dddd, *J* = 10.3, 6.6, 5.7, 2.6 Hz, C(7)*H*), 3.93 (1H, dd, *J* = 11.3, 2.6 Hz, C(8)*H*H), 3.73 (1H, dd, *J* = 11.3, 2.2 Hz, C(9)*H*H), 3.50-3.42 (1H, m, C(10)*H*), 3.37 (1H, dd, *J* = 11.2, 10.2 Hz, C(9)H*H*), 3.36 (1H, dd, *J* = 18.5, 11.3 Hz, C(8)H*H*), 3.20 (1H, dd, *J* = 16.2, 6.7 Hz, C(6)*H*H), 2.83 (1H, dd, *J* = 16.2, 5.8 Hz, C(6)H*H*), 2.43 (3H, s, SC(1)H_3), 1.46-1.21 (6H, m, C(11, 12 and 13)H_2), 0.98-0.78 (3H, m, C(14)H_3); **δ_C** (101 MHz, $CDCl_3$) (major signals only) 198.3, 142.7, 134.7, 132.4, 130.6, 125.3, 123.7, 75.1, 71.9, 71.7, 71.1, 42.3, 31.3, 27.4, 22.9, 16.1, 14.1; **ν_{max}**/ cm^{-1} (neat) 3019, 2929, 2856, 2360, 1671, 1587, 1558, 1463, 1433, 1354, 1274,

1197, 1136, 1103, 1045, 982, 925, 850, 752, 697, 665, 648; **m/z HRMS** (ESI^+) $[M + H]^+$ $C_{17}H_{25}O_3{}^{32}S^+$ calc. 309.1524, found 309.1515.

1-(2-(Methylthio)phenyl)-2-((2*R,5*S**)-5-phenyl-1,4-dioxan-2-yl)ethan-1-one (5q)**

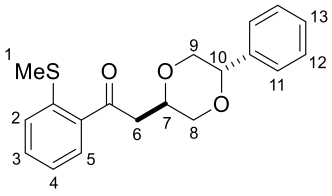

Following **general procedures H** and **I**. **General procedure H** used 2-(methylthio)benzaldehyde **1a** (40 μL, 0.300 mmol, 1.00 equiv.) and 2-phenyl-2-(prop-2-yn-1-yloxy)ethan-1-ol **2g** (63 mg, 0.360 mmol, 1.20 equiv.) with $Rh(nbd)_2BF_4$ (5.6 mg, 0.015 mmol, 0.050 equiv.), dcpe (6.3 mg, 0.015 mmol, 0.050 equiv.) and DCE (0.3 mL). **General procedure I** used HCl in dioxane (4 M in dioxane, 0.15 mL, 0.600 mmol, 2.00 equiv.) and DCE (4.0 mL). Purification of the crude residue (11:1 dr) by column chromatography (1:19 to 1:5 EtOAc:Petrol) yielded the title compound as an inseparable mixture of diastereoisomers **5q** (83.5 mg, 0.255 mmol, 85%) as an off-white powdered solid.

m.p. (CH_2Cl_2) 104–106 °C; δ_H (400 MHz, $CDCl_3$) (major signals only) 7.86 (1H, dd, *J* = 7.8, 1.5 Hz, *Ar*), 7.49 (1H, ddd, *J* = 8.5, 7.2, 1.5 Hz, *Ar*), 7.43-7.27 (6H, m, *Ar*), 7.21 (1H, ddd, *J* = 8.2, 7.6, 1.2 Hz, *Ar*), 4.58 (1H, dd, *J* = 10.4, 2.8 Hz, C(10)*H*), 4.32 (1H, m, C(7)*H*), 4.15 (1H, dd, *J* = 11.6, 2.6 Hz, C(8)*H*H), 3.88 (1H, dd, *J* = 11.7, 2.7 Hz, C(9)*H*H), 3.61 (1H, dd, *J* = 11.5, 10.4 Hz, C(8)H*H*), 3.57 (1H, dd, *J* = 11.7, 10.4 Hz, C(9)H*H*), 3.30 (1H, dd, *J* = 16.5, 6.4 Hz, C(6)*H*H), 2.93 (1H, dd, *J* = 16.4, 6.1 Hz, C(6)H*H*), 2.44 (3H, s, C(1)H_3); δ_C (101 MHz, $CDCl_3$) (major signals only) 198.1, 142.8, 138.1, 134.5, 132.6, 130.6, 128.6, 128.3, 126.4, 125.4, 123.7, 77.7, 72.8, 71.8, 71.3, 42.2, 16.2; ν_{max}/ cm^{-1} (neat) 2958, 2898, 2860, 1671, 1433, 1104, 753, 699; **m/z HRMS** (ESI^+) $[M + H]^+$ $C_{19}H_{21}O_3{}^{32}S^+$ calc. 329.1206, found 329.1204.

1-(2-(Methylthio)phenyl)-2-((2*R,5*S**)-5-(naphthalen-2-yl)-1,4-dioxan-2-yl)ethan-1-one (5r)**

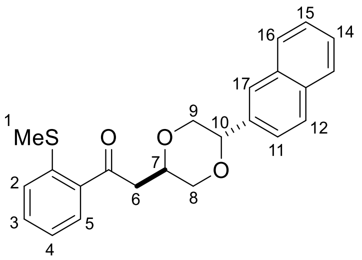

Following **general procedures H** and **I**. **General procedure H** used 2-(methylthio)benzaldehyde **1a** (40 μL, 0.300 mmol, 1.00 equiv.) and 2-(naphthalen-2-yl)-2-(prop-2-yn-1-yloxy)ethan-1-ol **2h** (81 mg, 0.360 mmol, 1.20 equiv.) with $Rh(nbd)_2BF_4$ (5.60 mg, 0.015 mmol, 0.050 equiv.), dcpe (6.30 mg, 0.015 mmol, 0.050 equiv.) and DCE (0.3 mL). **General procedure I** used HCl in dioxane (4 M in dioxane, 0.15 mL, 0.600 mmol, 2.00 equiv.) and DCE (4.0 mL). Purification of the crude residue (6:1 dr) by column chromatography (1:12 to 1:5 EtOAc:Petrol) yielded the title compound as an inseparable mixture of diastereoisomers **5r** (85.0 mg, 0.225 mmol, 75%) as an off-white powdered solid.

m.p. (CH_2Cl_2) 92–93 °C; **δ_H** (400 MHz, $CDCl_3$) (major signals only) 7.90-7.79 (5H, m, *Ar*), 7.53-7.42 (4H, m, *Ar*), 7.38-7.32 (1H, m, *Ar*), 7.22 (1H, ddd, *J* = 7.8, 7.2, 1.2 Hz, *Ar*), 4.75 (1H, dd, *J* = 10.4, 2.8 Hz, C(10)*H*), 4.37 (1H, m, C(7)*H*), 4.21 (1H, dd, *J* = 11.5, 2.6 Hz, C(8)*H*H), 3.98 (1H, dd, *J* = 11.7, 2.8 Hz, C(9)*H*H), 3.68 (1H, dd, *J* = 11.2, 9.1 Hz, C(8)H*H*), 3.65 (1H, dd, *J* = 11.7, 10.4 Hz, C(9)H*H*), 3.32 (1H, dd, *J* = 16.4, 6.4 Hz, C(6)*H*H), 2.96 (1H, dd, *J* = 16.4, 6.1 Hz, C(6)H*H*), 2.45 (3H, s, SC(1)H_3); **δ_C** (101 MHz, $CDCl_3$) (major signals only) 198.1, 142.8, 135.6, 134.5, 133.3, 133.2, 132.5, 130.6, 128.3, 128.1, 127.8, 126.3, 126.1, 125.4, 125.3, 124.2, 123.7, 77.7, 72.8, 71.8, 71.3, 42.2, 16.1; **ν_{max}**/ cm^{-1} (neat) 2851, 1669, 1587, 1556, 1463, 1433, 1357, 1341, 1274, 1198, 1102, 820, 748; **m/z HRMS** (ESI^+) $[M + H]^+$ $C_{23}H_{23}O_3{}^{32}S^+$ calc. 379.1362, found 379.1364.

1-(2-(Methylthio)phenyl)-2-((2*R,5*S**)-5-(4-(trifluoromethyl)phenyl)-1,4-dioxan-2-yl)ethan-1-one (5s)**

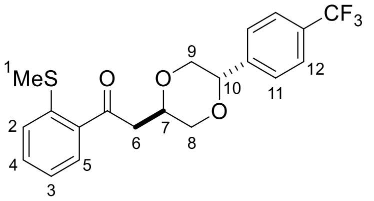

Following **general procedures H** and **I**. **General procedure H** used 2-(methylthio)benzaldehyde **1a** (40 μL, 0.300 mmol, 1.00 equiv.) and 2-(prop-2-yn-1-yloxy)-2-(4-(trifluoromethyl)phenyl)ethan-1-ol **2i** (88 mg, 0.360 mmol, 1.20 equiv.) with Rh(nbd)$_2$BF$_4$ (5.6 mg, 0.015 mmol, 0.050 equiv.), dcpe (6.3 mg, 0.015 mmol, 0.050 equiv.) and DCE (0.3 mL). **General procedure I** used HCl in dioxane (4 M in dioxane, 0.15 mL, 0.600 mmol, 2.00 equiv.) and DCE (4.0 mL). Purification of the crude residue (7:1 dr) by column chromatography (1:9 to 1:4 EtOAc:Petrol) yielded the title compound as an inseparable mixture of diastereoisomers **5s** (91.9 mg, 0.232 mmol, 77%) as an off-white powdered solid.

m.p. (CH_2Cl_2) 126–127 °C; **δ_F** (377 MHz, $CDCl_3$) (major signals only) -62.59 (C*F₃*); **δ_H** (400 MHz, $CDCl_3$) (major signals only) 7.88-7.82 (1H, m, *Ar*), 7.64-7.57 (2H, m, *Ar*), 7.55-7.44 (3H, m, *Ar*), 7.34 (1H, dd, *J* = 8.2, 1.1 Hz, *Ar*), 7.21 (1H, ddd, *J* = 7.8, 7.2 1.1 Hz, *Ar*), 4.63 (1H, dd, *J* = 10.4, 2.8 Hz, C(10)*H*), 4.35-4.26 (1H, m, C(7)*H*), 4.17 (1H, dd, *J* = 11.5, 2.6 Hz, C(8)*H*H), 3.89 (1H, dd, *J* = 11.8, 2.9 Hz, C(9)*H*H), 3.61 (1H, dd, *J* = 11.5, 10.3 Hz, C(8)H*H*), 3.50 (1H, dd, *J* = 11.7, 10.3 Hz, C(9)H*H*), 3.29 (1H, dd, *J* = 16.6, 6.3 Hz, C(6)*H*H), 2.94 (1H, dd, *J* = 16.5, 6.1 Hz, C(6)H*H*), 2.44 (3H, s, C(1)H_3); **δ_C** (101 MHz, $CDCl_3$) (major signals only) 197.9, 142.8, 142.1, 134.4, 132.6, 130.6, 130.3 (q, J_{CF} = 32.2 Hz), 126.6, 125.5 (q, J_{CF} = 3.7 Hz), 125.3, 124.2 (q, J_{CF} = 272.0 Hz), 123.7, 76.8, 72.6, 71.8, 71.2, 42.1, 16.1; **ν_{max}**/ cm^{-1} (neat) 2891, 1670, 1621, 1588, 1558, 1463, 1434, 1323, 1271, 1163, 1109, 1067, 1018, 981, 926, 908, 839, 751, 697, 680, 652, 609; **m/z HRMS** (ESI^+) $[M + H]^+$, $C_{20}H_{20}O_3F_3{}^{32}S^+$ calc. 397.1091, found 397.1079.

2-((2*R,6*S**)-6-Benzyl-1,4-dioxan-2-yl)-1-(2-(methylthio)phenyl)ethan-1-one (5t)**

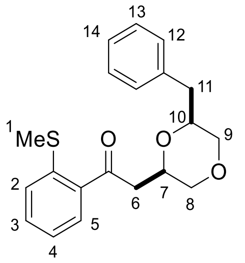

Following **general procedures H** and **I**. **General procedure H** used 2-(methylthio)benzaldehyde **1a** (40 μL, 0.300 mmol, 1.00 equiv.) and 1-phenyl-3-(prop-2-yn-1-yloxy)propan-2-ol **2j** (69 mg, 0.360 mmol, 1.20 equiv.) with $Rh(nbd)_2BF_4$ (5.6 mg, 0.015 mmol, 0.050 equiv.), dcpe (6.3 mg, 0.015 mmol, 0.050 equiv.) and DCE (0.3 mL). **General procedure I** used HCl in dioxane (4 M in dioxane, 0.15 mL, 0.600 mmol, 2.00 equiv.) and DCE (4.0 mL). Purification of the crude residue (11:1 dr) by column chromatography (1:9 to 1:4 EtOAc:Petrol) yielded the title compound as an inseparable mixture of diastereoisomers **5t** (80.1 mg, 0.234 mmol, 78%) as a viscous yellow oil.

δ_H (400 MHz, $CDCl_3$) (major signals only) 7.81-7.76 (1H, m, *Ar*), 7.46 (1H, ddd, *J* = 8.5, 7.2, 1.5 Hz, *Ar*), 7.31 (1H, dd, *J* = 8.2, 1.1 Hz, *Ar*), 7.28-7.11 (6H, m, *Ar*), 4.26-4.18 (1H, m, C(7)*H*), 3.90 (1H, dd, *J* = 11.1, 2.5 Hz, C(8)*H*H), 3.88-3.82 (1H, m, C(10)*H*), 3.68 (1H, dd, *J* = 11.5, 2.6 Hz, C(9)*H*H), 3.26 (1H, dd, *J* = 11.3, 10.3 Hz, C(8)H*H*), 3.23 (1H, dd, *J* = 11.6, 10.7 Hz, C(9)H*H*), 3.22 (1H, dd, *J* = 16.2, 6.1 Hz, C(6)*H*H), 2.88 (1H, dd, *J* = 16.2, 6.3 Hz, C(6)H*H*), 2.78 (1H, dd, *J* = 14.0, 6.8 Hz, C(11)*H*H), 2.56 (1H, dd, *J* = 14.0, 6.5 Hz, C(11)H*H*), 2.42 (3H, s, SC(1)H_3); **δ_C** (101 MHz, $CDCl_3$) (major signals only) 198.4, 142.5, 137.4, 134.7, 132.4, 130.7, 129.3, 128.4, 126.5, 125.3, 123.6, 76.3, 72.5, 70.5, 70.4, 42.5, 38.4, 16.1; **ν_{max}**/ cm^{-1} (neat) 3028, 2917, 2851, 1670, 1433, 1119, 753, 701; **m/z HRMS** (ESI^+) $[M + H]^+$ $C_{20}H_{23}O_3{}^{32}S^+$ calc. 343.1362, found 343.1358.

2-((2*R,6*S**)-6-Benzyl-1,4-dioxan-2-yl)-1-(4-bromo-2-(methylthio)phenyl)ethan-1-one (5u)**

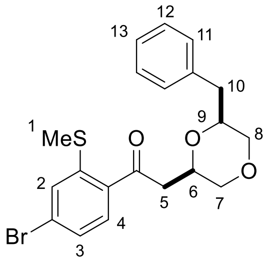

Following **general procedures H** and **I**. **General procedure H** used 4-bromo-2-(methylthio)benzaldehyde **1c** (46 mg, 0.200 mmol, 1.00 equiv.) and 1-phenyl-3-(prop-2-yn-1-yloxy)propan-2-ol **2j** (46 mg, 0.240 mmol, 1.20 equiv.) with $Rh(nbd)_2BF_4$ (3.7 mg, 0.010 mmol, 0.050 equiv.), dcpe (4.2 mg, 0.01 mmol, 0.050 equiv.) and DCE (0.2 mL). **General procedure I** used HCl in dioxane (4 M in dioxane, 0.100 mL, 0.400 mmol, 2.00 equiv.) and DCE (2.7 mL). Purification of the crude residue (3.5:1 dr) by column chromatography (1:9 to 1:5 EtOAc:Petrol) yielded the title compound as an inseparable mixture of diastereoisomers **5u** (59.5 mg, 0.142 mmol, 71%) as a yellow viscous oil.

δ_H (400 MHz, $CDCl_3$) (major signals only) 7.64 (1H, d, *J* = 8.3 Hz, *Ar*), 7.41 (1H, d, *J* = 1.7 Hz, *Ar*), 7.31-7.16 (5H m, *Ar*), 7.13 (1H, app d, *J* = 7.6 Hz, *Ar*), 4.23-4.13 (1H, m, C(6)*H*), 3.83-3.74 (2H, m, C(7)*H*H and C(9)*H*), 3.61 (1H, dd, *J* = 11.5, 2.5 Hz, C(8)*H*H), 3.23-3.12 (2H, m, C(7)H*H* and C(8)H*H*), 3.16 (1H, dd, *J* = 16.1, 6.5 Hz, C(5)*H*H), 2.74 (1H, dd, *J* = 16.0, 6.1 Hz, C(5)H*H*), 2.76 (1H, dd, *J* = 14.0, 7.0 Hz, C(10)*H*H), 2.63-2.52 (1H, dd, *J* = 14.1, 6.2 Hz, C(10)H*H*), 2.42 (3H, s, C(1)H_3); **δ_C** (101 MHz, $CDCl_3$) (major signals only) 197.7, 145.1, 137.3, 133.1, 132.0, 129.3, 128.4, 127.9, 127.8, 126.6, 126.5, 76.4, 72.5, 70.4, 70.4, 42.4, 38.4, 16.2; **ν_{max}**/ cm^{-1} (neat) 2981, 1723, 1670, 1609, 1521, 1347, 1237, 1091, 733, 670; **m/z HRMS** (ESI^+) $[M + H]^+$ $C_{20}H_{22}O_3{}^{79}Br^{32}S^+$ calc. 421.0468, found 421.0468, $C_{20}H_{22}O_3{}^{81}Br^{32}S^+$ calc. 423.0447, found 423.0446.

2-((2*R,5*S**,6*S**)-5,6-Dimethyl-1,4-dioxan-2-yl)-1-(2-(methylthio)phenyl)ethan-1-one (5v)**

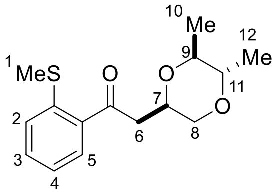

Following **general procedures H** and **I**. **General procedure H** used 2-(methylthio)benzaldehyde **1a** (40 μL, 0.300 mmol, 1.00 equiv.) and (2*S**,3*S**)-3-(prop-2-yn-1-yloxy)butan-2-ol **2k** (46 mg, 0.360 mmol, 1.20 equiv.) with $Rh(nbd)_2BF_4$ (5.6 mg, 0.015 mmol, 0.050 equiv.), dcpe (6.3 mg, 0.015 mmol, 0.050 equiv.) and DCE (0.3 mL). **General procedure I** used HCl in dioxane (4 M in dioxane, 0.15 mL, 0.600 mmol, 2.00 equiv.) and DCE (4.0 mL). Purification of the crude residue (16:1 dr) by column chromatography (1:12 to 1:5 EtOAc:Petrol) yielded the title compound as an inseparable mixture of diastereoisomers **5v** (66.2 mg, 0.236 mmol, 79%) as an off-white solid.

m.p. (CH_2Cl_2) 52–53 °C; **δ_H** (400 MHz, $CDCl_3$) (major signals only) 7.81 (1H, ddd, *J* = 11.7, 7.9, 1.4 Hz, *Ar*), 7.49-7.41 (1H, m, *Ar*), 7.31 (1H, app d, *J* = 8.0 Hz, *Ar*), 7.17 (1H, app td, *J* = 7.9, 1.1 Hz, *Ar*), 4.21 (1H, app dtd, *J* = 10.2, 6.3, 2.6 Hz, C(7)*H*), 3.92 (1H, dd, *J* = 11.2, 2.6 Hz, C(8)*H*H), 3.42-3.28 (2H, m, C(8)H*H* and C(9)*H*), 3.22 (1H, dd, *J* = 16.3, 6.3 Hz, C(6)*H*H), 3.19-3.14 (1H, m, C(11)*H*), 2.83 (1H, dd, *J* = 16.3, 6.5 Hz, C(6)H*H*), 2.41 (3H, s, C(1)H_3), 1.09 (3H, d, *J* = 6.3 Hz, C(12)H_3), 1.07 (3H, d, *J* = 6.3 Hz, C(10)H_3); **δ_C** (101 MHz, $CDCl_3$) (major signals only) 198.3, 142.5, 134.7, 132.4, 130.5, 125.3, 123.6, 77.4, 76.7, 72.0, 71.1, 42.6, 17.5, 17.3, 16.1; **ν_{max}**/ cm^{-1} (neat) 2975, 2851, 1670, 1587, 1558, 1433, 1381, 1360, 1278, 1201, 1108, 944, 753; **m/z HRMS** (ESI^+) $[M + H]^+$ $C_{15}H_{21}O_3{}^{32}S^+$ calc. 281.1206, found 281.1207.

1-(2-(Methylthio)phenyl)-2-((7*R*,9*S*,10*S*)-(octahydrobenzo[b][1,4]dioxin-2-yl)ethan-1-one (5w)

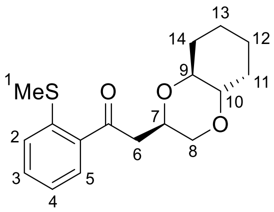

Following **general procedures H** and **I**. **General procedure H** used 2-(methylthio)benzaldehyde **1a** (40 μL, 0.300 mmol, 1.00 equiv.) and (1*S*,2*S*)-2-(prop-2-yn-1-yloxy)cyclohexan-1-ol **2l** (56 mg, 0.360 mmol, 1.20 equiv.) with Rh(nbd)$_2$BF$_4$ (5.6 mg, 0.015 mmol, 0.050 equiv.), dcpe (6.3 mg, 0.015 mmol, 0.050 equiv.) and DCE (0.3 mL). **General procedure I** used HCl in dioxane (4 M in dioxane, 0.15 mL, 0.600 mmol, 2.00 equiv.) and DCE (4.0 mL). Purification of the crude residue (12:1 dr) by column chromatography (1:9 to 1:5 EtOAc:Petrol) yielded the title compound as a single diastereoisomer **5w** (78.4 mg, 0.256 mmol, 85%, >99% ee) as an off-white solid.

m.p. (CH_2Cl_2) 91–95 °C; **δ_H** (400 MHz, $CDCl_3$) 7.82 (1H, dd, *J* = 7.8, 1.5 Hz, *Ar*), 7.49-7.43 (1H, m, *Ar*), 7.32 (1H, dd, *J* = 8.2, 1.1 Hz, *Ar*), 7.18 (1H, ddd, *J* = 8.2, 7.3, 1.2 Hz, *Ar*), 4.29 (1H, dddd, *J* = 10.3, 6.6, 6.1 2.6 Hz, C(7)*H*), 3.97 (1H, dd, *J* = 11.3, 2.6 Hz, C(8)H*H*), 3.42 (1H, dd, *J* = 11.3, 10.3 Hz, C(8)H*H*), 3.26 (1H, dd, *J* = 16.6, 6.1 Hz, C(6)*H*H), 3.30-3.21 (1H, m, C(9)*H*), 3.14-3.05 (1H, m, C(10)*H*), 2.86 (1H, dd, *J* = 16.5, 6.6 Hz, C(6)H*H*), 2.42 (3H, s, SC(1)H_3), 1.94-1.79 (2H, m, C(11 and 14)*H*H), 1.76-1.64 (2H, m, C(12 and 13)*H*H), 1.38-1.18 (4H, m, C(11 and 14)H*H* and C(12 and 13)H*H*); **δ_C** (101 MHz, $CDCl_3$) 198.2, 142.7, 134.6, 132.4, 130.6, 125.3, 123.6, 80.1, 79.6, 72.3, 71.5, 42.6, 30.4, 30.2, 24.4, 24.3, 16.1; **ν_{max}**/ cm^{-1} (neat) 2935, 2862, 1671, 1587, 1558, 1433, 1355, 1272, 1211, 1099, 753, 644; **m/z HRMS** (ESI^+) $[M + Na]^+$ $C_{17}H_{22}O_3{}^{23}Na^{32}S^+$ calc. 329.1182, found 329.1183; $[\alpha]_D^{25}$

+16.0° (c 0.25, $CHCl_3$); **Chiral HPLC** Daicel Chiralpack OJ-H, 10% IPA, 90% hexane, 1 mL/min, 25 ˚C, λ = 225 nm, 10 μL injection, >99% *ee*.

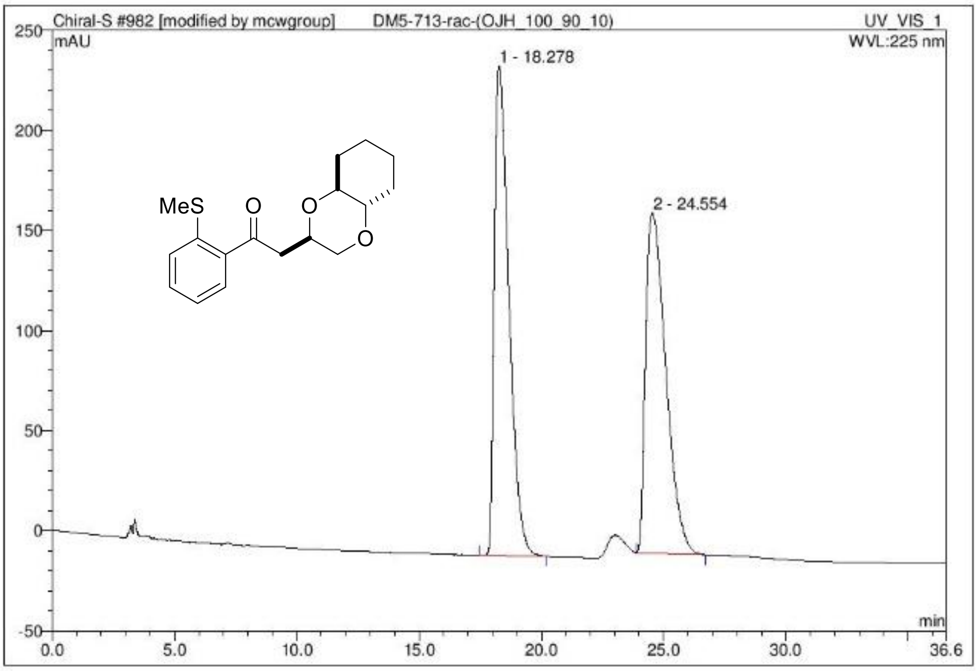

No.	Ret.Time min	Peak Name	Height mAU	Area mAU*min	Rel.Area %	Amount	Type
1	18.28	n.a.	244.810	169.776	50.96	n.a.	BMB*
2	24.55	n.a.	170.374	163.367	49.04	n.a.	BMB*
Total:			415.184	333.143	100.00	0.000	

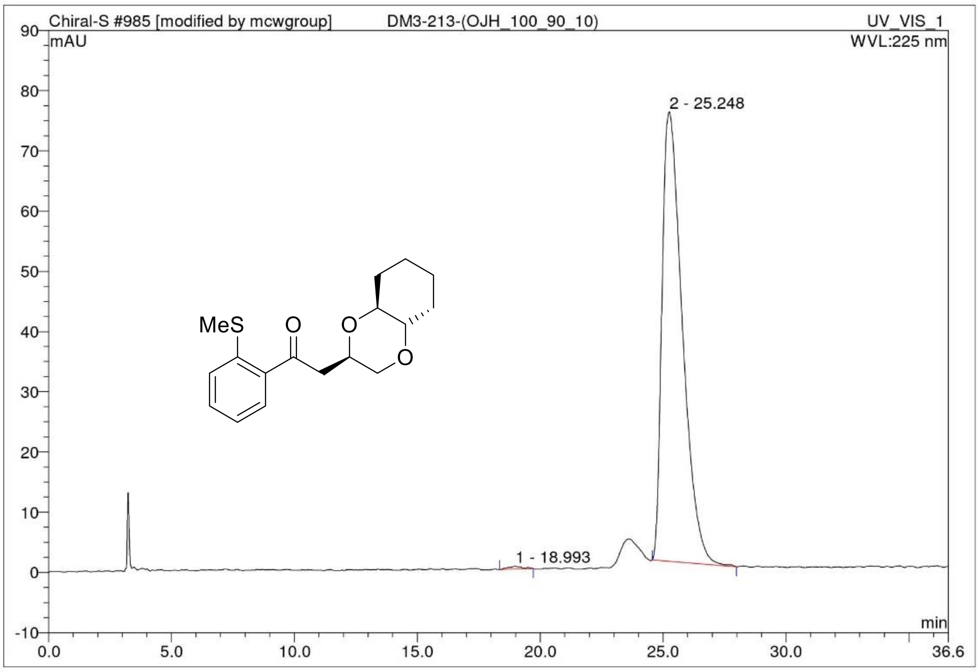

No.	Ret.Time min	Peak Name	Height mAU	Area mAU*min	Rel.Area %	Amount	Type
1	18.99	n.a.	0.442	0.325	0.45	n.a.	BMB*
2	25.25	n.a.	74.641	71.578	99.55	n.a.	BMB*
Total:			75.083	71.904	100.00	0.000	

2-((2*R,5*R**)-5-Isobutyl-4-(methylsulfonyl)morpholin-2-yl)-1-(2-(methylthio)phenyl)ethan-1-one (5x) and 2-((2*R**,5*S**)-5-isobutyl-4-(methylsulfonyl)morpholin-2-yl)-1-(2-(methylthio)phenyl)ethan-1-one (5x')**

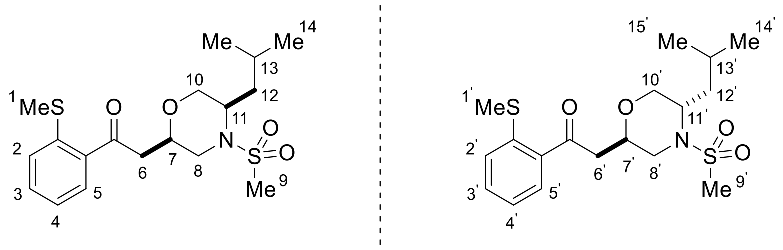

5x **5x'**

Following **general procedures H** and **I**. **General procedure H** used 2-(methylthio)benzaldehyde **1a** (40 µL, 0.300 mmol, 1.00 equiv.) and *N*-(1-hydroxy-4-methylpentan-2-yl)-*N*-(prop-2-yn-1-yl)methanesulfonamide **2m** (84 mg, 0.360 mmol, 1.20 equiv.) with $Rh(nbd)_2BF_4$ (5.6 mg, 0.015 mmol, 0.050 equiv.), dcpe (6.3 mg, 0.015 mmol, 0.050 equiv.) and DCE (0.3 mL). **General procedure I** used HCl in dioxane (4 M in dioxane, 0.15 mL, 0.600 mmol, 2.00 equiv.) and DCE (4.0 mL). Purification of the crude residue (2.8:1 dr) by column chromatography (CH_2Cl_2) yielded major diastereoisomer **5x** (55.9 mg, 0.145 mmol, 48%), and minor diastereoisomer **5x'** (15.7 mg, 0.041 mmol, 14%) as a colourless viscous oil and a white solid, respectively.

5x: δ_H (400 MHz, $CDCl_3$) 7.79 (1H, dd, *J* = 7.9, 1.5 Hz, *Ar*), 7.47 (1H, ddd, *J* = 8.6, 7.2, 1.5 Hz, *Ar*), 7.33 (1H, dd, *J* = 8.2, 1.1 Hz, *Ar*), 7.19 (1H, ddd, *J* = 8.3, 7.6, 1.1 Hz, *Ar*), 4.12-4.04 (1H, m, C(7)*H*), 3.84-3.77 (1H, m, C(11)*H*), 3.76 (1H, dd, *J* = 11.9, 1.1 Hz, C(10)*H*H), 3.71 (1H, dd, *J* = 11.7, 2.9 Hz, C(10)H*H*), 3.68 (1H, app dd, *J* = 13.6, 2.8 Hz, C(8)*H*H), 3.28 (1H, dd, *J* = 16.7, 6.2 Hz, C(6)*H*H), 3.04 (1H, dd, *J* = 13.7, 11.1 Hz, C(8)H*H*), 2.97 (1H, dd, *J* = 16.7, 6.0 Hz, C(6)H*H*), 2.94 (3H, s, C(9)H_3), 2.43 (3H, s, C(1)H_3), 1.66-1.57 (3H, m, C(12)H_2 and C(13)*H*), 0.98-0.91 (6H, m, C(14)H_3); δ_C (101 MHz, $CDCl_3$) 197.6, 142.8, 134.4, 132.6, 130.5, 125.4, 123.7, 72.4, 69.7, 51.0, 44.9, 43.5, 40.9, 37.8, 25.0, 22.8, 22.5, 16.1; ν_{max}/ cm^{-1}

(neat) 1669, 1587, 1558, 1464, 1433, 1327, 1278, 1205, 1149, 1100, 1073, 1045, 969, 912, 853, 776, 732, 694, 650; **m/z HRMS** (ESI$^+$) [M + Na]$^+$ $C_{18}H_{27}O_4N^{23}Na^{32}S_2^+$ calc. 408.1274, found 408.1275.

5x': **m.p.** (CH_2Cl_2) 115–118 °C; **δ_H** (400 MHz, $CDCl_3$) 7.87 (1H, app dd, *J* = 7.9, 1.5 Hz, *Ar*), 7.49 (1H, app ddd, *J* = 8.5, 7.3, 1.5 Hz, *Ar*), 7.34 (1H, dd, *J* = 7.7 Hz, 1H), 7.24 (1H, app td, *J* = 7.5, 1.2 Hz, *Ar*), 4.21-4.10 (2H, m, C(7')*H* and C(8')*H*), 4.09-4.00 (1H, m, C(11')*H*), 3.99-3.88 (2H, m, C(10' and 8')H*H*), 3.82 (1H, dd, *J* = 15.5, 3.8 Hz, C(6')*H*H), 3.61-3.52 (2H, m, C(6' and 10')H*H*), 2.96 (3H, s, C(14' or 15')*H*$_3$), 2.43 (3H, s, C(14' or 15')*H*$_3$), 1.64-1.50 (2H, m, C(12')*H*H and C(13')*H*), 1.43-1.35 (1H, m, C(12')H*H*), 0.99-0.92 (6H, m, C(14')*H*$_3$); **δ_C** (101 MHz, $CDCl_3$) 200.4, 143.0, 134.0, 132.8, 130.5, 125.7, 124.1, 76.8, 74.2, 56.0, 50.5, 43.2, 40.7, 39.7, 24.7, 22.8, 22.8, 16.3; **ν_{max}**/ cm^{-1} (neat) 2953, 2924, 1663, 1587, 1558, 1463, 1434, 1325, 1208, 1099, 1049, 972, 771, 742; **m/z HRMS** (ESI$^+$) [M + H]$^+$ $C_{18}H_{28}O_4N^{32}S_2^+$ calc. 386.1454, found 386.1456.

NOESY spectrum of 5x:

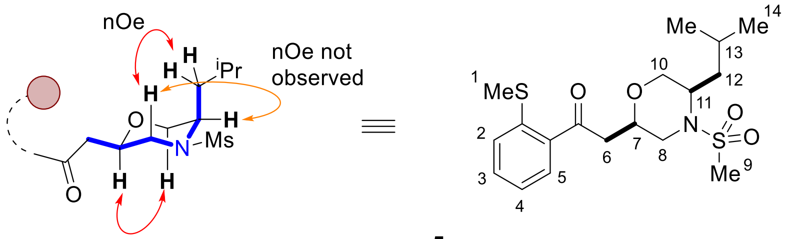

5x

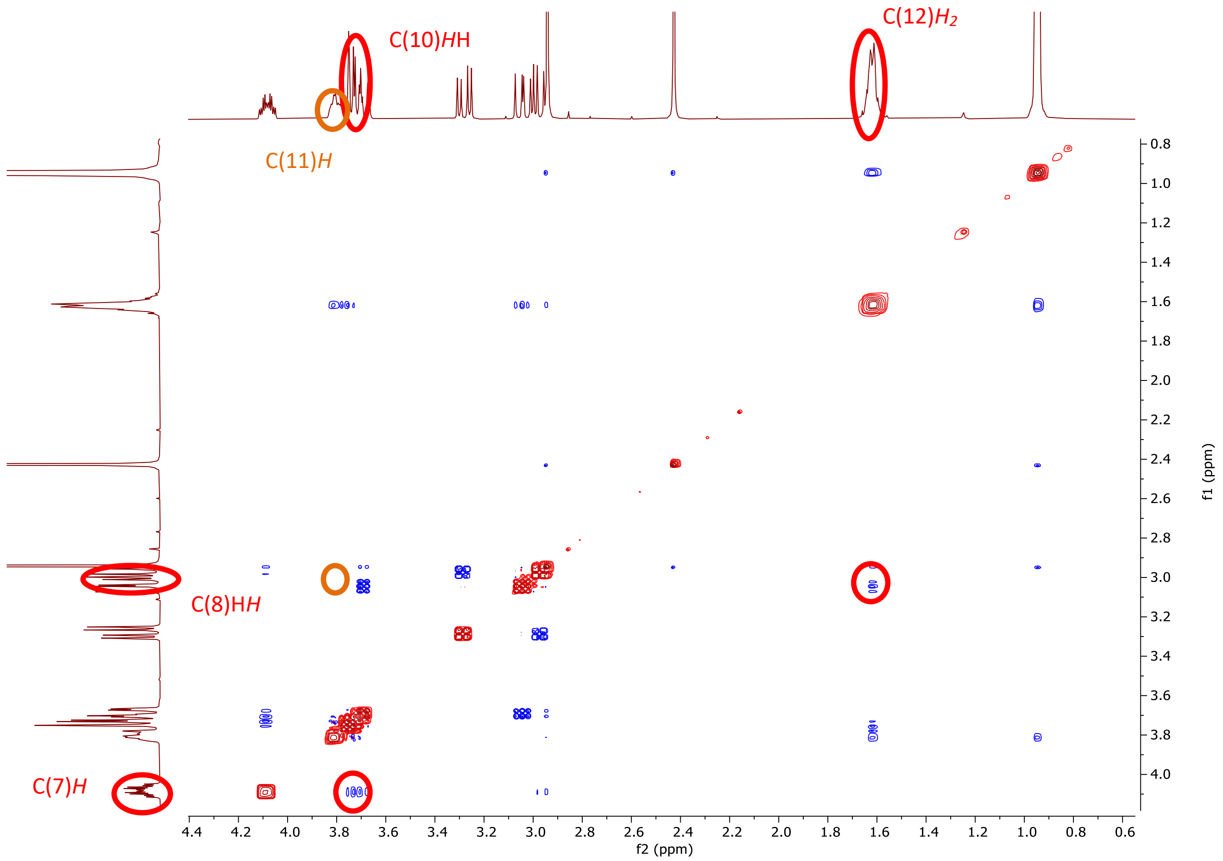

From the NOESY spectrum of (2*R**,*R**)-**5x** (above), we believe that two key NOESY interactions occur, firstly between C(10)*H*H (3.76 ppm) and C(7)*H* (4.04 ppm) environments, and also between C(8)H*H* (3.04 ppm) and C(12)H_2 (1.66–1.57 ppm) environments. Alongside the absence of a NOESY interaction between C(8)*H*H and C(11)*H*, this suggests a *syn* relative configuration is present. For the NOESY spectrum of minor *anti* diastereomer (2*R**,5*S**)-**5x**, neither of the C(7')*H* - C(10')*H*H or C(8')H*H* - C(12')H_2 interactions were clearly observable (shown below).

Absence of the C(7')*H* - C(10')*H*H interaction in 5x':

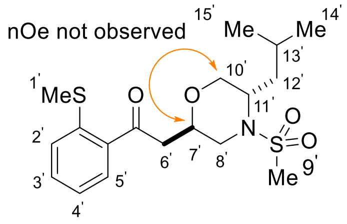

5x'

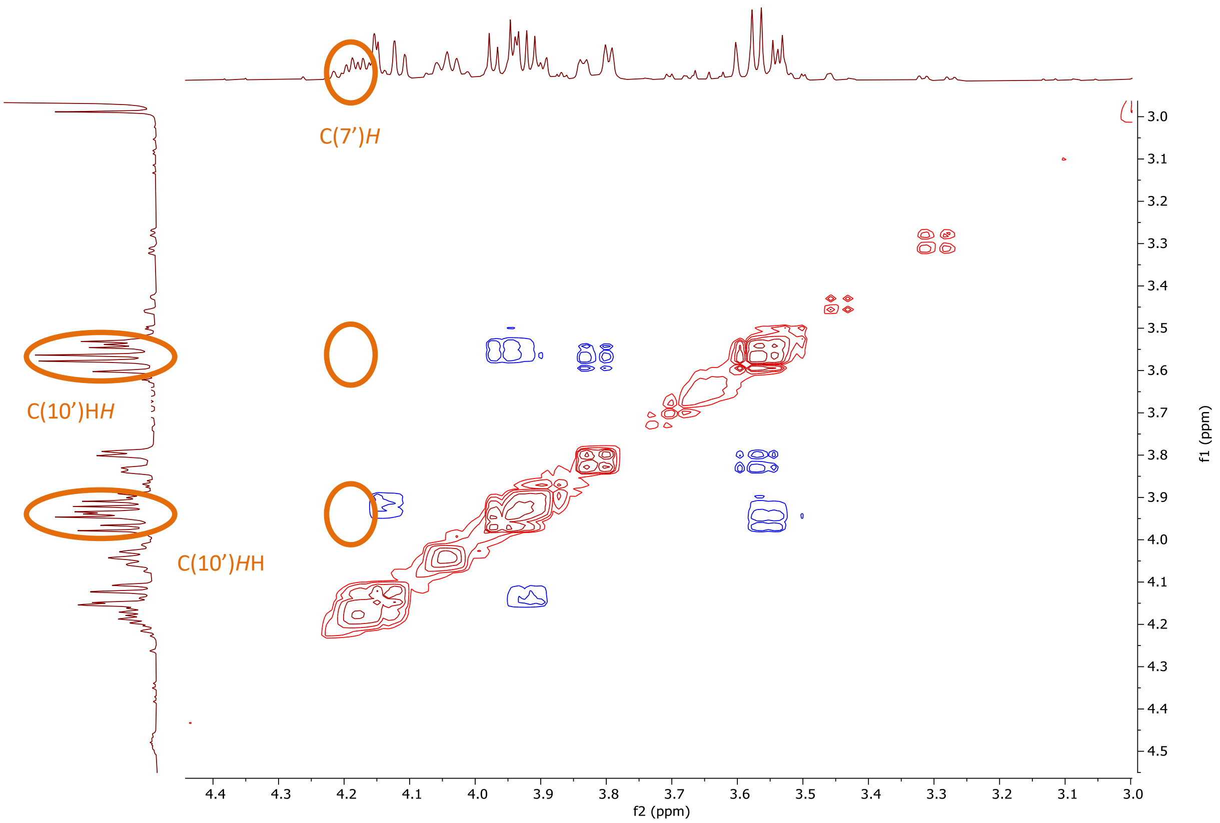

Absence of the C(8')*H*H - C(12')*H* interaction in 5x':

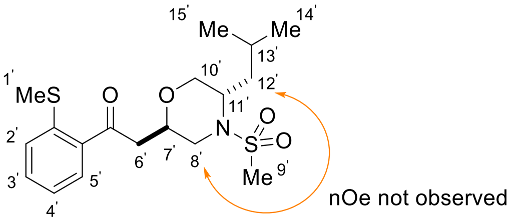

5x'

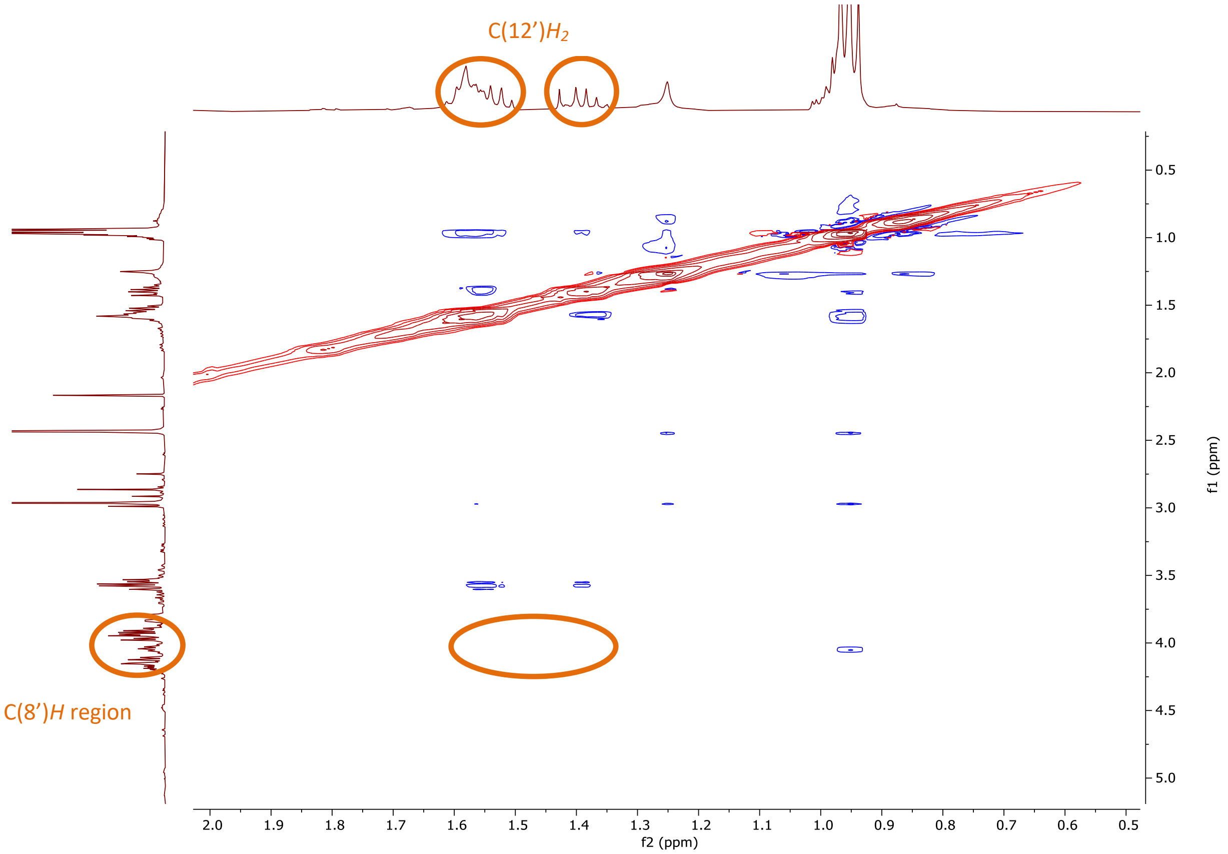

(3*R,8a*S**)-3-(2-(2-(Methylthio)phenyl)-2-oxoethyl)hexahydro-6H-pyrrolo[2,1-c][1,4]oxazin-6-one (5y)**

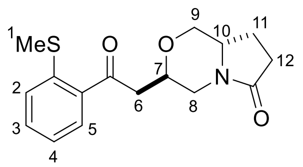

Following **general procedures H** and **I**. **General procedure H** used 2-(methylthio)benzaldehyde **1a** (40 μL, 0.300 mmol, 1.00 equiv.) and 5-(hydroxymethyl)-1-(prop-2-yn-1-yl)pyrrolidin-2-one **2n** (55 mg, 0.360 mmol, 1.20 equiv.) with $Rh(nbd)_2BF_4$ (5.6 mg, 0.015 mmol, 0.050 equiv.), dcpe (6.3 mg, 0.015 mmol, 0.050 equiv.) and DCE (0.3 mL). **General procedure I** used HCl in dioxane (4 M in dioxane, 0.15 mL, 0.600 mmol, 2.00 equiv.) and DCE (4.0 mL). Purification of the crude residue (>20:1 dr) by column chromatography (1:0:99 to 1:5:94 IPA:MeOH:CH_2Cl_2) yielded the title compound as a single diastereoisomer **5y** (58.1 mg, 0.190 mmol, 63%) as an off-white solid.

m.p. (CH_2Cl_2) 74–77 °C; **δ_H** (400 MHz, $CDCl_3$) 7.78 (1H, dd, *J* = 8.0, 1.5 Hz, *Ar*), 7.45 (1H, ddd, *J* = 8.2, 7.3, 1.5 Hz, *Ar*), 7.31 (1H, dd, *J* = 8.2, 1.1 Hz, *Ar*), 7.17 (1H, ddd, *J* = 8.2, 7.3, 1.2 Hz, *Ar*), 4.10 (1H, dd, *J* = 13.0, 2.9 Hz, C(8)*H*H), 4.00-3.95 (1H, dd, *J* = 11.1, 3.5 Hz, C(9)*H*H), 3.98-3.89 (1H, m, C(7)*H*), 3.68-3.53 (1H, m, C(10)*H*), 3.27 (1H, dd, *J* = 16.3, 7.5 Hz, C(6)*H*H), 3.22-3.12 (1H, app t, *J* = 11.0 Hz, C(9)H*H*), 2.93 (1H, dd, *J* = 16.3, 4.6 Hz, C(6)H*H*), 2.74-2.66 (1H, m, C(8)H*H*), 2.42-2.36 (2H, m, C(12)H_2), 2.41 (3H, s, SC(1)H_3), 2.15-2.05 (1H, m, C(11)*H*H), 1.57-1.46 (1H, m, C(11)H*H*); **δ_C** (101 MHz, $CDCl_3$) 197.5, 173.2, 142.7, 134.5, 132.4, 130.4, 125.4, 123.6, 72.8, 72.1, 54.5, 44.7, 43.3, 30.3, 20.6, 16.1; **ν_{max}**/ cm^{-1} (neat) 2917, 1668, 1587, 1558, 1433, 1353, 1302, 1267, 1190, 1102, 979, 915, 727, 650; **m/z HRMS** (ESI^+) $[M + H]^+$ $C_{16}H_{20}O_3N^{32}S^+$ calc. 306.1158, found 306.1158.

NOESY spectrum of 5y:

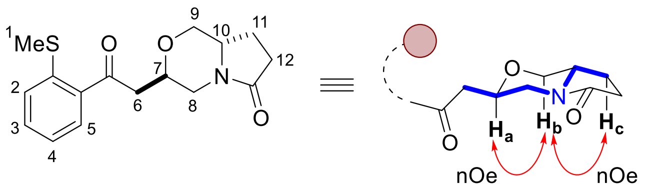

5y

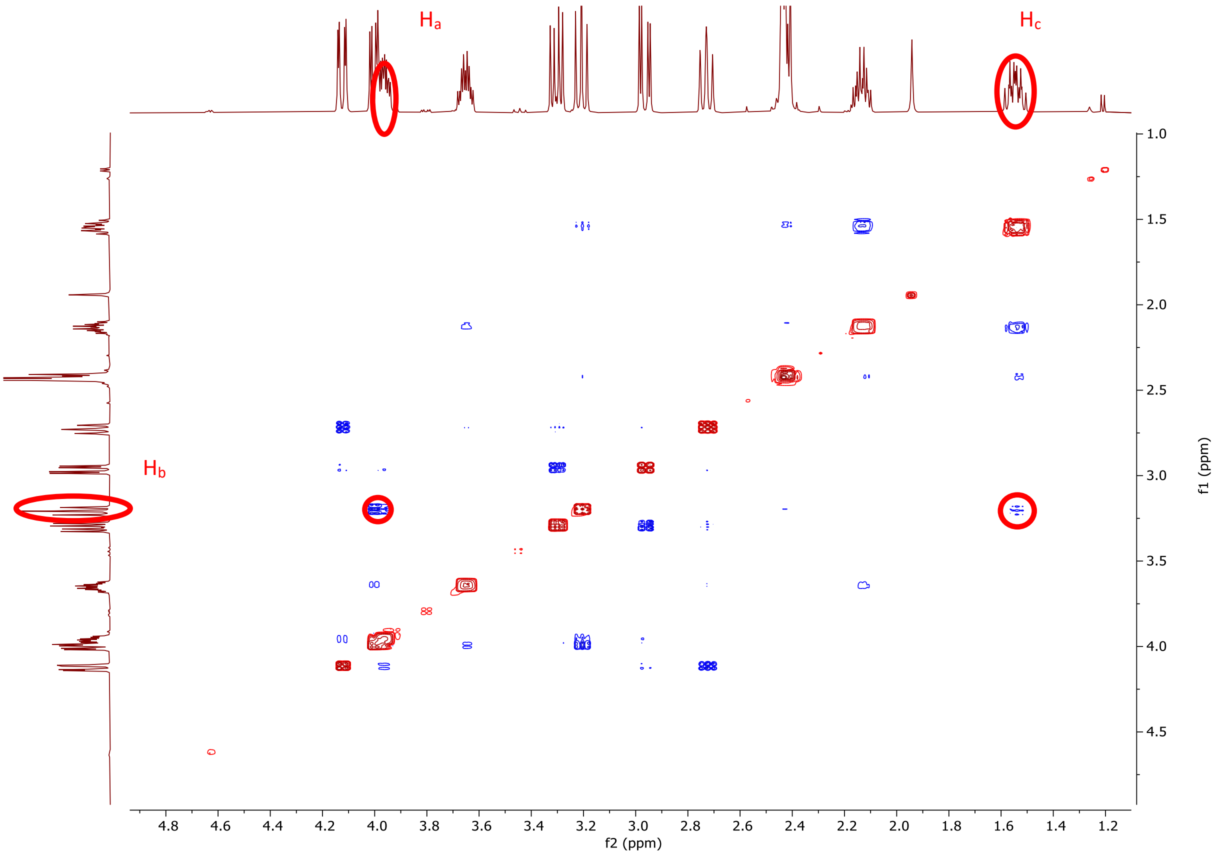

2-((3*R,6*S**)-5-Benzylmorpholin-3-yl)-1-(2-(methylthio)phenyl)ethan-1-one (5z) and 2-((3*R**,6*R**)-5-benzylmorpholin-3-yl)-1-(2-(methylthio)phenyl)ethan-1-one (5z')**

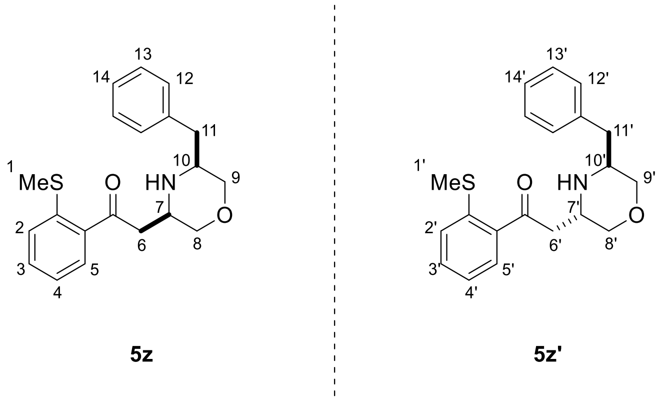

Following **general procedures H** and **J**. **General procedure H** used 2-(methylthio)benzaldehyde **1a** (40 μL, 0.300 mmol, 1.00 equiv.) and *tert*-butyl (1-phenyl-3-(prop-2-yn-1-yloxy)propan-2-yl)carbamate **2o** (104 mg, 0.360 mmol, 1.20 equiv.) with Rh(nbd)$_2$BF$_4$ (5.6 mg, 0.015 mmol, 0.050 equiv.), dcpe (6.3 mg, 0.015 mmol, 0.050 equiv.) and DCE (0.3 mL). **General procedure J** used *p*-TsOH·H$_2$O (114 mg, 0.600 mmol, 2.00 equiv.) and DCE (4.0 ml). Purification of the crude residue (2.4:1 dr) by column chromatography (1:1 Petrol:EtOAc to 0:100 EtOAc) yielded major diastereoisomer **5z** (33.4 mg, 0.098 mmol, 33%) as an off-white solid and minor diastereoisomer **5z'** (17.6 mg, 0.052 mmol, 17%, containing 15% **5z** by ^{1}H NMR integration) as a yellow viscous oil.

5z: **m.p.** (CH$_2$Cl$_2$) 58–60 °C; **δ$_H$** (400 MHz, CDCl$_3$) 7.79-7.72 (1H, m, *Ar*), 7.51-7.42 (1H, m, *Ar*), 7.34-7.25 (3H, m, *Ar*), 7.24-7.14 (4H, m, *Ar*), 3.84-3.74 (1H, m, C(8 and 9)*H*H), 3.50-3.41 (1H, m, C(7)*H*), 3.28-3.13 (3H, m, C(8 and 9)H*H*, C(10)*H*), 2.97-2.86 (2H, m, C(6)*H$_2$*), 2.63-2.53 (2H, m, C(11)*H$_2$*), 2.42 (3H, s, C(1)*H$_3$*); **δ$_C$** (101 MHz, CDCl$_3$) 199.5, 142.6, 137.7, 134.4, 132.6, 130.2, 129.2, 128.7, 126.6, 125.3, 123.7, 72.2, 71.5, 56.2, 51.5, 42.3, 39.4, 16.1; **ν$_{max}$**/ cm^{-1} (neat) 3323, 2847, 1667, 1587, 1558, 1454, 1433, 1107, 751, 701, 627; **m/z HRMS** (ESI$^+$) [M + H]$^+$ $C_{20}H_{24}O_2N_{32}S^+$ calc. 342.1522, found 342.1520.

5z': **δ_H** (400 MHz, $CDCl_3$) 7.77 (1H, dd, *J* = 7.9, 1.5 Hz, *Ar*'), 7.46 (1H, ddd, *J* = 8.6, 7.3, 1.5 Hz, *Ar*'), 7.35-7.25 (3H, m, *Ar*'), 7.25-7.13 (4H, m, *Ar*'), 3.85 (1H, dd, *J* = 11.1, 3.1 Hz, C(9')*H*H), 3.77 (1H, dd, *J* = 11.2, 3.1 Hz, C(8')*H*H), 3.73 (1H, dq, *J* = 4.9, 2.4 Hz, C(7')*H*), 3.57 (1H, dd, *J* = 11.1, 5.1 Hz, C(8')H*H*), 3.46 (1H, dd, *J* = 11.2, 6.5 Hz, C(9')H*H*), 3.31-3.11 (3H, m, C(6')H_2, C(10')*H*), 2.86-2.73 (2H, m, C(11')H_2), 2.42 (3H, s, C(1')H_3); **δ_C** (101 MHz, $CDCl_3$) 200.1, 142.5, 138.2, 134.6, 132.5, 130.3, 129.3, 128.8, 126.6, 125.3, 123.7, 71.2, 71.1, 52.0, 47.7, 41.1, 38.3, 16.1; **ν_{max}**/ cm^{-1} (neat) 3327, 2917, 2851, 1667, 1455, 1433, 1107, 751, 701; **m/z HRMS** (ESI^+) $[M + H]^+$ $C_{20}H_{24}O_2N^{32}S^+$ calc. 342.1522, found 342.1519.

2-((2*S,6*S**)-6-Butyltetrahydro-2*H*-pyran-2-yl)-1-(2-(methylthio)phenyl)ethan-1-one (5aa)**

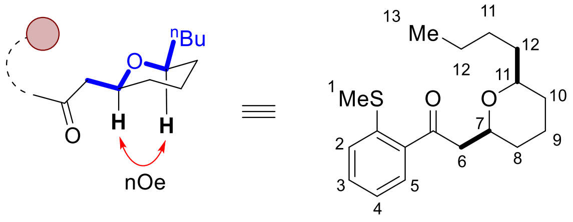

Following **general procedures H** and **I**. **General procedure H** used 2-(methylthio)benzaldehyde **1a** (40 μL, 0.300 mmol, 1.00 equiv.) and dec-9-yn-5-ol **2p** (56 mg, 0.360 mmol, 1.20 equiv.) with $Rh(nbd)_2BF_4$ (5.60 mg, 0.015 mmol, 0.050 equiv.), dcpe (6.30 mg, 0.015 mmol, 0.050 equiv.) and DCE (0.3 mL). **General procedure I** used HCl in dioxane (4.0 M in dioxane, 0.15 mL, 0.600 mmol, 2.00 equiv.) and DCE (4.0 mL). Purification of the crude residue (>20:1 dr) by column chromatography (1:49 to 1:24 EtOAc:Petrol) yielded the title compound as a single diastereoisomer **5aa** (56.8 mg, 0.187 mmol, 62%) as an off-white solid.

5aa: m.p. (CH_2Cl_2) 73–76 °C; **δ_H** (400 MHz, $CDCl_3$) 7.86 (1H, dd, *J* = 7.8, 1.4 Hz, *Ar*), 7.43 (1H, ddd, *J* = 8.7, 7.3, 1.5 Hz, *Ar*), 7.30 (1H, m, *Ar*), 7.16 (1H, app td, *J* = 7.8, 1.1 Hz, *Ar*), 3.99-3.85 (1H, m, C(7)*H*), 3.34-3.16 (2H, m, C(11)*H* and C(6)*H*H), 2.91 (1H, dd, *J* = 15.7, 6.2

Hz, C(6)H*H*), 2.41 (3H, s, C(1)H_3), 1.85-1.77 (1H, m, C(9)*H*H), 1.77-1.68 (1H, m, C(8)*H*H), 1.62-1.10 (10H, m, C(8 and 9)H*H*, C(10, 12, 13 and 14)H_2), 0.85 (3H, m, C(15)H_3); **δ_C** (101 MHz, $CDCl_3$) 200.0, 142.2, 135.3, 132.1, 130.8, 125.1, 123.5, 78.2, 74.8, 47.2, 36.3, 31.9, 31.5, 27.8, 23.7, 22.8, 16.1, 14.2; **ν_{max}**/ cm^{-1} (neat) 2930, 2858, 1670, 1587, 1559, 1462, 1433, 1372, 1344, 1280, 1194, 1067, 1046, 986; **m/z HRMS** (ESI^+) $[M + Na]^+$ $C_{18}H_{26}O_2{}^{23}Na^{32}S^+$ calc. 329.1546, found 329.1542.

1-(2-(methylthio)phenyl)-2-((2*R,5*S**)-5-phenyltetrahydrofuran-2-yl)ethan-1-one (5ab) and 1-(2-(methylthio)phenyl)-2-((2*R**,5*R**)-5-phenyltetrahydrofuran-2-yl)ethan-1-one (5ab')**

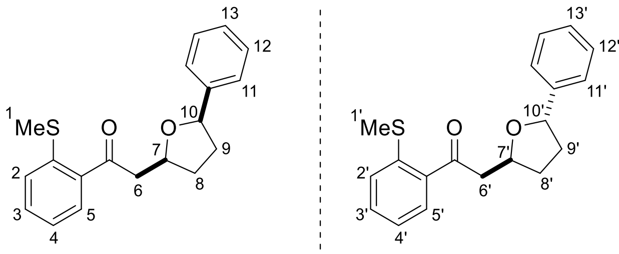

5ab **5ab'**

Following **general procedures H** and **I**. **General procedure H** used 2-(methylthio)benzaldehyde **1a** (40 μL, 0.300 mmol, 1.00 equiv.) and 1-phenylpent-4-yn-1-ol **2q** (58 mg, 0.360 mmol, 1.20 equiv.), with $Rh(nbd)_2BF_4$ (5.6 mg, 0.015 mmol, 0.050 equiv.), dcpe (6.3 mg, 0.015 mmol, 0.050 equiv.) and DCE (0.30 mL). **General procedure I** used HCl in dioxane (4 M in dioxane, 0.15 mL, 0.600 mmol, 2.00 equiv.) and DCE (4.0 mL). Purification of the crude residue (1:1 dr) by column chromatography (1:19 to 1:9 EtOAc:Petrol) yielded the title compound as an inseparable mixture of diastereoisomers **5ab** and **5ab'** (73.6 mg, 0.236 mmol, 79%) as an off white solid.

m.p. (CH_2Cl_2) 47–53 °C; **δ_H** (400 MHz, $CDCl_3$) (1:1 mixture) 7.92-7.87 (2H, m, *Ar*), 7.50-7.44 (2H, m, *Ar*), 7.36-7.29 (10H, m, *Ar*), 7.28-7.17 (4H, m, *Ar*), 5.04 (1H, dd, *J* = 8.0, 6.1 Hz, C(10')*H*), 4.90 (1H, t, *J* = 7.0 Hz, C(10)*H*), 4.82-4.73 (1H, m, C(7')*H*), 4.66-4.55 (1H, m, C(7)*H*), 3.59 (1H, dd, *J* = 16.3, 5.3 Hz, C(6)*H*H), 3.53 (1H, dd, *J* = 16.2, 5.4 Hz, C(6')*H*H),

3.18 (1H, dd, *J* = 16.3, 7.7 Hz, C(6)H*H*), 3.15 (1H, dd, *J* = 16.2, 7.5 Hz, C(6')*H*), 2.43 (3H, s, C(1 or 1')H_3), 2.43 (3H, s, C(1 or 1')H_3), 2.42-2.28 (4H, m, C(8 and 9)*H*H, C(8' and 9')*H*H), 1.97-1.71 (4H, m, C(8 and 9)H*H*, C(8' and 9')H*H*); **δ_C** (101 MHz, $CDCl_3$) (1:1 mixture) 199.5, 199.4, 143.6, 143.0, 142.6, 142.5, 134.6, 134.5, 132.3, 132.3, 130.7, 130.6, 128.4, 128.3, 127.2, 127.2, 125.8, 125.6, 125.1 (2C), 123.6, 123.5, 80.8, 80.4, 76.4, 76.3, 46.4, 46.3, 35.3, 34.2, 32.7, 31.7, 16.0 (2C); **ν_{max}**/ cm^{-1} (neat) 3650, 2980, 1668, 1586, 1557, 1493, 1462, 1433, 1382, 1270, 1082, 1054, 985, 951, 752, 700; **m/z HRMS** (ESI^+) $[M + H]^+$ $C_{19}H_{21}O_2{}^{32}S^+$ calc. 313.1258, found 313.1257.

NOESY spectrum of the 1:1 mixture 5ab and 5ab':

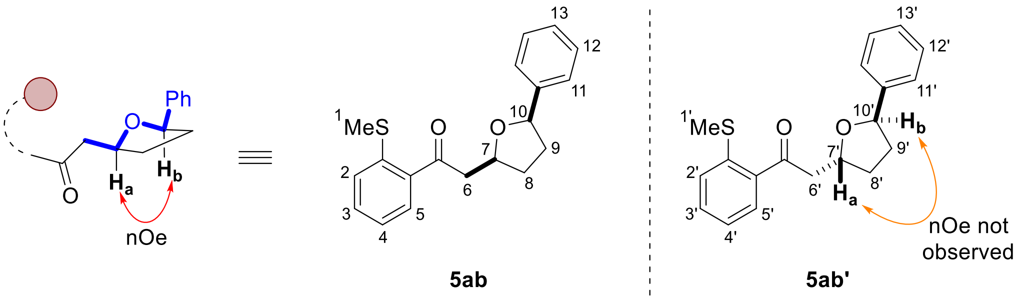

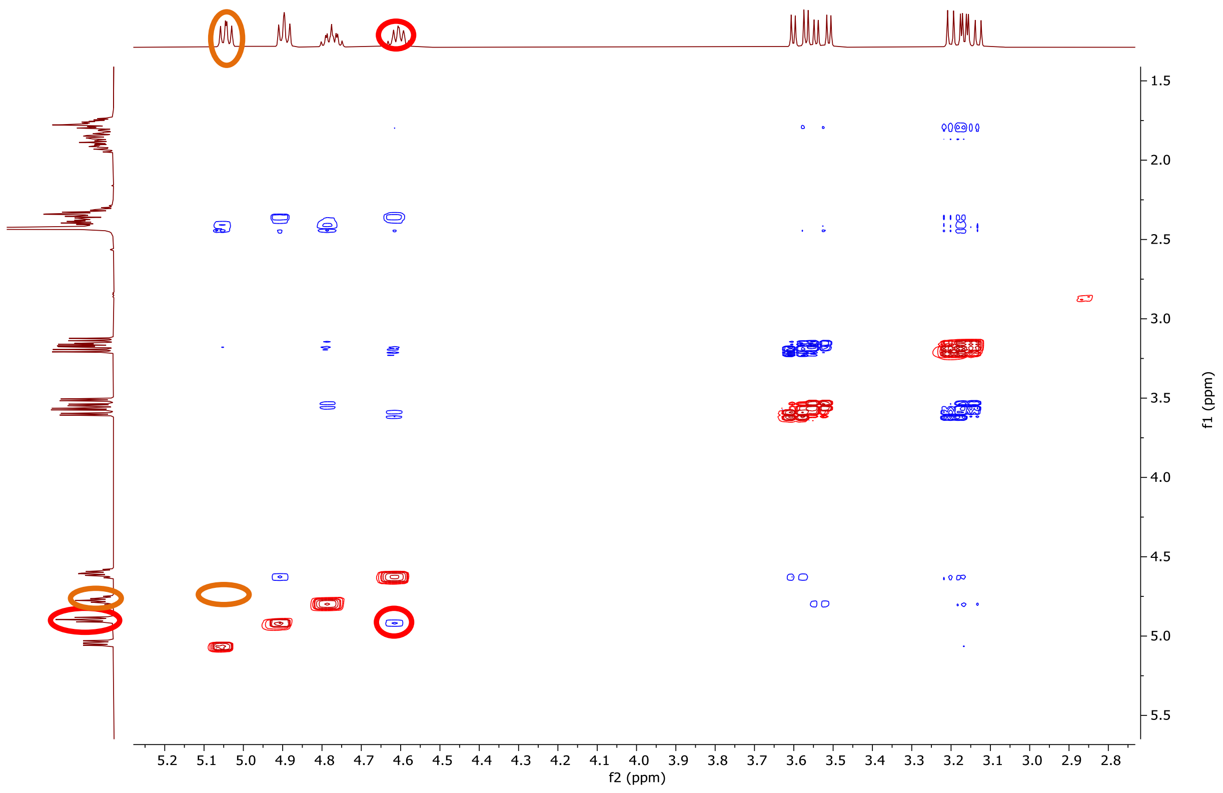

6.4 Alkyne scope with beta-amido aldehyde

2,2-Dimethyl-4-((2*R,3*R**)-3-methyl-1,4-dioxan-2-yl)-1-morpholinobutane-1,3-dione (5ac)**

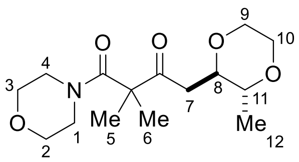

Following **general procedures H** and **I**. **Procedure H** used 2,2-dimethyl-3-morpholino-3-oxopropanal **1k** (55.5 mg, 0.300 mmol, 1.00 equiv.) and 2-(but-3-yn-2-yloxy)ethan-1-ol **2c** (41 mg, 0.360 mmol, 1.20 equiv.) with $Rh(nbd)_2BF_4$ (5.6 mg, 0.015 mmol, 0.05 equiv.), dcpe (6.3 mg, 0.015 mmol, 0.050 equiv.) and DCE (0.3 mL). **General procedure I** used HCl in dioxane (4.0 M in dioxane, 0.15 mL, 0.600 mmol, 2.00 equiv.) and DCE (4.0 mL). Purification of the crude residue (8.3:1 dr) by column chromatography (1:2 to 1:1 EtOAc:Petrol) yielded the title compound as an inseparable mixture of diastereoisomers **5ac** (80.1 mg, 0.268 mmol, 89%) as a pale-yellow viscous oil.

δ_H (500 MHz, $CDCl_3$) (major signals only) 3.81-3.07 (14H, m, C(1 to 4)H_2, C(9 and 10)H_2 and C(8 and 11)H), 2.61 (1H, dd, J = 16.9, 9.4 Hz, C(7)HH), 2.32 (1H, dd, J = 16.9, 2.4 Hz, C(7)HH), 1.33 (6H, d, J = 8.2 Hz, C(5 and 6)H_3), 1.04 (3H, d, J = 6.3 Hz, C(12)H_3); **δ_C** (101 MHz, $CDCl_3$) (major signals only) 207.5, 170.9, 76.8, 74.8, 66.8, 66.7, 66.6 (bs, 2C), 56.2, 46.6 (bs), 43.4 (bs), 39.9, 23.8, 22.8, 17.5; **ν_{max}**/ cm^{-1} (neat) 2855, 1712, 1637, 1422, 1273, 1247, 1113, 923, 847; **m/z HRMS** (ESI^+) $[M + Na]^+$ $C_{15}H_{25}O_5N^{23}Na^+$ calc. 322.1625, found 322.1625.

2,2-Dimethyl-1-morpholino-4-((2*R,3*R**)-3-pentyl-1,4-dioxan-2-yl)butane-1,3-dione (5ad)**

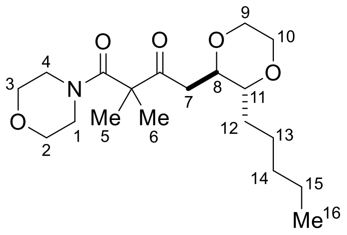

Following **general procedures H** and **I**. **General procedure H** used 2,2-dimethyl-3-morpholino-3-oxopropanal **1k** (55.5 mg, 0.300 mmol, 1.00 equiv.) and 2-(oct-1-yn-3-yloxy)ethan-1-ol **2d** (61 mg, 0.360 mmol, 1.20 equiv.) with $Rh(nbd)_2BF_4$ (5.6 mg, 0.015 mmol, 0.050 equiv.), dcpe (6.3 mg, 0.015 mmol, 0.050 equiv.) and DCE (0.3 mL). **General procedure I** used HCl in dioxane (4.0 M in dioxane, 0.15 mL, 0.600 mmol, 2.00 equiv.) and DCE (4.0 mL). Purification of the crude residue (11:1 dr) by column chromatography (1:4 to 2:3 EtOAc:Petrol) yielded the title compound as a single diastereoisomer **5ad** (72.5 mg, 0.204 mmol, 68%) as an off-white solid.

m.p. (CH_2Cl_2) 59–62 °C; **δ_H** (400 MHz, $CDCl_3$) 3.84-3.77 (1H, m, C(8)*H*), 3.77-3.72 (1H, m, C(9)*H*H), 3.72-3.12 (11H, br, C(1 to 4)H_2, C(9)H*H* and C(10)H_2), 3.25-3.18 (1H, m, C(11)*H*), 2.64 (1H, dd, *J* = 16.8, 9.3 Hz, C(7)*H*H), 2.39 (1H, app d, *J* = 16.8 Hz, C(7)H*H*), 1.53-1.43 (1H, m, C(12 to 15)H_2), 1.38 (3H, s, C(5 or 6)H_3), 1.36 (3H, s, C(5 or 6)H_3), 1.41 - 1.15 (7H, m, C(12 to 15)H_{2f}), 0.88 (3H, app t, *J* = 7.3 Hz, C(16)H_3); **δ_C** (101 MHz, $CDCl_3$) 207.7, 171.0, 78.6, 75.5, 66.9, 66.7 (bs, 2C), 66.6, 56.3, 46.4 (bs), 43.3 (bs), 39.9, 32.0, 31.5, 24.8, 23.9, 22.9, 22.6, 14.1; **ν_{max}**/ cm^{-1} (neat) 2926, 2855, 1713, 1638, 1422, 1360, 1272, 1247, 1184, 1115, 1022, 927, 888, 847; **m/z HRMS** (ESI^+) $[M + H]^+$ $C_{19}H_{34}O_5N^+$ calc. 356.2432, found 356.2434.

2,2-Dimethyl-4-((2*R,5*S**)-5-methyl-1,4-dioxan-2-yl)-1-morpholinobutane-1,3-dione (-(5ae)**

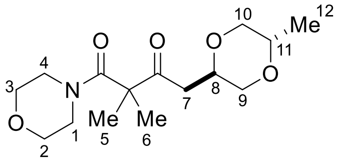

Following **general procedures H** and **I**. **General procedure H** used 2,2-dimethyl-3-morpholino-3-oxopropanal **1k** (55.5 mg, 0.300 mmol, 1.00 equiv.) and 2-(prop-2-yn-1-yloxy)propan-1-ol **2e** (41 mg, 0.360 mmol, 1.20 equiv.) with $Rh(nbd)_2BF_4$ (5.6 mg, 0.015 mmol, 0.050 equiv.), DPEPhos (8.1 mg, 0.015 mmol, 0.050 equiv.) and DCE (0.3 mL). **General procedure I** used HCl in dioxane (4 M in dioxane, 0.15 mL, 0.600 mmol, 2.00 equiv.) and DCE (4.0 mL). Purification of the crude residue (8:1 dr) by column chromatography (1:1 to 7:3 EtOAc:Petrol) yielded the title compound as an inseparable mixture of diastereoisomers **5ae** (80.3 mg, 0.269 mmol, 90%) as a yellow viscous oil.

δ_H (400 MHz, $CDCl_3$) (major signals only) 4.00 (1H, m, C(8)*H*), 3.71 (1H, dd, *J* = 11.3, 2.5 Hz, C(9)*H*H), 3.66-3.05 (10H, m, C(1 to 4)H_2, C(10)*H*H and C(11)*H*), 3.30 (1H, dd, *J* = 11.3, 10.6 Hz, C(9)H*H*), 3.25 (1H, dd, *J* = 11.4, 10.3 Hz, C(10)H*H*), 2.62 (1H, dd, *J* = 17.1, 8.0 Hz, C(7)*H*H), 2.26 (1H, dd, *J* = 17.1, 4.1 Hz, C(7)H*H*), 1.33 (3H, s, C(5 or 6)H_3), 1.33 (3H, s, C(5 or 6)H_3), 1.03 (3H, d, *J* = 6.3 Hz, C(12)H_3); δ_C (101 MHz, $CDCl_3$) (major signals only) 207.4, 170.9, 72.3, 71.1, 70.6, 70.5, 66.6 (bs, 2C), 56.1, 46.5 (bs), 43.3 (bs), 39.8, 23.7, 22.8, 16.7; ν_{max}/ cm^{-1} (neat) 2858, 1711, 1634, 1425, 1365, 1273, 1248, 1116, 1021, 908, 727, 648; **m/z HRMS** (ESI^+) $[M + Na]^+$ $C_{15}H_{25}O_5N^{23}Na^+$ calc. 322.1625, found 322.1623.

2,2-Dimethyl-1-morpholino-4-((2*R,5*S**)-5-phenyl-1,4-dioxan-2-yl)butane-1,3-dione (5af)**

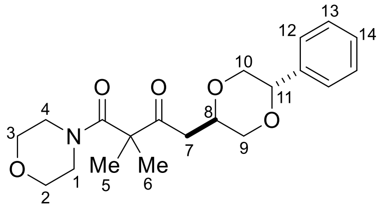

Following **general procedures H** and **I**. **General procedure H** used 2,2-dimethyl-3-morpholino-3-oxopropanal **1k** (55.5 mg, 0.300 mmol, 1.00 equiv.) and 2-phenyl-2-(prop-2-yn-1-yloxy)ethan-1-ol **2g** (63 mg, 0.360 mmol, 1.20 equiv.) with $Rh(nbd)_2BF_4$ (5.6 mg, 0.015 mmol, 0.050 equiv.), DPEPhos (8.1 mg, 0.015 mmol, 0.050 equiv.) and DCE (0.3 mL). **General procedure I** used HCl in dioxane (4.0 M in dioxane, 0.15 mL, 0.600 mmol, 2.00 equiv.) and DCE (4.0 mL). Purification of the crude residue (>20:1 dr) by column chromatography (1:4 to 1:1 EtOAc:Petrol) yielded the title compound as a single diastereoisomer **5af** (72.6 mg, 0.201 mmol, 67%) as a yellow viscous oil.

δ_H (400 MHz, $CDCl_3$) 7.39-7.24 (5H, m, *Ar*), 4.51 (1H, dd, *J* = 10.4, 2.8 Hz, C(11)*H*), 4.20 (1H, m, C(8)*H*), 3.94 (1H, dd, *J* = 11.5, 2.6 Hz, C(9)*H*H), 3.81 (1H, dd, *J* = 11.8, 2.8 Hz, C(10)*H*H), 3.75-3.09 (10H, br, C(1 to 4)H_2, C(9 and 10)H*H*), 2.72 (1H, dd, *J* = 17.2, 8.1 Hz, C(7)*H*H), 2.36 (1H, dd, *J* = 17.2, 4.1 Hz, C(7)H*H*), 1.39 (3H, s, C(5 or 6)H_3), 1.39 (3H, s, C(5 or 6)H_3); δ_C (101 MHz, $CDCl_3$) 207.3, 170.9, 137.9, 128.6, 128.3, 126.4, 77.6, 72.4, 70.8, 70.7, 66.6 (bs, 2C), 56.2, 46.7 (bs), 43.4 (bs), 39.8, 23.8, 22.9; ν_{max}/ cm^{-1} (neat) 2855, 1710, 1636, 1423, 1113, 1027, 701; **m/z HRMS** (ESI^+) $[M + H]^+$ $C_{20}H_{28}O_5N^+$ calc. 362.1957, found 362.1957.

4-((2*R,6*S**)-6-Benzyl-1,4-dioxan-2-yl)-2,2-dimethyl-1-morpholinobutane-1,3-dione (5ag)**

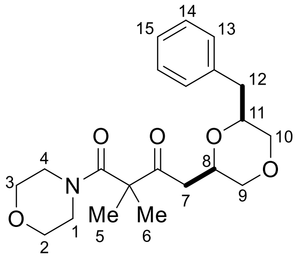

Following **general procedures H** and **I**. **General procedure H** used 2,2-dimethyl-3-morpholino-3-oxopropanal **1k** (55.5 mg, 0.300 mmol, 1.00 equiv.) and 1-phenyl-3-(prop-2-yn-1-yloxy)propan-2-ol **2j** (69 mg, 0.360 mmol, 1.20 equiv.) with $Rh(nbd)_2BF_4$ (5.6 mg, 0.015 mmol, 0.050 equiv.), DPEPhos (8.1 mg, 0.015 mmol, 0.050 equiv.) and DCE (0.3 mL). **General procedure H** used HCl in dioxane (4.0 M in dioxane, 0.15 mL, 0.600 mmol, 2.00 equiv.) and DCE (4.0 mL). Purification of the crude residue (11:1 dr) by column chromatography (1:4 to 1:1 EtOAc:Petrol) yielded the title compound as an inseparable mixture of diastereoisomers **5ag** (76.0 mg, 0.203 mmol, 68%) as a yellow viscous oil.

δ_H (400 MHz, $CDCl_3$) (major signals only) 7.33-7.16 (5H, m, *Ar*), 4.24-4.19 (1H, m, C(8)*H*), 3.99-3.87 (1H, m, C(11)*H*), 3.83-3.10 (8H, br, C(1 to 4)H_2), 3.75 (1H, dd, *J* = 11.2, 2.6 Hz, C(9)*H*H), 3.69 (1H, dd, *J* = 11.5, 2.5 Hz, C(10)*H*H), 3.23 (1H, dd, *J* = 11.2, 10.3 Hz, C(10)H*H*), 3.23 (1H, dd, *J* = 11.2, 10.4 Hz, C(9)H*H*), 2.79 (1H, dd, *J* = 14.0, 7.0 Hz, C(12)*H*H), 2.70 (1H, dd, *J* = 17.1, 7.8 Hz, C(7)*H*H), 2.61 (1H, dd, *J* = 14.2, 6.5 Hz, C(12)H*H*), 2.34 (1H, dd, *J* = 17.2, 4.4 Hz, C(7)H*H*), 1.38 (3H, s, C(5 or 6)H_3), 1.37 (3H, s, C(5 or 6)H_3); **δ_C** (101 MHz, $CDCl_3$) (major signals only) 207.3, 170.9, 137.1, 129.1, 128.5, 126.5, 75.9, 71.4, 70.3, 70.0, 66.6 (bs, 2C), 56.0, 46.3 (bs), 43.5 (bs), 40.1, 38.2, 23.6, 22.9; **ν_{max}**/ cm^{-1} (neat) 2853, 1711, 1637, 1496, 1454, 1423, 1360, 1272, 1248, 1183, 1115, 1022, 946, 915, 816, 731, 701, 640, **m/z HRMS** (ESI^+) $[M + Na]^+$ $C_{21}H_{29}O_5N^{23}Na^+$ calc. 398.1938, found 398.1939.

6.5 Production derivatisations

1-Phenyl-2-((2*R**,3*R**)-3-phenyl-1,4-dioxan-2-yl)ethan-1-one (11a)

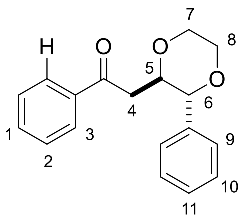

According to a modified literature procedure,[9] **5a** (30 mg, 0.092 mmol, >20:1 dr, 1.00 equiv.) was added to an oven-dried vial and vacuated and backfilled times with argon. A separate vial containing $Rh(nbd)_2BF_4$ (1.80 mg, 0.005 mmol, 0.050 equiv.) and dcpm (2.0 mg, 0.005 mmol, 0.050 equiv.) was additionally vac-cycled three times under argon, before adding anhydrous, argon degassed CH_2Cl_2 (1.0 mL). This mixture was then sonicated and hydrogen gas was bubbled through the resulting solution for 2-3 mins. The solvent was removed by a steady stream of argon gas before the residue dissolved by a known volume of anhydrous, argon degassed CH_2Cl_2 (0.6 mL). This solution was transferred to the vial containing substrate **5a** and stirred before triethoxysilane (34 µL, 0.018 mmol, 2.00 equiv.) was added. The resulting mixture was stirred at room temperature for 2 h. After this time, TLC analysis still showed a significant amount of starting material, so more triethoxysilane (34 µL, 0.018 mmol, 2.00 equiv.) was added and the mixture was stirred at room temperature for another 18 h. Product formation could be seen after this time; however some starting material was still remaining, so more triethoxysilane (102 µL, 0.054 mmol, 6.00 equiv., a total of 10.0 equiv.) was added and the reaction was stirred for another 4 h. After this time, only trace starting material could be seen by TLC analysis, and so the resulting solution was concentrated *in vacuo*. Purification by column chromatography (1:19 to 1:9 EtOAc:Petrol) yielded the title compound **11a** (15.6 mg, 0.055 mmol, 60%) as an off-white solid.

m.p. (CH_2Cl_2) 104–105 °C; **δ_H** (400 MHz, $CDCl_3$) 7.82-7.75 (2H, m, *Ar*), 7.55-7.49 (1H, m, *Ar*), 7.46-7.29 (7H, m, *Ar*), 4.38 (1H, d, *J* = 9.1 Hz, C(6)*H*), 4.24 (1H, app td, *J* = 9.1, 2.7 Hz, C(5)*H*), 3.97-3.80 (4H, m, C(6 and 7)H_2), 3.10 (1H, dd, *J* = 15.9, 9.0 Hz, C(4)*H*H), 2.66 (1H, dd, *J* = 15.8, 2.7 Hz, C(4)H*H*); **δ_C** (101 MHz, $CDCl_3$) 197.4, 138.1, 137.3, 133.2, 128.9, 128.8, 128.6, 128.3, 127.9, 83.0, 77.3, 67.2, 67.2, 40.4; **ν_{max}**/ cm^{-1} (neat) 3651, 2981, 2889, 1685, 1382, 1252, 1153, 1115, 1074, 954, 755, 700; **m/z HRMS** (ESI^+) $C_{18}H_{18}O_3{}^{23}Na$ $[M + Na]^+$ calc. 305.1148, found 305.1148.

1-(2-((*Z*)-2-(Methylthio)-2-phenylvinyl)phenyl)-2-((2*R,3*R**)-3-phenyl-1,4-dioxan-2-yl)ethan-1-one (11b)**

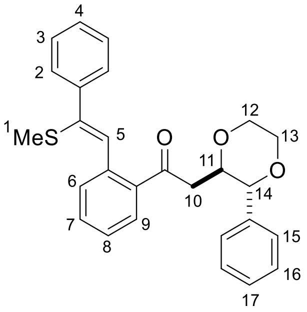

Prepared according to **general procedure H**, instead using **5a** (20.0 mg, 0.061 mmol, >20:1 dr, 1.00 equiv.), phenylacetylene (13 µL, 0.120 mmol, 2.00 equiv.), $Rh(nbd)_2BF_4$ (1.1 mg, 0.0031 mmol, 0.050 equiv.), Xantphos (1.8 mg, 0.0031 mmol, 0.050 equiv.) and DCE (0.2 mL, 0.3 M) under N_2, with reaction temperature at 100 °C.[36] After 5 h at 100 °C, TLC analysis showed low reaction conversion. Consequently, the reaction temperature was raised to 120 °C and the reaction mixture was stirred overnight (16 h). After this time, the reaction mixture was cooled to room temperature and concentrated *in vacuo*. Purification of the crude residue by column chromatography (1:19 to 1:9 EtOAc:Petrol) yielded the title compound **11b** (18.3 mg, 0.043 mmol, 70%) as a yellow viscous oil.

δ_H (400 MHz, $CDCl_3$) 7.69-7.52 (5H, m, *Ar*), 7.52-7.32 (9H, m, *Ar*), 7.03 (1H, s, C(5)*H*), 4.39 (1H, d, *J* = 9.1 Hz, C(14)*H*), 4.25 (1H, app td, *J* = 9.2, 2.9 Hz, C(11)*H*), 4.02-3.83 (4H, m, C(12

and 13)H_2), 3.17 (1H, dd, *J* = 15.8, 9.3 Hz, C(10)*H*H), 2.68 (1H, dd, *J* = 15.7, 2.8 Hz, (10)H*H*), 1.91 (3H, s, SC(1)H_3); **δC** (101 MHz, $CDCl_3$) 201.1, 140.1, 139.4, 138.3, 138.0, 136.7, 131.2, 130.9, 130.5, 128.8, 128.8, 128.5, 128.5, 128.1, 127.8, 127.2, 83.0, 77.6, 67.2, 67.1, 43.6, 16.3; **νmax**/ cm^{-1} (neat) 3735, 3059, 2959, 2951, 2855, 1685, 1490, 1473, 1269, 1100, 942, 758, 700, 648; **m/z HRMS** (ESI^+) $C_{27}H_{26}O_3{}^{23}Na^{32}S^+$ $[M + Na]^+$ calc. 453.1495, found 453.1494.

2-((2*R,3*R**)-3-Phenyl-1,4-dioxan-2-yl)-1-(2-(phenylethynyl)phenyl)ethan-1-one (11c)**

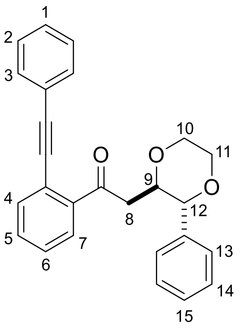

According to literature procedure,[8] $Rh(nbd)_2BF_4$ (2.8 mg, 0.0075 mmol, 0.050 equiv.) and dcpe (3.2 mg, 0.0075 mmol, 0.050 equiv.) were dissolved in DCE (2 mL). H_2 gas was bubbled through the solution for 2 min. The solution was purged with gas argon for 1 min. This was transferred *via* cannula to a mixture of aryl sulfide **5a** (49.0 mg, 0.150 mmol, >20:1 dr, 1.00 equiv.), phenylacetylene (33 μL, 0.300 mmol, 2.00 equiv.), copper bromide (43 mg, 0.300 mmol, 2.00 equiv.) and silver carbonate (41 mg, 0.150 mmol, 1.00 equiv.). The reaction mixture was heated at 80 °C for 16 h. Upon cooling to room temperature the mixture was concentrated under reduced pressure. Purification by column chromatography (1:19 to 1:9 EtOAc:Petrol) yielded the title compound **11c** (40.8 mg, 0.107 mmol, 71%) as a yellow viscous oil.

δH (400 MHz, $CDCl_3$) 7.56 (1H, dd, *J* = 7.7, 1.4 Hz, *Ar*), 7.53 (1H, dd, *J* = 7.8, 1.4 Hz, *Ar*), 7.46-7.29 (9H, m, *Ar*), 7.25-7.21 (3H, m, *Ar*), 4.31 (1H, d, *J* = 9.1 Hz, C(12)*H*), 4.23 (1H, app td, *J* = 9.2, 2.9 Hz, C(9)*H*), 3.94-3.76 (4H, m, C(10 and 11)H_2), 3.37 (1H, dd, *J* = 16.1, 9.2 Hz,

C(8)*H*H), 2.87 (1H, dd, *J* = 16.1, 2.9 Hz, C(8)H*H*); **δ_C** (101 MHz, $CDCl_3$) 200.7, 141.7, 137.9, 133.6, 131.7, 131.0, 128.8, 128.7, 128.5, 128.5, 128.4, 127.8, 122.9, 121.1, 94.9, 88.0, 83.0, 77.6, 67.2, 67.1, 44.2; **ν_{max}**/ cm^{-1} (neat) 3062, 2959, 2855, 1687, 1591, 1493, 1443, 1269, 1206, 1182, 1114, 995, 942, 914, 895, 756, 700, 691, 648, 614; **m/z HRMS** (ESI^+) $C_{26}H_{22}O_3{}^{23}Na^+$ $[M + Na]^+$ calc. 405.1461, found 405.1459.

(*R)-1-(2-(methylthio)phenyl)-2-((2*R**,3*R**)-3-phenyl-1,4-dioxan-2-yl)ethan-1-ol (11d) and (*S**)-1-(2-(Methylthio)phenyl)-2-((2*R**,3*R**)-3-phenyl-1,4-dioxan-2-yl)ethan-1-ol (11d')**

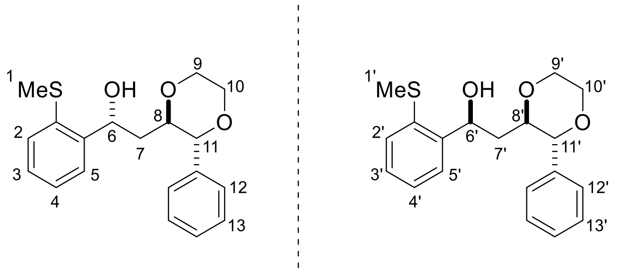

11d **11d'**

According to a modified literature procedure,[37] **5a** (30 mg, 0.091 mmol, >20:1 dr, 1.00 equiv.) and $Eu(OTf)_3$ (164 mg, 0.270 mmol, 3.00 equiv.) were added to a dry flask containing anhydrous Et_2O (1.6 mL). The flask was then purged with N_2 and cooled to –78 °C, with stirring. A solution of $LiBH_4$ (2 M in THF, 92 µL, 0.180 mmol, 2.00 equiv.) was then added dropwise and the resulting solution was stirred for 3 h at –78 °C. Once complete as monitored by TLC analysis, the mixture was quenched by addition of 1 M NaOH (1 mL) and the solution was warmed to room temperature. The mixture was then extracted with EtOAc (3 × 5 mL) and the combined organic extracts were dried over Na_2SO_4, filtered and concentrated *in vacuo*. Purification of the crude residue (8:1 dr) by column chromatography (1:4 EtOAc:Petrol) yielded a mixture of diastereoisomers (27.4 mg, 0.083 mmol, 91%), where pure fractions of both major diastereoisomer **11d** (23.1 mg, 0.070 mmol, 77%) and minor diastereoisomer **11d'**

(1.6 mg, 0.0048 mmol, 5%) could be partially isolated as an off-white solid and a colourless viscous oil respectively.

11d: **m.p.** (CH_2Cl_2) 139–140 °C; **δ_H** (400 MHz, $CDCl_3$) 7.43-7.38 (1H, m, *Ar*), 7.32-7.27 (5H, m, *Ar*), 7.25-7.12 (3H, m, *Ar*), 5.42-5.33 (1H, m, C(6)*H*), 4.32 (1H, d, *J* = 9.1 Hz, C(11)*H*), 4.00-3.87 (4H, m, C(9 and 10)H_2), 3.81 (1H, app td, *J* = 9.2, 2.7 Hz, C(8)*H*), 3.15-3.07 (1H, m, O*H*), 2.34 (3H, s, SC(1)H_3), 1.77 (1H, ddd, *J* = 14.7, 9.3, 2.8 Hz, C(7)*H*H), 1.60 (1H, ddd, *J* = 14.6, 8.9, 2.8 Hz, C(7)H*H*); **δ_C** (101 MHz, $CDCl_3$) 142.8, 138.1, 135.1, 128.6, 128.6, 127.9, 127.9, 126.9, 125.9, 125.6, 83.0, 78.0, 67.7, 67.1, 67.1, 37.7, 16.7; **ν_{max}**/ cm^{-1} (neat) 3448, 2954, 2917, 2855, 1439, 1266, 1115, 1094, 1036, 977, 943; **m/z HRMS** (ESI^+) $C_{19}H_{22}O_3{}^{23}Na^{32}S^+$ $[M + Na]^+$ calc. 353.1182, found 353.1181.

11d': **δ_H** (400 MHz, $CDCl_3$) 7.51-7.47 (1H, m, *Ar*'), 7.33-7.27 (5H, m, *Ar*'), 7.20-7.12 (3H, m, *Ar*'), 5.20 (1H, app dd, *J* = 9.0, 2.7 Hz, C(6')*H*), 4.21 (1H, d, *J* = 8.9 Hz, C(8')*H*), 4.05-3.86 (6H, m, O*H*, C(11')*H* and C(9' and 10')H_2), 2.23 (3H, s, SC(1')H_3), 1.70-1.58 (2H, m, C(7')H_2; **δ_C** (126 MHz, $CDCl_3$) 142.7, 137.9, 135.2, 128.7, 128.7, 127.8, 127.8, 127.1, 125.8, 125.7, 83.3, 82.0, 70.9, 67.1, 67.0, 39.0, 16.7; **ν_{max}**/ cm^{-1} (neat) 3427, 2979, 1380, 1141, 1109, 1083, 857; **m/z HRMS** (ESI^+) $C_{19}H_{22}O_3{}^{23}Na^{32}S^+$ $[M + Na]^+$ calc. 353.1182, found 353.1182.

(*R)-2-((2*R**,5*S**)-5-Methyl-1,4-dioxan-2-yl)-1-(2-(methylthio)phenyl)ethan-1-ol (11e) and (*S**)-2-((2*R**,5*S**)-5-methyl-1,4-dioxan-2-yl)-1-(2-(methylthio)phenyl)ethan-1-ol (11e')**

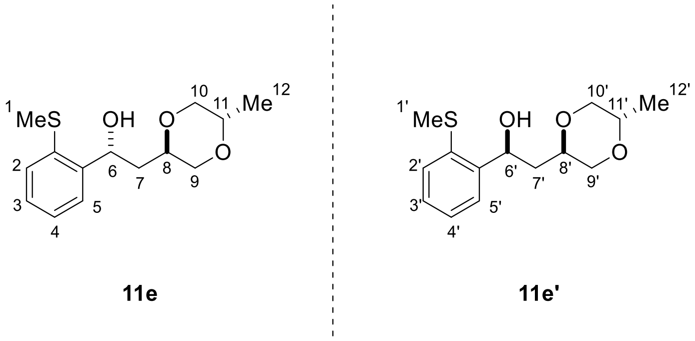

11e **11e'**

According to modified procedure,[37] **5o** (20.0 mg, 0.075 mmol, >20:1 dr, 1.00 equiv.) and Eu(OTf)$_3$ (135 mg, 0.225 mmol, 3.00 equiv.) were added to a dry flask containing anhydrous Et_2O (1.3 mL). The flask was then purged with N_2 and cooled to –78 °C, with stirring. A solution of $LiBH_4$ (2 M in THF, 75 µL, 0.150 mmol, 2.00 equiv.) was then added dropwise and the resulting solution was stirred for 2 h at –78 °C. Once complete as montiroed by TLC analysis, the mixture was quenched by addition of 1 M NaOH (1 mL) and the solution was warmed to room temperature. The mixture was then extracted with EtOAc (3 × 5 mL) and the combined organic extracts were dried over Na_2SO_4, filtered and concentrated *in vacuo*. Purification of the crude residue (3:1 dr) by column chromatography (1:19 to 1:5 EtOAc:Petrol) yielded a mixture of diastereoisomers (14.8 mg, 0.055 mmol, 74%), where pure fractions of both major diastereoisomer **11e** (8.1 mg, 0.03 mmol, 41%) and minor diastereoisomer **11e'** (3.6 mg, 0.015 mmol, 20%) could be partially isolated as off-white solids.

11e: **m.p.** (CH_2Cl_2) 109-113 °C; **δ_H** (400 MHz, $CDCl_3$) 7.55-7.50 (1H, m, *Ar*), 7.28-7.18 (3H, m, *Ar*), 5.40-5.31 (1H, m, C(6)*H*), 3.80 (1H, dd, *J* = 11.5, 2.5 Hz, C(10)*H*H), 3.77-3.62 (3H, m, C(8 and 11)*H*, C(9)*H*H), 3.52 (1H, dd, *J* = 11.5, 10.4 Hz, C(9)H*H*), 3.34 (1H, app d, *J* = 4.7 Hz, O*H*), 3.30 (1H, dd, *J* = 11.4, 10.2 Hz, C(10)*H*H), 2.47 (3H, s, SC(1)*H*$_3$), 1.86-1.79 (2H, m,

C(7)H_2), 1.07 (3H, d, J = 6.3 Hz, C(12)H_3); **δ_C** (101 MHz, $CDCl_3$) 142.5, 135.0, 127.9, 126.3, 125.8, 125.6, 73.0, 72.5, 71.2, 70.9, 68.1, 37.8, 16.8, 16.4; **ν_{max}**/ cm^{-1} (neat) 3421, 2972, 2853, 1440, 1378, 1350, 1280, 1150, 1119, 1097, 1051, 1023, 943, 886, 842, 822, 755, 735, 659, 618; **m/z HRMS** (ESI^+) $C_{14}H_{20}O_3{}^{23}Na^{32}S^+$ $[M + Na]^+$ calc. 291.1025, found 291.1025.

11e': **m.p.** (CH_2Cl_2) 105-106 °C; **δ_H** (400 MHz, $CDCl_3$) 7.60-7.56 (1H, m, *Ar*'), 7.25-7.17 (3H, m, *Ar*'), 5.35-5.31 (1H, m, C(6')*H*), 3.90 (1H, app tt, J = 10.1, 2.8 Hz, C(8')*H*), 3.85-3.78 (2H, m, O*H* and C(9')*H*H), 3.73 (1H, dd, J = 11.6, 2.5 Hz, C(10')*H*H), 3.71-3.61 (1H, m, C(11')*H*), 3.39 (1H, dd, J = 11.4, 10.2 Hz, C(9')H*H*), 3.37 (1H, dd, J = 11.6, 10.3 Hz, C(10')H*H*), 2.47 (3H, s, C(1')H_3), 1.80 (1H, app dt, J = 14.6, 2.7 Hz, C(7')*H*H), 1.64 (1H, app dt, J = 14.6, 9.8 Hz, C(7')H*H*), 1.10 (3H, d, J = 6.3 Hz, C(12')H_3); **δ_C** (101 MHz, $CDCl_3$) 142.4, 135.0, 127.9, 126.1, 125.8, 125.7, 76.0, 72.4, 71.3, 71.0, 70.5, 39.1, 16.9, 16.4; **ν_{max}**/ cm^{-1} (neat) 3435, 2920, 2853, 1441, 1348, 1310, 1282, 1118, 1100, 824, 755, 659; **m/z HRMS** (ESI^+) $C_{14}H_{20}O_3{}^{23}Na^{32}S^+$ $[M + Na]^+$ calc. 291.1025, found 291.1025.

(*E*)-1-(2-(Methylthio)phenyl)-2-((2*R,3*R**)-3-phenyl-1,4-dioxan-2-yl)ethan-1-one oxime (11f)**

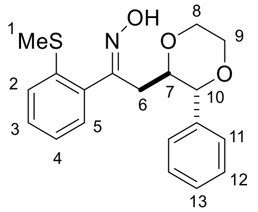

According to a modified literature procedure,[38] product **5a** (120 mg, 0.366 mmol, >20:1 dr, 1.00 equiv.), hydroxylamine hydrochloride (100 mg, 1.44 mmol, 4.00 equiv.) and pyridine (116 µL, 1.44 mmol, 4.00 equiv.) in MeOH (1.6 mL) were stirred at 60 °C for 20 h. Once complete as determined by TLC analysis, the solvent was removed *in vacuo*. H_2O (5 mL) was added and the residue extracted with EtOAc (3 × 5 mL). The combined organic extracts were washed with 1 M HCl (5 mL), dried over $MgSO_4$, filtered and concentrated *in vacuo*. Purification of the

crude residue by column chromatography (1:4 to 7:3 EtOAc:Petrol) yielded the title compound **11f** (69.9 mg, 0.204 mmol, 56%) as an off-white solid.

m.p. (CH_2Cl_2) 125–127 °C; **δ_H** (400 MHz, $CDCl_3$) 7.86-7.66 (1H, br, O*H*), 7.36-7.27 (5H, m, *Ar*), 7.22-7.12 (2H, m, *Ar*), 4.22 (1H, d, *J* = 8.9 Hz, C(10)*H*), 3.91-3.78 (3H, m, C(8 and 9)*H₂*), 3.77-3.60 (2H, m, C(7)*H*, C(8 and 9)*H₂*), 3.02 (1H, dd, *J* = 14.6, 10.6 Hz, C(6)*H*H), 2.57 (1H, dd, *J* = 14.6, 2.6 Hz, C(6)H*H*), 2.28 (3H, s, SC(1)H_3); **δ_C** (101 MHz, $CDCl_3$) 158.3, 138.1, 137.5, 136.0, 129.8, 129.2, 128.6, 128.6, 127.8, 127.2, 125.2, 83.3, 77.7, 67.0, 67.0, 30.6, 17.0; **ν_{max}**/ cm^{-1} (neat) 3340, 2856, 1699, 1585, 1522, 1473, 1434, 1354, 1271, 1115, 1046, 1008, 972, 942, 917, 756, 734, 700, 648; **m/z HRMS** (ESI^+) $C_{19}H_{21}O_3N^{23}Na^{32}S^+$ [M + Na]+ calc. 366.1134, found 366.1134.

3-(((2*R,3*R**)-3-phenyl-1,4-dioxan-2-yl)methyl)benzo[d]isothiazole (11g)**

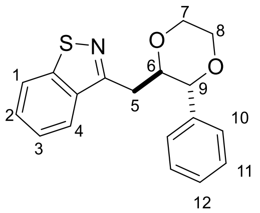

According to a modified literature procedure,[39] in an oven-dried microwave vial, oxime **11f** (69.9 mg, 0.204 mmol, >20:1 dr, 1.00 equiv.) was dissolved in pyridine (0.82 mL) and the vial was sealed under air. Acetic anhydride (78 µL, 0.816 mmol, 4.00 equiv.) was then added dropwise. This mixture was heated to 120 °C for 20 h. Once complete as determined by TLC analaysis, the reaction mixture was cooled to room temperature. Sat aq. NH_4Cl (20 mL) was added and the mixture was extracted with CH_2Cl_2 (3 × 20 mL). The combined organic extracts were dried over $MgSO_4$, filtered and concentrated *in vacuo*. Purification of the crude residue by column chromatography (1:19 to 1:9 EtOAc:Petrol) yielded the title compound **11g** (44.3 mg, 0.142 mmol, 70%) as an off-white solid.

m.p. (CH_2Cl_2) 108–110 °C; **δ_H** (400 MHz, $CDCl_3$) 7.87 (1H, app dt, *J* = 8.2, 0.9 Hz, *Ar*), 7.69 (1H, app dt, *J* = 8.2, 1.0 Hz, *Ar*), 7.52-7.44 (3H, m, *Ar*), 7.42-7.30 (4H, m, *Ar*), 4.43 (1H, d, *J* = 9.0 Hz, C(9)*H*), 4.15 (1H, td, *J* = 9.1, 3.0 Hz, C(6)*H*), 3.95-3.86 (2H, m, C(7 and 8)*H*H), 3.85-3.76 (2H, m, C(7 and 8)H*H*), 3.17 (1H, dd, *J* = 14.6, 9.1 Hz, C(5)*H*H), 2.98 (1H, dd, *J* = 14.6, 3.0 Hz, C(5)H*H*); **δ_C** (101 MHz, $CDCl_3$) 163.0, 152.5, 138.3, 135.2, 128.8, 128.8, 128.0, 127.4, 124.4, 123.7, 119.9, 83.2, 79.4, 67.1, 67.0, 33.7; **ν_{max}**/ cm^{-1} (neat) 2958, 2854, 1593, 1491, 1453, 1371, 1350, 1324, 1272, 114, 1009, 974, 942, 914, 894, 757, 735, 701, 649, 622; **m/z HRMS** (ESI^+) $C_{18}H_{18}O_2N^{32}S^+$ $[M + H]^+$ calc. 312.1053, found 312.1054**.**

7. Synthesis of *S*-heterocycles

7.1 Substrate reaction procedures

General procedure K: Tosylation of alkynols Method 1

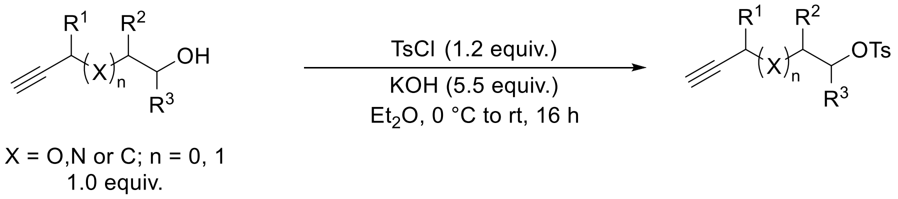

According to a modified literature procedure,[19] tosyl chloride (1.2 equiv.) was added to an oven dried flask followed by anhydrous diethyl ether. A given alkynol (1.0 equiv.) was introduced and the solution cooled to 0 °C. Powdered potassium hydroxide (5.5 equiv.) was then added in portions. The solution was allowed to warm to room temperature and stirred under a nitrogen atmosphere overnight. Upon completion, the suspension was added to ice water (20 mL) and extracted with EtOAc (3 × 20 mL). The combined organic extracts were dried over anhydrous $MgSO_4$, filtered and concentrated *in vacuo* to give the crude product.

General procedure L: Tosylation of alkynols Method 2

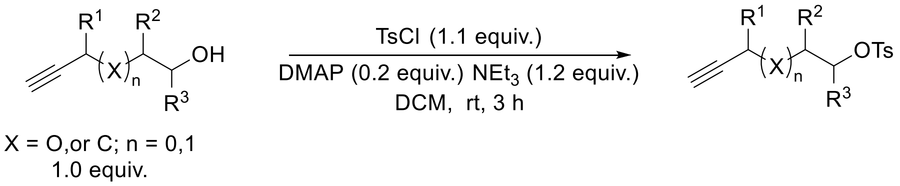

According to a modified literature procedure,[40] a given alkynol (1.0 equiv.), DMAP (0.2 equiv.), and TsCl (1.1 equiv.) were dissolved in CH_2Cl_2. Anhydrous NEt_3 (1.2 equiv.) was then introduced and the solution stirred at room temperature for 3 h, unless otherwise stated. Sat. aq. NH_4Cl (10 mL) was then added and the mixture extracted with CH_2Cl_2 (3 × 40 mL). The combined organic extracts were washed with brine (20 mL), dried over anhydrous $MgSO_4$, filtered and concentrated *in vacuo* to give the crude product.

General procedure M: Thiolation of tosylated compounds Method 1

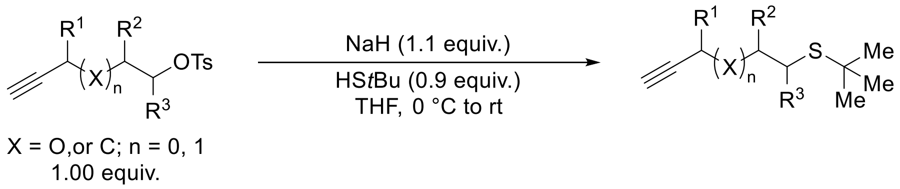

To a pre-dried flask was added NaH (60% dispersion in mineral oil,1.1 equiv.) and THF. The suspension was then cooled to 0 °C. *Tert*-Butylthiol (0.9 equiv.) was then introduced and the mixture stirred for 10 min. Next, a solution of a given tosylated alkynol (1.0 equiv) in THF was added to the suspension. Upon completion of the reaction, as indicated by TLC, the solvent was removed under reduced pressure. The crude mixture was then dissolved in Et_2O (20 mL), then sequentially washed with water (2 × 20 mL) and sat. aq. $NaHCO_3$ (1 × 20 mL). The combined organic extracts were dried over anhydrous $MgSO_4$, filtered and concentrated *in vacuo* to give the crude product.

General procedure N: Thiolation of tosylated compounds Method 2

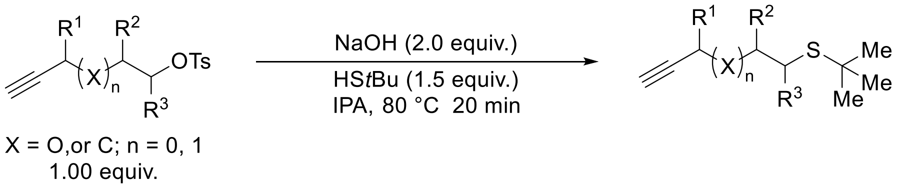

According to a modified literature procedure,[41] powdered NaOH (2.0 equiv.), isopropanol and *tert*-butylthiol (1.5 equiv.) were added to a pre-dried flask. The suspension was stirred for 10 min, after which a solution of a given tosylated alkynol (1.0 equiv.) in isopropanol was added to the suspension. The mixture was then heated at 80 °C under an argon atmosphere for 20 min, unless otherwise stated. Upon cooling to room temperature, the mixture was diluted with ice water (20 mL) and then extracted with EtOAc (3 × 20 mL). The combined organic extracts were dried over anhydrous $MgSO_4$, filtered and concentrated *in vacuo*.

General procedure O: Propargylation of alcohols

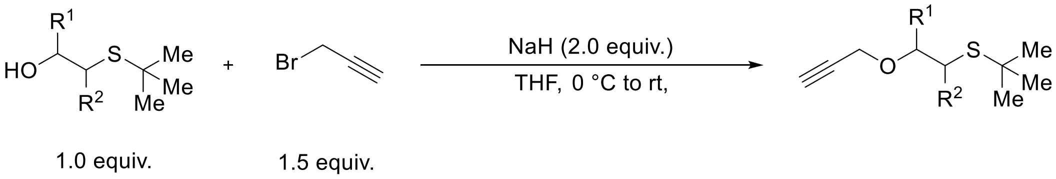

To a pre-dried flask was added NaH (60% dispersion in mineral oil, 2.0 equiv) and THF. The suspension was then cooled to 0 °C. A given alcohol (1.0 equiv.) was then introduced and the mixture stirred for 5 min. Next, propargyl bromide (80 w/w in toluene, 1.5 equiv.) was added dropwise over 20 min. The mixture was then warmed to room temperature and stirred until completion as monitored by TLC analysis. The reaction was quenched with sat. aq. NH_4Cl (10 mL), diluted with water (10 mL) then extracted with EtOAc (3 × 20 mL). The combined organic extracts were washed with brine (20 mL), dried over anhydrous $MgSO_4$, filtered and concentrated *in vacuo*.

General procedure P: Thiolation of alpha-bromo ketones

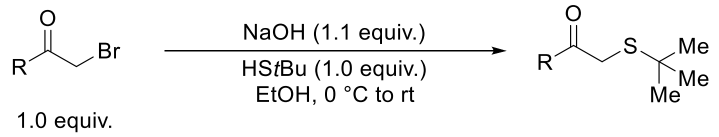

Powdered NaOH (1.1 equiv.) and ethanol were added to a pre-dried round-bottomed flask. The suspension was then cooled to 0 °C. *tert*-Butylthiol (1.0 equiv.) was then introduced dropwise and the mixture stirred for 10 min. Next, a solution of a given alpha-bromo ketone (1.0 equiv) in ethanol was added to the suspension. The reaction mixture was then stirred at room temperature. Upon completion of the reaction, as indicated by TLC, the solution was diluted with Et_2O (100 mL). The mixture was then washed with water (2 × 20 mL) and sat. aq. $NaHCO_3$ (1 × 20 mL). The combined organic extracts were dried over anhydrous $MgSO_4$, filtered and concentrated *in vacuo*. The resultant crude residue was then carried on to the next step.

General procedure Q: Thiolation of alpha-bromo ketones method 2

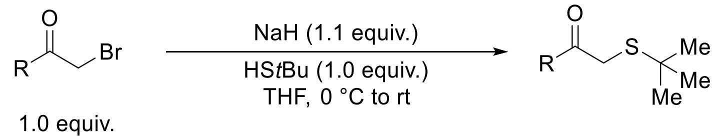

To a pre-dried flask was added NaH (60% dispersion in mineral oil, 1.1 equiv.) and THF. The suspension was then cooled to 0 °C. *Tert*-Butylthiol (1.0 equiv.) was then introduced and the mixture stirred for 10 min. Next, a solution of a given alpha-bromo ketone (1.0 equiv) in THF was added to the suspension. Upon completion of the reaction, as indicated by TLC, the solvent was removed under reduced pressure. The crude mixture was then dissolved in Et_2O (100 mL), then sequentially washed with water (2 × 20 mL) and sat. aq. $NaHCO_3$ (1 × 20 mL). The combined organic extracts were dried over anhydrous $MgSO_4$, filtered and concentrated *in vacuo* to give the crude product.

General Procedure R: $NaBH_4$ reduction of alpha-sulfido ketones

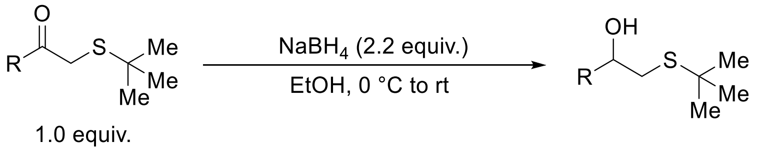

$NaBH_4$ (2.2 equiv.) and ethanol were added to a pre-dried round-bottomed flask. The suspension was then cooled to 0 °C. A solution of the crude ketone (1.0 equiv.) (from general prodecure P) dissolved in ethanol was then introduced dropwise. The mixture was then warmed to room temperature and stirred under a nitrogen atmosphere. Upon completion of the reaction, as indicated by TLC, the reaction was quenched by dropwise addition of 1M aq. HCl (10 mL) at 0 °C. The solution was then extracted with CH_2Cl_2 (3 × 20 mL). The combined organic extracts were dried over anhydrous $MgSO_4$, filtered and concentrated *in vacuo*. The resultant crude residue was used, without further purification, in the next step.

7.2 Aldehyde and alkyne substrates

3-Morpholino-3-oxopropanenitrile (S20), 1-(morpholine-4-carbonyl)cyclopentane-1-carbonitrile (S21) and 1-(morpholine-4-carbonyl)cyclopentane-1-carbaldehyde (1j)

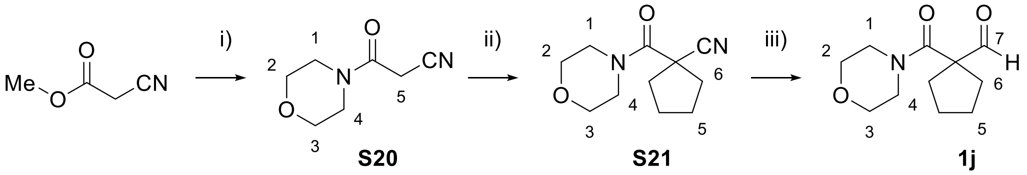

3-Morpholino-3-oxopropanenitrile (S20): Prepared acccording to a modified literature procedure,[42] methyl cyanoacetate (19.8 g, 200 mmol) and morpholine (17.5 mL, 200 mmol) were combined in a round-bottomed flask. The resulting solution was stirred at room temperature for 24 h under a nitrogen atmosphere. The solution was then concentrated under reduced pressure to give orange/red crystals. The crystals were washed with cold Et_2O (50 mL) and dried under vacuum, yielding title compound **S20** (22.3 g, 145 mmol, 72%) as orange/red crystals.

δ_H (400 MHz, $CDCl_3$) 3.71-3.62 (4H, m, C(1 to 4)H_2), 3.61-3.55 (2H, m, C(1 to 4)H_2), 3.52 (2H, s, C(5)H_2), 3.45-3.38 (2H, m, C(1 to 4)H_2); **δ_C** (101 MHz, $CDCl_3$) 165.9, 122.3, 66.8 (bs), 66.1 (bs), 47.5 (bs), 45.0, 43.9 (bs), 37.5, 25.3; **ν_{max}**/ cm^{-1} (neat) 2972, 2928, 2872, 2264, 1662, 1650, 1615, 1532, 1466, 1449, 1430, 1390, 1364, 1318, 1278, 1242, 1217, 1194, 1105, 1067, 1032, 1012, 975, 928, 900; **m/z HRMS** (ESI^+) $[M + H]^+$ $C_7H_{11}N_2O_2^+$ calc. 155.0815, found 155.0815. *Data is consistent with literature*[42]

1-(Morpholine-4-carbonyl)cyclopentane-1-carbonitrile (S21): Prepared according to a modified literature procedure.[16] To a pre-dried flask was added 3-morpholino-3-oxopropanenitrile **S20** (4.62 g, 30.0 mmol) and powdered K_2CO_3 (10.4 g, 75.0 mmol). DMF (60 mL) was added, followed by 1,4-dibromobutane (8.6 mL, 72.0 mmol) and 1-butyl-3-methylimidazolium tetrafluoroborate (1.1 mL, 6.0 mmol). The resulting mixture was stirred at room temperature overnight under a nitrogen atmosphere. The reaction mixture was then filtered and washed with Et_2O (30 mL). The filtrate was diluted with water (200 mL) and extracted with Et_2O (4 × 100 mL). The combined organic extracts were dried over anhydrous $MgSO_4$, filtered and concentrated *in vacuo*. Purification by column chromatography (7:3 then 3:2 Petrol:EtOAc) yielded title compound **S21** (5.19 g, 24.9 mmol, 83%) as white crystals.

m.p 44–45 °C; **δ_H** (400 MHz, $CDCl_3$) 3.79-3.61 (8H, m, C(1 to 4)H_2), 2.45-2.35 (2H, m, C(5 and 6)H_2), 2.27-2.21 (2H, m, C(5 and 6)H_2), 1.92-1.74 (4H, m, C(5 and 6)H_2); **δ_C** (101 MHz, $CDCl_3$) 165.9, 122.3, 66.8, 66.1, 47.5, 45.0, 43.9, 37.5, 25.3; **ν_{max}**/ cm^{-1} (neat) 2966, 2946, 2922, 2866, 2851, 2229, 1738, 1695, 1643,1455, 1424, 1358, 1331,1304, 1270, 1240, 1206, 1174, 1114, 1067, 1035, 1017, 994, 983, 953, 932, 912; **m/z HRMS** (ESI^+) $[M + H]^+$ $C_{11}H_{17}N_2O_2^+$ calc. 209.1285, found 209.1287. *Data is consistent with literature.*[16]

1-(Morpholine-4-carbonyl)cyclopentane-1-carbaldehyde (1j): Prepared according to a modified procedure.[16] Nitrile **S21** (1.25 g, 6.0 mmol) was dissolved in a mixture of water (15

mL) acetic acid (15 mL) and pyridine (30 mL). The solution was cooled to 0 ºC and Raney®-Nickel (50% w/w in water, 8.45 g, 72.0 mmol) was added followed by portion-wise addition of sodium hypophosphite monohydrate (4.45 g, 42.0 mmol). The reaction mixture was stirred at 90 ºC for 16 h. Upon cooling to room temperature, the mixture was filtered through a Celite® plug and the filtrate extracted with CH_2Cl_2 (3 × 30 mL). The combined organic extracts were washed with 1M HCl (50 mL), dried over anhydrous $MgSO_4$, filtered and concentrated *in vacuo*. The crude residue (676 mg) was purified according to a modified literature procedure,[43] by first dissolving it in a mixture of EtOAc (5 ml), EtOH (1 ml) and water (0.2 ml). Sodium bisulfite (286 mg, 2.75 mmol) was then introduced and the mixture stirred at 40 °C for 4 h. Upon cooling to room temperature, the mixture was filtered and washed with ethanol (10 mL). After drying, the bisulfite adduct (0.443 g) was obtained as a flaky white solid. This adduct was combined with TMS-Cl (0.49 mL, 3.88 mmol) and acetonitrile (4 mL). The mixture was then stirred at 40 °C for 2 h. Upon cooling to room temperature, the mixture was diluted with EtOAc (20 mL) and washed with water (3 × 20 mL) and brine (3 × 20 mL). The combined organics were dried over anhydrous $MgSO_4$, filtered and concentrated *in vacuo*, to give title compound **1j** (135 mg, 0.639 mmol, 46%) as a yellow oil.

δ_H (400 MHz, $CDCl_3$) 9.48 (1H, s, C(7)*H*=O), 3.63 (6H, s, C(1 to 4)H_2), 3.25 (2H, s, C(1 to 4)H_2), 2.32-2.01 (4H, m, C(6)H_2), 1.77-1.51 (4H, m, C(5)H_2); **δ_C** (101 MHz, $CDCl_3$) 197.9, 169.3, 66.9 (bs), 66.6 (bs), 65.5, 46.6 (bs), 43.5 (bs), 34.7, 31.6, 26.0, 24.8; **ν_{max}**/ cm^{-1} (neat) 3294, 2959, 2863, 2722, 1716, 1637, 1426, 1361, 1300, 1272, 1238, 1216, 1177, 1114, 1066, 1017, 943, 909; **m/z HRMS** (ESI^+) $[M + H]^+$ $C_{11}H_{18}NO_3^+$ calc. 212.1281, found 212.1283. *Data is consistent with literature[16]*

***S*-(Hex-5-yn-1-yl) ethanethioate (S22) and hex-5-yne-1-thiol (6a)**

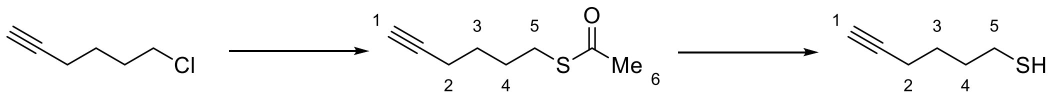

S22 **6a**

***S*-(Hex-5-yn-1-yl) ethanethioate (S22):** Prepared according to a modified procedure.[44] 6-Chlorohex-1-yne (0.6 mL, 5.0 mmol) was dissolved in anhydrous DMF (5 mL) followed by addition of potassium thioacetate (900 mg, 6.0 mmol). The mixture was stirred at 50 °C under an atmosphere of nitrogen for 3.5 h. Upon cooling to room temperature, the solution was extracted with Et_2O (3 × 20 mL). The combined organic extracts were washed with water (5 × 20 mL), dried over anhydrous $MgSO_4$, filtered and concentrated *in vacuo*. Purification by column chromatography (9:1 Petrol:Et_2O) afforded alkyne **S22** (0.6 g, 83%) as an orange/red oil.

δ_H (400 MHz, $CDCl_3$) 2.82 (2H, t, *J* = 7.1 Hz, C(5)H_2), 2.26 (3H, s, C(6)H_3), 2.15 (2H, td, *J* = 6.9, 2.7 Hz, C(2)H_2), 1.90 (1H, t, *J* = 2.6 Hz, C(1)*H*), 1.68-1.58 (2H, m, C(4)H_2), 1.58-1.48 (2H, m, C(3)H_2); **δ_C** (101 MHz, $CDCl_3$) 195.7, 83.8, 68.7, 30.6, 28.5, 28.5, 27.3, 17.9; **ν_{max}**/ cm^{-1} (neat) 3293, 2940, 2863, 2117, 1687, 1431, 1354, 1297, 1264, 1132, 1107, 954. *Data is consistent with literature[44]*

Hex-5-yne-1-thiol (6a): Prepared according to a modified literature procedure.[44] In a pre-dried flask, anhydrous THF (13 mL) was cooled to 0 °C and $LiAlH_4$ (19.9 mL, 1 M THF) introduced. The solution was stirred for 5 min then *S*-(hex-5-yn-1-yl) ethanethioate **S22** (2.34 g, 15 mmol) was added dropwise. The mixture was gradually warmed to room temperature with stirring under an atmosphere of nitrogen. After 20 min, the reaction was cooled to 0 °C and quenched with slow addition of water (10 mL). Next, 20 mL of 1 M HCl was added. The solution was extracted with Et_2O (3 × 20 mL). The combined organic extracts were dried over anhydrous $MgSO_4$, filtered and concentrated *in vacuo*. Purification by column chromatography

(98:2 Petrol:EtOAc) yielded title compound **6a** (300 mg, 12.3 mmol, 82%) as a pale-yellow oil.

δ_H (400 MHz, $CDCl_3$) 2.58-2.53 (1H, m, C(5)H_2S), 2.22 (2H, td, *J* = 6.9, 2.6 Hz, C(2)H_2), 1.96 (1H, t, *J* = 2.6 Hz, C≡C(1)*H*), 1.78-1.69 (2H, m, C(4)H_2), 1.68-1.60 (2H, m, C(3)H_2), 1.35 (1H, t, *J* = 7.8 Hz, S*H*); **δ_C** (101 MHz, $CDCl_3$) 83.9, 68.6, 32.9, 27.0, 24.1, 17.9; **ν_{max}**/ cm^{-1} (neat) 3293, 2933, 1433, 1264. *Data is consistent with literature.[44]*

Hex-5-yn-1-yl 4-methylbenzenesulfonate (S23) and *tert*-butyl(hex-5-yn-1-yl)sulfide (6b)

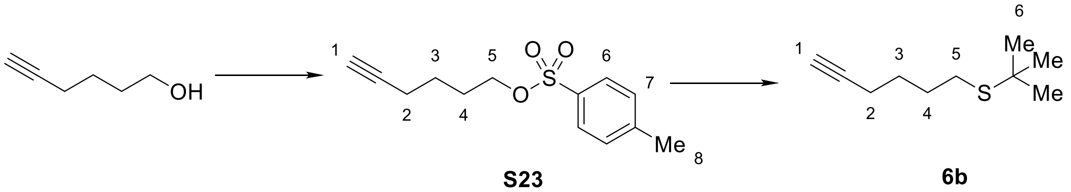

Hex-5-yn-1-yl 4-methylbenzenesulfonate (S23): Synthesised according to **general procedure K**, using hex-5-ynol (1.7 mL, 15.4 mmol) and diethyl ether (32 mL). Purification by column chromatography (4:1 Hexane:EtOAc) yielded title compound **6b** (3.80 g, 15.1 mmol, 99%) as a viscous colourless oil.

δ_H (400 MHz, $CDCl_3$) 7.79 (2H, d, *J* = 8.3 Hz, *Ar*), 7.35 (2H, d, *J* = 7.9 Hz, *Ar*), 4.06 (2H, t, *J* = 6.3 Hz, C(5)H_2), 2.45 (3H, s, C(8)H_3), 2.16 (2H, td, *J* = 6.9, 2.7 Hz, C(2)H_2), 1.92 (1H, t, *J* = 2.6 Hz, C(1)*H*), 1.81-1.74 (2H, m, C(3)H_2), 1.59-1.52 (2H, m, C(4)*H*); **δ_C** (101 MHz, $CDCl_3$) 144.7, 133.1, 129.8, 127.9, 83.4, 69.9, 68.9, 27.7, 24.2, 21.6, 17.7; **ν_{max}**/ cm^{-1} (neat) 3290, 2955, 2117, 1734, 1598, 1495, 1454, 1434, 1354, 1307, 1291, 1244, 1211, 1188, 1172, 1119, 1097, 1043, 1017, 931; **m/z HRMS** (ESI^+) $C_{13}H_{17}O_3S^+$ $[M+H]^+$ calc. 253.0893 found 253.0865; *Data consistent with literature.*[45]

tert-Butyl(hex-5-yn-1-yl)sulfide (6b): Synthesised according to **general procedure N**, using hex-5-yn-1-yl 4-methylbenzenesulfonate **S23** (760 mg, 3.03 mmol) and IPA (7.5 mL). Purification by column chromatography (96:4 Hexane:EtOAc) yielded title compound **6b** (360 mg, 2.11 mmol, 71%) as a pale-yellow oil.

δ_H (400 MHz, $CDCl_3$) 2.55 (2H, t, *J* = 7.1 Hz, C(5)H_2), 2.22 (2H, td, *J* = 6.8, 2.6 Hz, C(2)H_2), 1.94 (1H, t, *J* = 2.7 Hz, C(1)*H*), 1.78-1.58 (4H, m, C(3)H_2 and C(4)H_2), 1.32 (9H, s, C(6)H_3); **δ_C** (101 MHz, $CDCl_3$) 84.1, 68.5, 41.8, 30.9, 28.8, 27.8, 27.7, 18.0; **ν_{max}**/ cm^{-1} (neat) 3298, 2942, 2862, 2118, 1471, 1458, 1390, 1363, 1327, 1296, 1263, 1239, 1163, 1050, 933; **m/z HRMS** (ESI^+) $[M + H]^+$ $C_{10}H_{19}S^+$ calc. 171.1202, found 171.1205. *Data is consistent with literature.*[41]

2-(Prop-2-yn-1-yloxy)ethyl 4-methylbenzenesulfonate (S24) and *tert*-butyl(2-(prop-2-yn-1-yloxy)ethyl)sulfane (6c)

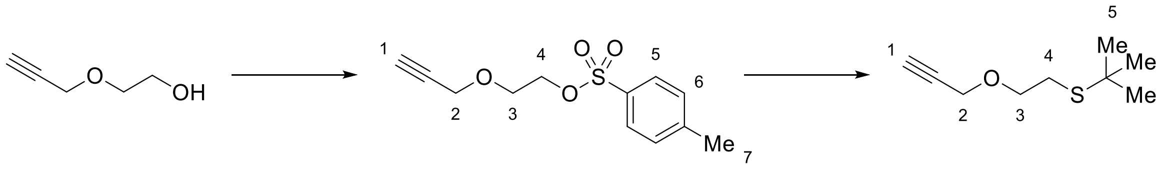

S24 **6c**

2-(Prop-2-yn-1-yloxy)ethyl 4-methylbenzenesulfonate (S24): Synthesised according to **general procedure K**, using 2-(prop-2-yn-1-yloxy)ethan-1-ol (1.20 g, 12.0 mmol) and diethyl ether (19.5 mL). Purification by column chromatography (7:3 Petrol:EtOAc) yielded title compound **S24** (2.90 g, 11.4 mmol, 95%) as a colourless oil.

δ_H (400 MHz, $CDCl_3$) 7.83-7.77 (2H, m, *Ar*), 7.38-7.31 (2H, m, *Ar*), 4.21-4.17 (2H, m, C(4)H_2), 4.12 (2H, d, *J* = 2.4 Hz, C(2)H_2), 3.75-3.70 (2H, m, C(3)H_2), 2.45 (3H, s, C(7)H_3), 2.42 (1H, t, *J* = 2.4 Hz, C(1)*H*); **δ_C** (101 MHz, $CDCl_3$) 144.8, 132.9, 129.8, 128.0, 78.9, 75.0, 68.8, 67.1, 58.3, 21.6; **ν_{max}**/ cm^{-1} (neat) 3654, 3283, 2980, 2885, 2118, 1597, 1495, 1449, 1352,

1307, 1291, 1243, 1211, 1189, 1173, 1095, 1017, 992, 958, 916; **m/z HRMS** (ESI^+) $[M + Na]^+$ $C_{12}H_{14}O_4{}^{23}NaS^+$ calc. 277.0505, found 277.0505. *Data is consistent with literature.*[19]

tert-Butyl(2-(prop-2-yn-1-yloxy)ethyl)sulfane (6c): Synthesised according to **general procedure N** using **S24** (2.54 g 10.0 mmol) and diethyl ether (25 mL), with stirring for 40 min. Purification by column chromatography (94:6 Hexane:EtOAc) yielded title compound **6c** (1.57 g, 9.11 mmol, 91%) as a colourless oil.

δ_H (400 MHz, $CDCl_3$) 4.18 (2H, d, *J* = 2.4 Hz, C(2)H_2), 3.68 (2H, t, *J* = 7.1 Hz, C(3)H_2), 2.76 (2H, t, *J* = 7.1 Hz, C(4)H_2), 2.43 (1H, t, *J* = 2.4 Hz, C(1)*H*), 1.33 (9H, s, 9H, C(5)H_3); **δ_C** (101 MHz, $CDCl_3$) 79.6, 74.7, 69.8, 58.2, 42.2, 31.1, 28.0; **ν_{max}**/ cm^{-1} (neat) 3291, 2960, 2898, 2863, 2116, 1607, 1459, 1390, 1364, 1290, 1270, 1162, 1090, 1045, 1011, 945, 908; **m/z HRMS** (ESI^+) $[M + H]^+$ $C_9H_{17}OS^+$ calc. 173.0995, found 173.0997.

2-(2-Iodophenyl)ethan-1-ol (S25), 2-(2-((trimethylsilyl)ethynyl)phenyl)ethan-1-ol (S26), 2-(2-ethynylphenyl)ethan-1-ol (S27), 2-ethynylphenethyl 4-methylbenzenesulfonate (S28) and *tert*-butyl(2-ethynylphenethyl)sulfane (6d)

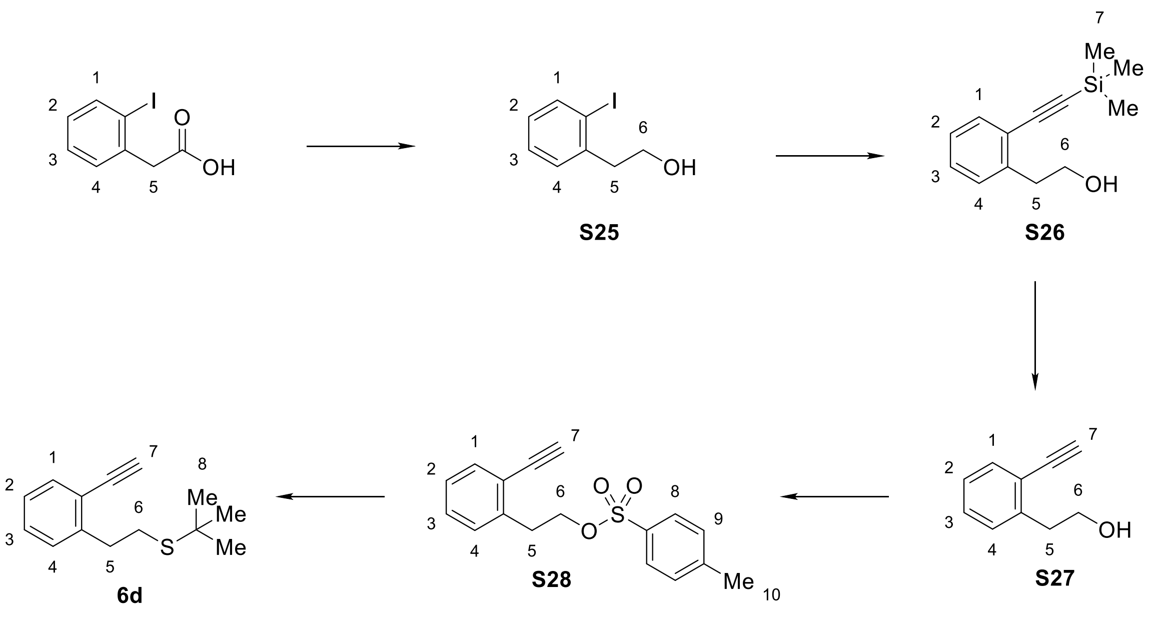

2-(2-Iodophenyl)ethan-1-ol (S25): According to a modified procedure,[46] 2-(2-iodophenyl)acetic acid (2.62 g, 10 mmol) was dissolved in THF (16 mL) and the solution cooled to 10 °C. $NaBH_4$ (757 mg , 20 mmol) was introduced followed by $BF_3{\cdot}OEt_2$ (2.5 mL, 20 mmol) and the solution stirred at 10 °C for 3 h. The reaction mixture was then allowed to warm to room temperature and left stirring overnight, unless otherwise stated. The reaction was then quenched by addition of water (20 mL) and MeOH (20 mL). The resulting mixture was extracted with Et_2O (4 × 30 mL) and washed with brine (20 mL). The combined organics were dried over anhydrous $MgSO_4$, filtered and concentrated *in vacuo*. Purification by column chromatography (7:3 Pentane:EtOAc) yielded title compound **S25** (2.36 g, 9.51 mmol, 95%) as an orange oil.

δ_H (400 MHz, $CDCl_3$) 7.90-7.70 (1H, m, *Ar*), 7.32-7.21 (2H, m, *Ar*), 6.95-6.86 (1H, m, *Ar*), 3.84 (2H, t, J = 6.8 Hz, C(6)H_2), 3.00 (2H, t, J = 6.8 Hz, C(5)H_2), 1.66 (bs, 1H, O*H*); **δ_C** (101 MHz, $CDCl_3$) 141.2, 139.8, 130.4, 128.4, 128.4, 100.9, 62.3, 43.8; **ν_{max}**/ cm^{-1} (neat) 3311, 2949, 2875, 1585, 1561, 1465, 1434, 1161, 1115, 1040, 1007; **m/z HRMS** (ESI^+) $[M + \text{radical ion}]^+$ $C_8H_9IO^+$ calc. 247.9693, found 247.9698. *Data is consistent with literature.*[46]

2-(2-((Trimethylsilyl)ethynyl)phenyl)ethan-1-ol (S26): According to a modified procedure,[47] compound **S25** (2.31 g, 9.3 mmol) was dissolved in anhydrous NEt_3 (27 mL). Trimethylsilyl acetylene (1.7 mL, 12.0 mmol), $PdCl_2(PPh_3)_2$ (0.130 g, 0.190 mmol) and CuI (7 mg, 9.30 mmol) were added to the solution and the mixture heated at 30 °C for 3 h, under an atmosphere of argon. Sat. aq. NH_4Cl (20 mL) was then added and the mixture extracted with CH_2Cl_2 (3 × 40 mL). The combined organics were washed with brine (20 mL), dried over anhydrous $MgSO_4$, filtered and concentrated *in vacuo*. Purification by column chromatography (3:2 Pentane:EtOAc) yielded title compound **S26** (1.56 g, 7.10 mmol, 76%) as a brown oil.

δ_H (400 MHz, $CDCl_3$) 7.49 (1H, dd, *J* = 7.6, 0.8 Hz, *Ar*), 7.32-7.23 (2H, m, *Ar*), 7.20 (1H, td, *J* = 7.3, 1.9 Hz, *Ar*), 3.94 (2H, t, *J* = 6.5 Hz C(6)H_2), 3.08 (2H, t, *J* = 6.5 Hz, C(5)H_2), 1.63 (1H, bs, O*H*), 0.28 (s, 9H, SiC(7)H_3); **δ_C** (101 MHz, $CDCl_3$) 141.0, 132.8, 129.7, 128.8, 126.5, 123.0, 103.8, 98.5, 62.9, 38.2, 0.1; **ν_{max}**/ cm^{-1} (neat) 3333, 2958, 2154, 1482, 1447, 1249, 1189, 1103, 1042, 946; **m/z HRMS** (ESI^+) $[M + H]^+$ $C_{13}H_{18}OSi^+$ calc. 219.1200, found 219.1201. *Data is consistent with literature.[47]*

2-(2-Ethynylphenyl)ethan-1-ol (S27): According to a modified procedure,[48] compound **S26** (1.04 g, 4.76 mmol) was dissolved in THF (11 mL). TBAF (1 M in THF, 5.95 mL, 5.95 mmol) was then introduced and the solution stirred at room temperature for 1.5 h. The mixture was diluted with water (20 mL) and extracted with CH_2Cl_2 (3 × 30 mL). The combined organics were washed with brine (20 mL), dried over anhydrous $MgSO_4$, filtered and concentrated *in vacuo*. Purification by column chromatography (1:1 Petrol:EtOAc) yielded title compound **S27** (482 mg, 3.29 mmol, 69%) as an orange oil.

δ_H (400 MHz, $CDCl_3$) 7.51 (1H, dd, *J* = 7.7, 1.4 Hz, *Ar*), 7.36-7.22 (2H, m, *Ar*), 7.20 (1H, td, *J* = 7.4, 1.7 Hz, *Ar*), 3.91 (2H, t, *J* = 6.7 Hz, C(6)H_2), 3.26 (1H, s, C(7)*H*), 3.08 (2H, t, *J* = 6.7 Hz, C(5)H_2); **δ_C** (101 MHz, $CDCl_3$) 141.1, 133.2, 129.8, 129.1, 126.6, 122.1, 82.3, 81.1, 63.0, 37.9; **ν_{max}**/ cm^{-1} (neat) 3289, 3065, 2933, 2879, 2102, 1599, 1483, 1446, 1244, 1161, 1101, 1040, 948; **m/z HRMS** (ESI^+) $[M + H]^+$ $C_{10}H_{11}O^+$ calc. 147.0804, found 147.0805. *Data is consistent with literature.[48]*

2-Ethynylphenethyl 4-methylbenzenesulfonate (S28): Synthesised according to **general procedure L**, using alkynol **S27** (480 mg, 3.29 mmol) and CH_2Cl_2 (13.2 mL). Purification by column chromatography (9:1 Petrol:EtOAc) yielded title compound **S28** (562 mg, 1.87 mmol, 57%) as an orange oil.

δ_H (400 MHz, $CDCl_3$) 7.73-7.65 (2H, m, *Ar*), 7.41 (1H, dd, *J* = 7.5, 1.5 Hz, *Ar*), 7.30-7.21 (3H, m, *Ar*), 7.22-7.13 (2H, m, 2H, *Ar*), 4.28 (2H, t, *J* = 7.0 Hz, C(6)H_2), 3.17 (1H, s, C(7)*H*), 3.14 (2H, t, *J* = 7.0 Hz, C(5)H_2), 2.43 (3H, s, C(10)H_3); **δ_C** (101 MHz, $CDCl_3$) 144.6, 138.7, 133.1, 133.1, 130.0, 129.8, 129.1, 127.9, 127.0, 122.0, 81.6, 81.5, 69.6, 34.2, 21.7; **ν_{max}**/ cm^{-1} (neat) 3279, 2960, 1598, 1484, 1448, 1355, 1306, 1292, 1188, 1173, 1096, 1055, 1037, 1019, 963, 903; **m/z LRMS** (ESI^+) 323.0 $[M + Na]^+$.

tert-Butyl(2-ethynylphenethyl)sulfane (6d): Synthesised according to **general procedure N**, using **S28** (542 mg, 3.70 mmol) and IPA (9.25 mL). Purification by column chromatography (9:1 Hexane:EtOAc) yielded title compound **6d** (464 mg, 2.12 mmol, 57%) as a pale-yellow oil.

δ_H (400 MHz, $CDCl_3$) 7.54-7.44 (1H, m, *Ar*), 7.33-7.22 (2H, m, *Ar*), 7.18 (1H, m, *Ar*), 3.27 (1H, s, C(7)*H*), 3.08-3.00 (m, 2H, C(5)H_2), 2.86-2.78 (2H, m, C(6)H_2), 1.34 (9H, s, C(8)H_3); **δ_C** (101 MHz, $CDCl_3$) 143.7, 133.1, 129.1, 129.1, 126.4, 121.6, 82.2, 81.1, 42.5, 35.6, 31.1, 29.0; **ν_{max}**/ cm^{-1} (neat) 3294, 2959, 2898, 2862, 2104, 1598, 1482, 1458, 1448, 1390, 1363, 1161, 1087, 1041, 947; **m/z HRMS** (ESI^+) $[M + H]^+$ $C_{14}H_{19}S^+$ calc. 219.2020, found 219.12042.

Pent-4-yn-1-yl 4-methylbenzenesulfonate (S29) *and tert*-butyl(pent-4-yn-1-yl)sulfide (6e)

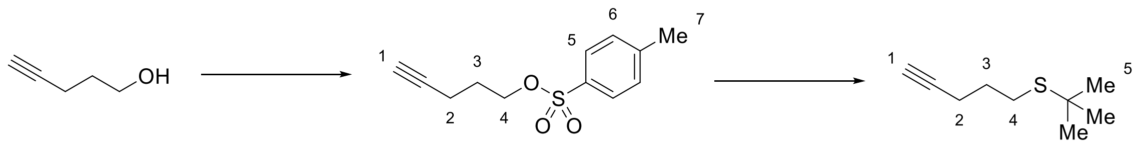

Pent-4-yn-1-yl 4-methylbenzenesulfonate (S29): Synthesised according to **general procedure K**, using pent-4-ynol (1.9 mL, 20.0 mmol) and diethyl ether (20 mL). Purification by column chromatography (4:1 Hexane:EtOAc) yielded title compound **6e** (4.79 g, 20 mmol, >99%) as a pale brown oil.

δ_H (400 MHz, $CDCl_3$) 7.86-7.73 (2H, m, *Ar*), 7.40-7.31 (2H, m, *Ar*), 4.15 (2H, t, *J* = 6.1 Hz, C(4)H_2), 2.45 (3H, s, C(7)H_3), 2.26 (2H, td, *J* = 6.9, 2.6 Hz, C(2)H_2), 1.92-1.81 (3H, m, C(1)*H* and C(3)H_2); **δ_C** (101 MHz,$CDCl_3$) 144.7, 133.0, 129.8, 127.9, 82.1, 69.4, 68.7, 27.7, 21.6, 14.7; **ν_{max}**/ cm^{-1} (neat) 3289, 3065, 2925, 2119, 1598, 1495, 1435, 1355, 1307, 1291, 1210, 1188, 1173, 1120, 1096, 1011, 973, 934, 920; **m/z HRMS** (ESI^+) $[M + Na]^+$ $C_{12}H_{14}O_3{}^{23}NaS^+$ calc. 261.05559, found 261.05561. *Data is consistent with literature.*[49]

tert-Butyl(pent-4-yn-1-yl)sulfide (6e): Synthesised according to **general procedure N**, using pent-4-yn-1-yl 4-methylbenzenesulfonate **S29** (700 mg, 3.03 mmol) and IPA (7.5 mL), with heating for 30 min. Purification by column chromatography (100% Hexane) yielded title compound **6e** (240 mg, 1.51 mmol, 51%) as a yellow oil.

δ_H (400 MHz, $CDCl_3$) 2.64 (2H, t, *J* = 7.3 Hz, C(4)H_2), 2.32 (2H, dd, *J* = 6.9, 2.7 Hz, C(2)H_2), 1.97 (1H, t, *J* = 2.7 Hz, C(1)*H*), 1.83-1.76 (2H, m, C(3)H_2), 1.33 (9H, s, C(5)H_3); **δ_C** (101 MHz, $CDCl_3$) 83.7, 68.9, 42.2, 31.1, 28.8, 27.3, 17.9; **ν_{max}**/ cm^{-1} (neat) 3301, 2959, 2941, 2926, 2863, 2118, 1472, 1459, 1431, 1390,1364, 1346, 1326, 1281, 1258, 1163, 1024, 932; **m/z HRMS** (ESI^+) $[M + H]^+$ $C_9H_{17}S^+$ calc.157.1045, found 157.1047.

2-(But-3-yn-2-yloxy)ethyl 4-methylbenzenesulfonate (S30) and (2-(but-3-yn-2-yloxy)ethyl)(*tert*-butyl)sulfane (6f)

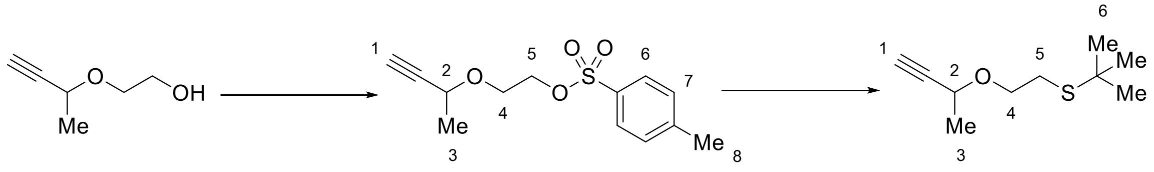

2c **S30** **6f**

2-(But-3-yn-2-yloxy)ethyl 4-methylbenzenesulfonate (S30): Synthesised according to **general procedure L**, using alkynol **2c** (200 mg, 1.75 mmol) and CH_2Cl_2 (7 mL), with stirring for 3.5 h. Purification by column chromatography (8:2 Petrol:EtOAc) yielded title compound **S30** (367 mg, 1.37 mmol, 78%) as a pale-yellow oil.

δ_H (400 MHz, $CDCl_3$) 7.84-7.77 (2H, m, *Ar*), 7.38-7.30 (2H, m, *Ar*), 4.26-4.07 (3H, m, C(5)H_2 and C(2)*H*), 3.91-3.83 (1H, m, C(4)*H*H), 3.66-3.53 (1H, m, C(4)H*H*), 2.44 (3H, s, C(8)H_3), 2.40 (1H, d, *J* = 2.0 Hz, C(1)*H*), 1.37 (3H, d, *J* = 6.6 Hz, C(3)H_3); **δ_C** (101 MHz, $CDCl_3$) 144.9, 133.2, 129.9, 128.1, 83.0, 73.6, 69.2, 66.0, 65.6, 21.9, 21.7; **ν_{max}**/ cm^{-1} (neat) 3282, 2988, 2936, 2871, 2109, 1740, 1598,1495, 1450, 1400, 1354, 1291, 1241, 1211, 1189, 1174, 1146, 1118, 1096, 1016, 955, 917; **m/z HRMS** (ESI^+) $[M + Na]^+$ $C_{13}H_{16}O_4{}^{23}NaS^+$ calc. 291.0662, found 291.0661.

(2-(But-3-yn-2-yloxy)ethyl)(tert-butyl)sulfane (6f): Synthesised according to **general procedure N**, using **S30** (360 mg, 1.34 mmol) and IPA (3.35 mL), with stirring for 1 h. Purification by column chromatography (98:2 Pentane:Et_2O) yielded title compound **6f** (181 mg, 0.970 mmol, 72%) as a colourless oil.

δ_H (400 MHz, $CDCl_3$) 4.20 (1H, qd, *J* = 6.6, 2.0 Hz, C(2)*H*), 3.90-3.81 (1H, m, C(4)*H*H), 3.59-3.47 (1H, m, C(4)H*H*), 2.75 (2H, t, *J* = 7.4 Hz, C(5)H_2), 2.42 (1H, d, *J* = 2.0 Hz, C(1)*H*), 1.45 (3H, d, *J* = 6.6 Hz, C(3)H_3), 1.33 (9H, s, C(6)H_3); **δ_C** (101 MHz, $CDCl_3$) 83.7, 73.1, 68.7, 65.3,

42.2, 31.1, 28.1, 22.1; **ν_{max}**/ cm^{-1} (neat) 3293, 2961, 2939, 2899, 2864, 2108, 1459, 1390, 1364, 1326, 1290, 1163, 1101, 1071, 1025, 926, 908; **m/z HRMS** (ESI^+) $[M + H]^+$ $C_{10}H_{19}OS^+$ calc.187.1151, found 187.1154.

2-((1-Phenylprop-2-yn-1-yl)oxy)ethyl 4-methylbenzenesulfonate (S31) and *tert*-butyl(2-((1-phenylprop-2-yn-1-yl)oxy)ethyl)sulfane (6g)

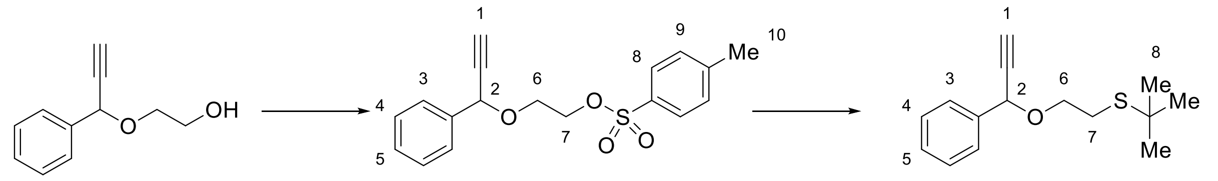

2-((1-Phenylprop-2-yn-1-yl)oxy)ethyl 4-methylbenzenesulfonate (S31): Synthesised according to **general procedure K**, using **2b** (270 mg, 1.53 mmol) and 2.5 mL, with stirring over 3 h. Purification by column chromatography (9:1 to 8:2 Petrol:EtOAc) yielded title compound **S31** (306 mg, 0.925 mmol, 60%) as a yellow oil.

δ_H (400 MHz, $CDCl_3$) 7.80-7.75 (2H, m, *Ar*), 7.46-7.42 (2H, m, *Ar*), 7.39-7.33 (3H, m, *Ar*), 7.32-7.24 (2H, m, *Ar*), 5.18 (1H, d, *J* = 2.2 Hz, C(2)*H*), 4.29-4.14 (2H, m, C(7)H_2), 3.87-3.79 (1H, m, C(6)*H*H), 3.78-3.67 (1H, m, C(6)H*H*), 2.64 (1H, d, *J* = 2.2 Hz, C(1)*H*), 2.42 (3H, s, C(10)H_3); **δ_C** (101 MHz, $CDCl_3$) 144.8, 137.5, 133.1, 129.9, 128.8, 128.6, 128.1, 127.4, 80.8, 76.4, 71.7, 69.1, 65.5, 21.7; **ν_{max}**/ cm^{-1} (neat) 3282, 3064, 2924, 2871, 2115, 1722, 1684, 1597, 1494, 1452, 1400, 1354, 1307, 1273, 1210, 1189, 1174, 1095, 1011, 959, 918; **m/z HRMS** (ESI^+) $[M + Na]^+$ $C_{18}H_{18}O_4{}^{23}NaS^+$ calc. 353.0818, found 353.0817.

tert-Butyl(2-((1-phenylprop-2-yn-1-yl)oxy)ethyl)sulfane (6g): Synthesised according to **general procedure M**, using alkyne **S31** (300 mg, 0.900 mmol) and THF (3 mL). Purification

by column chromatography (9:1 to 7:3 Hexane:EtOAc) yielded title compound **6g** (141 mg, 0.568 mmol, 63%) as a yellow oil.

δ_H (400 MHz, $CDCl_3$) 7.55-7.49 (2H, m, *Ar*), 7.41-7.29 (3H, m, *Ar*), 5.23 (1H, d, *J* = 2.2 Hz, C(2)*H*), 3.84-3.75 (1H, m, C(6)*H*H), 3.70-3.59 (1H, m, C(6)*H*H), 2.82-2.74 (2H, m, C(7)H_2), 2.65 (1H, d, *J* = 2.2 Hz, C(1)*H*), 1.31 (9H, s, C(8)H_3); **δ_C** (101 MHz, $CDCl_3$) 138.0, 128.6, 127.5, 81.5, 75.9, 71.5, 68.2, 42.3, 31.1, 28.1; **ν_{max}**/ cm^{-1} (neat) 3289, 3063, 3032, 2960, 2925, 2898, 2863, 2113, 1719, 1602, 1493, 1454, 1390, 1364, 1272, 1189, 1162, 1086, 1070, 1029, 1006, 940, 916; **m/z HRMS** (ESI^+) $[M + Na]^+$ $C_{15}H_{20}O^{23}NaS^+$ calc. 271.1127, found 271.1127.

***tert*-Butyl(3,3-dimethyl-2-(prop-2-yn-1-yloxy)butyl)sulfane (6h)**

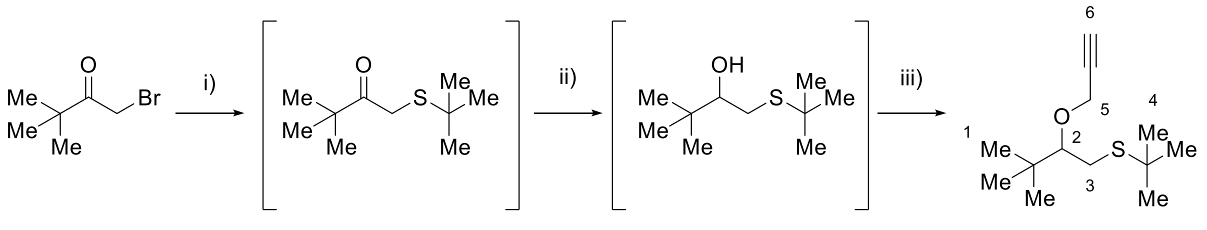

6h

Steps i) and ii): synthesised according to **general procedures Q** and **R**, using 1-bromo-3,3-dimethylbutan-2-one (895 mg, 5.00 mmol), THF (6 mL) and ethanol (12.5 mL), affording a colourless oil. **Step iii):** synthesised according to **general procedure O** using THF (8 mL) with stirring for 16 h. Purification by column chromatography (100:0 to 98:2 Pentane:Et_2O) yielded title compound **6h** (381 mg, 1.67 mmol, 33% over 3 steps) as a bright yellow oil.

δ_H (400 MHz, $CDCl_3$) 4.45 (1H, dd, *J* = 15.4, 2.4 Hz, C(5)*H*H), 4.32 (1H, dd, *J* = 15.5, 2.4 Hz, C(5)H*H*), 3.17 (1H, dd, *J* = 8.0, 3.1 Hz, C(2)*H*), 2.78 (1H, dd, *J* = 12.8, 3.0 Hz, C(3)*H*H), 2.61 (1H, dd, *J* = 12.8, 8.0 Hz, C(3)*H*H), 2.40 (1H, t, *J* = 2.4 Hz, C(6)*H*), 1.32 (9H, s, C(1)H_3), 0.95 (9H, s, C(4)H_3); **δ_C** (101 MHz, $CDCl_3$) 87.3, 80.7, 73.9, 59.9, 42.5, 36.1, 30.9, 30.2, 26.4; **ν_{max}**/ cm^{-1} (neat) 3312, 2958, 2900, 2865, 1478, 1459, 1393, 1363, 1337, 1259, 1218, 1162,

1081, 1055, 1008, 982, 963, 933,903; **m/z HRMS** (ESI$^+$) [M + Na]$^+$ $C_{13}H_{24}O^{23}NaS^+$ calc. 251.1451, found 251.1441.

***tert*-Butyl(2-cyclohexyl-2-(prop-2-yn-1-yloxy)ethyl)sulfane (6i)**

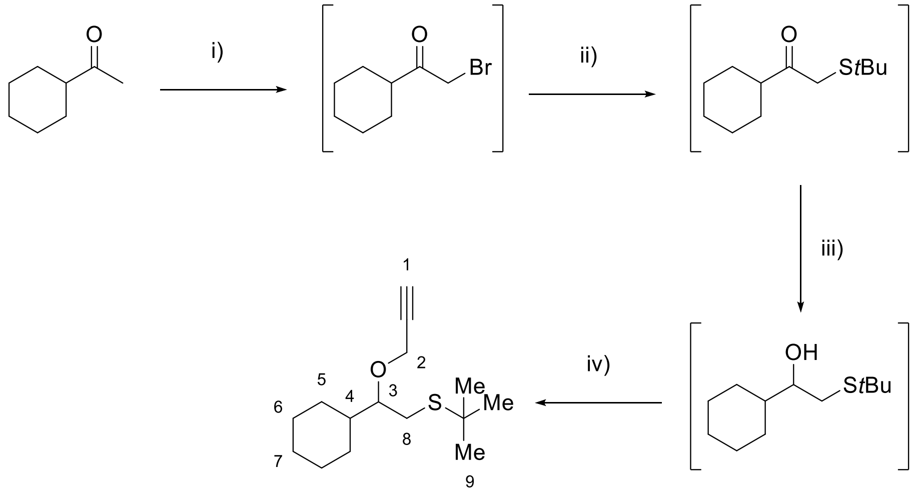

6i

Step i) According to a modified procedure,[50] to a pre-dried flask was added, acetylcyclohexane (1.2 mL, 10.0 mmol) and MeOH (7.5 mL). The solution was then cooled to 0 °C and bromine (0.52 mL, 10.0 mmol) was added in one portion. The mixture was then stirred at the same temperature for 7 h, after which water was introduced (10 mL). The solution was then stirred overnight at room temperature under a nitrogen atmosphere. To the resulting mixture was added Et_2O (20 mL) and brine (10 mL). The organic layer was then separated and washed with sat. aq. $NaHCO_3$ (10 mL) and brine (10 ml). The organics were dried over anhydrous $MgSO_4$, filtered and concentrated *in vacuo*, yielding a colourless oil. The crude intermediate was used in the next step withour further purification. **Steps ii) and iii** synthesised according to **general procedures Q** and **R** using THF (14 mL) and ethanol (9 mL) respectively. **Step iv)** synthesised according to **general procedure O** using THF (8 mL), with stirring for 16 h. Purification by column chromatography (100:0 to 98:2 Pentane:Et_2O) yielded title compound **6i** (678 mg, 2.66 mmol, 27%, over 4 steps) as a bright yellow oil.

δ_H (400 MHz, $CDCl_3$) 4.25 (2H, dd, *J* = 6.3, 2.4 Hz, C(2)H_2), 3.42 (1H, q, *J* = 5.6 Hz, C(3)*H*), 2.72 (2H, dd, *J* = 5.8, 0.9 Hz, C(8)H_2), 2.40 (1H, t, *J* = 2.4 Hz, C(1)*H*), 1.83-1.56 (5H, m, C(5 to 7)H_2 and C(4)*H*), 1.37-0.99 (15H, m, C(9)H_3 and C(5 to 7)H_2); **δ_C** (101 MHz, $CDCl_3$) 82.8, 80.6, 74.0, 57.7, 42.1, 40.9, 31.0, 29.5, 29.4, 27.8, 26.6, 26.4, 26.3; **ν_{max}**/ cm^{-1} (neat) 3310, 2972, 2959, 2924, 2852, 1448, 1391, 1363, 1339, 1265, 1162, 1071, 1035, 1016, 970, 936, 910; **m/z HRMS** (ESI^+) $[M + Na]^+$ $C_{15}H_{27}O^{23}NaS^+$ calc. 277.1597, found 277.1598.

***tert*-Butyl(3-phenyl-2-(prop-2-yn-1-yloxy)propyl)sulfane (6j)**

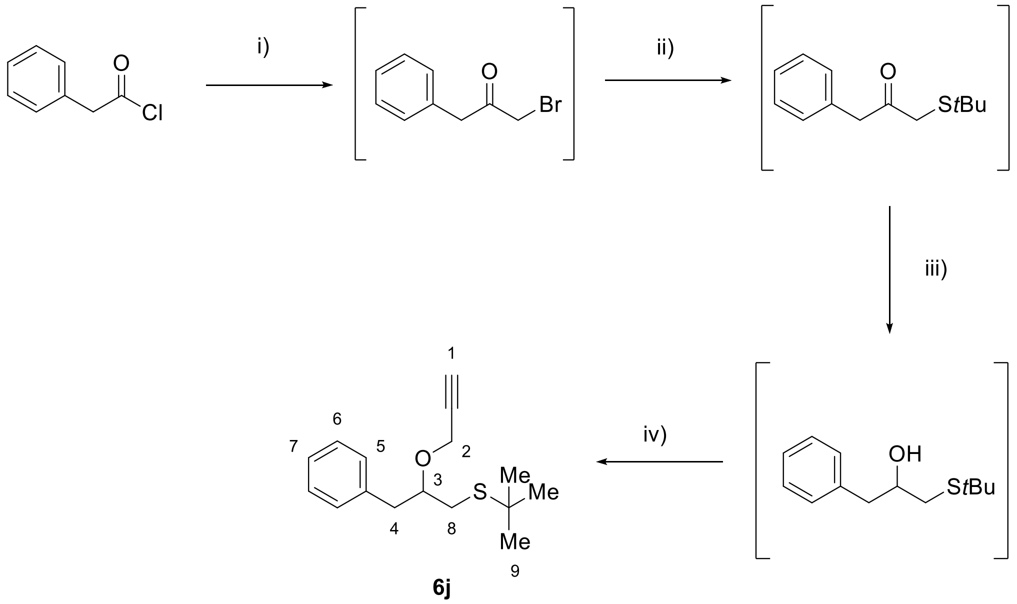

Step i) According to a modified procedure,[51] 2-phenylacetyl chloride (773 mg, 5.00 mmol) was dissolved in anhydrous acetonitrile (50 mL). The solution was then cooled to 0 °C and trimethylsilyl diazomethane (2 M in hexanes, 3.75 mL, 3.4 mmol) was added dropwise under an argon atmosphere. The mixture was then warmed to room temperature and stirred for 1 h, after which $HBr_{(aq)}$ (48% w/w, 3.2 mL) was added dropwise at 0 °C. The mixture was then warmed to room temperature and stirred for 2 h. The solution was next diluted with ice water (100 mL) and then extracted with EtOAc (3 × 20 mL). The combined organics were dried over anhydrous $MgSO_4$, filtered and then concentrated *in vacuo*. The crude residue was then used

in the next step without further purification. **Steps ii) and iii)** Synthesised according to **general procedures P** and **R** using ethanol (8 mL) in both steps. **Step iv)** synthesised according to **general procedure O** using THF (8 mL), with stirring for 16 h. Purification by column chromatography (100:0 to 98:2 Pentane:Et_2O) yielded title compound **6j** (822 mg, 3.13 mmol, 63% over 4 steps) as a yellow oil.

δ_H (400 MHz, $CDCl_3$) 7.32-7.24 (4H, m, *Ar*), 7.24-7.18 (1H, m, *Ar*), 4.16 (2H, dd, *J* = 6.1, 2.4 Hz, C(2)H_2), 3.93-3.84 (1H, m, C(3)*H*), 2.97 (1H, dd, *J* = 13.8, 5.6 Hz, C(4)*H*H), 2.88 (1H, dd, *J* = 13.9, 6.6 Hz, C(4)H*H*), 2.72 (1H, dd, *J* = 12.5, 5.2 Hz, C(8)*H*H), 2.63 (1H, dd, *J* = 12.5, 6.6 Hz, C(8)H*H*), 2.38 (1H, t, *J* = 2.4 Hz, C(1)*H*), 1.30 (9H, s, C(9)H_3); **δ_C** (101 MHz, $CDCl_3$) 138.3, 129.7, 128.4, 126.4, 80.1, 79.7, 74.3, 57.1, 42.3, 40.0, 31.8, 31.0; **ν_{max}**/ cm^{-1} (neat) 3306, 3290, 3086, 3062, 3028, 2972, 2959, 2923, 2898, 2861, 2115, 1646, 1603,1495, 1456, 1391, 1364, 1341, 1295, 1268, 1213, 1162, 1091, 1077, 1065, 1029, 1009, 915; **m/z HRMS** (ESI^+) $[M + Na]^+$ $C_{16}H_{22}{}^{23}NaOS^+$ calc. 285.1284, found 285.1285.

***tert*-Butyl(2-(prop-2-yn-1-yloxy)butyl)sulfane (6k)**

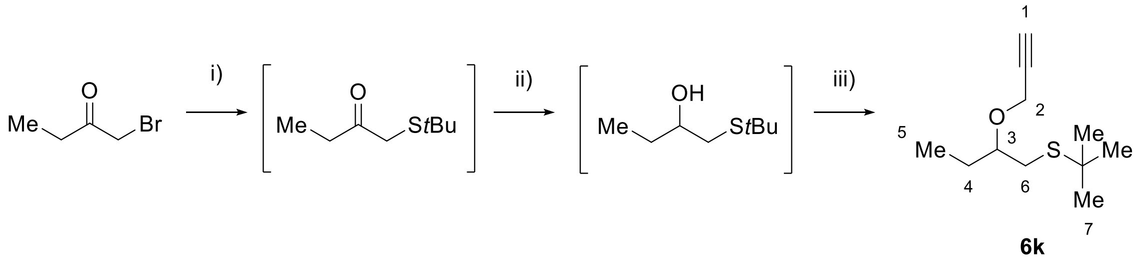

6k

Steps i) and **ii):** synthesised according to **general procedures Q** and **R**, using 1-bromobutan-2-one (0.51 mL, 5.00 mmol), THF (8 mL) and ethanol (12 mL), affording a colourless oil. **Step iii):** synthesised according to **general procedure O** using THF (6 mL), with stirring for 18 h. Purification by column chromatography (100:0 to 98:2 Petrol:Et_2O) yielded title compound **6k** (85.3 mg, 0.43 mmol, 8% over 3 steps) as a bright yellow oil.

δ_H (400 MHz, $CDCl_3$) 4.24 (2H, d, J = 2.4 Hz, C(2)H_2), 3.62-3.56 (1H, m, C(3)H), 2.75 (1H, dd, J = 12.4, 5.2 Hz, C(6)HH), 2.63 (1H, dd, J = 12.4, 6.9 Hz, C(6)HH), 2.41 (1H, t, J = 2.4 Hz, C(1)H), 1.75-1.65 (1H, m, C(4)HH), 1.62-1.53 (1H, m, C(4)HH), 1.33 (9H, s, C(7)H_3), 0.95 (3H, t, J = 7.1 Hz, C(5)H_3); δ_C (101 MHz, $CDCl_3$) 80.4, 79.6, 74.1, 56.8, 42.1, 31.6, 31.0, 26.4, 9.4; **ν_{max}**/ cm^{-1} (neat) 3310, 2960, 2924, 2860, 2116, 1459, 1364, 1342, 1259, 1234, 1163, 1082, 1012, 940, 917; **m/z HRMS** (ESI^+) $[M + Na]^+$ $C_{11}H_{20}O^{23}NaS^+$ calc. 223.1127, found 223.1129.

(5-Oxo-1-(prop-2-yn-1-yl)pyrrolidin-2-yl)methyl 4-methylbenzenesulfonate (S32) and 5-((*tert*-Butylthio)methyl)-1-(prop-2-yn-1-yl)pyrrolidin-2-one (6l)

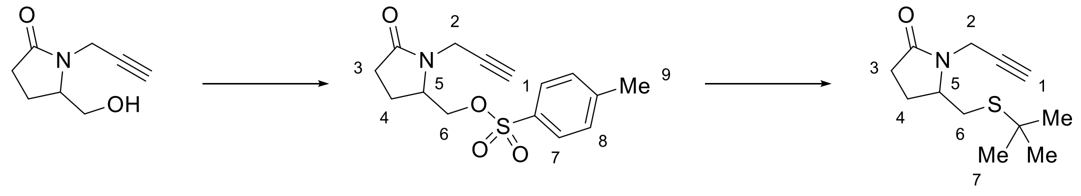

(5-Oxo-1-(prop-2-yn-1-yl)pyrrolidin-2-yl)methyl 4-methylbenzenesulfonate (S32): Synthesised according to **general procedure K**, using alkynol **2n** (0.54 g, 3.50 mmol) and diethyl ether (6 mL), with stirring for 2 h. Purification by column chromatography (1:1 to 7:3 EtOAc:Petrol) yielded title compound **S32** (926 mg, 3.07 mmol, 86%) as white crystals.

m.p. 64–65 °C; δ_H (400 MHz, $CDCl_3$) 7.83-7.75 (2H, m, *Ar*), 7.40-7.34 (2H, m, *Ar*), 4.47 (1H, dd, J = 17.8, 2.6 Hz, C(2)HH), 4.21 (1H, dd, J = 10.7, 3.7 Hz, C(6)HH), 4.11 (1H, dd, J = 10.7, 3.6 Hz, C(6)HH), 4.05-3.99 (1H, m, C(5)H), 3.51 (1H, ddd, J = 17.9, 2.5, 1.1 Hz, C(2)HH), 2.50-2.39 (4H, m, C(9)H_3 and C(3)HH), 2.38-2.28 (1H, m, C(3)HH), 2.26-2.11 (2H, m, C(1)H and C(4)HH), 1.95-1.83 (1H, m, C(4)HH); δ_C (151 MHz, $CDCl_3$) 174.8, 145.5, 132.6, 130.2, 128.1, 72.9, 68.8, 55.8, 30.4, 29.8, 21.8, 21.1; **ν_{max}**/ cm^{-1} (neat) 3281, 2955, 2364, 2118, 1688,

1597, 1494, 1440, 1415, 1358, 1307, 1293, 1279, 1253, 1211, 1189, 1174, 1120, 1096, 1048, 1019, 991, 950; **m/z HRMS** (ESI$^+$) [M + H]$^+$ $C_{15}H_{17}NO_4S^+$ calc. 308.0951, found 308.0952.

5-((tert-Butylthio)methyl)-1-(prop-2-yn-1-yl)pyrrolidin-2-one (6l): According to a modified procedure,[41] to a pre-dried flask was added powdered KOH (300 mg, 5.34 mmol, 2.0 equiv.), anhydrous acetonitrile (4.0 mL) and *tert*-butylthiol (0.45 mL, 4.0 mmol 1.5 equiv.). The suspension was stirred for 10 min, after which **S32** (820 mg, 2.67 mmol, 1.0 equiv) dissolved in acetonitrile (8.0 mL) was added to the suspension. The mixture was then stirred at room temperature under a nitrogen atmosphere for 16 h. The mixture was next diluted with ice water (20 mL) and then extracted with EtOAc (3 × 20 mL). The combined organics were dried over anhydrous $MgSO_4$, filtered and then concentrated *in vacuo*. Purification by column chromatography (7:3 to 6:4 Petrol:EtOAc) yielded title compound **6l** (522 mg, 2.32 mmol, 87%) as a colourless oil.

δ_H (400 MHz, $CDCl_3$) 4.59 (1H, dd, *J* = 17.7, 2.6 Hz, C(2)*H*H), 4.05-3.95 (1H, m, C(5)*H*), 3.74 (1H, ddd, *J* = 17.8, 2.5, 1.1 Hz, C(2)H*H*), 2.90 (1H, dd, *J* = 12.3, 3.4 Hz, C(6)*H*H), 2.66 (1H, dd, *J* = 12.3, 8.1 Hz, C(6)*H*H), 2.52-2.42 (1H, m, C(3)*H*H), 2.41-2.29 (1H, m, C(3)H*H*), 2.29-2.16 (2H, m, C(1)*H* and C(4)*H*H), 1.97-1.84 (1H, m, C(4)H*H*), 1.34 (9H, s, C(7)H_3); **δ_C** (101 MHz, $CDCl_3$) 174.7, 77.8, 72.4, 56.9, 42.4, 31.7, 31.0, 30.3, 30.0, 23.6; **ν_{max}**/ cm^{-1} (neat) 3310, 2960, 2926, 2901, 2865, 1748, 1696, 1459, 1393, 1364, 1338, 1287, 1229, 1163, 1021; **m/z HRMS** (ESI$^+$) [M + H]$^+$ $C_{12}H_{20}NOS^+$ calc. 226.1260, found 226.1262.

2-(*tert*-Butylthio)-1-cyclopropylethan-1-ol (S33) and *tert*-butyl(2-cyclopropyl-2-(prop-2-yn-1-yloxy)ethyl)sulfane (6m)

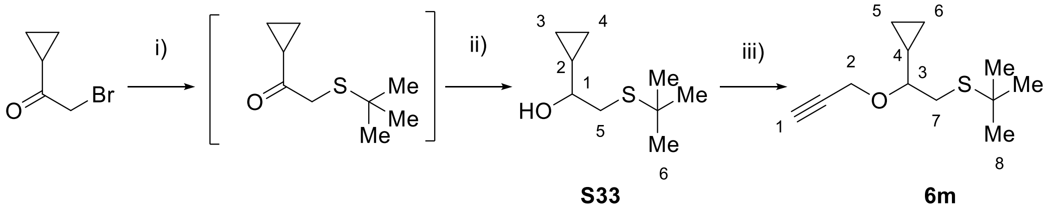

***2-(tert-Butylthio)-1-cyclopropylethan-1-ol (S33)*:** **Steps i)** and **ii)**: Synthesised according to **general procedures Q** and **R**, using 2-bromo-1-cyclopropylethan-1-one (815 mg, 5.00 mmol), THF (12 mL) and ethanol (12 mL). Purification by column chromatography (9:1 Pentane:Et_2O) yielded title compound **S33** (714 mg, 4.09 mmol, 82%) as a yellow oil.

δ_H (400 MHz, $CDCl_3$) 2.99 (1H, td, *J* = 8.4, 3.6 Hz, C(1)*H*), 2.91 (1H, dd, *J* = 12.7, 3.6 Hz, 1H, C(5)*H*H), 2.68 (1H, dd, *J* = 12.7, 8.6 Hz, C(5)H*H*), 2.27 (1H, bs, O*H*), 1.33 (9H, s, C(6)H_3), 0.89-0.98 (1H, m, C(2)*H*), 0.59-0.49 (2H, m, C(3 and 4)H_2), 0.41-0.36 (1H, m, C(3 and 4)H_2), 0.27-0.22 (1H, m, C(3 and 4)H_2); **δ_C** (101 MHz, $CDCl_3$) 74.9, 42.5, 36.5, 31.2, 16.8, 3.0, 2.3; **ν_{max}**/ cm^{-1} (neat) 3419, 3080, 3004, 2960, 2924, 2899, 2863, 2361, 1652, 1459, 1429, 1391, 1364, 1320, 1261, 1162, 1117, 1044, 1021, 917; **m/z HRMS** (ESI^+) [M + radical ion]$^+$ $C_9H_{18}OS^+$ calc. 174.1078, found 174.1073.

***tert-Butyl(2-cyclopropyl-2-(prop-2-yn-1-yloxy)ethyl)sulfane (6m)*:** **Step iii):** synthesised according to **general procedure O**, using alcohol **S33** (872 mg, 5.00 mmol) and THF (8 mL), with stirring for 16 h. Purification by column chromatography (98:2 to 94:6 Pentane:Et_2O) yielded title compound **6m** (771 mg, 3.63 mmol, 73%) as a pale-yellow oil.

δ_H (400 MHz, $CDCl_3$) 4.34 (2H, dd, *J* = 6.5, 2.4 Hz, C(2)H_2), 3.07 (1H, dt, *J* = 8.6, 5.8 Hz, C(3)*H*), 2.82 (2H, d, *J* = 5.8 Hz, C(7)H_2), 2.39 (1H, t, *J* = 2.4 Hz, C(1)*H*), 1.33 (9H, s, C(8)H_3), 0.97-0.88 (1H, m, C(4)*H*), 0.65-0.59 (1H, m, C(5 and 6)H_2), 0.56-0.47 (2H, m, C(5 and 6)H_2),

0.25-0.19 (1H, m, C(5 and 6)H_2); **δ_C** (101 MHz, $CDCl_3$) 81.7, 80.2, 74.3, 56.1, 41.9, 33.7, 31.0, 14.2, 4.4, 1.4; **ν_{max}**/ cm^{-1} (neat) 3309, 3081, 3005, 2972, 2959, 2923, 2898, 2862, 2114, 1473, 1459, 1443, 1392, 1364, 1326, 1295, 1267, 1211, 1162, 1122, 1095, 1075, 1023, 972, 931, 916; **m/z HRMS** (ESI^+) $[M + Na]^+$ $C_{12}H_{20}O^{23}NaS^+$ calc. 235.1127, found 235.1129.

tert-Butyl(2-phenyl-2-(prop-2-yn-1-yloxy)ethyl)sulfane (6n)

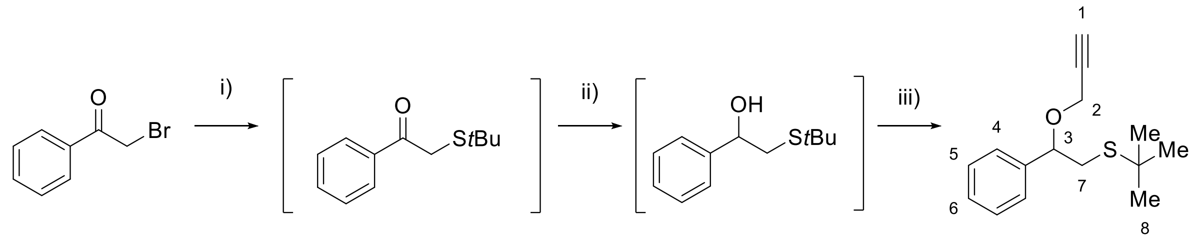

6n

Steps i) and **ii)**: synthesised according to **general procedure Q** and **R,** using 2-bromoacetophenone (990 mg, 5.00 mmol), THF (4 mL) and ethanol (4.5 mL). **Step iii)** synthesised according to **general procedure O** using THF (4 mL) with stirring for 16 h**.** Purification by column chromatography (100:0 to 98:2 Hexane:Et_2O) yielded title compound **6n** (611 mg, 2.46 mmol, 49% over three steps) as a bright yellow-orange oil.

δ_H (400 MHz, $CDCl_3$) 7.41-7.29 (5H, m, *Ar*), 4.66 (1H, dd, *J* = 7.7, 5.9 Hz, OC(3)*H*), 4.15 (dd, *J* = 15.8, 2.4 Hz, C(2)*H*H), 3.89 (1H, dd, *J* = 15.8, 2.4 Hz, C(2)H*H*), 3.01 (1H, dd, *J* = 12.4, 7.7 Hz, C(7)*H*H), 2.80 (1H, dd, *J* = 12.4, 5.9 Hz, C(7)H*H*), 2.41 (1H, t, *J* = 2.4 Hz, C(1)*H*), 1.30 (9H, s, C(8)H_3); **δ_C** (101 MHz, $CDCl_3$) 140.0, 128.7, 128.5, 127.2, 80.5, 79.7, 74.6, 55.9, 42.3, 35.8, 31.0; **ν_{max}**/ cm^{-1} (neat) 3307, 3287, 3062, 3030, 2972, 2958, 2923, 2898, 2860, 2115, 1602, 1492, 1473, 1456, 1389, 1364, 1286, 1267, 1236, 1209, 1161, 1087, 1068, 1025, 1007, 961, 949, 918; **m/z HRMS** (ESI^+) $[M + Na]^+$ $C_{15}H_{20}O^{23}NaS^+$ calc. 271.1127, found 271.1129.

tert-Butyl(2-(naphthalen-2-yl)-2-(prop-2-yn-1-yloxy)ethyl)sulfane (6o)

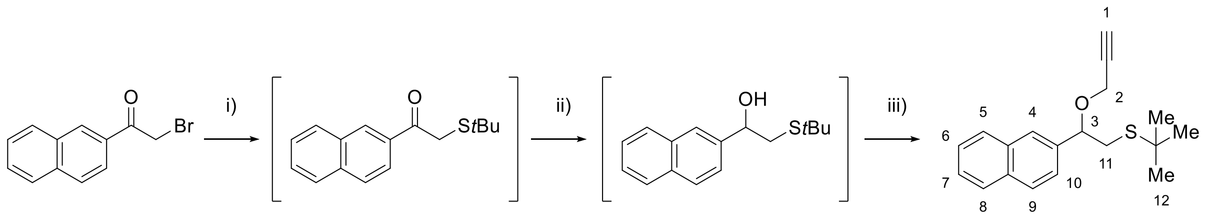

6o

Steps i) and **ii):** synthesised according to **general procedures Q** and **R**, using 2-bromo-2′-acetonaphthone (1.25 g, 5.00 mmol), THF (12 mL) and ethanol (12 mL), affording a yellow oil. **Step iii):** synthesised according to **general procedure O** using THF (8 mL), with stirring for 16 h. Purification by column chromatography (100:0 to 96:4 Pentane:Et_2O) yielded title compound **6o** (722 mg, 2.42 mmol, 48%) as a bright-yellow oil.

δ_H (400 MHz, $CDCl_3$) 7.88-7.80 (4H, m, *Ar*), 7.52-7.46 (3H, m, *Ar*), 4.83 (1H, dd, *J* = 7.5, 6.1 Hz, C(3)*H*), 4.18 (1H, dd, *J* = 15.8, 2.4 Hz, C(2)*H*H), 3.92 (1H, dd, *J* = 15.8, 2.4 Hz, C(2)H*H*), 3.10 (1H, dd, *J* = 12.4, 7.5 Hz, C(11)*H*H), 2.89 (1H, dd, *J* = 12.4, 6.1 Hz, C(11)H*H*), 2.43 (1H, t, *J* = 2.4 Hz, C(1)*H*), 1.31 (9H, s, C(12)H_3); **δ_C** (101 MHz, $CDCl_3$) 137.3, 133.5, 133.2, 128.7, 128.1, 127.9, 126.9, 126.4, 126.3, 124.4, 80.7, 79.6, 74.7, 56.0, 42.3, 35.7, 31.0; **ν_{max}**/ cm^{-1} (neat) 3291, 3055, 2959, 2924, 2897, 2861, 1601, 1508, 1458, 1442, 1364, 1317, 1270, 1162, 1123, 1072, 1016, 948, 916; **m/z HRMS** (ESI^+) $[M + Na]^+$ $C_{19}H_{23}O^{23}NaS^+$ calc. 321.1284, found 321.1284.

(2-(4-Bromophenyl)-2-(prop-2-yn-1-yloxy)ethyl)(*tert*-butyl)sulfane (6p)

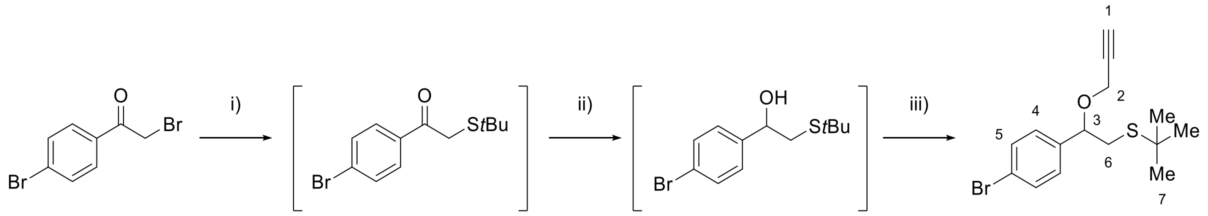

6p

Steps i) and **ii):** synthesised according to **general procedure Q** and **R**, using 2-bromo-1-(4-bromophenyl)ethan-1-one (1.39 g, 5.00 mmol), THF (8.5 mL) and ethanol (10 mL). **Step iii):** synthesised according to **general procedure O** using THF (4 mL), with stirring for 3.5 h. Purification by column chromatography (9:1 to 1:1 Petrol:EtOAc) yielded title compound **6p** (487 mg, 1.49 mmol, 30%) as a yellow oil.

δ_H (400 MHz, $CDCl_3$) 7.44-7.36 (2H, m, *Ar*), 7.20-7.08 (2H, m, *Ar*), 4.52 (1H, t, *J* = 6.8 Hz, C(3)*H*), 4.04 (1H, dd, *J* = 15.8, 2.4 Hz, C(2)*H*H), 3.79 (1H, dd, *J* = 15.8, 2.4 Hz, C(2)H*H*), 2.88 (1H, dd, *J* = 12.4, 7.2 Hz, C(6)*H*H), 2.66 (1H, dd, *J* = 12.4, 6.5 Hz, C(6)H*H*), 2.32 (1H, t, *J* = 2.4 Hz, C(1)*H*), 1.19 (9H, s, C(7)H_3); **δ_C** (101 MHz, $CDCl_3$) 139.1, 131.8, 129.0, 122.4, 79.9, 79.4, 74.9, 56.0, 42.4, 35.5, 31.0; **ν_{max}**/ cm^{-1} (neat) 3297, 2971, 2958, 2923, 2898, 2860, 2116, 1591, 1485, 1458, 1441, 1406, 1388, 1364, 1340, 1294, 1267, 1235, 1211, 1161, 1088, 1068, 1009, 956, 915; **m/z HRMS** (ESI^+) $[M + Na]^+$ $C_{15}H_{19}BrO^{23}NaS^+$ calc. 351.0211, found 351.0212.

2-(*tert*-Butylthio)-2-phenylethan-1-ol (S34) and *tert*-butyl(1-phenyl-2-(prop-2-yn-1-yloxy)ethyl)sulfane (6q)

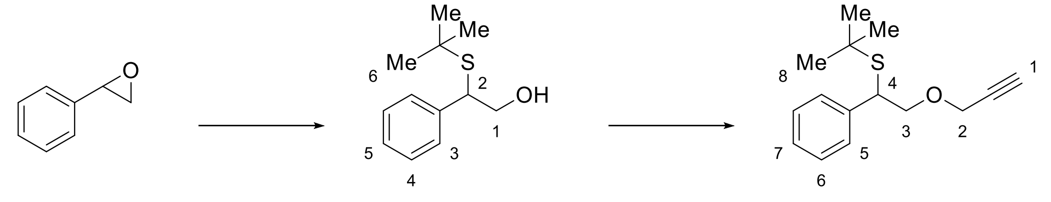

2-(tert-Butylthio)-2-phenylethan-1-ol (S34): According to a modified procedure,[52] to a pre-dried flask was added $InCl_3$ (221 mg, 1.0 mmol) was and CH_2Cl_2 (20 mL). The mixture was then cooled to 0 °C, after which styrene oxide (1.1 mL, 10.0 mmol) and *tert*-butylthiol (1.1 mL 10.0 mmol) were introduced. The solution was then stirred under a nitrogen atmosphere, at room temperature for 16 h. The mixture was then diluted with water (40 mL) and extracted with CH_2Cl_2 (3 × 20 mL). The combined organics were dried over anhydrous $MgSO_4$, and then concentrated *in vacuo*. Purification by column chromatography (9:1 Pentane:EtOAc) yielded title compound **S34** (760 mg, 3.63 mmol, 36%) as a pale-yellow oil.

δ_H (400 MHz, $CDCl_3$) 7.37-7.30 (4H, m, *Ar*), 7.27-7.22 (1H, m, *Ar*), 3.98 (1H, dd, *J* = 8.6, 6.4 Hz, C(2)*H*), 3.77 (1H, dd, *J* = 11.3, 6.4 Hz, C(1)*H*H), 3.63 (1H, dd, *J* = 11.3, 8.5 Hz, C(1)*H*H), 2.04 (1H, bs, O*H*), 1.30 (9H, s, C(6)H_3); **δ_C** (101 MHz, $CDCl_3$) 141.9, 128.8, 128.0, 127.4, 66.7, 51.1, 44.4, 31.6; **ν_{max}**/ cm^{-1} (neat) 3409, 3060, 3027, 2959, 2896, 2863, 1600, 1491, 1452, 1390, 1364, 1339, 1249, 1160, 1052, 1028, 977, 932, 908; **m/z HRMS** (ESI^+) $[M + Na]^+$ $C_{12}H_{18}O^{23}NaS^+$ calc. 233.0971, found 233.0973.

tert-Butyl(1-phenyl-2-(prop-2-yn-1-yloxy)ethyl)sulfane (6q): Synthesised according to **general procedure O**, using alcohol **S34** (740 mg, 3.5 mmol), NaH (280 mg, 7 mmol), propargyl bromide (0.67 mL, 7.00 mmol) and THF (4 mL). Stirred for 16 h. Purification by

column chromatography (100:0 to 98:2 Pentane:Et_2O) yielded title compound **6q** (630 mg 2.53 mmol, 72%) as a bright yellow oil.

δ_H (400 MHz, $CDCl_3$) 7.37-7.29 (4H, m, *Ar*), 7.25-7.20 (1H, m, *Ar*), 4.11 (2H, t, *J* = 2.6 Hz, C(2)H_2), 4.05 (1H, dd, *J* = 8.4, 6.2 Hz, C(4)*H*), 3.88-3.78 (2H, m, C(3)H_2), 2.40 (1H, t, *J* = 2.4 Hz, C(1)*H*), 1.29 (9H, s, C(8)H_3); **δ_C** (101 MHz, $CDCl_3$) 142.4, 128.6, 128.0, 127.2, 79.6, 74.7, 74.3, 58.3, 47.0, 44.3, 31.5; **ν_{max}**/ cm^{-1} (neat) 3286, 3061, 3028, 2959, 2896, 2860, 2116, 1601, 1583, 1492, 1470, 1452, 1391, 1363, 1266, 1236, 1160, 1097, 1080, 1029, 1003, 985, 948, 908; **m/z HRMS** (ESI^+) $[M + Na]^+$ $C_{15}H_{20}O^{23}NaS^+$ calc. 271.1127, found 271.1127.

7.3 Scope reaction procedures

The first step of all scope reactions was performed using the rhodium-catalysed hydroacylation **general procedure H** from section **6.1**. All reactions were performed on a 0.2 mmol scale, using $Rh(nbd)_2BF_4$ (3.7 mg, 0.010 mmol, 0.050 equiv.), dcpe (4.2 mg, 0.010 mmol, 0.050 equiv.) and DCE (0.2 mL), with aldehyde (1.0 equiv.) and alkyne (1.2-1.5 equiv.). This step was followed by **general procedure R**.

General procedure S: The crude hydroacylation product was diluted with DCE (1.8 mL) (in the same microwave vial) and TFA (1.0-10.0 equiv.) was introduced. The mixture was then stirred at 40 °C for a given amount of time, after which the solution was filtered through a pad of silica with EtOAc. The filtrate was then concentrated under reduced pressure.

7.4 *S*-heterocycle compounds

1-(2-(Methylthio)phenyl)-2-(tetrahydro-2*H*-thiopyran-2-yl)ethan-1-one (7a)

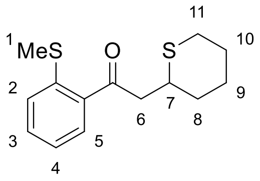

Synthesised according to **general procedures H** and **S**. **General procedure H** used 2-(methylthio)benzaldehyde **1a** (26 µL, 0.200 mmol, 1.0 equiv.) and *tert*-butyl(hex-5-yn-1-yl)sulfide **6b** (41 mg, 0.240 mmol, 1.20 equiv.). **General procedure S** used TFA (40 µL, 0.400 mmol), with stirring for 4 h. Purification by column chromatography (9:1 Pentane:Et_2O) yielded title compound **7a** (43.9 mg, 0.165 mmol, 82%) as a yellow oil.

Gram-Scale Reaction: Following **general procedures H** and **S**. **General procedure H** used 2-(methylthio)benzaldehyde **1a** (0.13 mL, 1.00 mmol, 1.00 equiv.) and *tert*-butyl(hex-5-yn-1-yl)sulfide **6b** (200 mg, 1.20 mmol, 1.20 equiv.), with $Rh(nbd)_2BF_4$ (18.7 mg, 0.05 mmol, 0.05 equiv.), dcpe (21.4 mg, 0.05 mmol, 0.05 equiv.) and DCE (1 mL) in a microwave vial. For **general procedure S**, a separate oven-dried round-bottom flask was backfilled under argon three times. The crude reaction mixture from **general procedure H** was diluted with DCE (19 mL) and transferred over to this flask *via* a syringe. TFA (0.2 mL, 2.00 mmol, 2.00 equiv.) was next introduced and the resulting solution was stirred for 6 h at 40 °C (before work-up according to **general procedure S**). Purification by column chromatography (column 92:8 Hexane:EtOAc then 9:1 Hexane:EtOAc), yielded title compound **7a** (130 mg, 0.50 mmol, 50%) as a yellow oil. This could be used in subsequent derivatisations (see SI, section **7.5**).

δ_H (400 MHz, $CDCl_3$) 7.80 (1H, dd, *J* = 7.8, 1.5 Hz, *Ar*), 7.49-7.45 (1H, m, *Ar*), 7.34-7.32 (1H, d, *J* = 8.0 Hz, *Ar*), 7.21-7.17 (1H, m, *Ar*), 3.44-3.37 (1H, m, C(7)*H*), 3.15-3.01 (2H, m, C(6)H_2),

2.79-2.72 (1H, m, C(11)*H*H), 2.63-2.57 (1H, m, C(11)H*H*), 2.43 (3H, s, C(1)H_3), 2.16-2.08 (1H, m, C(8)*H*H), 1.96-1.90 (1H, m, C(8)*H*H), 1.87-1.79 (1H, m, C(9)*H*H), 1.66-1.55 (m, 1H, C(10)H*H*), 1.50-1.41 (2H, m, C(8)H*H* and C(9)H*H*); **δ_C** (101 MHz, $CDCl_3$) 198.8, 142.6, 134.6, 132.4, 130.2, 125.3, 123.6, 46.0, 37.9, 34.4, 29.5, 27.0, 25.9, 16.1; **ν_{max}**/ cm^{-1} (neat) 2921, 2851, 2360, 1671, 1586, 1558, 1459, 1433, 1361, 1342, 1279, 1213, 1177, 1139, 1080, 1045, 1017, 984, 924; **m/z HRMS** (ESI^+) $[M + Na]^+$ $C_{14}H_{18}O^{23}NaS_2^+$ calc. 289.0691, found 289.0692.

1-(4-Bromo-2-(methylthio)phenyl)-2-(tetrahydro-2*H*-thiopyran-2-yl)ethan-1-one (7b)

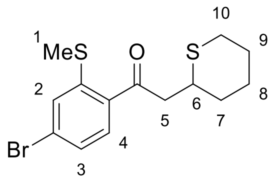

Synthesised according to **general procedures H** and **S**. **General procedure H** used aldehyde **1c** (46 mg, 0.200 mmol), and *tert*-butyl(hex-5-yn-1-yl)sulfide **6b** (41 mg, 0.240 mmol). **General procedure S** used TFA (40 µL, 0.400 mmol), with stirring for 4 h. Purification by column chromatography (4:1 Hexane:Et_2O) yielded title compound **7b** (54.5 mg, 0.160 mmol, 79%) as a colourless oil.

δ_H (400 MHz, $CDCl_3$) 7.66 (1H, d, J = 8.3 Hz, *Ar*), 7.41 (1H, d, J = 1.9 Hz, *Ar*), 7.31 (1H, dd, J = 8.4, 1.8 Hz, *Ar*), 3.40-3.33 (1H, m, C(6)*H*), 3.11-2.96 (2H, m, C(5)H_2), 2.78-2.71 (1H, m, C(10)*H*H), 2.62-2.56 (1H, m, C(10)H*H*), 2.42 (3H, s, C(1)H_3), 2.13-2.07 (1H, m, C(7)*H*H), 1.96-1.89 (1H, m, C(9)*H*H), 1.87-1.79 (1H, m, C(8)*H*H), 1.65-1.54 (1H, m, C(9)H*H*), 1.49-1.37 (2H, m, C(7)H*H* and C(8)H*H*); **δ_C** (101 MHz, $CDCl_3$) 197.9, 145.4, 132.9, 131.5, 127.8, 126.6, 45.8, 37.8, 34.4, 29.5, 27.0, 25.9, 16.2; **ν_{max}**/ cm^{-1} (neat) 2921, 2851, 1672, 1572, 1536,

1458, 1436, 1364, 1342, 1312, 1275, 1210, 1177, 1148, 1095, 1041, 982, 925; **m/z HRMS** (ESI$^+$) [M + H]$^+$ $C_{14}H_{18}O^{79}BrS_2^+$ calc. 346.9955, found 346.9956.

1-(2-(Methylthio)-3-(trifluoromethyl)phenyl)-2-(tetrahydro-2*H*-thiopyran-2-yl)ethan-1-one (7c)

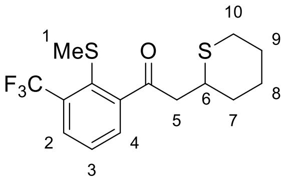

Synthesised according to **general procedures H** and **S**. **General procedure H** used aldehyde **1b** (44 mg, 0.200 mmol), and *tert*-butyl(hex-5-yn-1-yl)sulfide **6b** (41 mg, 0.240 mmol). **General procedure S** used TFA (40 µL, 0.400 mmol), with stirring for 4 h. Purification by column chromatography (9:1 Petane:Et_2O) yielded title compound **7c** (60.9 mg, 0.182 mmol, 91%) as a orange oil.

δ_F (377 MHz, $CDCl_3$) -59.73; **δ_H** (400 MHz, $CDCl_3$) 7.77-7.75 (1H, m, *Ar*), 7.54-7.46 (1H, m, *Ar*), 7.44-7.39 (1H, m, *Ar*), 3.41-3.39 (1H, br, C(6)*H*), 3.17-3.05 (2H, m, C(5)H_2), 2.78-2.68 (1H, m, C(10)*H*H), 2.65-2.56 (1H, m, C(10)*H*H), 2.32 (3H, s, C(1)H_3), 2.18-2.09 (1H, m, C(7)*H*H), 1.97-1.89 (1H, m, C(9)*H*H), 1.88-1.82 (1H, m, C(8)*H*H), 1.68-1.55 (1H, m, C(9)H*H*), 1.55-1.42 (2H, m, C(7)H*H* and C(8)H*H*); **δ_C** (101 MHz, $CDCl_3$) 203.4, 151.2, 135.2 (q, J_{CF} = 29.5 Hz), 130.9, 129.7, 129.3, 127.9 (q, J_{CF} = 5.6 Hz), 123.4 (q, J_{CF} = 274.0 Hz), 50.1, 37.1, 34.1, 29.2, 27.0, 25.7, 22.5; **ν_{max}**/ cm^{-1} (neat) 2924, 2853, 1706, 1578, 1422, 1317, 1281, 1137, 1100, 1014, 975; **m/z HRMS** (ESI$^+$) [M + H]$^+$ $C_{15}H_{18}F_3OS_2^+$ calc. 335.0746, found 335.0746.

1-(4,5-Dimethoxy-2-(methylthio)phenyl)-2-(tetrahydro-2*H*-thiopyran-2-yl)ethan-1-one (7d)

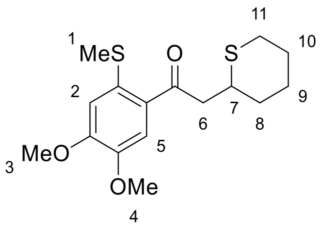

Synthesised according to **general procedures H** and **S**. **General procedure H** used aldehyde **1d** (43 mg, 0.200 mmol) and *tert*-butyl(hex-5-yn-1-yl)sulfide **6b** (41 mg, 0.240 mmol). **General procedure S** used TFA (40 µL, 0.400 mmol), with stirring for 4 h. Purification by column chromatography (1:1 Pentane:Et_2O) yielded title compound **7d** (24.4 mg, 0.0747 mmol, 37%) as a yellow oil.

δ_H (400 MHz, $CDCl_3$) 7.29 (1H, s, *Ar*), 6.81 (1H, s, *Ar*), 3.96 (3H, s, C(3 or 4)H_3), 3.92 (3H, s, C(3 or 4)H_3), 3.45-3.35 (1H, br, C(7)*H*), 3.12-2.97 (2H, m, C(6)H_2), 2.31-2.72 (1H, m, C(11)*H*H), 2.64-2.56 (1H, m, C(11)H*H*), 2.44 (3H, s, C(1)H_3), 2.17-2.09 (1H, m, C(8)*H*H), 1.98-1.90 (1H, m, C(10)*H*H), 1.87-1.80 (1H, m, C(9)*H*H), 1.65-1.54 (1H, m, C(10)H*H*), 1.47-1.38 (2H, m, C(8)H*H* and C(9)H*H*); δ_C (101 MHz, $CDCl_3$) 197.2, 152.7, 145.7, 136.4, 113.4, 108.7, 56.5, 56.1, 45.9, 38.1, 34.6, 27.1, 26.0, 16.8; ν_{max}/ cm^{-1} (neat) 2920, 2850, 1660, 1598, 1552, 1502, 1437, 1346, 1268, 1203, 1167, 1030; **m/z HRMS** (ESI^+) $[M + Na]^+$ $C_{16}H_{22}O_3{}^{23}NaS_2^+$ calc. 349.0903, found 349.0903.

1-(3-(Methylthio)thiophen-2-yl)-2-(tetrahydro-2*H*-thiopyran-2-yl)ethan-1-one (7e)

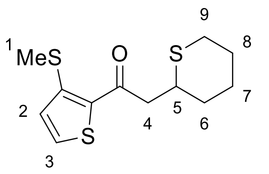

Synthesised according to **general procedures H** and **S**. **General procedure H** used aldehyde **1e** (32 mg, 0.200 mmol) and *tert*-butyl(hex-5-yn-1-yl)sulfide **6b** (41 mg, 0.240 mmol). **General procedure S** used TFA (40 µL , 0.400 mmol), with stirring for 4 h. Purification by column chromatography (85:15 Pentane:EtOAc) yielded title compound **7e** (44.7 mg, 0.164 mmol, 82%) as an orange oil.

δ_H (400 MHz, $CDCl_3$) 7.54 (1H, d, *J* = 5.2 Hz, *Ar*), 7.04 (1H, d, *J* = 5.2 Hz, *Ar*), 3.44-3.38 (1H, m, C(5)*H*), 3.00-2.88 (2H, m, C(4)H_2), 2.77-2.70 (1H, m, C(9)*H*H), 2.63-2.58 (1H, m, C(9)*H*H), 2.53 (3H, s, C(1)H_3), 2.14-2.09 (1H, m, C(6)*H*H), 1.96-1.89 (1H, m, C(8)*H*H), 1.86-1.81 (1H, m, C(7)*H*H), 1.68-1.56 (1H, m, C(8)H*H*), 1.52-1.40 (2H, m, C(6)H*H* and C(7)H*H*); **δ_C** (101 MHz, $CDCl_3$) 189.7, 146.4, 131.2, 126.4, 46.9, 38.1, 34.3, 29.4, 27.0, 25.8, 16.7; **ν_{max}**/ cm^{-1} (neat) 2850, 1641, 1478, 1397, 1355, 1173, 1004; **m/z HRMS** (ESI^+) $[M + H]^+$ $C_{12}H_{16}NOS_3^+$ calc. 273.0436, found 273.0436.

2,2-Dimethyl-1-morpholino-4-(tetrahydro-2*H*-thiopyran-2-yl)butane-1,3-dione (7f)

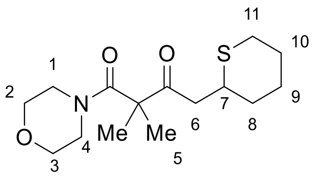

Synthesised according to **general procedures H** and **S**. **General procedure H** used aldehyde **1k** (37 mg, 0.200 mmol) and *tert*-butyl(hex-5-yn-1-yl)sulfide **6b** (41 mg, 0.240 mmol). **General procedure S** used TFA (40 µL, 0.400 mmol), with stirring for 4 h. Purification by

column chromatography (1:1 Pentane:EtOAc) yielded title compound **7f** (40.8 mg, 0.136 mmol, 68%) as a yellow oil.

δ_H (400 MHz, $CDCl_3$) 3.63 (8H, br, C(1 to 4)*H_2*), 3.32-3.22 (1H, m, C(7)*H*), 2.81-2.64 (1H, m, C(11)*H*H), 2.63-2.52 (3H, m, C(11)H*H* and C(6)*H_2*), 1.99-1.87 (2H, m, C(10)*H*H and C(8)*H*H), 1.87-1.76 (1H, m, C(9)*H*H), 1.72–1.48 (1H, m, C(10)H*H*), 1.49-1.39 (2H, m, C(8)H*H* and C(9)H*H*), 1.38 (6H, br, C(5)*H_3*); **δ_C** (101 MHz, $CDCl_3$) 207.8, 170.9, 66.6 (bs, 2C), 55.8, 46.7 (bs), 44.0, 44.0 (bs), 36.4, 34.2, 29.1, 26.8, 25.7, 23.7, 23.2; **ν_{max}**/ cm^{-1} (neat) 2925, 2852, 1708, 1639, 1422, 1362, 1272, 1247, 1183, 1115, 1022; **m/z HRMS** (ESI^+) $[M + Na]^+$ $C_{15}H_{25}O_3N^{23}NaS^+$ calc. 322.1447, found 322.1448.

1-(4-Bromo-2-(methylthio)phenyl)-2-(1,4-oxathian-3-yl)ethan-1-one (7h)

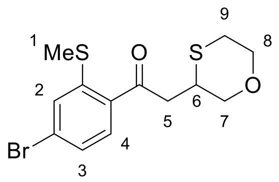

Synthesised according to **general procedures H** and **S**. **General procedure H** used aldehyde **1c** (46 mg, 0.200 mmol) and *tert*-butyl(2-(prop-2-yn-1-yloxy)ethyl)sulfane **6c** (41 mg, 0.240 mmol). **General procedure S** used TFA (60 µL , 0.600 mmol), with stirring for 16 h. Purification by column chromatography (9:1 to 1:1 Pentane:Et_2O) yielded title compound **7h** (35.1 mg, 0.101 mmol, 51%) as a yellow oil.

δ_H (400 MHz, $CDCl_3$) 7.69 (1H, d, *J* = 8.4 Hz, *Ar*), 7.42 (1H, d, *J* = 1.8 Hz, *Ar*), 7.32 (1H, dd, *J* = 8.3, 1.8 Hz, *Ar*), 4.11 (1H, dd, *J* = 11.7, 2.7 Hz, C(7)*H*H), 3.95 (1H, ddd, *J* = 11.7, 6.4, 3.2 Hz, C(8)*H*H), 3.86 (1H, ddd, *J* = 11.8, 6.8, 3.2 Hz, C(8)H*H*), 3.67 (1H, dd, *J* = 11.7, 6.4 Hz, C(7)H*H*), 3.41 (1H, qd, *J* = 6.6, 2.7 Hz, C(6)*H*), 3.30-3.18 (2H, m, C(5)*H_2*), 2.77-2.63 (2H, m, C(9)*H_2*), 2.43 (3H, s, C(1)*H_3*); **δ_C** (101 MHz, $CDCl_3$) 197.2, 145.5, 132.5, 131.4, 128.1, 127.9,

126.6, 73.0, 68.6, 42.0, 34.0, 26.0, 16.2; **ν_{max}**/ cm^{-1} (neat) 2918, 2852, 1671, 1572, 1537, 1457, 1434, 1410, 1365, 1319, 1285, 1270, 1243, 1212, 1149, 1106, 1094, 1045, 1017, 987, 974, 924, 903; **m/z HRMS** (ESI^+) $[M + Na]^+$ $C_{13}H_{15}BrO_2{}^{23}NaS_2{}^+$ calc. 370.9567, found 370.9567.

1-(2-(Methylthio)-3-(trifluoromethyl)phenyl)-2-(1,4-oxathian-3-yl)ethan-1-one (7i)

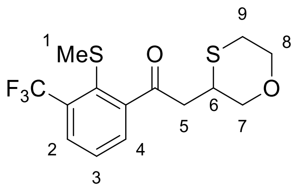

Synthesised according to **general procedures H** and **S**. **General procedure H** used aldehyde **1b** (44 mg, 0.200 mmol) and *tert*-butyl(2-(prop-2-yn-1-yloxy)ethyl)sulfane **6c** (41 mg, 0.240 mmol). **General procedure S** used TFA (80 µL, 0.800 mmol), with stirring for 16 h. Purification by column chromatography (9:1 to 7:3 Petrol:Et_2O) yielded title compound **7i** (23.1 mg, 0.069 mmol, 34%) as a yellow oil.

δ_F (377 MHz, $CDCl_3$) -59.76; **δ_H** (400 MHz, $CDCl_3$) 7.78 (1H, dd, J = 7.8, 1.5 Hz, *Ar*), 7.51 (1H, td, J = 7.7, 0.9 Hz, *Ar*), 7.41 (1H, dd, J = 7.7, 1.5 Hz, *Ar*), 4.15 (1H, dd, J = 11.7, 2.8 Hz, C(7)*H*H), 3.98 (1H, ddd, J = 11.8, 5.5, 3.7 Hz, C(8)*H*H), 3.84 (1H, ddd, J = 11.7, 6.4, 3.9 Hz, C(8)H*H*), 3.70 (1H, dd, J = 11.7, 6.8 Hz, C(7)H*H*), 3.42 (1H, qd, J = 6.9, 2.8 Hz, C(6)*H*), 3.31-3.28 (1H, dd, J = 17.9, 6.9 Hz, C(5)*H*H), 3.21 (1H, dd, J = 17.9, 6.9 Hz, C(5)H*H*), 2.75-2.66 (2H, m, C(9)H_2), 2.33 (3H, s, C(1)H_3); **δ_C** (101 MHz, $CDCl_3$) (major signals only) 202.6, 150.8, 135.1 (q, J_{CF} = 29.7 Hz), 130.8, 129.4, 129.3, 127.9 (q, J_{CF} = 5.6 Hz), 123.3 (q, J_{CF} = 273.9 Hz), 72.9, 68.4, 46.1, 33.6, 26.0, 22.4; **ν_{max}**/ cm^{-1} (neat) 2926, 2854, 2348, 1706, 1578, 1422, 1318, 1161, 1130, 1101, 1053, 927; **m/z HRMS** (ESI^+) $[M + H]^+$ $C_{14}H_{16}F_3O_2S_2{}^+$ calc. 353.0487, found 353.0487.

2,2-Dimethyl-1-morpholino-4-(1,4-oxathian-3-yl)butane-1,3-dione (7j)

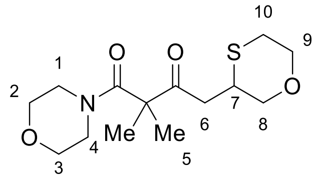

Synthesised according to **general procedures H** and **S**. **General procedure H** used aldehyde **1k** (37 mg, 0.200 mmol) and *tert*-butyl(2-(prop-2-yn-1-yloxy)ethyl)sulfane **6c** (41 mg, 0.240 mmol). **General procedure S** used TFA (80 µL, 0.800 mmol), with stirring for 16 h. Purification by column chromatography (7:3 EtOAc:Pentane) yielded title compound **7j** (49.8 mg, 0.16 mmol, 83%) as a yellow oil.

δ_H (400 MHz, $CDCl_3$) 3.99 (1H, dd, J = 11.7, 2.8 Hz, C(8)*H*H), 3.93-3.79 (2H, m, C(9)H_2), 3.67-3.51 (9H, m, C(1 to 4)H_2 and C(8)H*H*), 3.24 (1H, qd, J = 6.7, 2.7 Hz, C(7)*H*), 2.78 (2H, dd, J = 6.8, 2.1 Hz, C(6)H_2), 2.72-2.55 (2H, m, SC(10)H_2), 1.38 (6H, br, C(5)H_3); **δ_C** (101 MHz, $CDCl_3$) 207.6, 170.8, 72.8, 68.6, 66.6 (bs, 2C), 55.8, 46.3 (bs), 43.4 (bs), 40.3, 33.1, 25.5, 23.6, 23.2; **ν_{max}**/ cm^{-1} (neat) 2921, 2853, 1708, 1637, 1456, 1421, 1385, 1363, 1272, 1249, 1183, 1112, 1095, 1063, 1021, 995, 980, 951, 926; **m/z HRMS** (ESI^+) $[M + H]^+$ $C_{14}H_{23}NO_4S^+$ calc. 302.1421, found 302.1420.

1-(1-(Morpholine-4-carbonyl)cyclopentyl)-2-(1,4-oxathian-3-yl)ethan-1-one (7k)

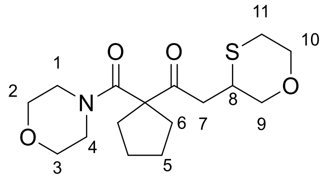

Synthesised according to **general procedures H** and **S**. **Procedure H** used aldehyde **1j** (42.3 mg, 0.200 mmol) and *tert*-butyl(2-(prop-2-yn-1-yloxy)ethyl)sulfane **6c** (41 mg, 0.240 mmol). **General procedure S** used TFA (0.16 mL, 2.00 mmol), with stirring for 22 h. Purification by column chromatography (7:3 Et_2O:Pentane) yielded title compound **7k** (18.4 mg, 0.056 mmol, 49%) as a yellow oil.

δ_H (400 MHz, $CDCl_3$) 3.98 (1H, dd, *J* = 11.7, 2.7 Hz, C(8)*H*H), 3.95-3.79 (2H, m, C(10)H_2), 3.73-3.48 (9H, m, C(1 to 4)H_2 and C(9)H*H*), 3.30-3.17 (2H, m, C(7)*H*H and C(8)*H*), 2.83-2.66 (2H, m, C(7)H*H* and C(11)*H*H), 2.64-2.55 (1H, m, C(11)H*H*), 2.42-1.94 (4H, m, C(5 and 6)H_2), 1.73-1.50 (4H, m, C(5 and 6)H_2); **δ_C** (101 MHz, $CDCl_3$) 205.1, 170.6, 72.9, 68.6, 67.2, 66.9, 66.3, 46.3, 43.3, 40.8, 34.2, 33.4, 33.2, 26.3, 26.3, 25.5; **ν_{max}**/ cm^{-1} (neat) 3504, 3018, 2986, 2952, 2917, 2853, 2360, 1708, 1637, 1453, 1422, 1358, 1298, 1273, 1238, 1203, 1178, 1114, 1064, 1030, 993, 914; **m/z HRMS** (ESI^+) $[M + Na]^+$ $C_{16}H_{25}NO_4{}^{23}NaS^+$ calc. 350.1397, found 350.1397.

2-(Isothiochroman-1-yl)-1-(2-(methylthio)phenyl)ethan-1-one (7l)

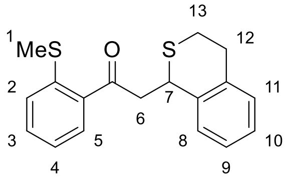

Synthesised according to **general procedures H** and **S**. **General procedure H** used aldehyde **1a** (26 µL, 0.200 mmol) and *tert*-butyl(2-ethynylphenethyl)sulfane **6d** (52.4 mg, 0.240 mmol). **General procedure S** used TFA (40 µL, 0.400 mmol), with stirring for 4 h. Purification by

column chromatography (9:1 Pentane:Et_2O) yielded title compound **7l** (53.1 mg, 0.169 mmol, 84%) as yellow oil.

δ_H (400 MHz, $CDCl_3$) 7.78 (1H, dd, *J* = 7.9, 1.5 Hz, *Ar*), 7.51-7.42 (1H, m, *Ar*), 7.38-7.31 (1H, m, *Ar*), 7.20-7.11 (5H, m, *Ar*), 4.67 (1H, dd, *J* = 8.8, 5.1 Hz, C(7)*H*), 3.70 (1H, dd, *J* = 17.1, 8.8 Hz, C(6)*H*H), 3.47 (1H, dd, *J* = 17.1, 5.1 Hz, C(6)*H*H), 3.11-3.05 (2H, m, C(12)H_2), 3.01-2.90 (1H, m, C(13)*H*H), 2.89-2.79 (1H, m, C(13)*H*H), 2.44 (3H, s, C(1)H_3); **δ_C** (101 MHz, $CDCl_3$) 198.3, 142.7, 137.9, 136.7, 134.6, 132.4, 130.2, 129.7, 127.5, 127.0, 126.7, 125.4, 123.6, 48.7, 36.5, 31.0, 24.7, 16.2; **ν_{max}**/ cm^{-1} (neat) 3058, 3018, 2919, 2853, 2357, 1670, 1586, 1557, 1489, 1460, 1432, 1376, 1347, 1319, 1290,1273, 1233, 1205, 1171, 1141, 1107, 1079, 1045, 978, 903; **m/z HRMS** (ESI^+) $[M + Na]^+$ $C_{18}H_{18}O^{23}NaS_2^+$ calc. 337.0691, found 337.0693.

1-(2-(Methylthio)phenyl)-2-(tetrahydrothiophen-2-yl)ethan-1-one (7m)

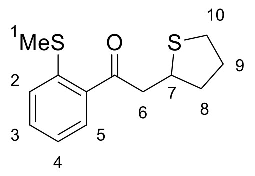

Synthesised according to **general procedures H** and **S**. **General procedure H** used aldehyde **1a** (26 µL, 0.200 mmol) and *tert*-butyl(pent-4-yn-1-yl)sulfide **6e** (38 mg, 0.240 mmol). **General procedure S** used TFA (20 µL, 0.200 mmol), with stirring for 2 h. Purification by column chromatography (8:2 Pentane:Et_2O) yielded title compound **7m** (22.1 mg, 0.088 mmol, 44%) as a yellow oil.

δ_H (400 MHz, $CDCl_3$) 7.81 (1H, dd, *J* = 7.9, 1.5 Hz, *Ar*), 7.49-7.44 (1H, m, *Ar*), 7.34-7.32 (1H, m, *Ar*), 7.21-7.17 (1H, m, *Ar*), 3.92 (1H, p, *J* = 6.8 Hz, C(7)*H*), 3.31 (1H, dd, *J* = 17.1, 6.7 Hz, C(6)*H*H), 3.24 (1H, dd, *J* = 17.1, 6.7 Hz, C(6)H*H*), 2.96-2.83 (2H, m, C(10)H_2), 2.43 (3H, s, C(1)H_3), 2.30-2.22 (1H, m, C(8)*H*H), 2.12-2.04 (1H, m, C(9)*H*H), 2.01-1.91 (1H, m, C(9)H*H*),

1.68-1.60 (1H, m, C(8)H*H*); **δ_C** (101 MHz, $CDCl_3$) 199.5, 142.4, 134.3, 132.2, 130.1, 125.2, 123.5, 48.0, 43.4, 37.0, 32.4, 30.2, 16.0; **ν_{max}**/ cm^{-1} (neat) 2918, 2857, 1667, 1586, 1557, 1461, 1431, 1359, 1268, 1240, 1204, 1172, 1139, 1079, 1045, 1003, 977; **m/z** [**HRMS** (ESI^+) $[M + Na]^+$ $C_{13}H_{16}O^{23}NaS_2^+$ calc. 275.0535, found 275.0534.

2-((2*R,3*R**)-2-methyl-1,4-oxathian-3-yl)-1-(2-(methylthio)phenyl)ethan-1-one (7n)**

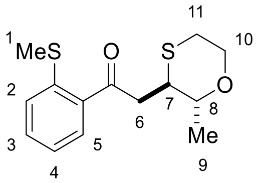

Synthesised according to **general procedures H** and **S**. **General procedure H** used aldehyde **1a** (26 µL, 0.200 mmol) and (2-(but-3-yn-2-yloxy)ethyl)(*tert*-butyl)sulfane **6f** (44.7 mg, 0.240 mmol). **General procedure S** used TFA (20 µL, 0.200 mmol), with stirring for 3 h. Purification of the crude residue (2:1 dr) by column chromatography (6:4 Pentane:Et_2O) yielded title compound as an inseparable mixture of diastereoisomers **7n** (12.7 mg, 0.045 mmol, 22%) as an orange oil.

δ_H (400 MHz, $CDCl_3$) (major signals only) 7.94-7.72 (1H, m, *Ar*), 7.51-7.43 (1H, m, *Ar*), 7.38-7.31 (1H, m, *Ar*), 7.24-7.17 (1H, m, *Ar*), 4.25-4.13 (1H, m, C(10)*H*H), 3.77-3.68 (1H, m, C(10)*H*H), 3.65-3.53 (1H, m, C(8)*H*), 3.43-3.29 (1H, m, C(7)*H*), 3.27-3.17 (1H, m, C(6)*H*H), 3.03-2.90 (2H, m, C(6)*H*H and C(11)*H*H), 2.44 (3H, s, C(1)H_3), 2.44-2.38 (1H, m, C(11)*H*H), 1.27 (3H, d, J = 6.3 Hz, C(9)H_3); **δ_C** (101 MHz, $CDCl_3$) (major signals only) 198.1, 142.7, 132.5, 130.2, 125.56, 123.7, 78.2, 67.9, 42.7, 40.2, 29.8, 28.2, 19.8, 16.2; **ν_{max}**/ cm^{-1} (neat) 2979, 2917, 2853, 2363, 2342, 1672, 1586, 1558, 1461, 1433, 1376, 1352, 1296, 1273, 1246, 1221,

1174, 1140, 1107, 1089, 1047, 1017, 978; **m/z HRMS** (ESI^+) $[M + Na]^+$ $C_{14}H_{18}O_2{}^{23}NaS_2{}^+$ calc. 351.0695, found 351.0697.

2-((2*R,3*R**)-2-methyl-1,4-oxathian-3-yl)-1-(2-(methylthio)-3-(trifluoromethyl)phenyl)ethan-1-one (7o)**

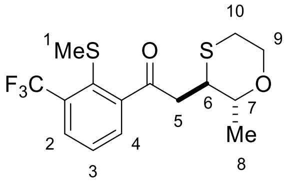

Synthesised according to **general procedures H** and **S**. **General procedure H** used aldehyde **1a** (44 µL, 0.200 mmol) and (2-(but-3-yn-2-yloxy)ethyl)(*tert*-butyl)sulfane **6g** (44.7 mg, 0.240 mmol). **General procedure S** used TFA (80 µL, 0.800 mmol), with stirring for 22 h. Purification of the crude residue (2.4:1 dr) by column chromatography (94:6 to 8:2 Petrol:Et_2O) yielded title compound as an inseparable mixture of diastereoisomers **7o** (33.9 mg, 0.097 mmol, 48%) as a yellow oil.

δ_F (377 MHz, $CDCl_3$) (major signals only) -59.72; **δ_H** (400 MHz, $CDCl_3$) (major signals only) 7.79-7.77 (1H, m, *Ar*), 7.54-7.45 (3H, m, *Ar*), 4.20-4.15 (1H, m, C(9)*H*H), 3.75-3.69 (1H, m, C(9)H*H*), 3.63-3.56 (2H, m, C(5)*H*H and C(7)*H*), 3.38 (1H, dd, J = 17.9, 4.0 Hz, C(5)H*H*), 3.09-3.05 (1H, m, C(6)*H*), 3.03-2.91 (1H, m, C(10)*H*H), 2.42 (1H, dt, J = 13.6, 2.6 Hz, C(10)*H*H), 2.32 (3H, s, C(1)H_3), 1.31 (3H, d, J = 6.3 Hz, C(8)H_3); **δ_C** (101 MHz, $CDCl_3$) (major signals only); 202.7, 150.9, 135.1 (q, J_{CF} = 29.5 Hz), 129.4, 129.3, 128.0 (q, J_{CF} = 5.6 Hz), 123.4 (q, J_{CF} = 273.8 Hz), 77.8, 68.0, 42.4, 36.4, 28.1, 22.5, 19.8; **ν_{max}**/ cm^{-1} (neat) 2979, 2927, 2854, 1707, 1577, 1421, 1377, 1316, 1298, 1277, 1162, 1133, 1101, 1052, 1019, 976; **m/z HRMS** (ESI^+) $[M + H]^+$ $C_{15}H_{18}O_2F_3S_2{}^+$ calc. 351.0695, found 351.0697.

2,2-dimethyl-4-((2*R,3*R**)-2-methyl-1,4-oxathian-3-yl)-1-morpholinobutane-1,3-dione (7p)**

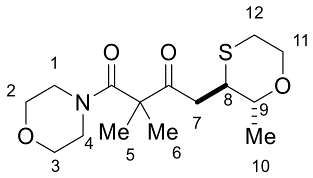

Synthesised according to **general procedures H** and **S**. **General procedure H** used aldehyde **1k** (37 mg, 0.200 mmol) and (2-(but-3-yn-2-yloxy)ethyl)(*tert*-butyl)sulfane **6f** (44.7 mg, 0.240 mmol). **General procedure S** used TFA (80 µL, 0.8 mmol), with stirring for 16 h. Purification of the crude residue (3:1 dr) by column chromatography (7:3 EtOAc:Pentane) yielded title compound as an inseparable mixture of diastereoisomers **7p** (51.3 mg, 0.163 mmol, 81%) as a yellow-orange oil.

δ_H (400 MHz, $CDCl_3$) (major signals only) 4.21-4.08 (1H, m, C(11)*H*H), 3.81-3.50 (10H, m, C(1 to 4)H_2), C(9)*H* and C(11)H*H*), 3.19-3.10 (1H, m, SC(8)*H*), 2.96-12.86 (1H, m, C(12)*H*H), 2.78-2.68 (1H, m, C(7)*H*H), 2.63-2.54 (1H, m, C(7)*H*H), 2.45-2.37 (1H, m, C(12)*H*H), 1.39 (3H, s, C(5 or 6)H_3), 1.39 (3H, s, C(5 or 6)H_3), 1.22 (3H, d, J = 6.3 Hz, C(10)H_3); **δ_C** (101 MHz, $CDCl_3$) (major signals only) 208.4, 170.8, 77.0, 67.5, 66.6 (bs, 2C), 55.9, 40.5, 38.9, 27.8, 23.8, 23.3, 19.5; **ν_{max}**/ cm^{-1} (neat) 2922, 2854, 1710, 1637, 1460, 1421, 1376, 1364, 1298, 1272, 1247, 1182, 1112, 1089, 1067, 1021, 954; **m/z HRMS** (ESI$^+$) $[M + H]^+$ $C_{15}H_{26}NO_4{}^{23}NaS^+$ calc. 316.1577, found 316.1579.

2,2-dimethyl-1-morpholino-4-((2*R,3*R**)-2-phenyl-1,4-oxathian-3-yl)butane-1,3-dione (7q)**

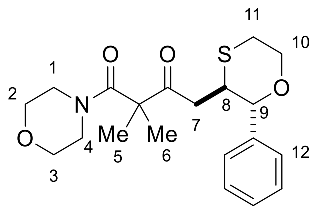

Synthesised according to **general procedures H** and **S**. **General procedure H** used aldehyde **1k** (37 mg, 0.200 mmol) and *tert*-butyl(2-((1-phenylprop-2-yn-1-yl)oxy)ethyl)sulfane **6g** (59.6 mg, 0.240 mmol). **General procedure S** used TFA (0.06 mL, 0.6 mmol), with stirring for 16 h. Purification of the crude residue (2:1 dr) by column chromatography (9:1 to 1:1 Petrol:EtOAc) yielded title compound as an inseparable mixture of diastereoisomers **7q** (32.3 mg, 0.856 mmol, 43%) as a yellow oil.

δ_H (400 MHz, $CDCl_3$) (major signals only) 7.38-7.28 (5H, m, *Ar*), 4.39-4.31 (2H, m, C(9)*H* and C(10)*H*H), 3.88 (1H, td, *J* = 11.9, 2.0 Hz, C(10)*H*H), 3.60 (1H, td, *J* = 9.8, 2.5 Hz, C(8)*H*), 3.49-3.30 (4H, m, C(1 to 4)H_2), 3.30-3.15 (1H, m, C(11)*H*H), 3.14-2.89 (2H, m, C(1 to 4)H_2), 2.84-2.64 (2H, m, C(1 to 4)H_2), 2.53-2.39 (2H, m, C(11)*H*H and C(7)*H*H), 2.28-2.20 (1H, m, C(7)*H*H), 1.28 (3H, s, C(5 or 6)H_3), 1.18 (3H, s, C(5 or 6)H_3); **δ_C** (101 MHz, $CDCl_3$) (major signals only); 206.4, 170.6, 139.2, 128.8, 128.8, 127.4, 85.3, 69.5, 66.5 (bs), 65.8 (bs), 55.4, 46.2 (bs), 43.0 (bs), 39.9, 38.7, 28.4, 23.7, 22.4; **ν_{max}**/ cm^{-1} (neat) 2918, 2855, 1714, 1639, 1421, 1361, 1298, 1272, 1248, 1183, 1115, 1023; **m/z HRMS** (ESI^+) $[M + Na]^+$ $C_{20}H_{27}NO_4{}^{23}NaS^+$ calc. 400.1577, found 400.1557.

4-((3*R,6*S**)-6-(*tert*-butyl)-1,4-oxathian-3-yl)-2,2-dimethyl-1-morpholinobutane-1,3-dione (7r)**

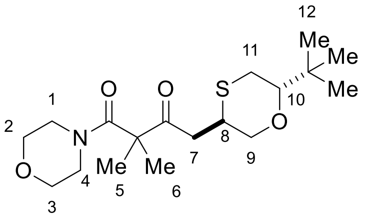

Synthesised according to **general procedures H** and **S**. **General procedure H** used aldehyde **1k** (37 mg, 0.200 mmol) and *tert*-butyl(3,3-dimethyl-2-(prop-2-yn-1-yloxy)butyl)sulfane **6h** (54.8 mg, 0.240 mmol). **General procedure S** used TFA (80 µL, 0.800 mmol), with stirring for 30 min. Purification of the crude residue (4:1 dr) by column chromatography (7:3 to 1:1 Petrol:EtOAc) yielded title compound as an inseparable mixture of diastereoisomers **7r** (53.2 mg, 0.149 mmol, 74%) as a yellow oil.

δ_H (400 MHz, $CDCl_3$) (major signals only) 4.15 (1H, m, C(9)*H*H), 3.61 (8H, br, C(1 to 4)*H_2*), 3.38-3.25 (2H, m, C(9)H*H* and C(8)*H*), 3.08 (1H, dd, *J* = 10.7, 1.7 Hz, C(10)*H*), 2.74 (1H, dd, *J* = 13.3, 10.7 Hz, C(11)*H*H), 2.43-2.36 (3H, m, C(11)H*H* and C(7)*H_2*), 1.34 (3H, s, C(5 or 6)*H_3*), 1.34 (3H, s, C(5 or 6)*H_3*), 0.87 (9H, s, C(12)*H_3*); **δ_C** (101 MHz, $CDCl_3$) (major signals only) 207.0, 170.7, 85.8, 74.7, 66.6 (bs, 2C), 55.8, 46.44 (bs), 43.44 (bs), 39.3, 34.9, 33.8, 27.8, 26.0, 23.7, 23.1; **ν_{max}**/ cm^{-1} (neat) 2957, 2855, 2363, 1710, 1639, 1466, 1421, 1363, 1301, 1272, 1248, 1182, 1150, 1114, 1091, 1065, 1022, 975, 950, 916; **m/z HRMS** (ESI^+) $[M + H]^+$ $C_{18}H_{32}NO_4S^+$ calc. 358.2047, found 358.2051.

4-((3*R,6*S**)-6-cyclohexyl-1,4-oxathian-3-yl)-2,2-dimethyl-1-morpholinobutane-1,3-dione (7s)**

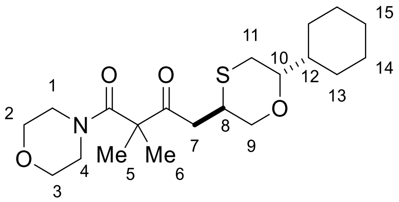

Synthesised according to **general procedures H** and **S**. **General procedure H** used aldehyde **1k** (37 mg, 0.200 mmol) and *tert*-butyl(2-cyclohexyl-2-(prop-2-yn-1-yloxy)ethyl)sulfane **6i** (61.1 mg, 0.240 mmol). **General procedure S** used TFA (80 µL, 0.8 mmol), with stirring for 1.5 h. Purification of the crude residue (3:1 dr) by column chromatography (8:1 to 1:1 Petrol:EtOAc) yielded title compound as an inseparable mixture of diastereoisomers **7s** (55.4 mg, 0.144 mmol, 72%) as a pale-yellow oil.

δ_H (400 MHz, $CDCl_3$) (major signals only) 4.16-4.10 (1H, m, C(9)*H*H), 3.73-3.46 (8H, br, C(1 to 4)H_2), 3.42-3.22 (2H, m, C(9)H*H* and C(8)*H*), 3.22-3.16 (1H, m, C(10)*H*), 2.77-2.60 (1H, m, C(11)*H*H), 2.45-2.37 (3H, m, C(11)H*H* and C(7)H_2), 1.88-1.80 (1H, m, Cy-*H*), 1.76-1.66 (2H, m, Cy-*H*), 1.67-1.58 (2H, m, Cy-*H*), 1.35 (3H, s, C(5 or 6)H_3), 1.35 (3H, s, C(5 or 6)H_3), 1.27-0.82 (6H, m, Cy-*H*); **δ_C** (101 MHz, $CDCl_3$) (major signals only) 207.0, 170.7, 82.2, 74.2, 66.6 (bs, 2C), 55.8, 42.9, 39.4, 33.9, 29.9, 28.9, 28.7, 26.5, 26.2, 26.1, 23.7, 23.1; **ν_{max}**/ cm^{-1} (neat) 2922, 2851, 2246, 1709, 1638, 1447, 1420, 1385, 1362, 1300, 1272, 1247, 1183, 1150, 1113, 1089, 1020, 977, 950, 918; **m/z HRMS** (ESI^+) $[M + H]^+$ $C_{20}H_{34}NO_4S^+$ calc. 384.2203, found 384.2200.

4-((3*R,6*S**)-6-benzyl-1,4-oxathian-3-yl)-2,2-dimethyl-1-morpholinobutane-1,3-dione (7t)**

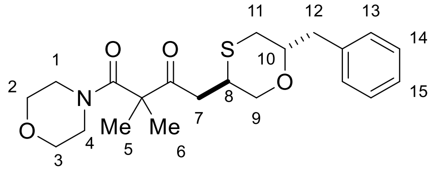

Synthesised according to **general procedures H** and **S**. **General procedure H** used aldehyde **1k** (37 mg, 0.200 mmol) and *tert*-butyl(3-phenyl-2-(prop-2-yn-1-yloxy)propyl)sulfane **6j** (63 mg, 0.240 mmol). **General procedure S** used TFA (80 µL, 0.800 mmol), with stirring for 2.5 h. Purification of the crude residue (2.6:1 dr) by column chromatography (7:3 to 1:1 Petrol:EtOAc) yielded title compound as an inseparable mixture of diastereoisomers **7t** (60.0 mg, 0.153 mmol, 77%) as an orange oil.

δ_H (400 MHz, $CDCl_3$) (major signals only) 7.27-7.08 (5H, m, *Ar*), 4.08 (1H, dd, *J* = 10.8, 2.4 Hz, C(9)*H*H), 3.67-3.59 (1H, m, C(10)*H*), 3.59-3.45 (8H, br, C(1 to 4)H_2), 3.38-3.23 (2H, m, C(9)H*H* and C(8)*H*), 2.89-2.81 (1H, m, C(11)*H*H), 2.69-2.53 (2H, m, C(11)H*H* and C(12)*H*H), 2.37 (1H, d, *J* = 6.3 Hz, C(7)H_2), 2.31 (1H, dd, *J* = 13.6, 1.9 Hz, C(12)*H*H), 1.29 (3H, s, C(5 or 6)H_3), 1.29 (3H, s, C(5 or 6)H_3); **δ_C** (101 MHz, $CDCl_3$) (major signals only) 206.9, 170.6, 137.5, 129.4, 128.5, 126.5, 78.4, 73.9, 66.6 (bs, 2C), 55.8, 46.4 (bs), 43.4 (bs), 42.3, 39.3, 33.7, 31.8, 23.7, 23.1; **ν_{max}**/ cm^{-1} (neat) 3499, 3026, 2919, 2854, 1734, 1708, 1636, 1495, 1453, 1421, 1385, 1361, 1300, 1271, 1246, 1183, 1144, 1113, 1092, 1020, 950, 911; **m/z HRMS** (ESI^+) $[M + H]^+$ $C_{21}H_{30}NO_4S^+$ calc. 392.1890, found 392.1886.

4-((3*R,6*S**)-6-ethyl-1,4-oxathian-3-yl)-2,2-dimethyl-1-morpholinobutane-1,3-dione (7u)**

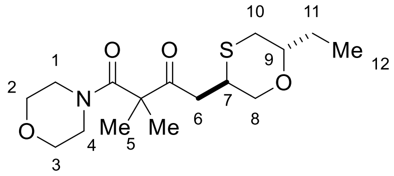

Synthesised according to **general procedures H** and **S**. **General procedure H** used aldehyde **1k** (37 mg, 0.200 mmol) and *tert*-butyl(2-(prop-2-yn-1-yloxy)butyl)sulfane **6k** (48.1 mg, 0.240 mmol). **General procedure S** used TFA (80 µL, 0.800 mmol), with stirring for 2.5 h. Purification of the crude residue (2.6:1 dr) by column chromatography (7:3 to 1:1 Petrol:EtOAc) yielded title compound as an inseparable mixture of diastereoisomers **7u** (26.8 mg, 0.0986 mmol, 49%) as a yellow oil.

δ_H (400 MHz, $CDCl_3$) (major signals only) 4.17-4.10 (1H, m, C(8)*H*H), 3.62 (8H, br, C(1 to 4)*H*$_2$), 3.50-3.20 (3H, m, C(8)H*H*, C(9)*H* and C(7)*H*), 2.67 (1H, dd, *J* = 13.5, 10.4 Hz, C(6)*H*H), 2.44 (2H, d, *J* = 6.4 Hz, C(10)*H*$_2$), 2.40 (1H, dd, *J* = 13.6, 1.9 Hz, C(6)H*H*), 1.62-1.41 (2H, m, C(11)*H*$_2$), 1.36-1.35 (6H, m, C(5)*H*$_3$), 0.92 (3H, t, *J* = 7.5 Hz, C(12)*H*$_3$); δ_C (101 MHz, $CDCl_3$) (major signals only) 207.0, 170.7, 79.1, 73.9, 66.8 (bs, 2C), 55.8, 39.4, 33.9, 31.9, 28.8, 23.7, 23.1, 9.9; ν_{max}/ cm^{-1} (neat) 2962, 2920, 2853, 1709, 1638, 1464, 1421, 1363, 1302, 1272, 1248, 1184, 1115, 1082, 1020, 949, 903; **m/z HRMS** (ESI^+) $[M + H]^+$ $C_{16}H_{28}NO_4{}^{23}NaS^+$ calc. 330.1734, found 330.1729.

2,2-dimethyl-1-morpholino-4-((3*R,8a*S**)-6-oxohexahydro-1H-pyrrolo[2,1-c][1,4]thiazin-3-yl)butane-1,3-dione (7v)**

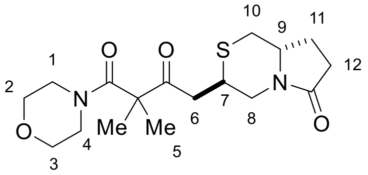

Synthesised according to **general procedures H** and **S**. **General procedure H** used aldehyde **1k** (37 mg, 0.200 mmol) and 5-((*tert*-butylthio)methyl)-1-(prop-2-yn-1-yl)pyrrolidin-2-one **6l** (54.1 mg, 0.24 mmol). **General procedure S** used TFA (0.1 mL, 2.0 mmol) with stirring for 22 h. Purification of the crude residue (3:1 dr) by column chromatography (100:0 to 9:1 EtOAc:MeOH) yielded title compound as an inseparable mixture of diastereoisomers **7v** (62 mg, 0.180 mmol, 88%) as a yellow oil.

δ_H (400 MHz, $CDCl_3$) (major signals only) 4.46 (1H, dd, *J* = 13.0, 3.2 Hz, C(8)*H*H), 3.79-3.61 (9H, m, C(1 to 4)H_2 and C(9)*H*), 3.36-3.23 (1H, m, C(7)*H*), 2.75-2.65 (5H, m, C(6)H_2, C(8)H*H* and C(10)H_2), 2.54-2.46 (2H, m, C(12)H_2), 2.42-2.31 (1H, m, C(11)*H*H), 1.82-1.69 (1H, m, C(11)H*H*), 1.50-1.44 (6H, br, C(5)H_3); δ_C (101 MHz, $CDCl_3$) (major signals only) 206.2, 174.1, 170.6, 66.6 (bs, 2C), 57.4, 55.8, 47.3, 46.6 (bs), 43.4 (bs), 40.5, 35.2, 34.9, 30.0, 24.7, 23.6, 23.2; ν_{max}/ cm^{-1} (neat) 3492, 2918, 2856, 1683, 1637, 1421, 1366, 1302, 1271, 1251, 1183, 1115, 1064, 1022, 922; **m/z HRMS** (ESI^+) $[M + H]^+$ $C_{17}H_{27}N_2O_4S^+$ calc. 355.1687, found 355.1686.

4-((3*R,6*S**)-6-cyclopropyl-1,4-oxathian-3-yl)-2,2-dimethyl-1-morpholinobutane-1,3-dione (7w)**

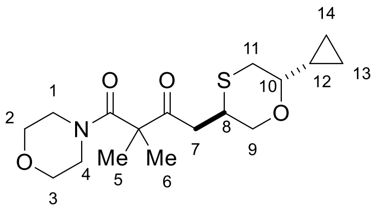

Synthesised according to **general procedures H** and **S**. **General procedure H** used aldehyde **1k** (37 mg, 0.200 mmol) and *tert*-butyl(2-cyclopropyl-2-(prop-2-yn-1-yloxy)ethyl)sulfane **6m** (56 mg, 0.240 mmol). **General procedure S** used TFA (80 µL, 0.800 mmol), with stirring for 2.5 h. Purification of the crude residue (2.7:1 dr) by column chromatography (1:1 Petrol:EtOAc) yielded title compound as an inseparable mixture of diastereoisomers **7w** (6.6 mg, 0.0193 mmol, 10%) as a pale-yellow oil.

δ_H (400 MHz, $CDCl_3$) (major signals only) 4.16 (1H, dd, *J* = 11.1, 2.8 Hz, C(9)*H*H), 3.63 (8H, br, C(1 to 4)H_2), 3.45-3.36 (1H, m, C(8)*H*), 3.36-3.26 (1H, m, C(9)H*H*), 2.90-2.73 (2H, m, C(7)*H*H and C(10)*H*), 2.53 (1H, dd, *J* = 13.2, 1.6 Hz, C(7)H*H*), 2.44 (2H, dd, *J* = 6.6, 1.4 Hz, C(11)H_2), 1.37 (3H, s, C(5 or 6)H_3), 1.36 (3H, s, C(5 or 6)H_3), 0.95-0.85 (1H, m, C(12)*H*), 0.64-0.46 (2H, m, C(13 and 14)H_2), 0.44-0.33 (1H, m, C(13 and 14)H_2), 0.29-0.16 (1H, m, C(13 and 14)H_2); **δ_C** (101 MHz, $CDCl_3$) (major signals only) 207.0, 170.7, 82.0, 74.0, 66.7 (bs, 2C), 55.9, 39.4, 33.8, 31.9, 23.8, 23.2, 15.8, 3.4, 2.0; **ν_{max}**/ cm^{-1} (neat) 3310, 2972, 2959, 2924, 2852, 1448, 1391, 1363, 1339, 1265, 1162, 1071, 1035, 1016, 970, 936, 910; **m/z HRMS** (ESI^+) $[M + H]^+$ $C_{17}H_{28}NO_4{}^{23}NaS^+$ calc. 342.1734, found 342.1732.

2,2-dimethyl-1-morpholino-4-((3*R,5*S**)-5-phenyl-1,4-oxathian-3-yl)butane-1,3-dione (7y)**

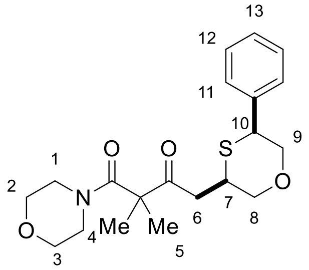

Synthesised according to **general procedures H** and **S**. **General procedure H** used aldehyde **1k** (37 mg, 0.200 mmol) and *tert*-butyl(1-phenyl-2-(prop-2-yn-1-yloxy)ethyl)sulfane **6q** (59.6 mg, 0.240 mmol). **General procedure S** used TFA (0.12 mL, 1.2 mmol), with stirring for 16 h. Purification of the crude residue (>20:1 dr) by column chromatography (7:3 to 8:2 Et_2O:Pentane) yielded title compound as a single diastereoisomer **7y** (43.7 mg, 0.116 mmol, 58%) as a yellow oil.

δ_H (400 MHz, $CDCl_3$) 7.37-7.24 (5H, m, *Ar*), 4.24-4.14 (3H, m, C(8)H_2O and C(9)*H*H), 3.70-3.54 (10H, m, C(7)*H*, C(9)H*H* and C(1 to 4)H_2), 3.34 (1H, t, *J* = 10.9 Hz, C(10)*H*), 2.53 (2H, d, *J* = 6.7 Hz, C(6)H_2), 1.37 (6H, br, C(5)H_3); **δ_C** (101 MHz, $CDCl_3$) 206.8, 199.8, 170.7, 137.5, 128.8, 128.1, 128.0, 73.7, 72.7, 66.6 (bs, 2C), 45.6, 39.4, 35.9, 23.7, 23.2; **ν_{max}**/ cm^{-1} (neat) 3505, 2987, 2922, 2852, 2247, 2063, 1709, 1636, 1491, 1451, 1422, 1386, 1361, 1298, 1272, 1248, 1182, 1152, 1111, 1092, 1065, 1036, 1018, 979, 948, 913; **m/z HRMS** (ESI^+) $[M + H]^+$ $C_{20}H_{28}NO_4S^+$ calc. 378.1734, found 378.1729.

7.5 Product derivatisations

1-(2-(Phenylethynyl)phenyl)-2-(tetrahydro-2*H*-thiopyran-2-yl)ethan-1-one (11h)

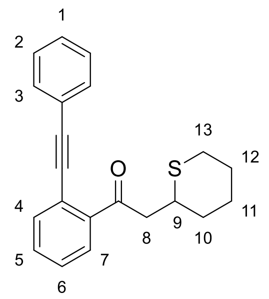

According to literature procedure,[8] Rh(nbd)$_2$BF$_4$ (6 mg, 0.016 mmol) and dcpe (7 mg, 0.016 mmol) were dissolved in DCE (2 mL). H_2 gas was bubbled through the solution for 2 min. The solution was purged with argon for 1 min. This was transferred *via* cannula to a mixture of cyclic sulfide **7a** (87 mg, 0.320 mmol), phenylacetylene (70 µL, 0.640 mmol), copper bromide (92 mg, 0.640 mmol) and silver carbonate (88 mg, 0.320 mmol). The reaction mixture was heated at 80 °C for 16 h. Upon cooling to room temperature the mixture was concentrated under reduced pressure. Purification by column chromatography (100:0 to 9:1 Hexane:Et$_2$O) yielded title compound **11h** (32.0 mg, 0.0999 mmol, 31%) as a red-orange oil.

δ_H (400 MHz, CDCl$_3$) 7.72-7.68 (1H, m, *Ar*), 7.64-7.61 (1H, m, *Ar*), 7.59-7.54 (2H, m, *Ar*), 7.47 (1H, td, J = 7.5, 1.5 Hz, *Ar*), 7.41 (1H, dd, J = 7.6, 1.4 Hz, *Ar*), 7.39-7.34 (3H, m, *Ar*), 3.45-3.37 (1H, m, C(9)*H*), 3.36 (1H, s, C(8)*H*H), 3.34 (1H, d, J = 1.5 Hz, C(8)*H*H), 2.77-2.68 (1H, m, C(13)*H*H), 2.63-2.55 (1H, m, C(13)*H*H), 2.15-2.07 (1H, m, C(10)*H*H), 1.96-1.87 (1H, m, C(12)*H*H), 1.85-1.77 (1H, m, C(11)*H*H), 1.65-1.56 (1H, m, C(12)*H*H), 1.51-1.37 (2H, m, C(10)*H*H and C(11)*H*H); **δ_C** (101 MHz, CDCl$_3$) 200.8, 141.1, 133.9, 131.7, 131.2, 128.9, 128.6, 128.5, 122.9, 121.4, 95.0, 88.3, 48.2, 37.8, 34.3, 29.3, 27.0, 25.8; **ν_{max}**/ cm^{-1} (neat) 3980, 3902, 3855, 3820, 3746, 3690, 3676, 3648, 3567, 3484,3371, 3060, 2926, 2849, 2668, 2357, 2343, 2214, 2160, 2085, 2058, 2013, 1987, 1813, 1683, 1590, 1560, 1541, 1493, 1473, 1440,

1397, 1361, 1344, 1286, 1209, 1177, 1096, 1069, 1045, 1024, 990, 965, 925; **m/z HRMS** (ESI$^+$) [M + H]$^+$ $C_{21}H_{20}NO^{23}NaS^+$ calc. 343.1127, found 343.1128.

2-(1,1-Dioxidotetrahydro-2*H*-thiopyran-2-yl)-1-(2-(phenylethynyl)phenyl)ethan-1-one (11i)

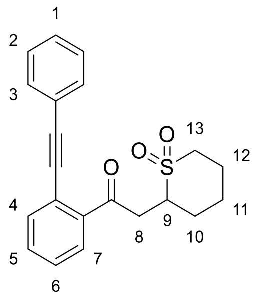

According to a modified procedure,[53] **11h** (28.9 mg, 0.090 mmol) was dissolved in anhydrous CH_2Cl_2 (0.6 mL) in a microwave vial. *m*-CPBA (47 g, 0.270 mmol) was introduced. The mixture was stirred under an argon atmosphere at r.t. for 3 h. The reaction was diluted with CH_2Cl_2 (2 mL) and washed with sat. aq. $NaHCO_3$ (2 mL). The aqueous layer was extracted with CH_2Cl_2 (5 × 2 mL). The combined organics were washed with brine (5 mL), dried over $MgSO_4$, filtered and then concentrated under reduced pressure. Purification by column chromatography (6:4 Petrol:EtOAc) yielded title compound **11i** (26.2 mg, 0.074 mmol, 83%) as a yellow-orange oil.

δ_H (400 MHz, $CDCl_3$) 7.77 (1H, dd, *J* = 8.0, 1.2 Hz, *Ar*), 7.69-7.57 (3H, m, *Ar*), 7.51 (1H, td, *J* = 7.6, 1.4 Hz, *Ar*), 7.43 (1H, dd, *J* = 7.7, 1.4 Hz, *Ar*), 7.43-7.33 (3H, m, *Ar*), 3.99-3.89 (1H, m, C(8)*H*H), 3.73-3.64 (1H, m, C(9)*H*), 3.54-3.44 (1H, m, C(8)H*H*), 3.17-3.09 (1H, m, C(13)*H*H), 3.05-2.94 (1H, m, C(13)H*H*), 2.32-2.22 (1H, m, C(10)*H*H), 2.14-2.02 (2H, m, C(12)H_2), 1.90-1.74 (2H, m, C(10)H*H* and C(11)*H*H), 1.66-1.51 (1H, m, C(11)H*H*); **δ_C** (101 MHz, $CDCl_3$) 197.9, 139.5, 134.4, 132.0, 131.9, 129.0, 128.8, 128.6, 128.5, 122.7, 122.0, 95.9, 88.1, 57.8, 52.1, 37.5, 30.4, 24.5, 24.2; **ν_{max}**/ cm^{-1} (neat) 3386, 3061, 2928, 2850, 2214, 1681, 1590, 1561, 1493, 1473, 1442, 1404, 1349, 1317, 1284, 1224, 1207, 1187, 1163, 1126, 1071,

1025, 1014, 991, 951, 927; **HRMS** (ESI^+) $[M + H]^+$ $C_{21}H_{20}O_3S^+$ calc. 353.1206, found 353.1201.

2-((3-Phenylisoquinolin-1-yl)methyl)tetrahydro-2*H*-thiopyran 1,1-dioxide (11j)

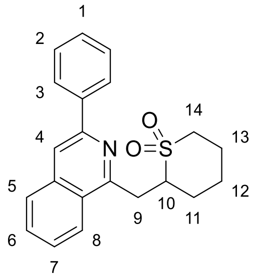

According to a modified procedure,[54] to a pre-dried flask was added ammonium acetate (27.4 mg, 0.360 mmol, 5.50 equiv.), $AgNO_3$ (9 mg, 0.053 mmol, 0.800 equiv.) and compound **11i** (22.5 mg, 0.064 mmol, 1.00 equiv.). Following this, *t*-BuOH (0.53 ml) was introduced and the mixture stirred at 60 °C for 16 h, under nitrogen. Upon cooling, the reaction was quenched with sat. aq. $NaHCO_3$ and stirred for an additional 2 h. The mixture was next filtered through a cotton plug and washed with EtOAc (10-20 mL). The filtrate was then dried over anhydrous $MgSO_4$, filtered and concentrated under reduced pressure. Purification by column chromatography (7:3 then 6:4 Pentane:EtOAc) yielded title compound **11j** (5.8 mg, 0.017 mmol, 27%) as a yellow oil.

$\boldsymbol{\delta_H}$ (400 MHz, $CDCl_3$) 8.22-8.13 (3H, m, *Ar*), 8.00 (1H, s, *Ar*), 7.89 (1H, d, *J* = 8.1 Hz, *Ar*), 7.74-7.65 (1H, m, *Ar*), 7.65-7.57 (1H, m, *Ar*), 7.55-7.48 (2H, m, *Ar*), 7.47-7.38 (1H, m, *Ar*), 4.17 (1H, dd, *J* = 15.6, 2.4 Hz, C(9)*H*), 4.05-3.93 (1H, m, C(10)*H*), 3.50 (1H, dd, *J* = 15.6, 10.7 Hz, C(9)*H*), 3.27-3.16 (1H, m, C(14)*H*), 3.13-3.01 (1H, m, C(14)*H*), 2.39-2.29 (1H, m, C(11)*H*), 2.21-2.12 (2H, m, C(13)*H*), 1.99-1.84 (2H, m, C(11)*H* and C(12)*H*), 1.62-1.45 (1H, m, C(12)*H*); $\boldsymbol{\delta_C}$ (101 MHz, $CDCl_3$) 156.1, 137.2, 130.4, 128.9, 128.7, 128.0, 127.6, 126.8, 124.7, 60.5, 52.2, 30.0, 24.7, 24.2; $\boldsymbol{\nu_{max}}$/ cm^{-1} (neat) 2924, 2852, 2367, 1698, 1621, 1591, 1570,

1499, 1443, 1304, 1283, 1212, 1164, 1126, 1028, 927, 908; **HRMS** (ESI$^+$) [M + H]$^+$ $C_{21}H_{22}NO_2S^+$ calc. 352.1366, found 352.1364.

8. References

[1] B. Bieszczad, M. Barbasiewicz, *Chemistry - A European Journal* **2015**, *21*, 10322-10325.

[2] R. J. Altenbach, M. E. Brune, S. A. Buckner, M. J. Coghlan, A. V. Daza, A. Fabiyi, M. Gopalakrishnan, R. F. Henry, A. Khilevich, M. E. Kort, I. Milicic, V. E. Scott, J. C. Smith, K. L. Whiteaker, W. A. Carroll, *Journal of Medicinal Chemistry* **2006**, *49*, 6869-6887.

[3] S. Jogula, B. Dasari, M. Khatravath, G. Chandrasekar, S. S. Kitambi, P. Arya, *European Journal of Organic Chemistry* **2013**, *2013*, 5036-5040.

[4] S. Nag, L. Lehmann, G. Kettschau, M. Toth, T. Heinrich, A. Thiele, A. Varrone, C. Halldin, *Bioorganic & Medicinal Chemistry* **2013**, *21*, 6634-6641.

[5] P. R. Likhar, M. Praveen Kumar, A. K. Bandyopadhyay, *Synlett* **2001**, *2001*, 0836-0838.

[6] R. Ben-Othman, M. Othman, S. Coste, B. Decroix, *Tetrahedron* **2008**, *64*, 559-567.

[7] C. González-Rodríguez, R. J. Pawley, A. B. Chaplin, A. L. Thompson, A. S. Weller, M. C. Willis, *Angewandte Chemie International Edition* **2011**, *50*, 5134-5138.

[8] M. Arambasic, M. K. Majhail, R. N. Straker, J. D. Neuhaus, M. C. Willis, *Chemical Communications* **2019**, *55*, 2757-2760.

[9] J. F. Hooper, R. D. Young, A. S. Weller, M. C. Willis, *Chemistry - A European Journal* **2013**, *19*, 3125-3130.

[10] M. Mohankumar, B. Chattopadhyay, R. Hadji, L. Sanguinet, A. R. Kennedy, V. Lemaur, J. Cornil, O. Fenwick, P. Samorì, Y. Geerts, *ChemPlusChem* **2019**, *84*, 1263-1269.

[11] M. K. Majhail, P. M. Ylioja, M. C. Willis, *Chemistry - A European Journal* **2016**, *22*, 7879-7884.

[12] P.-H. Li, L.-Z. Yu, X.-Y. Zhang, M. Shi, *Organic Letters* **2018**, *20*, 4516-4520.

[13] S. Zheng, G. Lingyue, M. J. H. Ong, D. Jacquemin, A. Romieu, J.-A. Richard, R. Srinivasan, *Organic & Biomolecular Chemistry* **2019**, *17*, 4291-4300.

[14] F. Schaufelberger, O. Ramström, *Chemistry - A European Journal* **2015**, *21*, 12735-12740.

[15] J. Barwick-Silk, S. Hardy, M. C. Willis, A. S. Weller, *Journal of the American Chemical Society* **2018**, *140*, 7347-7357.

[16] T. J. Coxon, M. Fernández, J. Barwick-Silk, A. I. McKay, L. E. Britton, A. S. Weller, M. C. Willis, *Journal of the American Chemical Society* **2017**, *139*, 10142-10149.

[17] V. Theodorou, K. Skobridis, A. G. Tzakos, V. Ragoussis, *Tetrahedron Letters* **2007**, *48*, 8230-8233.

[18] M. Schade, B. Merla, B. Lesch, M. Wagener, S. Timmermanns, K. Pletinckx, T. Hertrampf, *Journal of Medicinal Chemistry* **2020**, *63*, 11801-11808.

[19] J. Bucher, T. Wurm, K. S. Nalivela, M. Rudolph, F. Rominger, A. S. K. Hashmi, *Angewandte Chemie International Edition* **2014**, *53*, 3854-3858.

[20] J. Adrio, J. C. Carretero, *Journal of the American Chemical Society* **2007**, *129*, 778-779.

[21] T. Harada, K. Muramatsu, K. Mizunashi, C. Kitano, D. Imaoka, T. Fujiwara, H. Kataoka, *The Journal of Organic Chemistry* **2008**, *73*, 249-258.

[22] M. W. C. Robinson, A. M. Davies, R. Buckle, I. Mabbett, S. H. Taylor, A. E. Graham, *Organic & Biomolecular Chemistry* **2009**, *7*, 2559.

[23] S. Kulasegaram, R. J. Kulawiec, *The Journal of Organic Chemistry* **1997**, *62*, 6547-6561.

[24] R. C. Durley, M. L. Grapperhaus, B. S. Hickory, M. A. Massa, J. L. Wang, D. P. Spangler, D. A. Mischke, B. L. Parnas, Y. M. Fobian, N. P. Rath, D. D. Honda, M. Zeng, D. T. Connolly, D. M.

Heuvelman, B. J. Witherbee, M. A. Melton, K. C. Glenn, E. S. Krul, M. E. Smith, J. A. Sikorski, *Journal of Medicinal Chemistry* **2002**, *45*, 3891-3904.
[25] V. Gudla, R. Balamurugan, *The Journal of Organic Chemistry* **2011**, *76*, 9919-9933.
[26] A. J. Cagnoni, O. Varela, J. Kovensky, M. L. Uhrig, *Organic & Biomolecular Chemistry* **2013**, *11*, 5500.
[27] R. Grigg, V. Sridharan, M. Thornton-Pett, J. Wang, J. Xu, J. Zhang, *Tetrahedron* **2002**, *58*, 2627-2640.
[28] Q. Yang, Y.-Y. Lai, W.-J. Xiao, H. Alper, *Tetrahedron Letters* **2008**, *49*, 7334-7336.
[29] P. R. Auburn, P. B. Mackenzie, B. Bosnich, *Journal of the American Chemical Society* **1985**, *107*, 2033-2046.
[30] A. Basak, S. C. Ghosh, A. K. Das, V. Bertolasi, *Organic & Biomolecular Chemistry* **2005**, *3*, 4050.
[31] S. J. Welsch, M. Umkehrer, C. Kalinski, G. Ross, C. Burdack, J. Kolb, M. Wild, A. Ehrlich, L. A. Wessjohann, *Tetrahedron Letters* **2015**, *56*, 1025-1029.
[32] T. S. Cooper, A. S. Larigo, P. Laurent, C. J. Moody, A. K. Takle, *Org. Biomol. Chem.* **2005**, *3*, 1252-1262.
[33] L. A. Perego, R. Blieck, A. Groué, F. Monnier, M. Taillefer, I. Ciofini, L. Grimaud, *ACS Catalysis* **2017**, *7*, 4253-4264.
[34] T. Rizk, E. J. F. Bilodeau, A. M. Beauchemin, *Angewandte Chemie International Edition* **2009**, *48*, 8325-8327.
[35] B. Janza, A. Studer, *Organic Letters* **2006**, *8*, 1875-1878.
[36] M. Arambasic, J. F. Hooper, M. C. Willis, *Organic Letters* **2013**, *15*, 5162-5165.
[37] K. Asano, S. Matsubara, A. Matsumoto, *Synlett* **2015**, *26*, 1872-1874.
[38] J.-L. Zhan, M.-W. Wu, D. Wei, B.-Y. Wei, Y. Jiang, W. Yu, B. Han, *ACS Catalysis* **2019**, *9*, 4179-4188.
[39] D. M. McKinnon, K. R. Lee, *Canadian Journal of Chemistry* **1988**, *66*, 1405-1409.
[40] K. S. Feldman, T. D. Cutarelli, R. Di Florio, *The Journal of Organic Chemistry* **2002**, *67*, 8528-8537.
[41] R. Bell, P. D. Cottam, J. Davies, D. N. Jones, *Journal of the Chemical Society, Perkin Transactions 1* **1981**, 2106.
[42] A. Manikowski, Z. Kolarska, *Synthetic Communications* **2009**, *39*, 3621-3638.
[43] D. P. Kjell, B. J. Slattery, M. J. Semo, *The Journal of Organic Chemistry* **1999**, *64*, 5722-5724.
[44] M. L. Capobianco, E. Marchesi, D. Perrone, M. L. Navacchia, *Bioconjugate Chemistry* **2013**, *24*, 1398-1407.
[45] A. Hatano, N. Terado, Y. Kanno, T. Nakamura, G. Kawai, *Synthetic Communications* **2019**, *49*, 136-145.
[46] L. A. Aronica, L. Giannotti, S. Giuntini, A. M. Caporusso, *European Journal of Organic Chemistry* **2014**, *2014*, 6858-6862.
[47] S. J. Hein, H. Arslan, I. Keresztes, W. R. Dichtel, *Organic Letters* **2014**, *16*, 4416-4419.
[48] S. Prescher, F. Polzer, Y. Yang, M. Siebenbürger, M. Ballauff, J. Yuan, *Journal of the American Chemical Society* **2014**, *136*, 12-15.
[49] F. Pape, L. T. Brechmann, J. F. Teichert, *Chemistry – A European Journal* **2018**.
[50] Y.-H. Peng, F.-Y. Liao, C.-T. Tseng, R. Kuppusamy, A.-S. Li, C.-H. Chen, Y.-S. Fan, S.-Y. Wang, M.-H. Wu, C.-C. Hsueh, J.-Y. Chang, L.-C. Lee, C. Shih, K.-S. Shia, T.-K. Yeh, M.-S. Hung, C.-C. Kuo, J.-S. Song, S.-Y. Wu, S.-H. Ueng, *Journal of Medicinal Chemistry* **2020**, *63*, 1642-1659.
[51] M. Mori, D. Deodato, M. Kasula, D. M. Ferraris, A. Sanna, A. De Logu, M. Rizzi, M. Botta, *Bioorganic & Medicinal Chemistry Letters* **2018**, *28*, 637-641.
[52] O. Naoki, M. Toru, K. Satoshi, O. Katsuo, *Chemistry Letters* **2001**, *30*, 906-907.
[53] S. Sangeetha, P. Muthupandi, G. Sekar, *Organic Letters* **2015**, *17*, 6006-6009.
[54] V. Reddy, A. S. Jadhav, R. Vijaya Anand, *Organic & Biomolecular Chemistry* **2015**, *13*, 3732-3741.

9. NMR Spectra of Novel Compounds

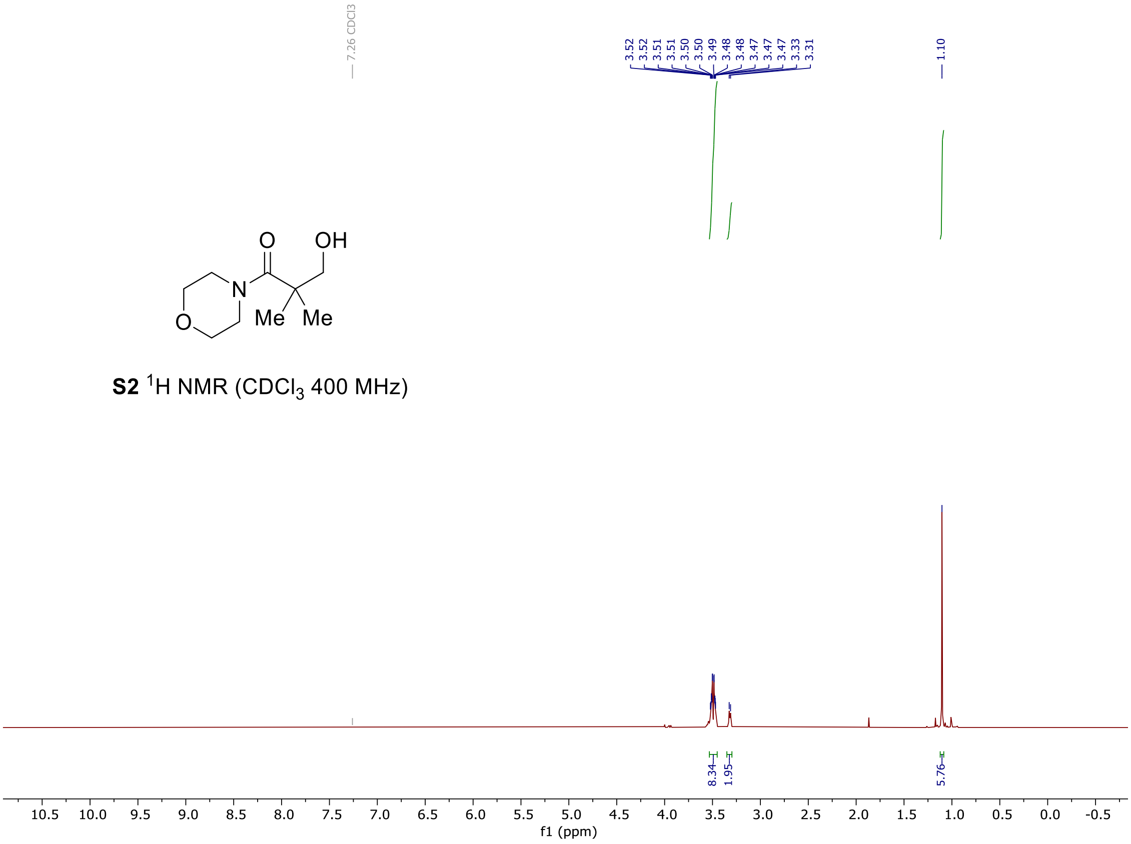

S2 ^{1}H NMR (CDCl$_3$ 400 MHz)

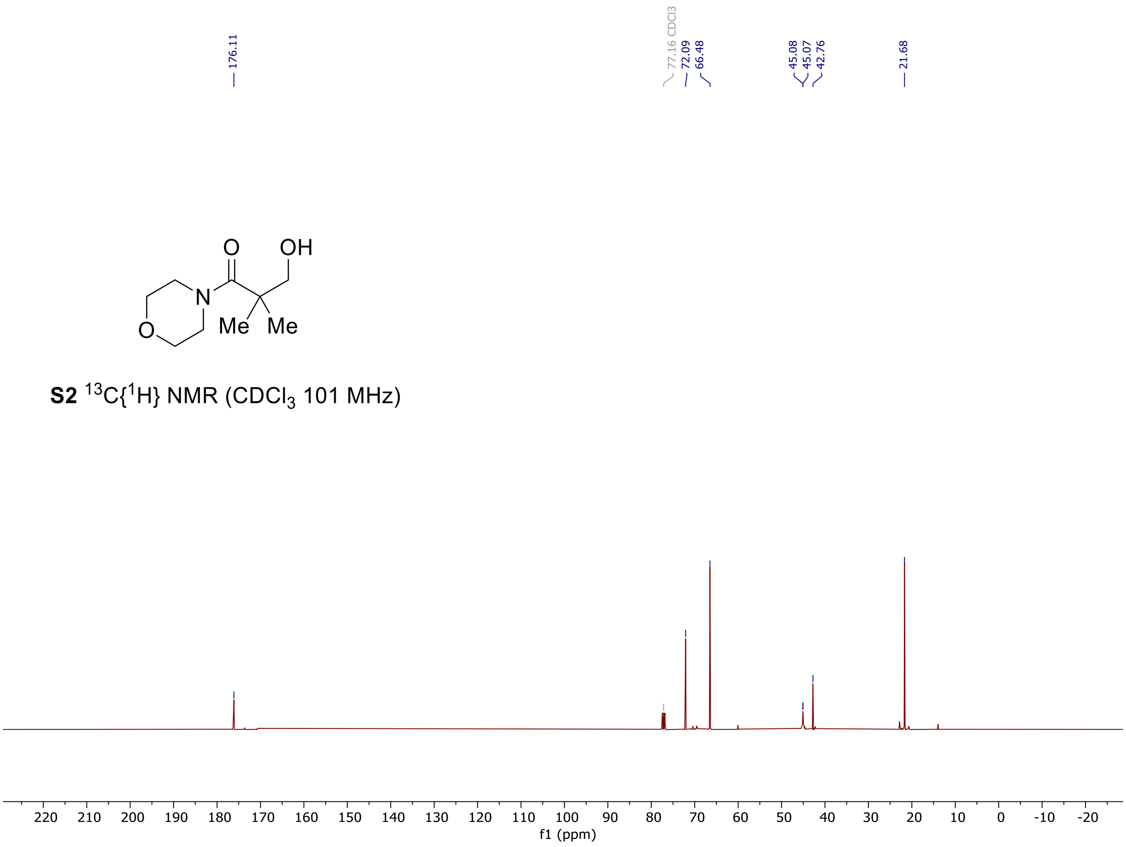

S2 ^{13}C{^{1}H} NMR (CDCl$_3$ 101 MHz)

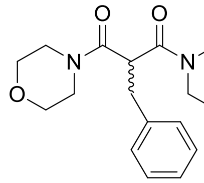

S4 ^{1}H NMR ($CDCl_3$ 400 MHz)

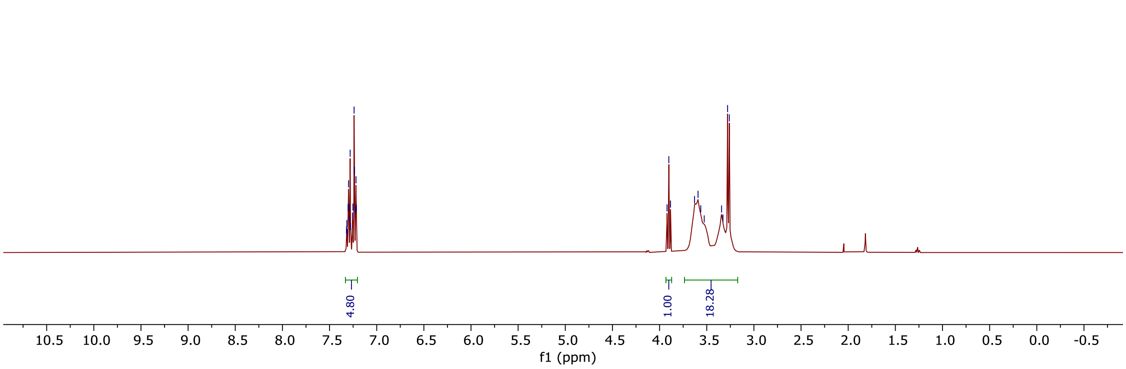

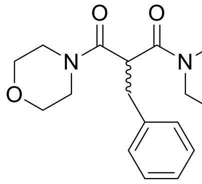

S4 ^{13}C{^{1}H} NMR ($CDCl_3$ 101 MHz)

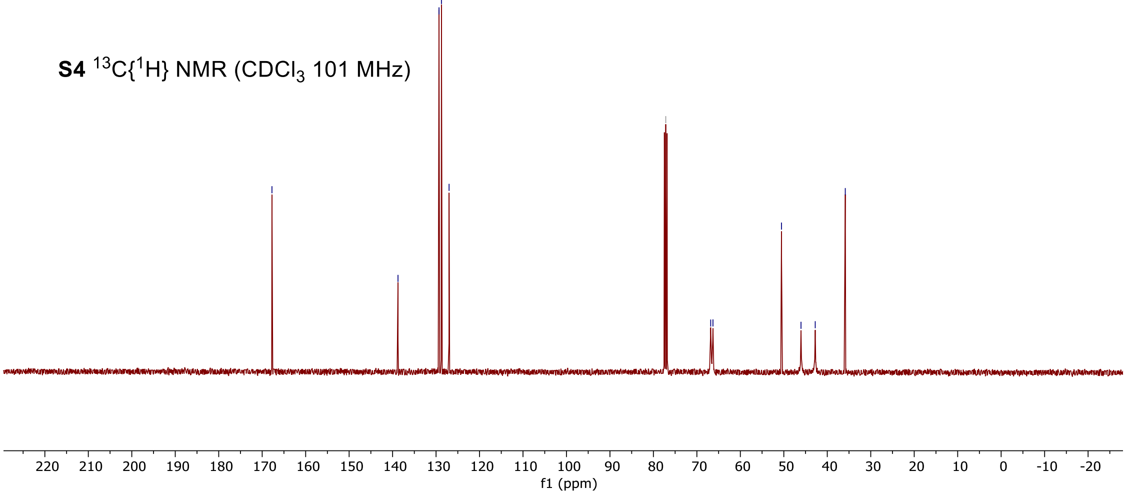

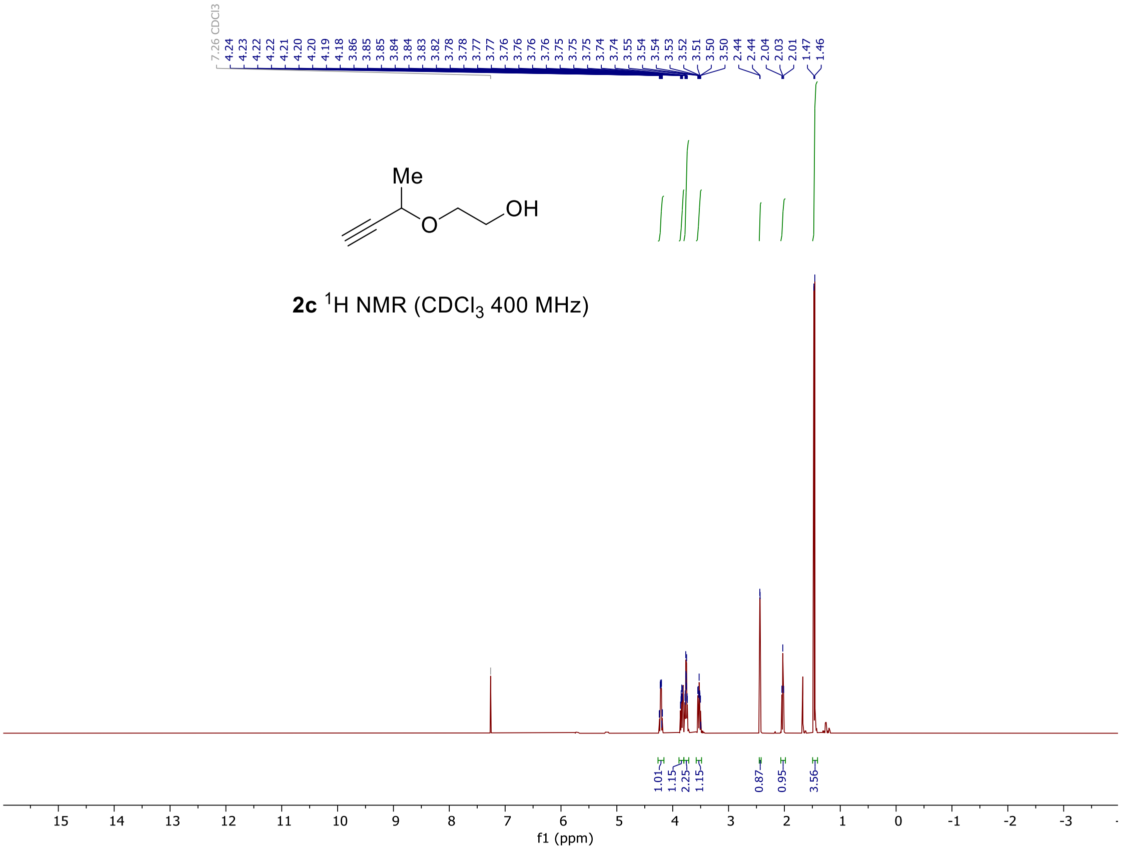

2c ^{1}H NMR ($CDCl_3$ 400 MHz)

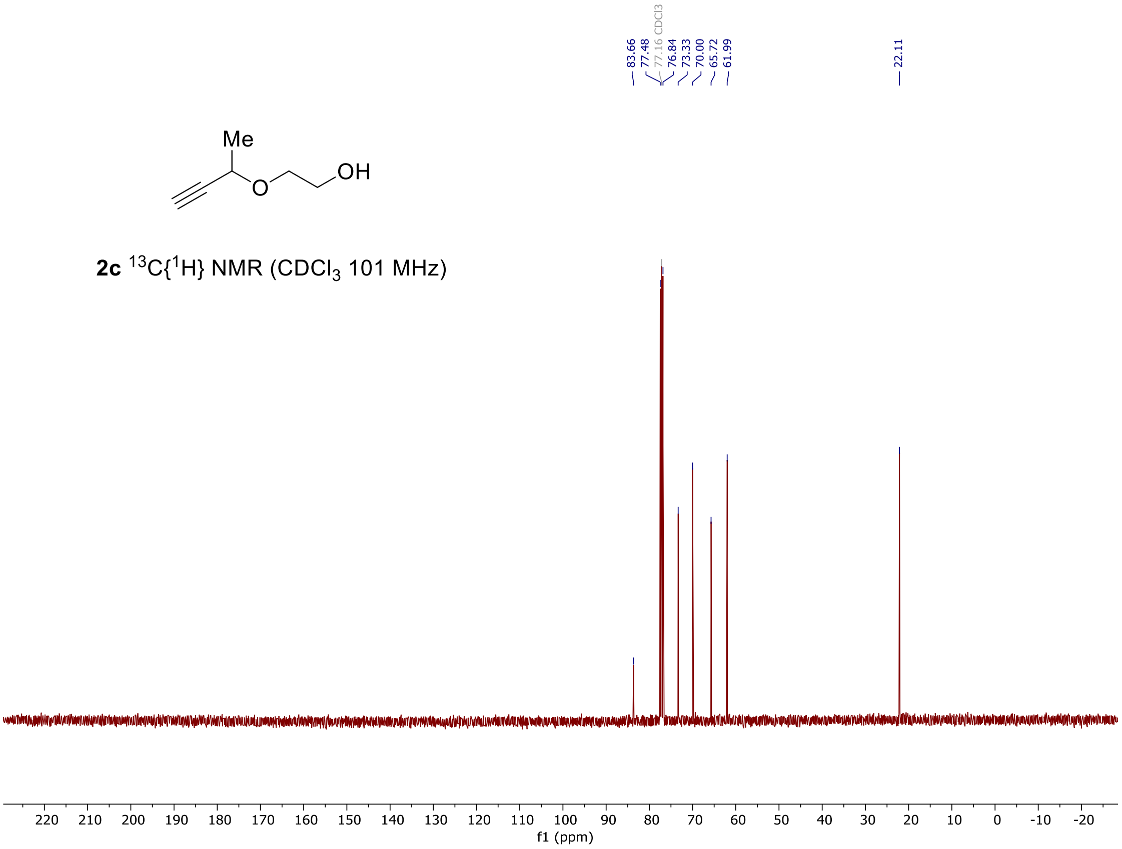

2c $^{13}C\{^{1}H\}$ NMR ($CDCl_3$ 101 MHz)

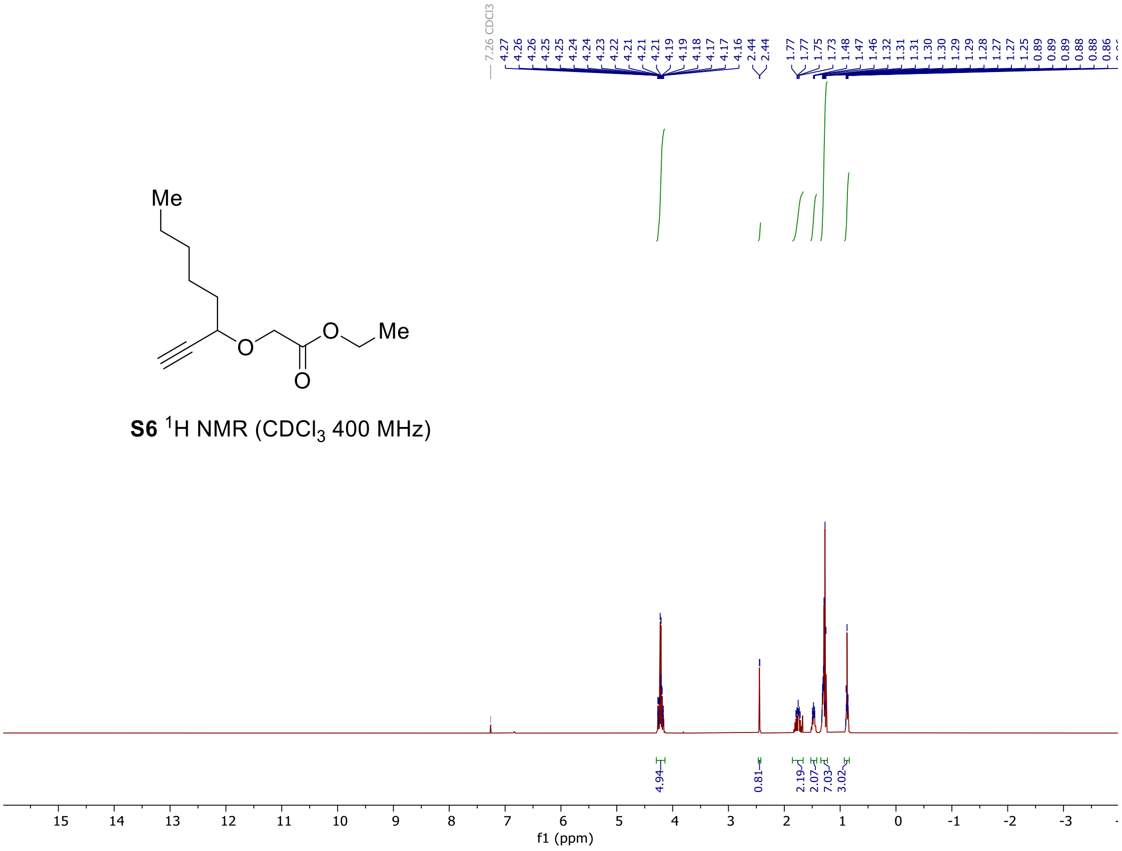

S6 ^{1}H NMR ($CDCl_3$ 400 MHz)

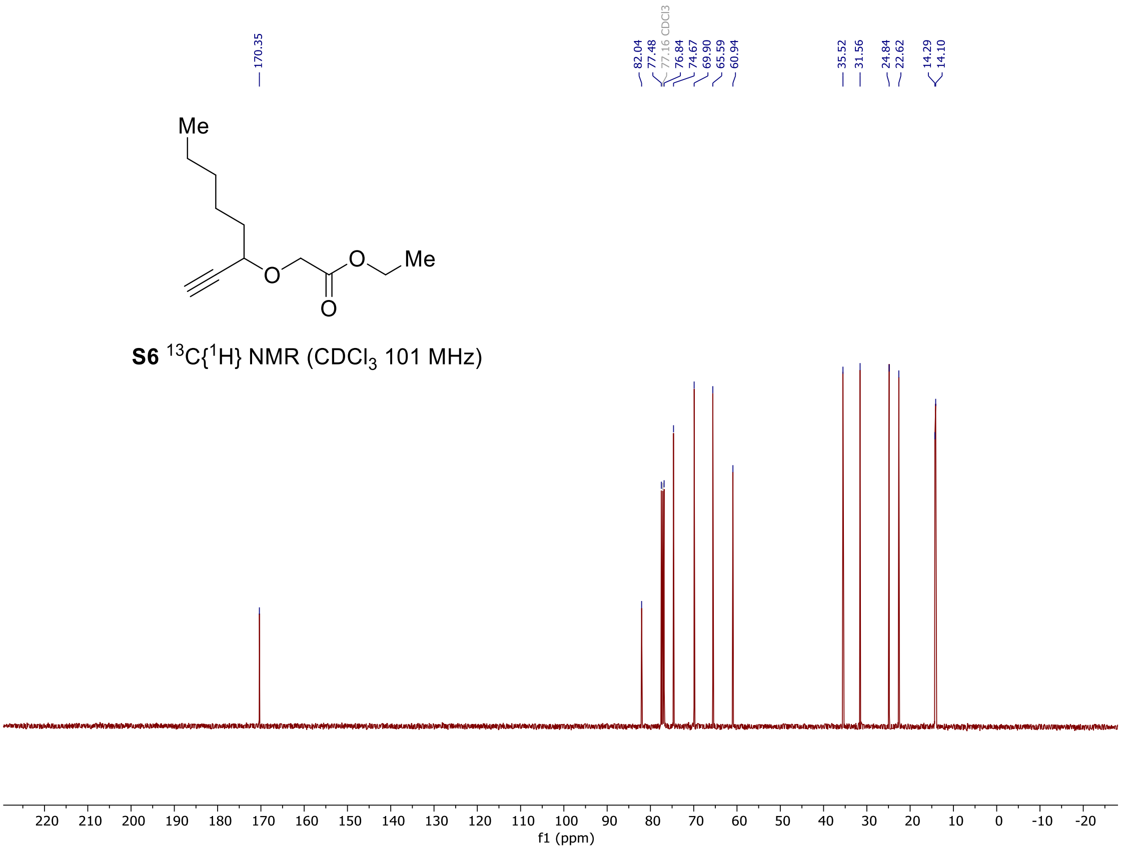

S6 $^{13}C\{^{1}H\}$ NMR ($CDCl_3$ 101 MHz)

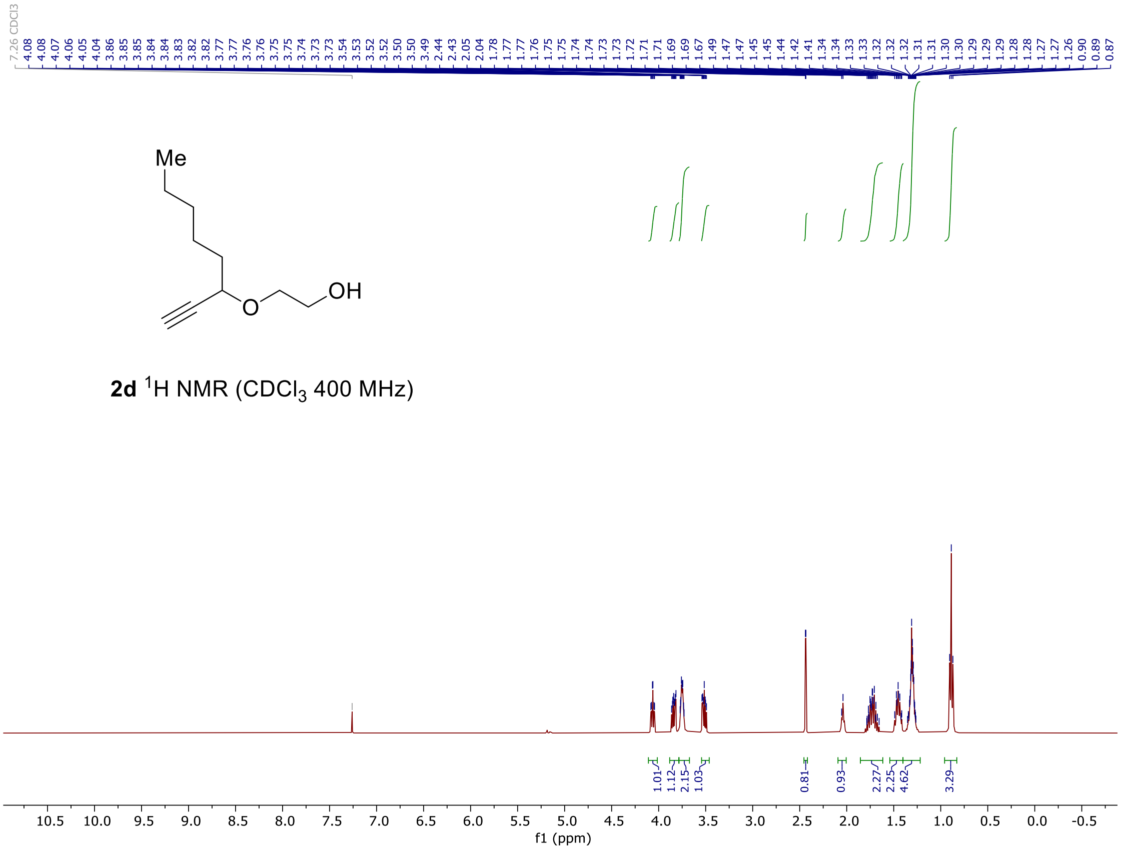

2d ^{1}H NMR ($CDCl_3$ 400 MHz)

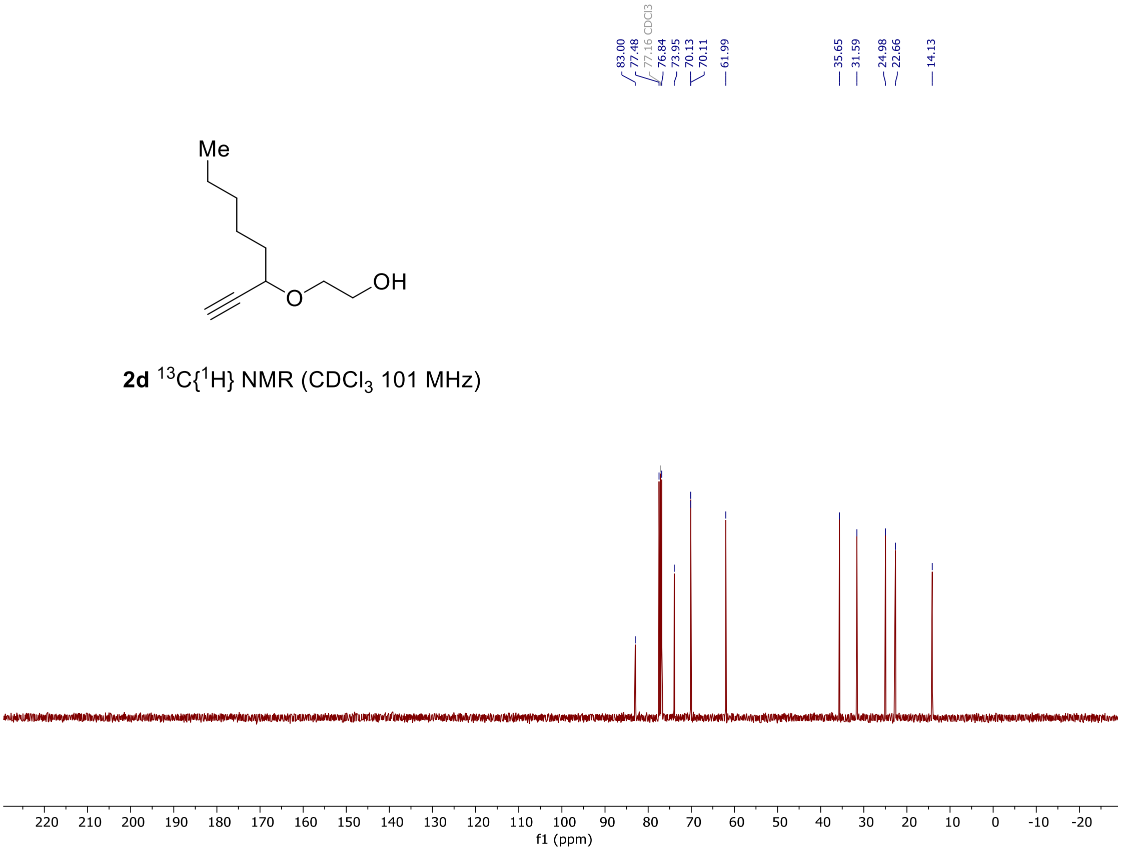

2d ^{13}C{^{1}H} NMR ($CDCl_3$ 101 MHz)

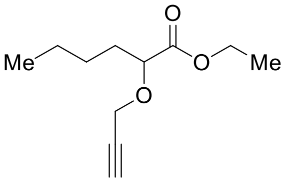

S8 ^{1}H NMR (CDCl$_3$ 400 MHz)

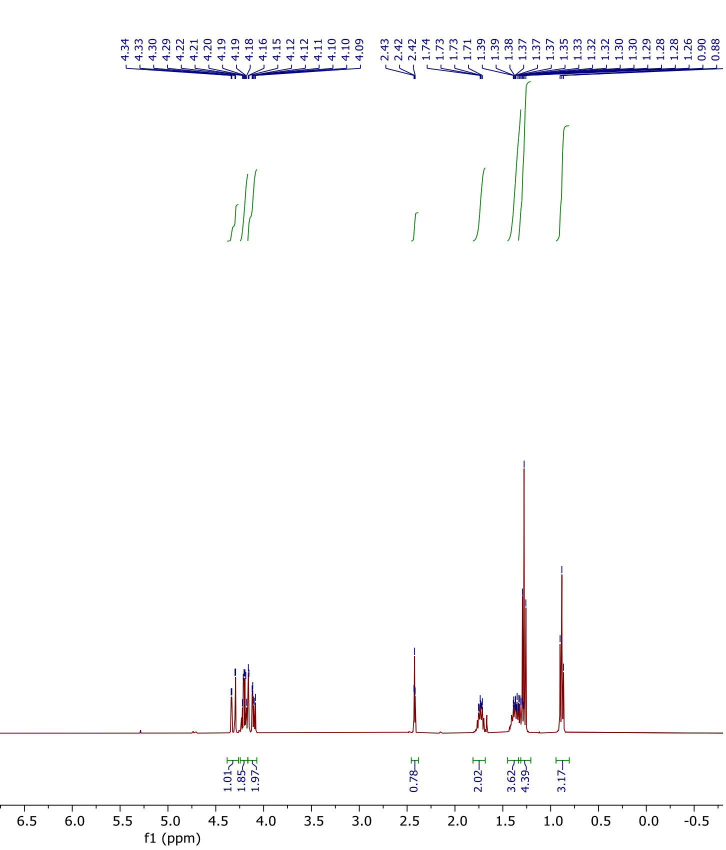

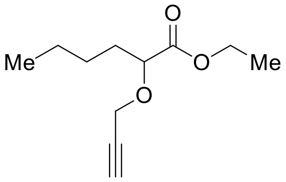

S8 ^{13}C{^{1}H} NMR (CDCl$_3$ 101 MHz)

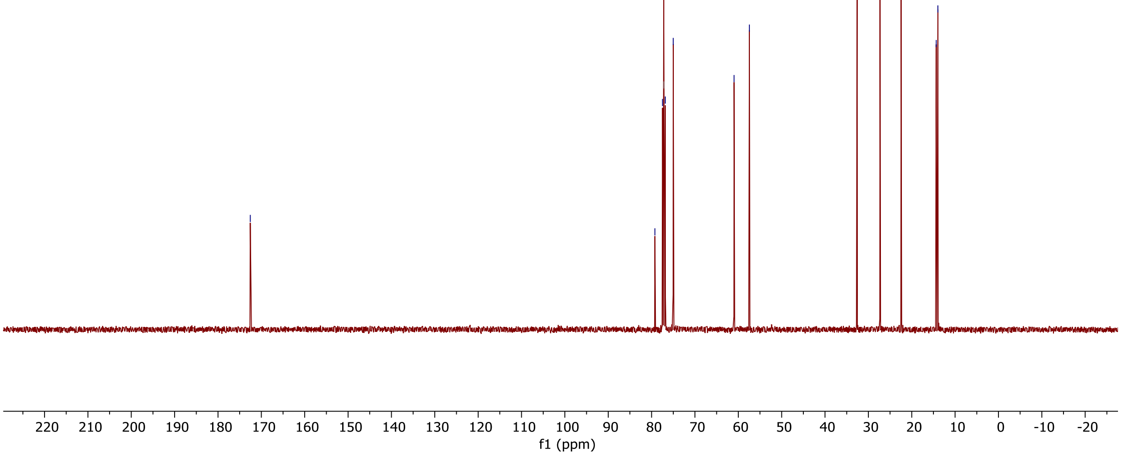

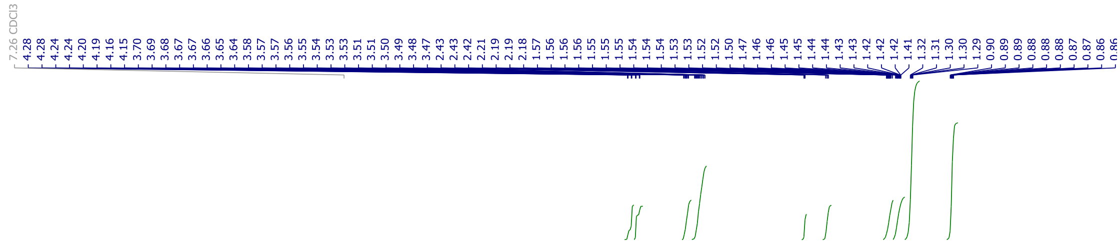

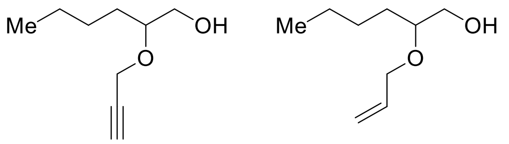

2f and **S9** ^{1}H NMR ($CDCl_3$ 400 MHz)

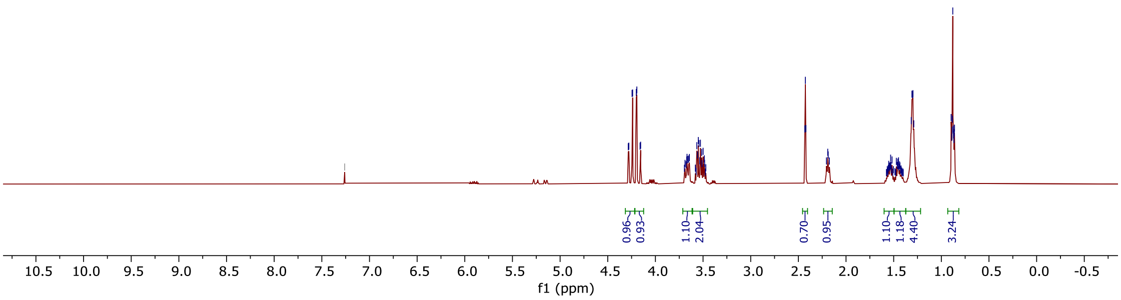

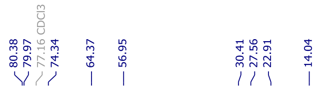

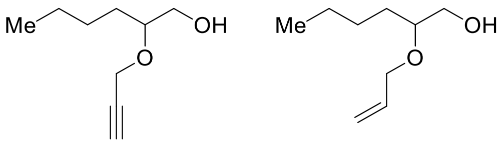

2f and **S9** $^{13}C\{^{1}H\}$ NMR ($CDCl_3$ 101 MHz)

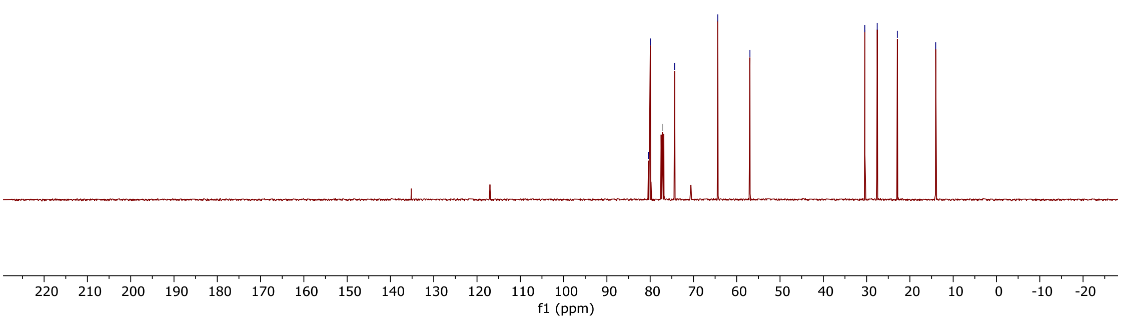

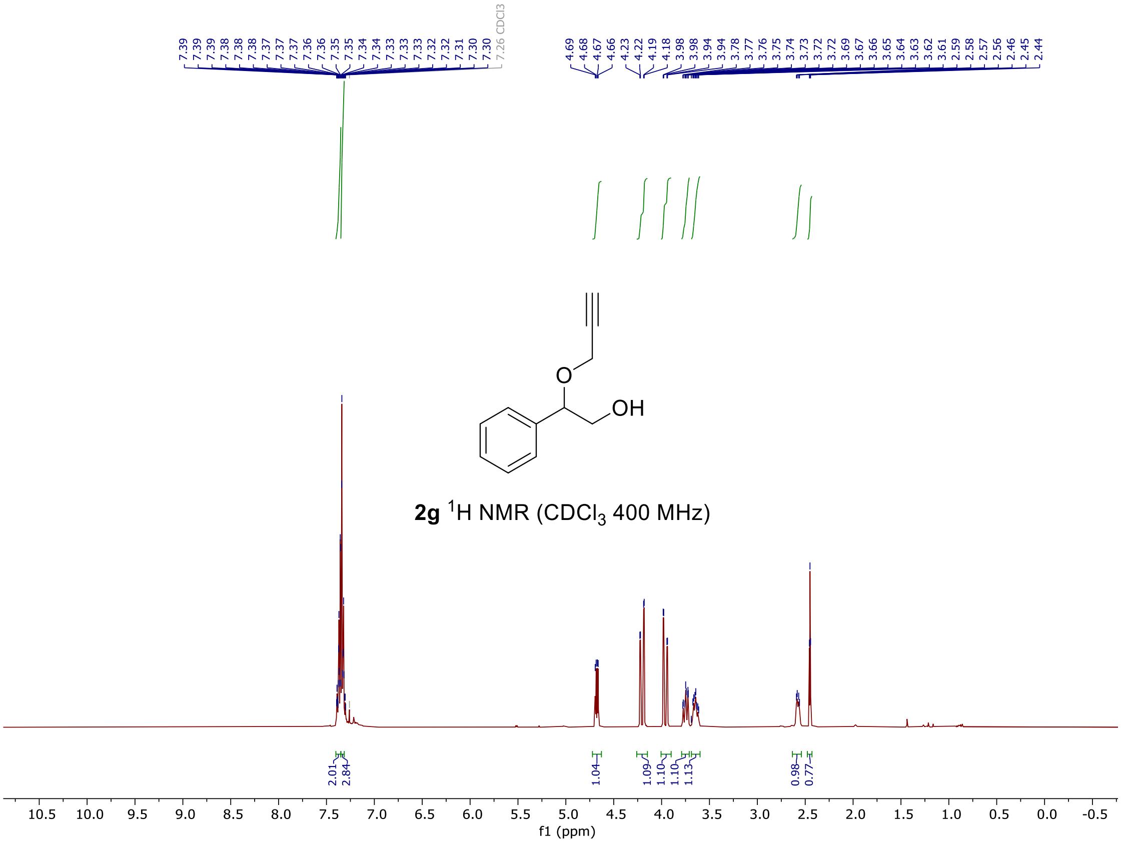

2g ^{1}H NMR ($CDCl_3$ 400 MHz)

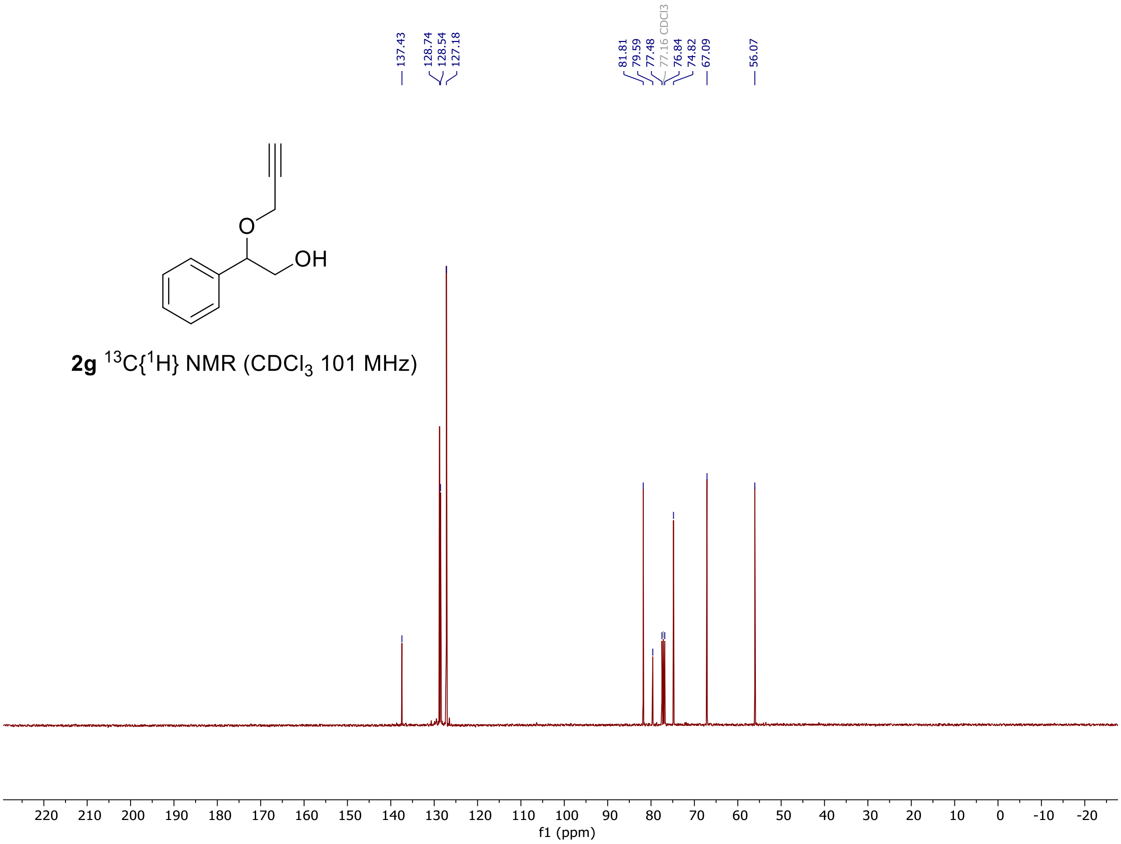

2g $^{13}C\{^{1}H\}$ NMR ($CDCl_3$ 101 MHz)

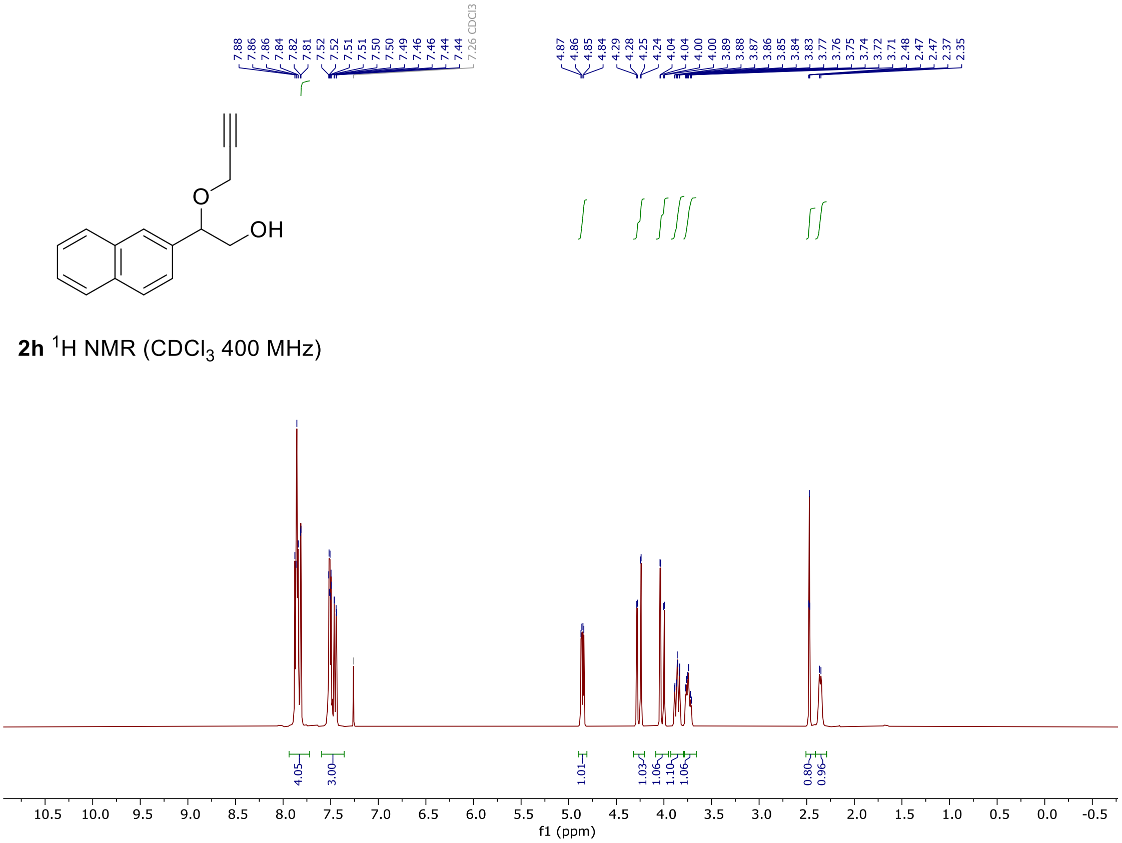
7.88 7.86 7.86 7.84 7.82 7.81 7.52 7.52 7.51 7.51 7.50 7.49 7.46 7.46 7.44 7.44 7.26 CDCl3
4.87 4.86 4.85 4.84 4.29 4.28 4.25 4.24 4.04 4.04 4.00 4.00 3.89 3.88 3.87 3.86 3.85 3.84 3.83 3.77 3.76 3.75 3.74 3.72 3.71 2.48 2.47 2.47 2.37 2.35
OH
O
2h ^{1}H NMR ($CDCl_3$ 400 MHz)
4.05 3.00 1.01 1.03 1.06 1.10 1.06 0.80 0.96
10.5 10.0 9.5 9.0 8.5 8.0 7.5 7.0 6.5 6.0 5.5 5.0 4.5 4.0 3.5 3.0 2.5 2.0 1.5 1.0 0.5 0.0 -0.5
f1 (ppm)

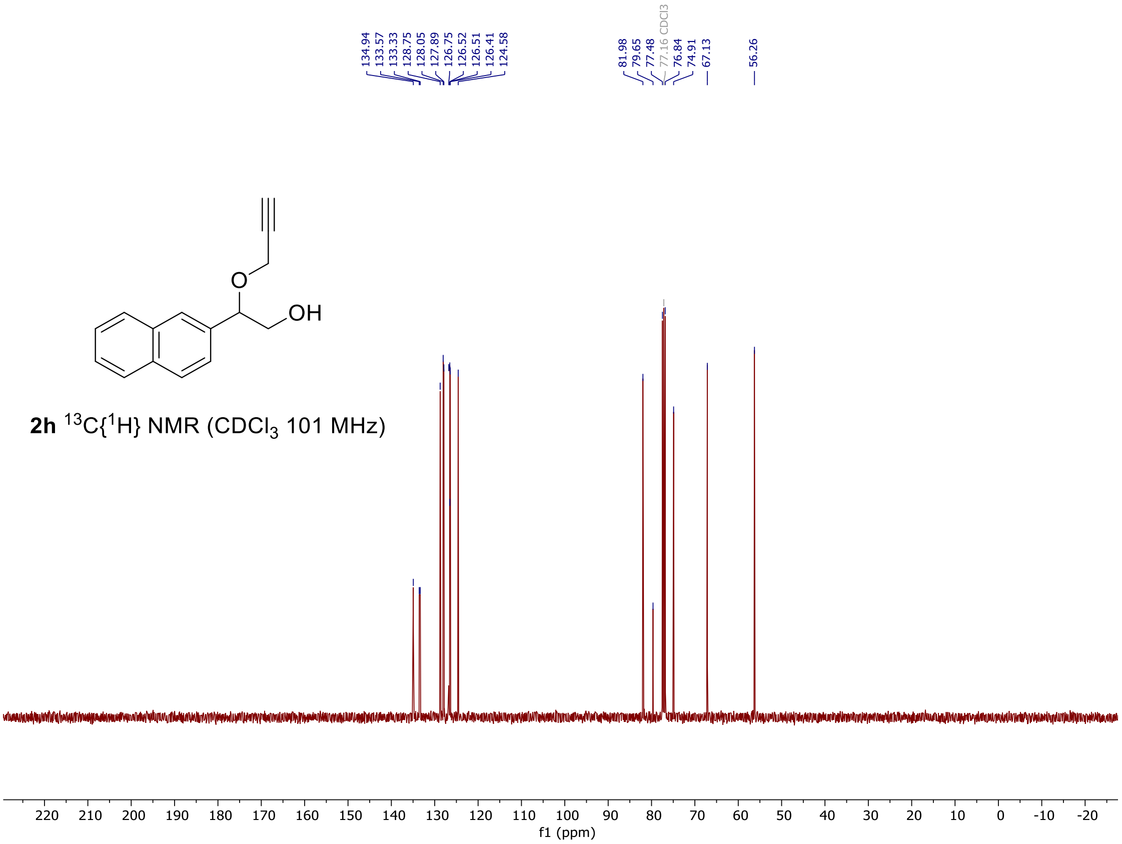
134.94 133.57 133.33 128.75 128.05 127.89 126.75 126.52 126.41 124.58
81.98 79.65 77.48 77.16 CDCl3 76.84 74.91 67.13 56.26
OH
O
2h ^{13}C{^{1}H} NMR ($CDCl_3$ 101 MHz)
220 210 200 190 180 170 160 150 140 130 120 110 100 90 80 70 60 50 40 30 20 10 0 -10 -20
f1 (ppm)

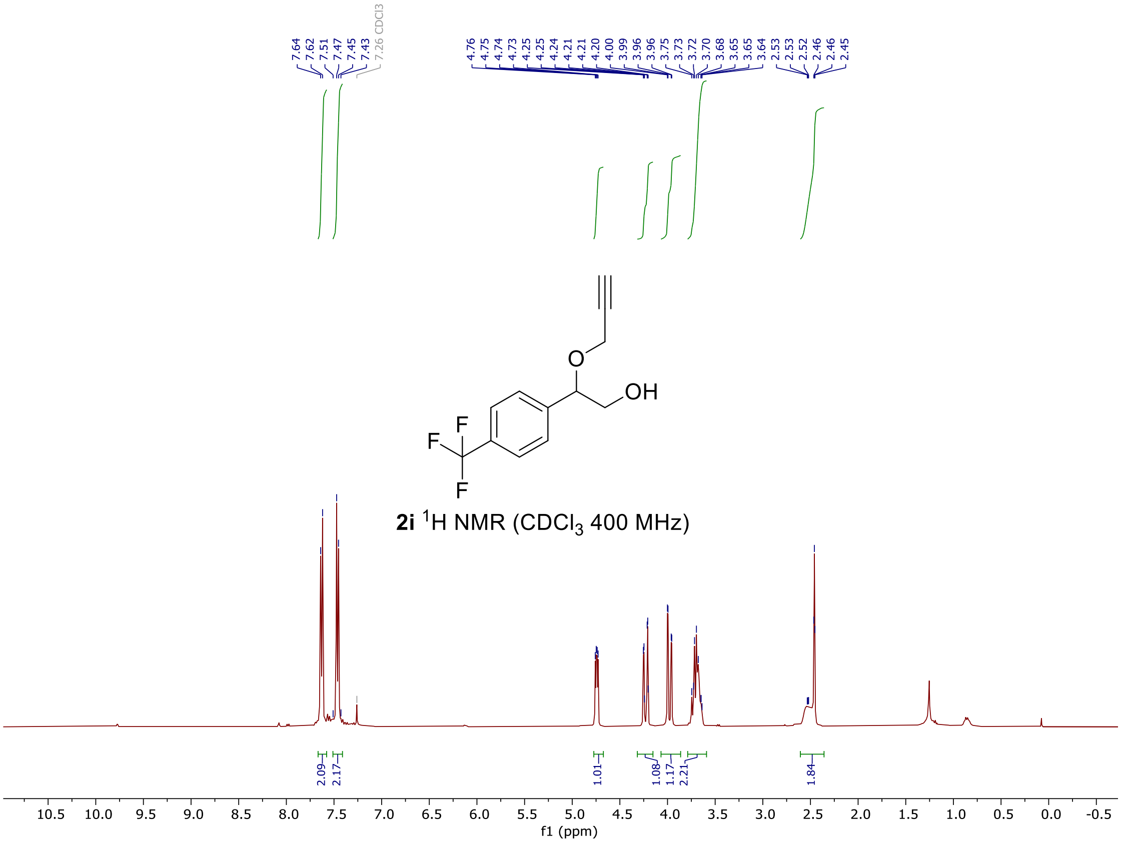
7.64 7.62 7.51 7.47 7.45 7.43 7.26 CDCl3
4.76 4.75 4.74 4.73 4.25 4.25 4.24 4.21 4.21 4.20 4.00 3.99 3.96 3.96 3.75 3.73 3.72 3.70 3.68 3.65 3.65 3.64 2.53 2.53 2.52 2.46 2.46 2.45
OH
O
F
F
F
2i ¹H NMR (CDCl3 400 MHz)
2.09 2.17 1.01 1.08 1.17 2.21 1.84
f1 (ppm)

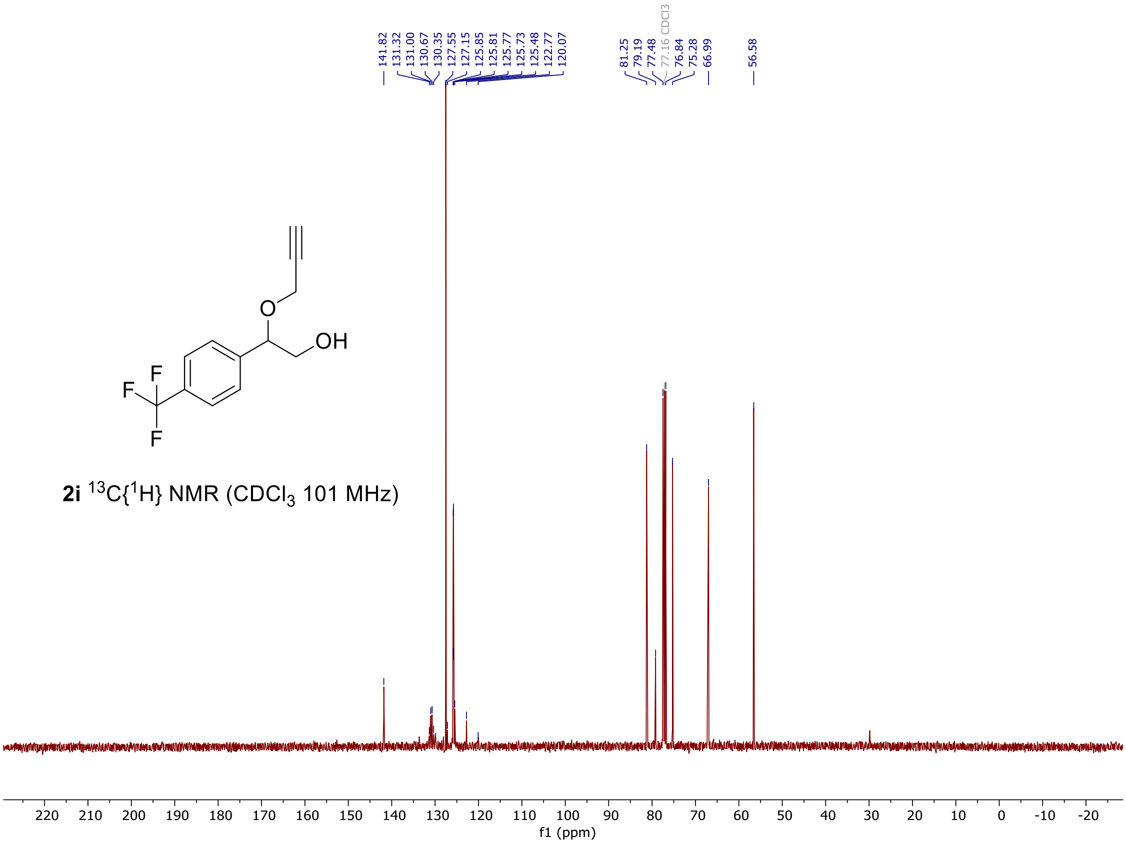
141.82 131.32 131.00 130.67 130.35 127.55 127.15 125.85 125.81 125.77 125.73 125.48 122.77 120.07
81.25 79.19 77.48 77.16 CDCl3 76.84 75.28 66.99
56.58
OH
O
F
F
F
2i ¹³C{¹H} NMR (CDCl3 101 MHz)
f1 (ppm)

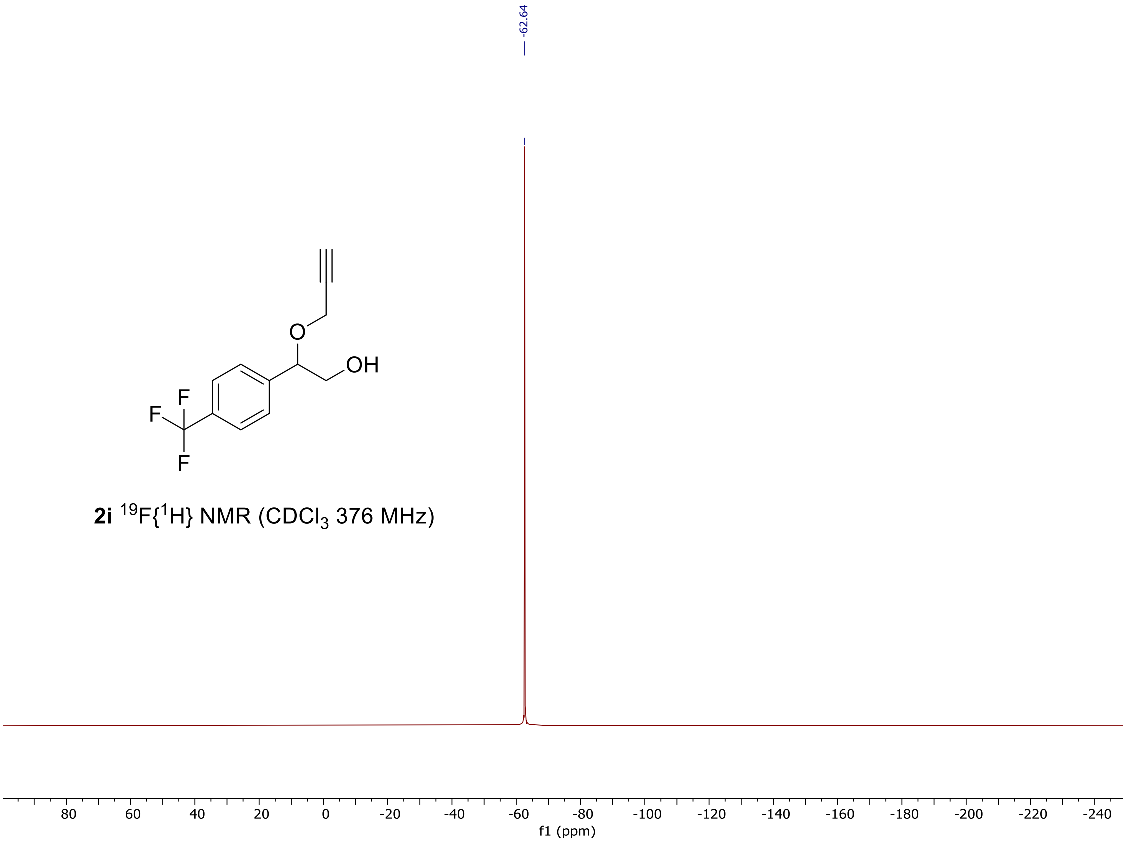
-62.64
OH
O
F
F
F
2i 19F{1H} NMR (CDCl3 376 MHz)
80 60 40 20 0 -20 -40 -60 -80 -100 -120 -140 -160 -180 -200 -220 -240
f1 (ppm)

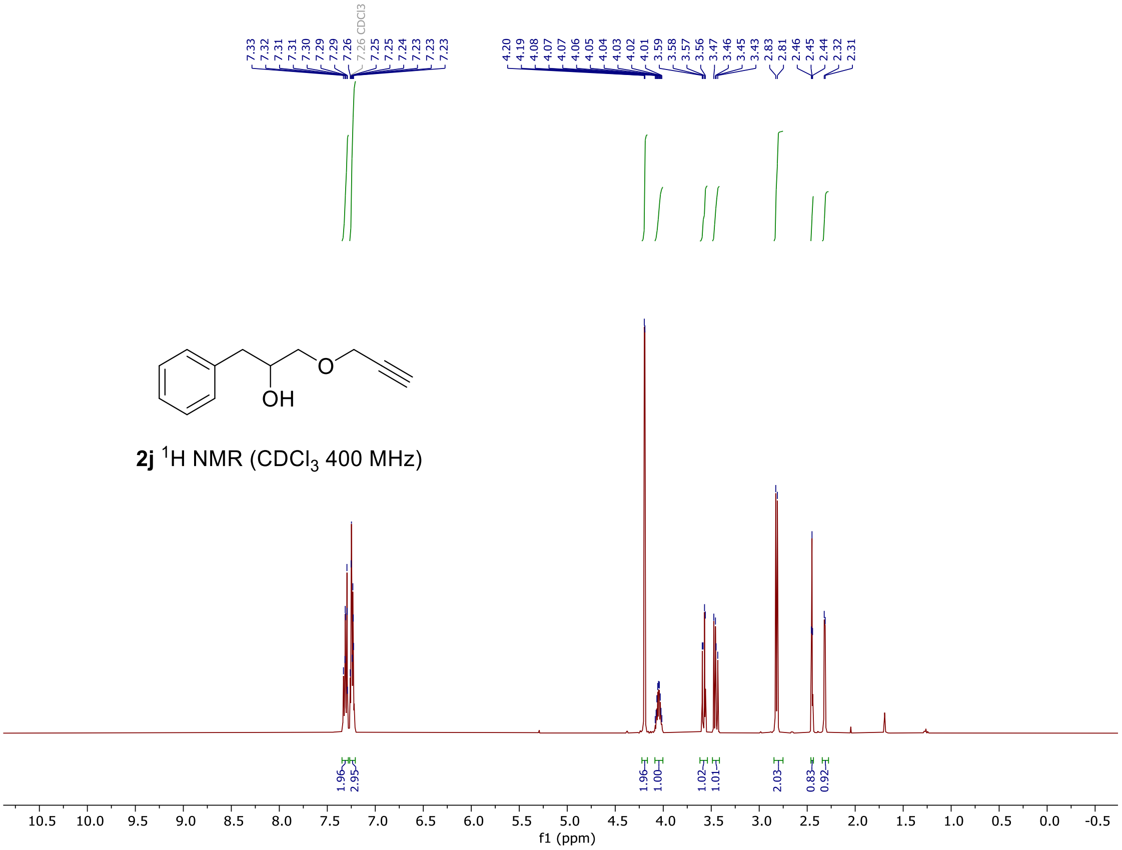

2j ^{1}H NMR ($CDCl_3$ 400 MHz)

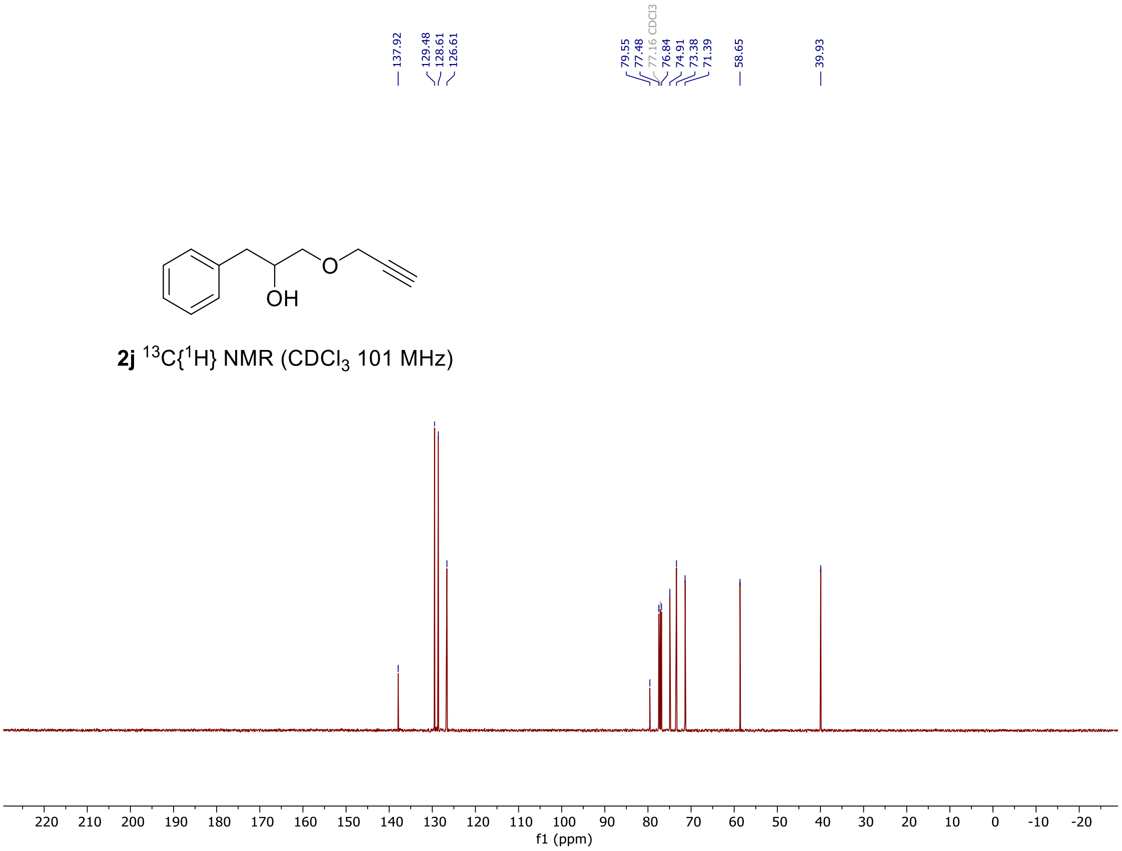

2j ^{13}C{^{1}H} NMR ($CDCl_3$ 101 MHz)

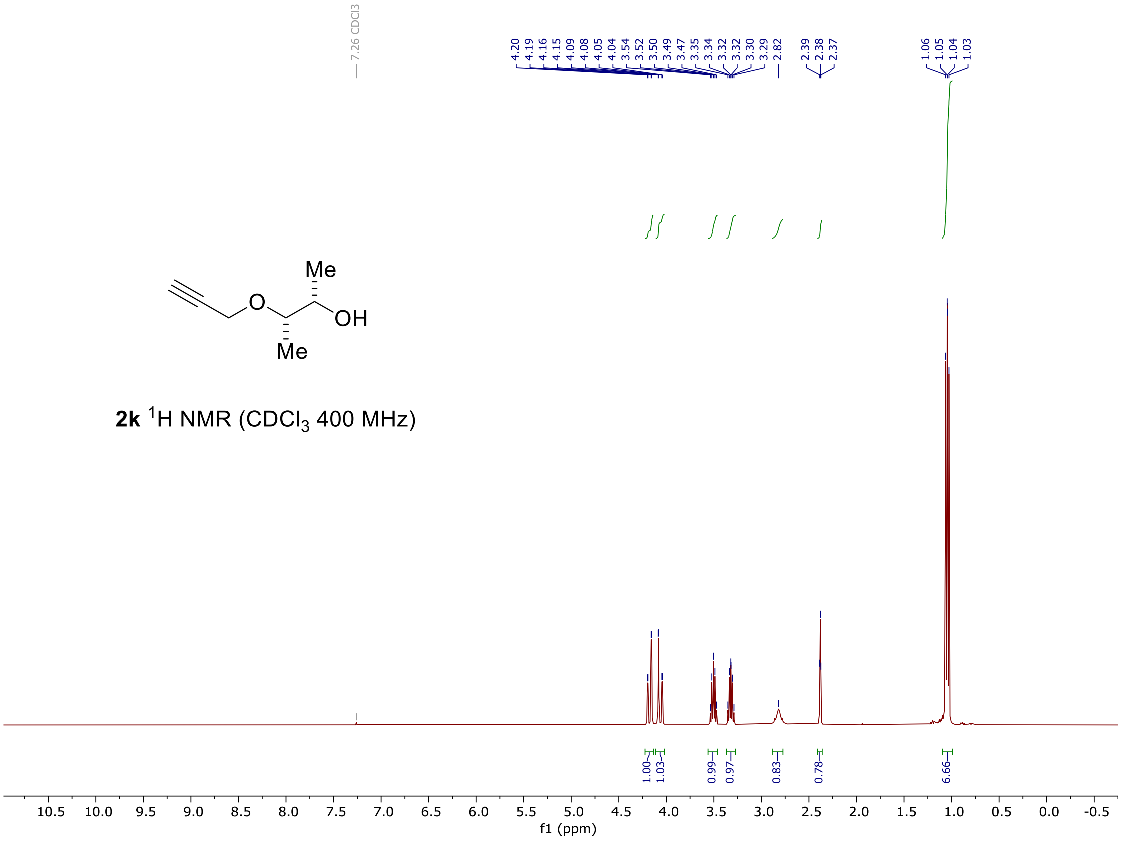
7.26 CDCl3
4.20
4.19
4.16
4.15
4.09
4.08
4.05
4.04
3.54
3.52
3.50
3.49
3.47
3.35
3.34
3.32
3.32
3.30
3.29
2.82
2.39
2.38
2.37
1.06
1.05
1.04
1.03
Me
O
OH
Me
2k ¹H NMR (CDCl₃ 400 MHz)
1.00
1.03
0.99
0.97
0.83
0.78
6.66
10.5 10.0 9.5 9.0 8.5 8.0 7.5 7.0 6.5 6.0 5.5 5.0 4.5 4.0 3.5 3.0 2.5 2.0 1.5 1.0 0.5 0.0 -0.5
f1 (ppm)

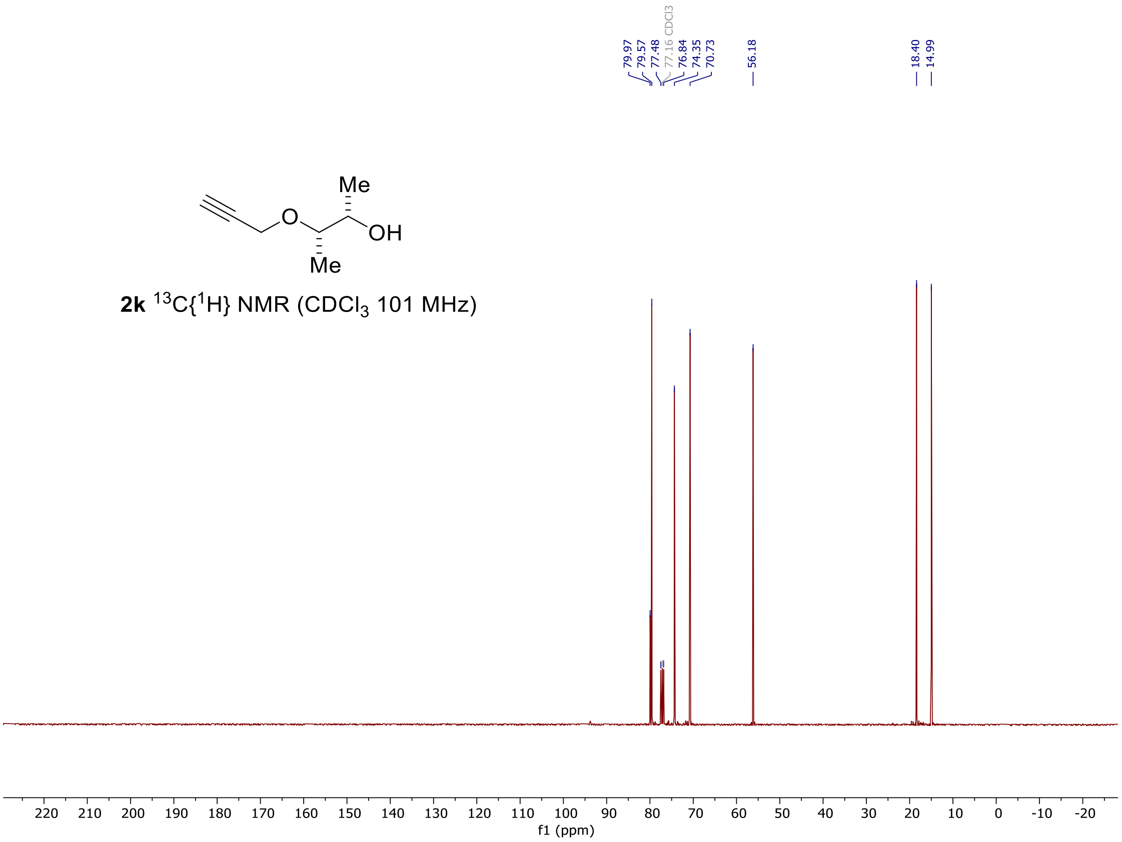
79.97
79.57
77.48
77.16 CDCl3
76.84
74.35
70.73
56.18
18.40
14.99
Me
O
OH
Me
2k ¹³C{¹H} NMR (CDCl₃ 101 MHz)
220 210 200 190 180 170 160 150 140 130 120 110 100 90 80 70 60 50 40 30 20 10 0 -10 -20
f1 (ppm)

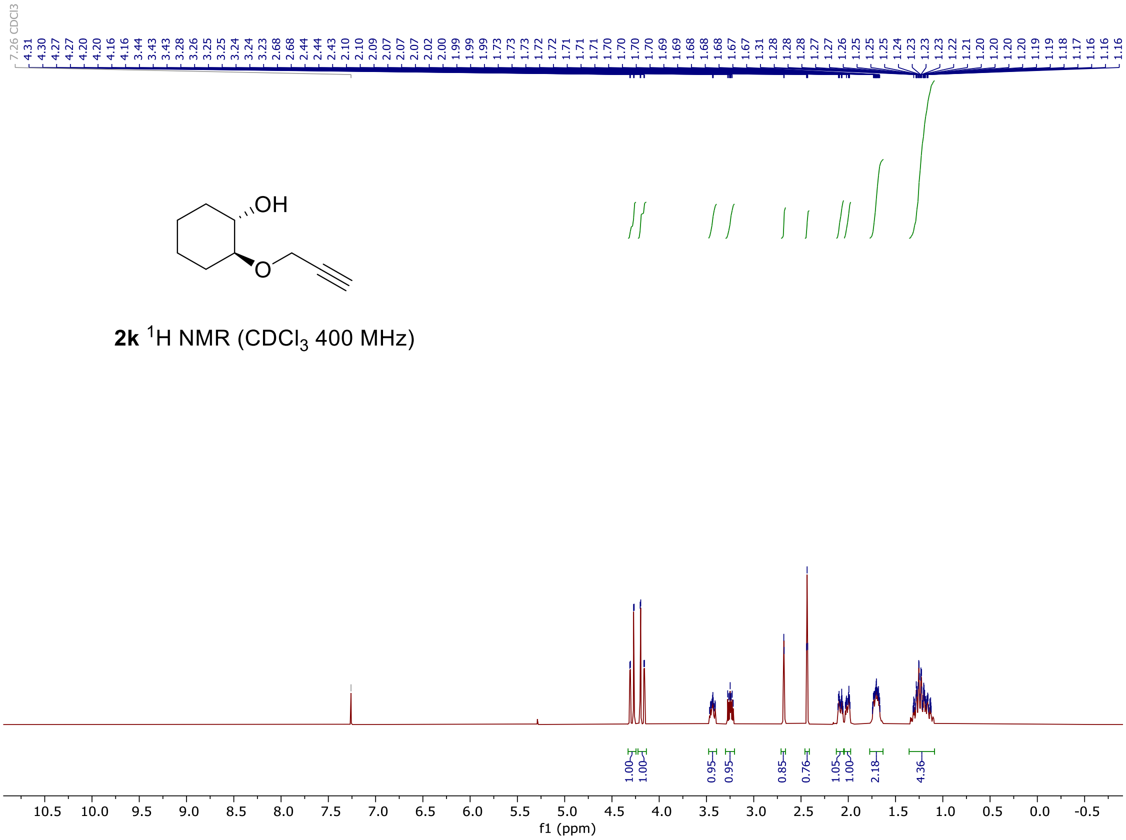

2k ^{1}H NMR ($CDCl_3$ 400 MHz)

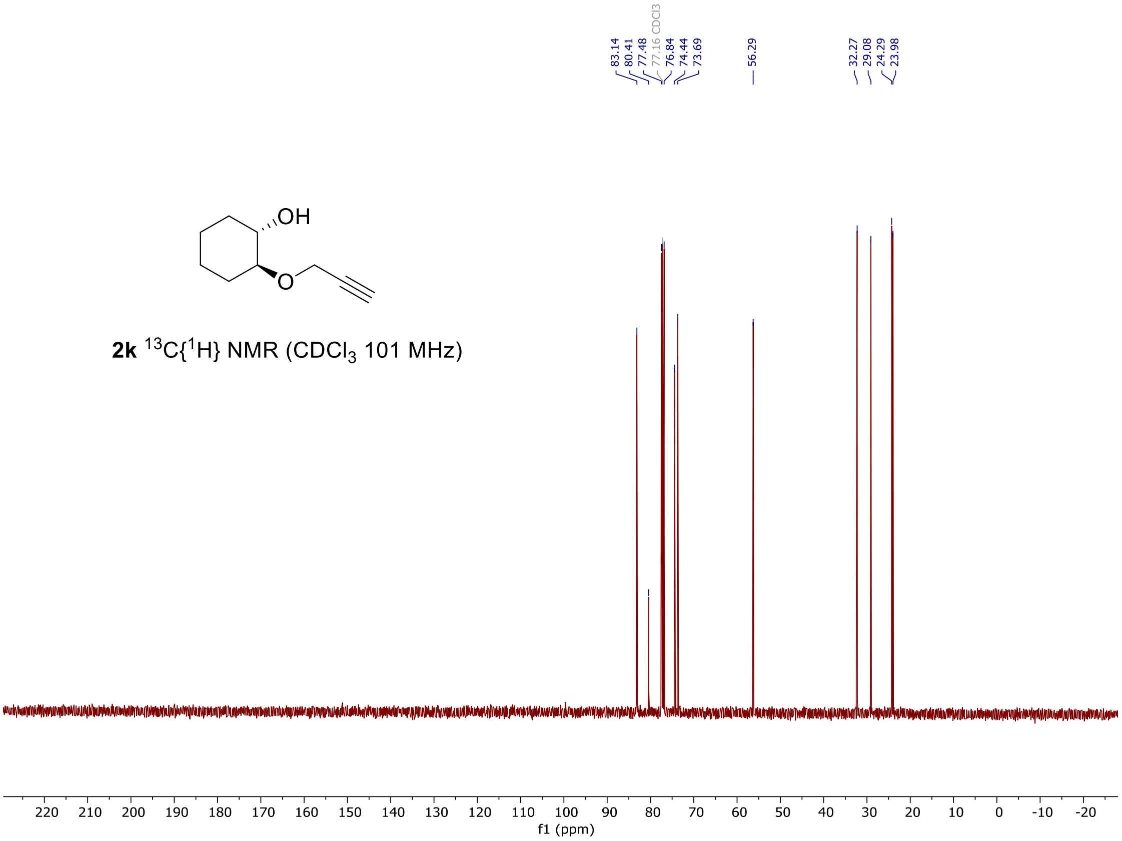

2k $^{13}C\{^{1}H\}$ NMR ($CDCl_3$ 101 MHz)

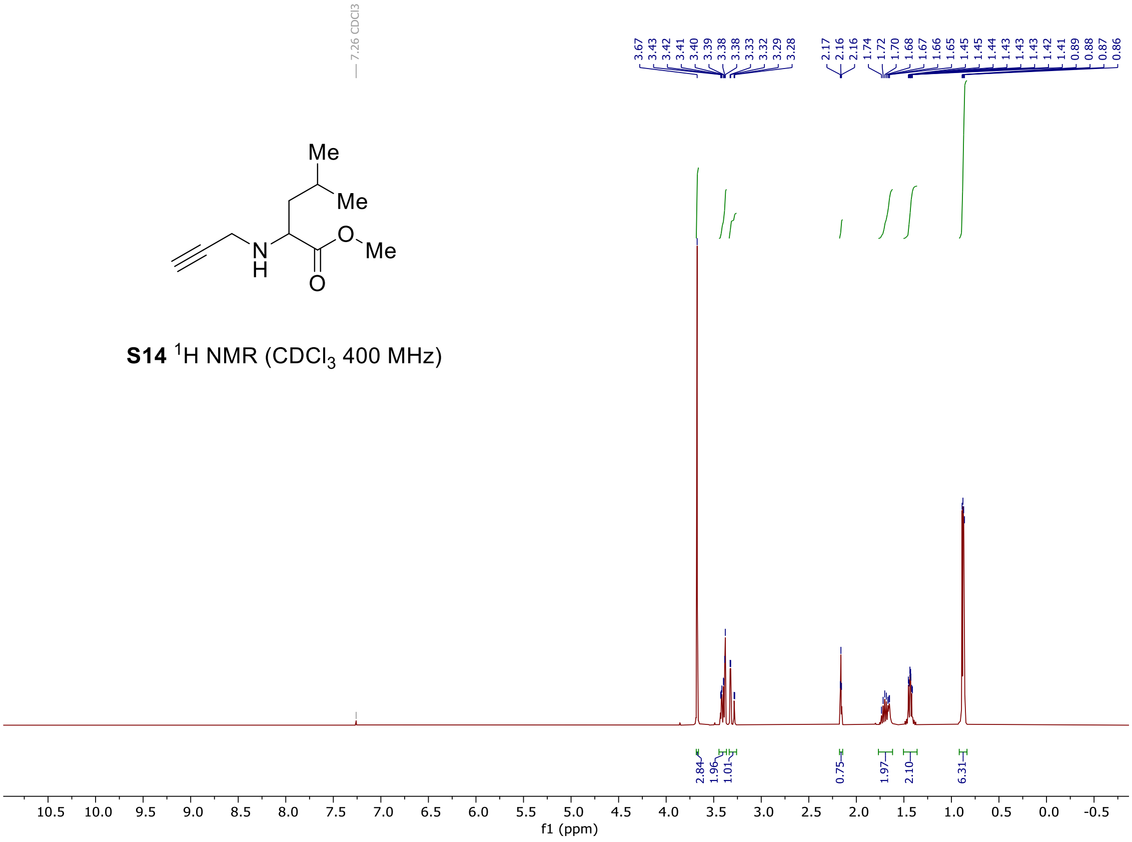

S14 ^{1}H NMR ($CDCl_3$ 400 MHz)

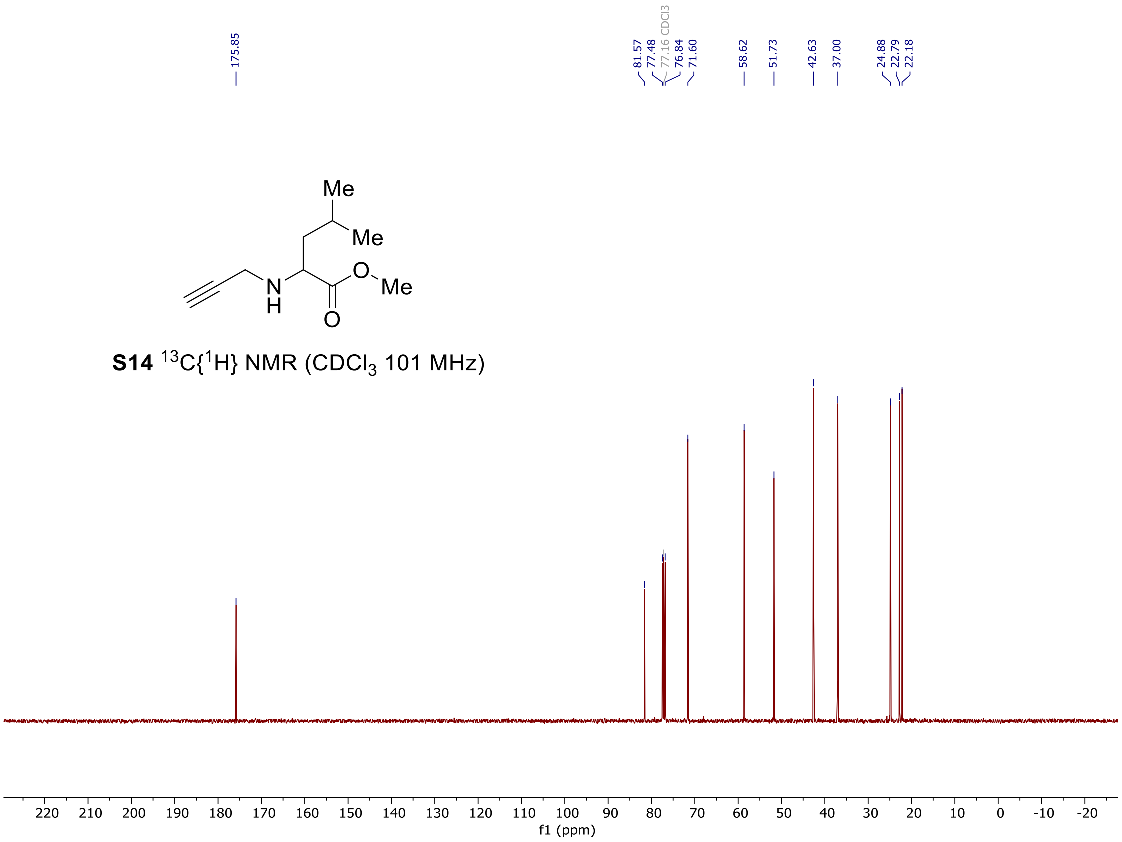

S14 $^{13}C\{^{1}H\}$ NMR ($CDCl_3$ 101 MHz)

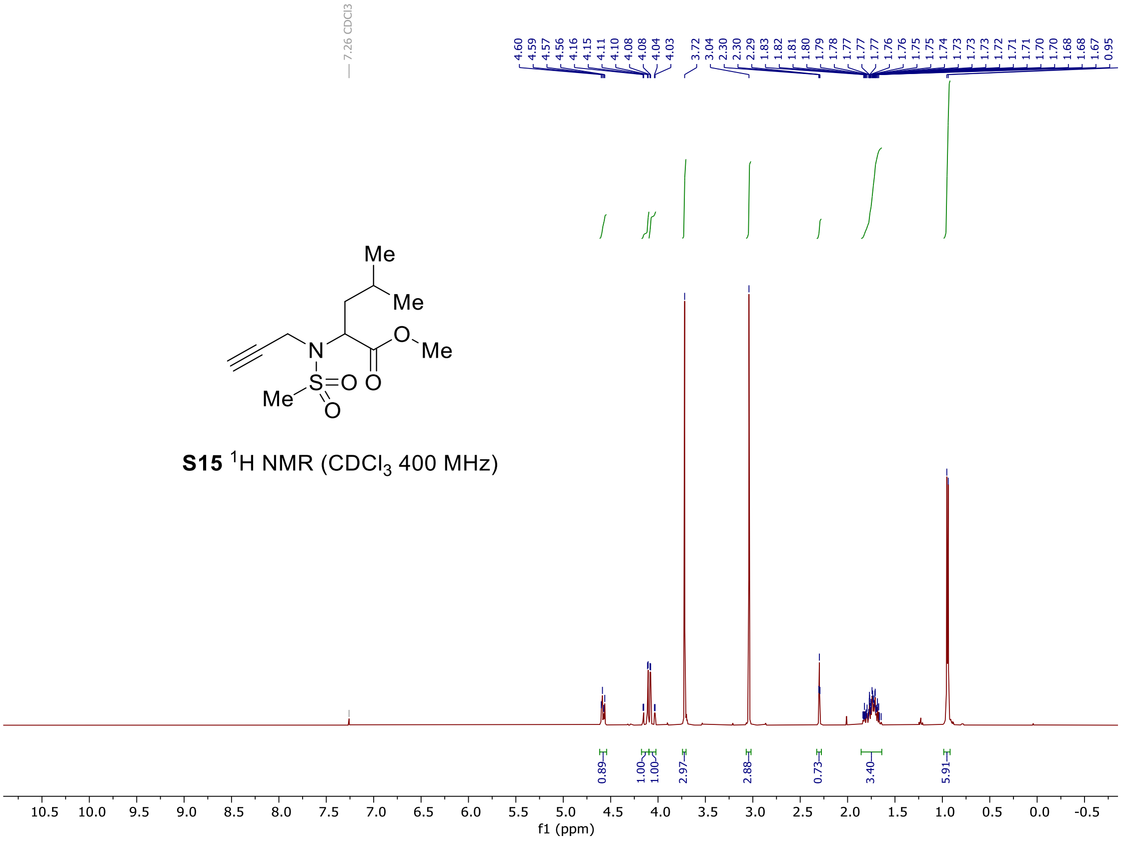

S15 ^{1}H NMR ($CDCl_3$ 400 MHz)

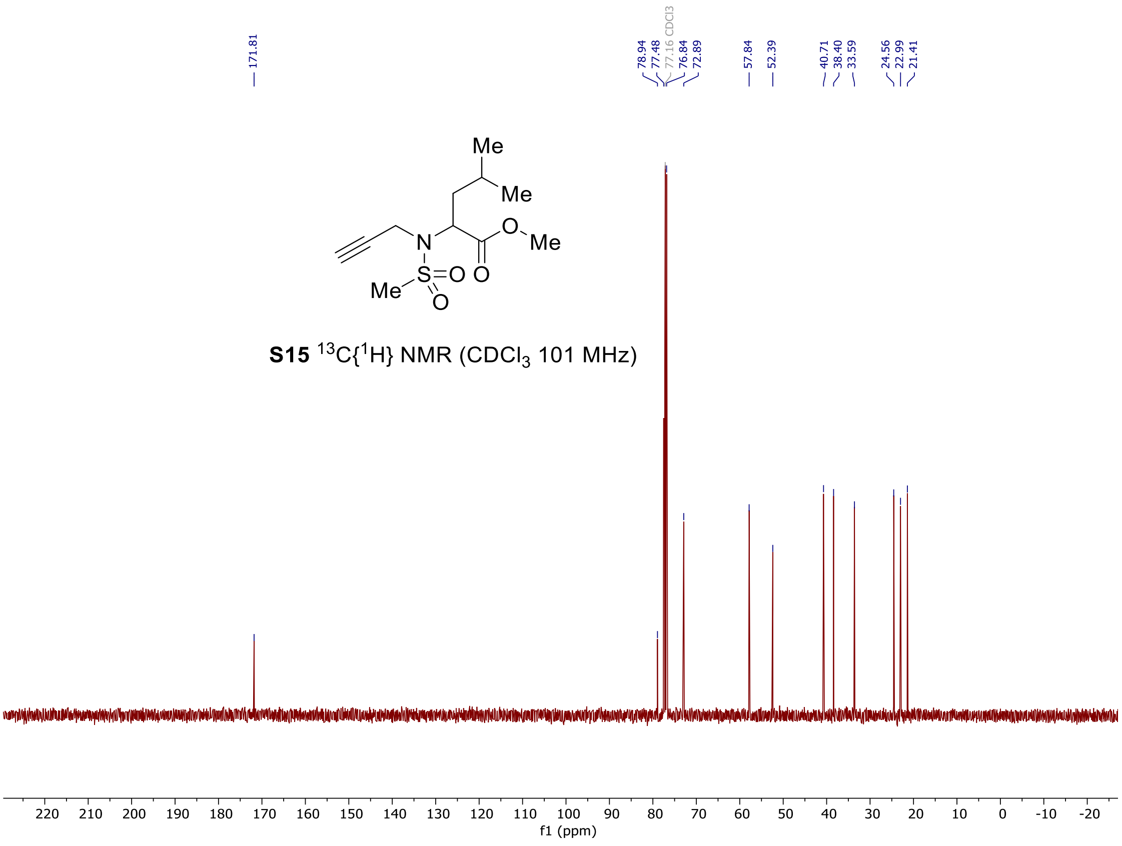

S15 ^{13}C{^{1}H} NMR ($CDCl_3$ 101 MHz)

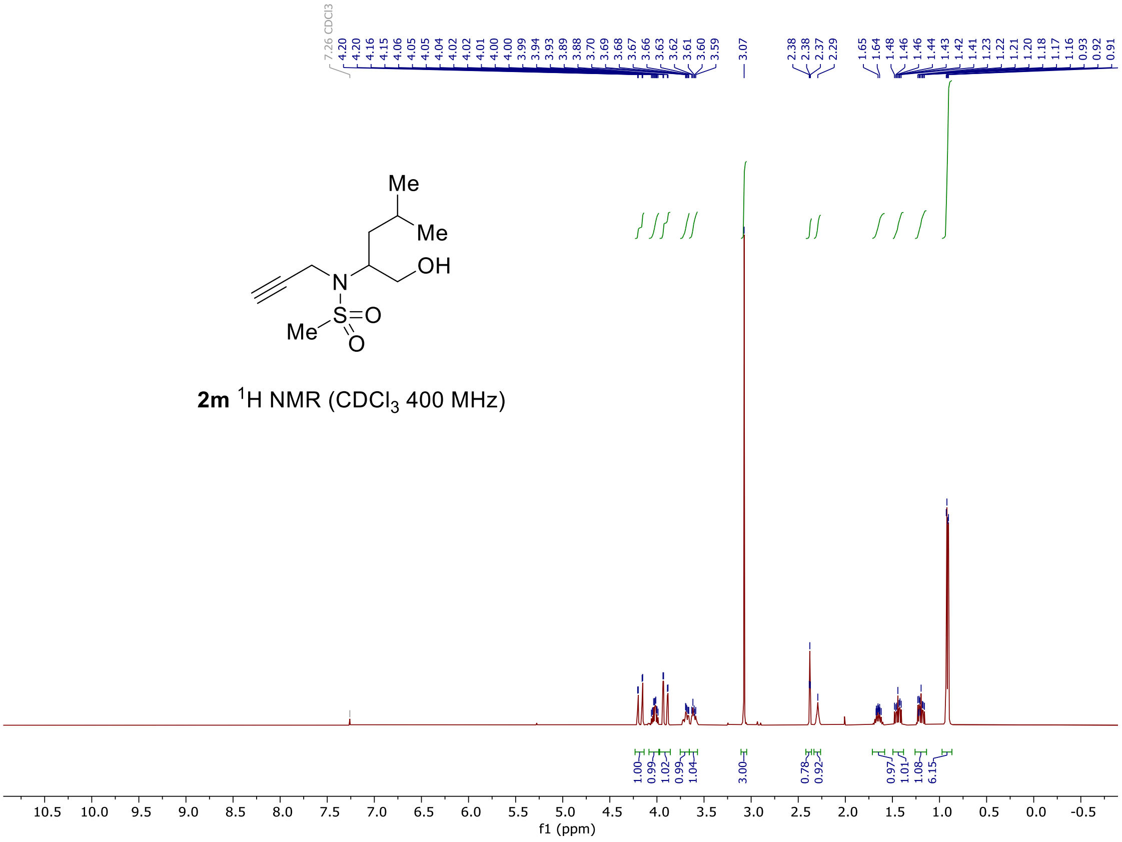

2m ^{1}H NMR ($CDCl_3$ 400 MHz)

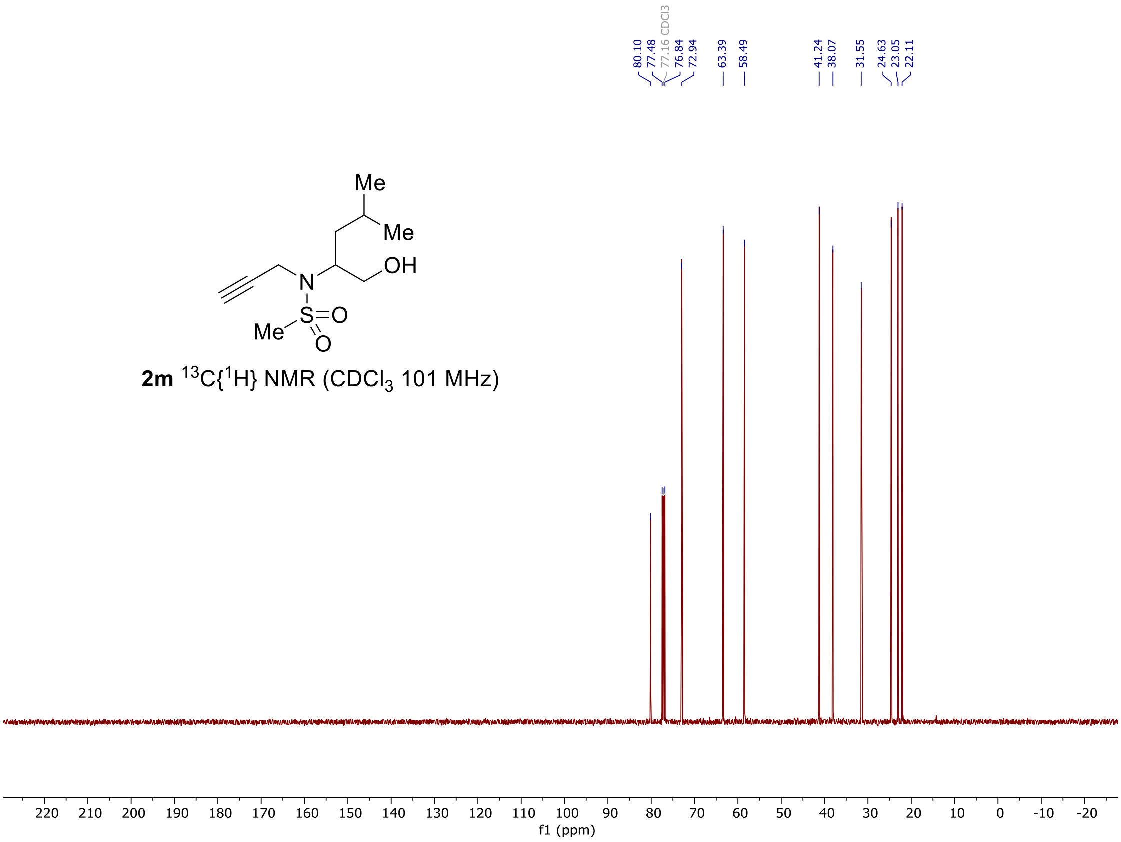

2m $^{13}C\{^{1}H\}$ NMR ($CDCl_3$ 101 MHz)

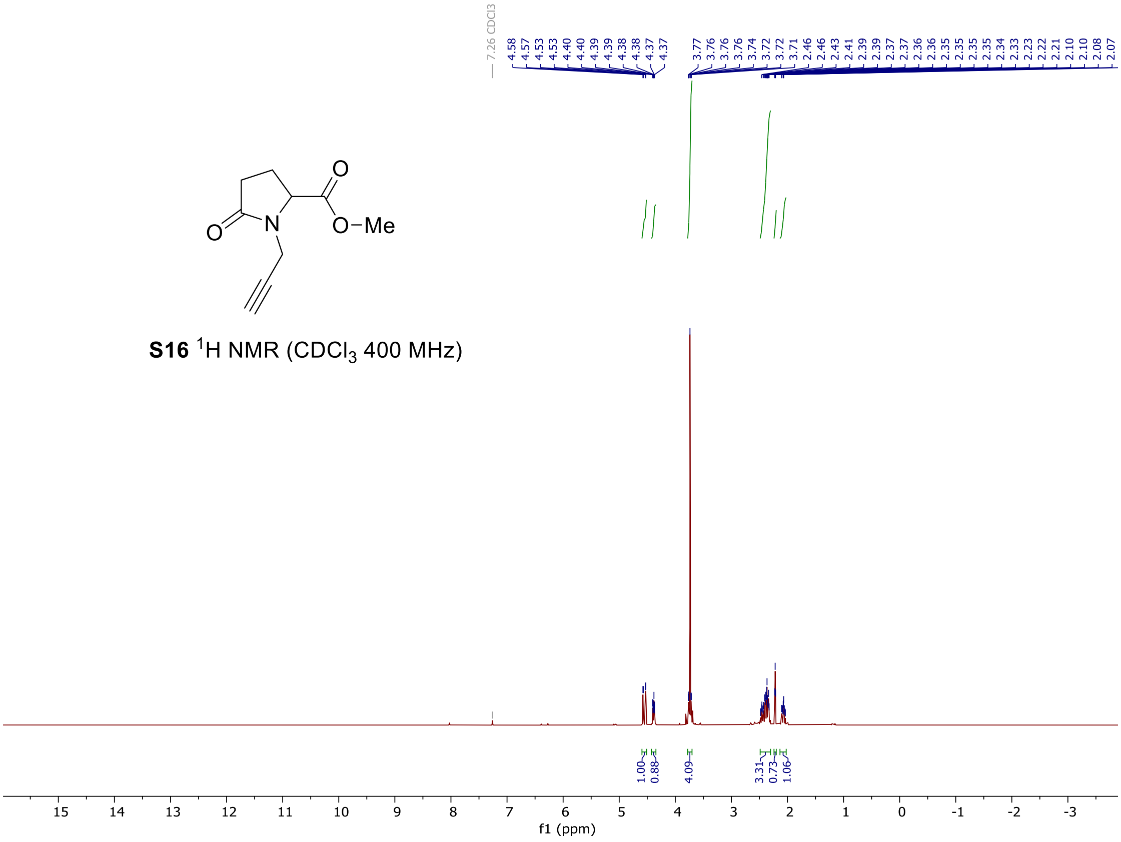

S16 ^{1}H NMR ($CDCl_3$ 400 MHz)

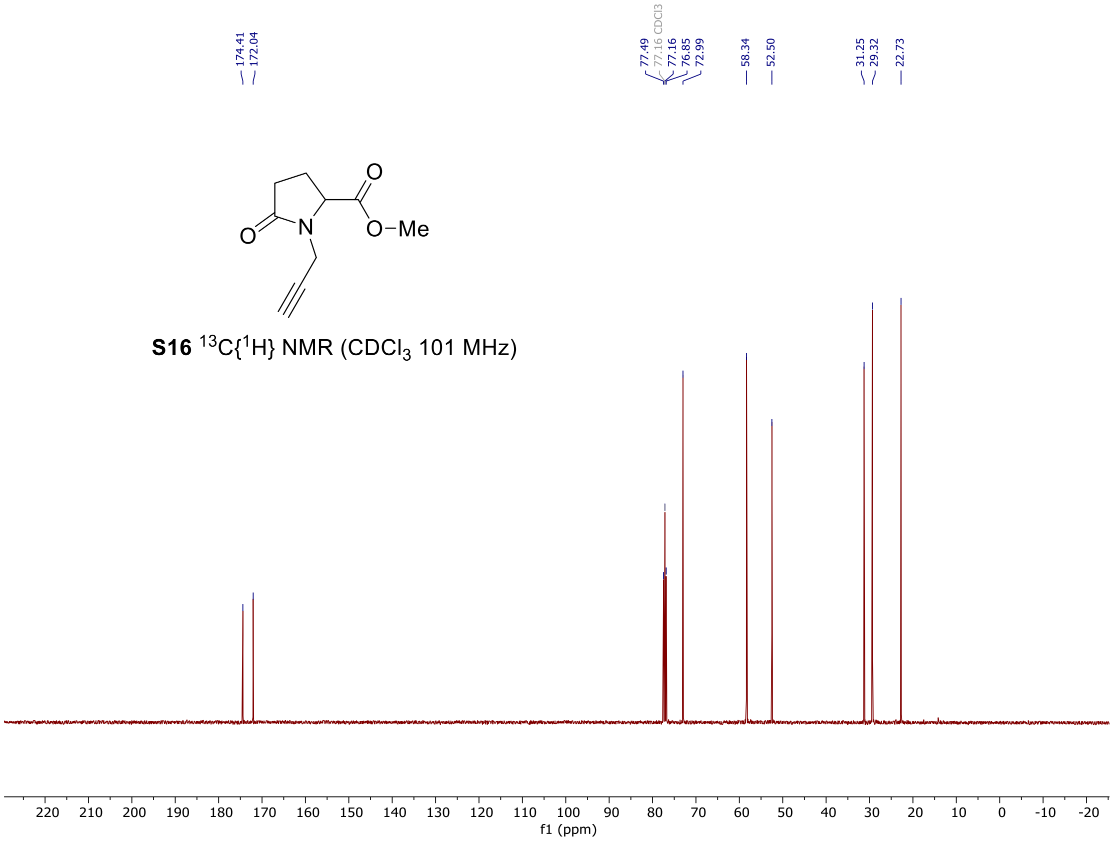

S16 ^{13}C{^{1}H} NMR ($CDCl_3$ 101 MHz)

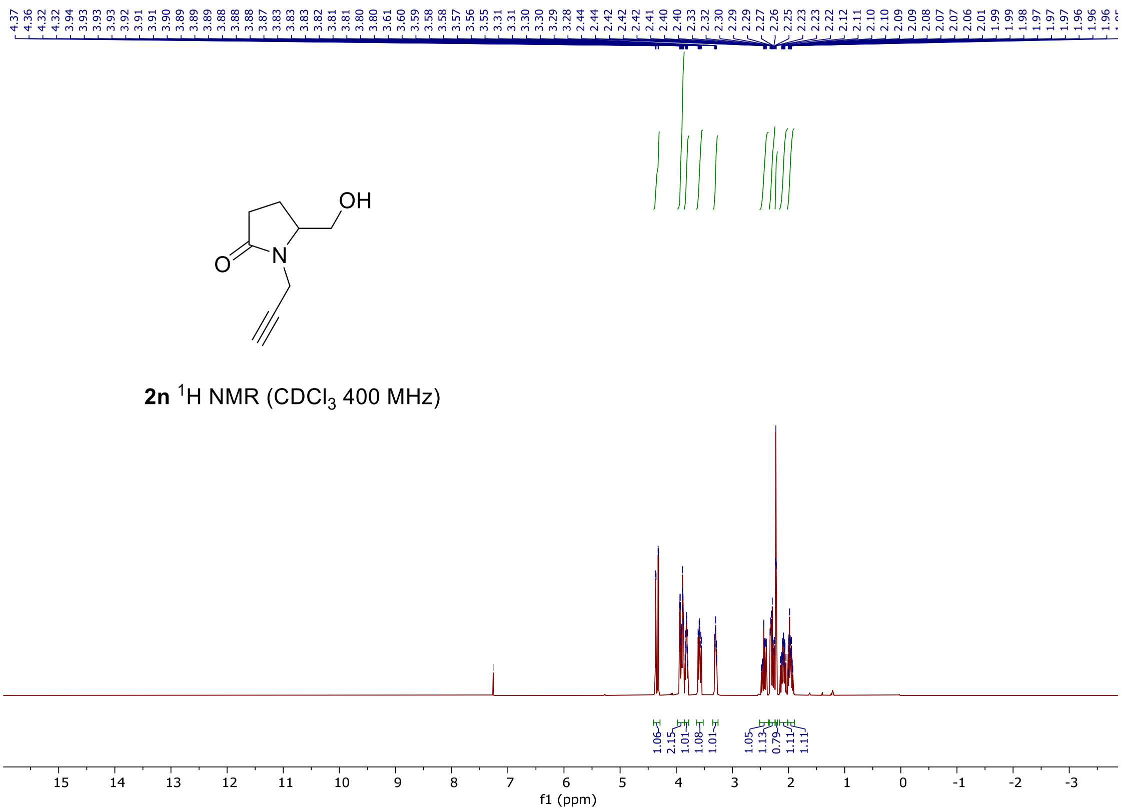

2n ^{1}H NMR ($CDCl_3$ 400 MHz)

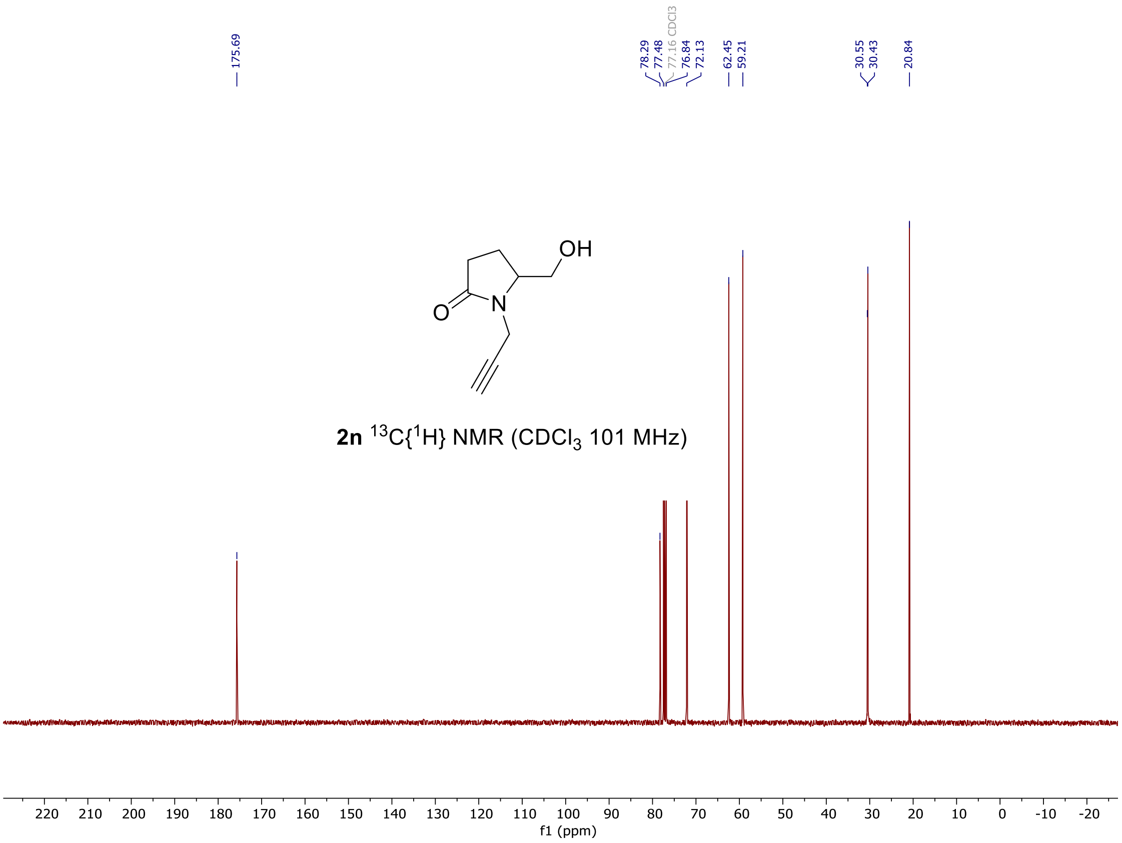

2n $^{13}C\{^{1}H\}$ NMR ($CDCl_3$ 101 MHz)

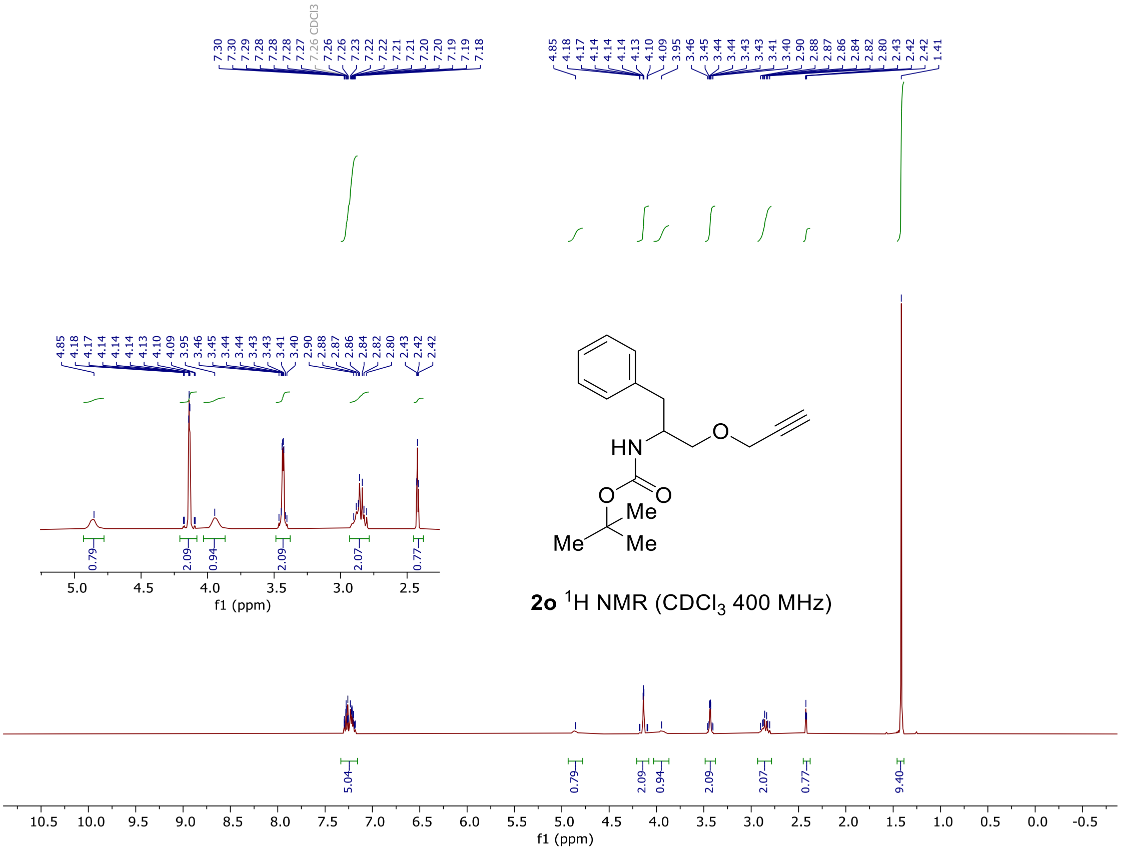
2o ¹H NMR (CDCl₃ 400 MHz)
HN
O
Me
f1 (ppm)

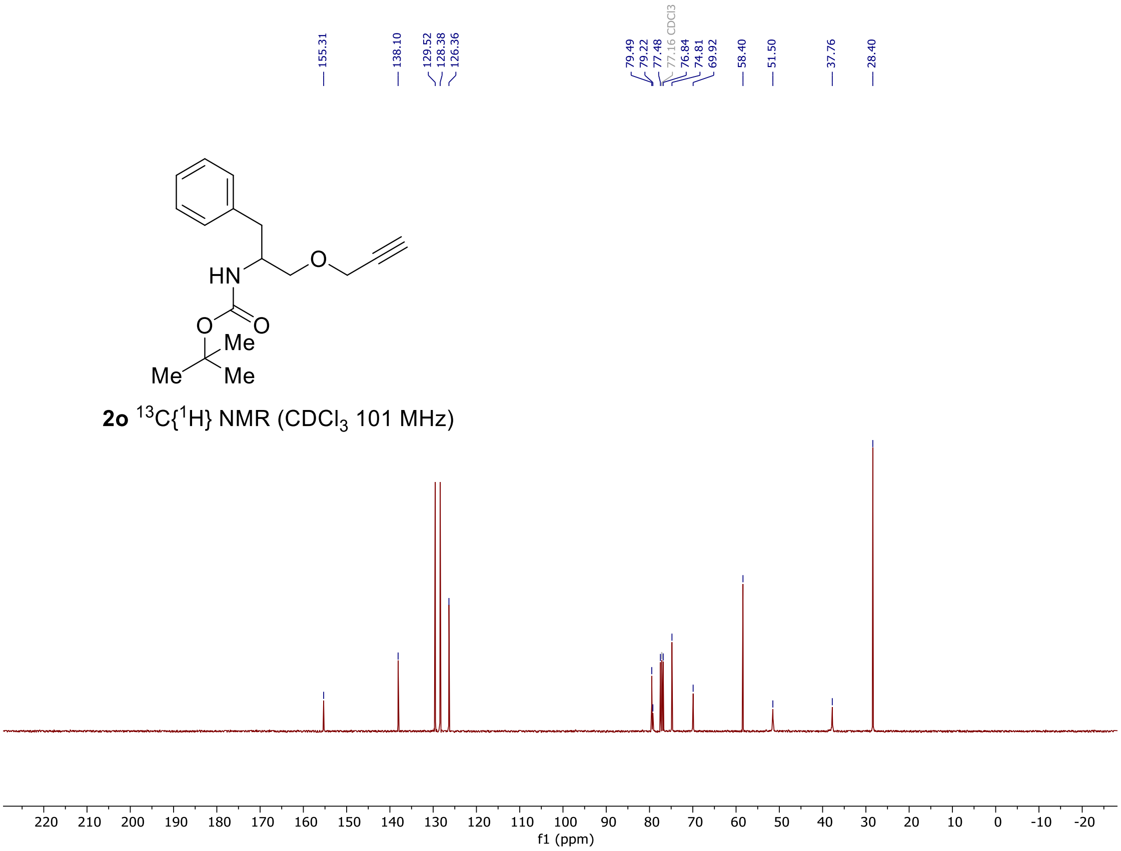
155.31
138.10
129.52
128.38
126.36
79.49
79.22
77.48
77.16 CDCl3
76.84
74.81
69.92
58.40
51.50
37.76
28.40
2o ¹³C{¹H} NMR (CDCl₃ 101 MHz)
f1 (ppm)

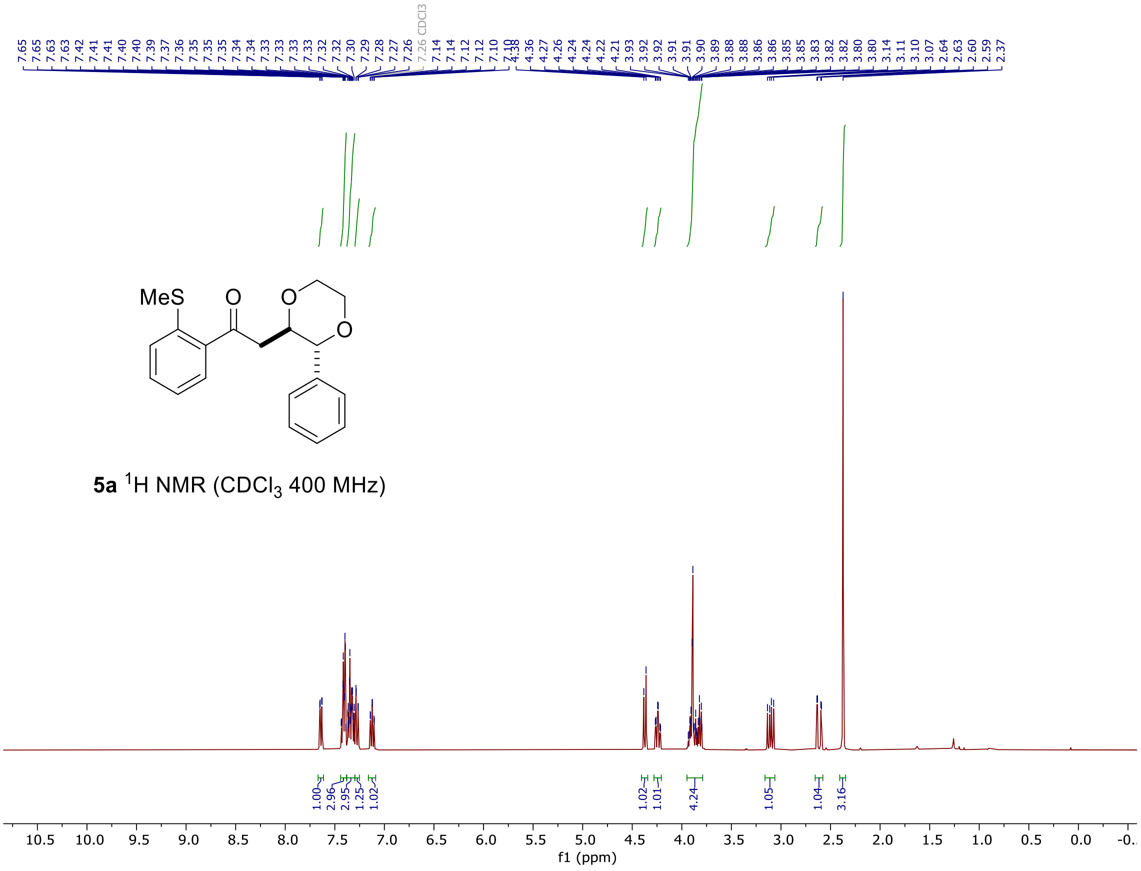

5a ^{1}H NMR ($CDCl_3$ 400 MHz)

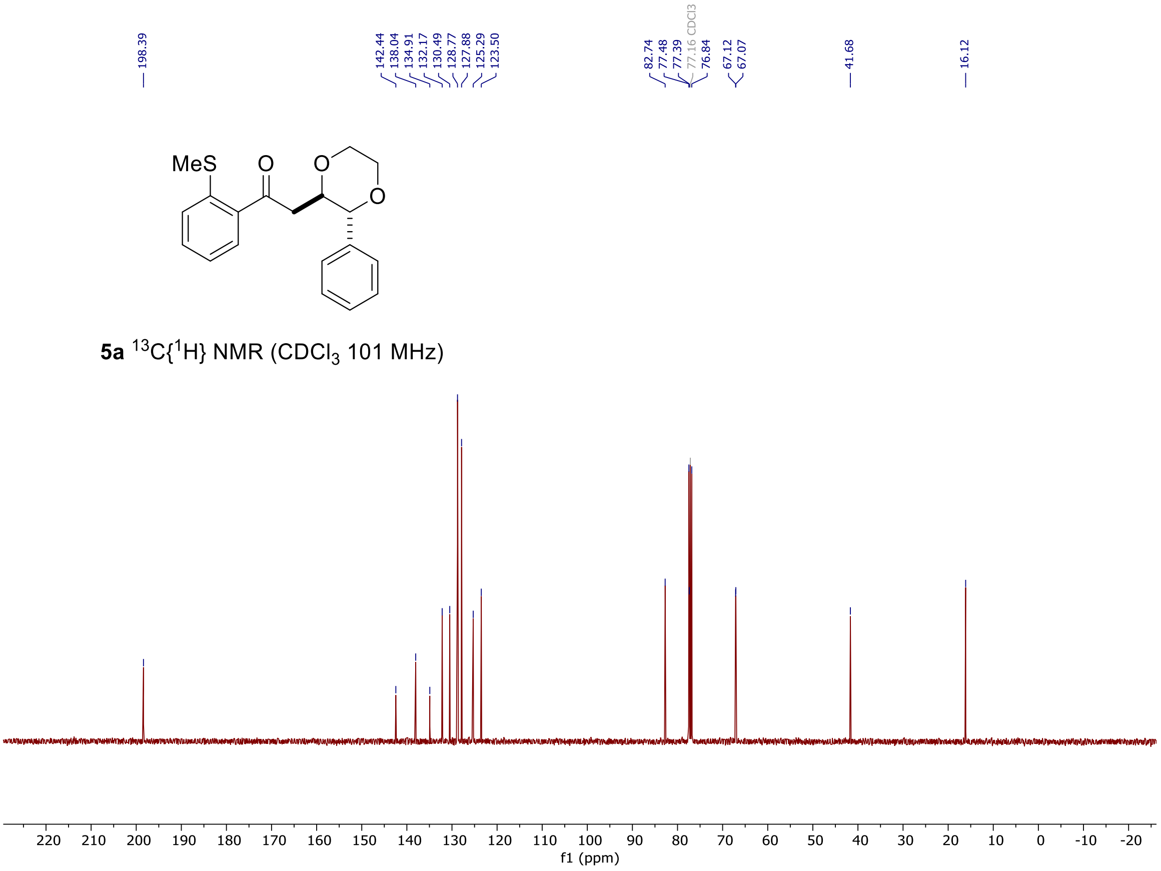

5a $^{13}C\{^{1}H\}$ NMR ($CDCl_3$ 101 MHz)

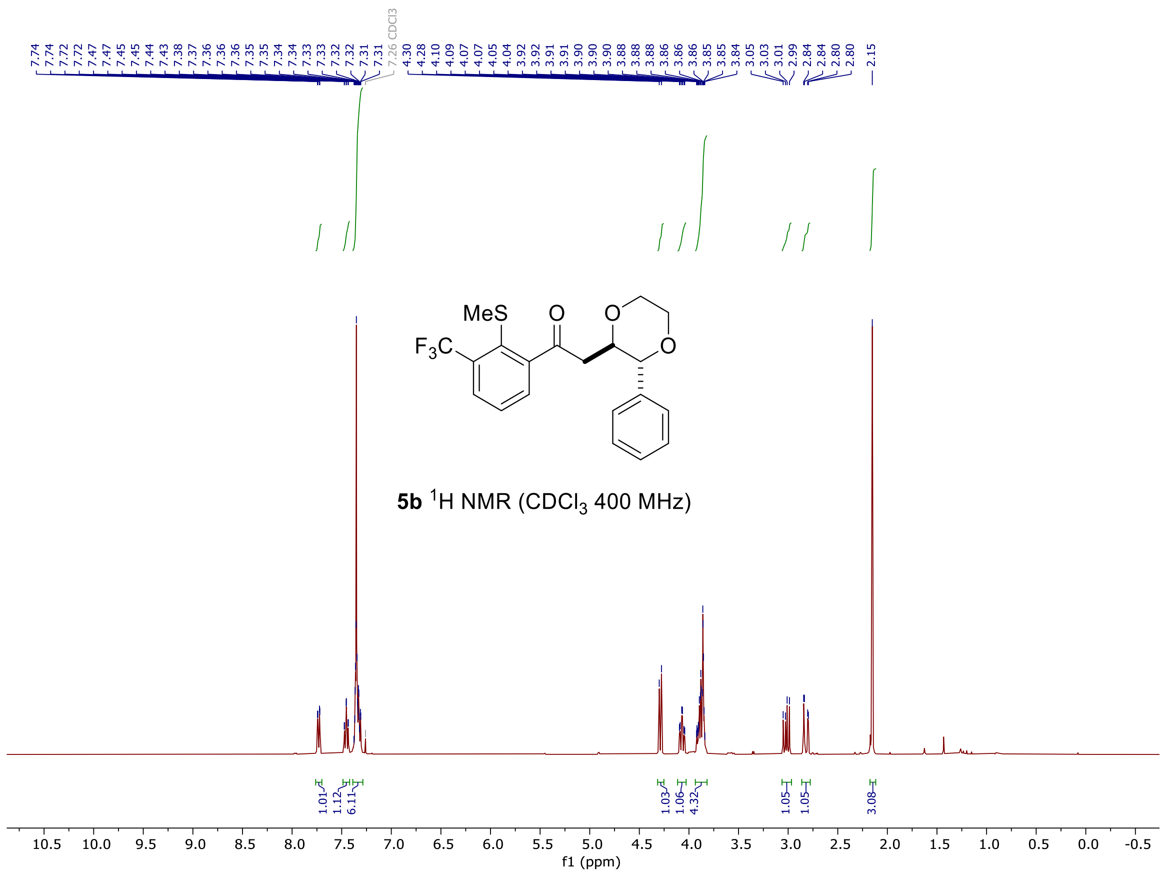
MeS
O
O
O
F3C
5b 1H NMR (CDCl3 400 MHz)
f1 (ppm)

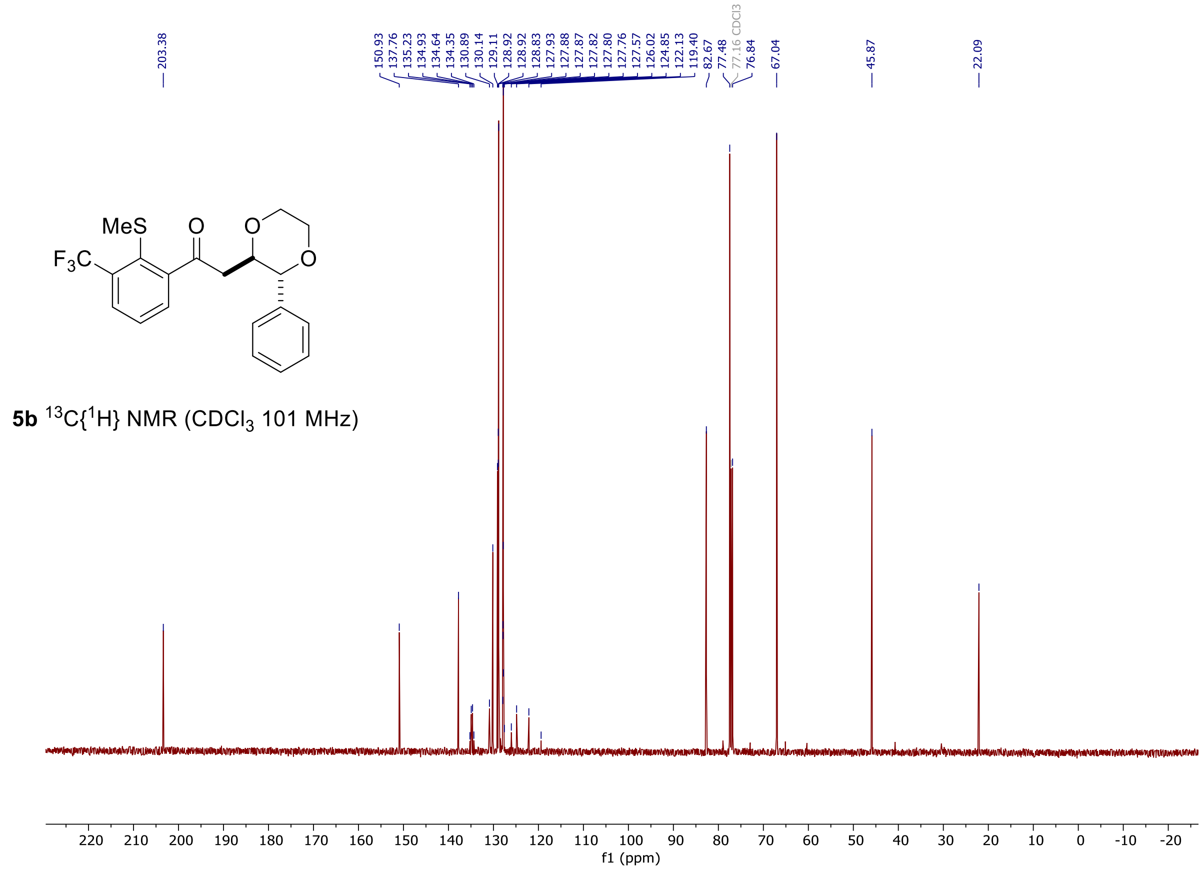
MeS
O
O
O
F3C
5b 13C{1H} NMR (CDCl3 101 MHz)
f1 (ppm)

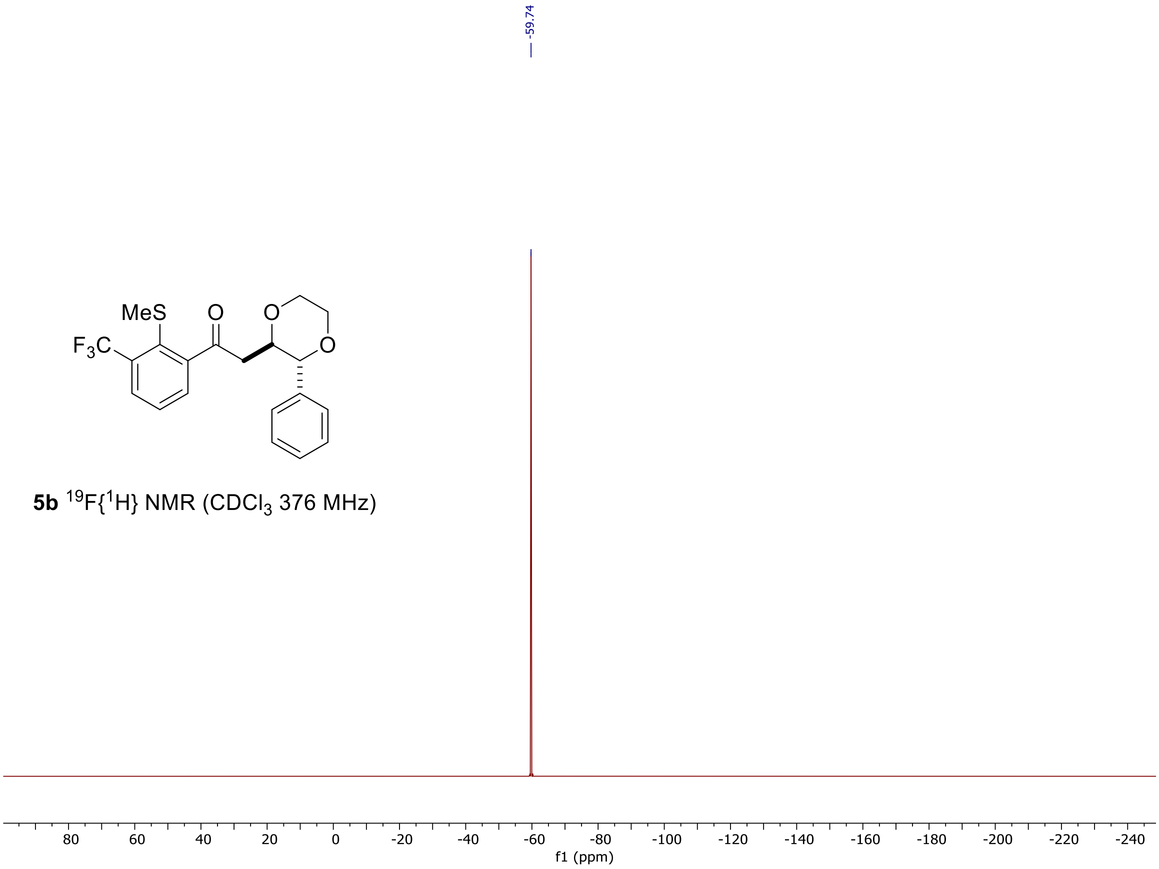

5b $^{19}F\{^1H\}$ NMR ($CDCl_3$ 376 MHz)

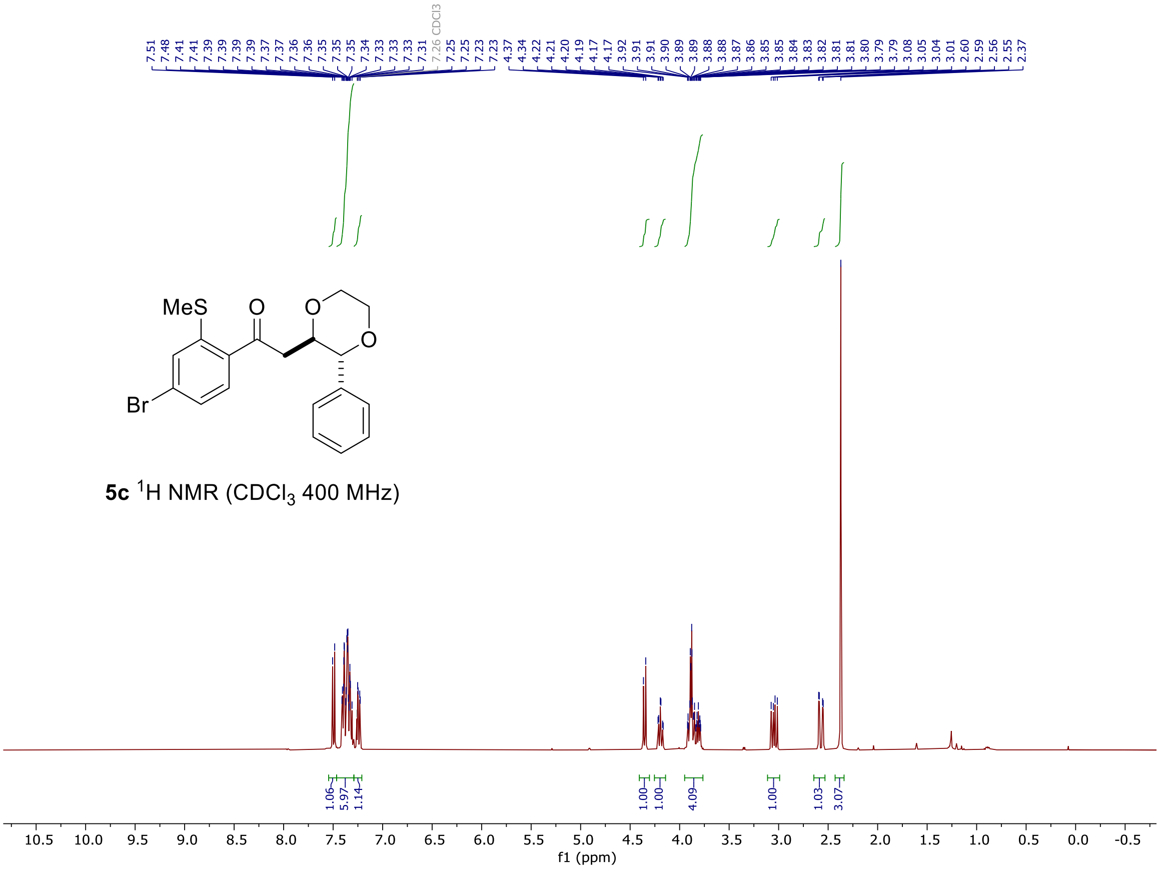
7.51 7.48 7.41 7.41 7.39 7.39 7.39 7.39 7.37 7.37 7.36 7.36 7.35 7.35 7.35 7.34 7.33 7.33 7.33 7.31 7.26 CDCl3 7.25 7.25 7.23 7.23 4.37 4.34 4.22 4.21 4.20 4.19 4.17 4.17 3.92 3.91 3.91 3.90 3.89 3.89 3.88 3.88 3.87 3.86 3.85 3.85 3.84 3.83 3.82 3.81 3.81 3.80 3.79 3.79 3.08 3.05 3.04 3.01 2.60 2.59 2.56 2.55 2.37
MeS
O
O
O
Br
5c ¹H NMR (CDCl₃ 400 MHz)
1.06 5.97 1.14 1.00 1.00 4.09 1.00 1.03 3.07
10.5 10.0 9.5 9.0 8.5 8.0 7.5 7.0 6.5 6.0 5.5 5.0 4.5 4.0 3.5 3.0 2.5 2.0 1.5 1.0 0.5 0.0 -0.5
f1 (ppm)

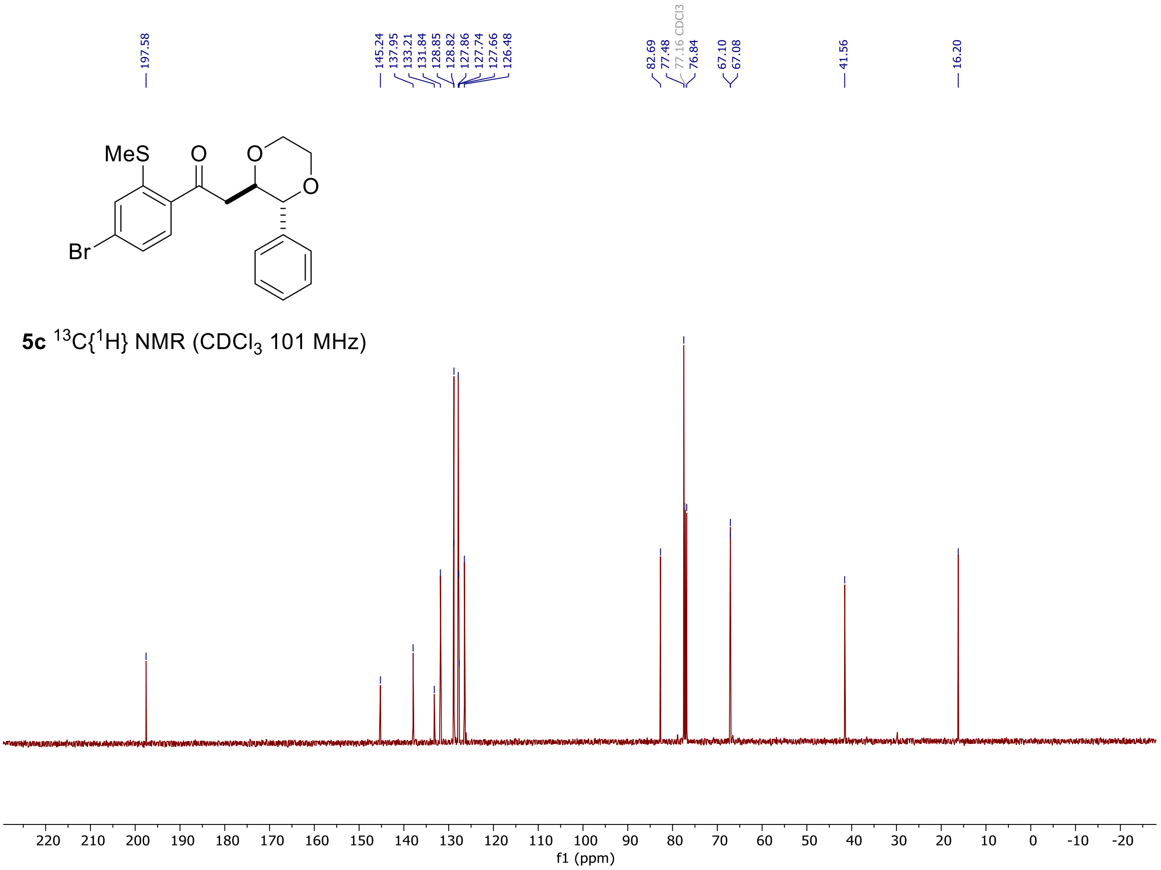
197.58
145.24 137.95 133.21 131.84 128.85 128.82 127.86 127.74 127.66 126.48
82.69 77.48 77.16 CDCl3 76.84 67.10 67.08
41.56
16.20
MeS
O
O
O
Br
5c ¹³C{¹H} NMR (CDCl₃ 101 MHz)
220 210 200 190 180 170 160 150 140 130 120 110 100 90 80 70 60 50 40 30 20 10 0 -10 -20
f1 (ppm)

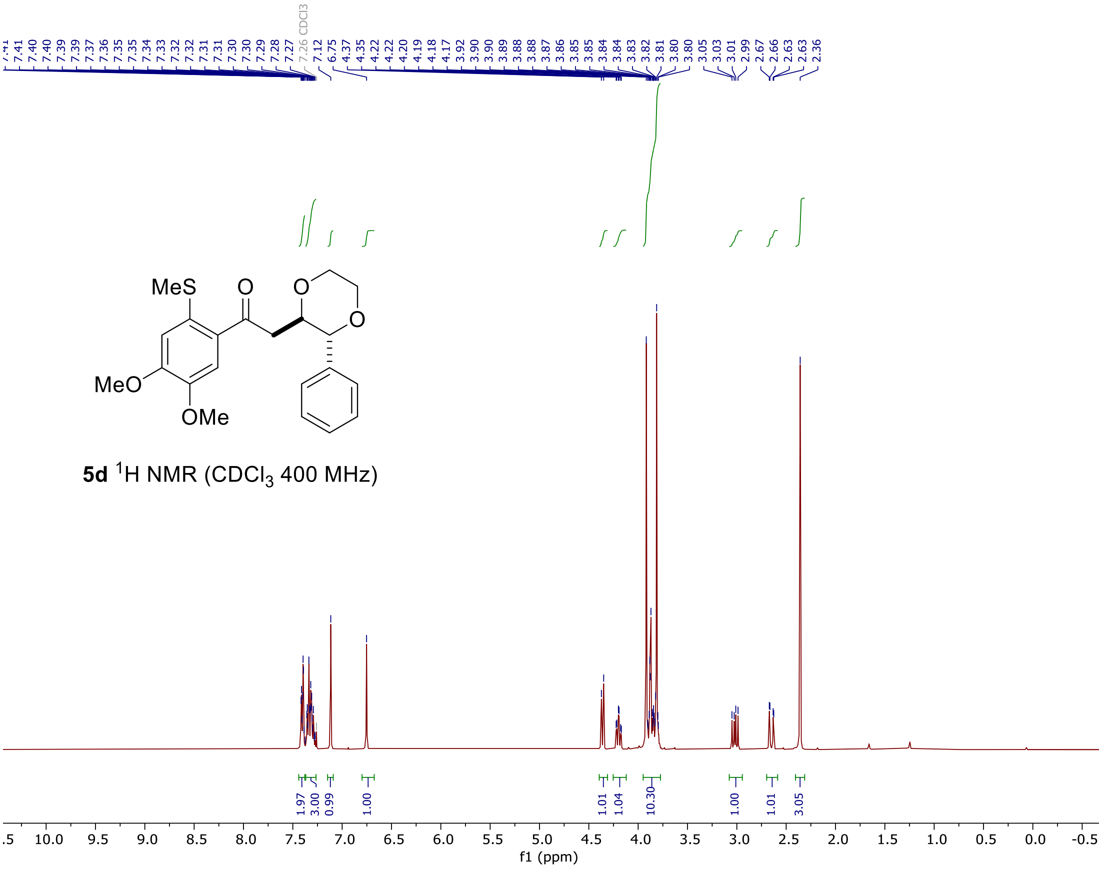

5d ^{1}H NMR ($CDCl_3$ 400 MHz)

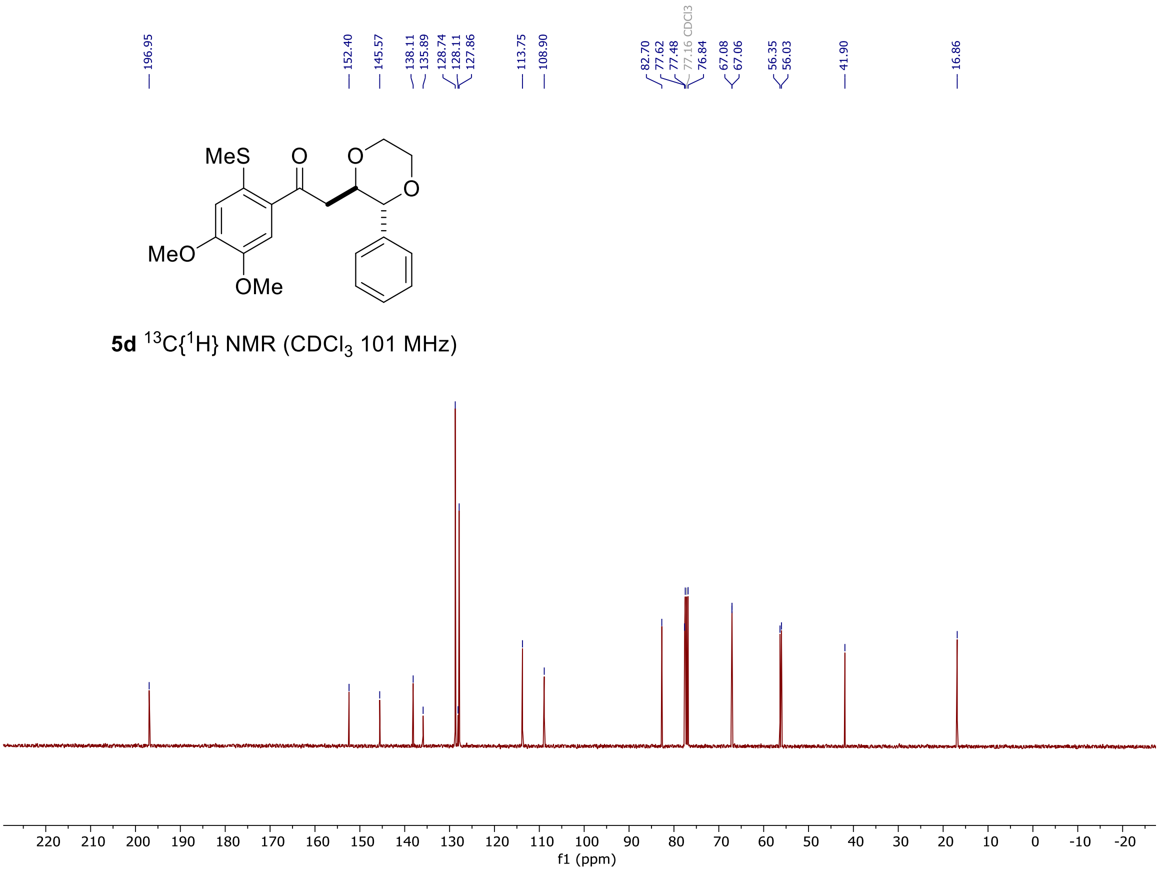

5d ^{13}C{^{1}H} NMR ($CDCl_3$ 101 MHz)

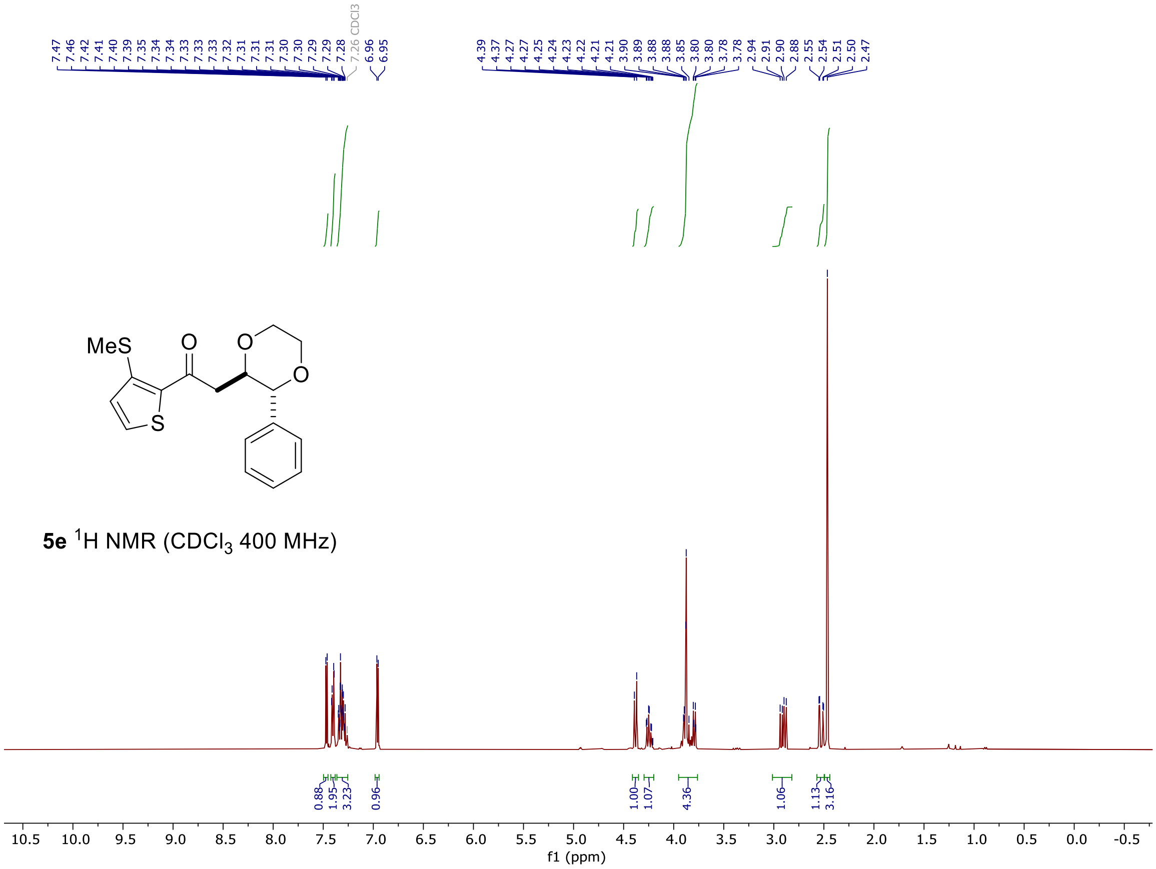

5e ^{1}H NMR ($CDCl_3$ 400 MHz)

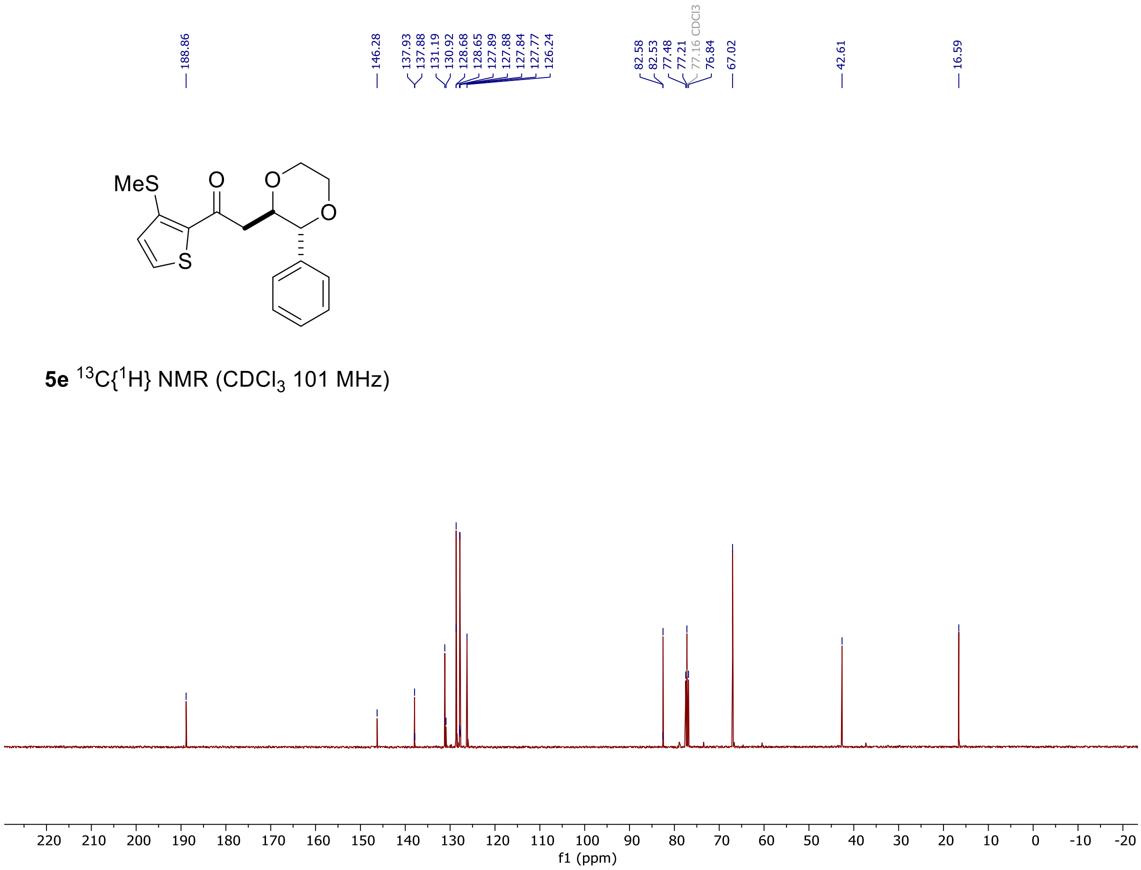

5e ^{13}C{^{1}H} NMR ($CDCl_3$ 101 MHz)

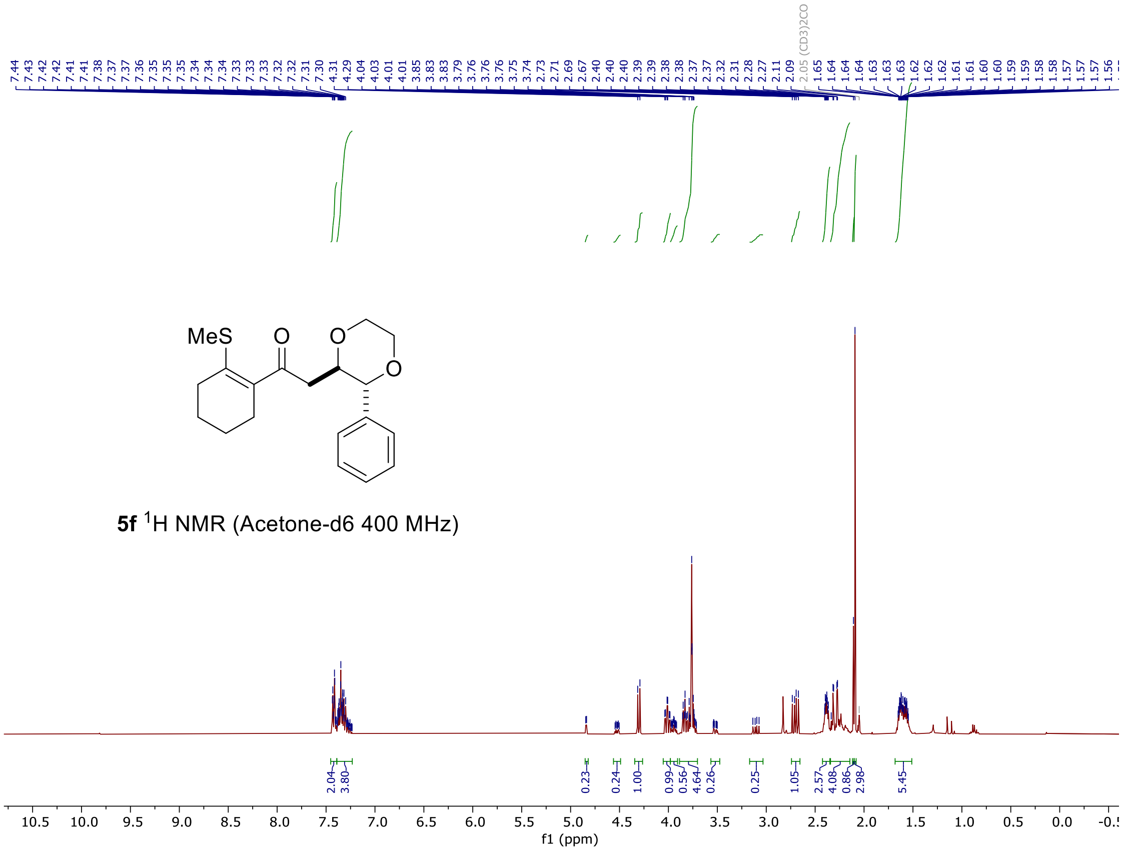

5f ^{1}H NMR (Acetone-d6 400 MHz)

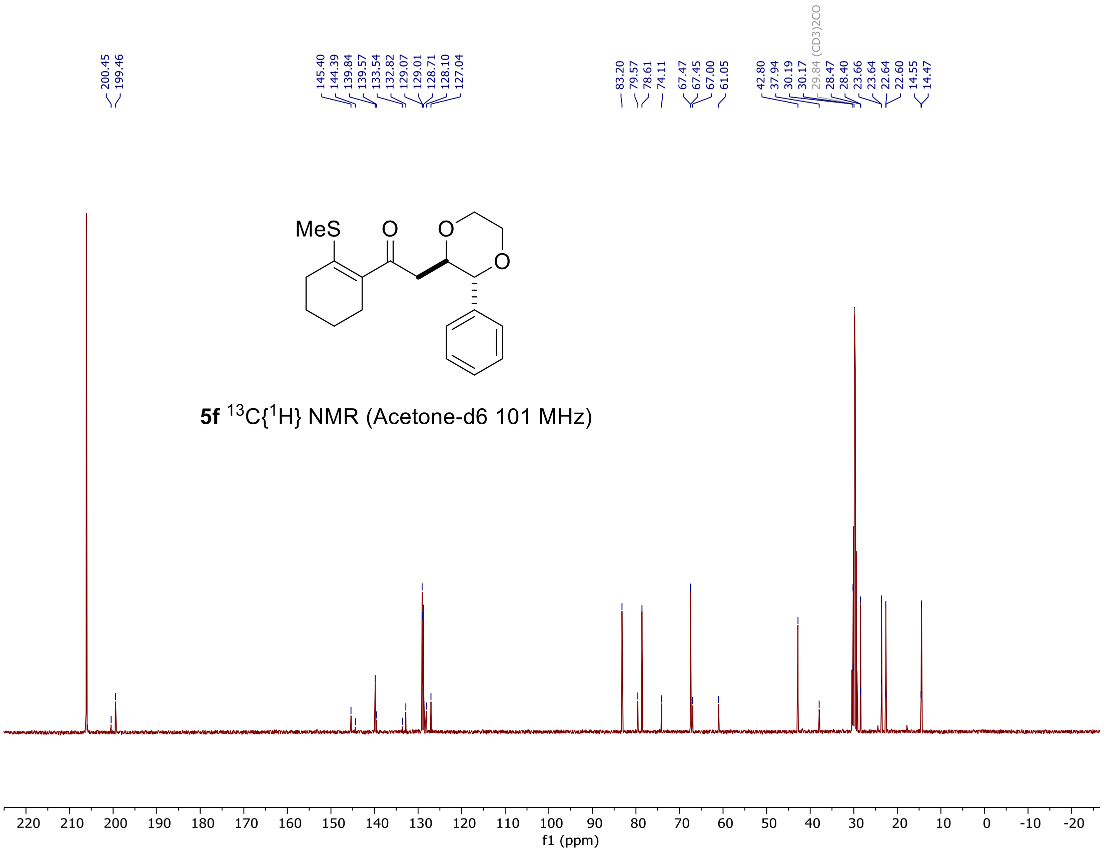

5f ^{13}C{^{1}H} NMR (Acetone-d6 101 MHz)

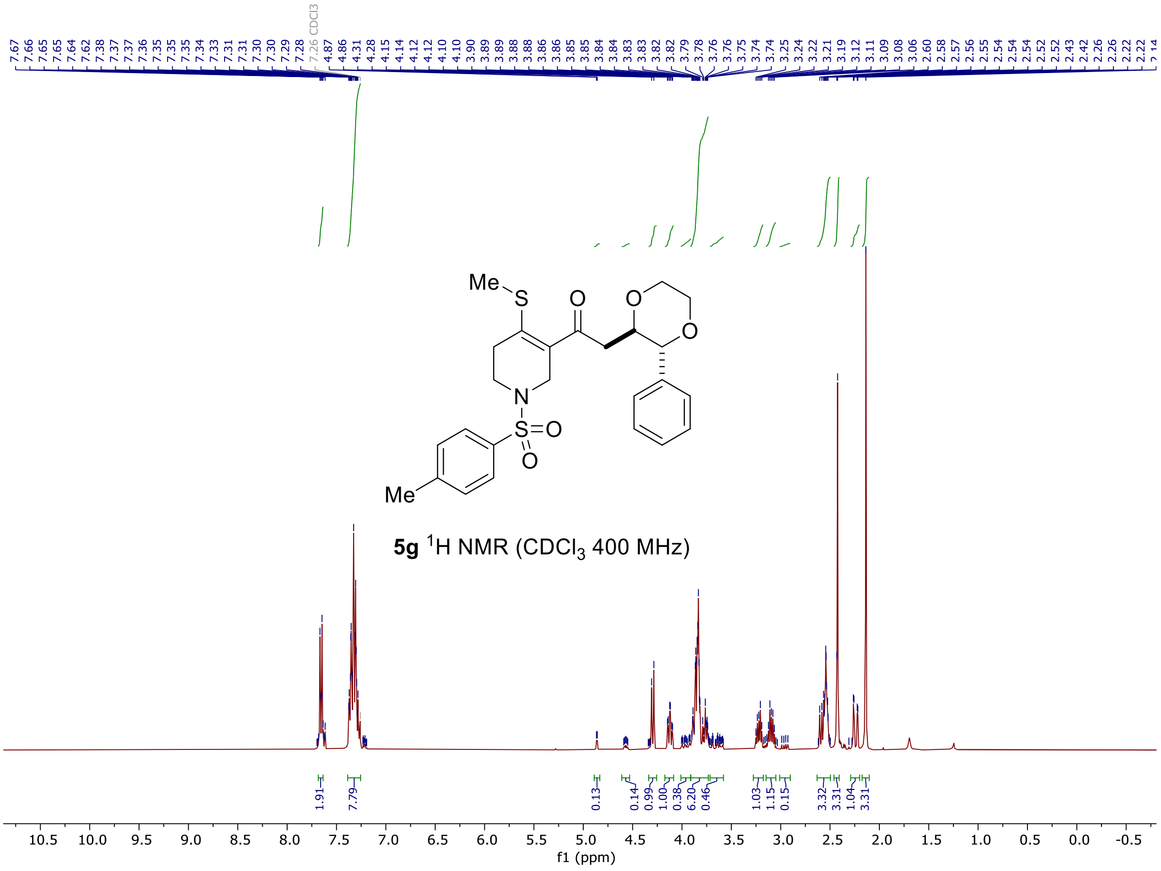
7.67 7.66 7.65 7.65 7.64 7.62 7.38 7.37 7.37 7.36 7.35 7.35 7.35 7.34 7.33 7.31 7.31 7.30 7.30 7.29 7.28 7.26 CDCl3 4.87 4.86 4.31 4.28 4.15 4.14 4.12 4.12 4.10 4.10 3.90 3.89 3.89 3.88 3.88 3.86 3.86 3.85 3.85 3.84 3.84 3.83 3.83 3.82 3.82 3.79 3.78 3.76 3.76 3.75 3.74 3.74 3.74 3.25 3.24 3.22 3.22 3.21 3.19 3.12 3.11 3.09 3.08 3.06 2.60 2.58 2.57 2.56 2.55 2.54 2.54 2.54 2.52 2.52 2.43 2.42 2.26 2.26 2.22 2.22 [illegible]
Me S O O O N S O O Me
5g ¹H NMR (CDCl₃ 400 MHz)
1.91 7.79 0.13 0.14 0.99 1.00 0.38 6.20 0.46 1.03 1.15 0.15 3.32 3.31 1.04 3.31
f1 (ppm)

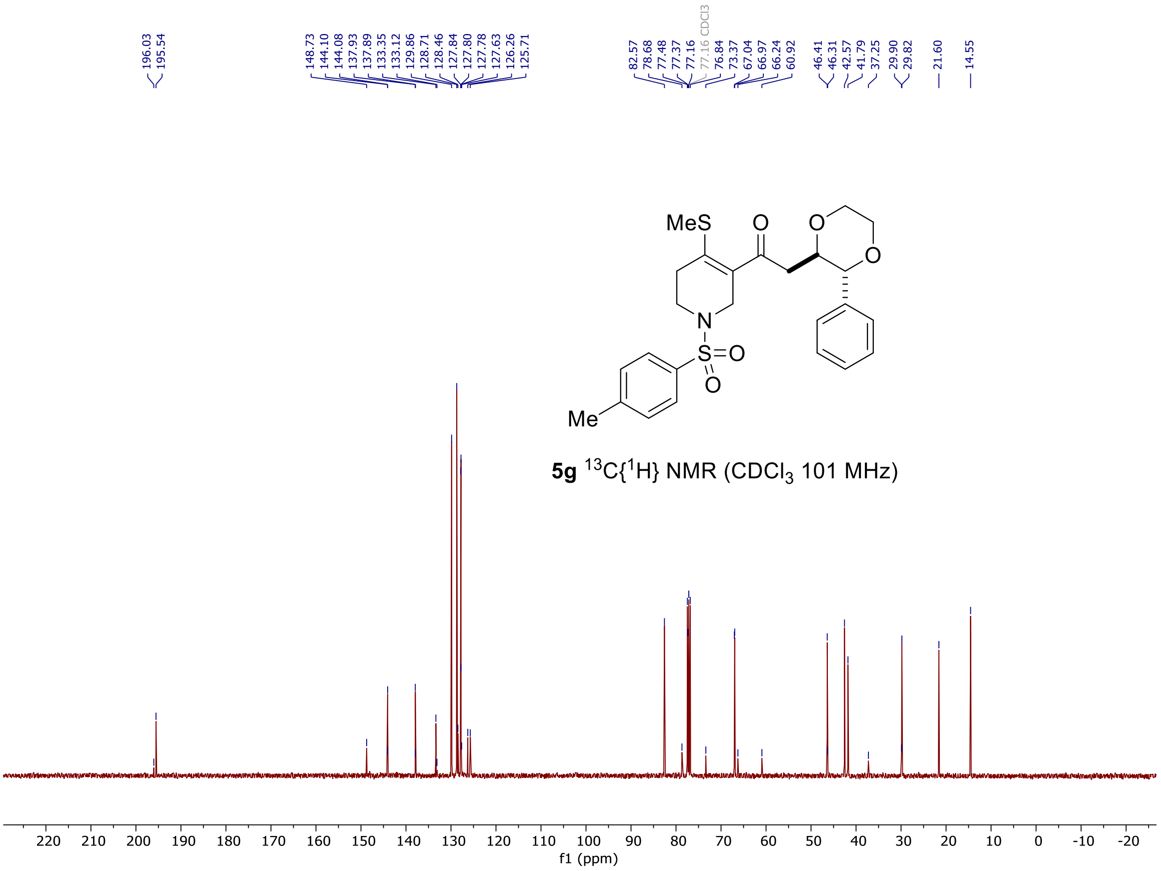
196.03 195.54 148.73 144.10 144.08 137.93 137.89 133.35 133.12 129.86 128.71 128.46 127.84 127.80 127.78 127.63 126.26 125.71 82.57 78.68 77.48 77.37 77.16 CDCl3 76.84 73.37 67.04 66.97 66.24 60.92 46.41 46.31 42.57 41.79 37.25 29.90 29.82 21.60 14.55
MeS O O O N S O O Me
5g ¹³C{¹H} NMR (CDCl₃ 101 MHz)
f1 (ppm)

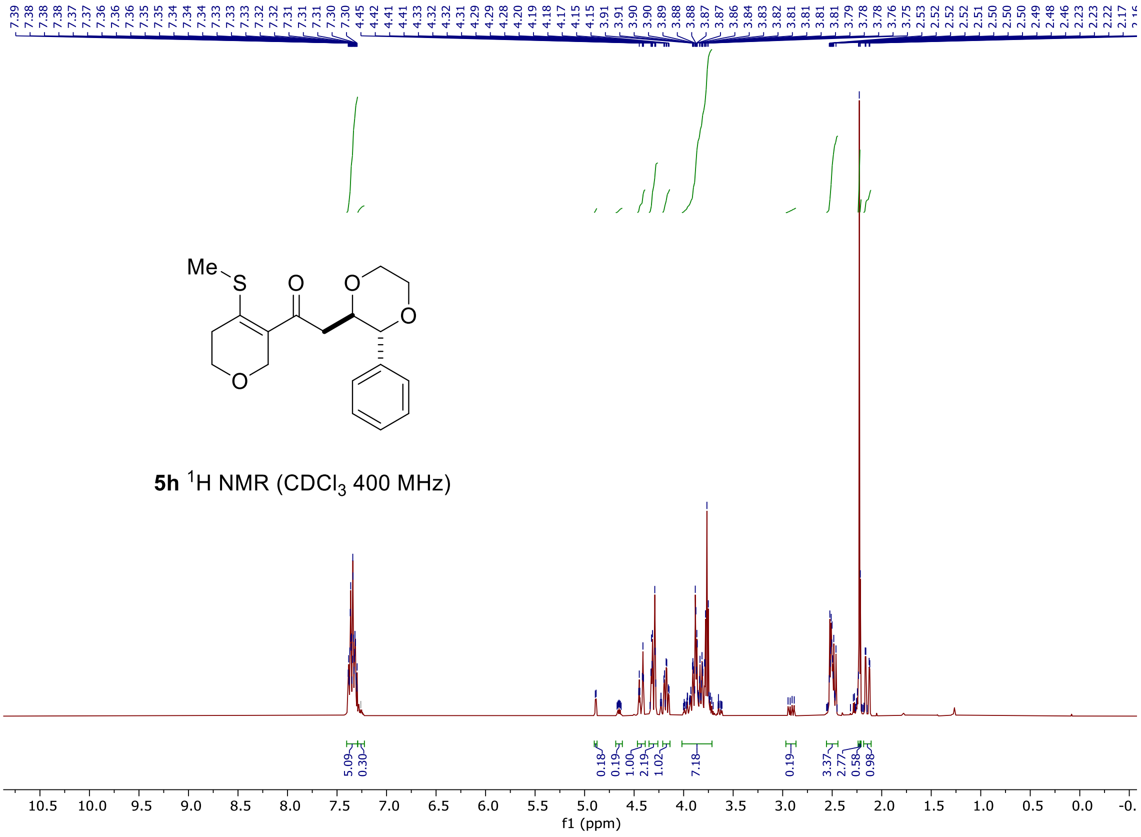
Me S O O O O
5h ^{1}H NMR ($CDCl_3$ 400 MHz)
f1 (ppm)

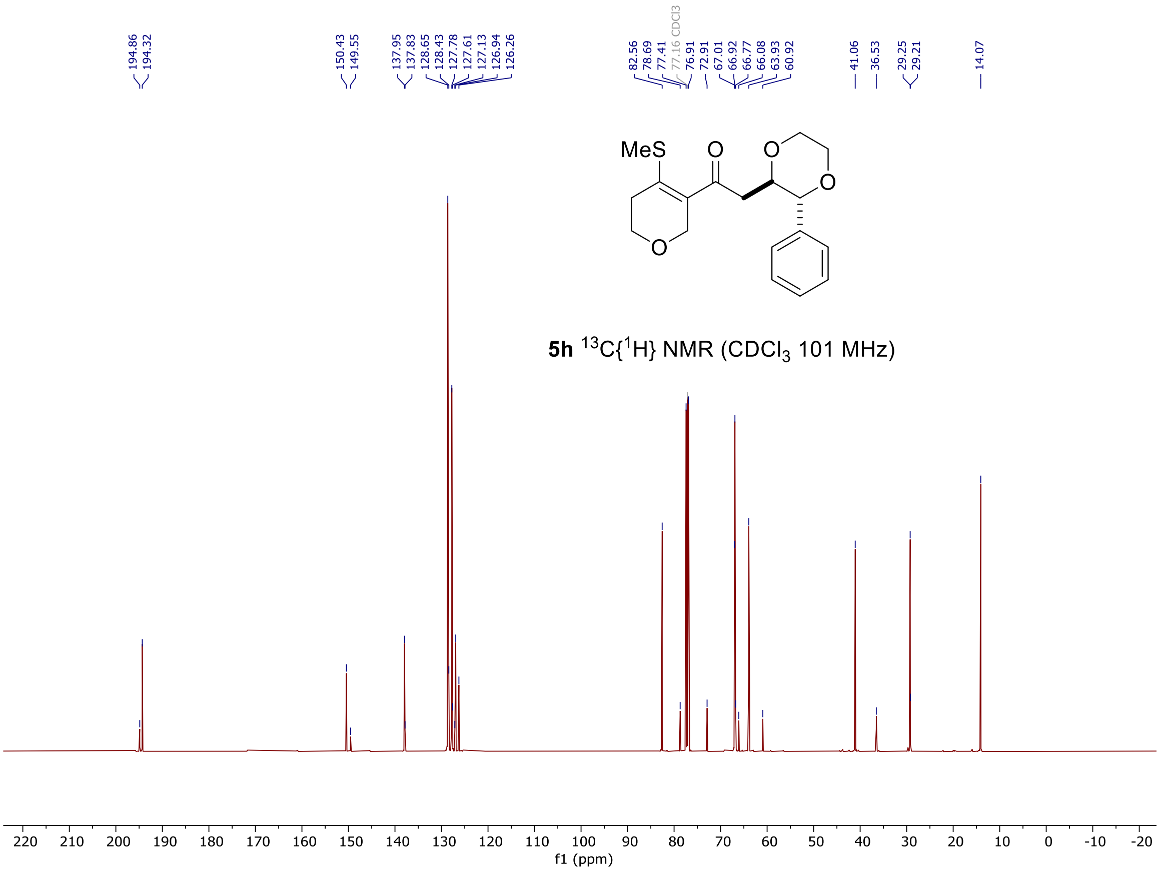
MeS O O O O
5h $^{13}C\{^{1}H\}$ NMR ($CDCl_3$ 101 MHz)
f1 (ppm)

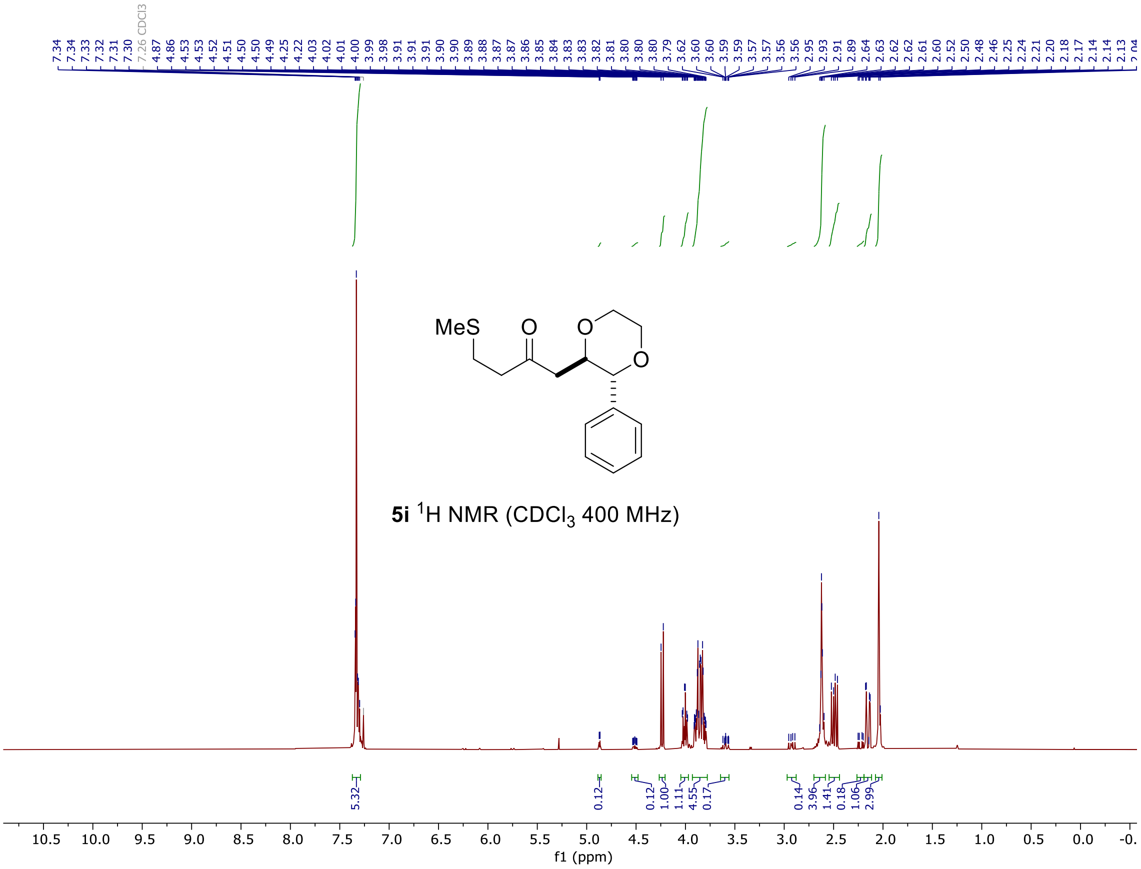
MeS
O
O
O
5i ^{1}H NMR ($CDCl_3$ 400 MHz)
f1 (ppm)

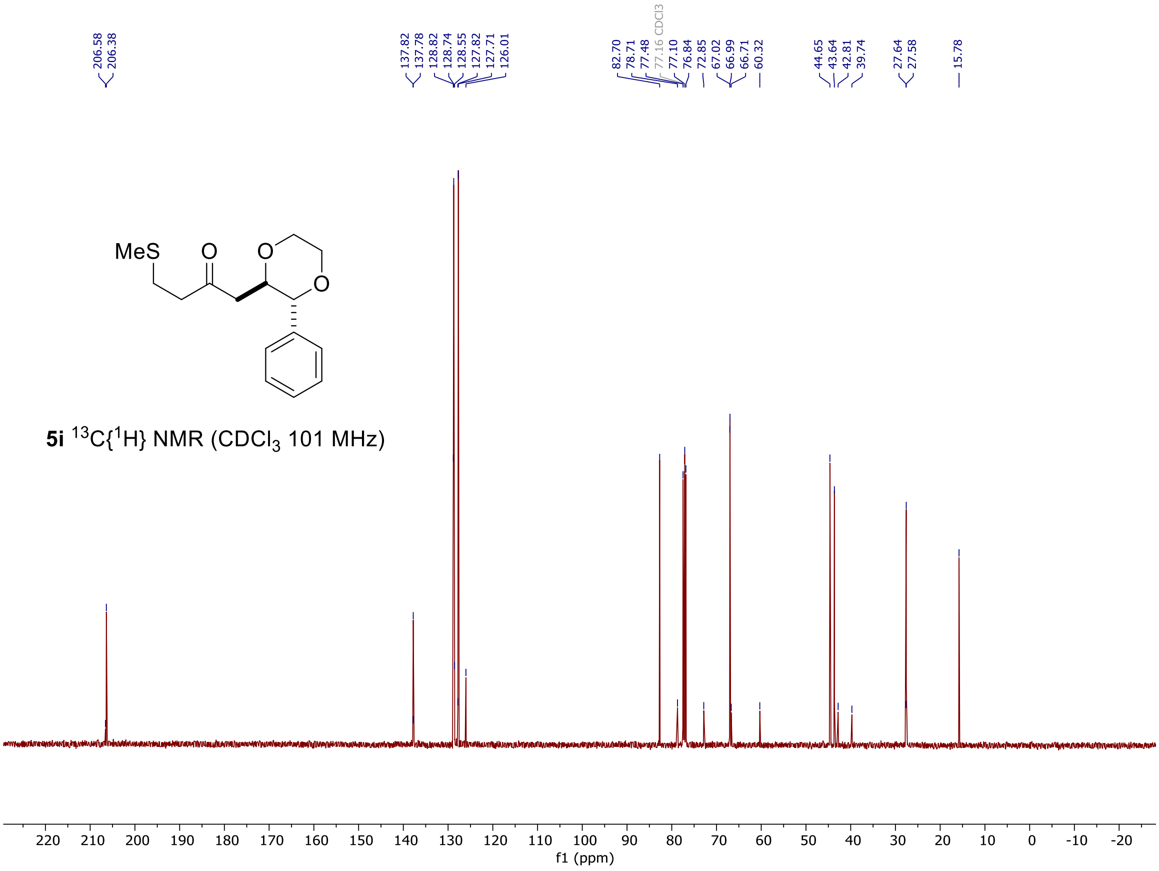
MeS
O
O
O
5i $^{13}C\{^{1}H\}$ NMR ($CDCl_3$ 101 MHz)
f1 (ppm)

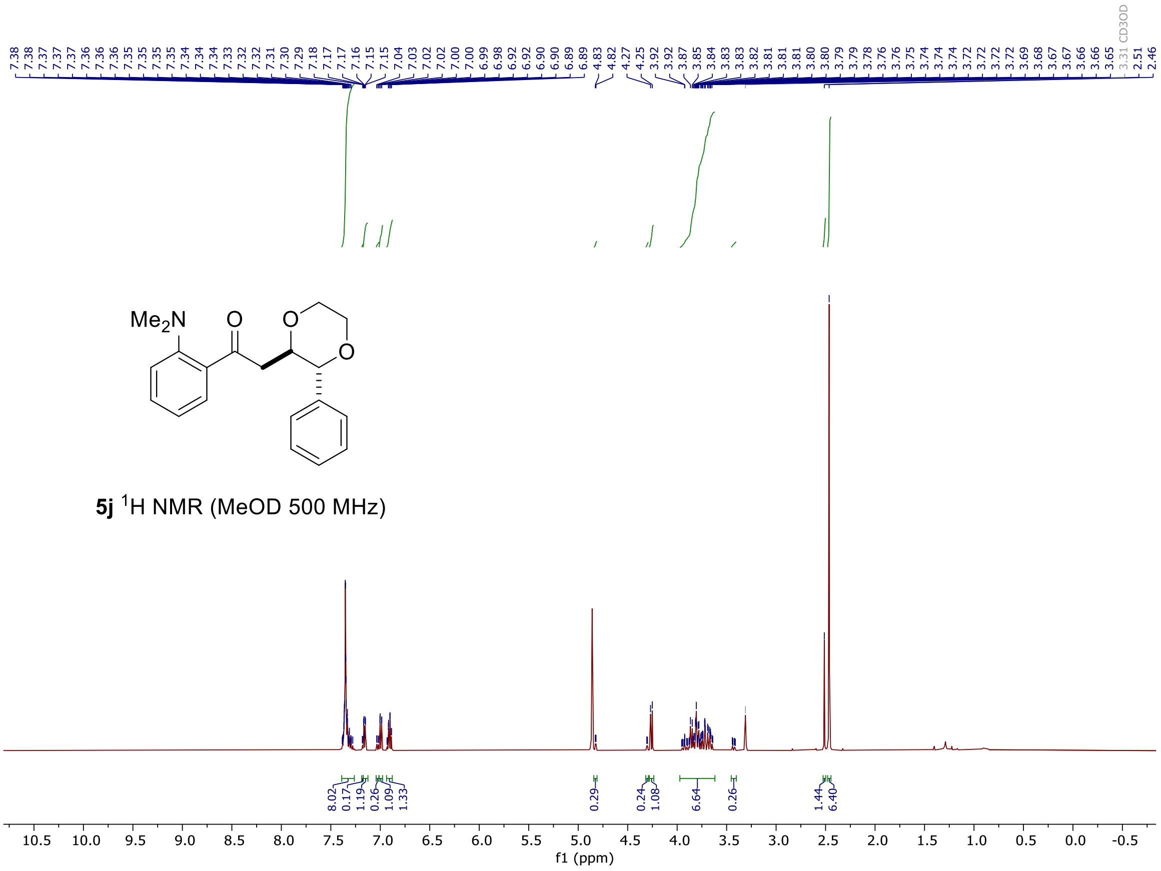

5j ^{1}H NMR (MeOD 500 MHz)

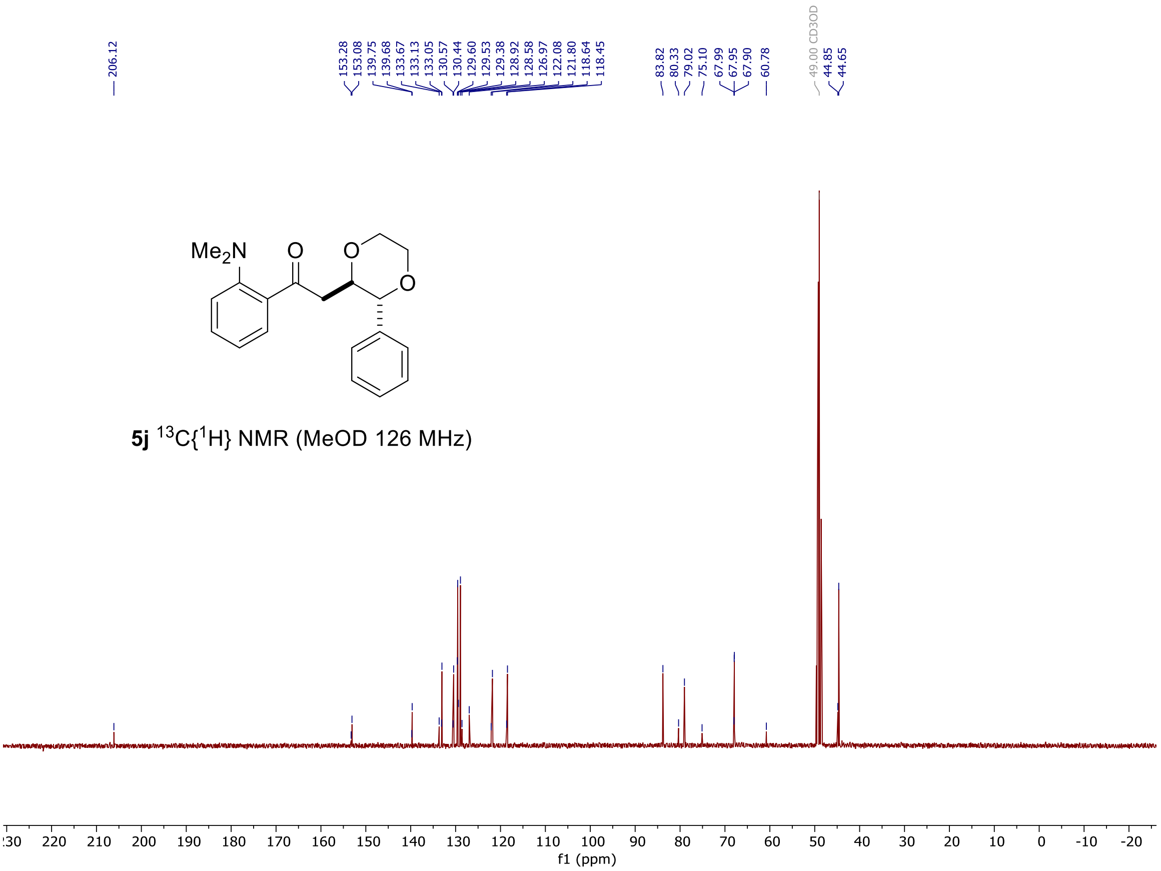

5j ^{13}C{^{1}H} NMR (MeOD 126 MHz)

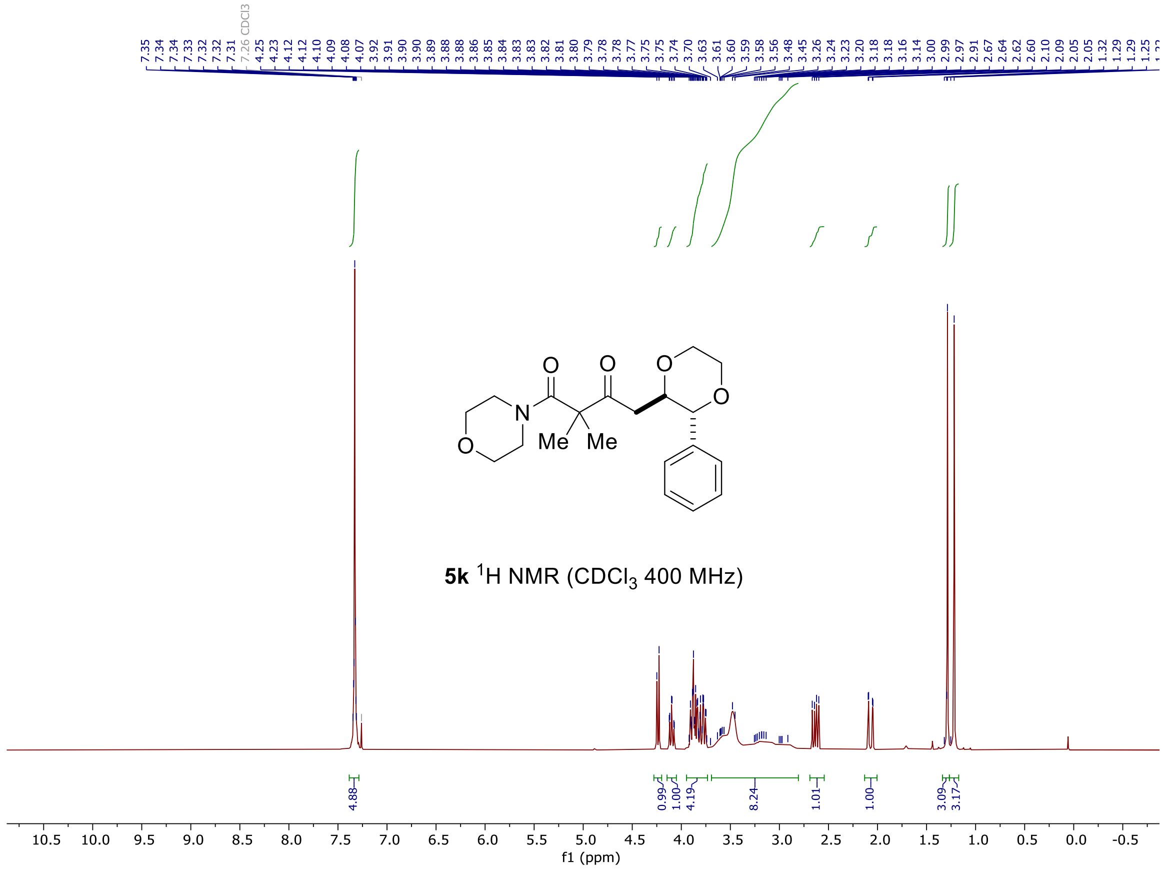
5k ¹H NMR (CDCl3 400 MHz)
Me Me
f1 (ppm)

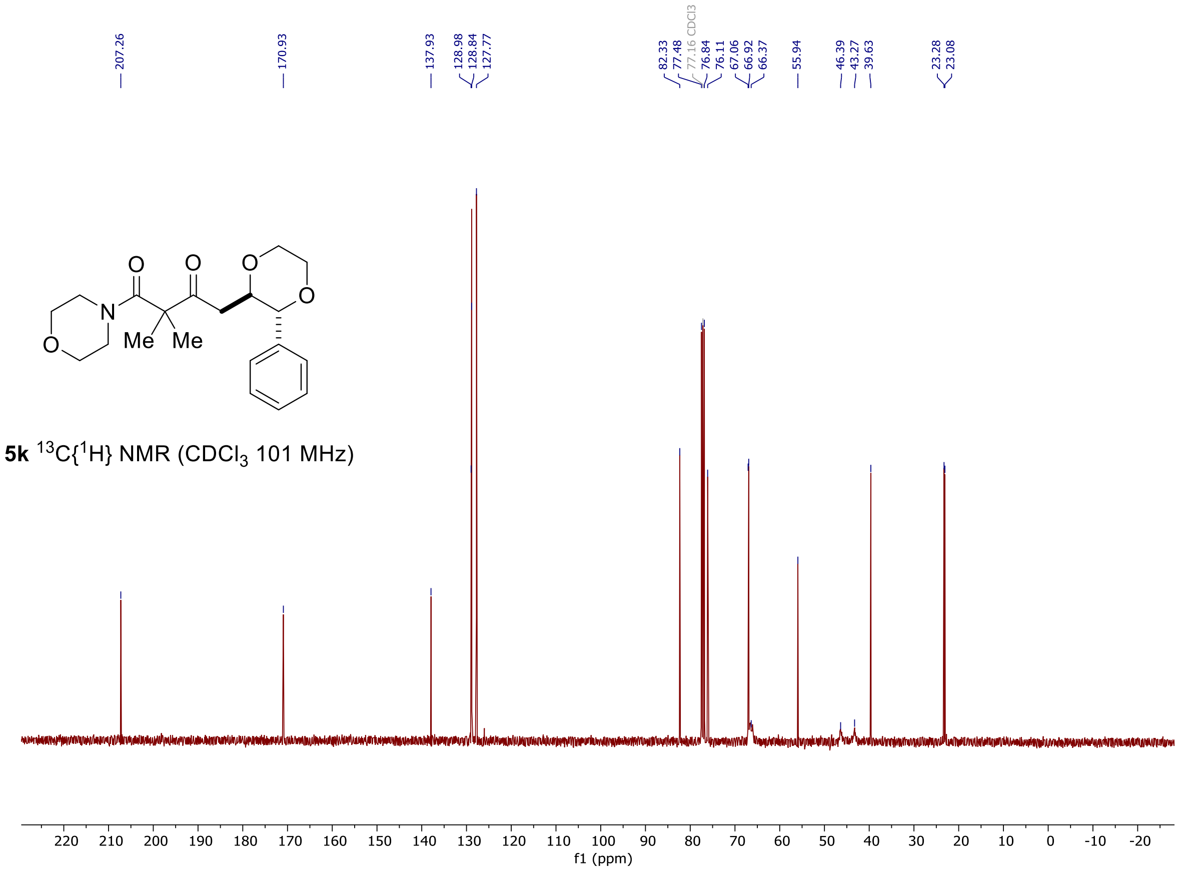
5k ¹³C{¹H} NMR (CDCl3 101 MHz)
Me Me
207.26
170.93
137.93
128.98
128.84
127.77
82.33
77.48
77.16 CDCl3
76.84
76.11
67.06
66.92
66.37
55.94
46.39
43.27
39.63
23.28
23.08
f1 (ppm)

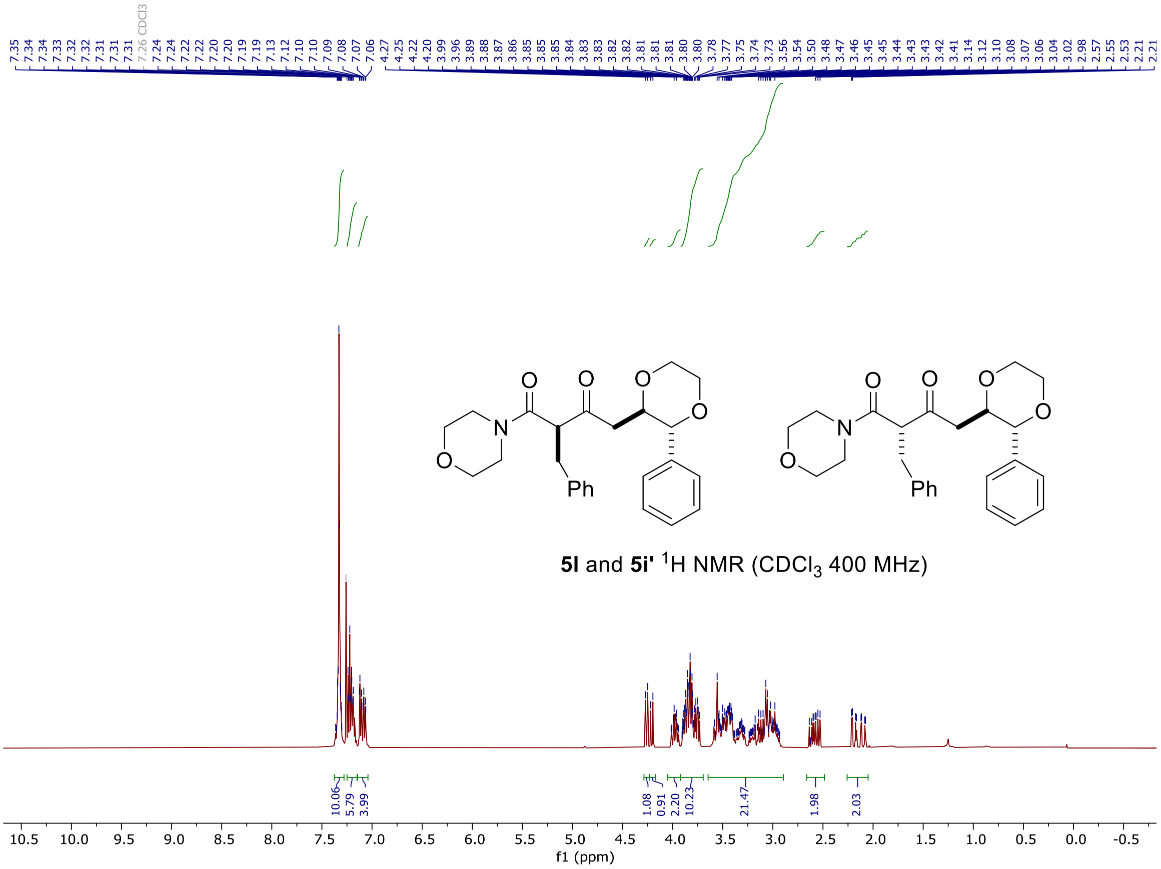

5l and **5i'** ^{1}H NMR ($CDCl_3$ 400 MHz)

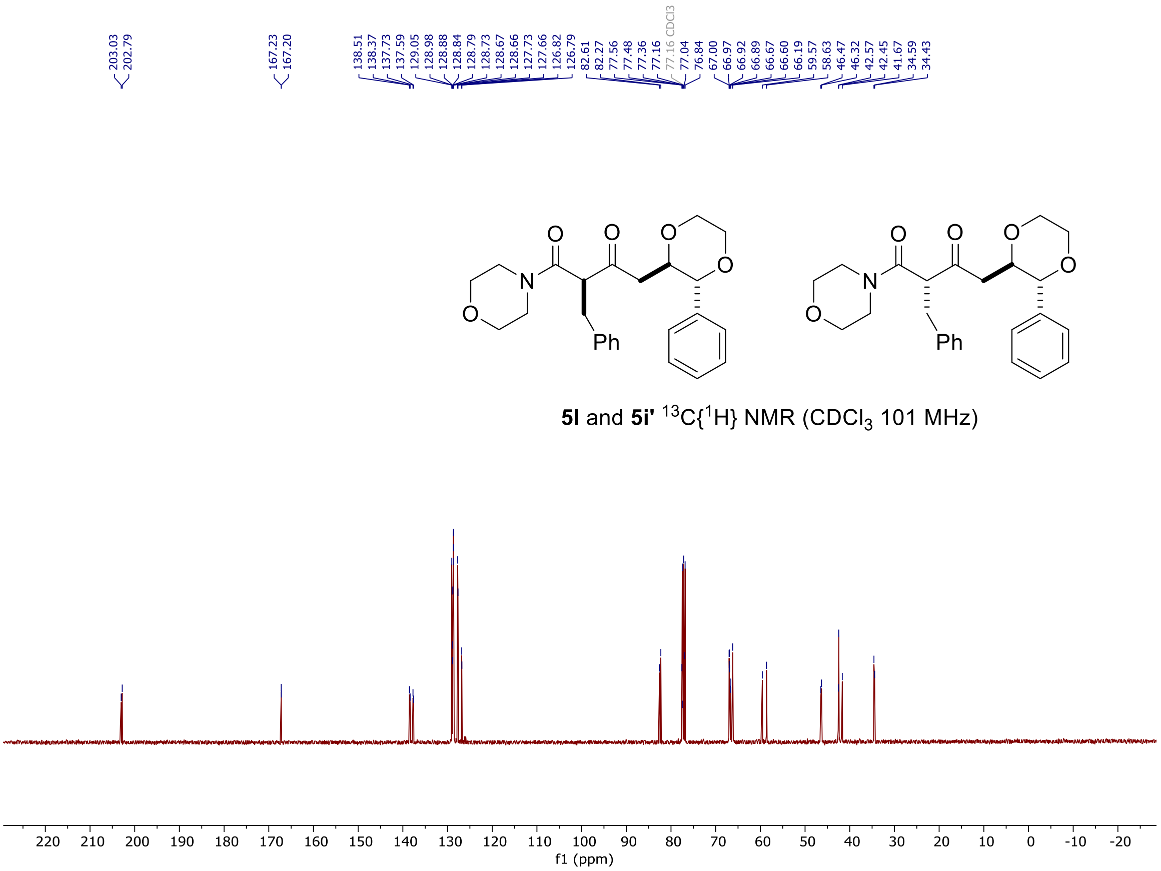

5l and **5i'** $^{13}C\{^{1}H\}$ NMR ($CDCl_3$ 101 MHz)

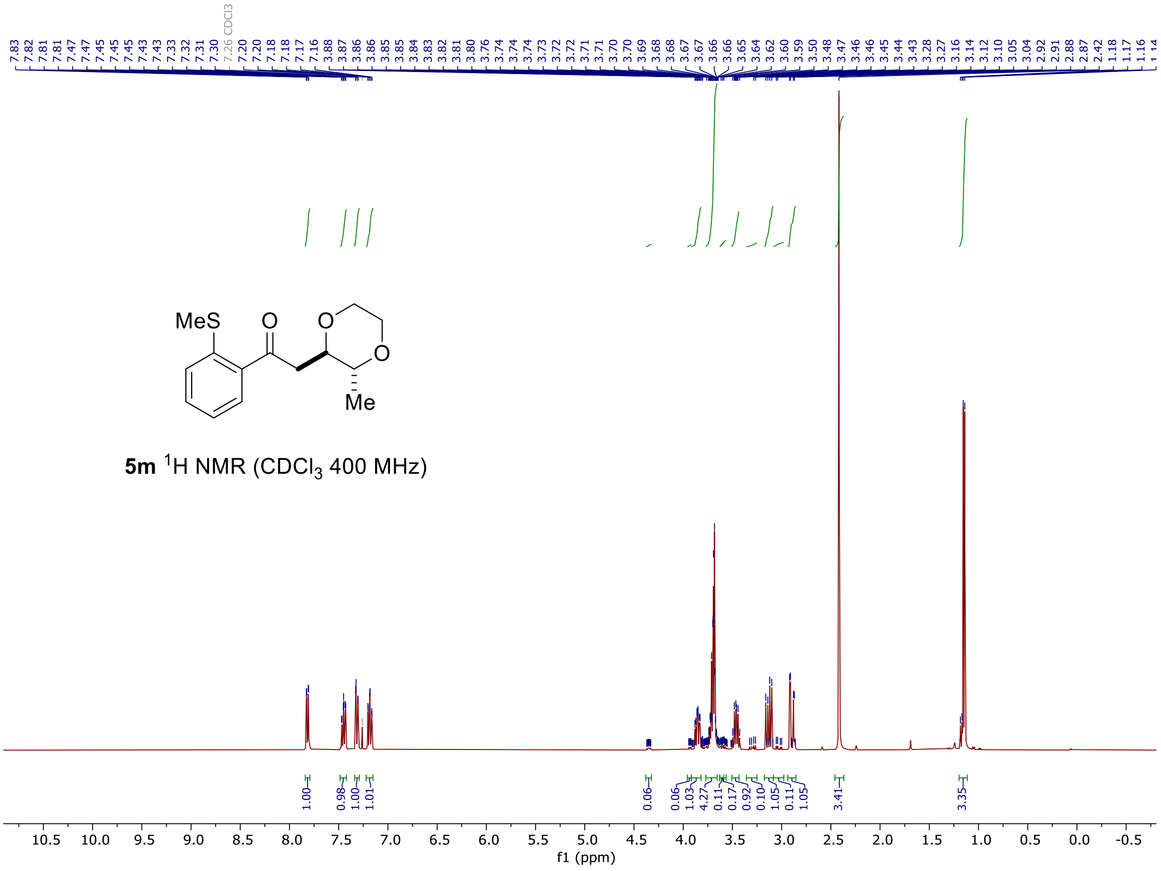

5m ^{1}H NMR ($CDCl_3$ 400 MHz)

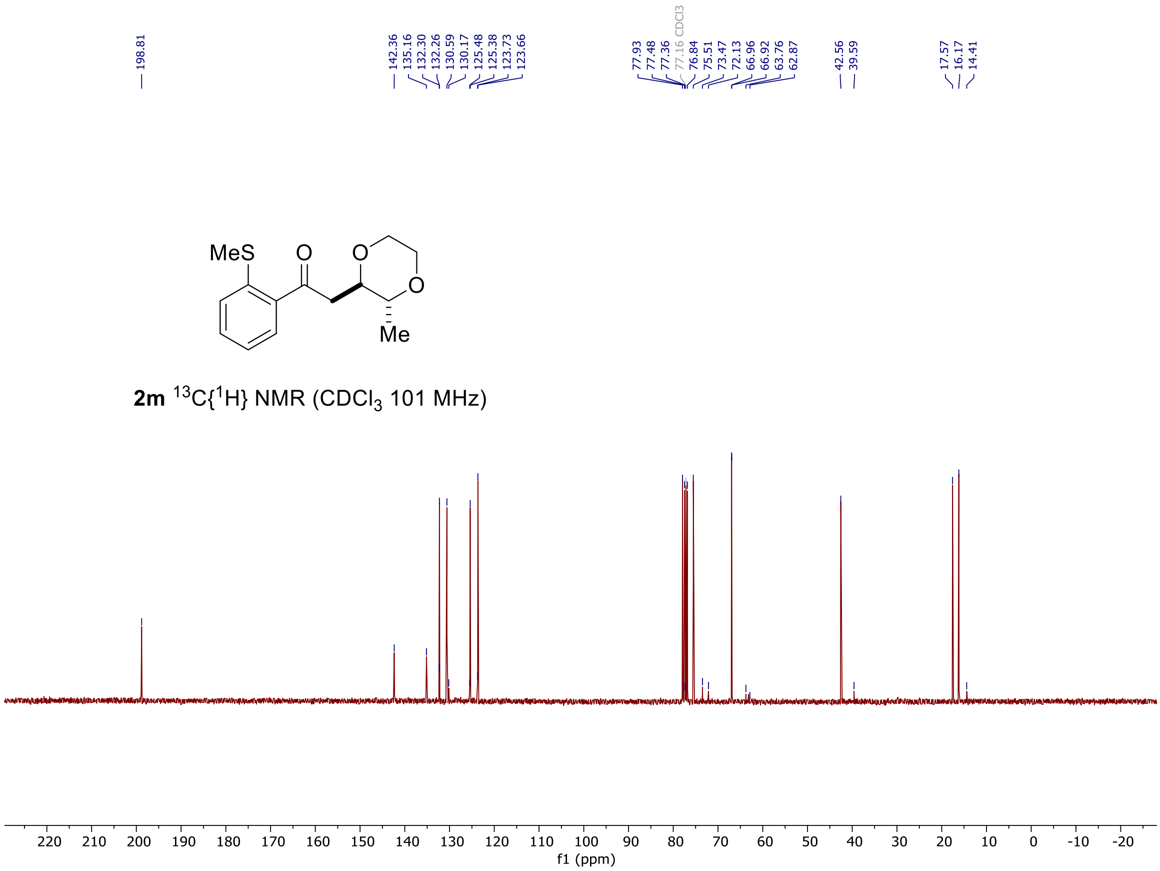

2m $^{13}C\{^{1}H\}$ NMR ($CDCl_3$ 101 MHz)

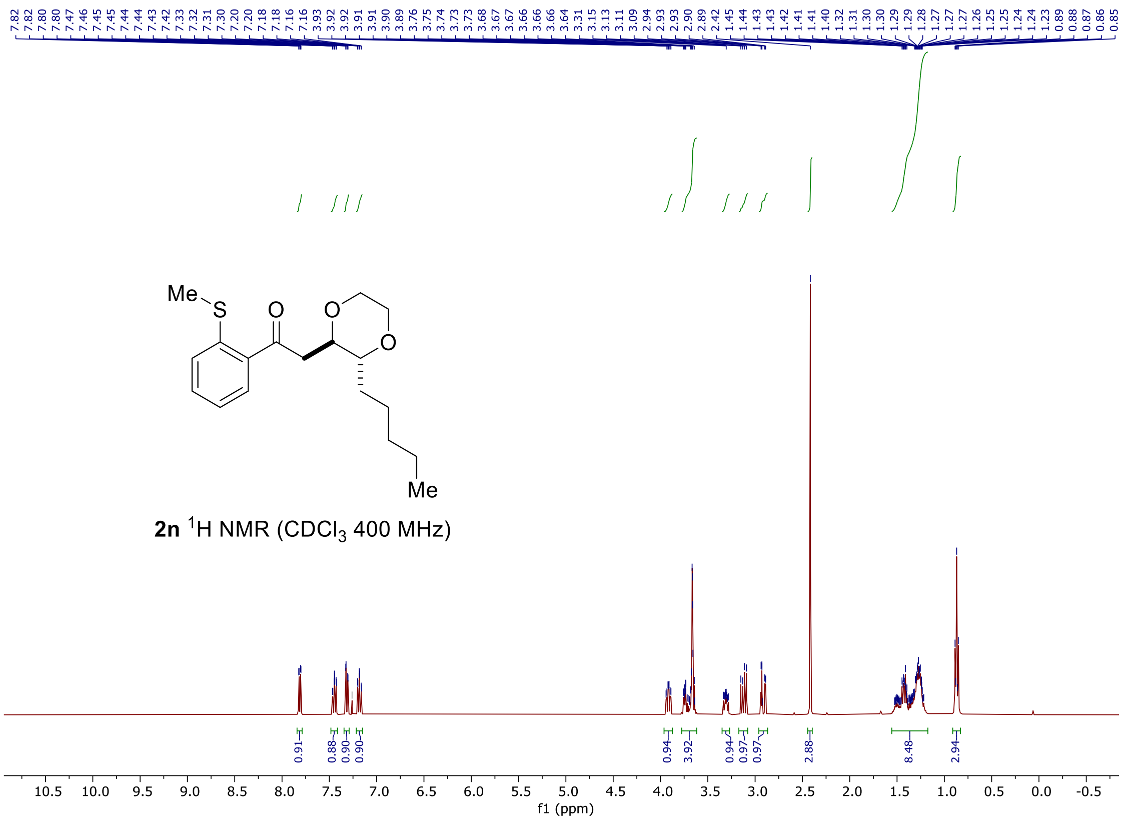

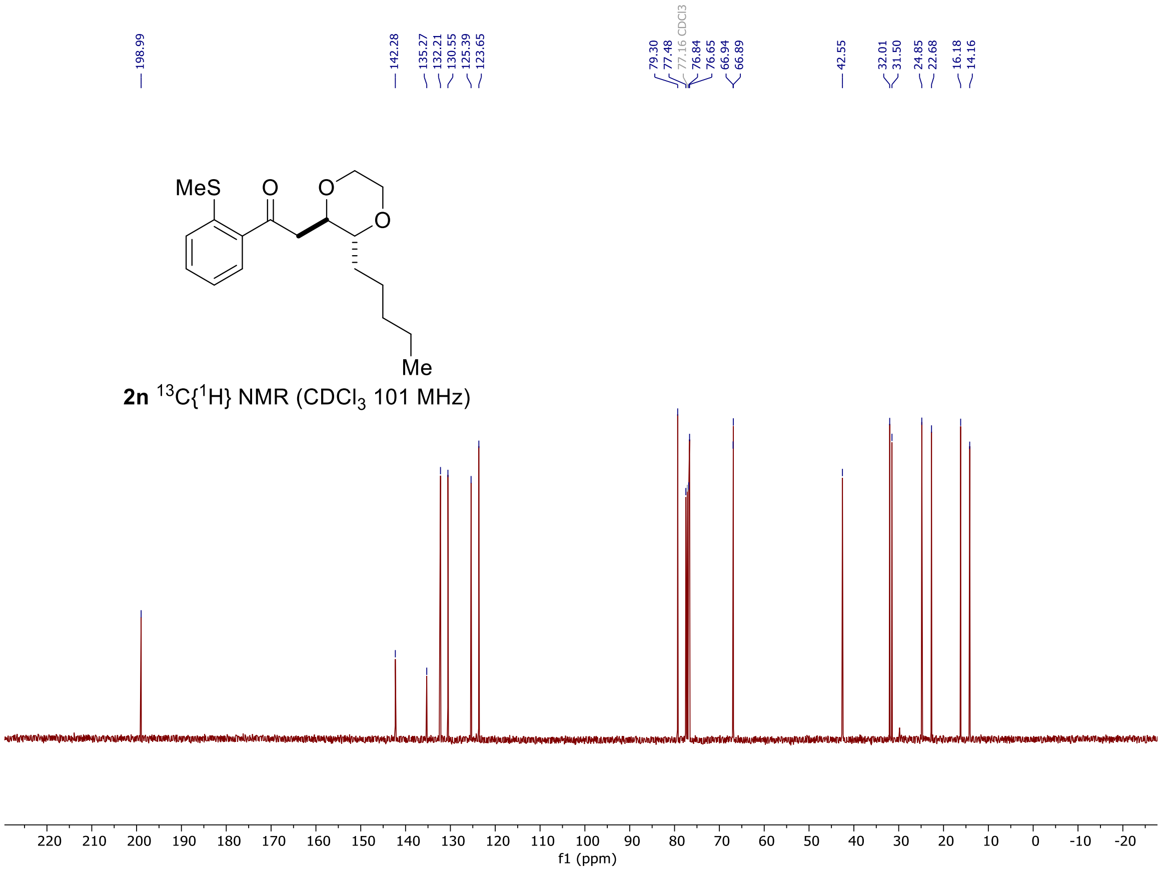

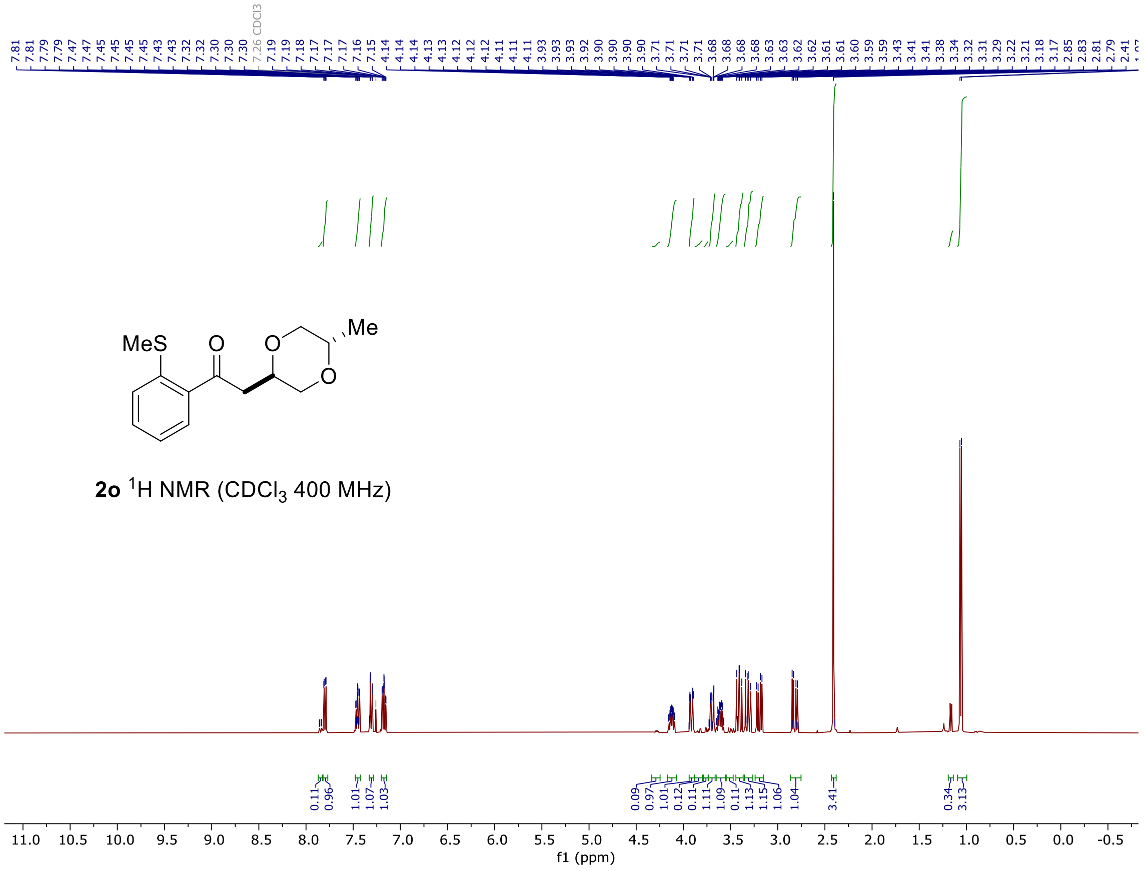
7.81 7.81 7.79 7.79 7.47 7.47 7.45 7.45 7.45 7.43 7.43 7.32 7.32 7.30 7.30 7.26 CDCl3 7.19 7.19 7.18 7.17 7.17 7.17 7.16 7.15 4.14 4.14 4.14 4.13 4.13 4.12 4.12 4.11 4.11 4.11 3.93 3.93 3.93 3.92 3.90 3.90 3.90 3.90 3.71 3.71 3.71 3.68 3.68 3.68 3.68 3.63 3.62 3.62 3.61 3.60 3.59 3.59 3.43 3.41 3.41 3.38 3.34 3.32 3.31 3.29 3.22 3.21 3.18 3.17 2.85 2.83 2.81 2.79 2.41
MeS
O
O
Me
O
2o ¹H NMR (CDCl3 400 MHz)
0.11 0.96 1.01 1.07 1.03 0.09 0.97 1.01 0.12 0.11 1.11 1.09 0.11 1.13 1.15 1.06 1.04 3.41 0.34 3.13
11.0 10.5 10.0 9.5 9.0 8.5 8.0 7.5 7.0 6.5 6.0 5.5 5.0 4.5 4.0 3.5 3.0 2.5 2.0 1.5 1.0 0.5 0.0 -0.5
f1 (ppm)

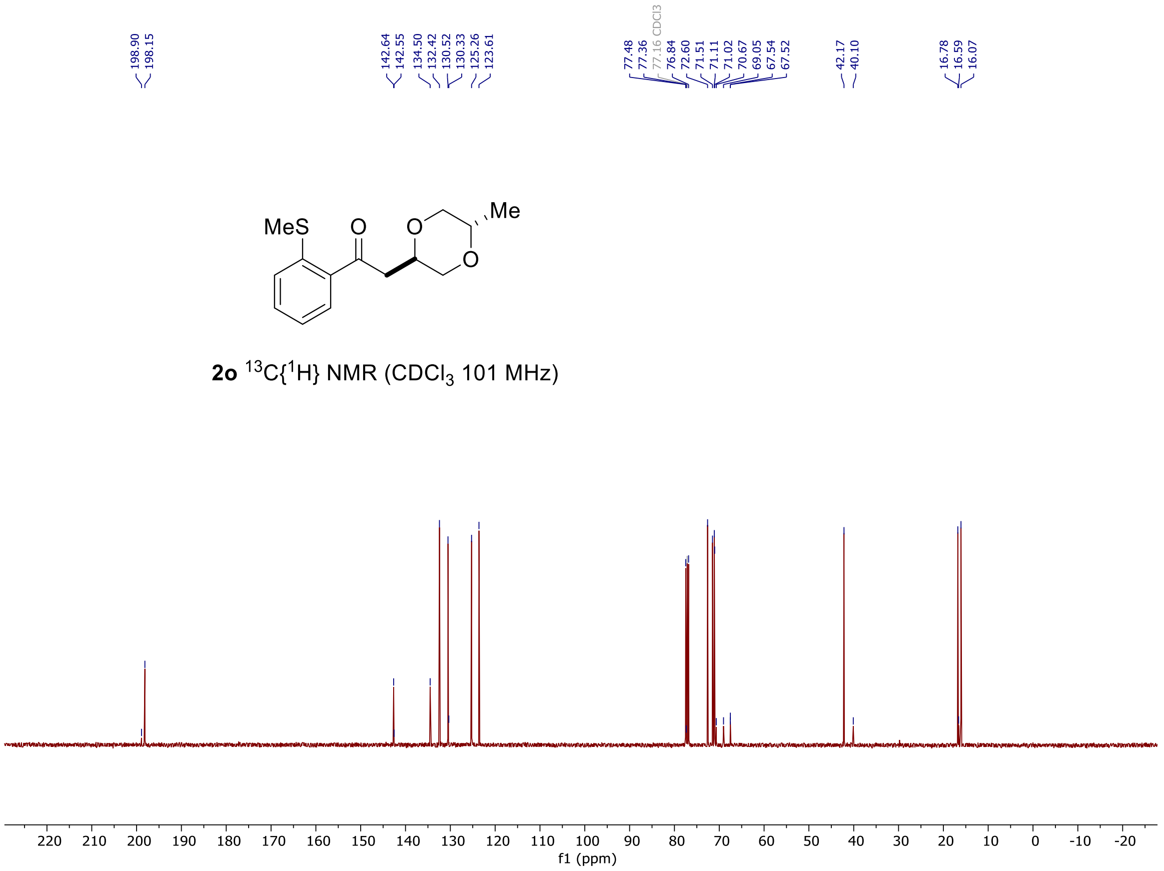
198.90 198.15 142.64 142.55 134.50 132.42 130.52 130.33 125.26 123.61 77.48 77.36 77.16 CDCl3 76.84 72.60 71.51 71.11 71.02 70.67 69.05 67.54 67.52 42.17 40.10 16.78 16.59 16.07
MeS
O
O
Me
O
2o ¹³C{¹H} NMR (CDCl3 101 MHz)
220 210 200 190 180 170 160 150 140 130 120 110 100 90 80 70 60 50 40 30 20 10 0 -10 -20
f1 (ppm)

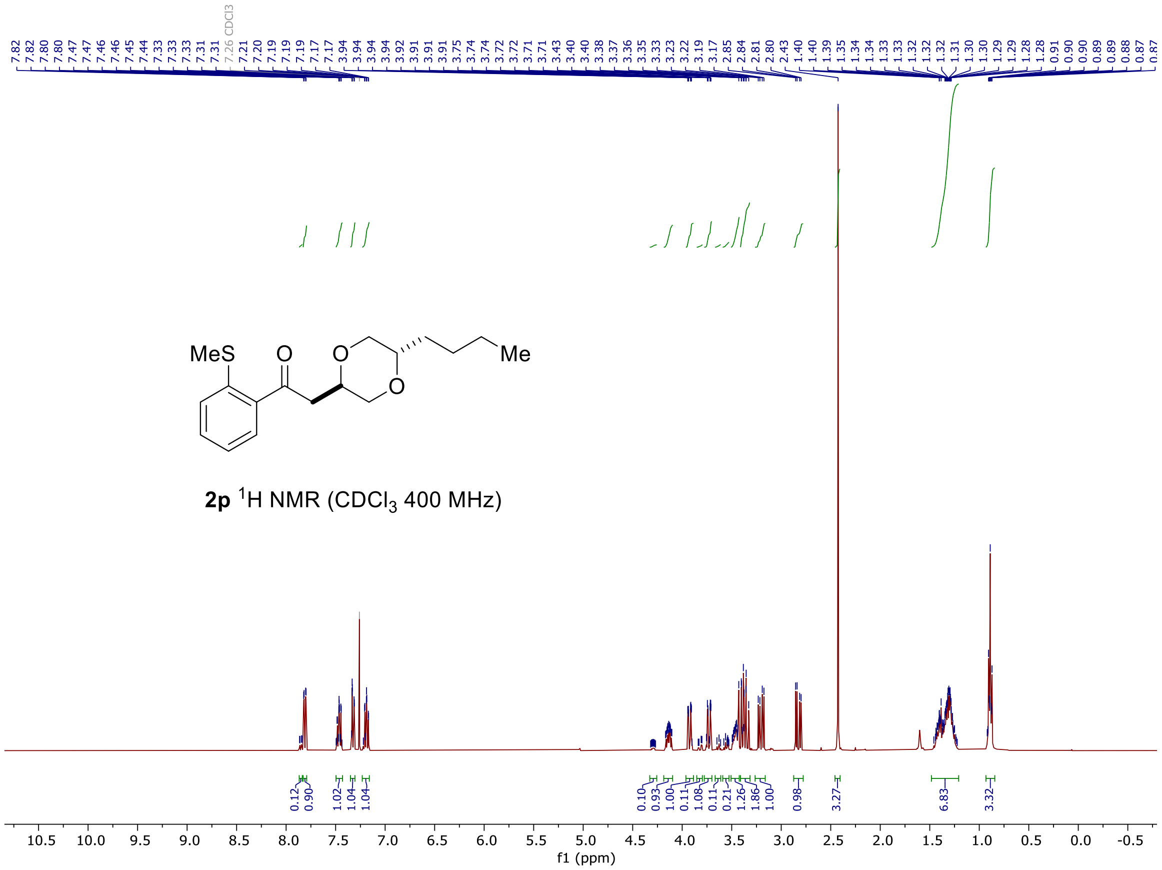

2p ^{1}H NMR ($CDCl_3$ 400 MHz)

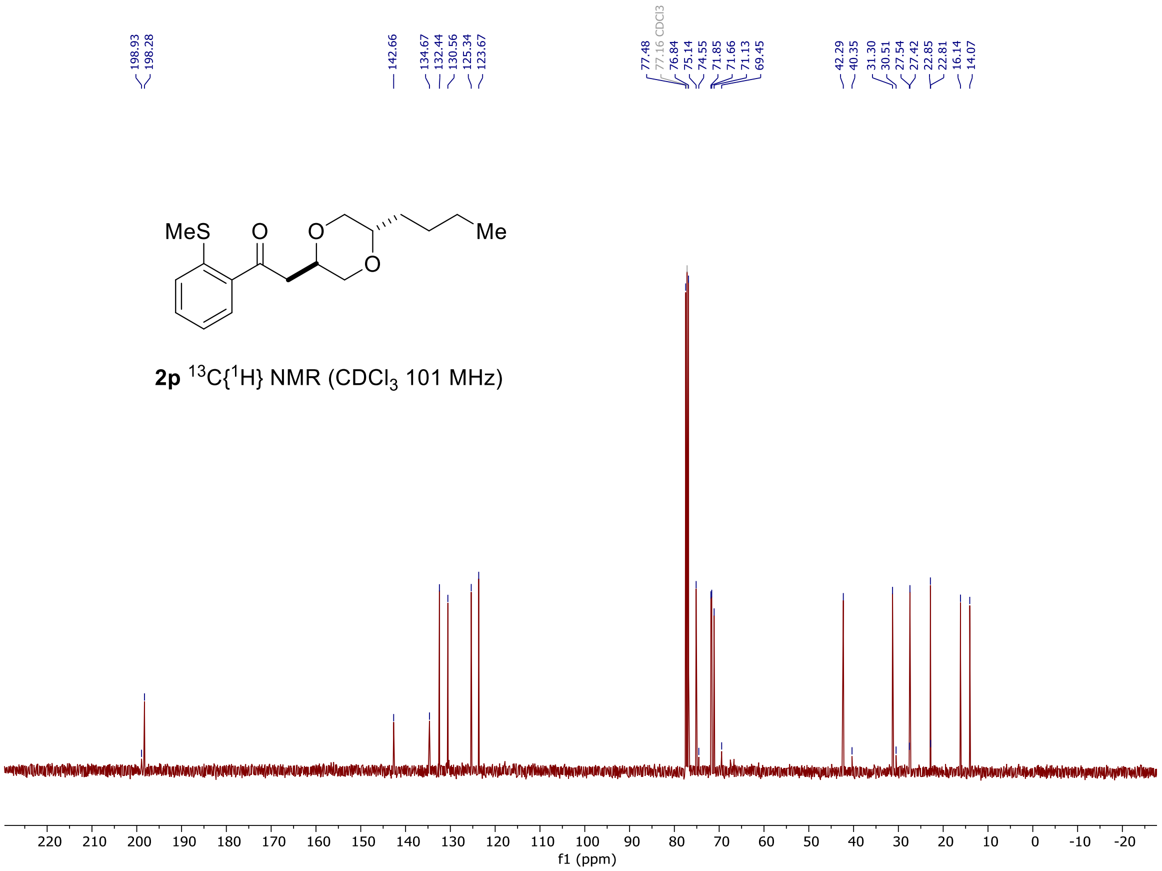

2p $^{13}C\{^{1}H\}$ NMR ($CDCl_3$ 101 MHz)

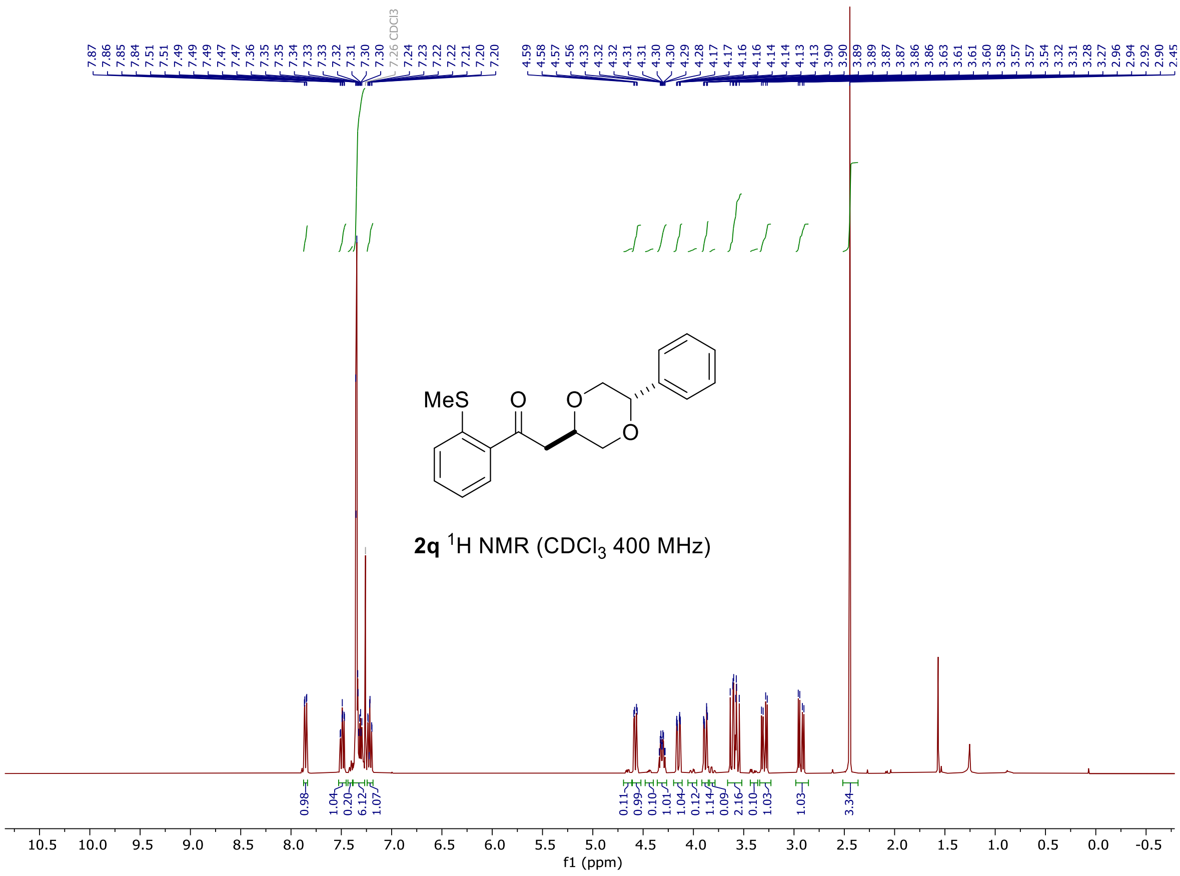
2q ^{1}H NMR ($CDCl_3$ 400 MHz)
f1 (ppm)

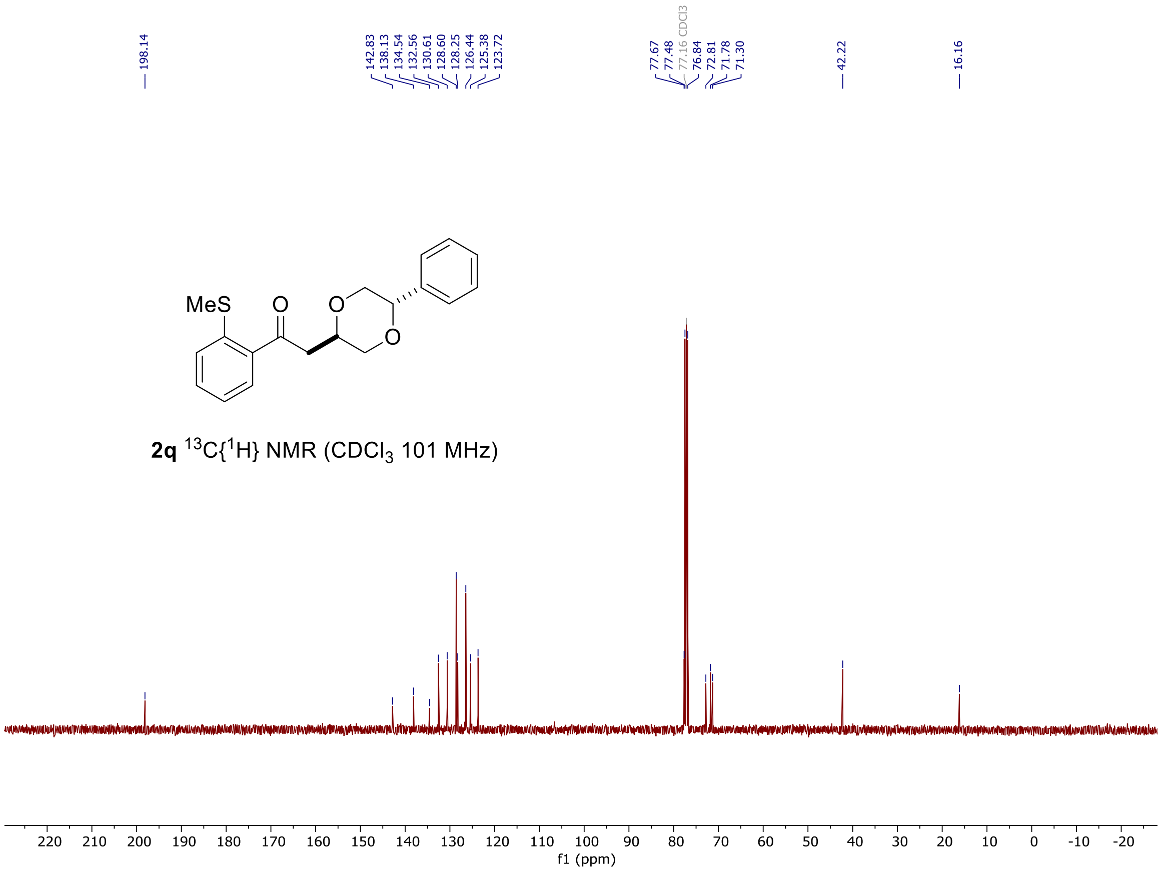
2q $^{13}C\{^{1}H\}$ NMR ($CDCl_3$ 101 MHz)
f1 (ppm)

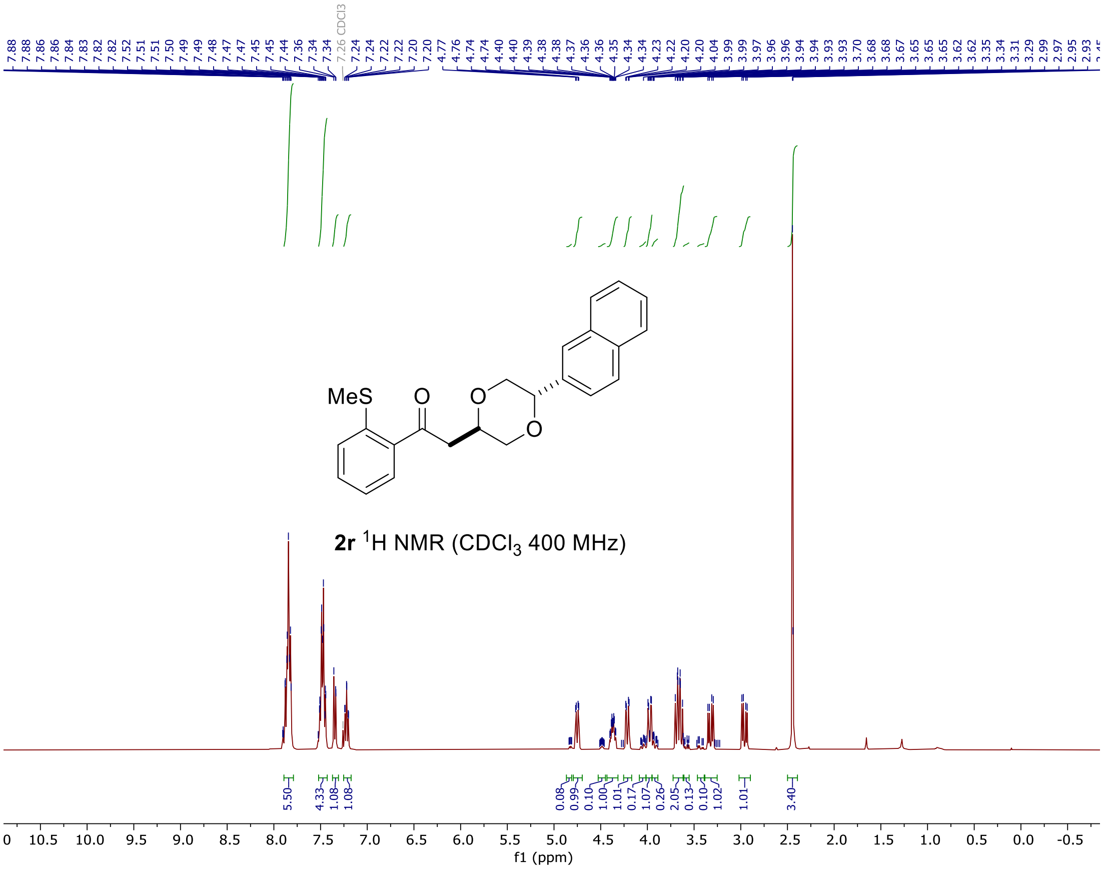

2r ^{1}H NMR ($CDCl_3$ 400 MHz)

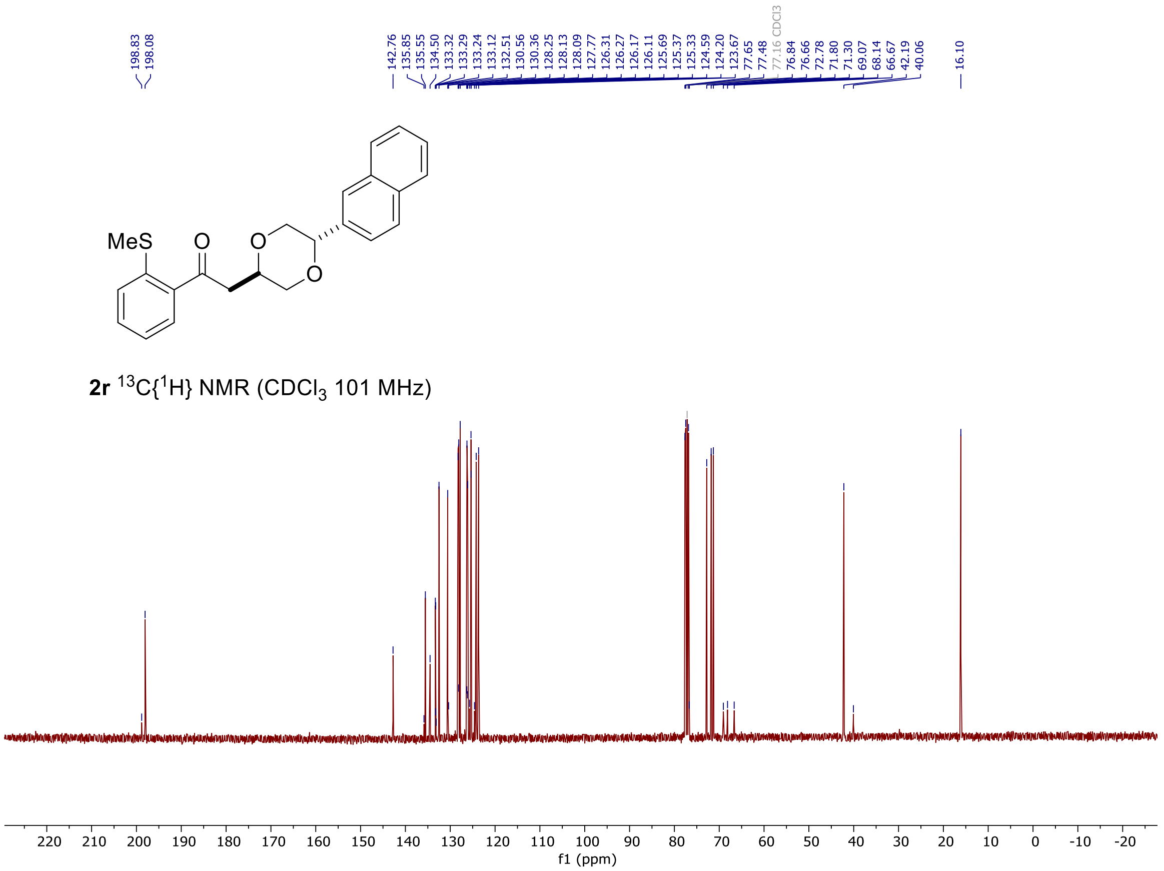

2r $^{13}C\{^{1}H\}$ NMR ($CDCl_3$ 101 MHz)

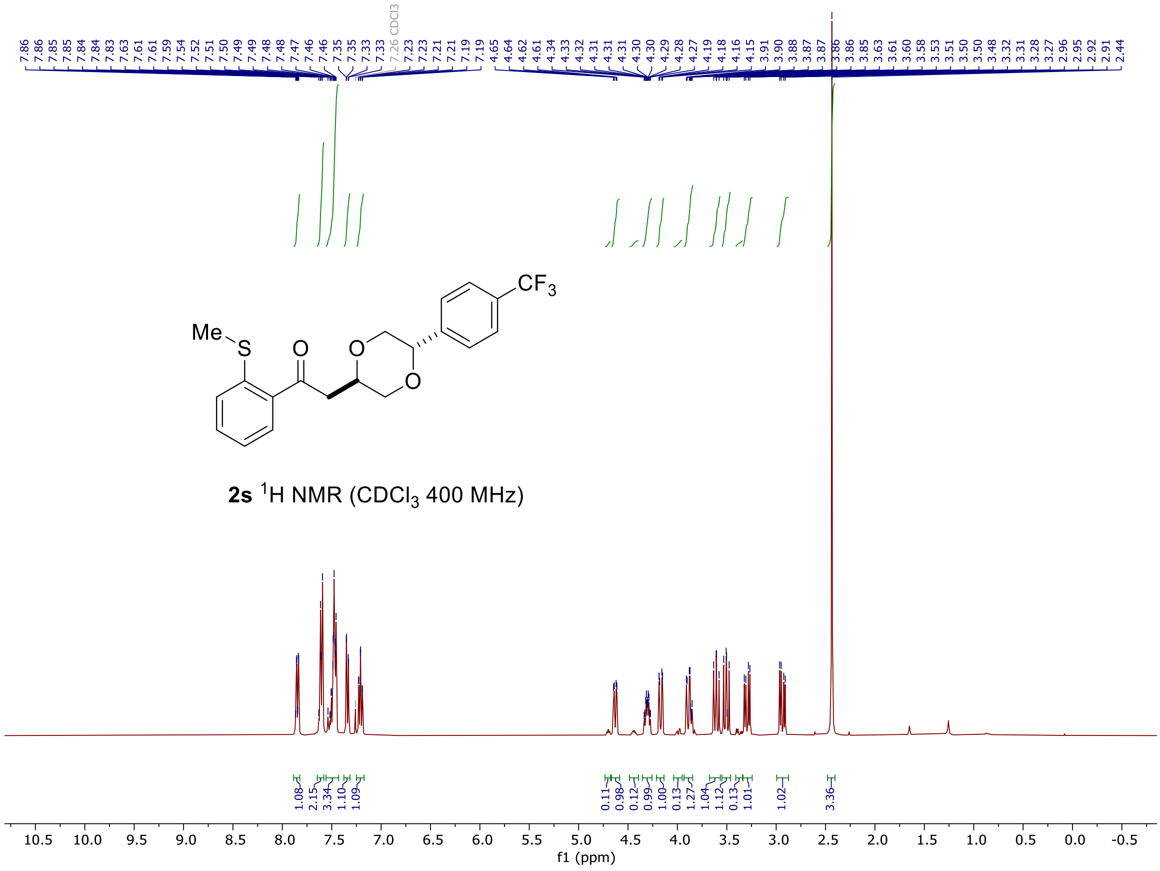
CF3
Me
S
O
O
O
2s ¹H NMR (CDCl3 400 MHz)
f1 (ppm)

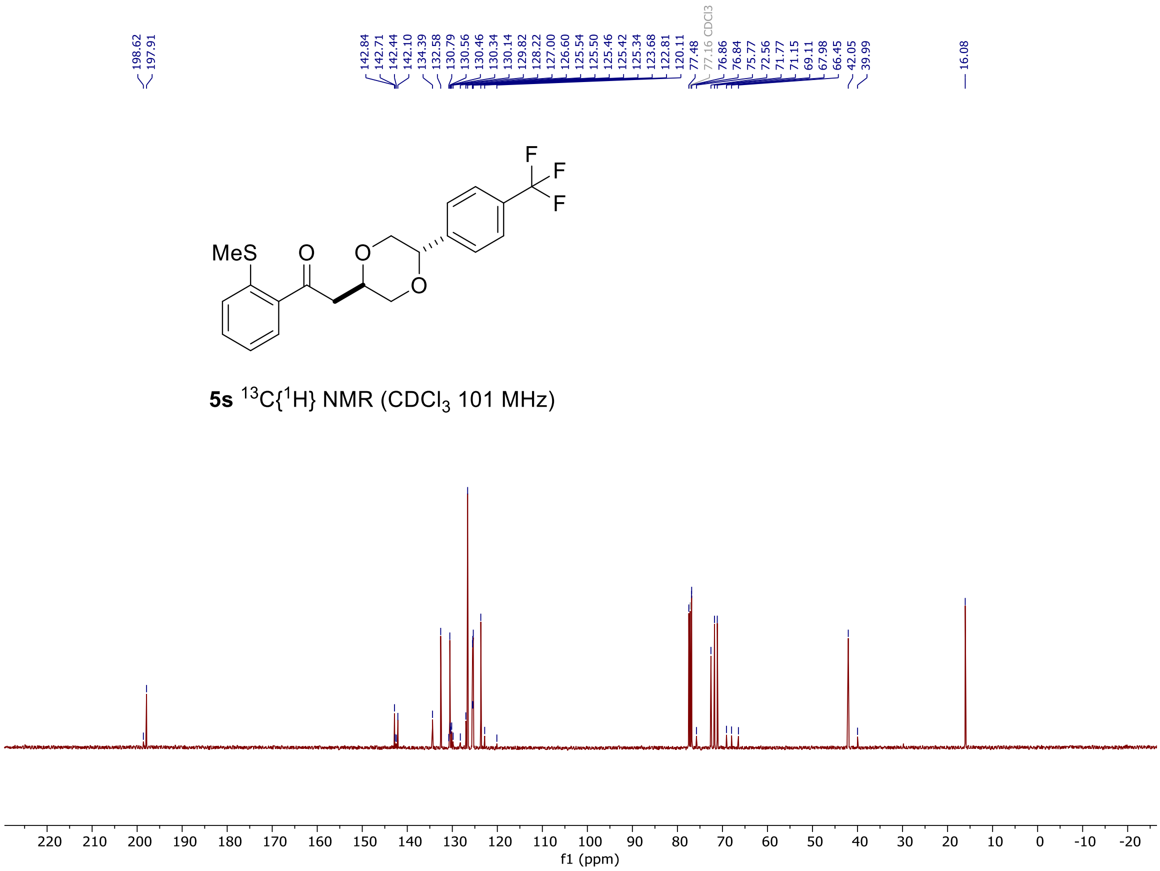
F
F
F
MeS
O
O
O
5s ¹³C{¹H} NMR (CDCl3 101 MHz)
f1 (ppm)

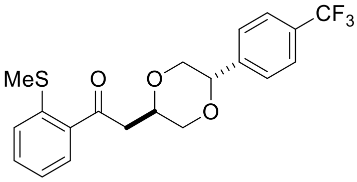

5s $^{19}F\{^{1}H\}$ NMR ($CDCl_3$ 377 MHz)

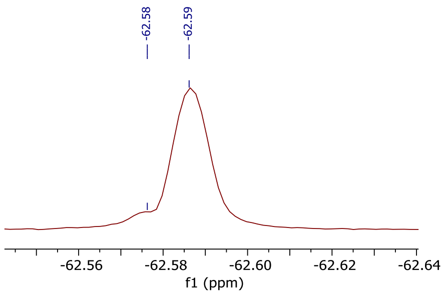

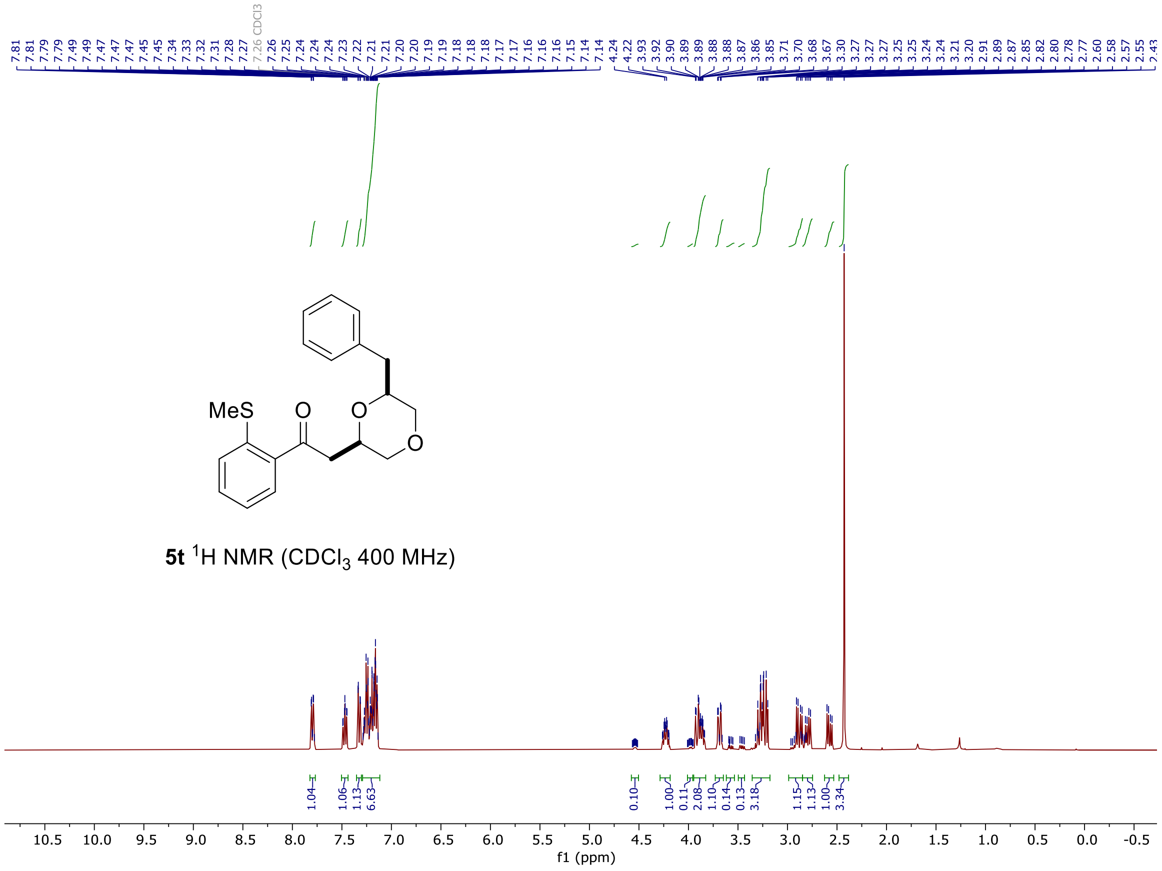

5t ^{1}H NMR ($CDCl_3$ 400 MHz)

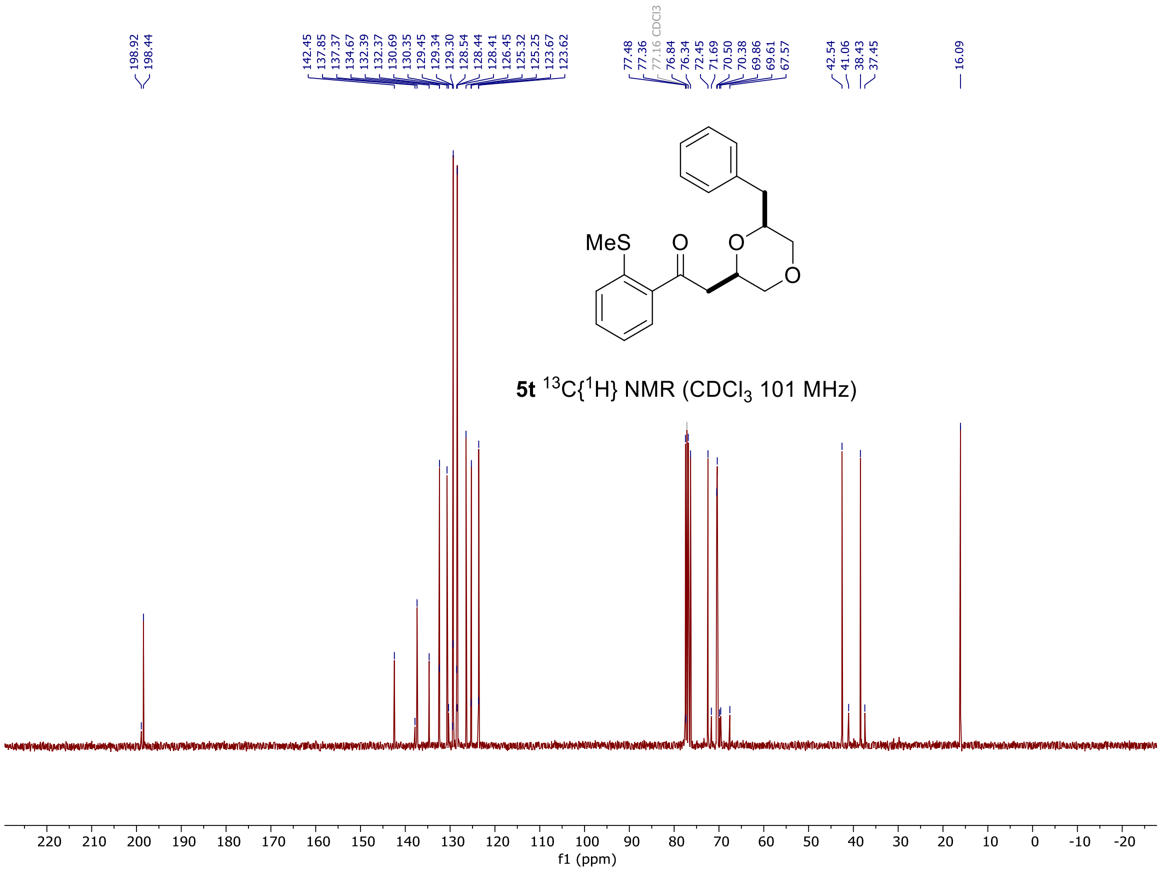

5t $^{13}C\{^1H\}$ NMR ($CDCl_3$ 101 MHz)

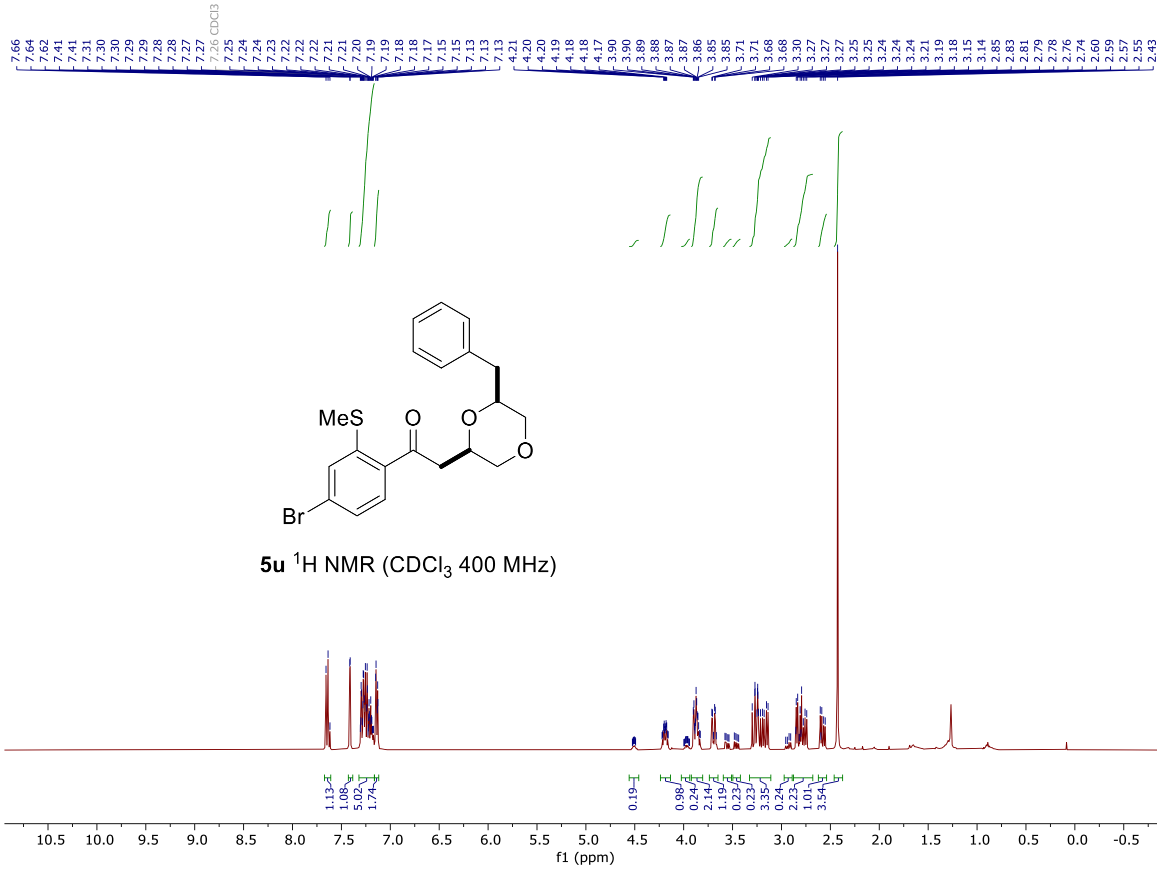
7.66 7.64 7.62 7.41 7.41 7.31 7.30 7.29 7.29 7.28 7.28 7.27 7.27 7.26 CDCl3 7.25 7.24 7.24 7.23 7.22 7.22 7.22 7.21 7.21 7.20 7.19 7.19 7.18 7.18 7.17 7.15 7.15 7.13 7.13 7.13 4.21 4.20 4.19 4.18 4.18 4.17 3.90 3.89 3.88 3.87 3.87 3.86 3.85 3.85 3.71 3.71 3.68 3.30 3.27 3.27 3.25 3.25 3.24 3.24 3.24 3.21 3.19 3.18 3.15 3.14 2.85 2.83 2.81 2.79 2.78 2.76 2.74 2.60 2.59 2.57 2.55 2.43
MeS
O
O
O
Br
5u ^{1}H NMR ($CDCl_3$ 400 MHz)
1.13 1.08 5.02 1.74 0.19 0.98 0.24 2.14 1.19 0.23 0.23 3.35 0.24 2.23 1.01 3.54
10.5 10.0 9.5 9.0 8.5 8.0 7.5 7.0 6.5 6.0 5.5 5.0 4.5 4.0 3.5 3.0 2.5 2.0 1.5 1.0 0.5 0.0 -0.5
f1 (ppm)

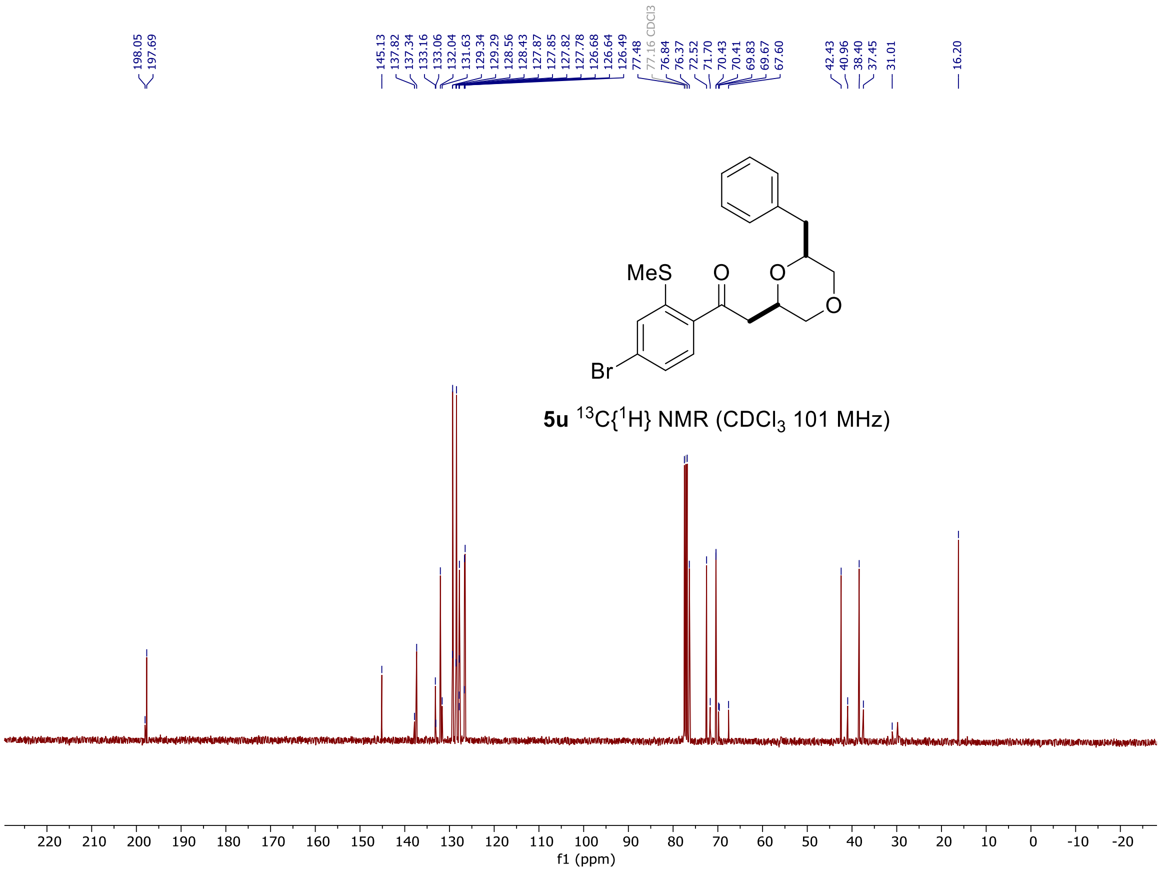
198.05 197.69 145.13 137.82 137.34 133.16 133.06 132.04 131.63 129.34 129.29 128.56 128.43 127.87 127.85 127.82 127.78 126.68 126.64 126.49 77.48 77.16 CDCl3 76.84 76.37 72.52 71.70 70.43 70.41 69.83 69.67 67.60 42.43 40.96 38.40 37.45 31.01 16.20
MeS
O
O
O
Br
5u ^{13}C{^{1}H} NMR ($CDCl_3$ 101 MHz)
220 210 200 190 180 170 160 150 140 130 120 110 100 90 80 70 60 50 40 30 20 10 0 -10 -20
f1 (ppm)

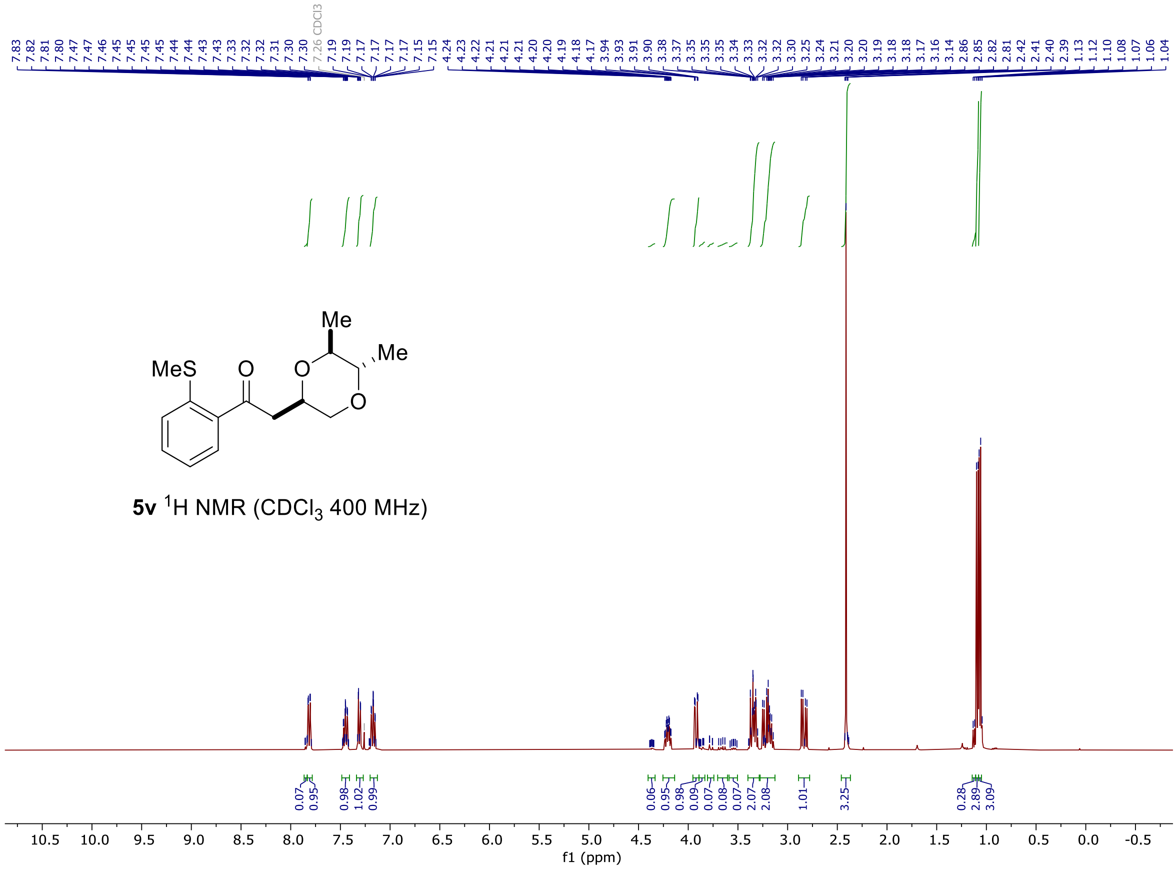
7.83 7.82 7.81 7.80 7.47 7.47 7.46 7.45 7.45 7.45 7.45 7.44 7.44 7.43 7.43 7.33 7.32 7.32 7.31 7.30 7.30 7.26 CDCl3 7.19 7.19 7.17 7.17 7.17 7.17 7.15 7.15 4.24 4.23 4.22 4.21 4.21 4.21 4.20 4.20 4.19 4.18 4.17 3.94 3.93 3.91 3.90 3.38 3.37 3.35 3.35 3.35 3.34 3.33 3.32 3.32 3.30 3.25 3.24 3.21 3.20 3.20 3.19 3.18 3.18 3.17 3.16 3.14 2.86 2.85 2.82 2.81 2.42 2.41 2.40 2.39 1.13 1.12 1.10 1.08 1.07 1.06 1.04
MeS
O
O
Me
Me
O
5v ¹H NMR (CDCl3 400 MHz)
0.07 0.95 0.98 1.02 0.99 0.06 0.95 0.98 0.09 0.07 0.08 0.07 2.07 2.08 1.01 3.25 0.28 2.89 3.09
10.5 10.0 9.5 9.0 8.5 8.0 7.5 7.0 6.5 6.0 5.5 5.0 4.5 4.0 3.5 3.0 2.5 2.0 1.5 1.0 0.5 0.0 -0.5
f1 (ppm)

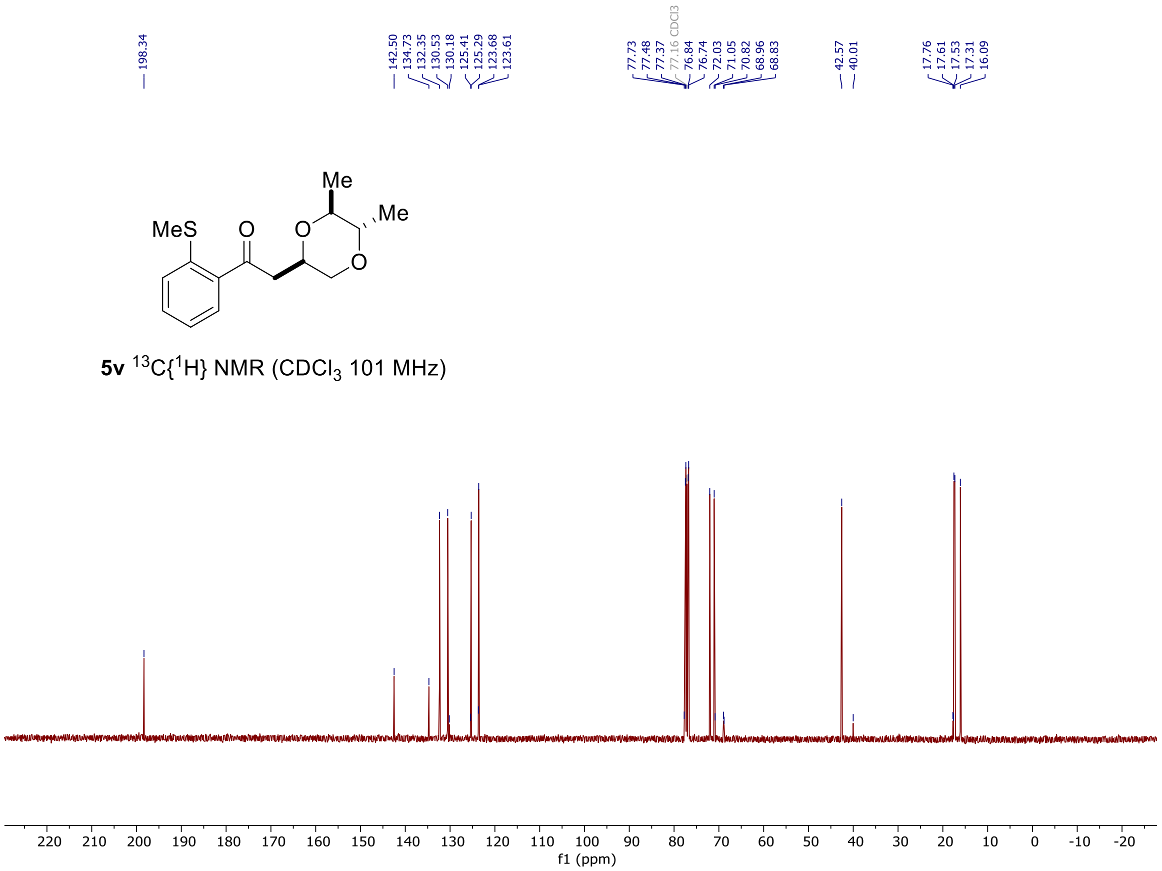
198.34
142.50 134.73 132.35 130.53 130.18 125.41 125.29 123.68 123.61
77.73 77.48 77.37 77.16 CDCl3 76.84 76.74 72.03 71.05 70.82 68.96 68.83
42.57 40.01
17.76 17.61 17.53 17.31 16.09
MeS
O
O
Me
Me
O
5v ¹³C{¹H} NMR (CDCl3 101 MHz)
220 210 200 190 180 170 160 150 140 130 120 110 100 90 80 70 60 50 40 30 20 10 0 -10 -20
f1 (ppm)

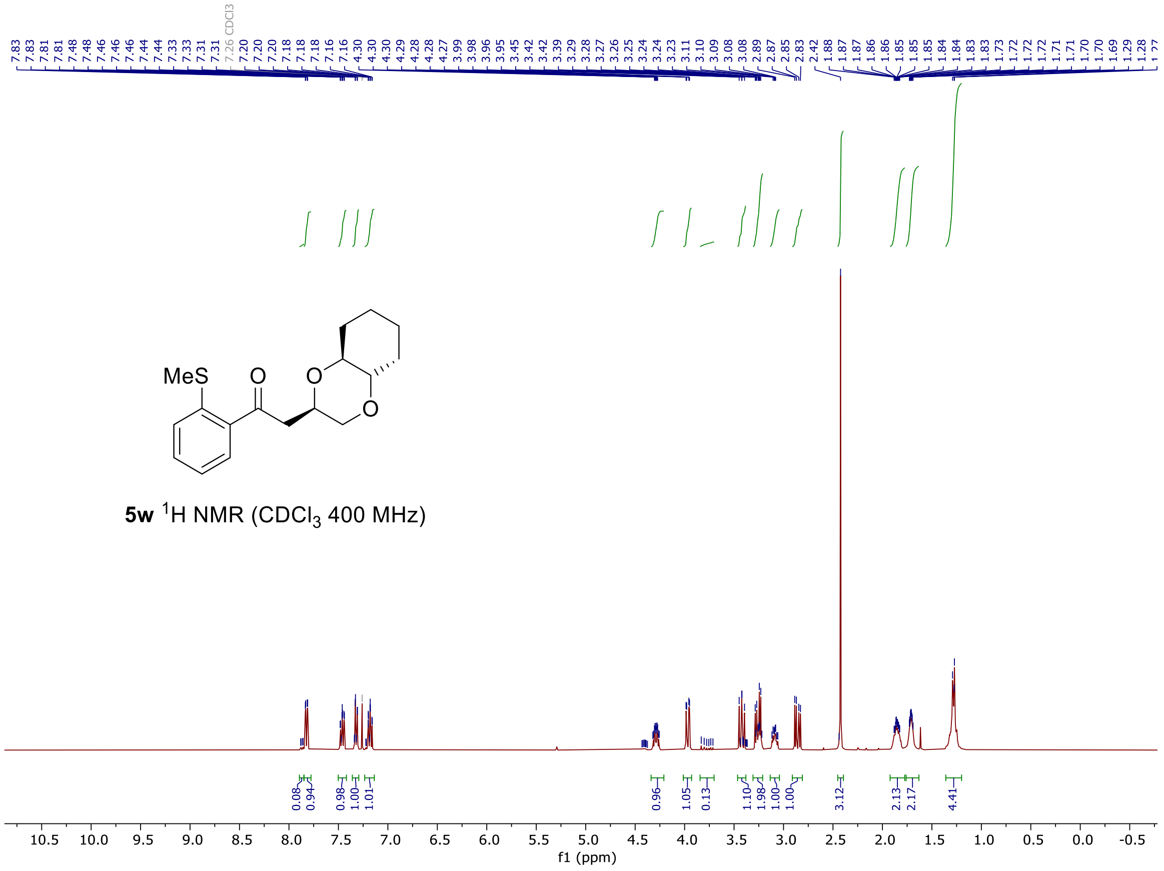

5w ^{1}H NMR ($CDCl_3$ 400 MHz)

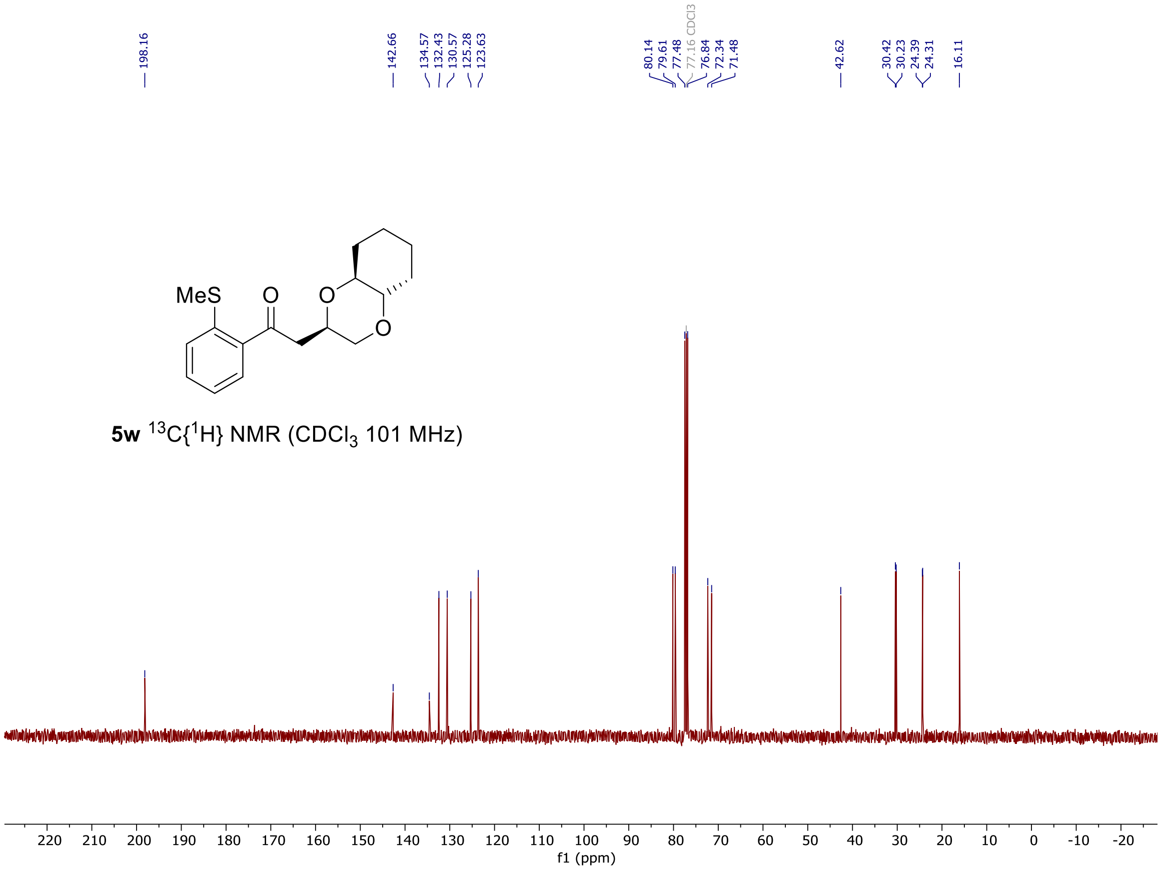

5w $^{13}C\{^{1}H\}$ NMR ($CDCl_3$ 101 MHz)

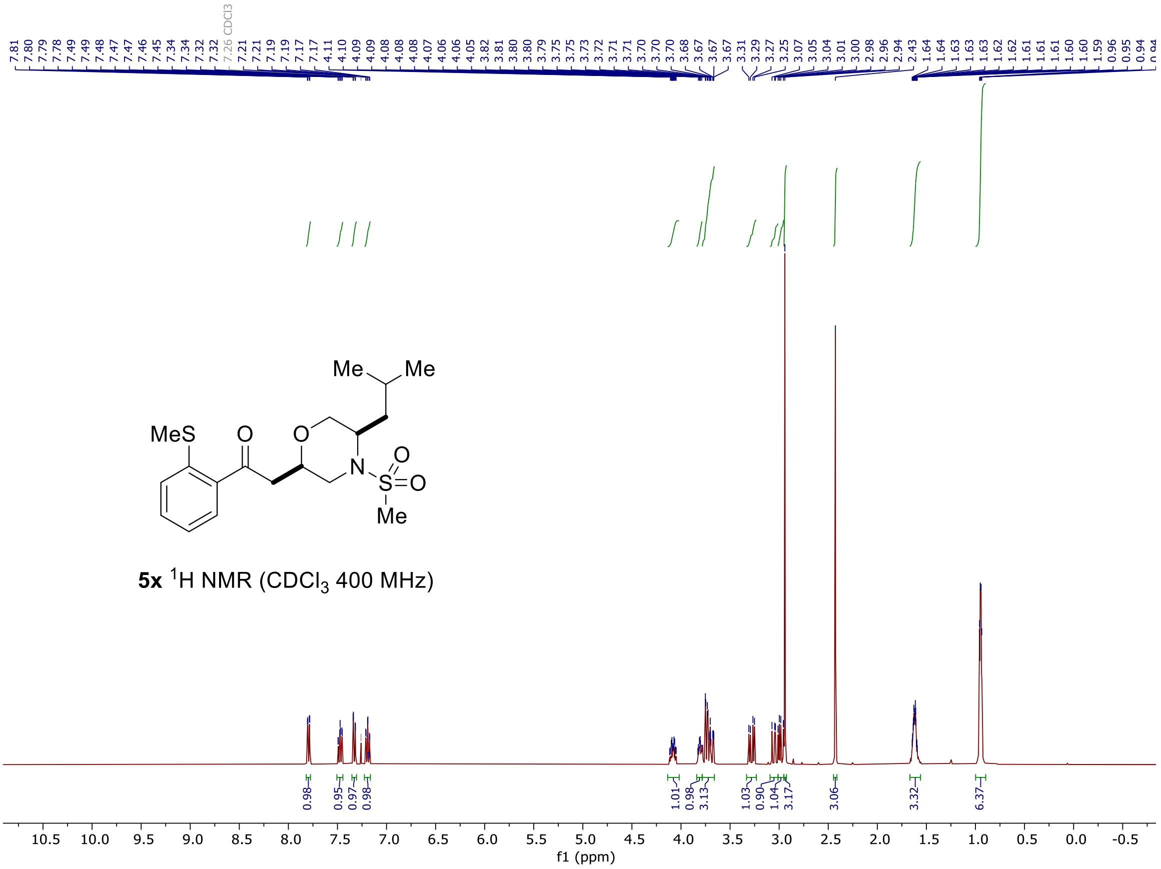

Me
Me
MeS
O
O
N
S
O
O
Me
5x ^{1}H NMR ($CDCl_3$ 400 MHz)
f1 (ppm)

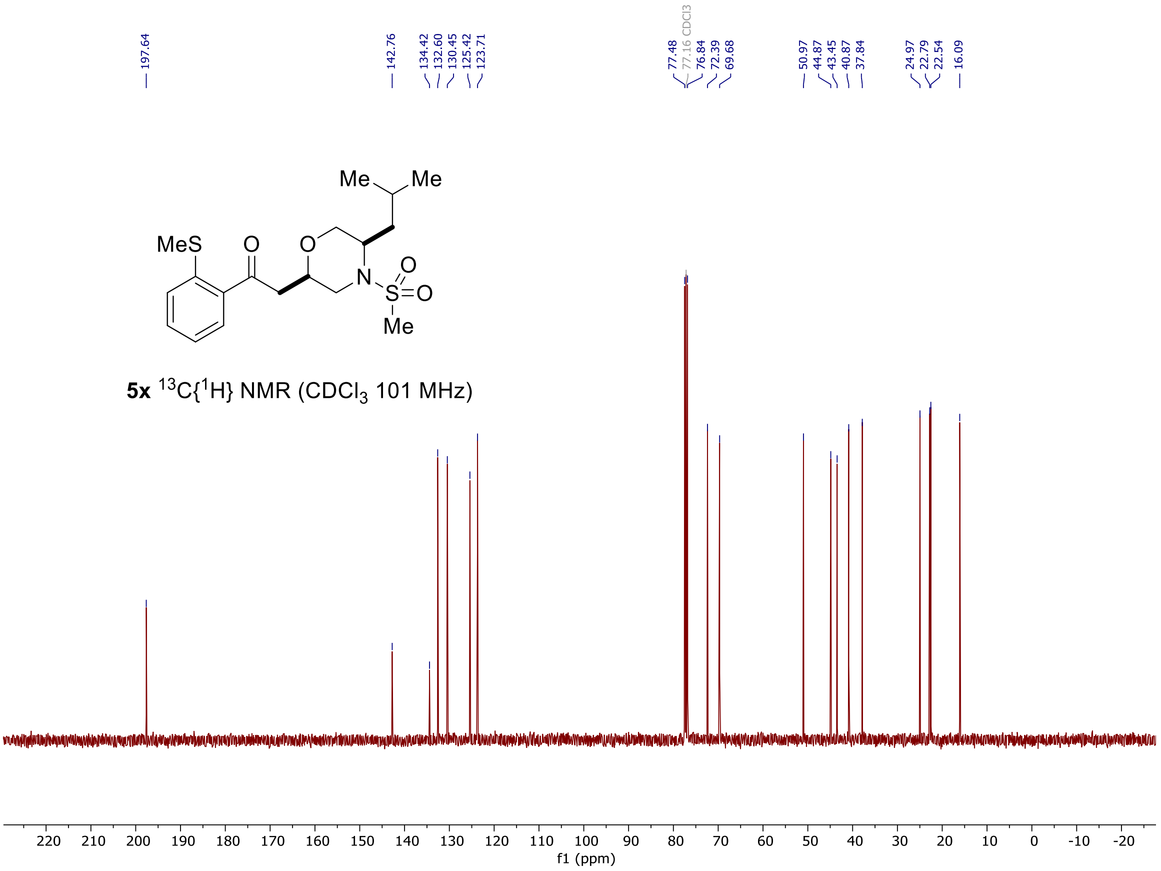

197.64
142.76
134.42
132.60
130.45
125.42
123.71
77.48
77.16 CDCl3
76.84
72.39
69.68
50.97
44.87
43.45
40.87
37.84
24.97
22.79
22.54
16.09
Me
Me
MeS
O
O
N
S
O
O
Me
5x ^{13}C{^{1}H} NMR ($CDCl_3$ 101 MHz)
f1 (ppm)

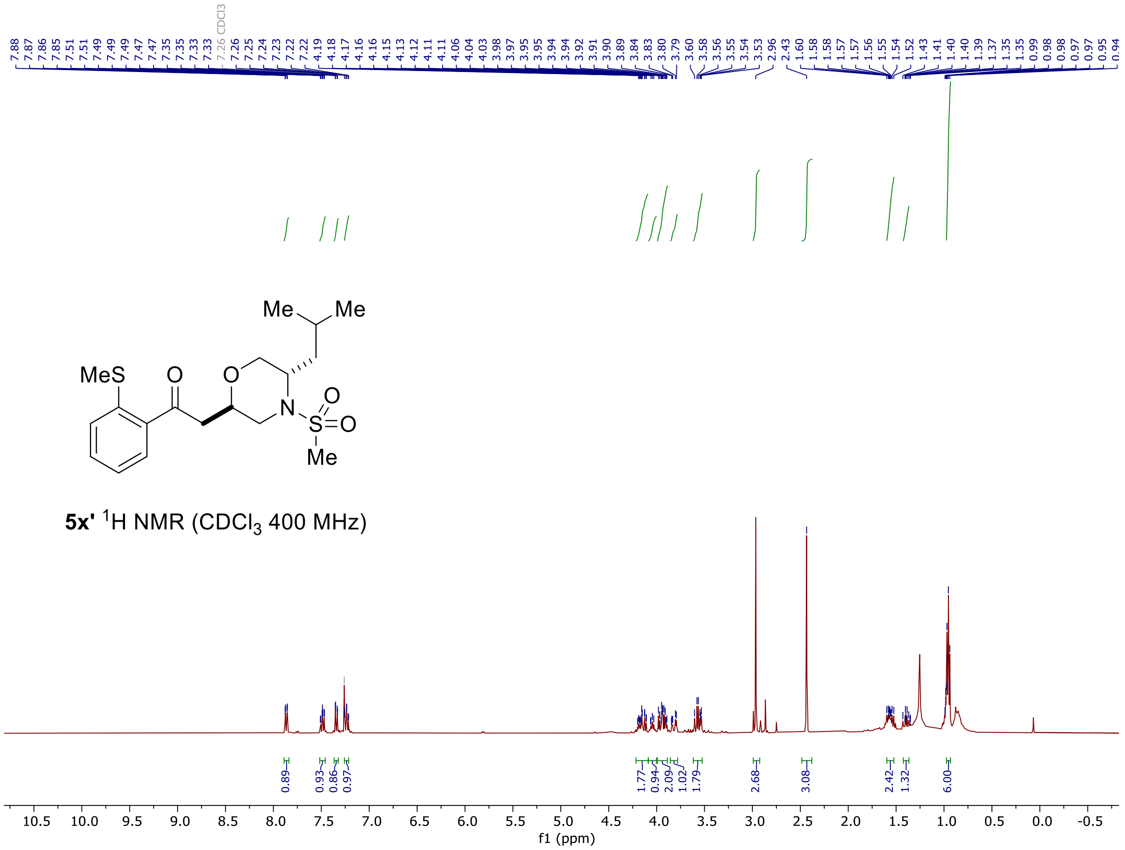

5x' ^{1}H NMR ($CDCl_3$ 400 MHz)

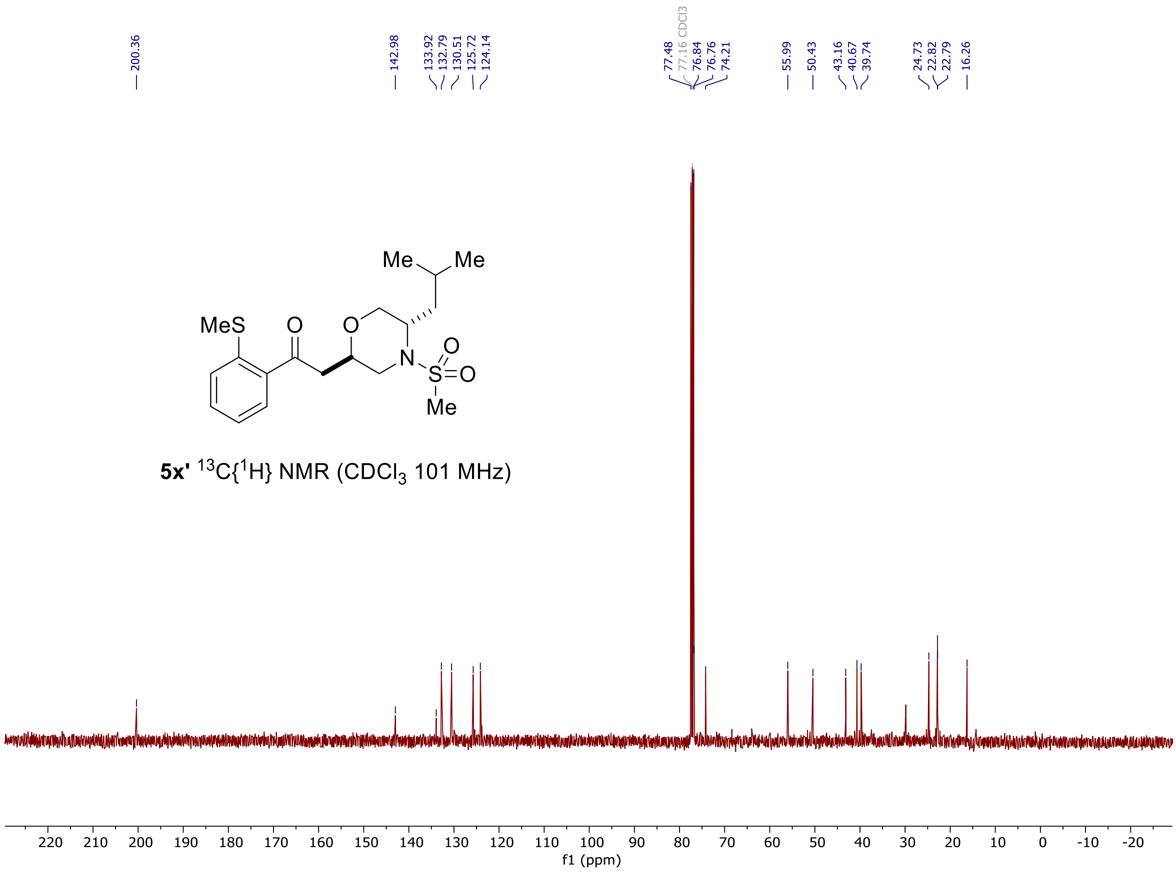

5x' $^{13}C\{^{1}H\}$ NMR ($CDCl_3$ 101 MHz)

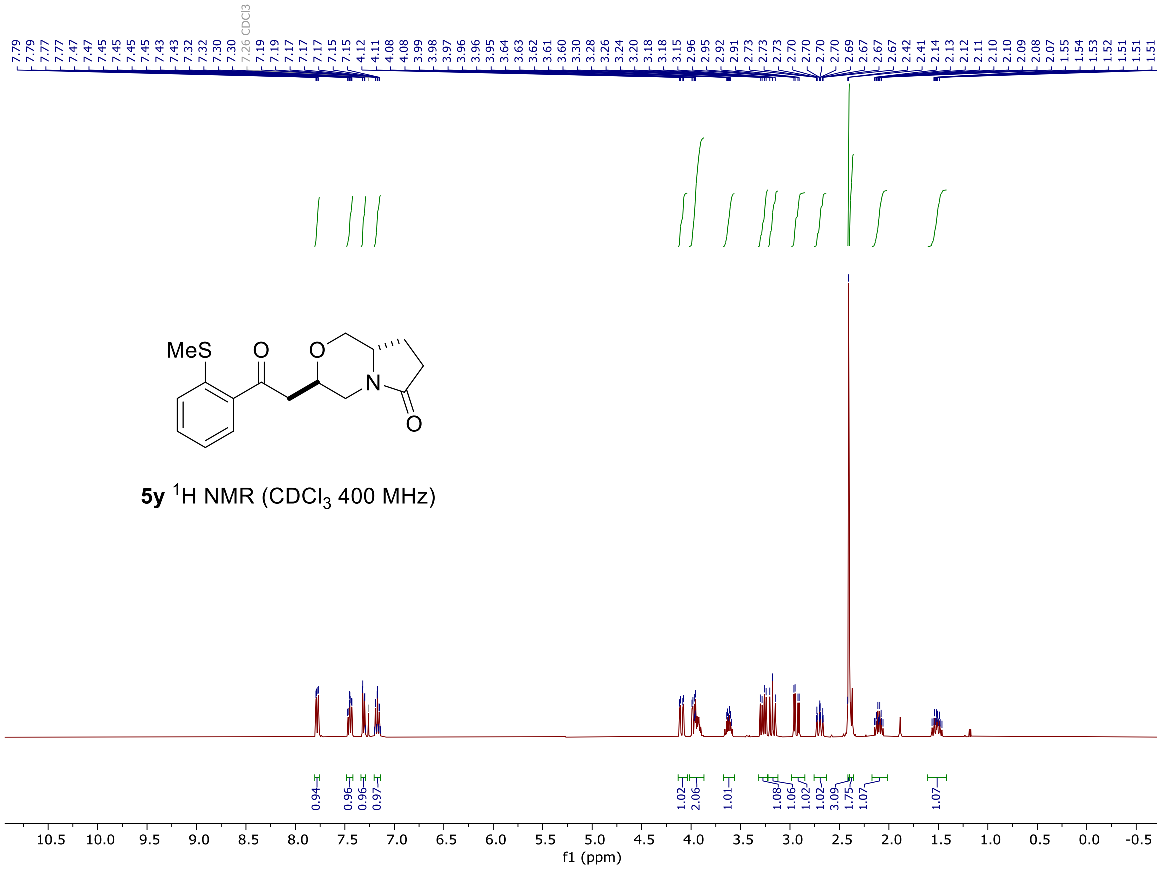

5y ¹H NMR ($CDCl_3$ 400 MHz)

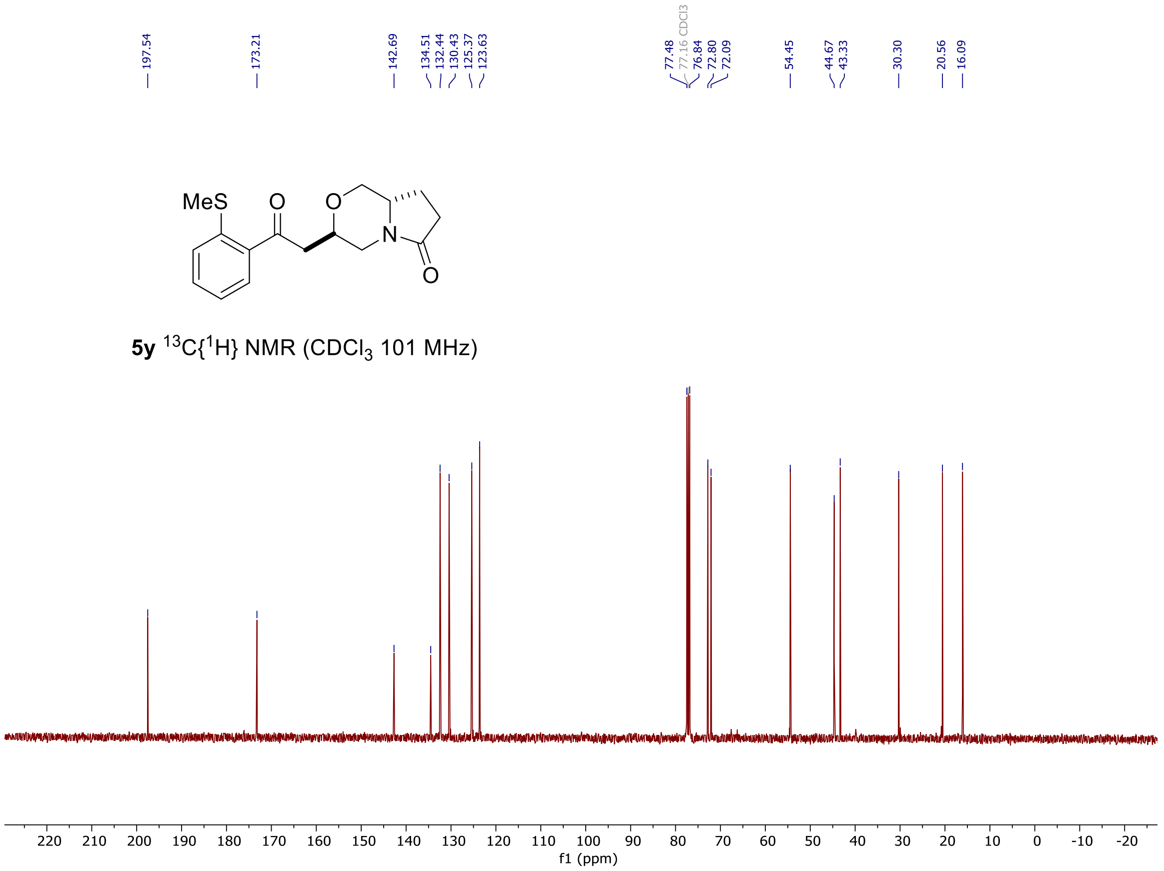

5y ¹³C{¹H} NMR ($CDCl_3$ 101 MHz)

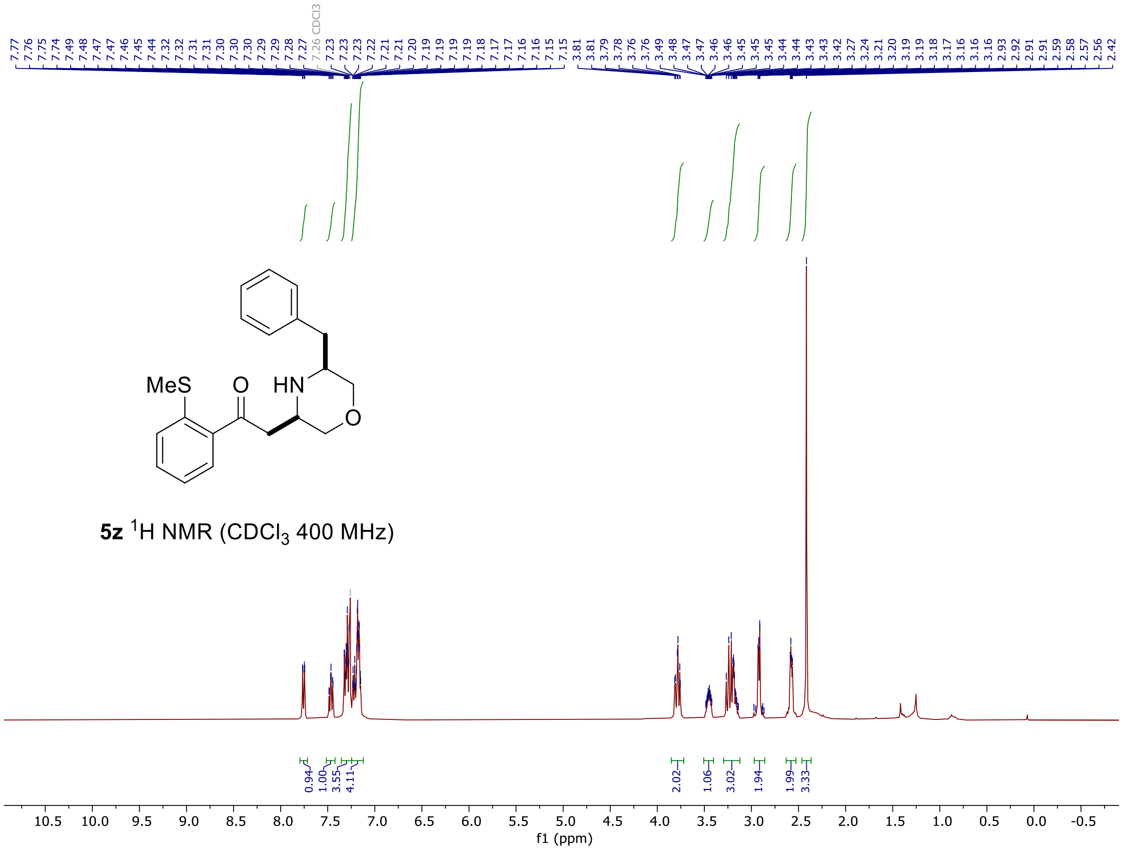
7.77 7.76 7.75 7.74 7.49 7.48 7.47 7.47 7.46 7.45 7.44 7.32 7.32 7.31 7.31 7.30 7.30 7.30 7.29 7.29 7.28 7.27 7.26 CDCl3 7.23 7.23 7.23 7.23 7.22 7.21 7.21 7.20 7.19 7.19 7.19 7.19 7.18 7.17 7.17 7.16 7.16 7.15 7.15
3.81 3.81 3.79 3.78 3.76 3.76 3.49 3.48 3.47 3.47 3.46 3.46 3.45 3.45 3.45 3.44 3.44 3.43 3.43 3.42 3.27 3.24 3.21 3.20 3.19 3.19 3.18 3.17 3.16 3.16 2.93 2.92 2.91 2.91 2.59 2.58 2.57 2.56 2.42
MeS
O
HN
O
5z 1H NMR (CDCl3 400 MHz)
0.94
1.00
3.55
4.11
2.02
1.06
3.02
1.94
1.99
3.33
10.5 10.0 9.5 9.0 8.5 8.0 7.5 7.0 6.5 6.0 5.5 5.0 4.5 4.0 3.5 3.0 2.5 2.0 1.5 1.0 0.5 0.0 -0.5
f1 (ppm)

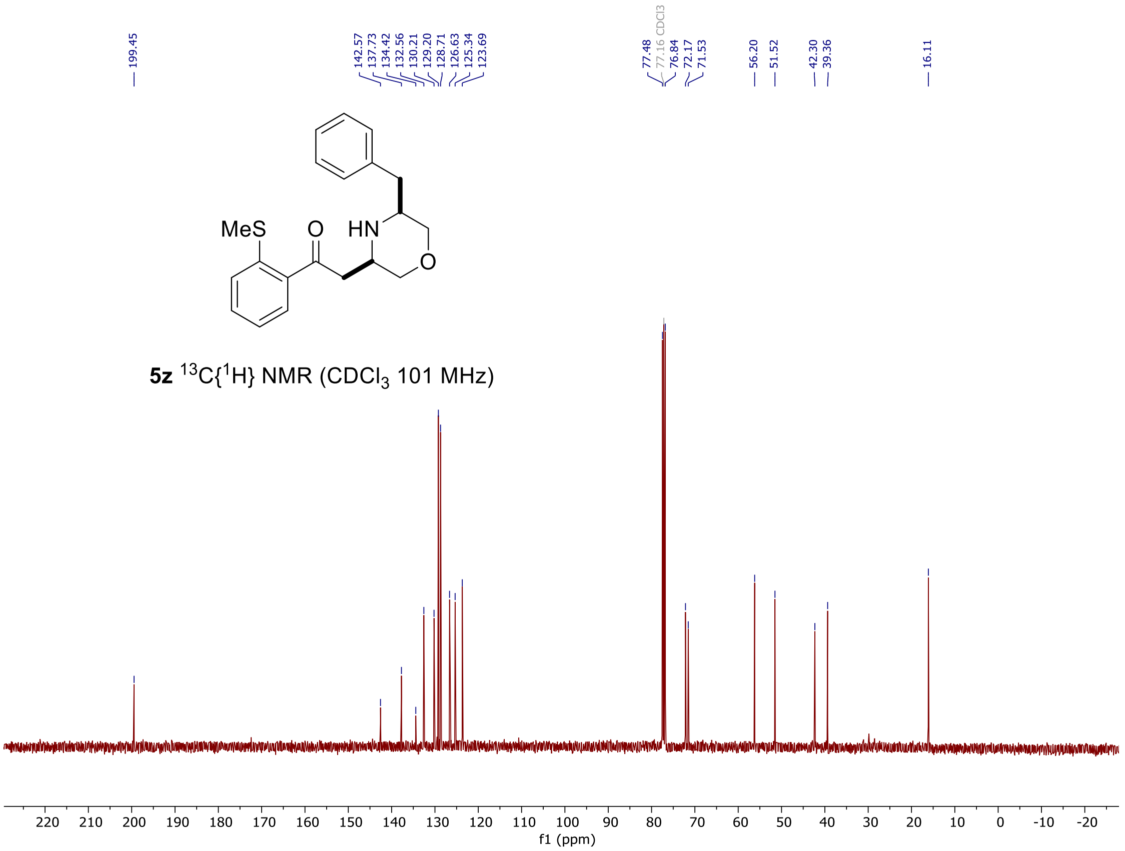
199.45
142.57 137.73 134.42 132.56 130.21 129.20 128.71 126.63 125.34 123.69
77.48 77.16 CDCl3 76.84 72.17 71.53
56.20 51.52 42.30 39.36 16.11
MeS
O
HN
O
5z 13C{1H} NMR (CDCl3 101 MHz)
220 210 200 190 180 170 160 150 140 130 120 110 100 90 80 70 60 50 40 30 20 10 0 -10 -20
f1 (ppm)

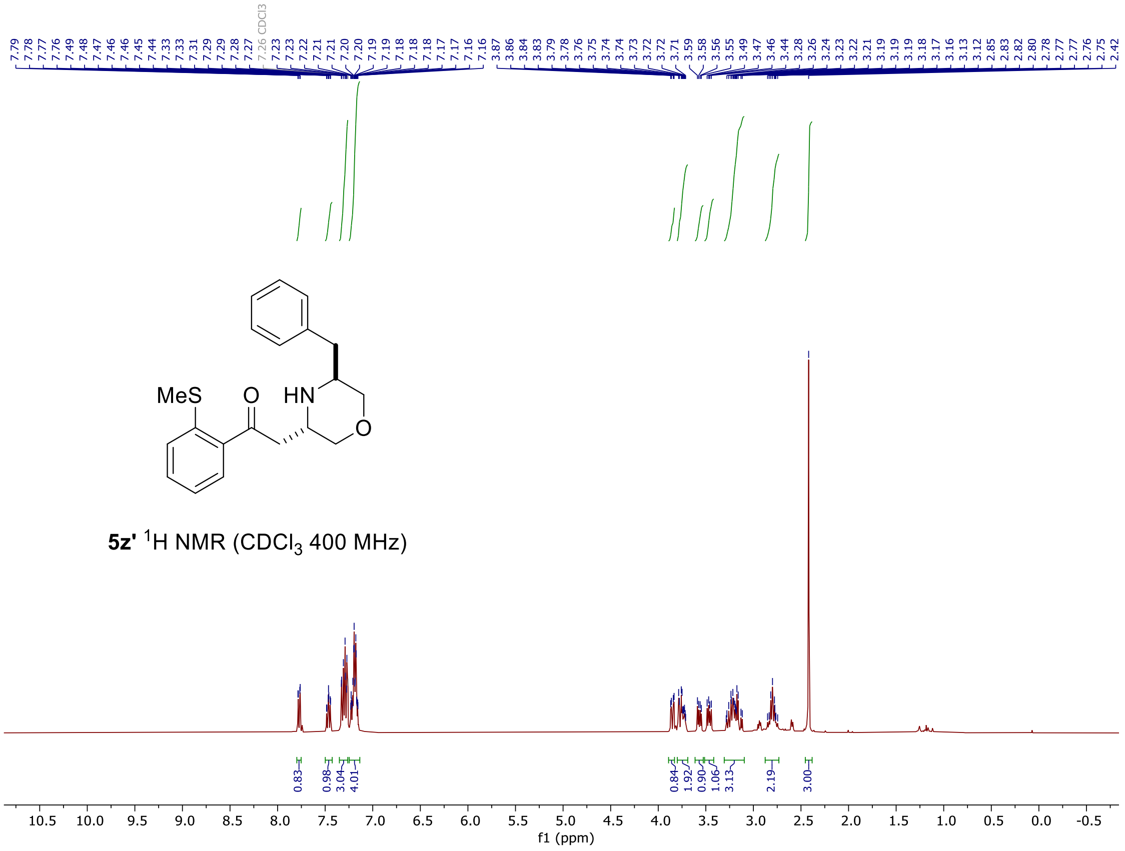

5z' ^{1}H NMR ($CDCl_3$ 400 MHz)

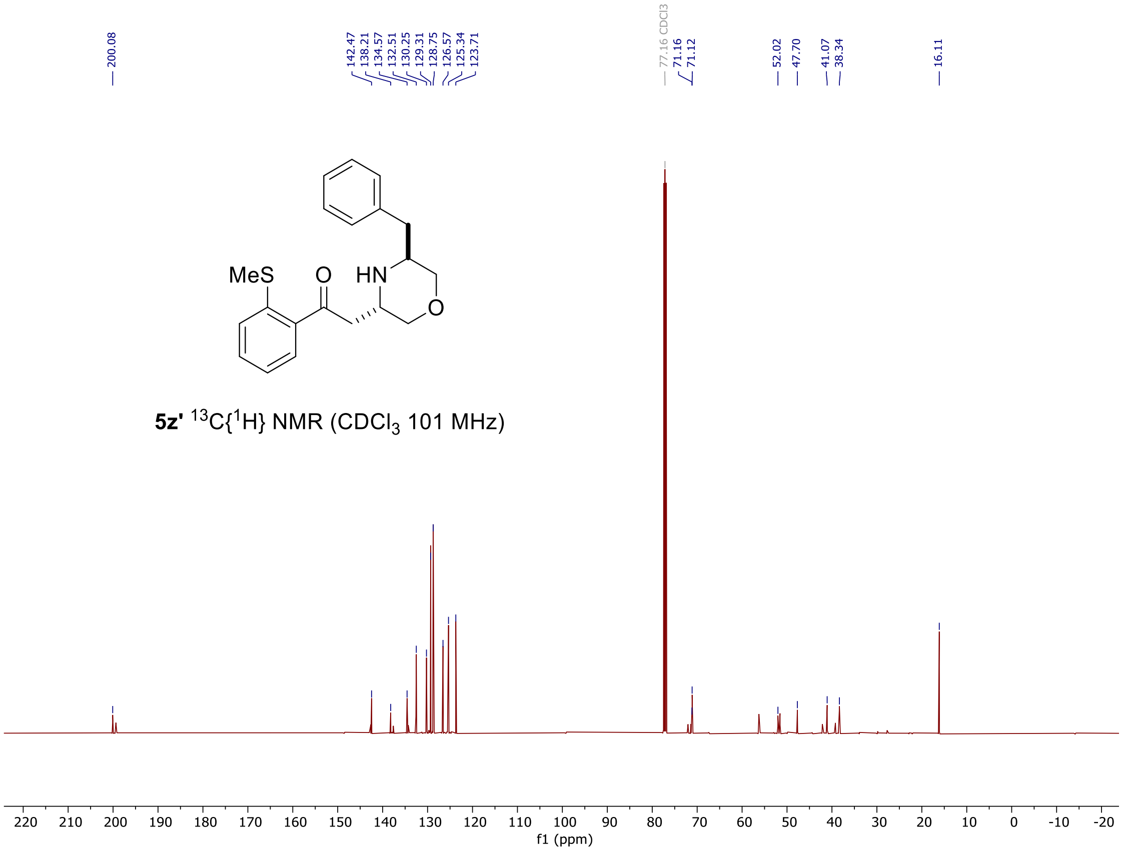

5z' $^{13}C\{^{1}H\}$ NMR ($CDCl_3$ 101 MHz)

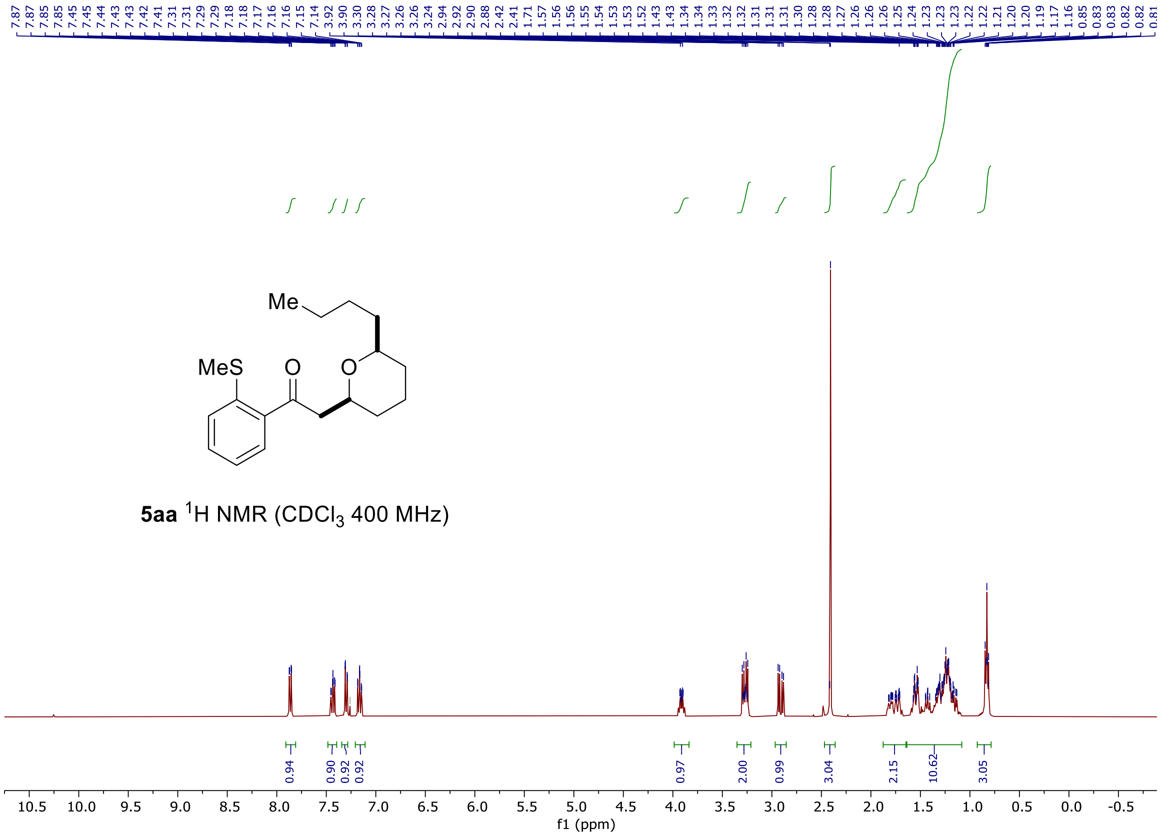
Me
MeS
O
O
5aa ¹H NMR (CDCl3 400 MHz)
f1 (ppm)

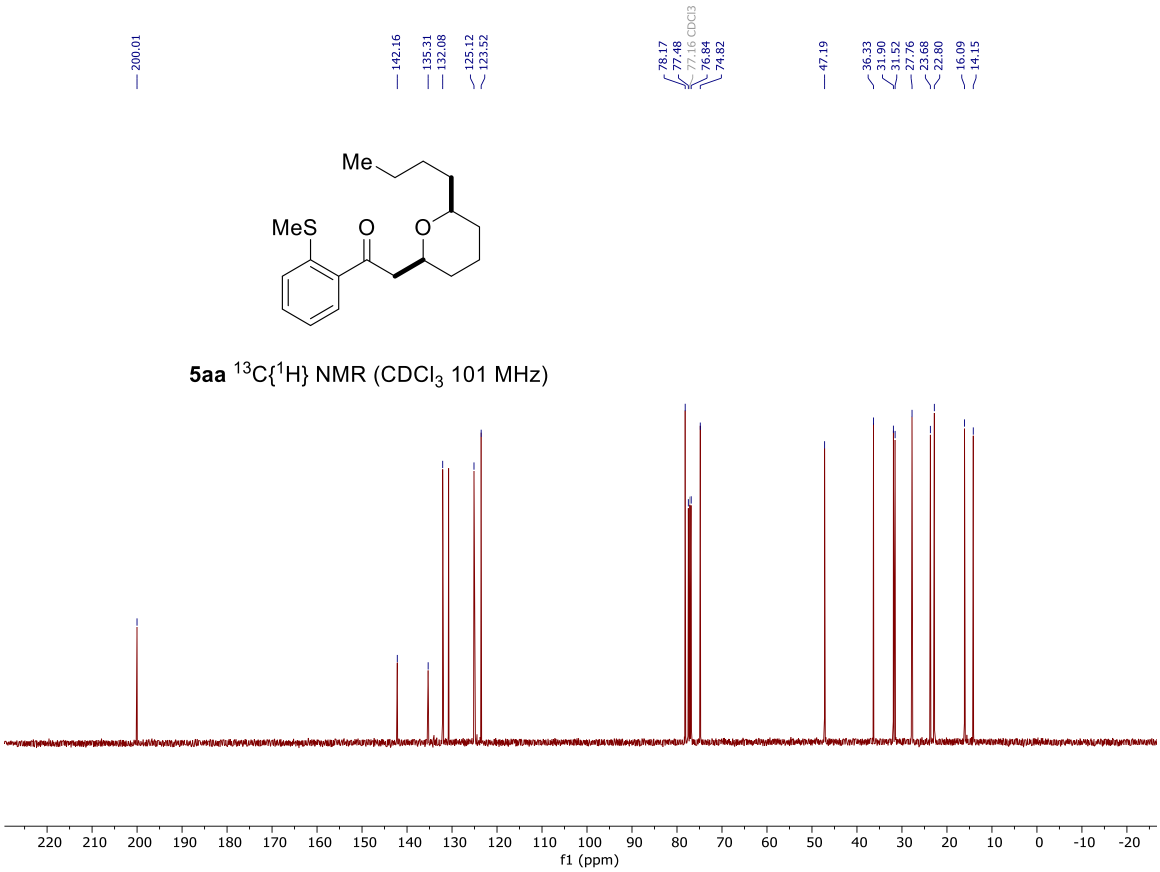
Me
MeS
O
O
5aa ¹³C{¹H} NMR (CDCl3 101 MHz)
f1 (ppm)

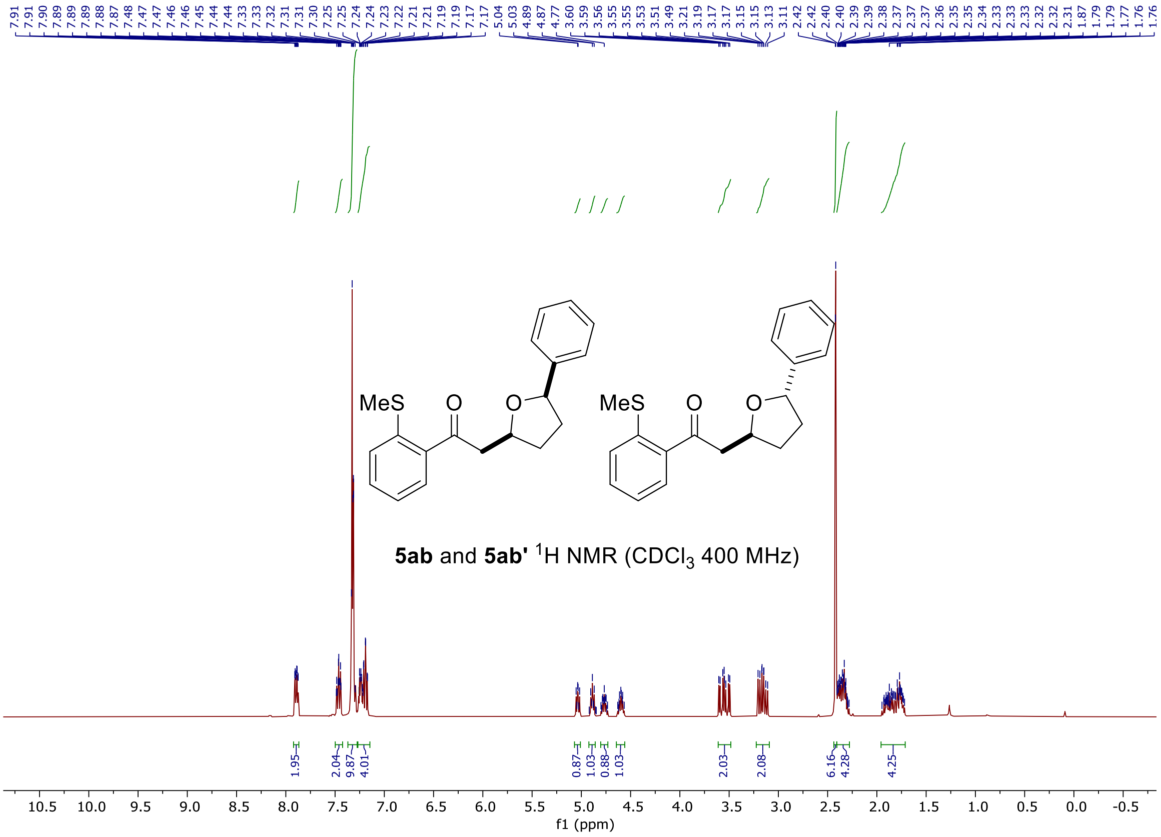
7.91 7.91 7.90 7.89 7.89 7.88 7.87 7.48 7.47 7.46 7.45 7.44 7.44 7.33 7.32 7.31 7.31 7.30 7.25 7.25 7.24 7.24 7.23 7.22 7.21 7.21 7.19 7.19 7.17 7.17 5.04 5.03 4.89 4.87 4.77 3.60 3.59 3.56 3.55 3.55 3.53 3.51 3.49 3.21 3.19 3.17 3.17 3.15 3.15 3.13 3.13 3.11 2.42 2.42 2.40 2.40 2.39 2.39 2.38 2.37 2.37 2.36 2.35 2.35 2.34 2.33 2.33 2.33 2.32 2.32 2.31 1.87 1.79 1.77 1.76 1.76
MeS
O
O
MeS
O
O
5ab and **5ab'** ^{1}H NMR (CDCl$_3$ 400 MHz)
1.95 2.04 9.87 4.01 0.87 1.03 0.88 1.03 2.03 2.08 6.16 4.28 4.25
10.5 10.0 9.5 9.0 8.5 8.0 7.5 7.0 6.5 6.0 5.5 5.0 4.5 4.0 3.5 3.0 2.5 2.0 1.5 1.0 0.5 0.0 -0.5
f1 (ppm)

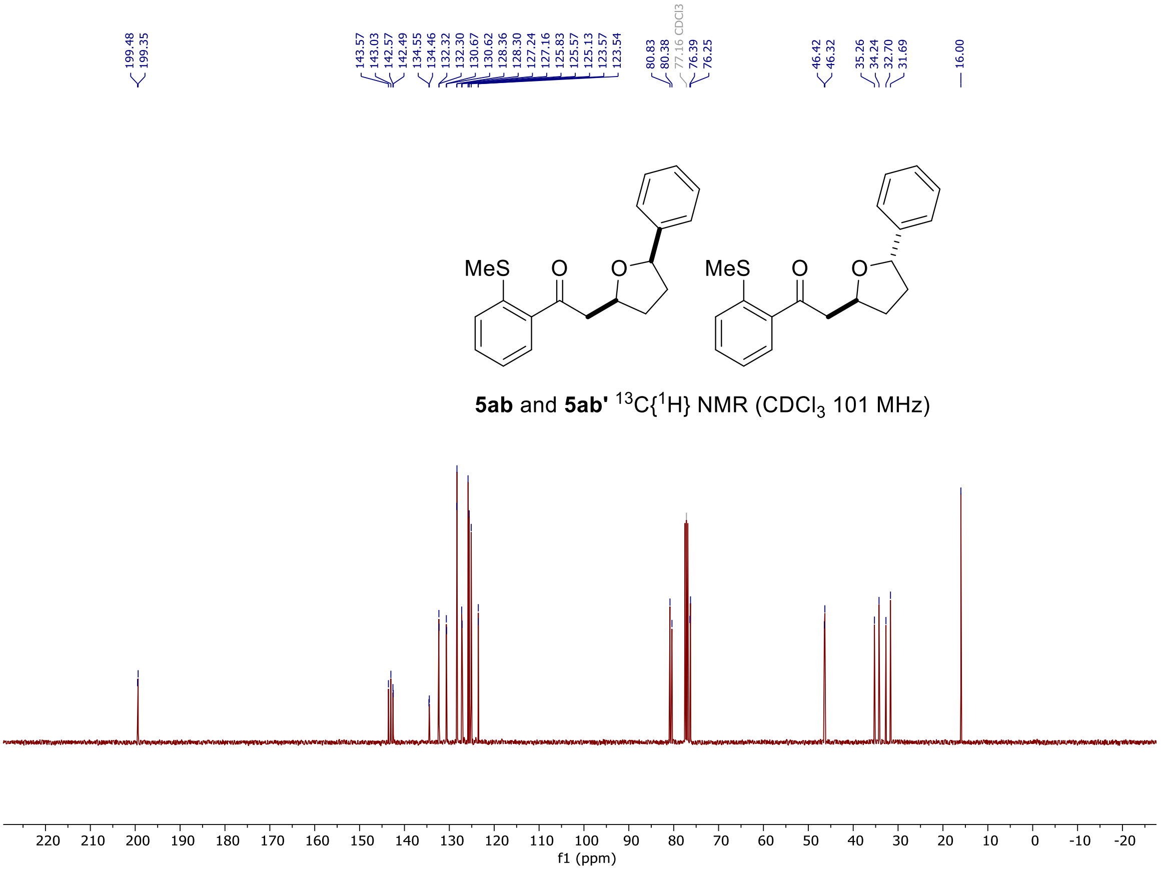
199.48 199.35 143.57 143.03 142.57 142.49 134.55 134.46 132.32 132.30 130.67 130.62 128.36 128.30 127.24 127.16 125.83 125.57 125.13 123.57 123.54 80.83 80.38 77.16 CDCl3 76.39 76.25 46.42 46.32 35.26 34.24 32.70 31.69 16.00
MeS
O
O
MeS
O
O
5ab and **5ab'** ^{13}C{^{1}H} NMR (CDCl$_3$ 101 MHz)
220 210 200 190 180 170 160 150 140 130 120 110 100 90 80 70 60 50 40 30 20 10 0 -10 -20
f1 (ppm)

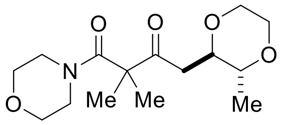

5ac ^{1}H NMR ($CDCl_3$ 500 MHz)

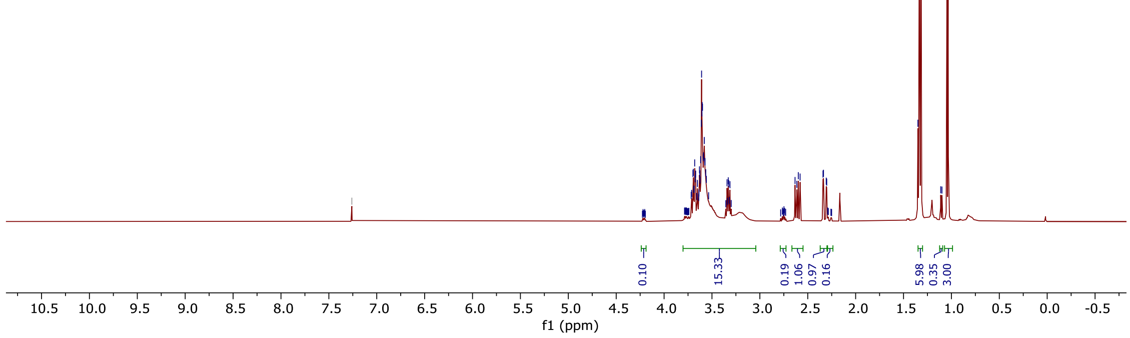

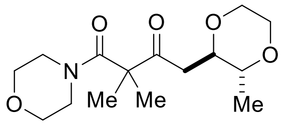

5ac ^{13}C{^{1}H} NMR ($CDCl_3$ 101 MHz)

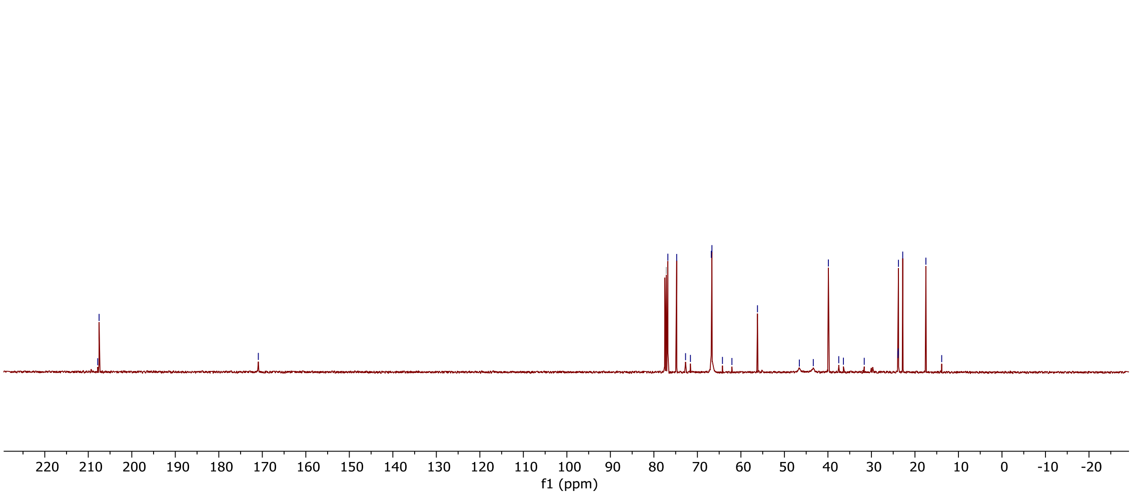

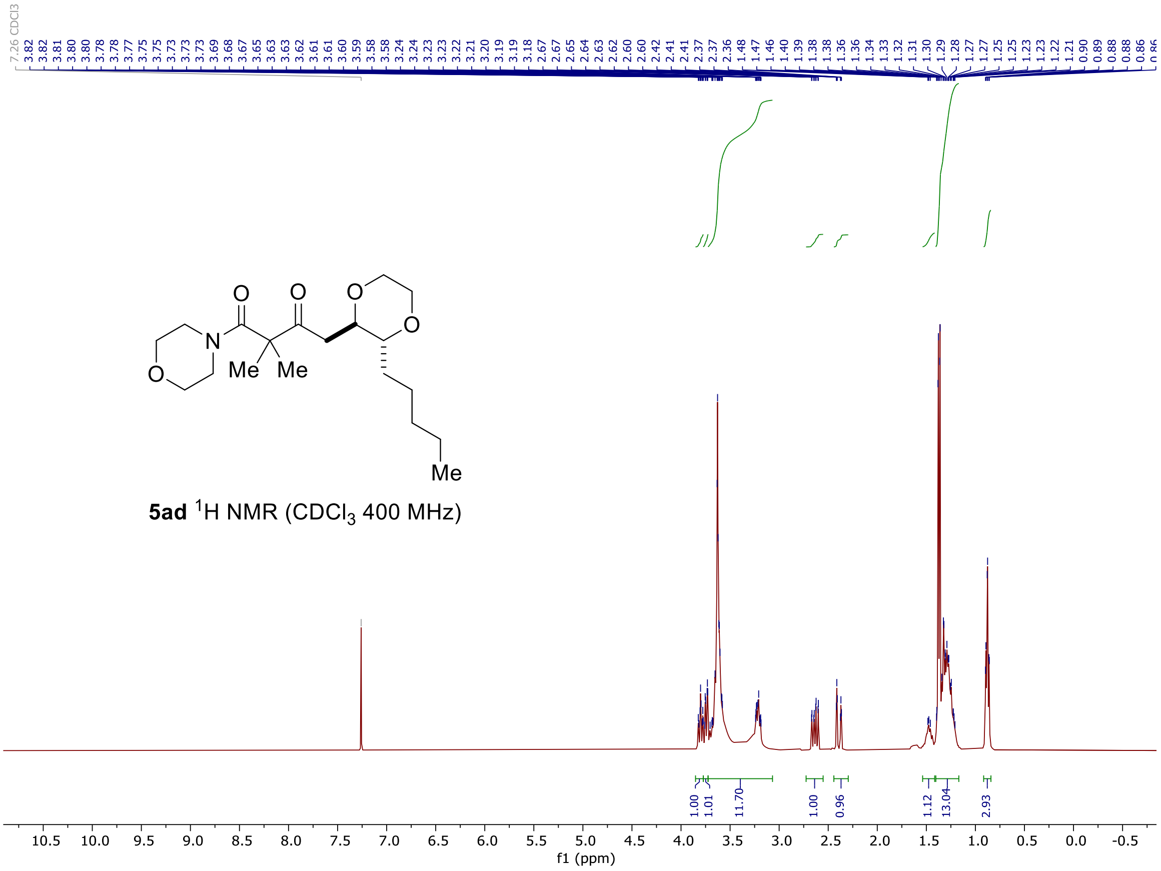
7.26 CDCl3
3.82
3.82
3.81
3.80
3.80
3.78
3.78
3.77
3.75
3.75
3.73
3.73
3.73
3.69
3.68
3.67
3.65
3.63
3.63
3.62
3.61
3.61
3.60
3.59
3.58
3.58
3.24
3.24
3.23
3.23
3.22
3.21
3.20
3.19
3.19
3.18
2.67
2.67
2.65
2.64
2.63
2.62
2.60
2.60
2.42
2.41
2.41
2.37
2.37
2.36
1.48
1.47
1.46
1.40
1.39
1.38
1.38
1.36
1.36
1.34
1.33
1.32
1.31
1.30
1.29
1.28
1.27
1.27
1.25
1.25
1.23
1.23
1.22
1.21
0.90
0.89
0.88
0.88
0.86
O
O
O
O
N
O
Me
Me
Me
5ad 1H NMR (CDCl3 400 MHz)
1.00
1.01
11.70
1.00
0.96
1.12
13.04
2.93
f1 (ppm)

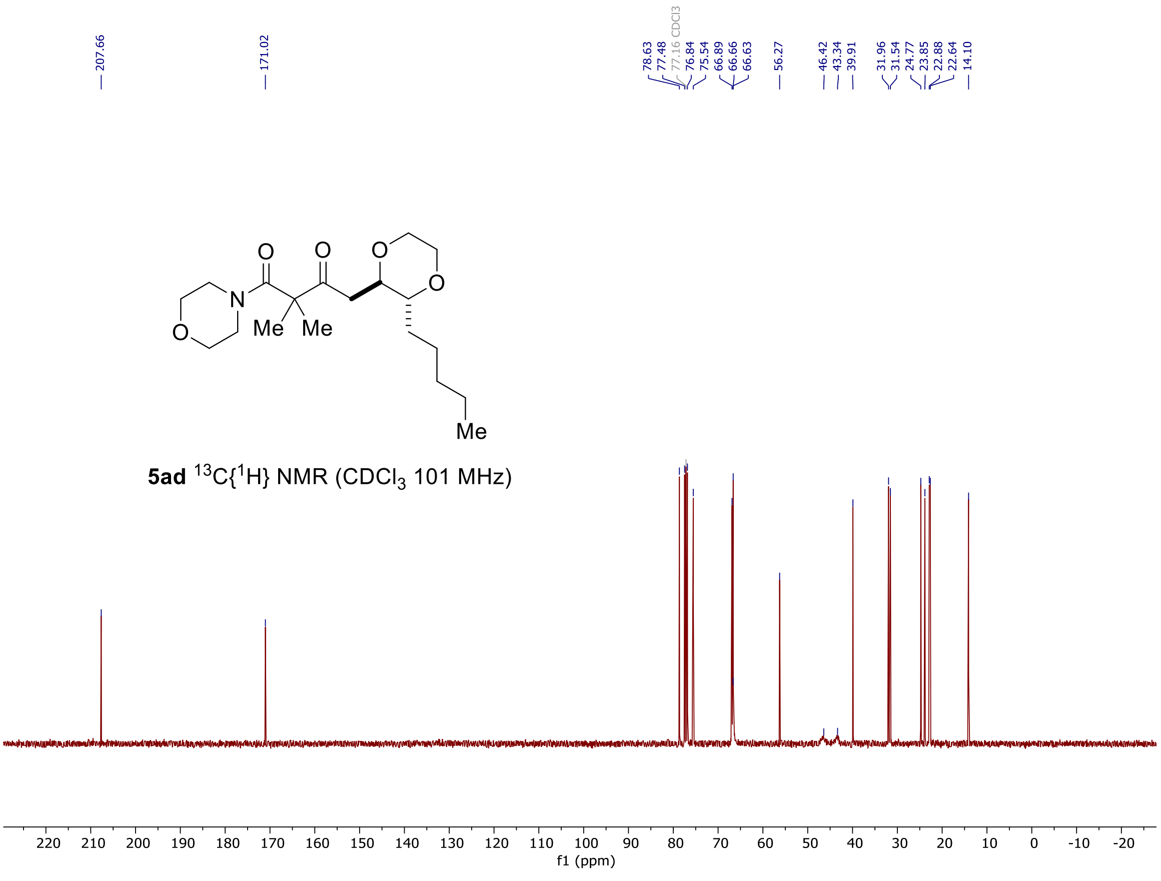
207.66
171.02
78.63
77.48
77.16 CDCl3
76.84
75.54
66.89
66.66
66.63
56.27
46.42
43.34
39.91
31.96
31.54
24.77
23.85
22.88
22.64
14.10
O
O
O
O
N
O
Me
Me
Me
5ad 13C{1H} NMR (CDCl3 101 MHz)
f1 (ppm)

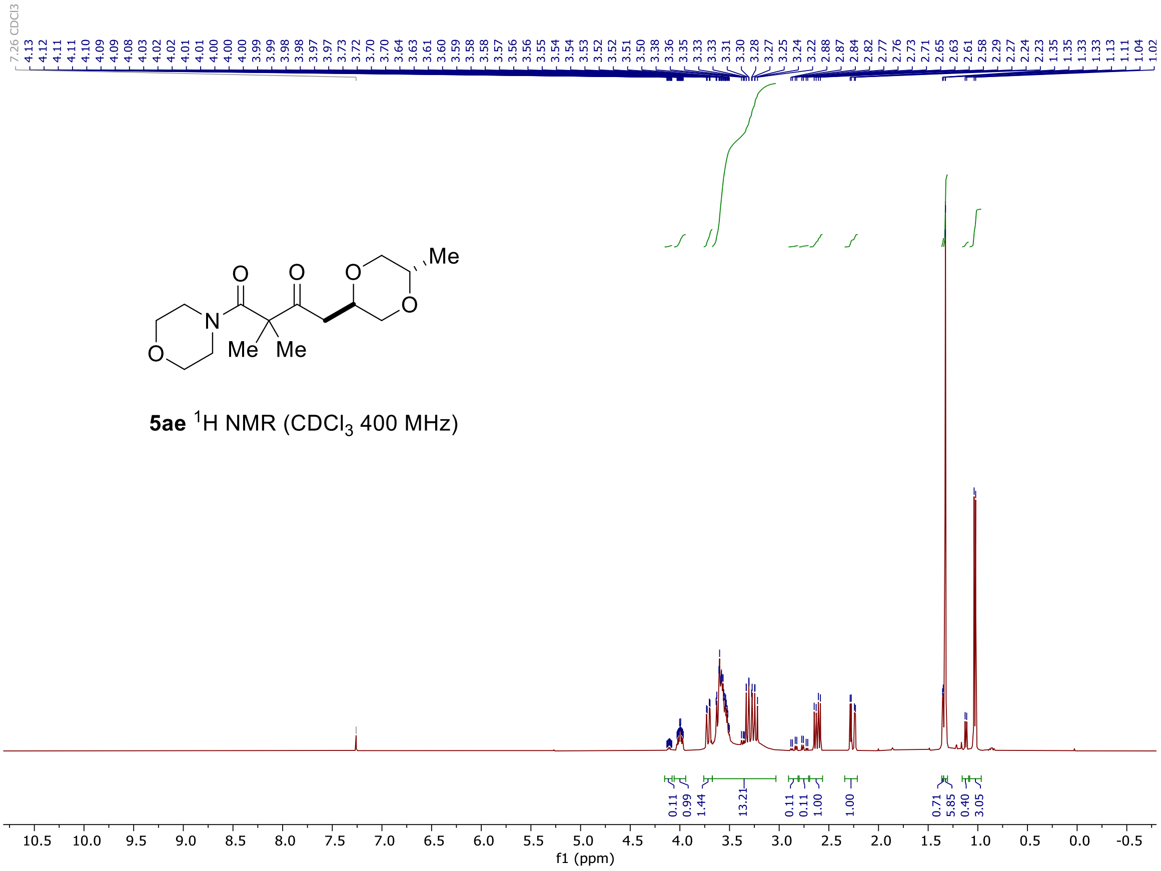

5ae ^{1}H NMR ($CDCl_3$ 400 MHz)

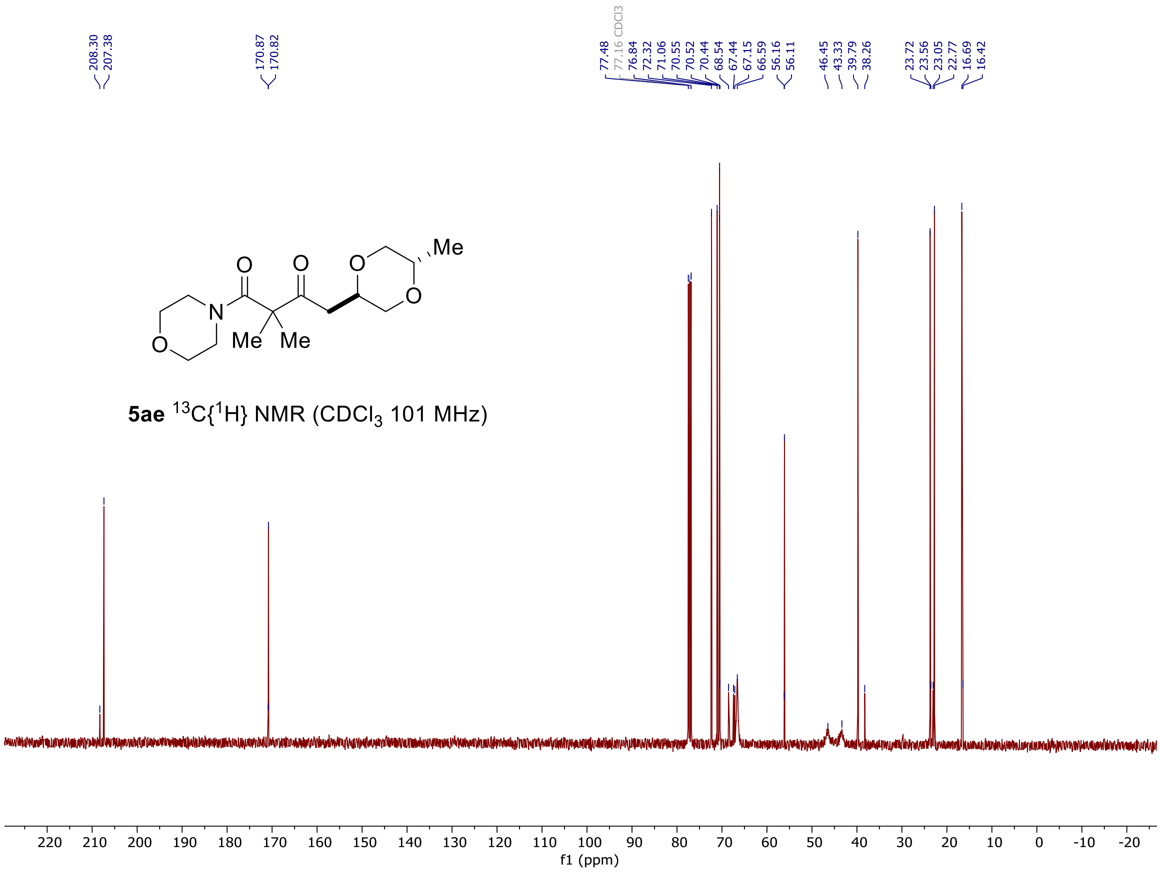

5ae $^{13}C\{^{1}H\}$ NMR ($CDCl_3$ 101 MHz)

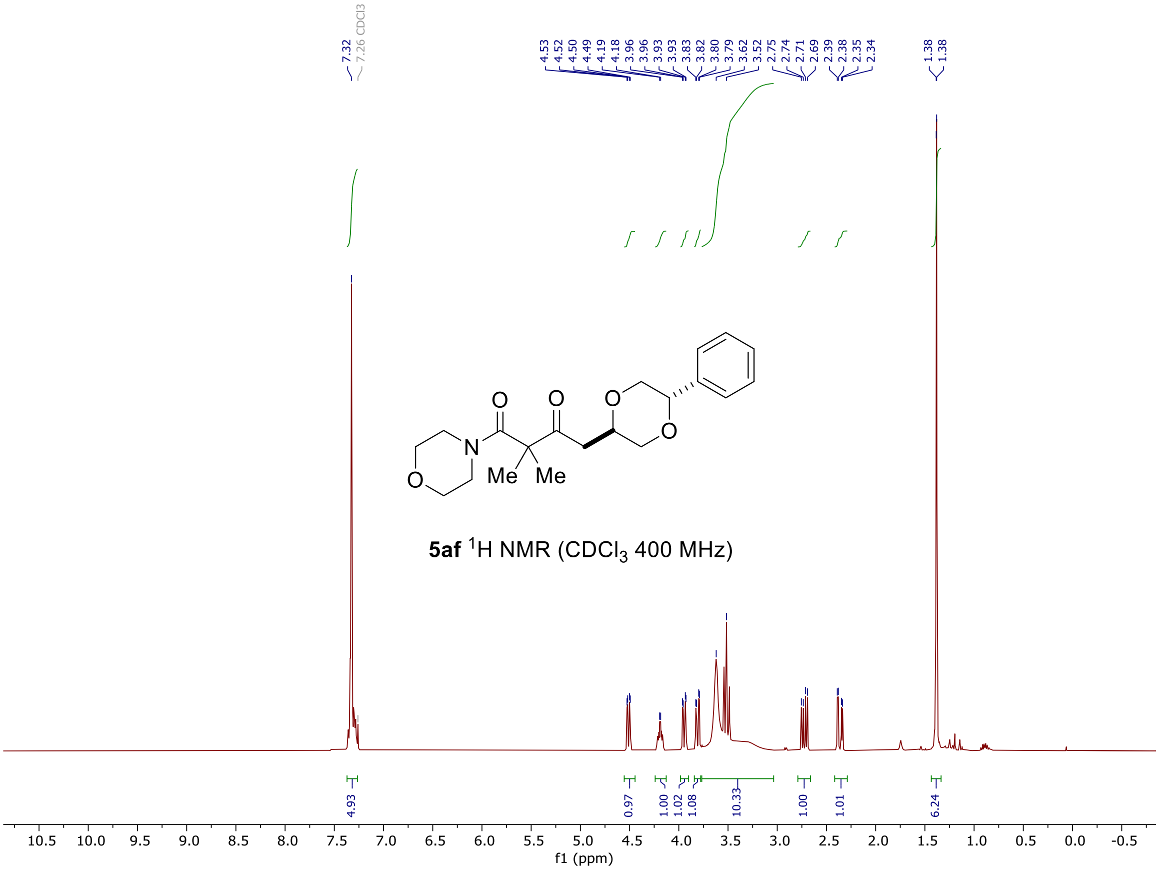
7.32
7.26 CDCl3
4.53 4.52 4.50 4.49 4.19 4.18 3.96 3.96 3.93 3.93 3.83 3.82 3.80 3.79 3.62 3.52 2.75 2.74 2.71 2.69 2.39 2.38 2.35 2.34
1.38 1.38
O O O O N O Me Me
5af ¹H NMR (CDCl3 400 MHz)
4.93 0.97 1.00 1.02 1.08 10.33 1.00 1.01 6.24
f1 (ppm)

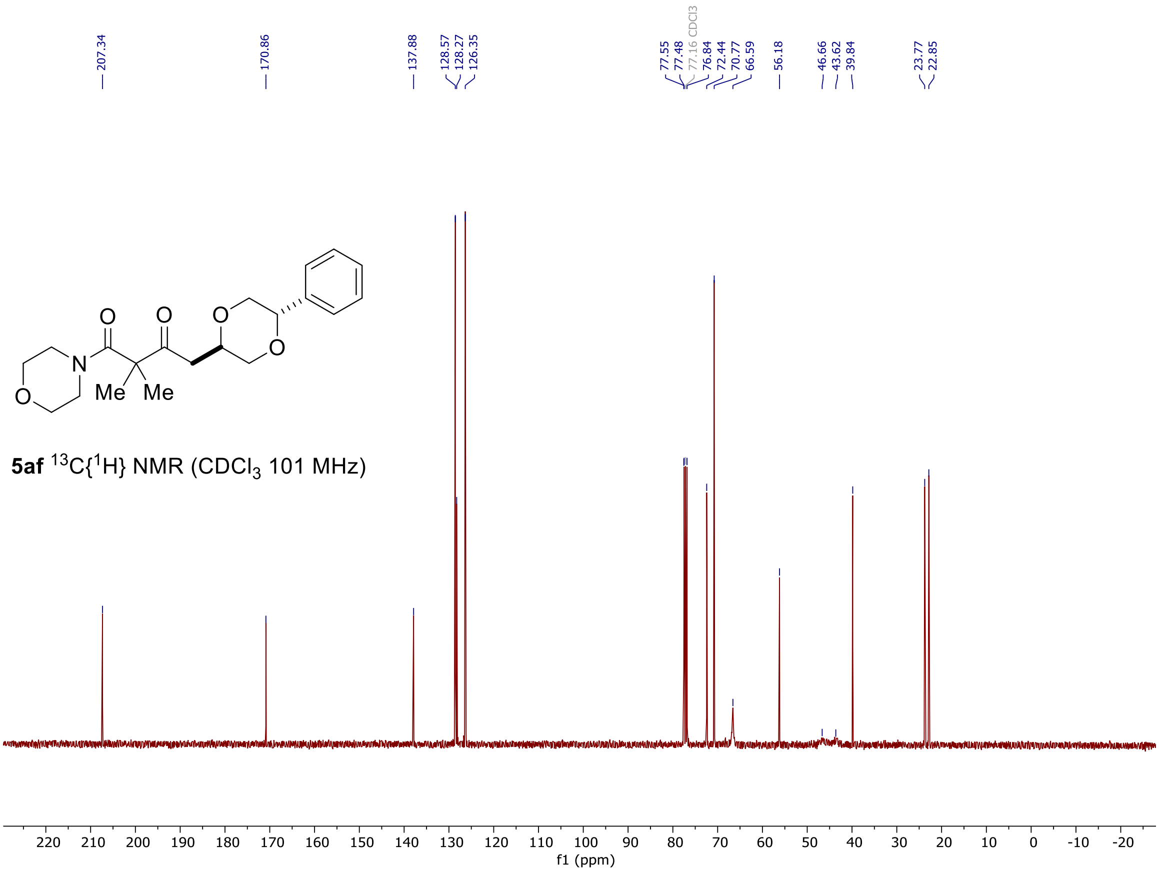
207.34
170.86
137.88
128.57 128.27 126.35
77.55 77.48 77.16 CDCl3 76.84 72.44 70.77 66.59
56.18
46.66 43.62 39.84
23.77 22.85
O O O O N O Me Me
5af ¹³C{¹H} NMR (CDCl3 101 MHz)
f1 (ppm)

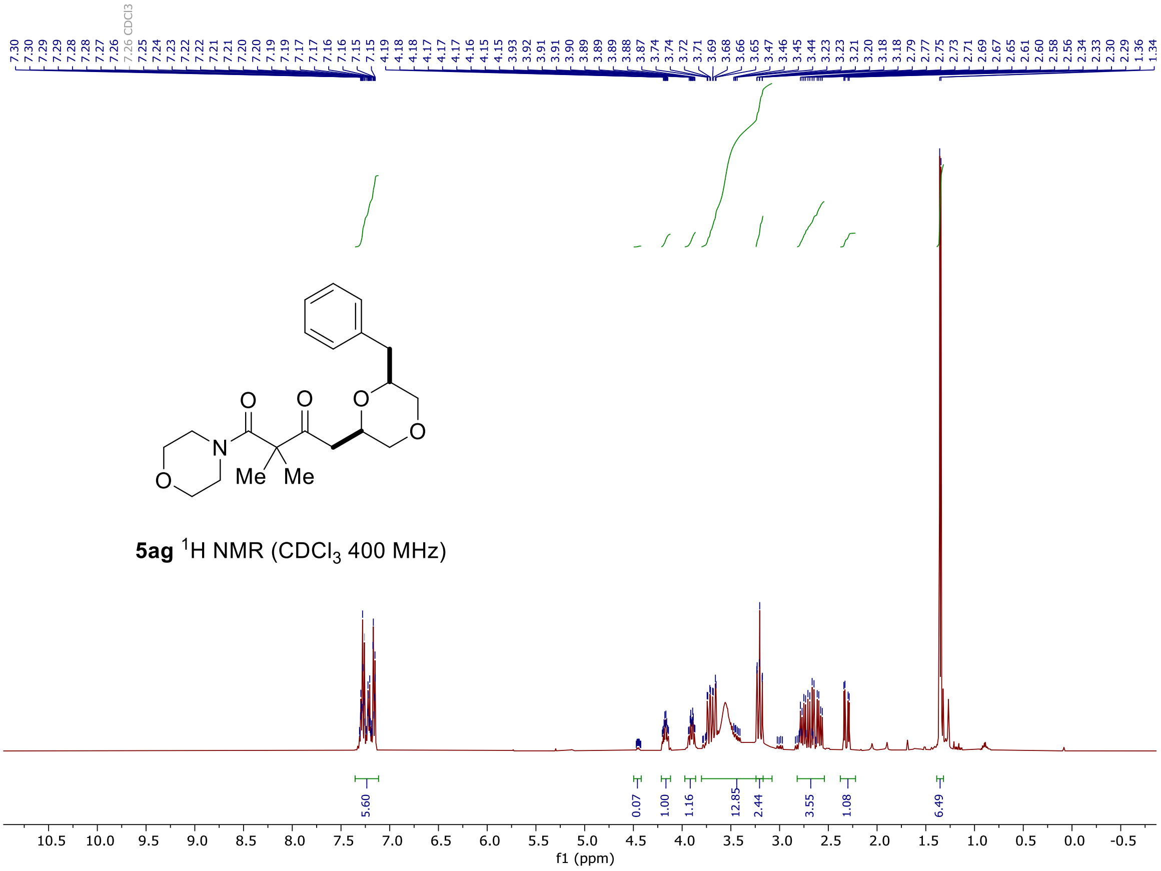

5ag ^{1}H NMR ($CDCl_3$ 400 MHz)

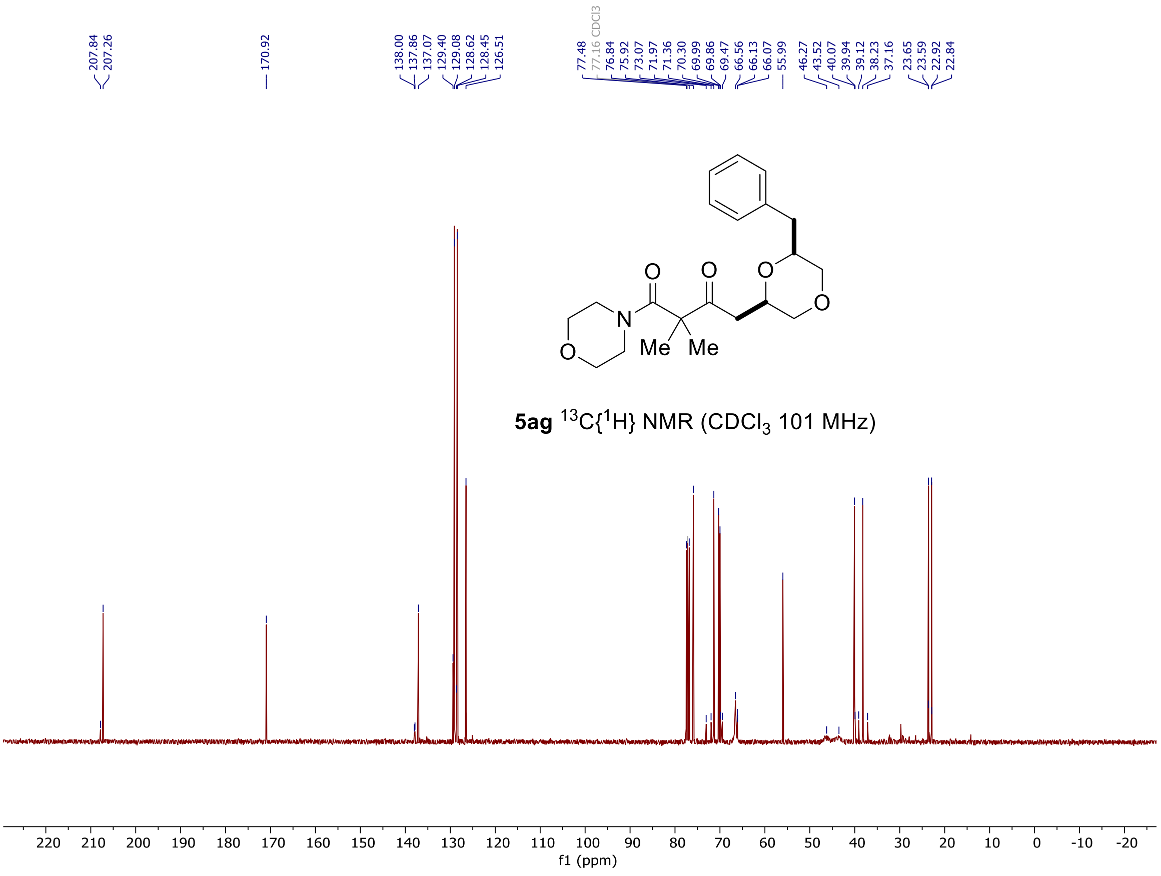

5ag $^{13}C\{^{1}H\}$ NMR ($CDCl_3$ 101 MHz)

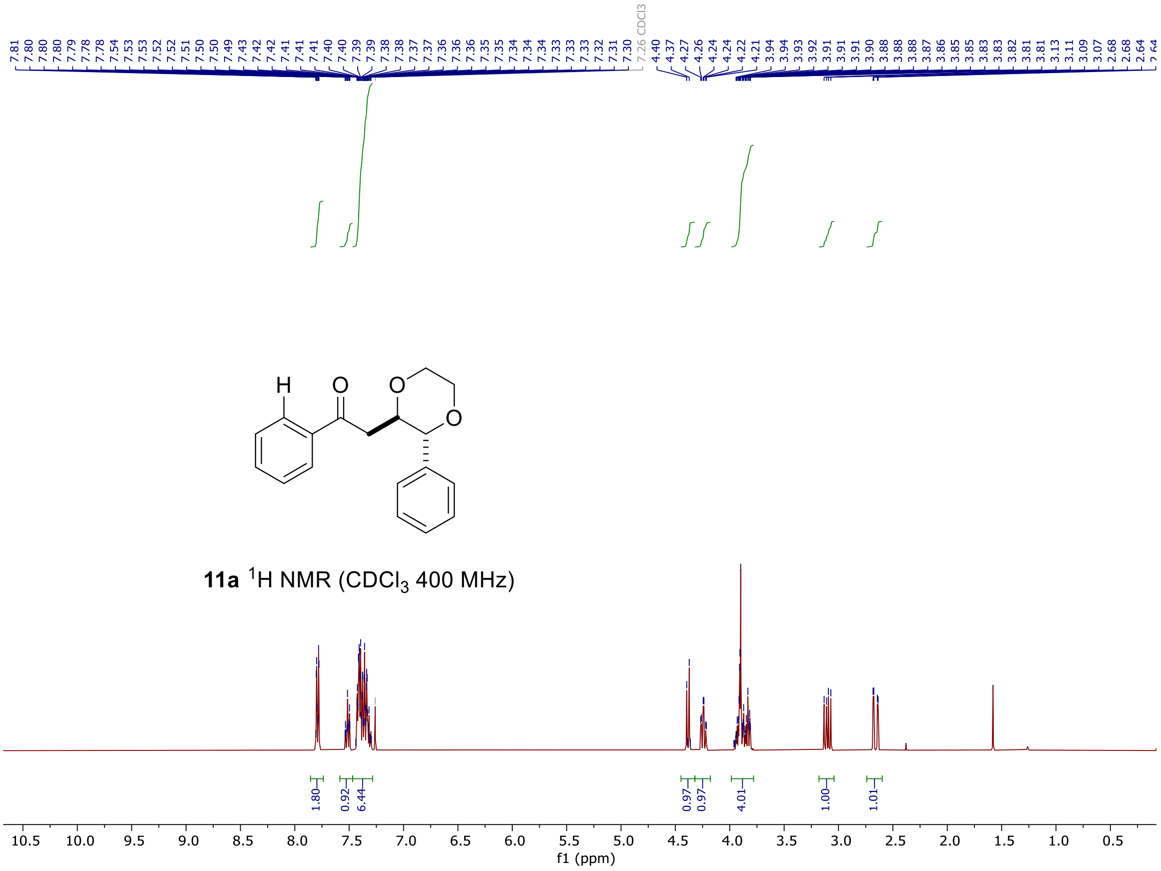

11a ^{1}H NMR (CDCl$_3$ 400 MHz)

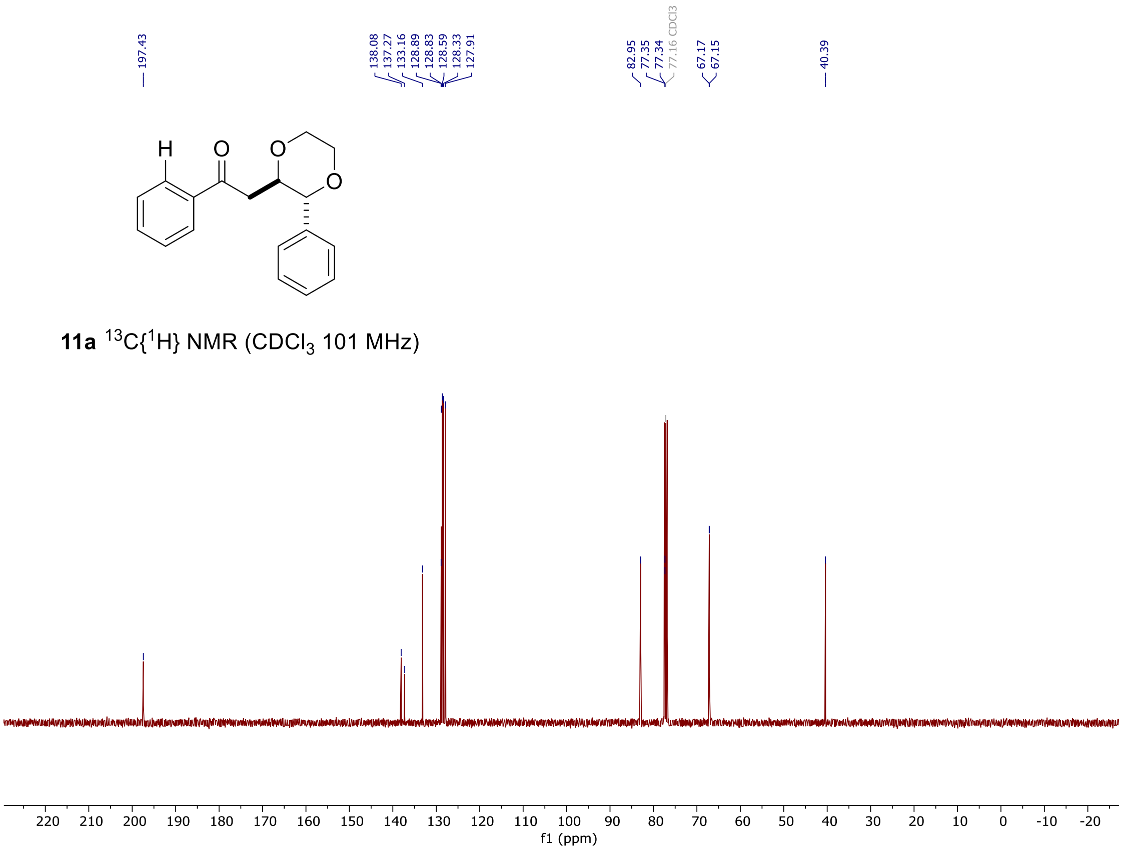

11a ^{13}C{^{1}H} NMR (CDCl$_3$ 101 MHz)

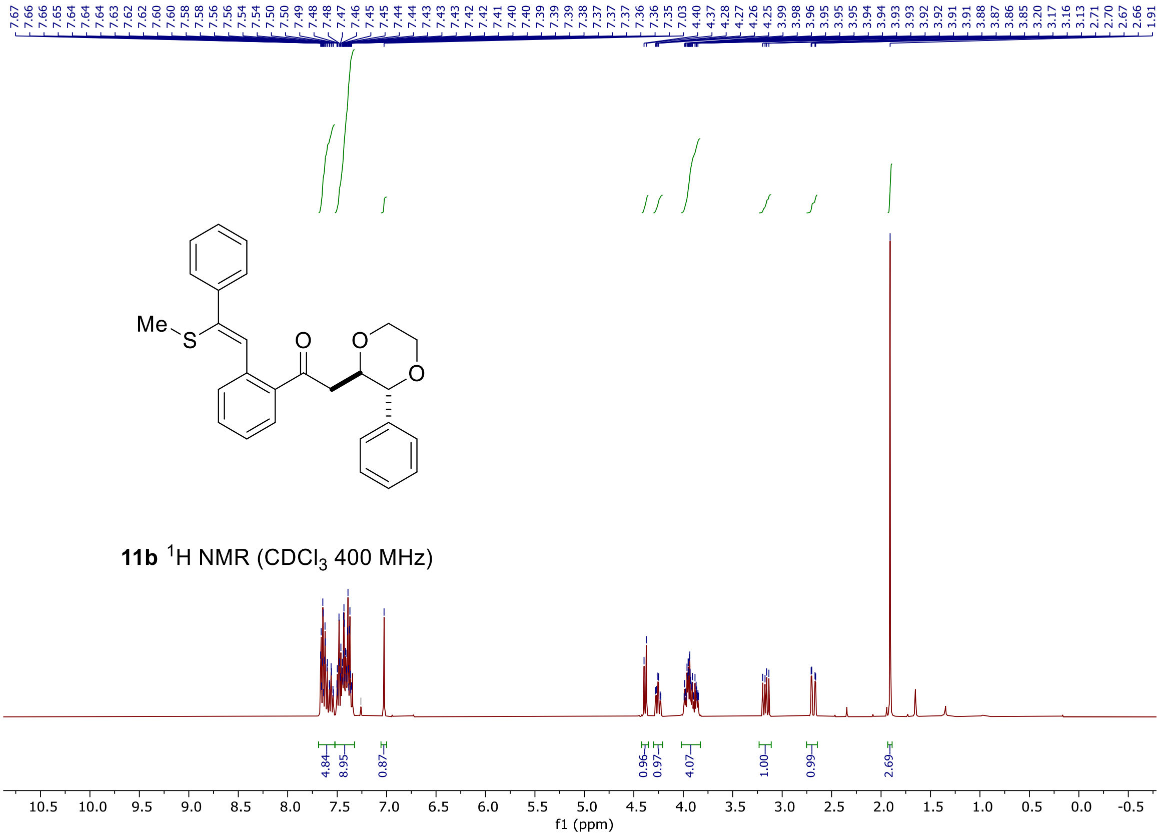

11b ^{1}H NMR (CDCl$_3$ 400 MHz)

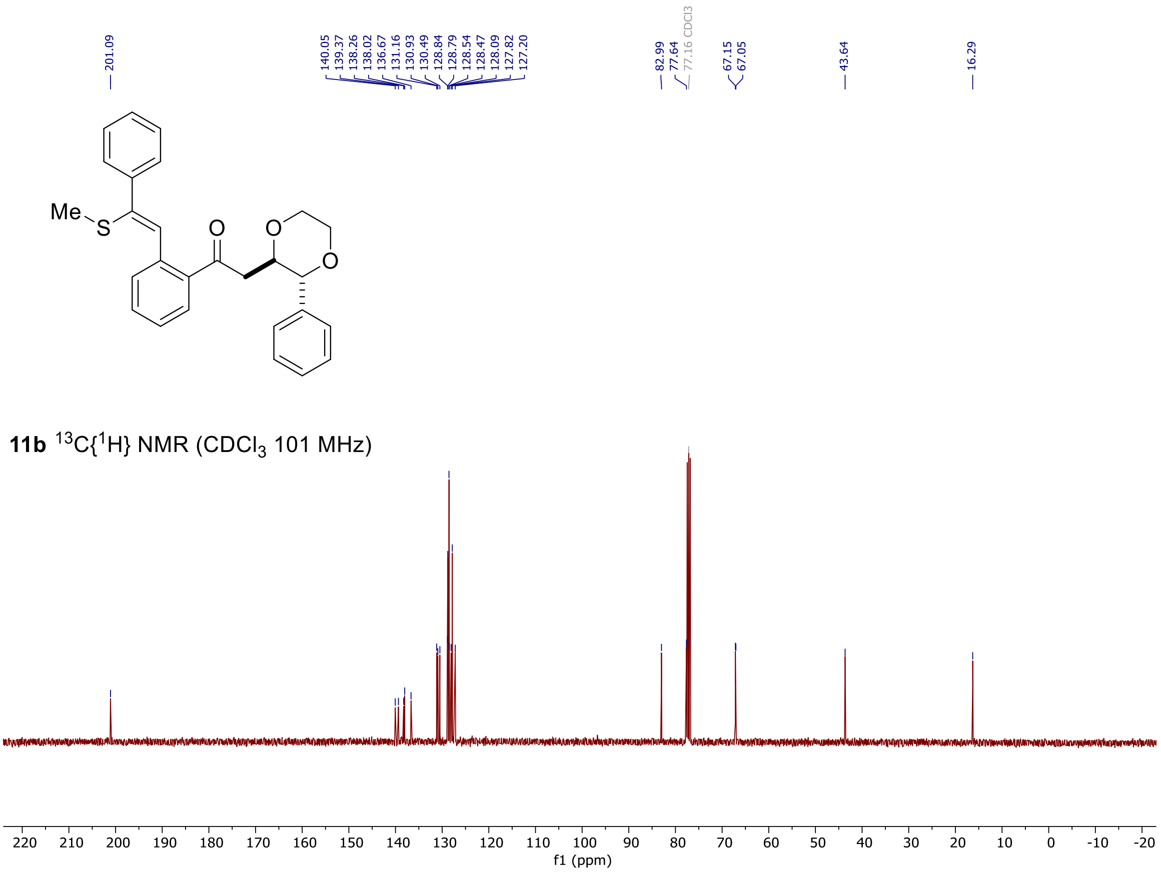

11b ^{13}C{^{1}H} NMR (CDCl$_3$ 101 MHz)

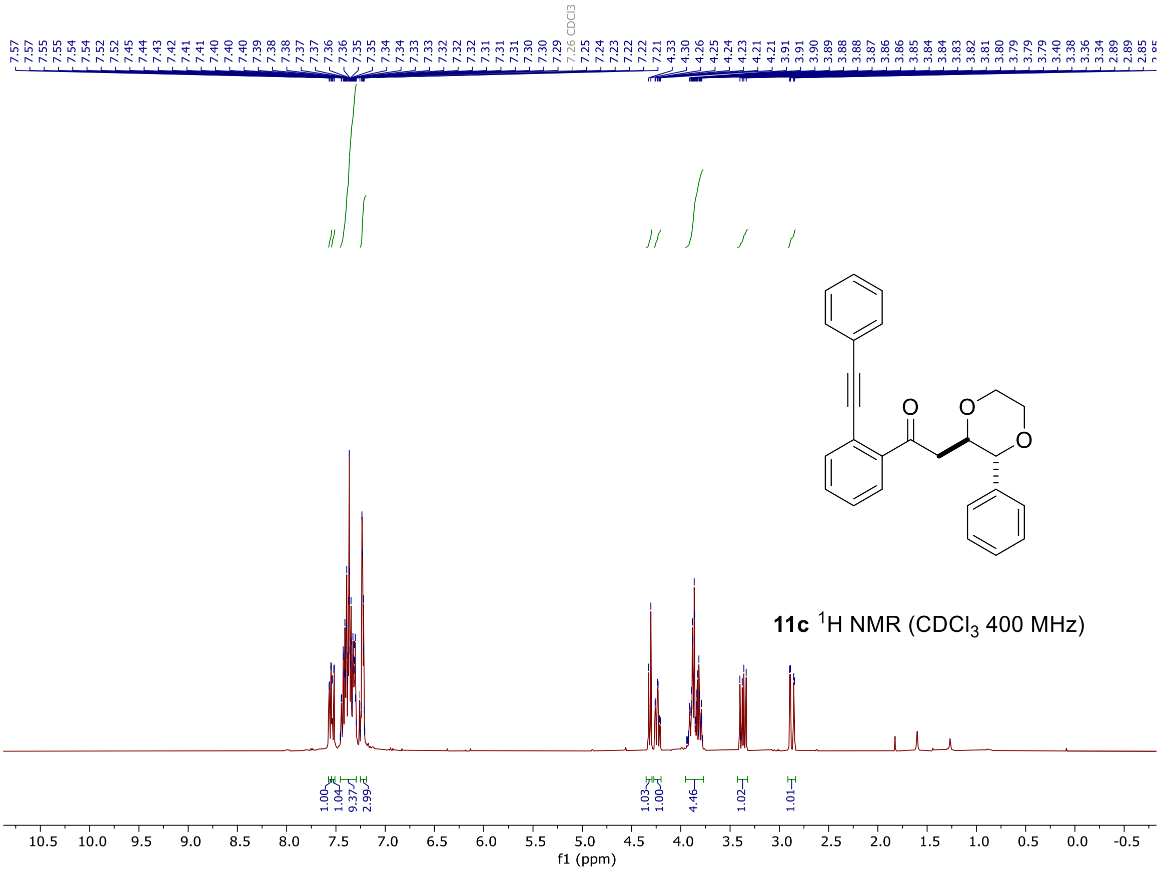
7.57 7.57 7.55 7.55 7.54 7.54 7.52 7.52 7.45 7.44 7.43 7.42 7.41 7.41 7.40 7.40 7.39 7.38 7.38 7.37 7.37 7.36 7.36 7.35 7.35 7.34 7.33 7.33 7.32 7.32 7.32 7.31 7.31 7.31 7.30 7.30 7.29 7.26 CDCl3 7.25 7.24 7.23 7.22 7.22 7.21 4.33 4.30 4.26 4.25 4.24 4.23 4.21 4.21 3.91 3.91 3.90 3.89 3.88 3.88 3.87 3.86 3.86 3.85 3.84 3.84 3.83 3.82 3.81 3.80 3.79 3.79 3.40 3.38 3.36 3.34 2.89 2.89 2.85
O
O
O
11c ^{1}H NMR (CDCl$_3$ 400 MHz)
1.00 1.04 9.37 2.99 1.03 1.00 4.46 1.02 1.01
10.5 10.0 9.5 9.0 8.5 8.0 7.5 7.0 6.5 6.0 5.5 5.0 4.5 4.0 3.5 3.0 2.5 2.0 1.5 1.0 0.5 0.0 -0.5
f1 (ppm)

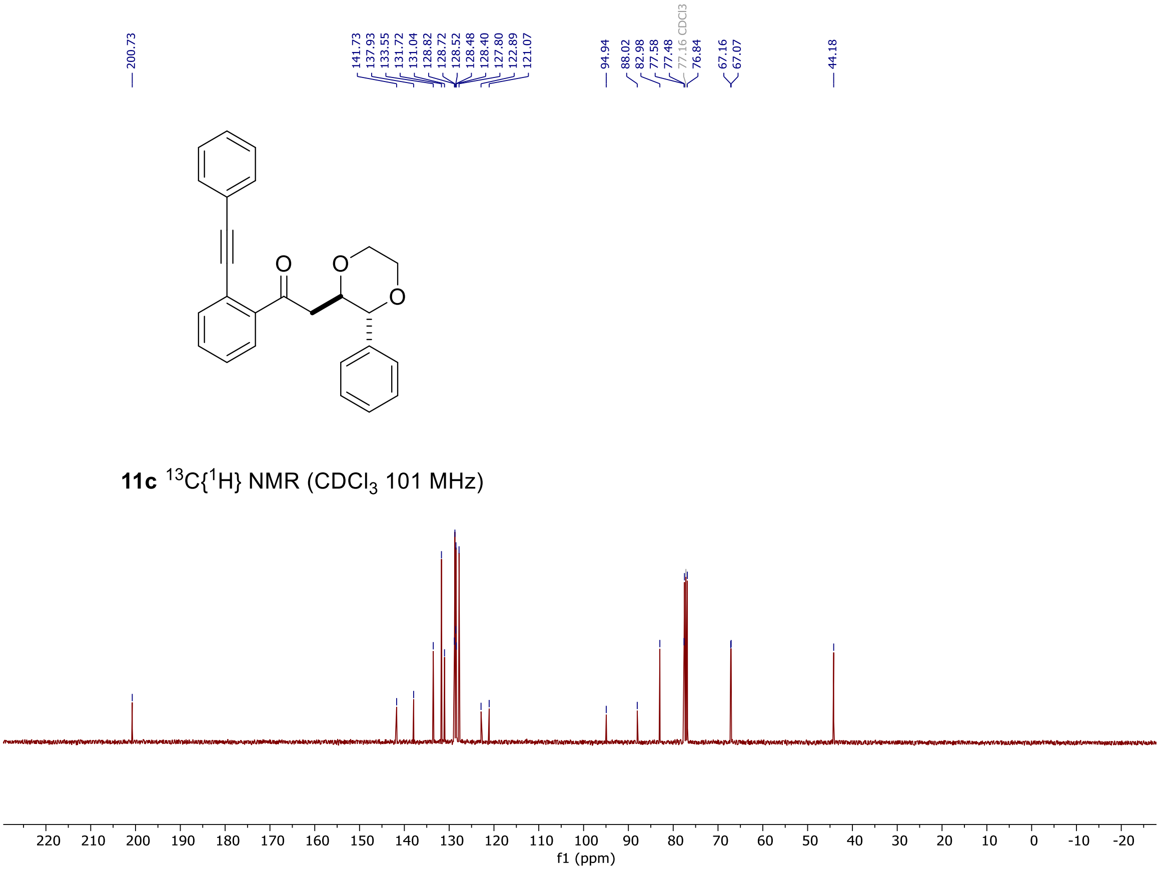
200.73
141.73 137.93 133.55 131.72 131.04 128.82 128.72 128.52 128.48 128.40 127.80 122.89 121.07
94.94 88.02 82.98 77.58 77.48 77.16 CDCl3 76.84 67.16 67.07
44.18
O
O
O
11c ^{13}C{^{1}H} NMR (CDCl$_3$ 101 MHz)
220 210 200 190 180 170 160 150 140 130 120 110 100 90 80 70 60 50 40 30 20 10 0 -10 -20
f1 (ppm)

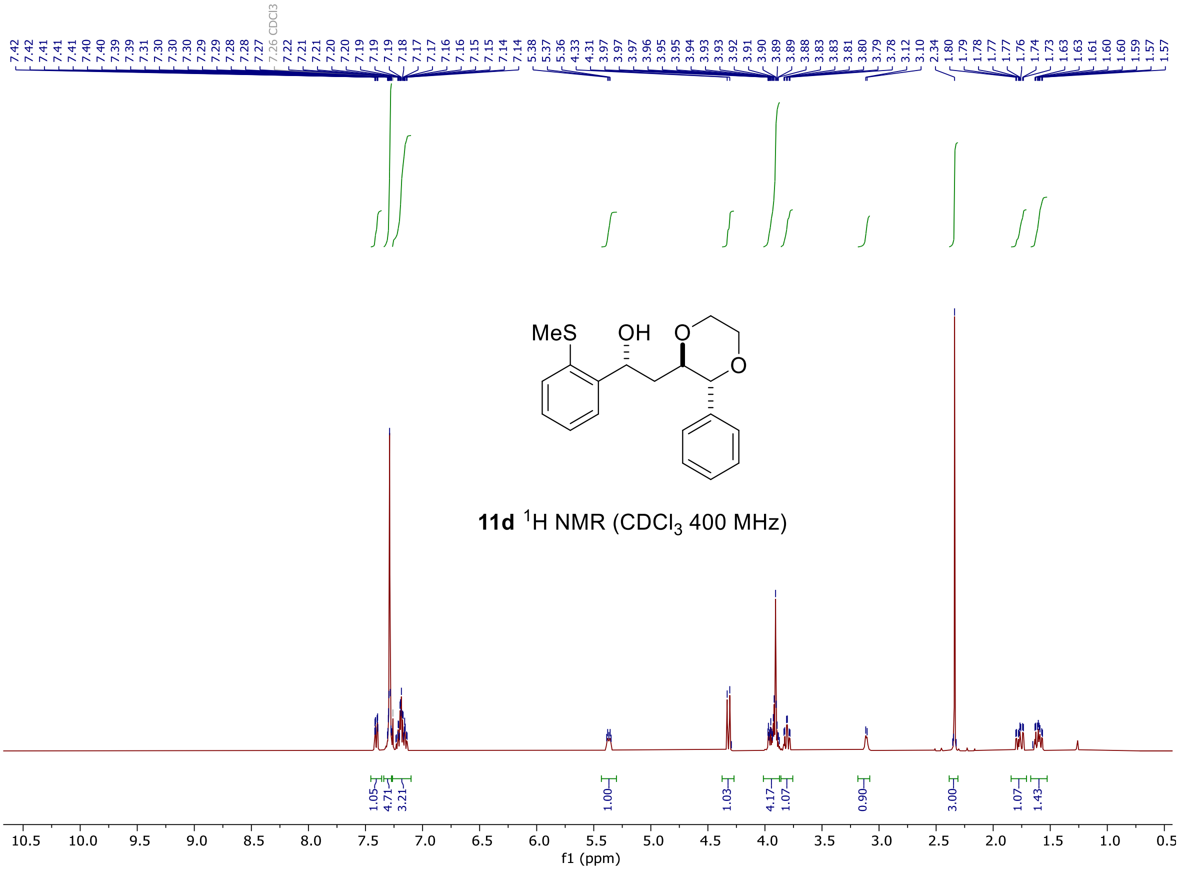
7.42 7.42 7.41 7.41 7.40 7.40 7.39 7.39 7.31 7.30 7.30 7.29 7.29 7.28 7.27 7.26 CDCl3 7.22 7.21 7.21 7.20 7.20 7.19 7.19 7.19 7.18 7.17 7.17 7.16 7.15 7.15 7.14 5.38 5.37 5.36 4.33 4.31 3.97 3.97 3.96 3.95 3.95 3.94 3.93 3.93 3.92 3.91 3.90 3.89 3.89 3.88 3.83 3.83 3.81 3.80 3.79 3.78 3.12 3.10 2.34 1.80 1.79 1.78 1.77 1.77 1.76 1.74 1.73 1.63 1.63 1.61 1.60 1.59 1.57 1.57
MeS
OH
O
O
11d 1H NMR (CDCl3 400 MHz)
1.05 4.71 3.21 1.00 1.03 4.17 1.07 0.90 3.00 1.07 1.43
10.5 10.0 9.5 9.0 8.5 8.0 7.5 7.0 6.5 6.0 5.5 5.0 4.5 4.0 3.5 3.0 2.5 2.0 1.5 1.0 0.5
f1 (ppm)

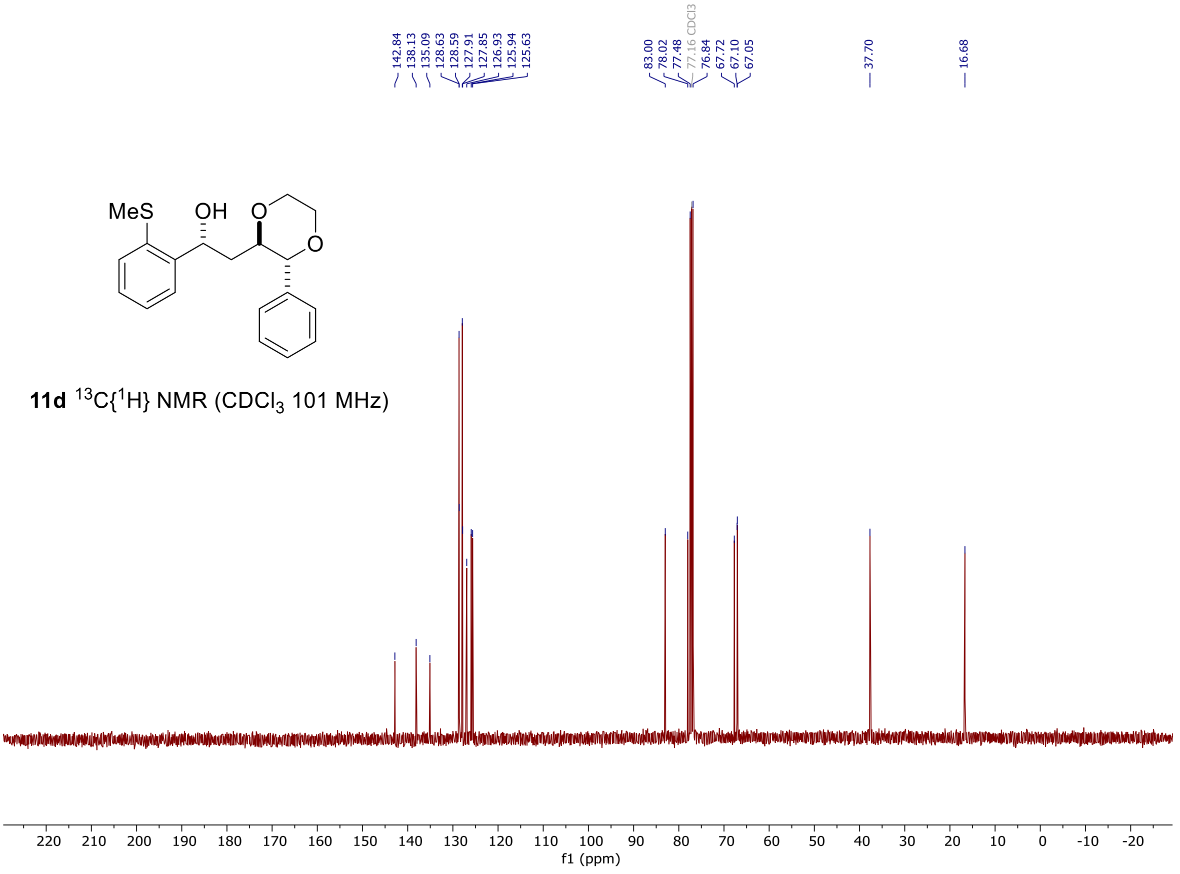
142.84 138.13 135.09 128.63 128.59 127.91 127.85 126.93 125.94 125.63 83.00 78.02 77.48 77.16 CDCl3 76.84 67.72 67.10 67.05 37.70 16.68
MeS
OH
O
O
11d 13C{1H} NMR (CDCl3 101 MHz)
220 210 200 190 180 170 160 150 140 130 120 110 100 90 80 70 60 50 40 30 20 10 0 -10 -20
f1 (ppm)

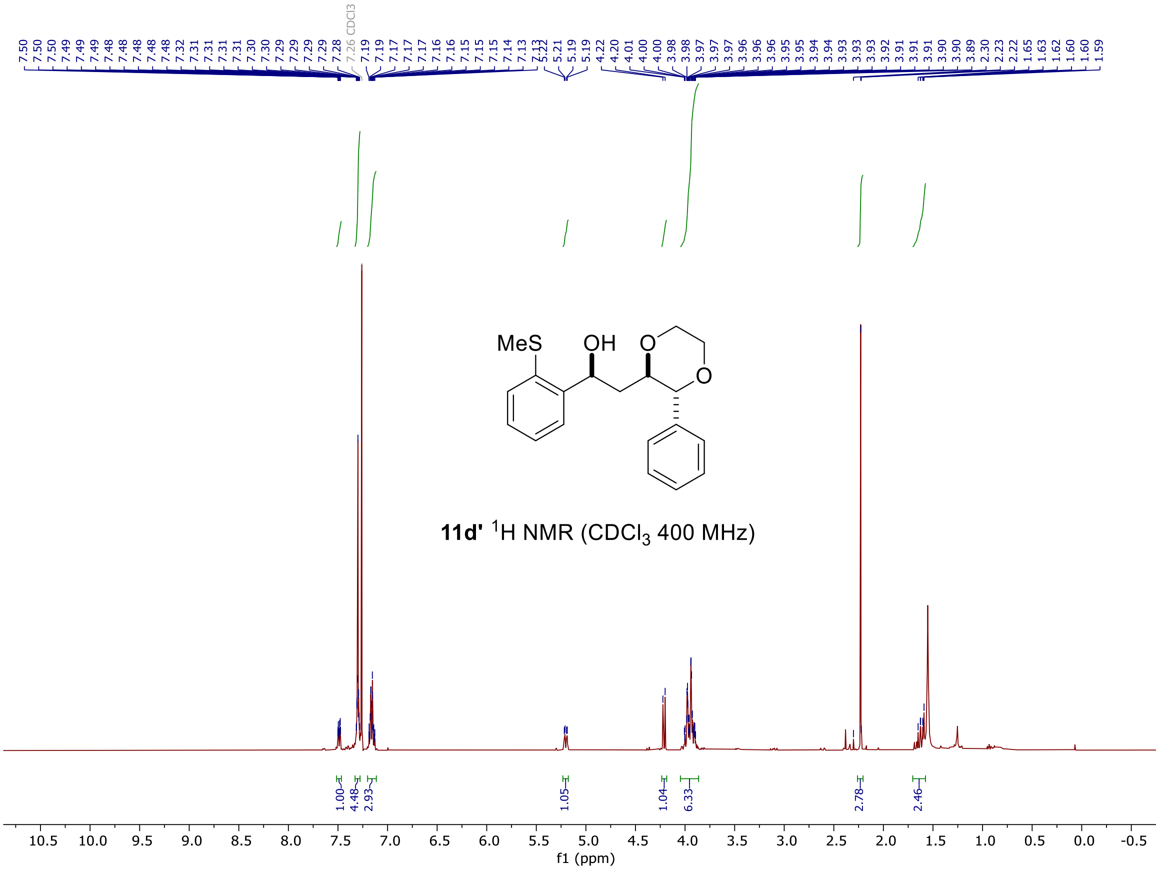

11d' ^{1}H NMR ($CDCl_3$ 400 MHz)

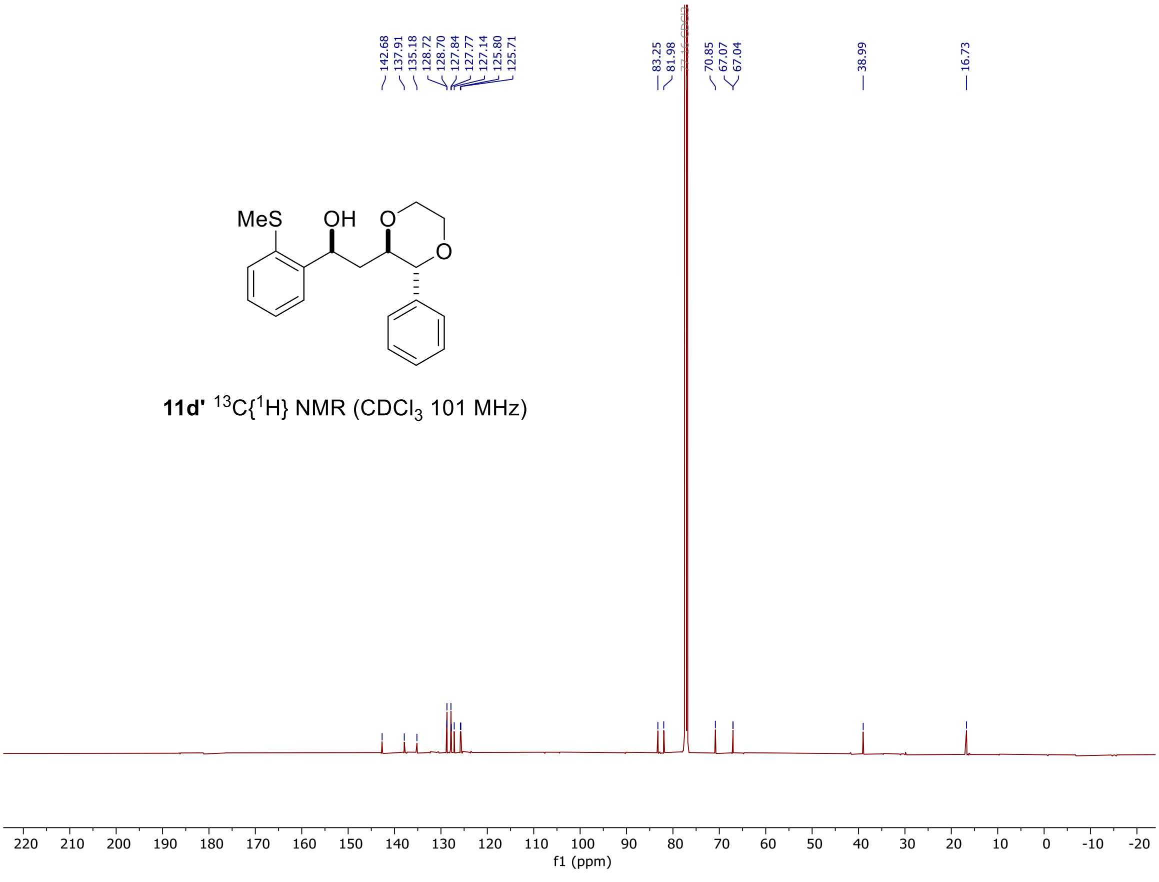

11d' $^{13}C\{^1H\}$ NMR ($CDCl_3$ 101 MHz)

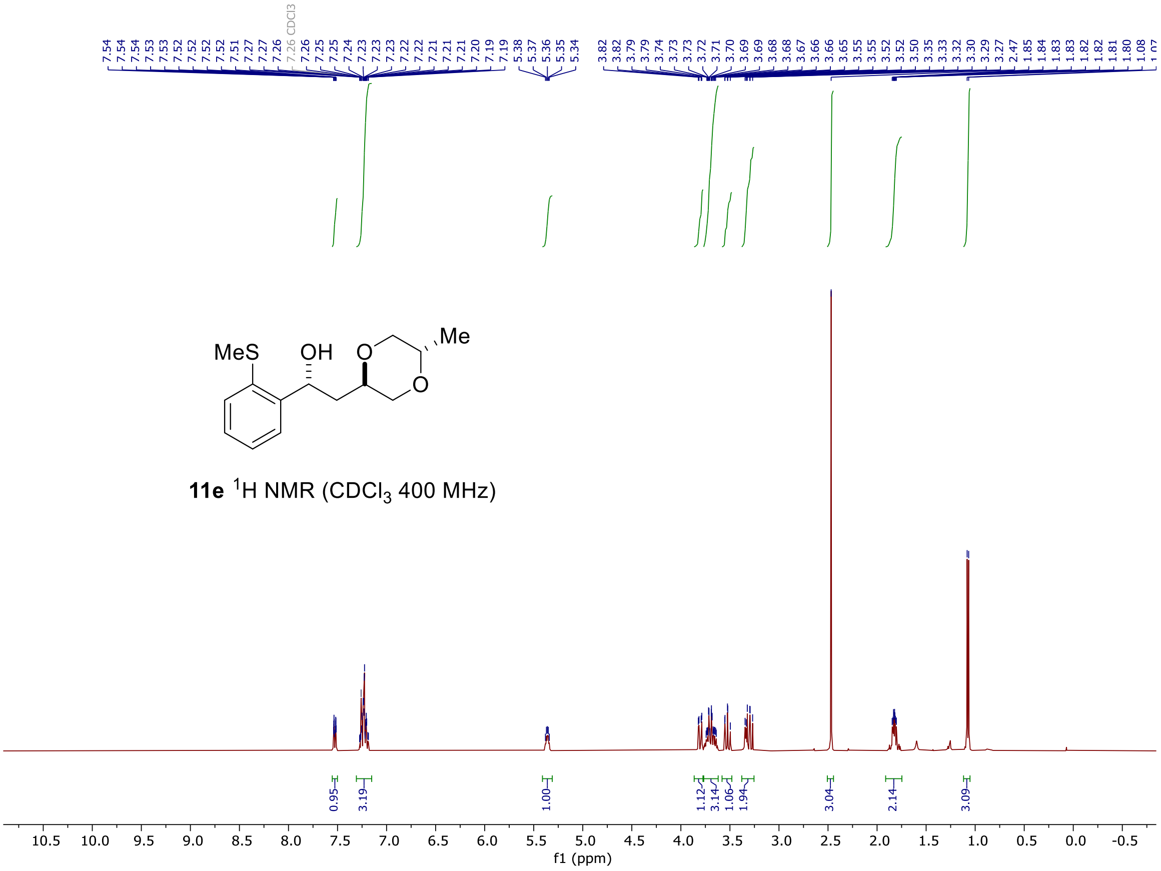
7.54 7.54 7.54 7.53 7.53 7.52 7.52 7.52 7.52 7.51 7.27 7.27 7.26 CDCl3 7.26 7.25 7.25 7.24 7.23 7.23 7.23 7.23 7.22 7.22 7.21 7.21 7.21 7.20 7.19 7.19
5.38 5.37 5.36 5.35 5.34
3.82 3.82 3.79 3.79 3.74 3.73 3.73 3.72 3.72 3.71 3.70 3.69 3.69 3.68 3.68 3.67 3.66 3.66 3.65 3.55 3.55 3.52 3.52 3.50 3.35 3.33 3.32 3.30 3.29 3.27 2.47 1.85 1.84 1.83 1.83 1.82 1.82 1.81 1.80 1.08
MeS
OH
O
Me
O
11e 1H NMR (CDCl3 400 MHz)
0.95 3.19 1.00 1.12 3.14 1.06 1.94 3.04 2.14 3.09
10.5 10.0 9.5 9.0 8.5 8.0 7.5 7.0 6.5 6.0 5.5 5.0 4.5 4.0 3.5 3.0 2.5 2.0 1.5 1.0 0.5 0.0 -0.5
f1 (ppm)

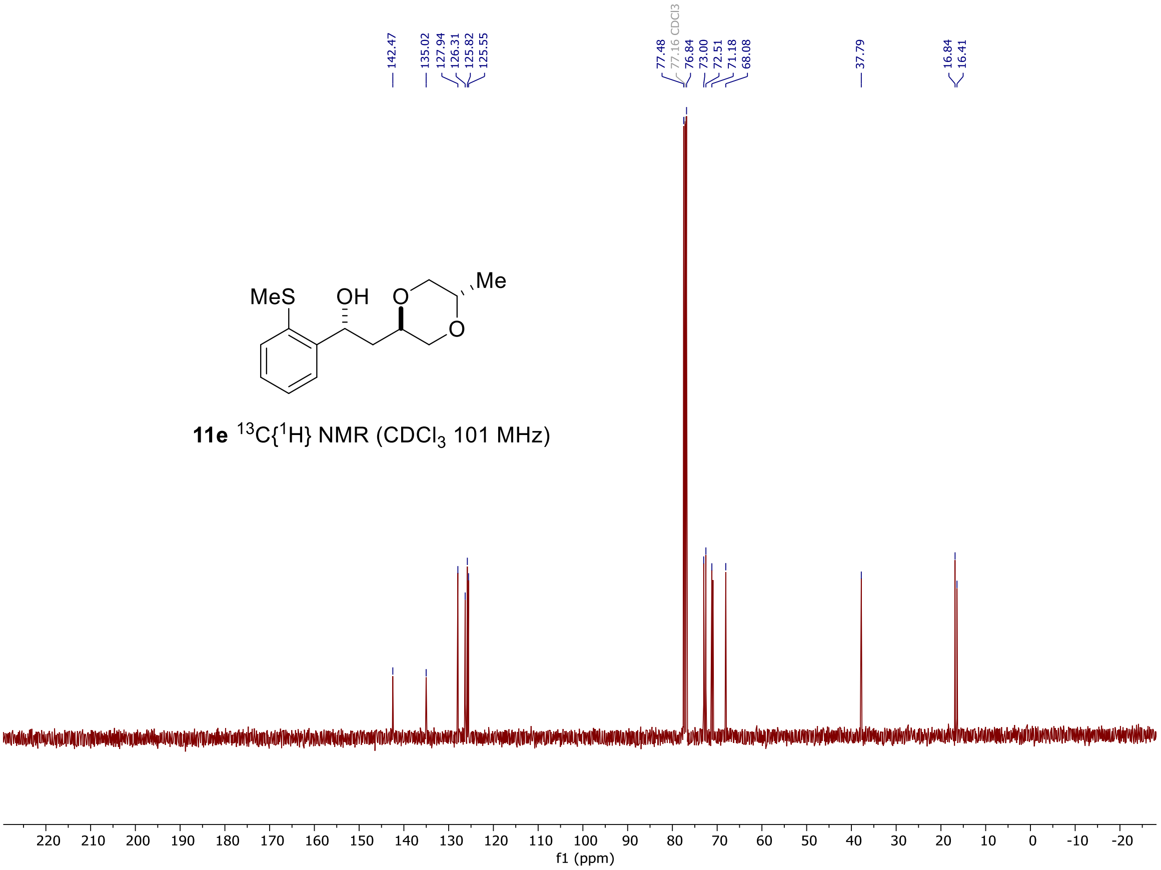
142.47
135.02 127.94 126.31 125.82 125.55
77.48 77.16 CDCl3 76.84 73.00 72.51 71.18 68.08
37.79
16.84 16.41
MeS
OH
O
Me
O
11e 13C{1H} NMR (CDCl3 101 MHz)
220 210 200 190 180 170 160 150 140 130 120 110 100 90 80 70 60 50 40 30 20 10 0 -10 -20
f1 (ppm)

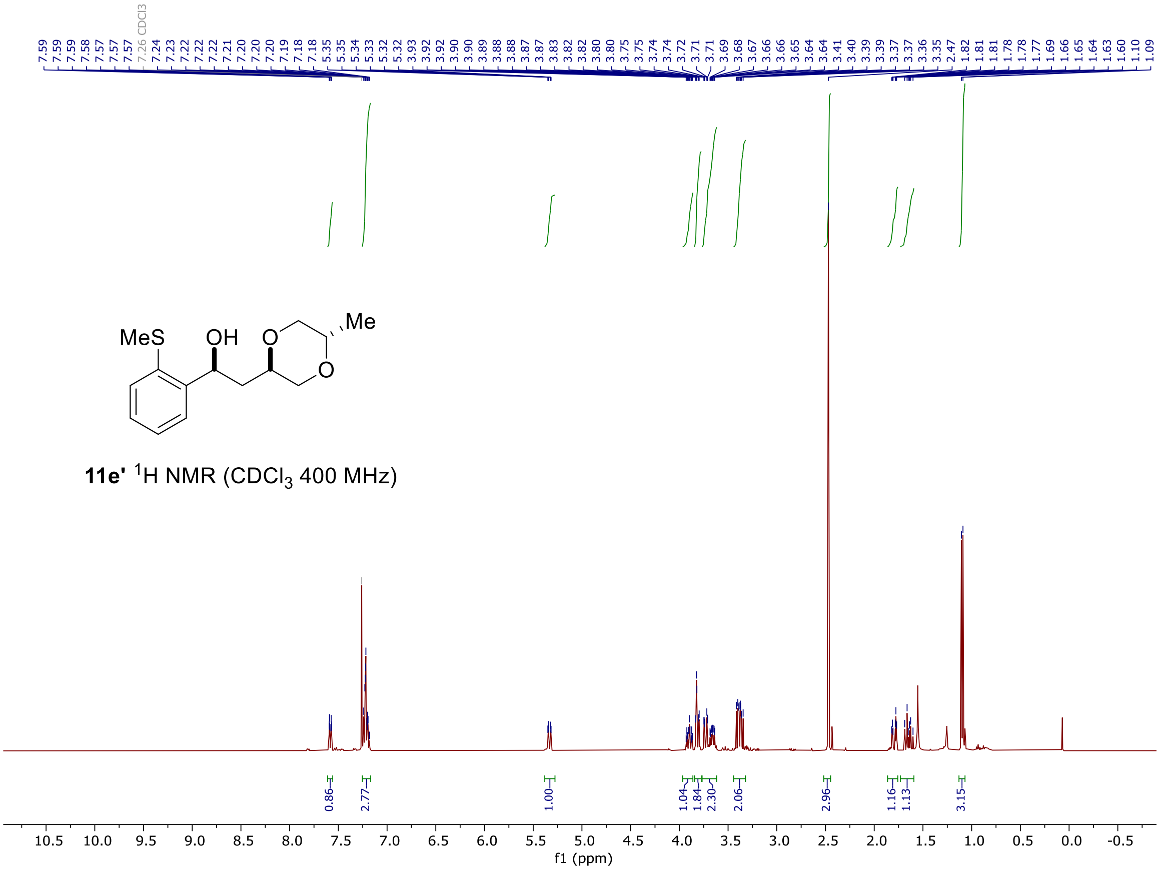

11e' ^{1}H NMR ($CDCl_3$ 400 MHz)

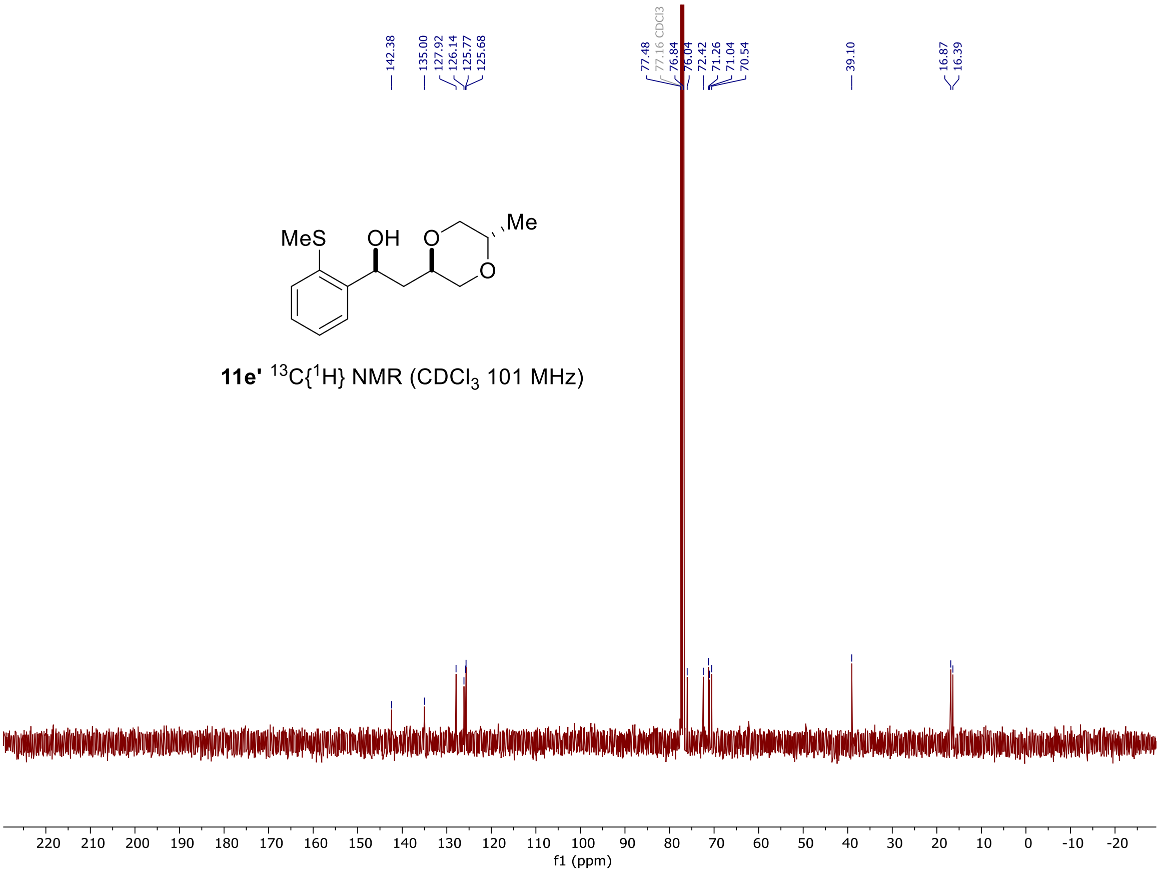

11e' ^{13}C{^{1}H} NMR ($CDCl_3$ 101 MHz)

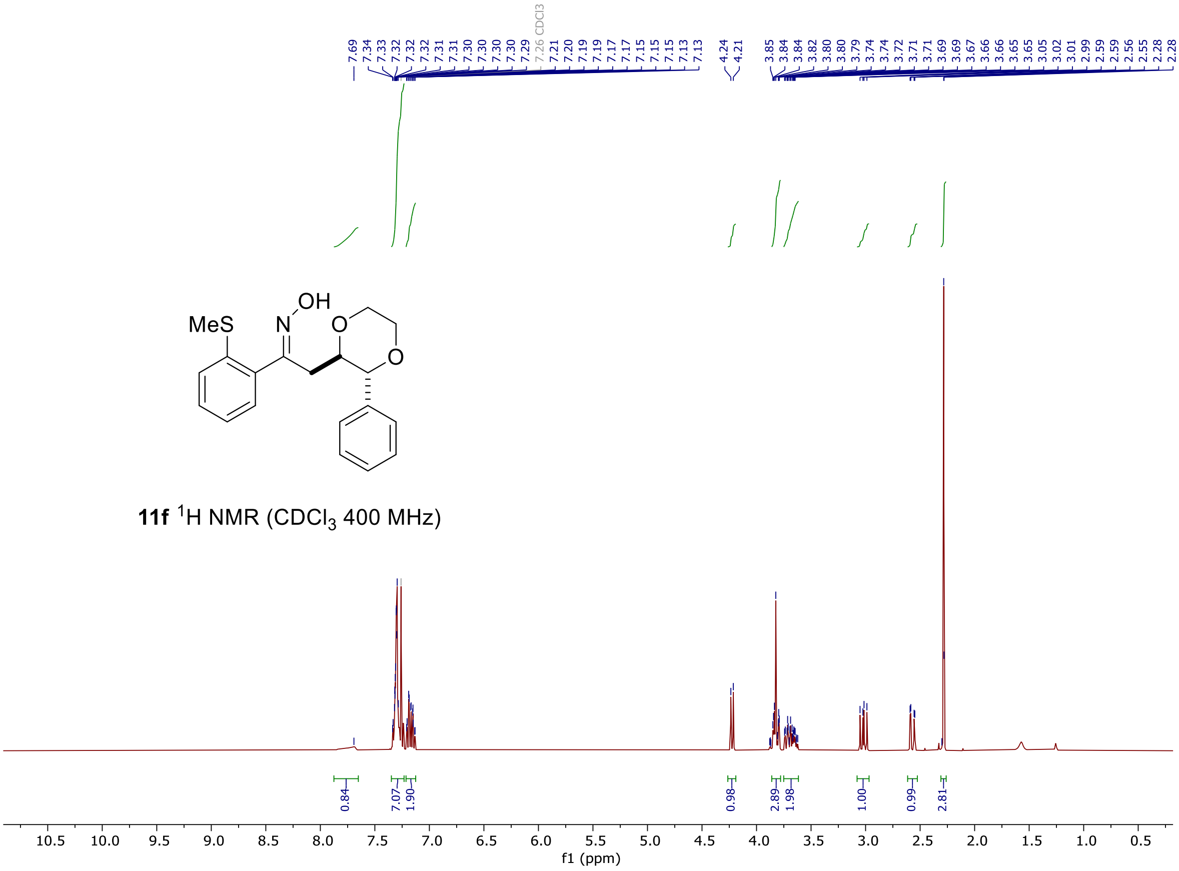

11f ^{1}H NMR (CDCl$_3$ 400 MHz)

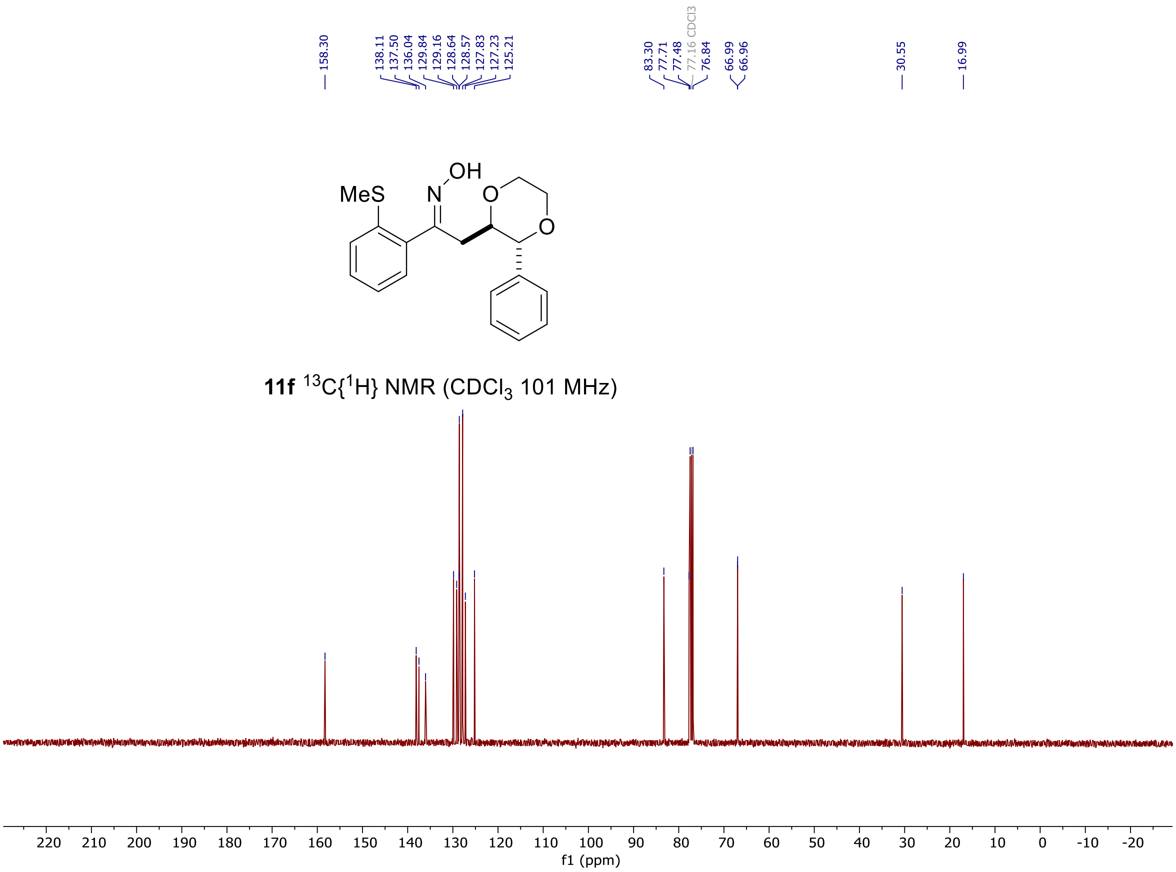

11f ^{13}C{^{1}H} NMR (CDCl$_3$ 101 MHz)

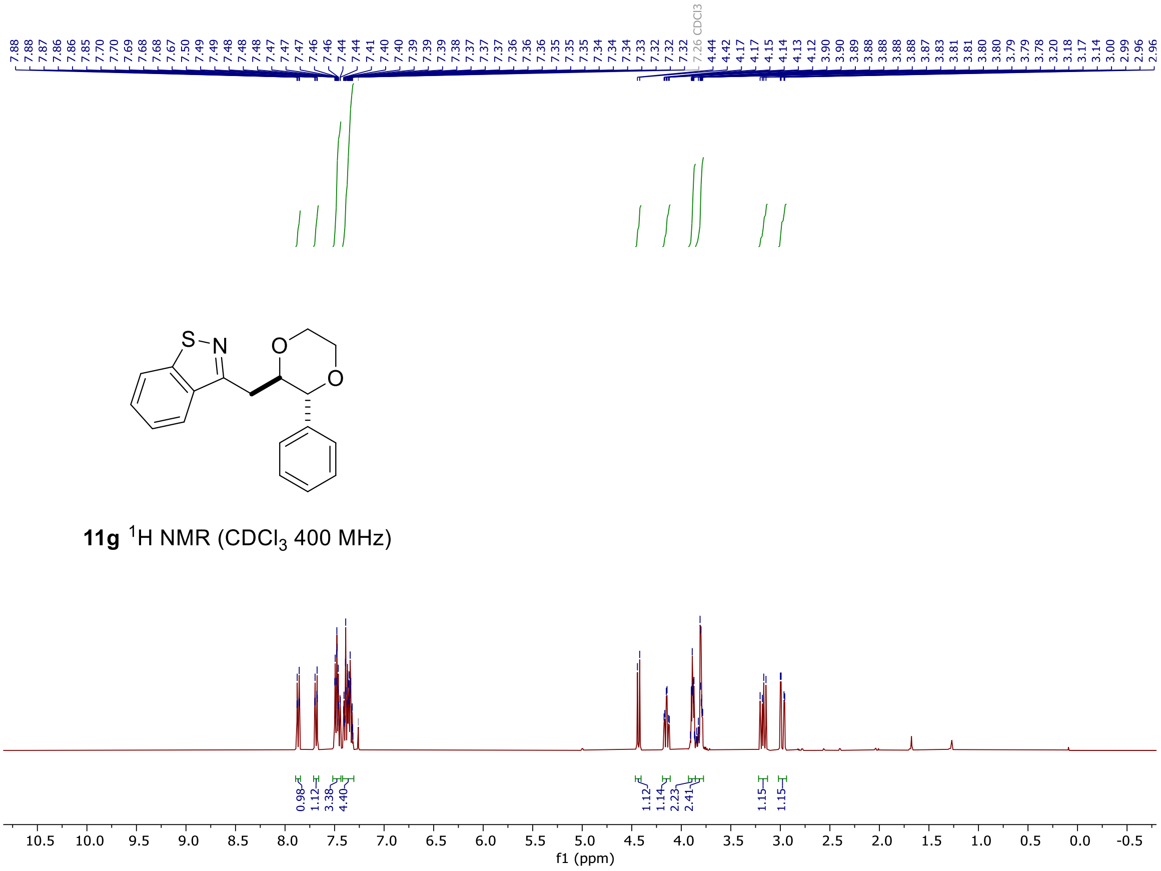

11g ^{1}H NMR ($CDCl_3$ 400 MHz)

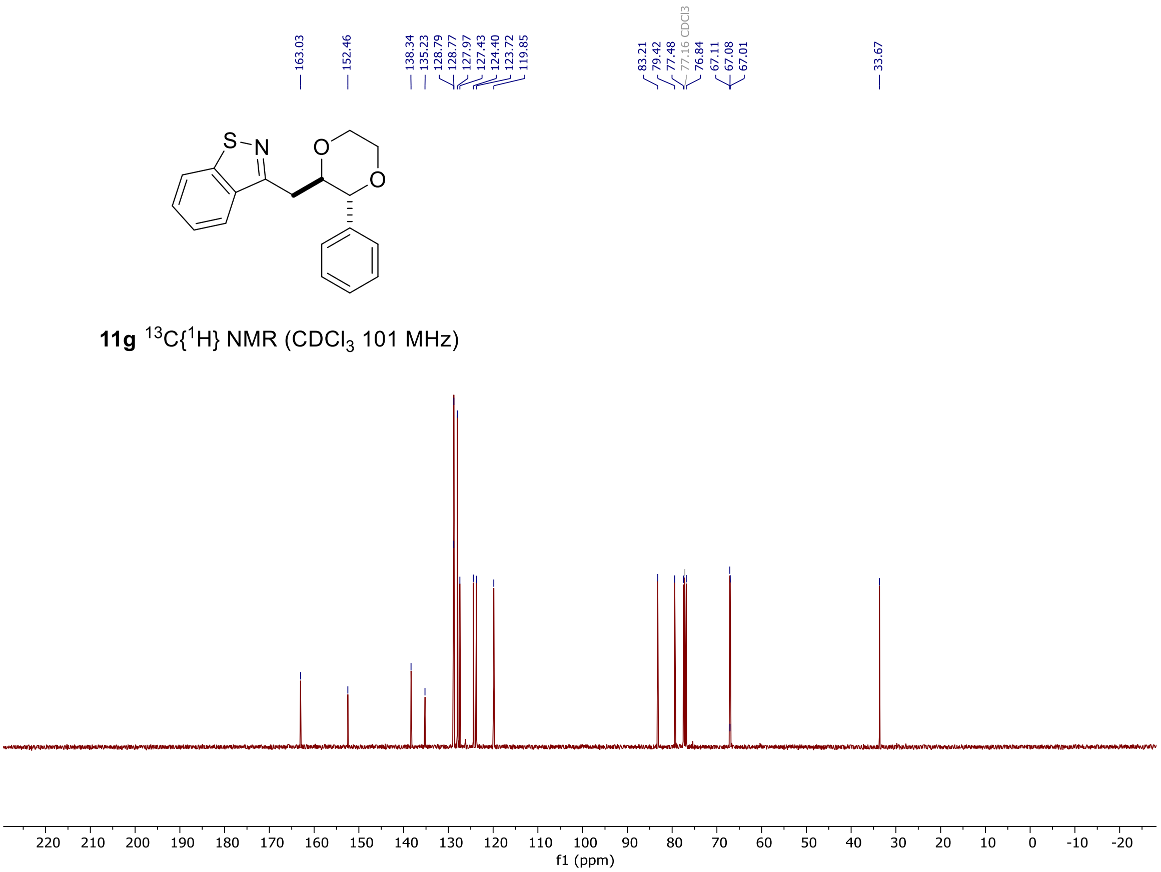

11g $^{13}C\{^{1}H\}$ NMR ($CDCl_3$ 101 MHz)

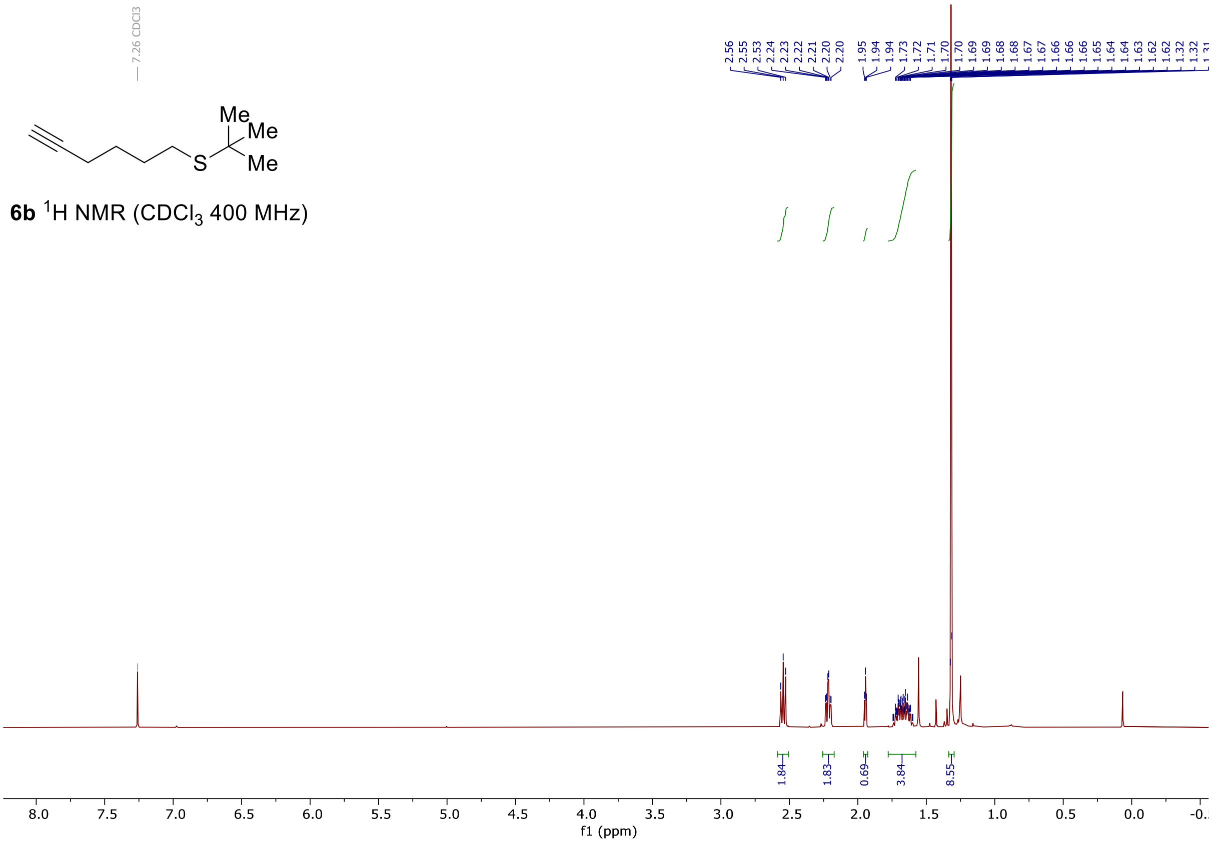
7.26 CDCl3
Me
Me
Me
S
6b ¹H NMR (CDCl3 400 MHz)
2.56
2.55
2.53
2.24
2.23
2.22
2.21
2.20
2.20
1.95
1.94
1.94
1.73
1.72
1.71
1.70
1.70
1.69
1.69
1.68
1.68
1.67
1.66
1.66
1.66
1.65
1.64
1.64
1.63
1.62
1.62
1.32
1.84
1.83
0.69
3.84
8.55
f1 (ppm)

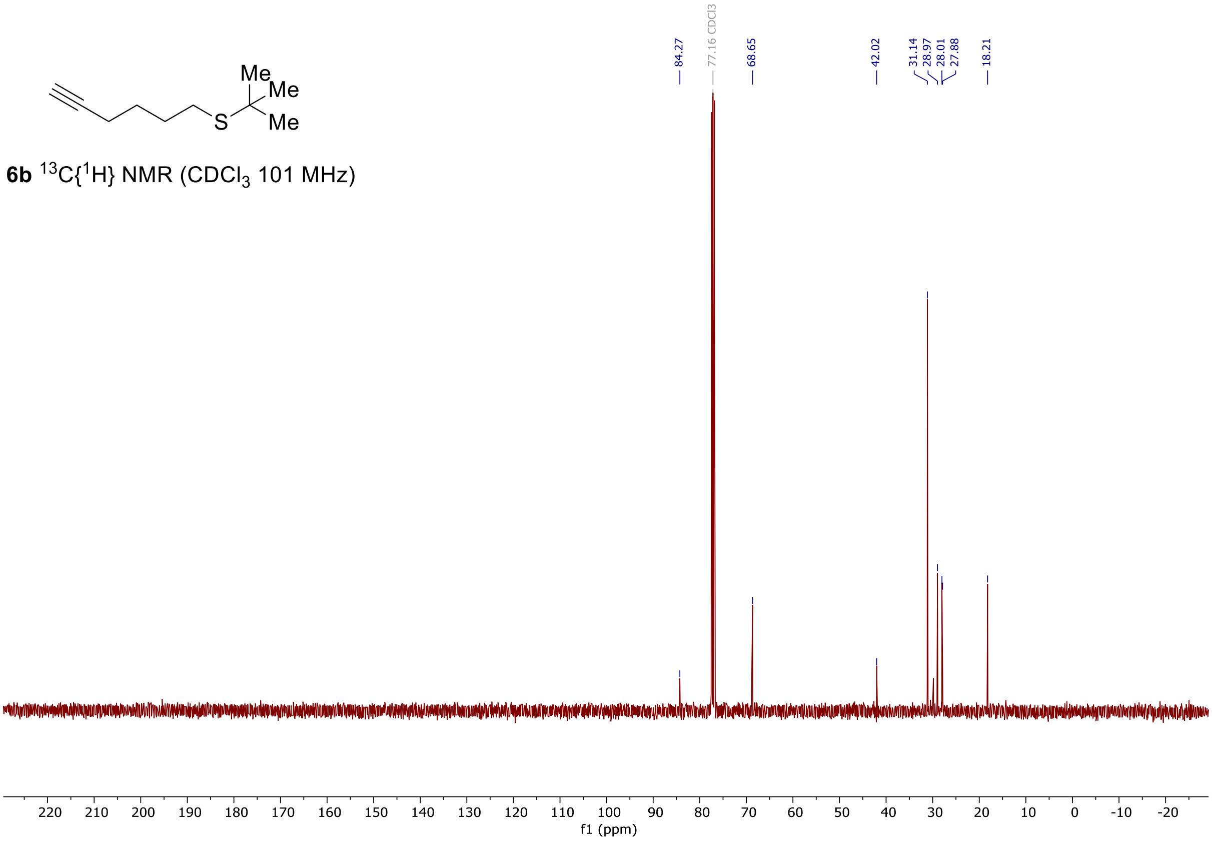
84.27
77.16 CDCl3
68.65
42.02
31.14
28.97
28.01
27.88
18.21
Me
Me
Me
S
6b ¹³C{¹H} NMR (CDCl3 101 MHz)
f1 (ppm)

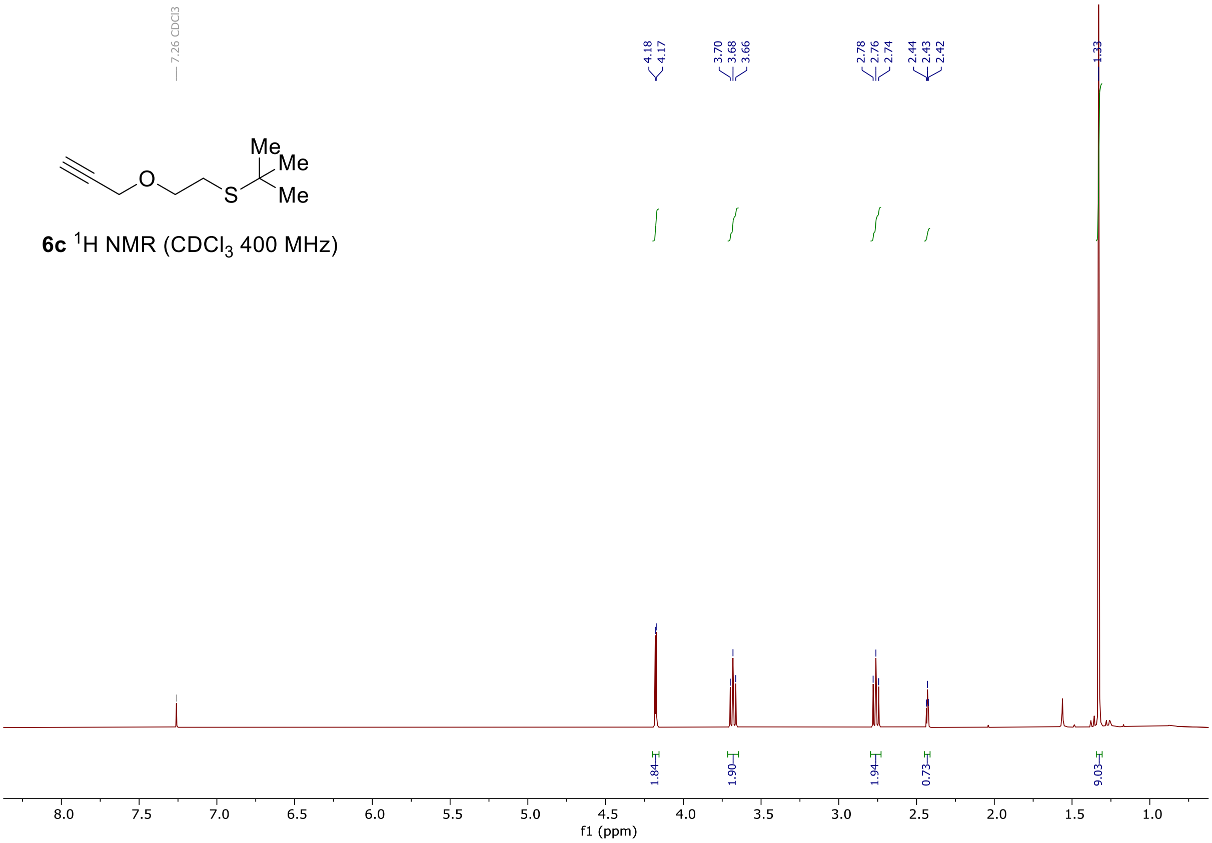

6c ^{1}H NMR ($CDCl_3$ 400 MHz)

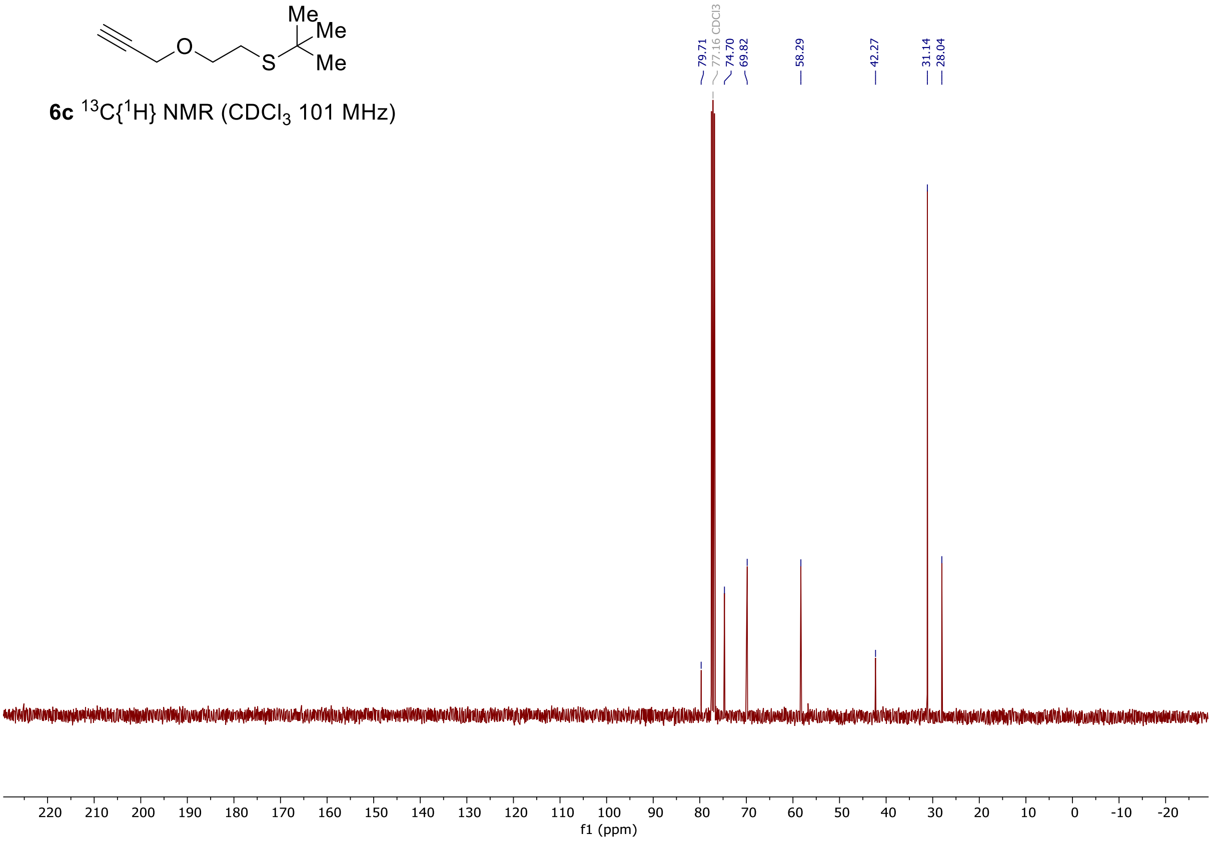

6c $^{13}C\{^{1}H\}$ NMR ($CDCl_3$ 101 MHz)

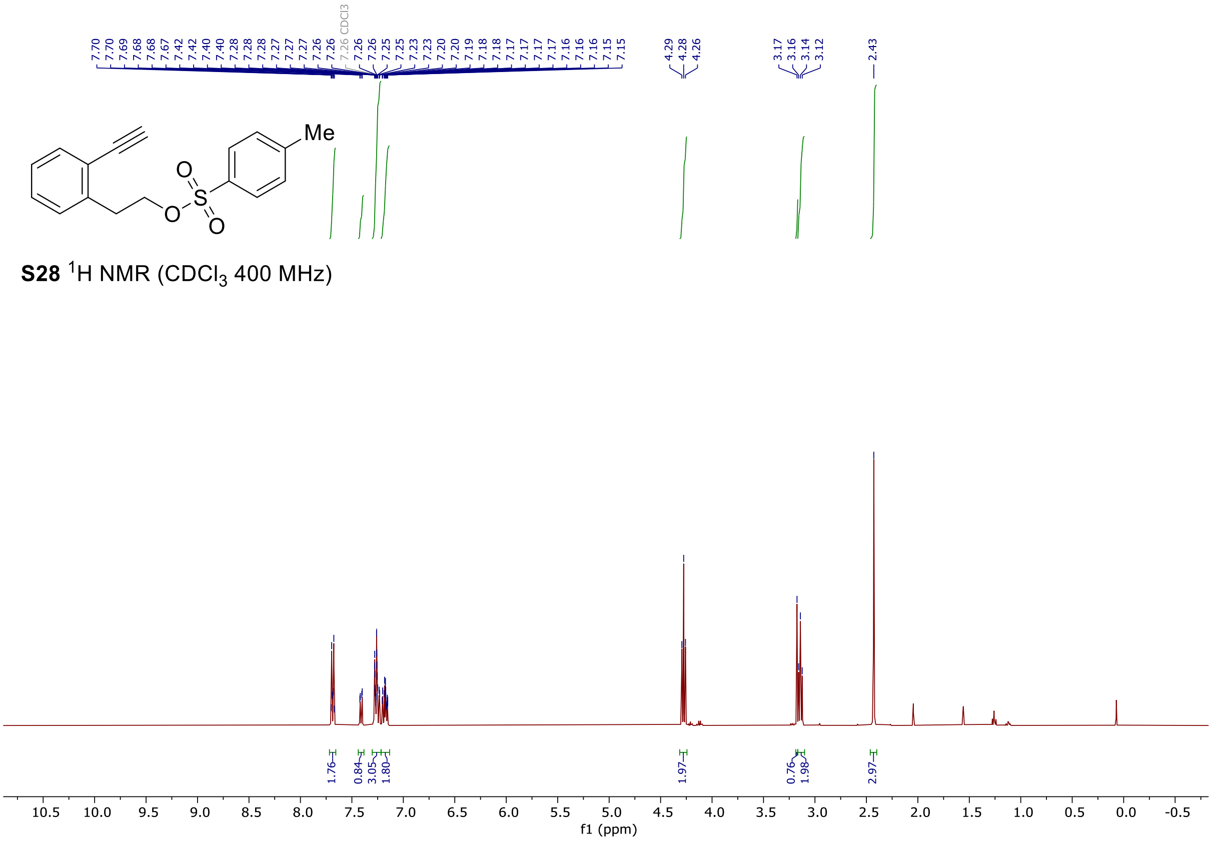

S28 ^{1}H NMR ($CDCl_3$ 400 MHz)

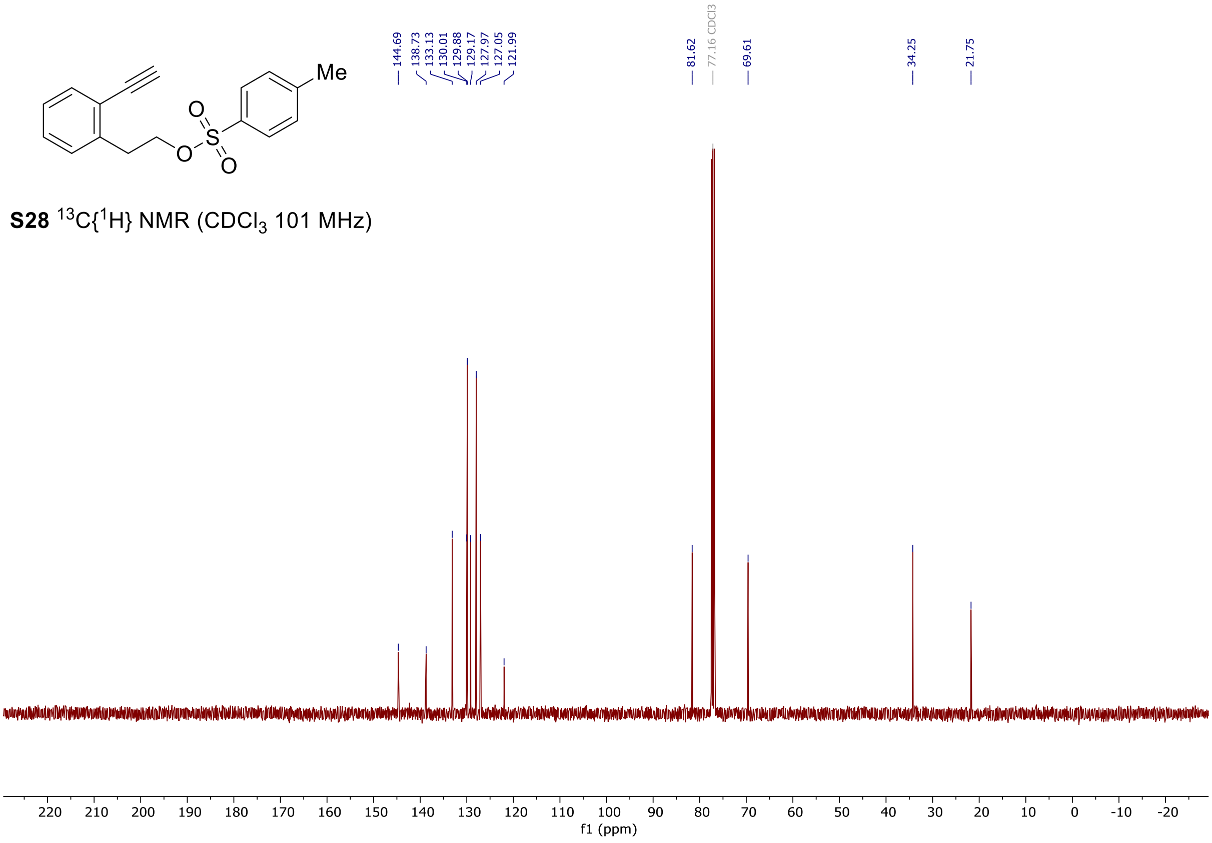

S28 ^{13}C{^{1}H} NMR ($CDCl_3$ 101 MHz)

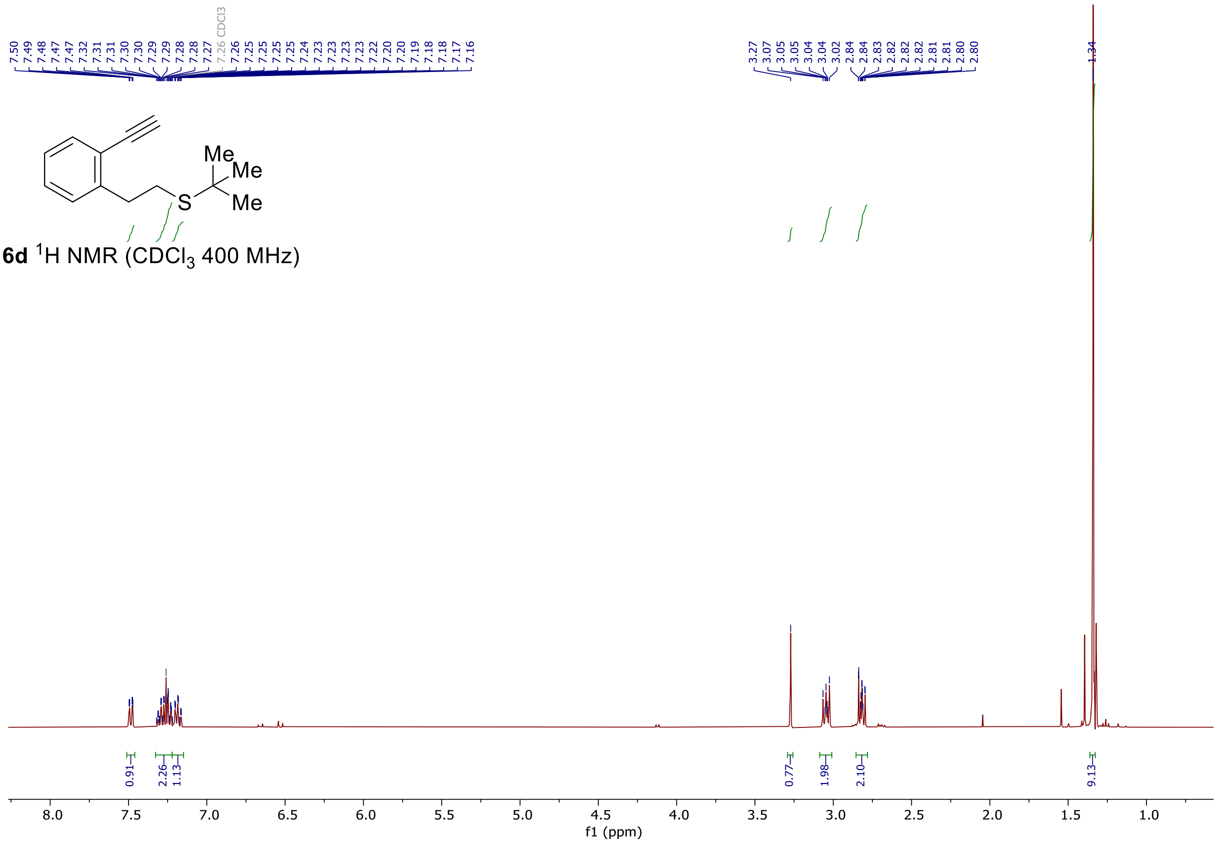

6d ^{1}H NMR ($CDCl_3$ 400 MHz)

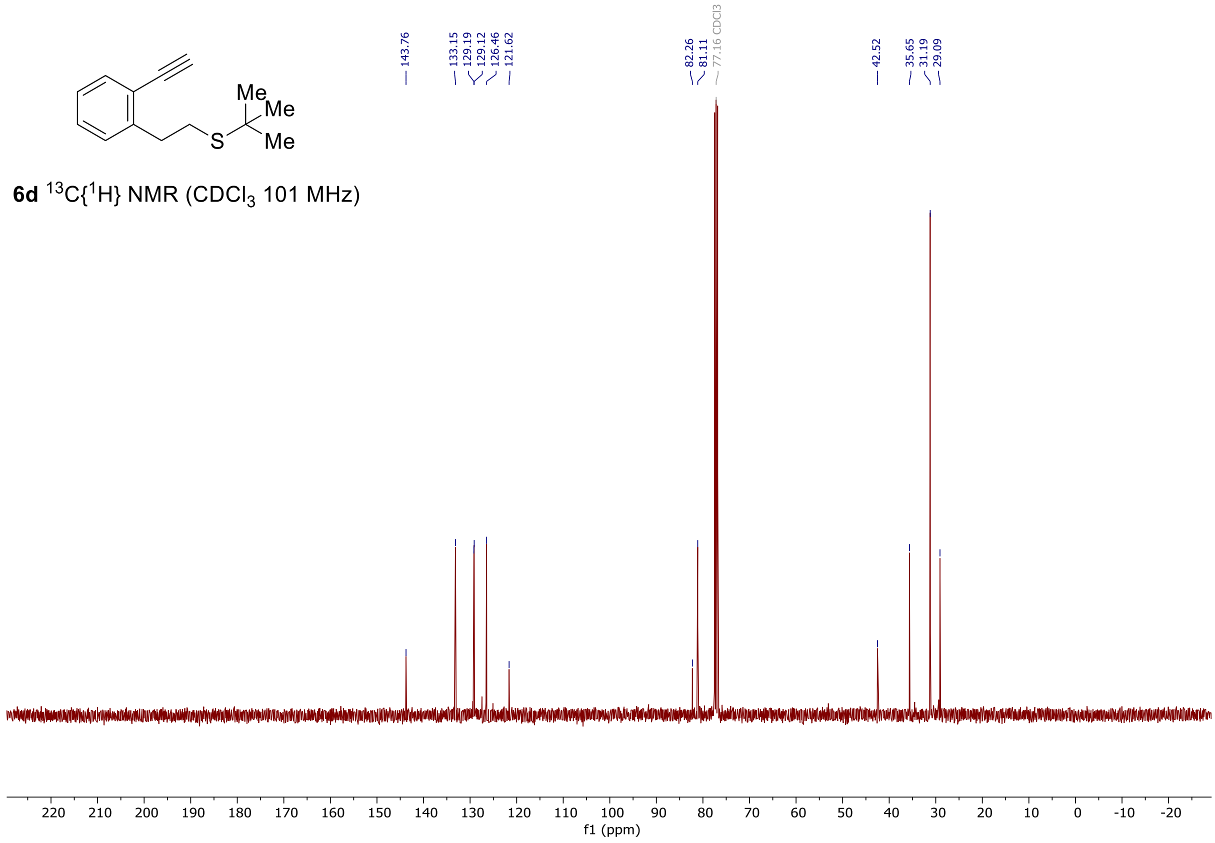

6d $^{13}C\{^{1}H\}$ NMR ($CDCl_3$ 101 MHz)

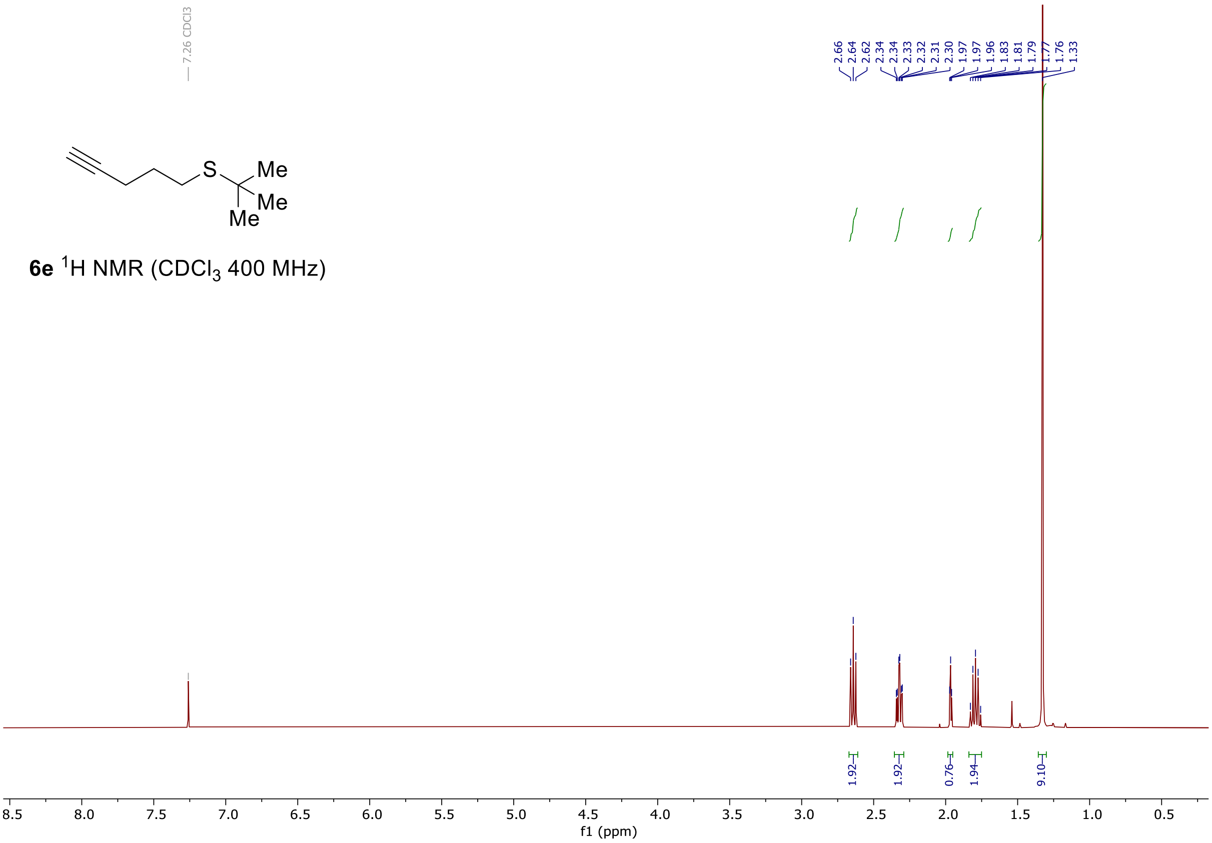

6e ^{1}H NMR ($CDCl_3$ 400 MHz)

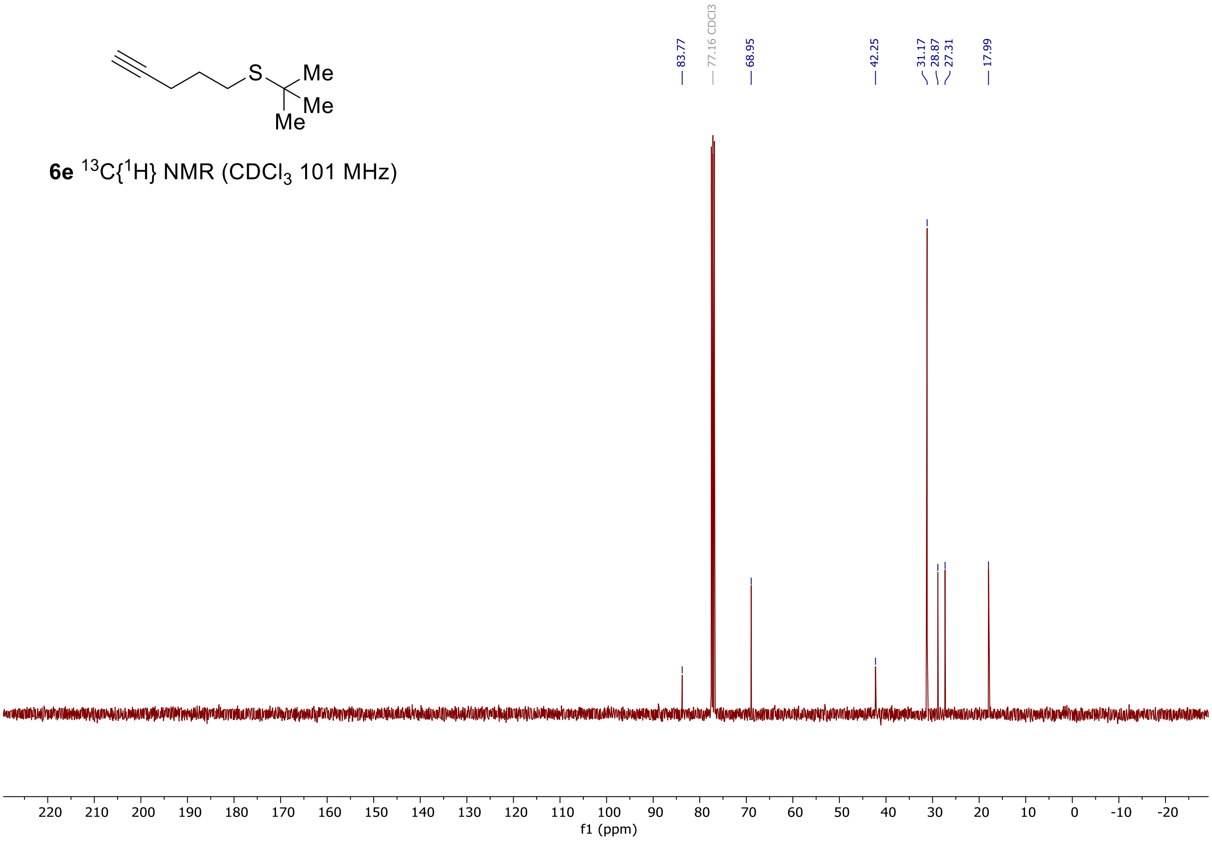

6e ^{13}C{^{1}H} NMR ($CDCl_3$ 101 MHz)

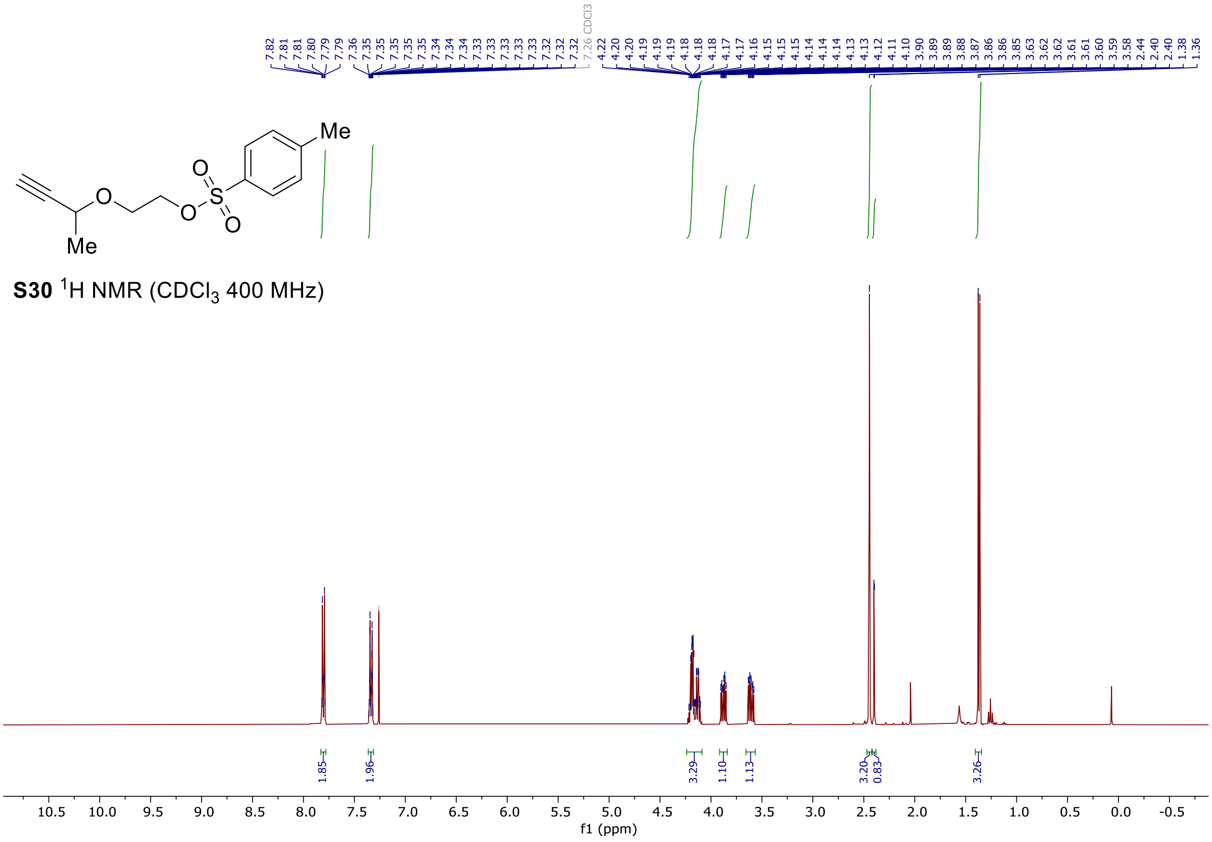

S30 ^{1}H NMR ($CDCl_3$ 400 MHz)

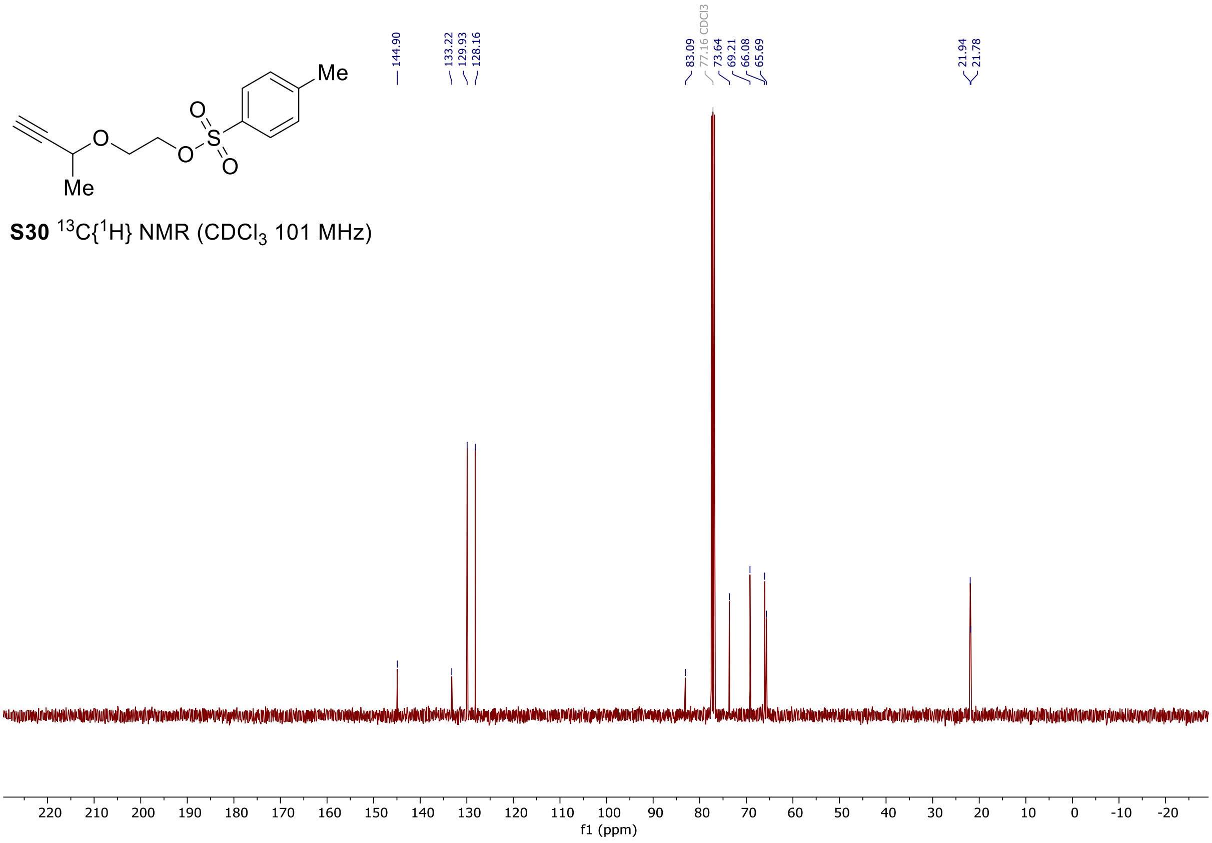

S30 ^{13}C{^{1}H} NMR ($CDCl_3$ 101 MHz)

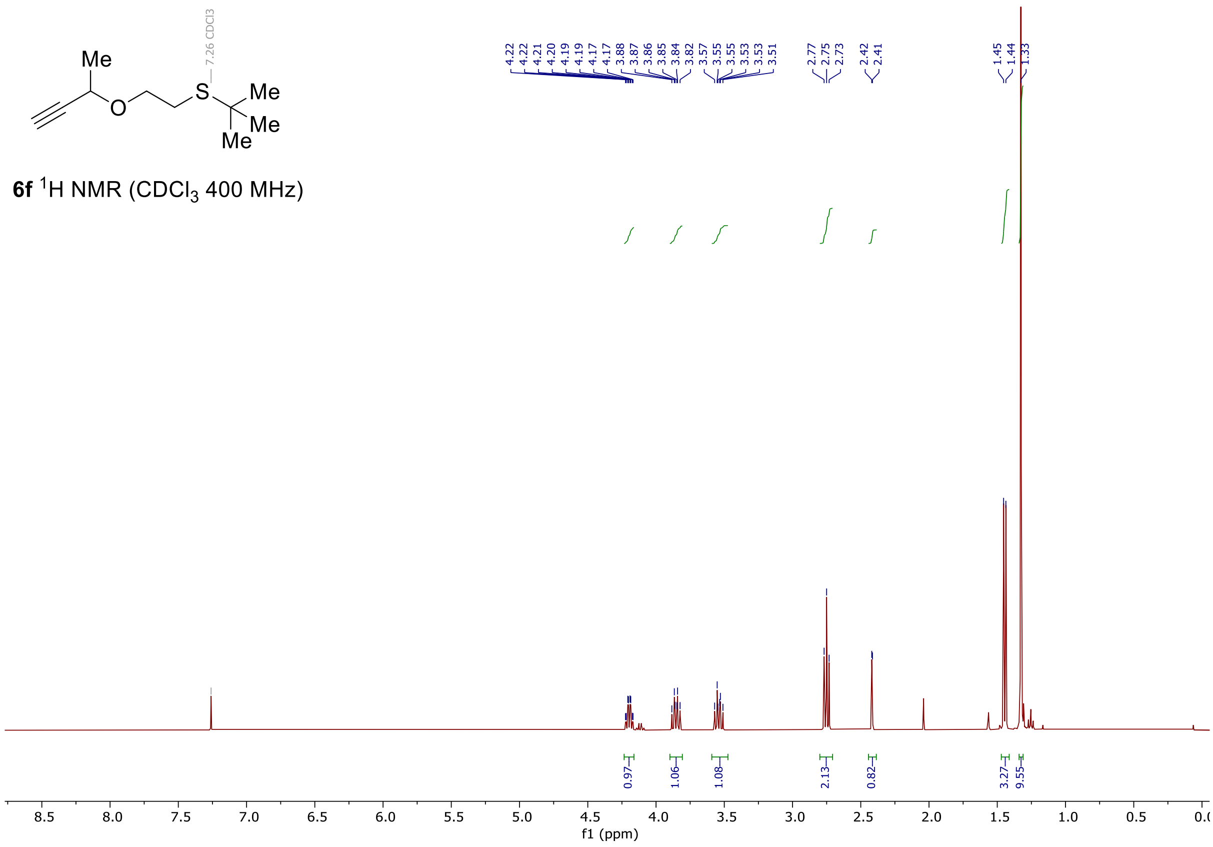

6f ^{1}H NMR ($CDCl_3$ 400 MHz)

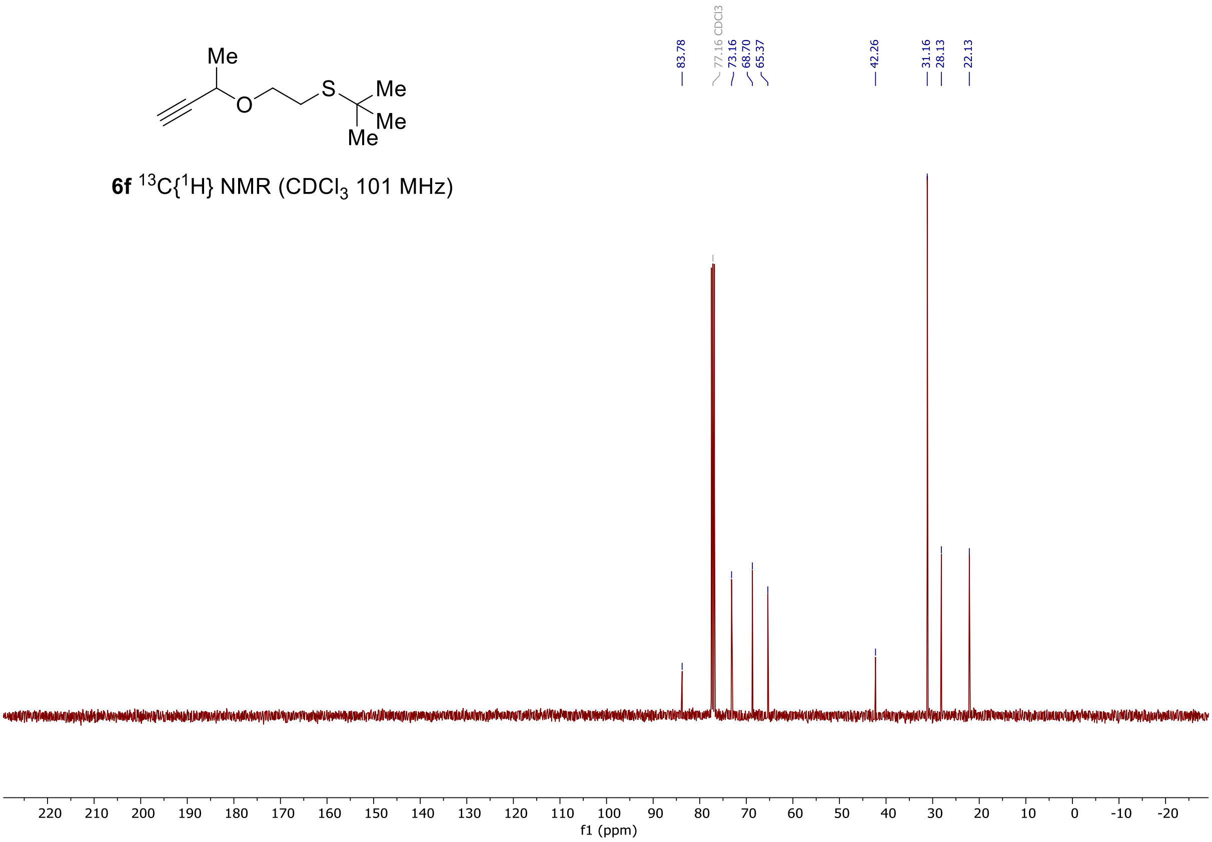

6f $^{13}C\{^{1}H\}$ NMR ($CDCl_3$ 101 MHz)

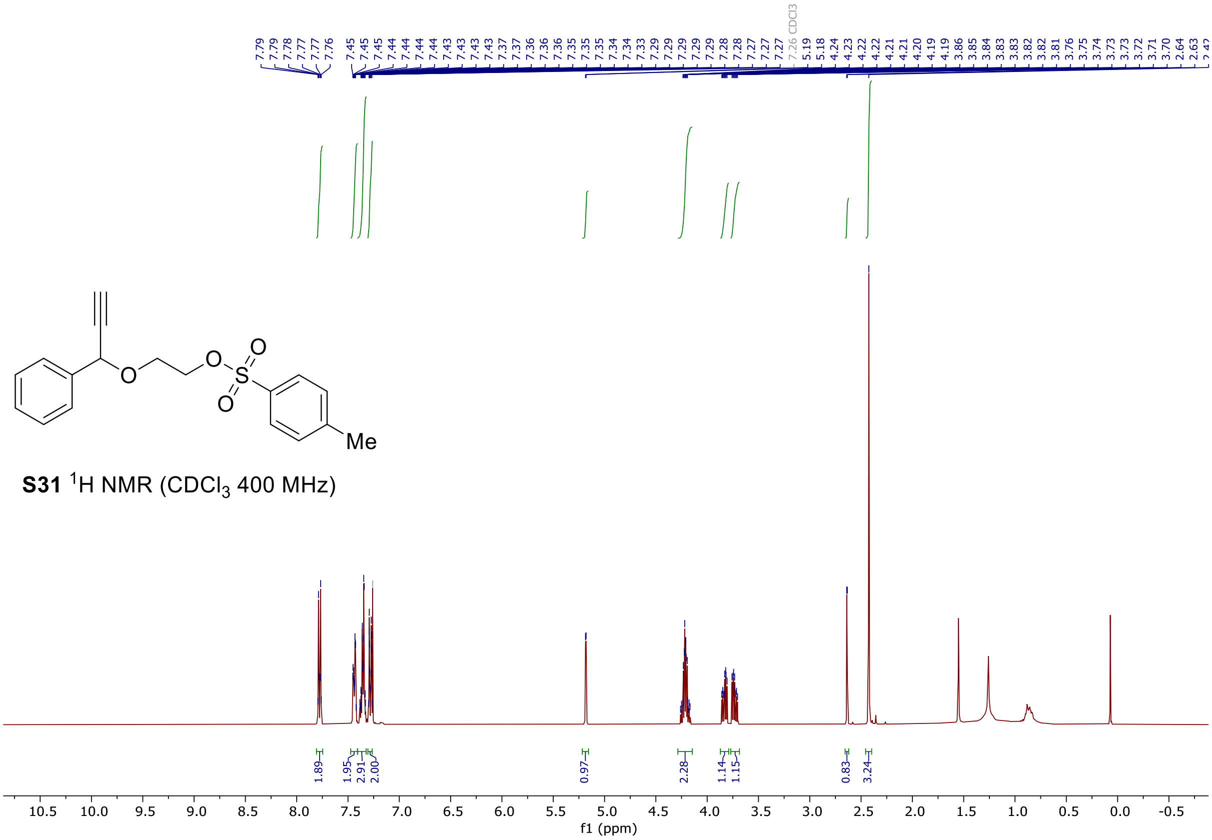

S31 ^{1}H NMR ($CDCl_3$ 400 MHz)

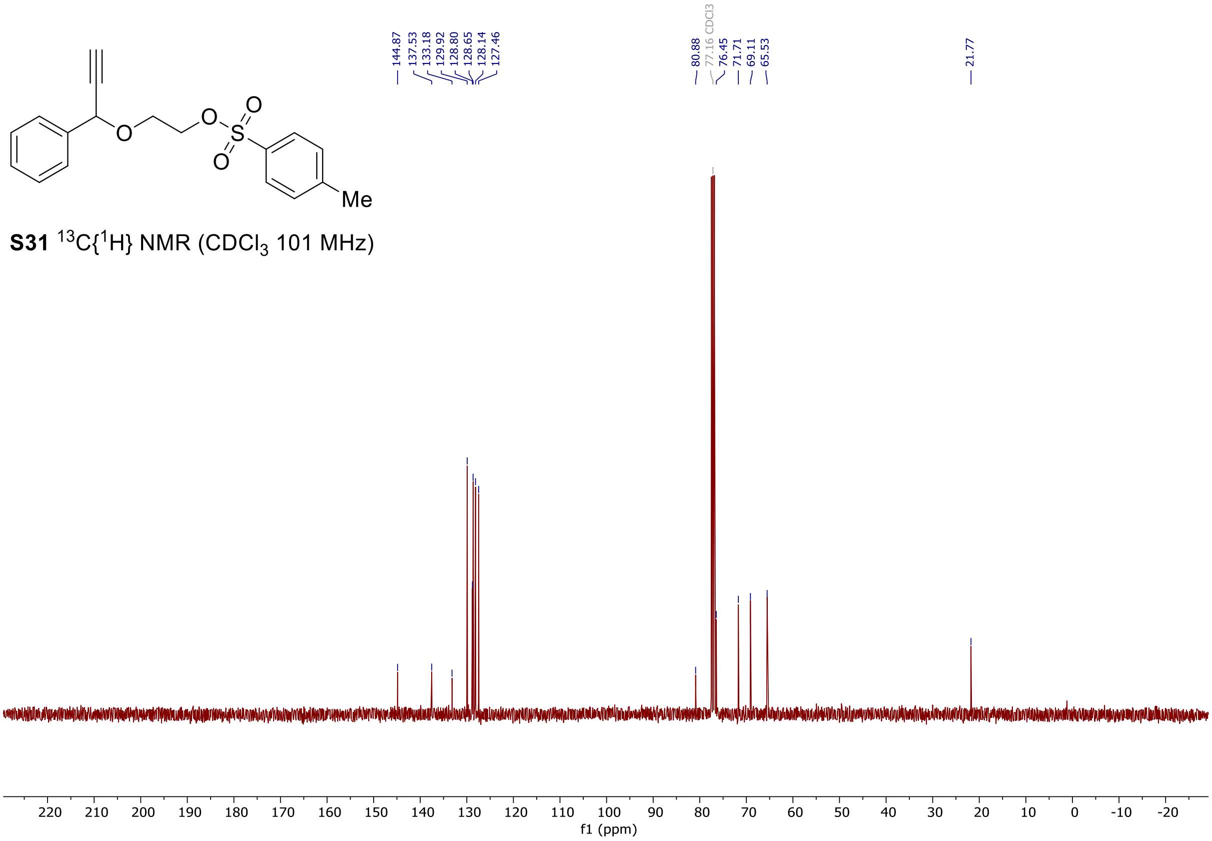

S31 ^{13}C{^{1}H} NMR ($CDCl_3$ 101 MHz)

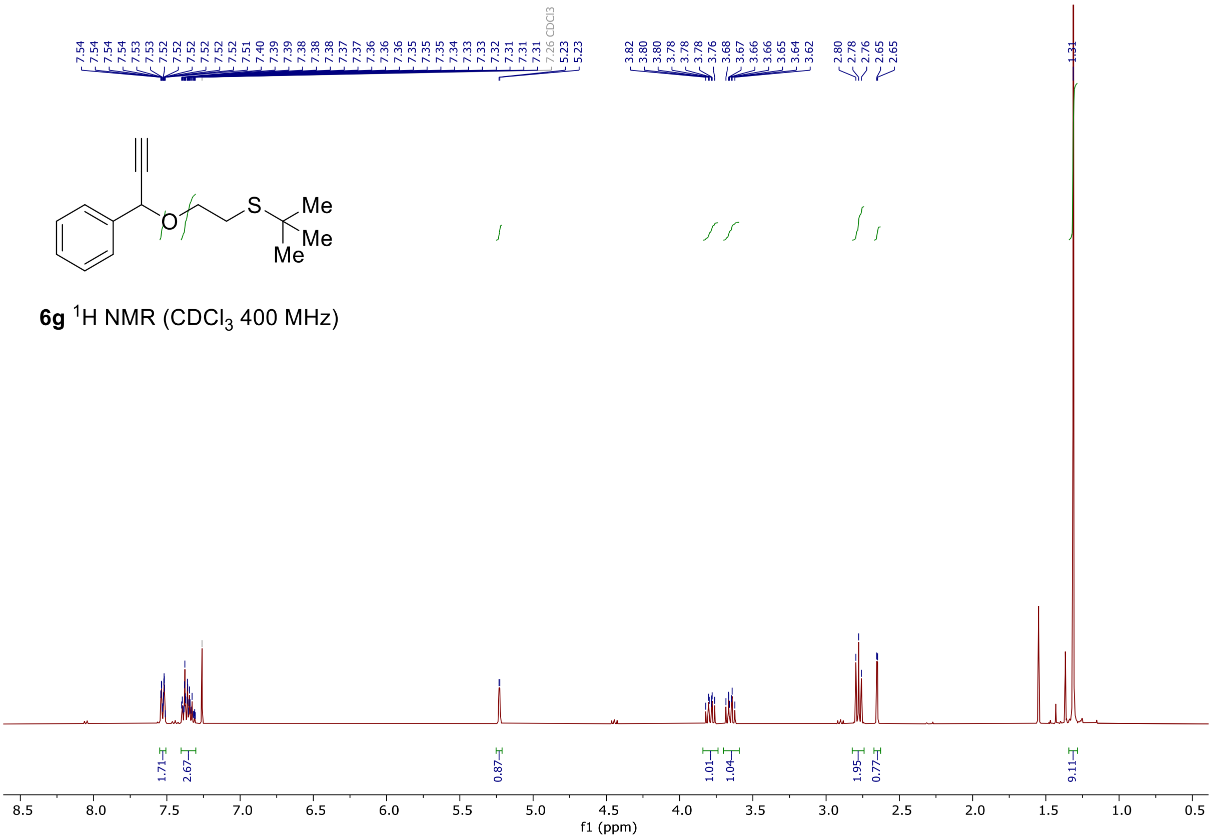

6g ^{1}H NMR ($CDCl_3$ 400 MHz)

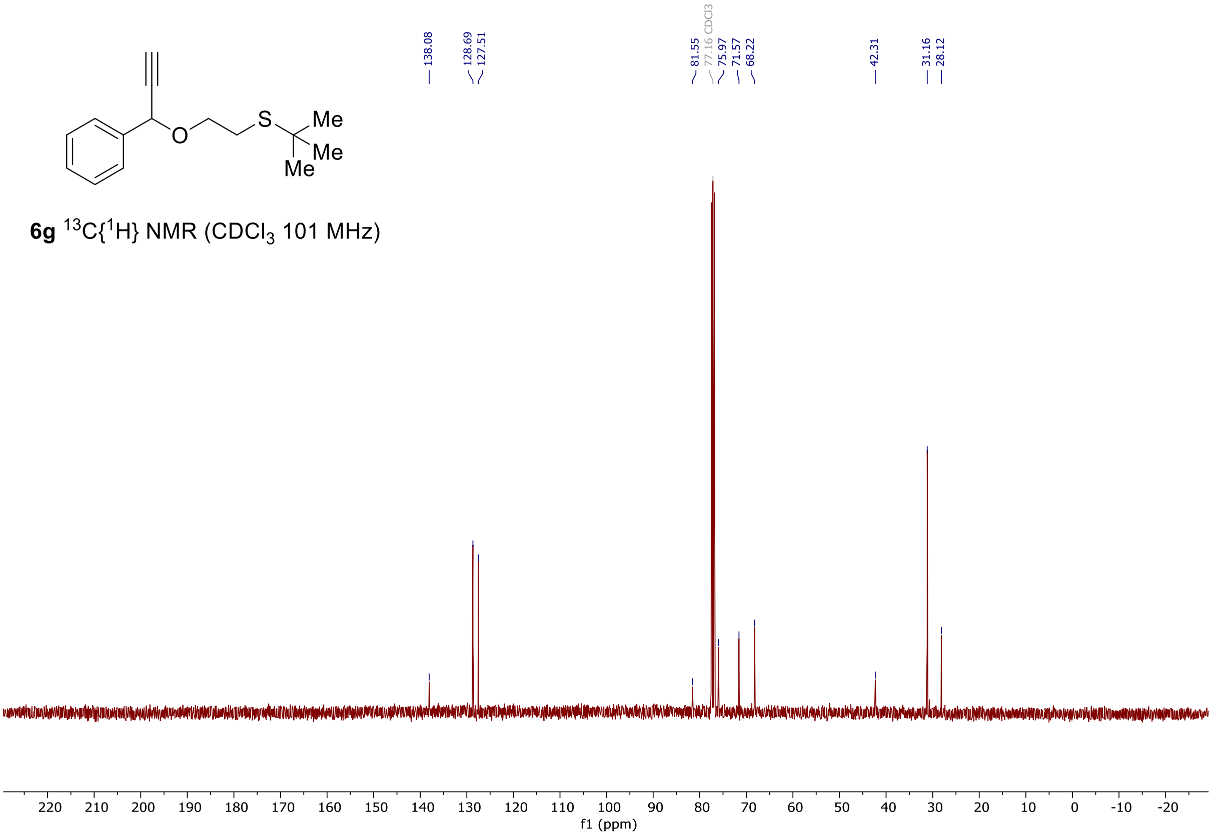

6g $^{13}C\{^{1}H\}$ NMR ($CDCl_3$ 101 MHz)

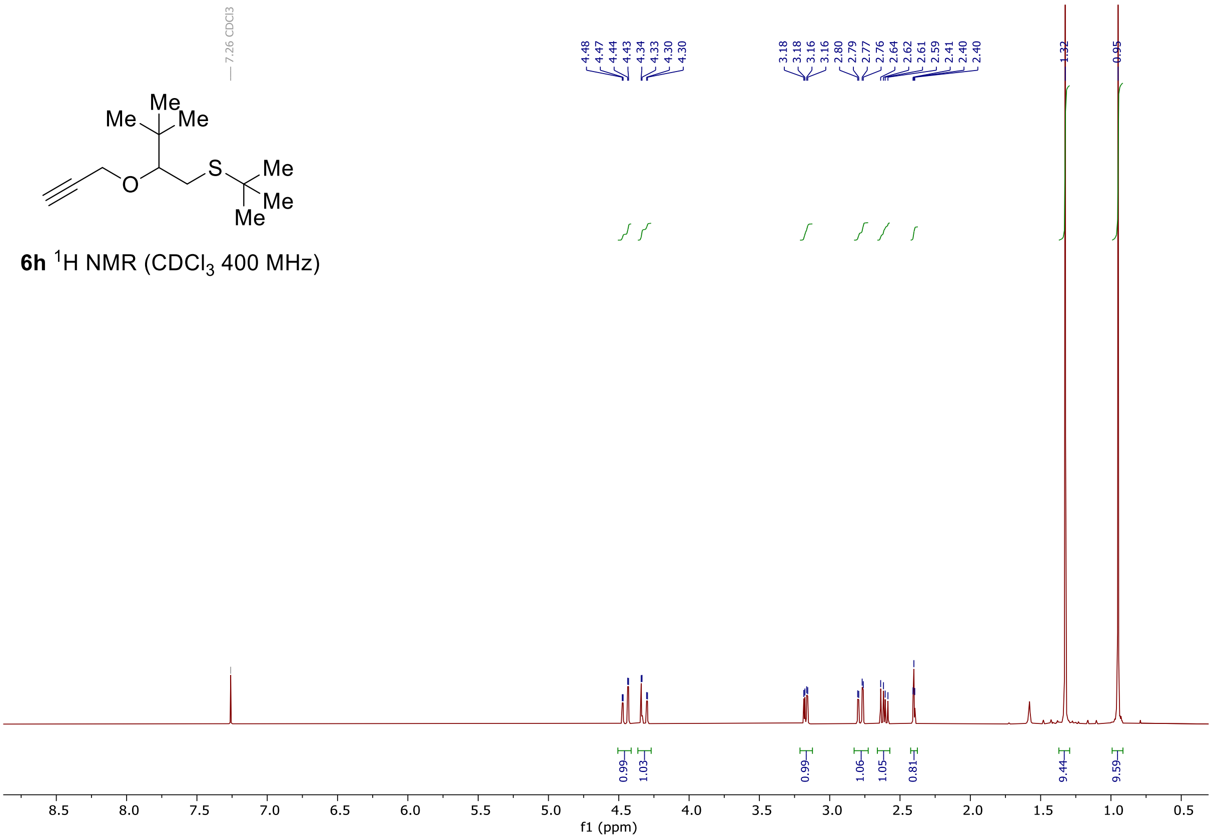

6h ¹H NMR (CDCl3 400 MHz)
7.26 CDCl3
4.48 4.47 4.44 4.43 4.34 4.33 4.30 4.30
3.18 3.18 3.16 3.16 2.80 2.79 2.77 2.76 2.64 2.62 2.61 2.59 2.41 2.40 2.40
1.32
0.95
0.99 1.03 0.99 1.06 1.05 0.81 9.44 9.59
8.5 8.0 7.5 7.0 6.5 6.0 5.5 5.0 4.5 4.0 3.5 3.0 2.5 2.0 1.5 1.0 0.5
f1 (ppm)

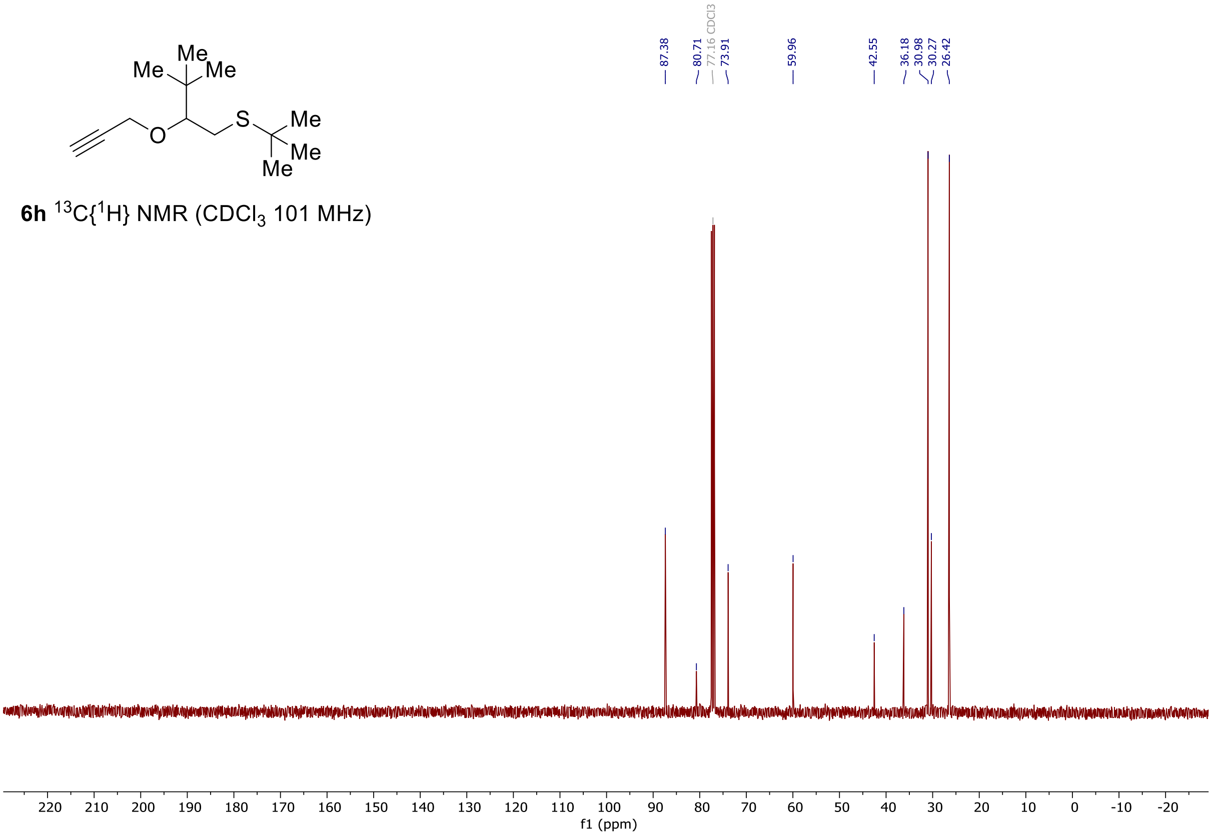

6h ¹³C{¹H} NMR (CDCl3 101 MHz)
87.38
80.71
77.16 CDCl3
73.91
59.96
42.55
36.18
30.98
30.27
26.42
220 210 200 190 180 170 160 150 140 130 120 110 100 90 80 70 60 50 40 30 20 10 0 -10 -20
f1 (ppm)

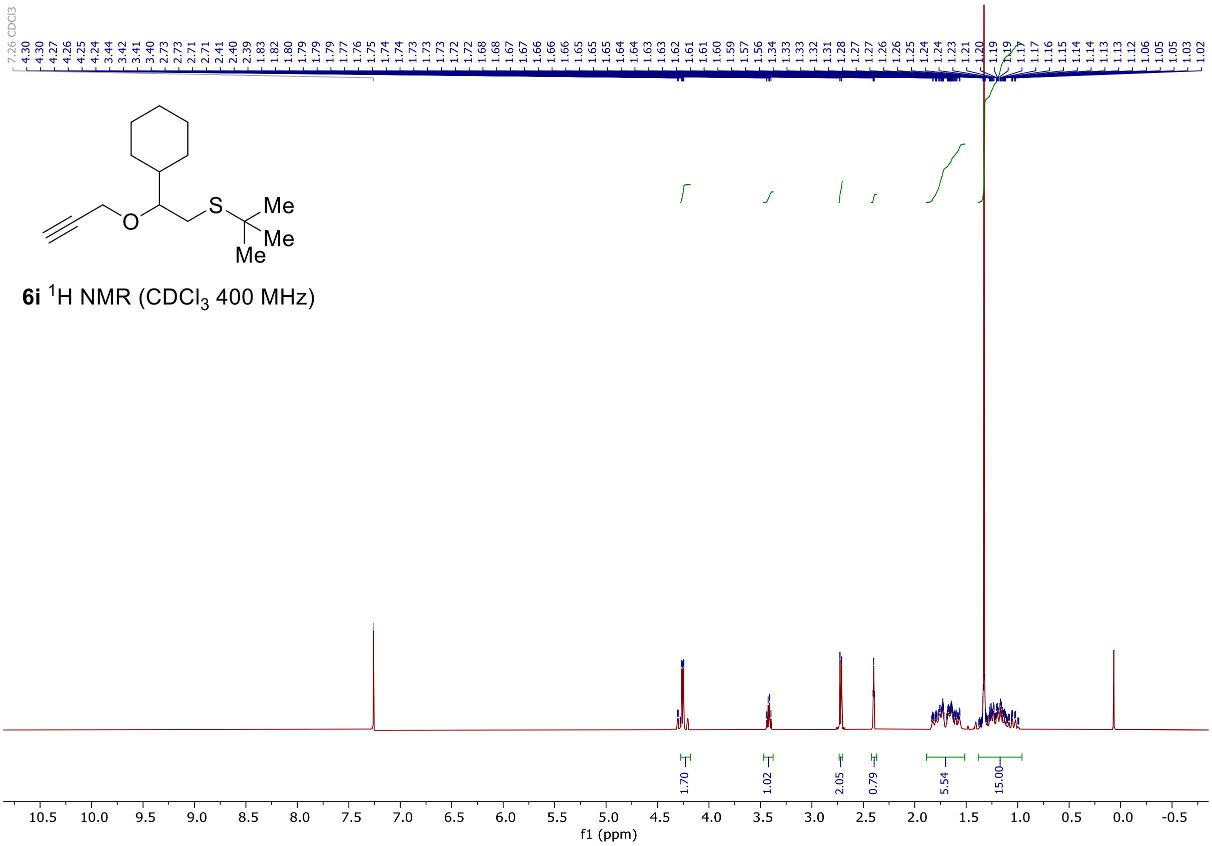

6i ^{1}H NMR ($CDCl_3$ 400 MHz)

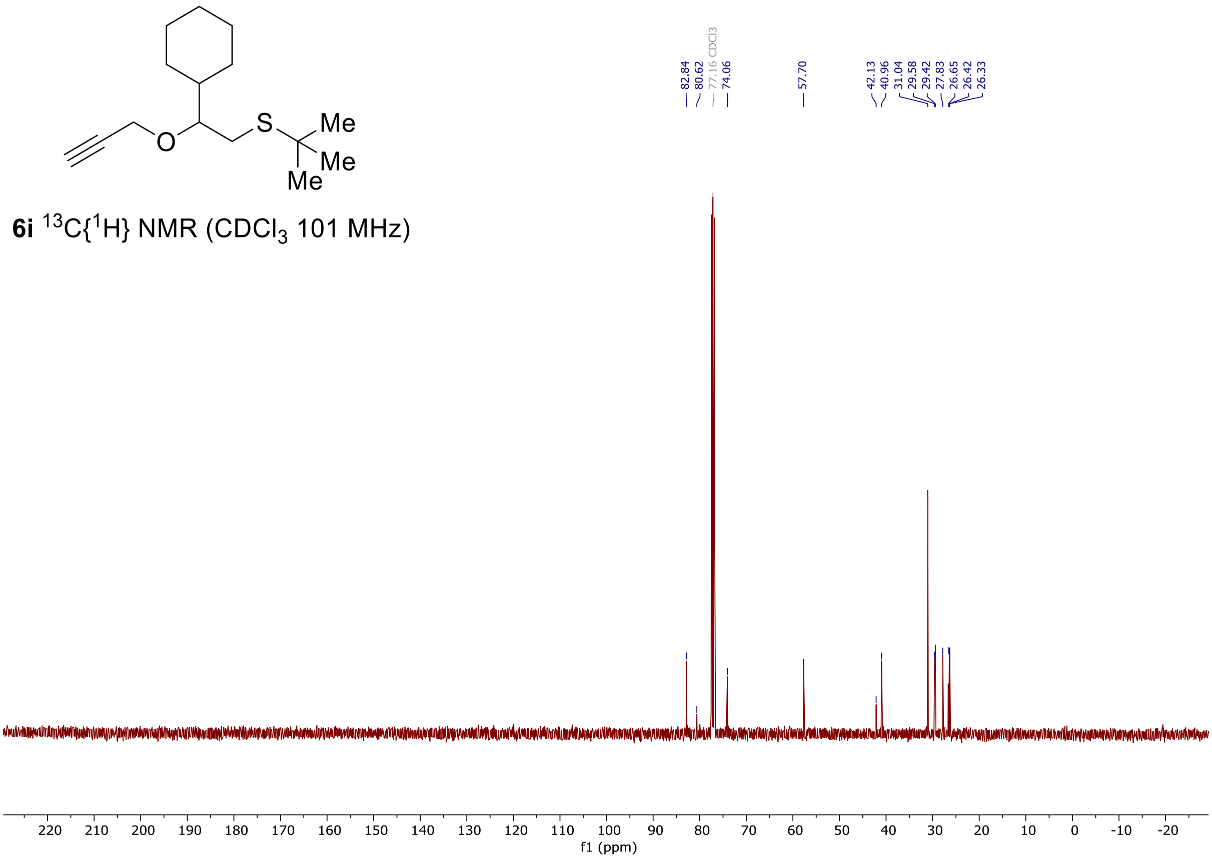

6i $^{13}C\{^1H\}$ NMR ($CDCl_3$ 101 MHz)

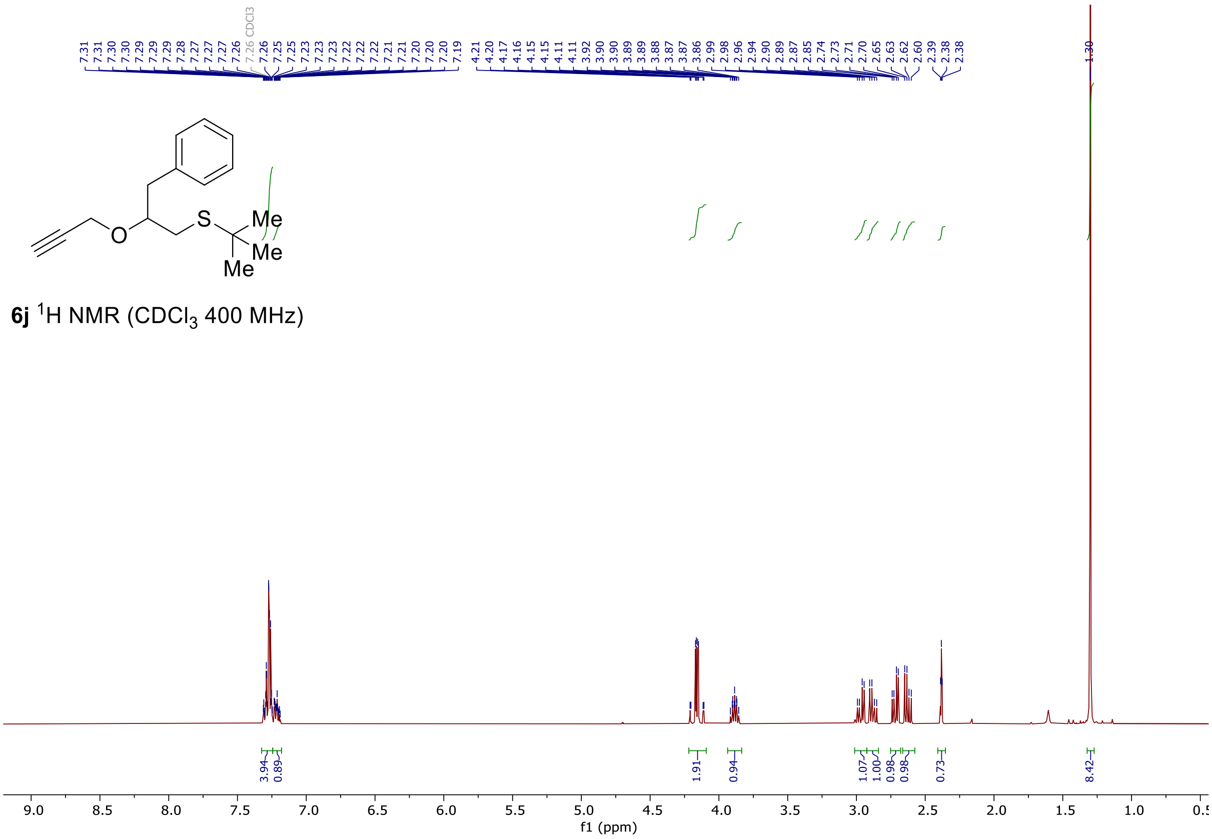

6j ^{1}H NMR ($CDCl_3$ 400 MHz)

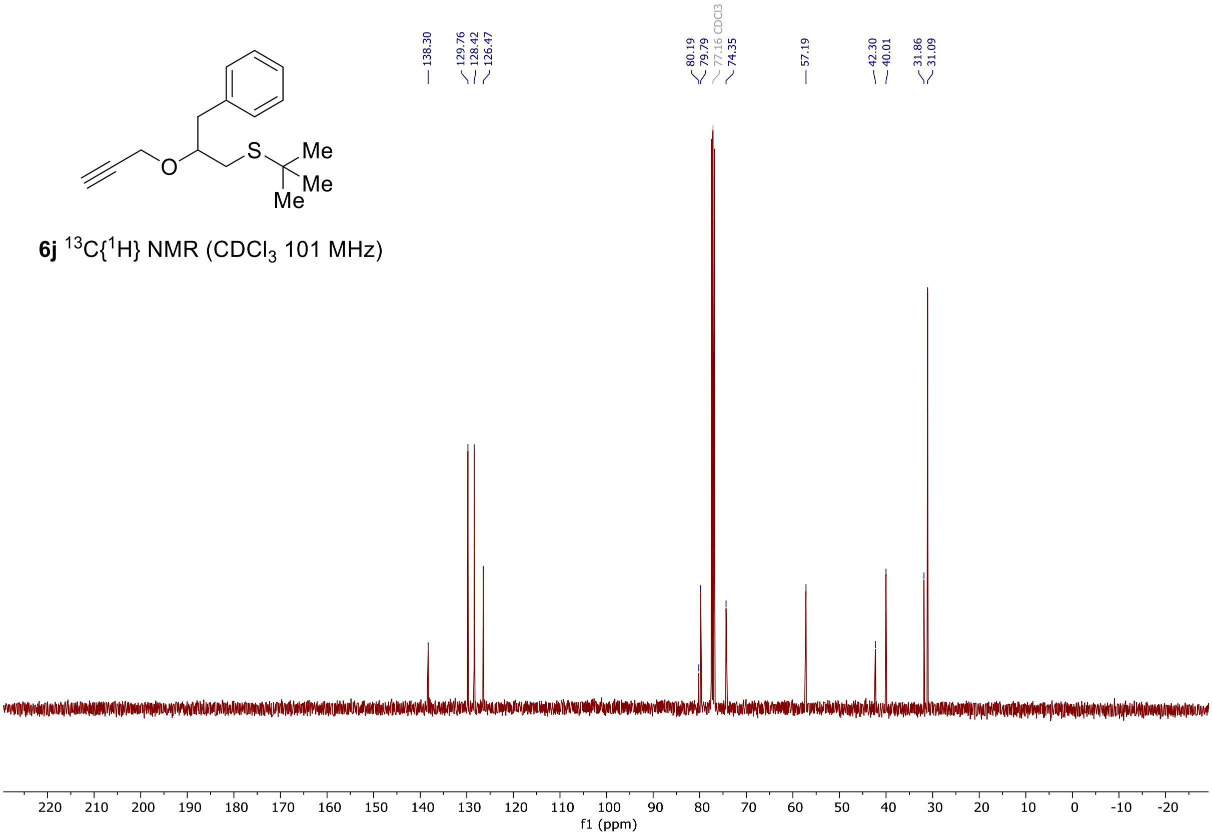

6j ^{13}C{^{1}H} NMR ($CDCl_3$ 101 MHz)

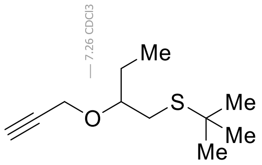

6k ¹H NMR (CDCl$_3$ 400 MHz)

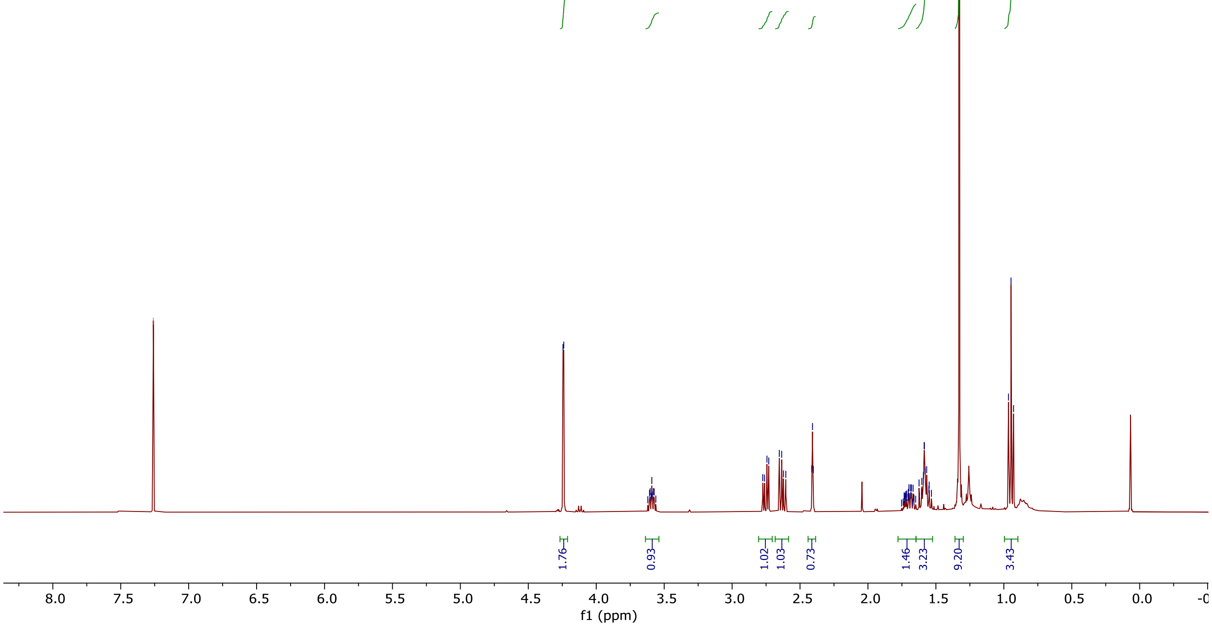

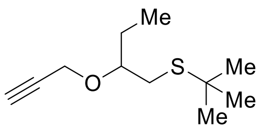

6k $^{13}C\{^1H\}$ NMR (CDCl$_3$ 101 MHz)

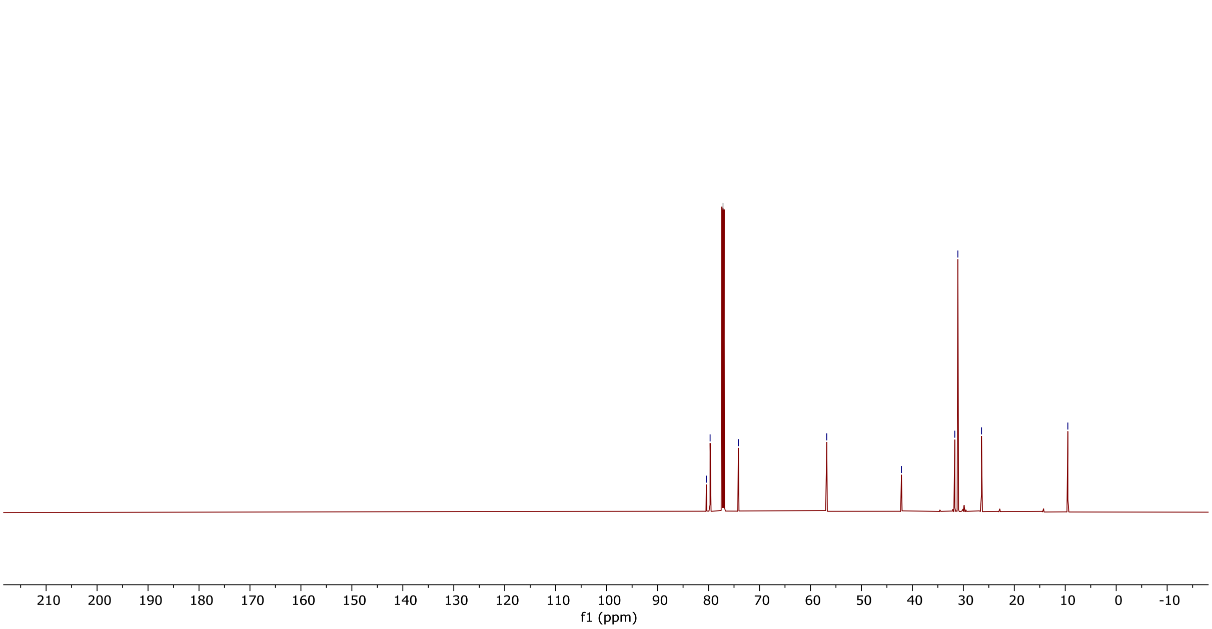

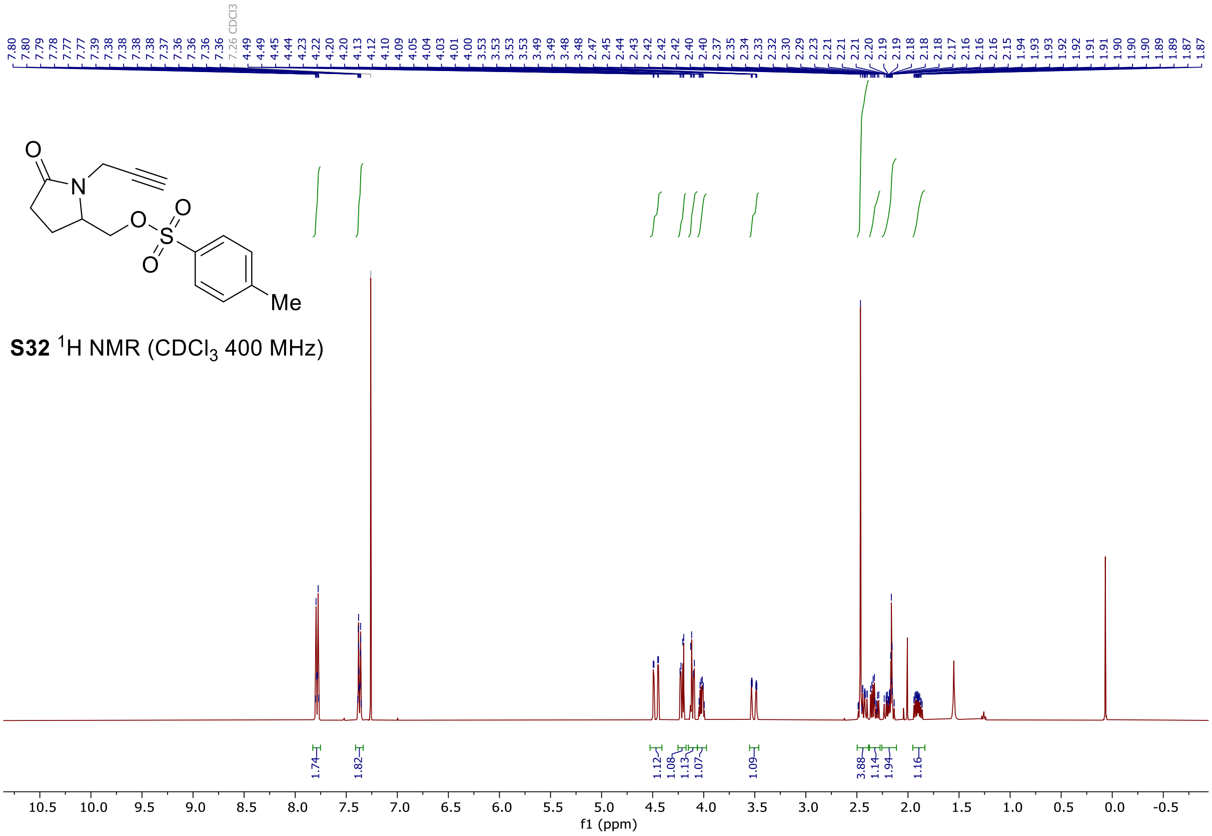

7.80 7.80 7.79 7.78 7.77 7.77 7.39 7.38 7.38 7.38 7.38 7.37 7.36 7.36 7.36 7.36 7.26 CDCl3 4.49 4.49 4.45 4.44 4.23 4.22 4.20 4.20 4.13 4.12 4.10 4.09 4.05 4.04 4.03 4.01 4.00 3.53 3.53 3.53 3.53 3.49 3.49 3.48 3.48 2.47 2.45 2.44 2.43 2.42 2.42 2.42 2.40 2.40 2.37 2.35 2.34 2.33 2.32 2.30 2.29 2.23 2.21 2.21 2.21 2.20 2.19 2.19 2.18 2.18 2.18 2.17 2.16 2.16 2.16 2.15 1.94 1.93 1.93 1.92 1.92 1.91 1.91 1.90 1.90 1.89 1.87 1.87
O
N
O
S
O
O
Me
S32 1H NMR (CDCl3 400 MHz)
1.74 1.82 1.12 1.08 1.13 1.07 1.09 3.88 1.14 1.94 1.16
10.5 10.0 9.5 9.0 8.5 8.0 7.5 7.0 6.5 6.0 5.5 5.0 4.5 4.0 3.5 3.0 2.5 2.0 1.5 1.0 0.5 0.0 -0.5
f1 (ppm)

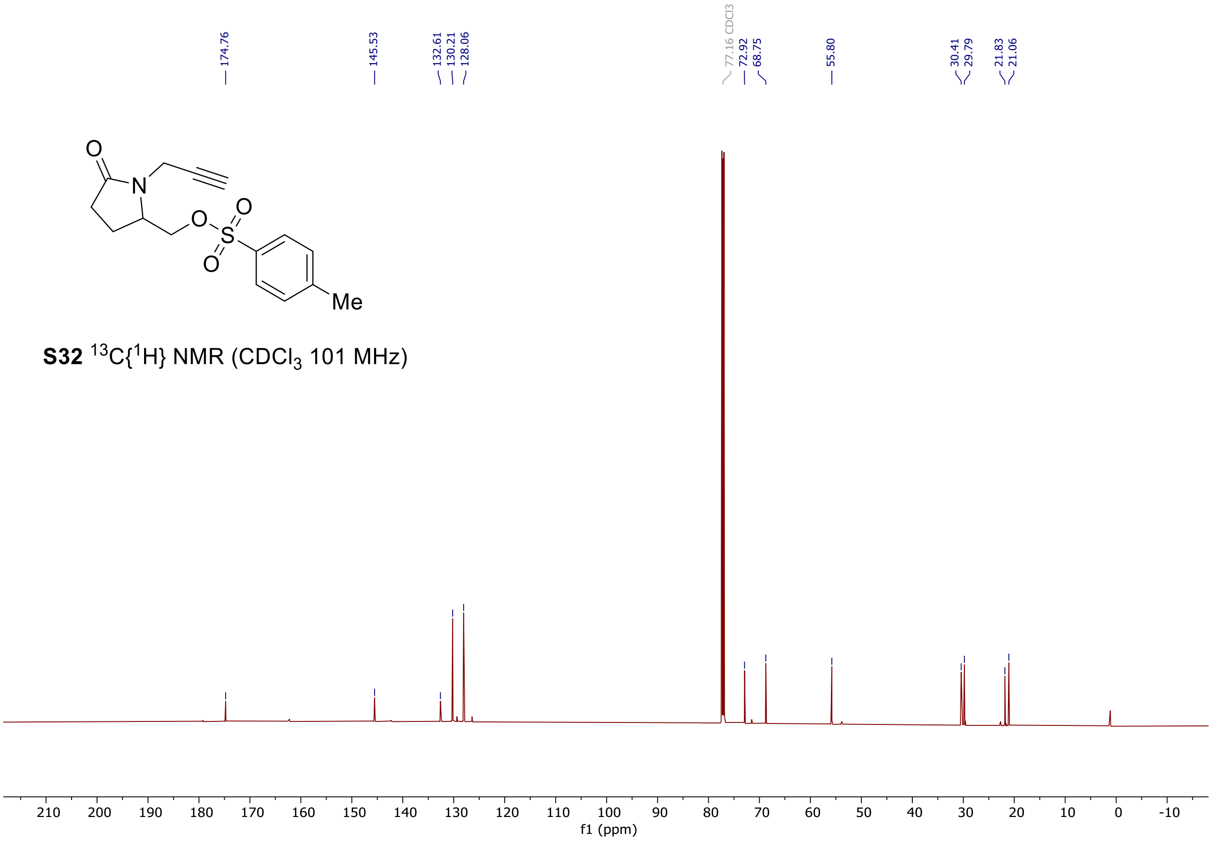

174.76 145.53 132.61 130.21 128.06 77.16 CDCl3 72.92 68.75 55.80 30.41 29.79 21.83 21.06
O
N
O
S
O
O
Me
S32 13C{1H} NMR (CDCl3 101 MHz)
210 200 190 180 170 160 150 140 130 120 110 100 90 80 70 60 50 40 30 20 10 0 -10
f1 (ppm)

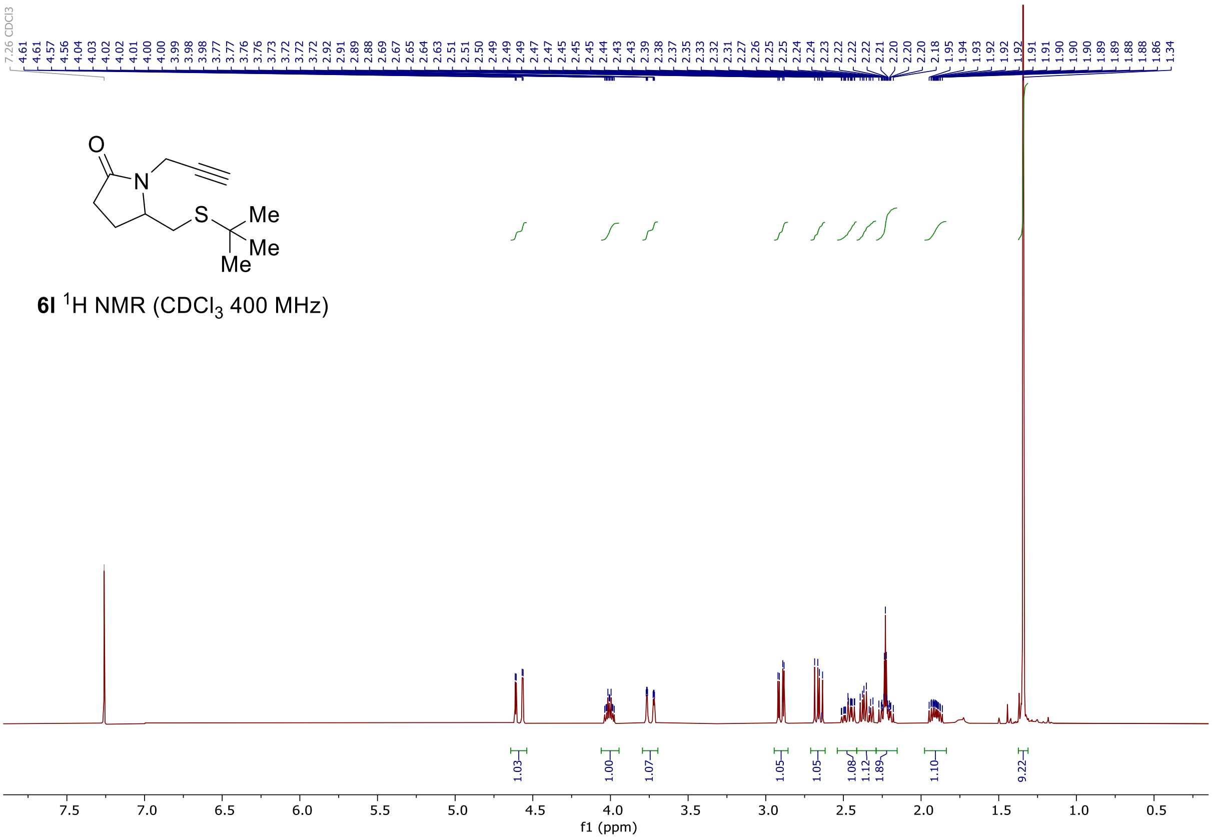

6l ^{1}H NMR ($CDCl_3$ 400 MHz)

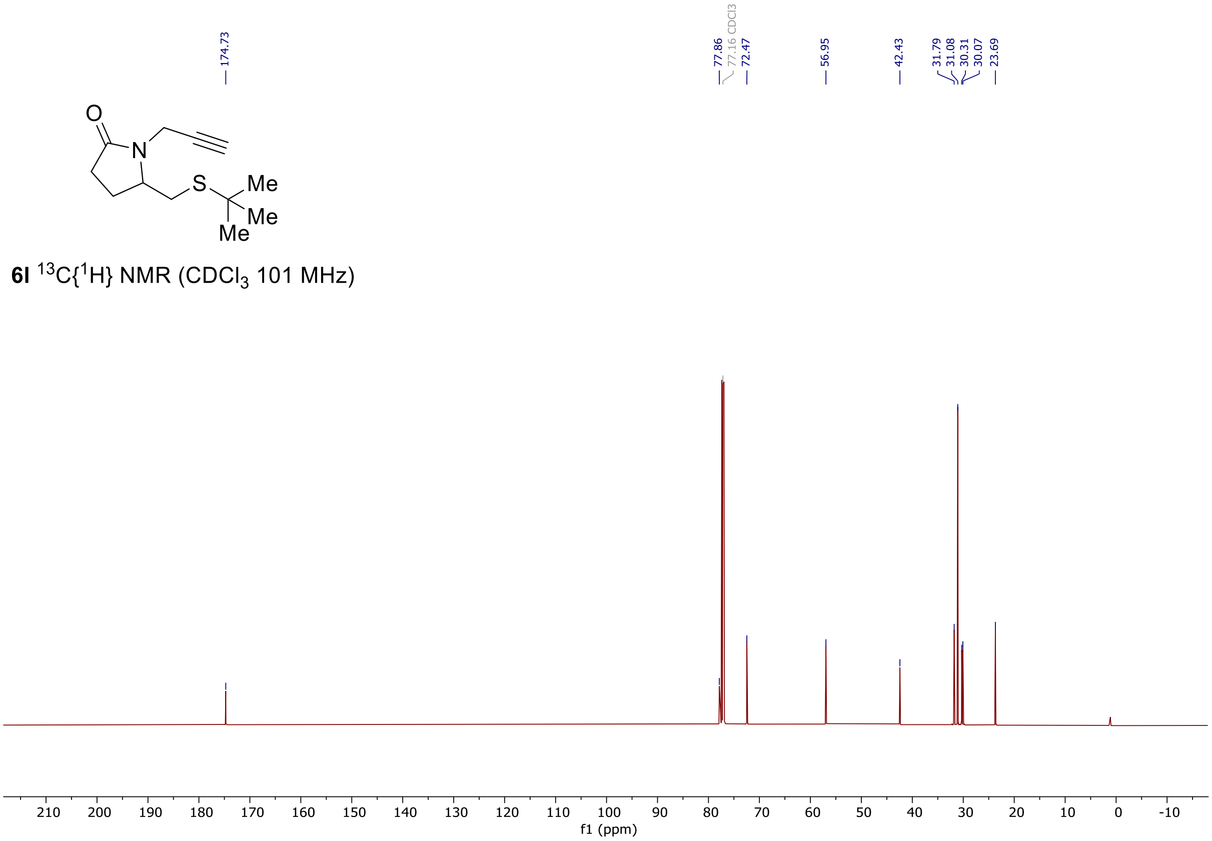

6l $^{13}C\{^{1}H\}$ NMR ($CDCl_3$ 101 MHz)

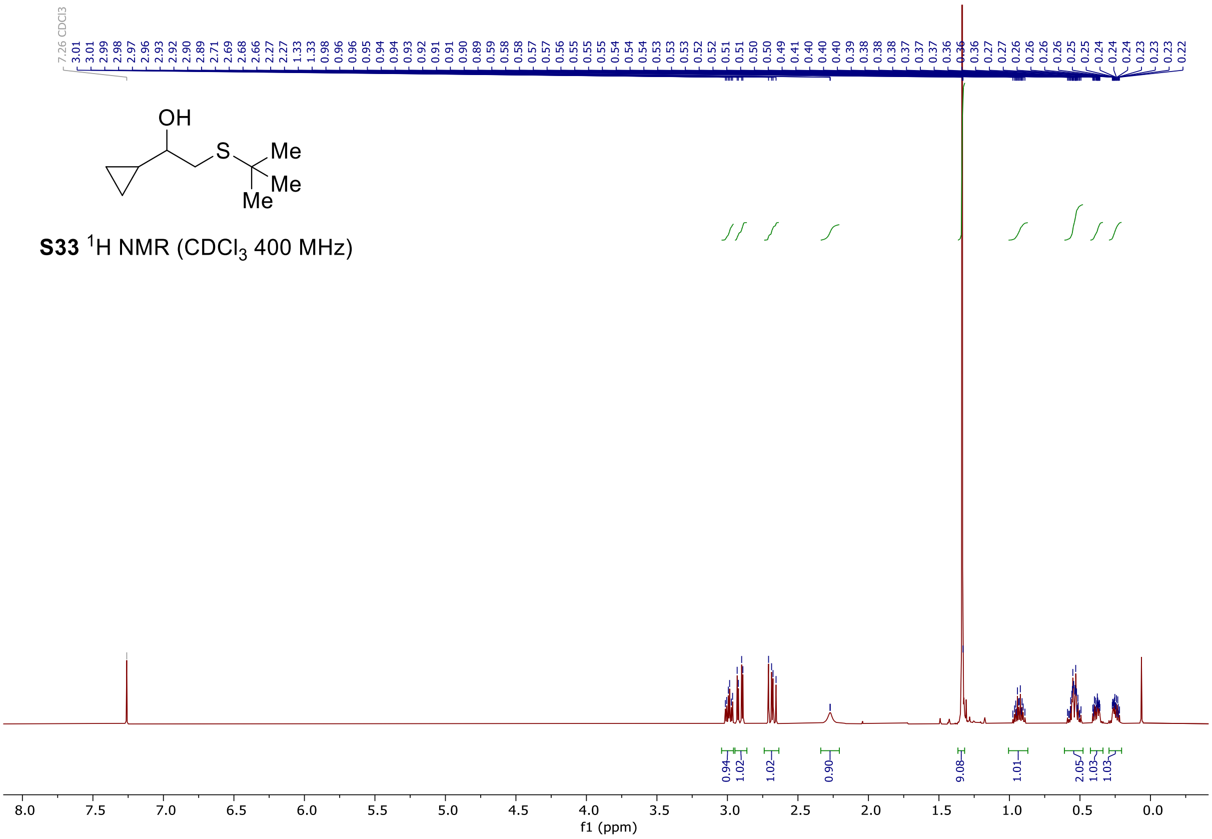

S33 ^{1}H NMR ($CDCl_3$ 400 MHz)

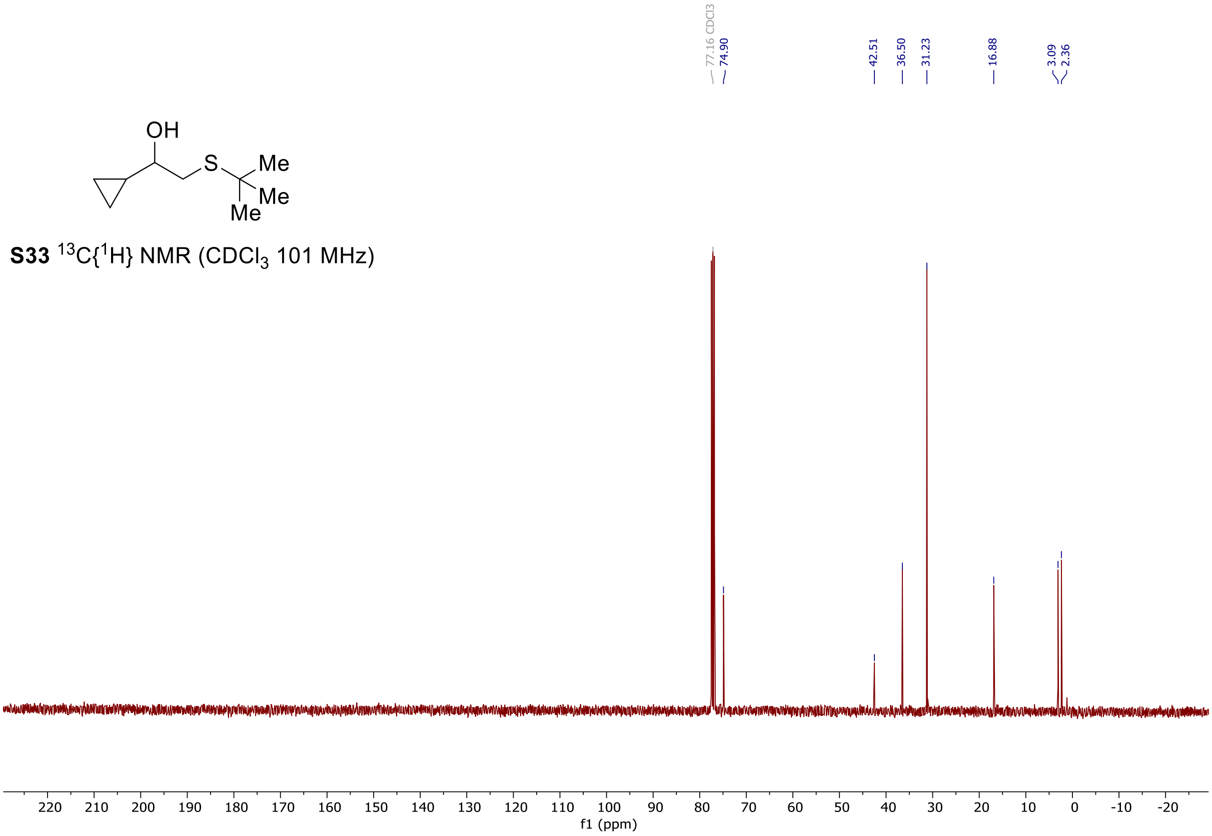

S33 $^{13}C\{^{1}H\}$ NMR ($CDCl_3$ 101 MHz)

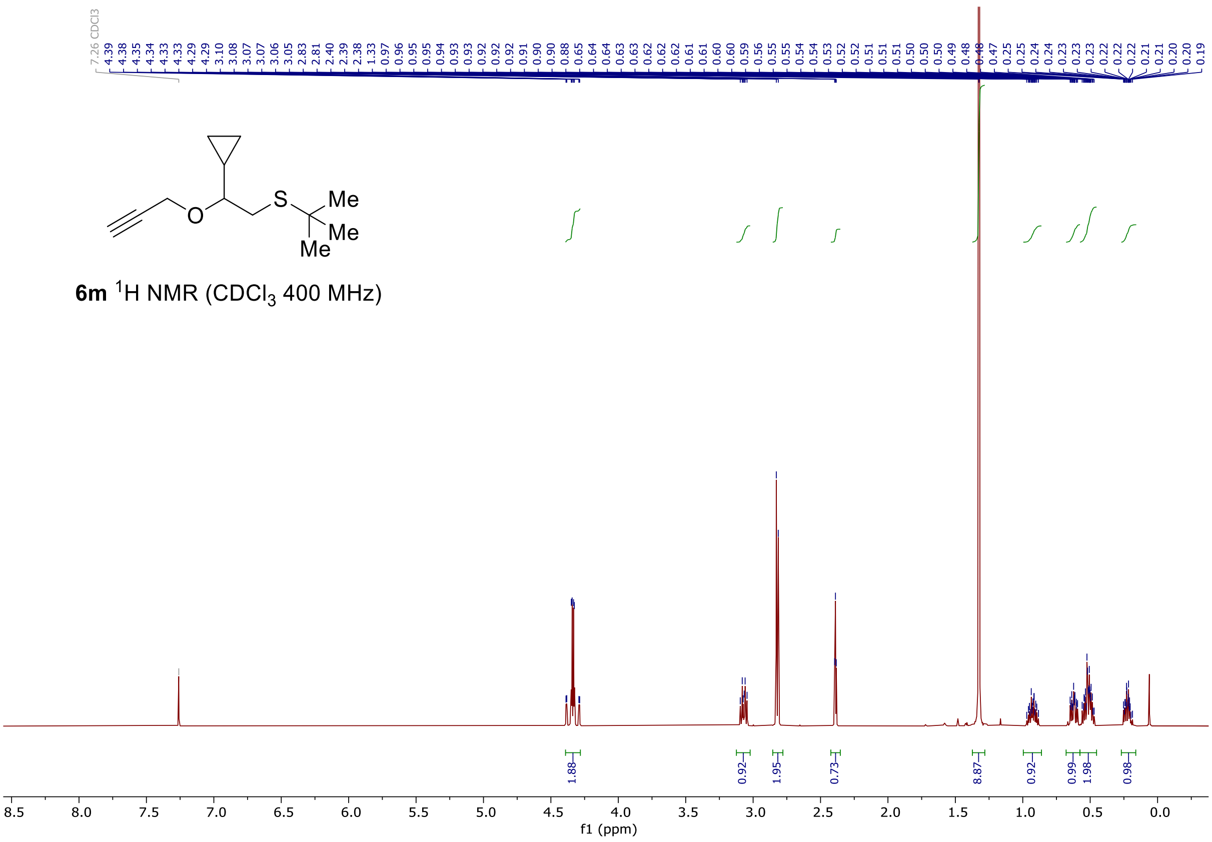

6m ^{1}H NMR ($CDCl_3$ 400 MHz)

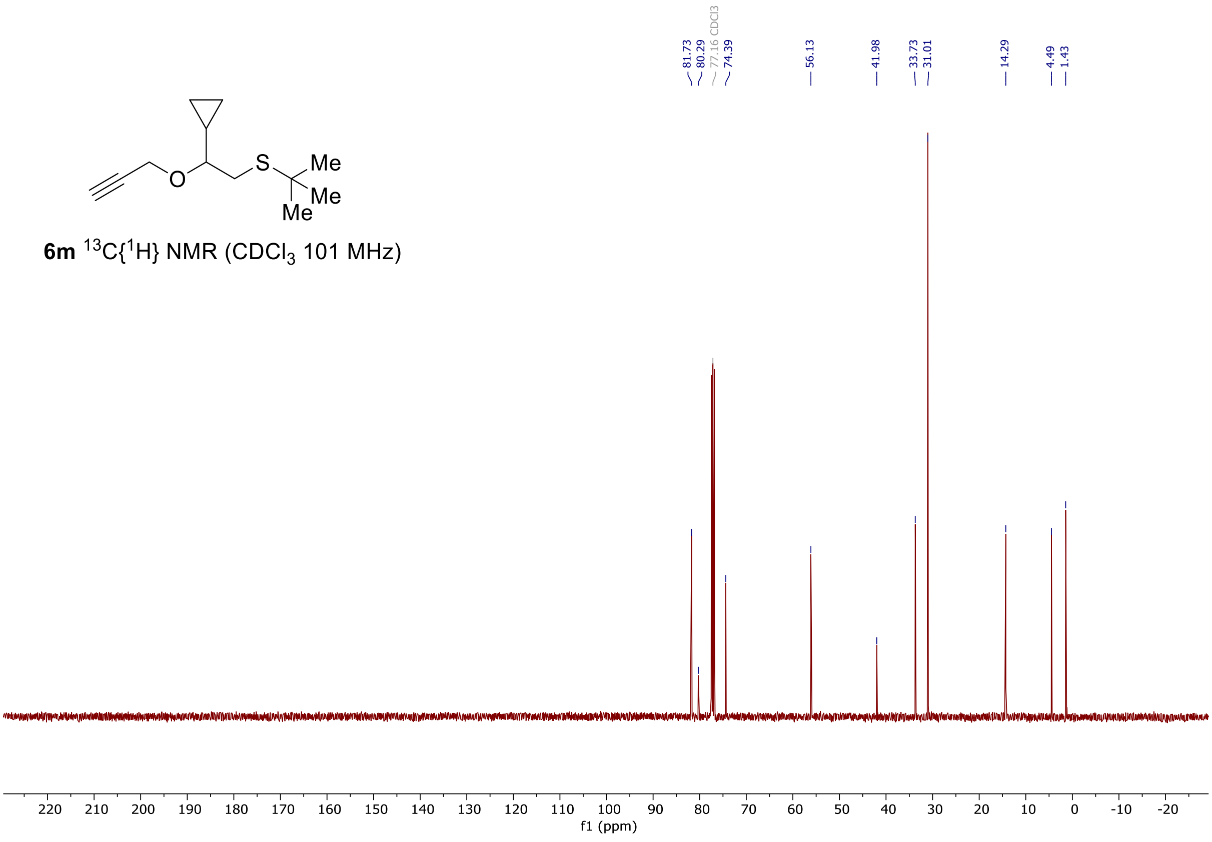

6m $^{13}C\{^1H\}$ NMR ($CDCl_3$ 101 MHz)

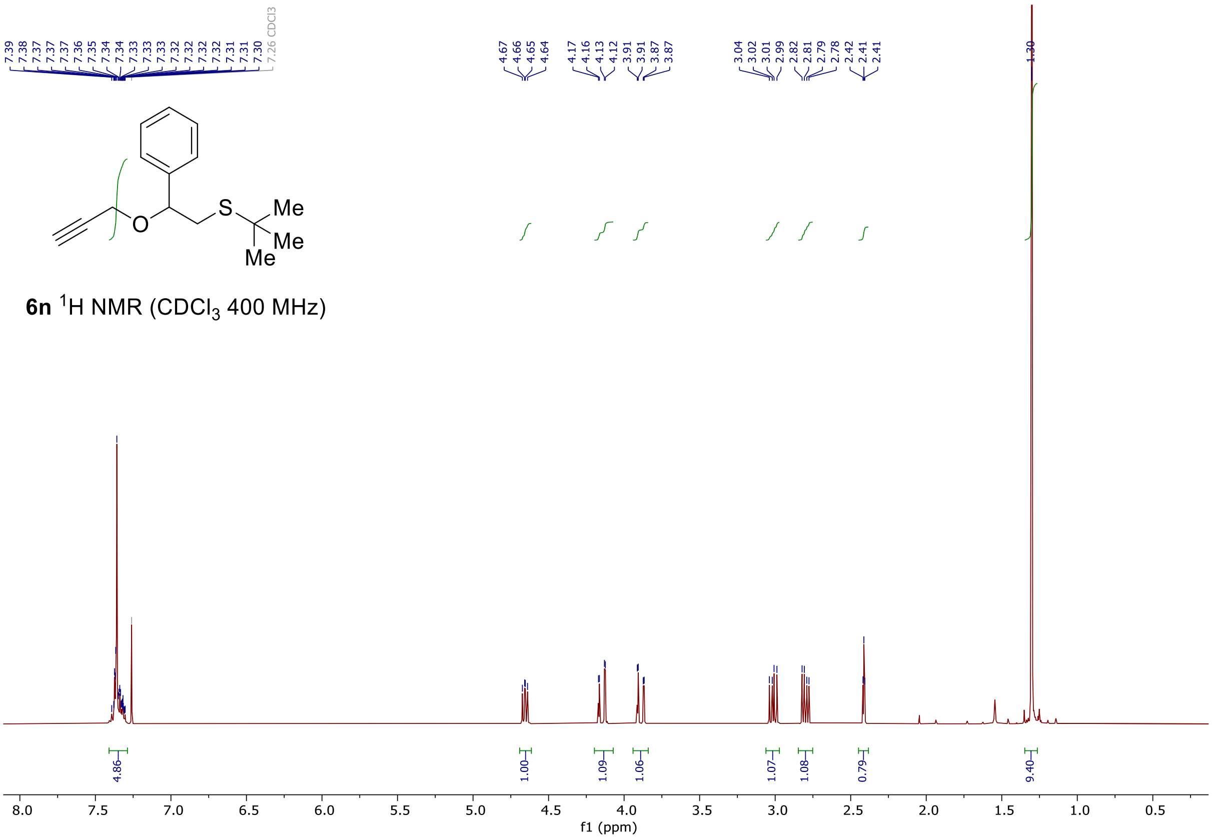

6n ^{1}H NMR ($CDCl_3$ 400 MHz)

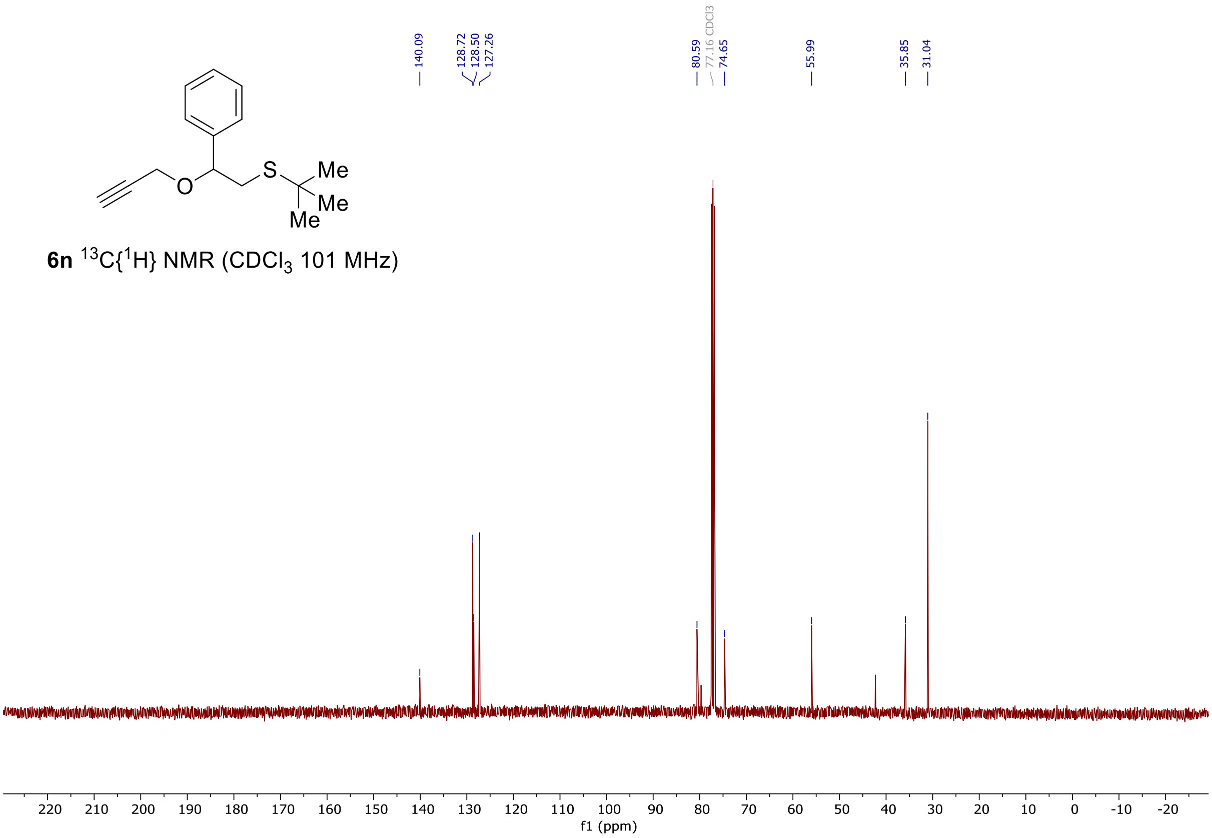

6n ^{13}C{^{1}H} NMR ($CDCl_3$ 101 MHz)

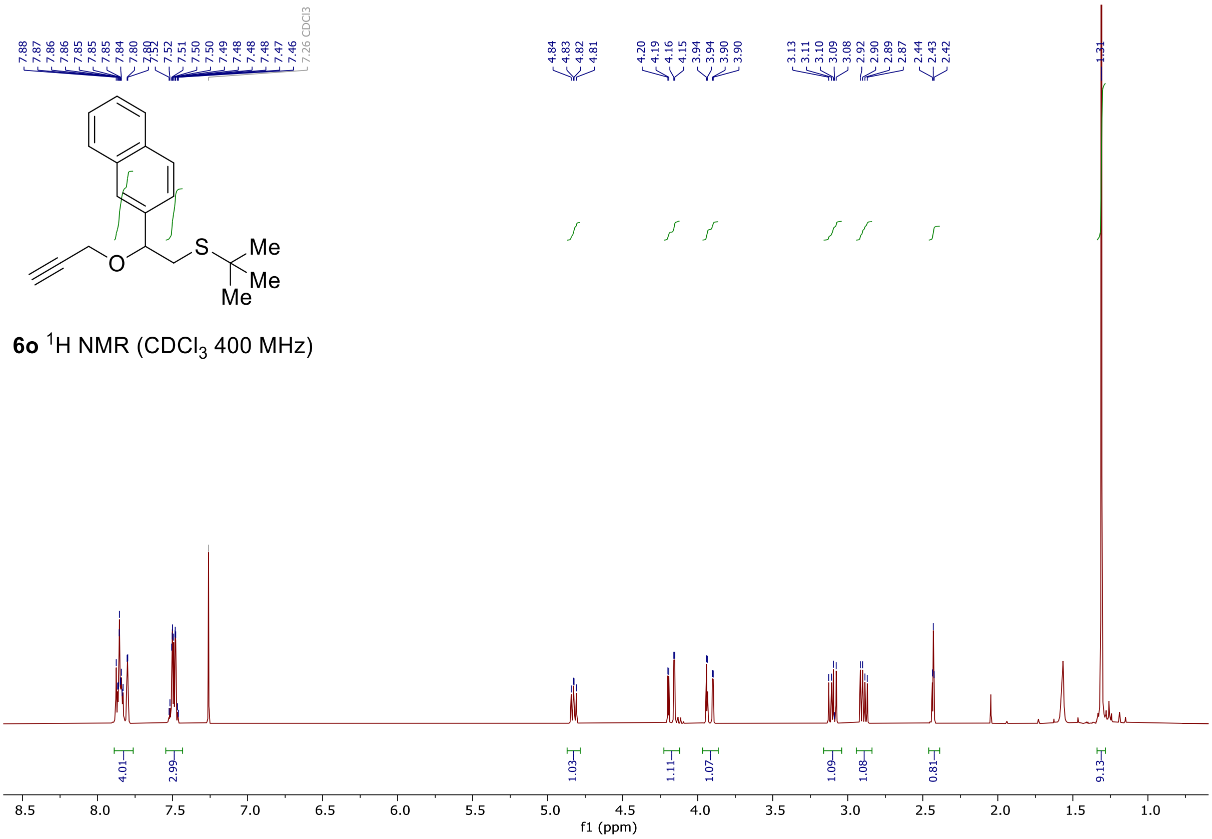

6o ^{1}H NMR (CDCl$_3$ 400 MHz)

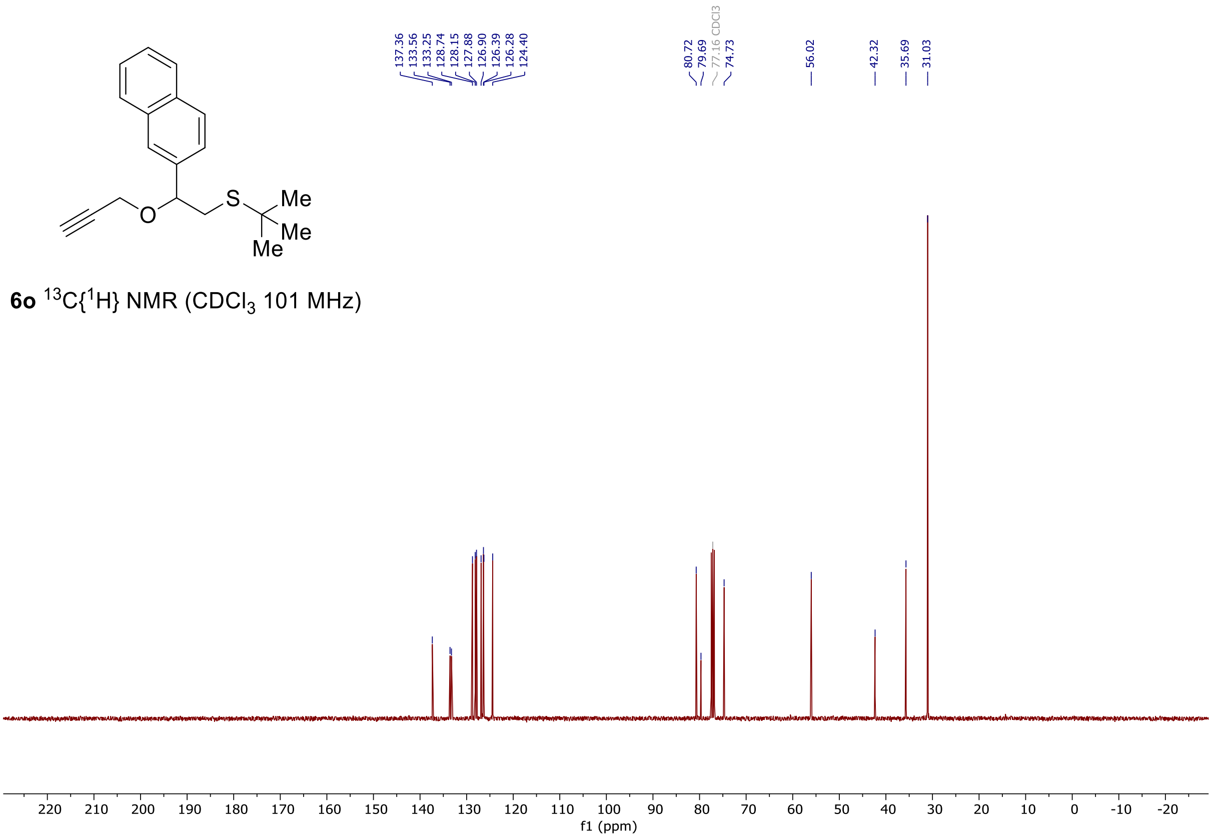

6o ^{13}C{^{1}H} NMR (CDCl$_3$ 101 MHz)

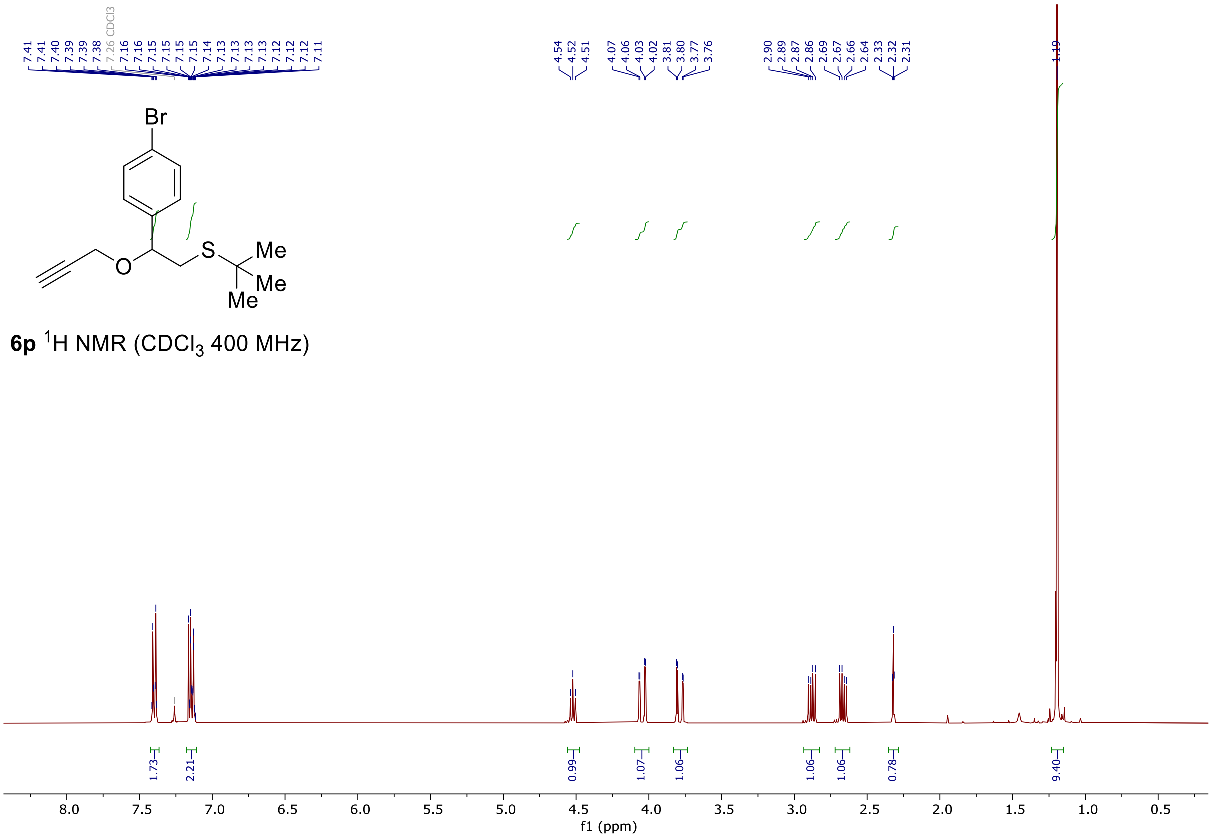

6p ^{1}H NMR ($CDCl_3$ 400 MHz)

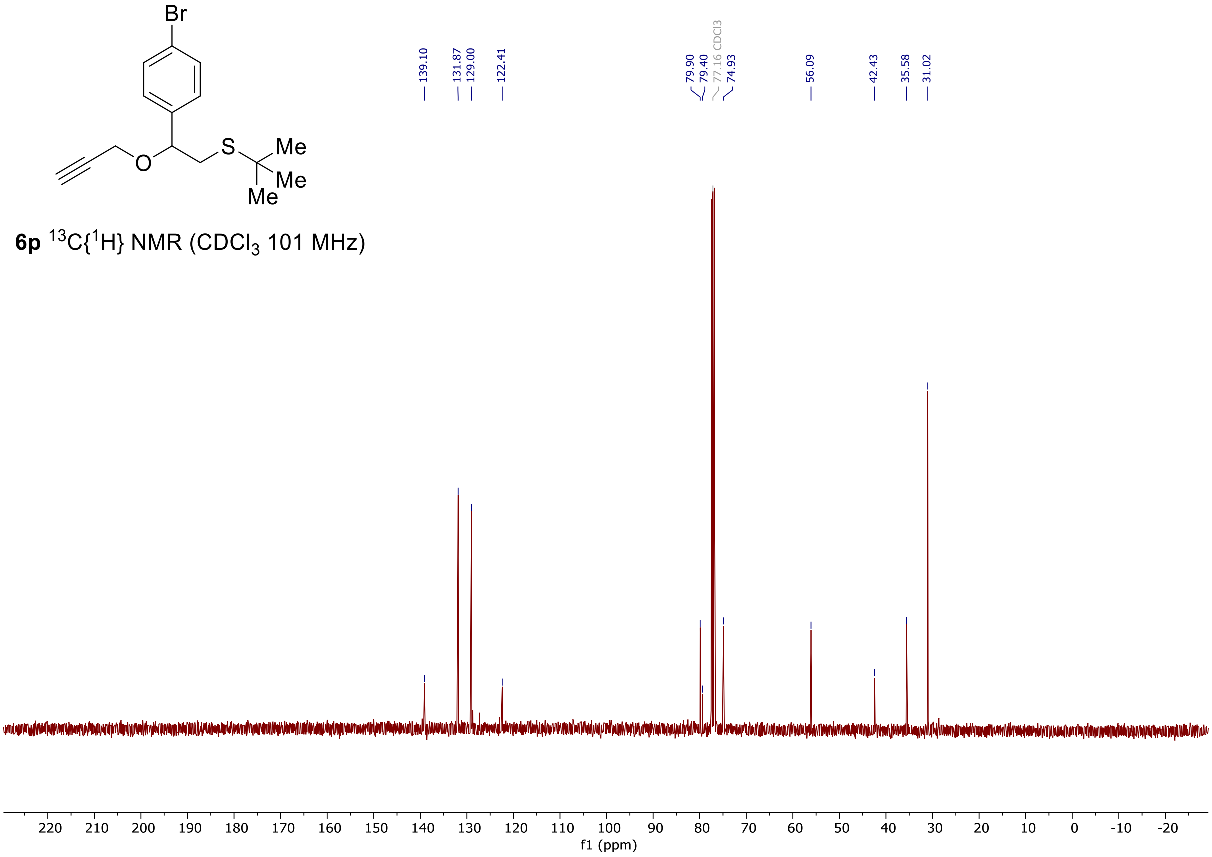

6p ^{13}C{^{1}H} NMR ($CDCl_3$ 101 MHz)

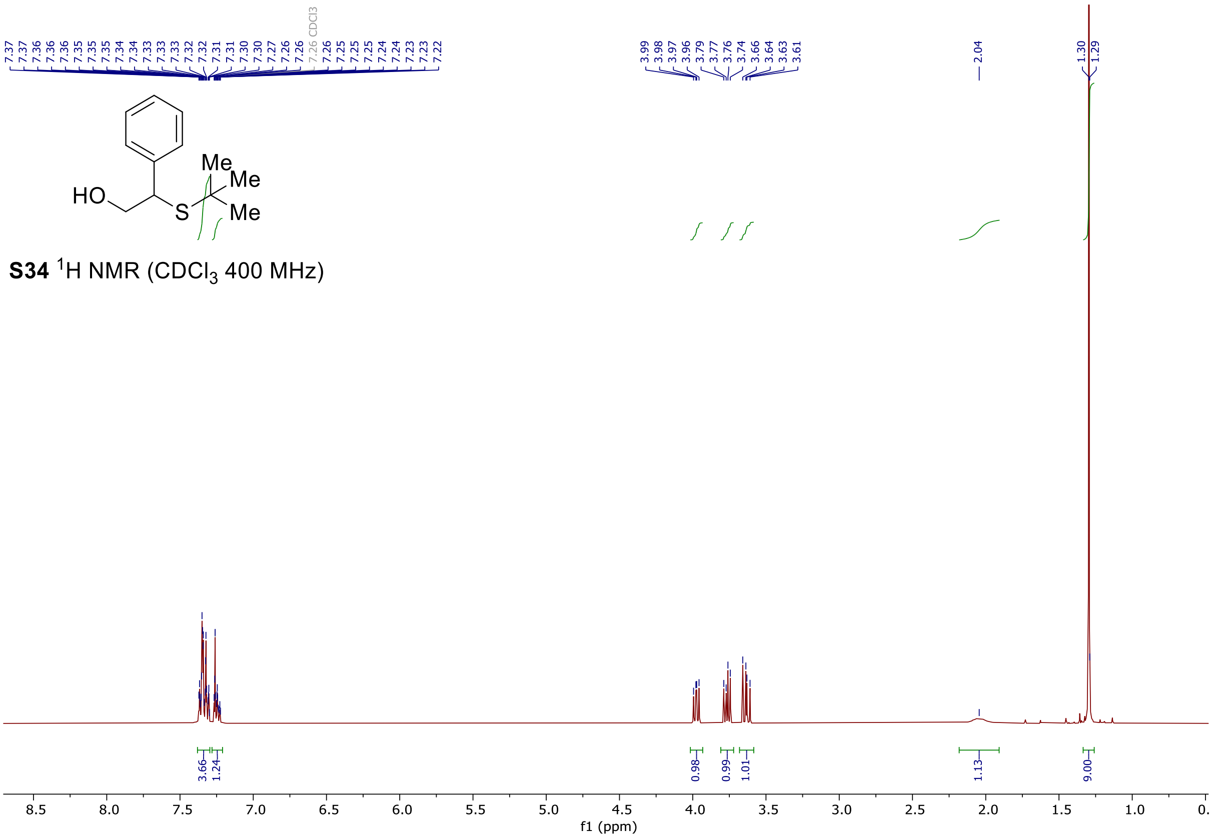

S34 ^{1}H NMR ($CDCl_3$ 400 MHz)

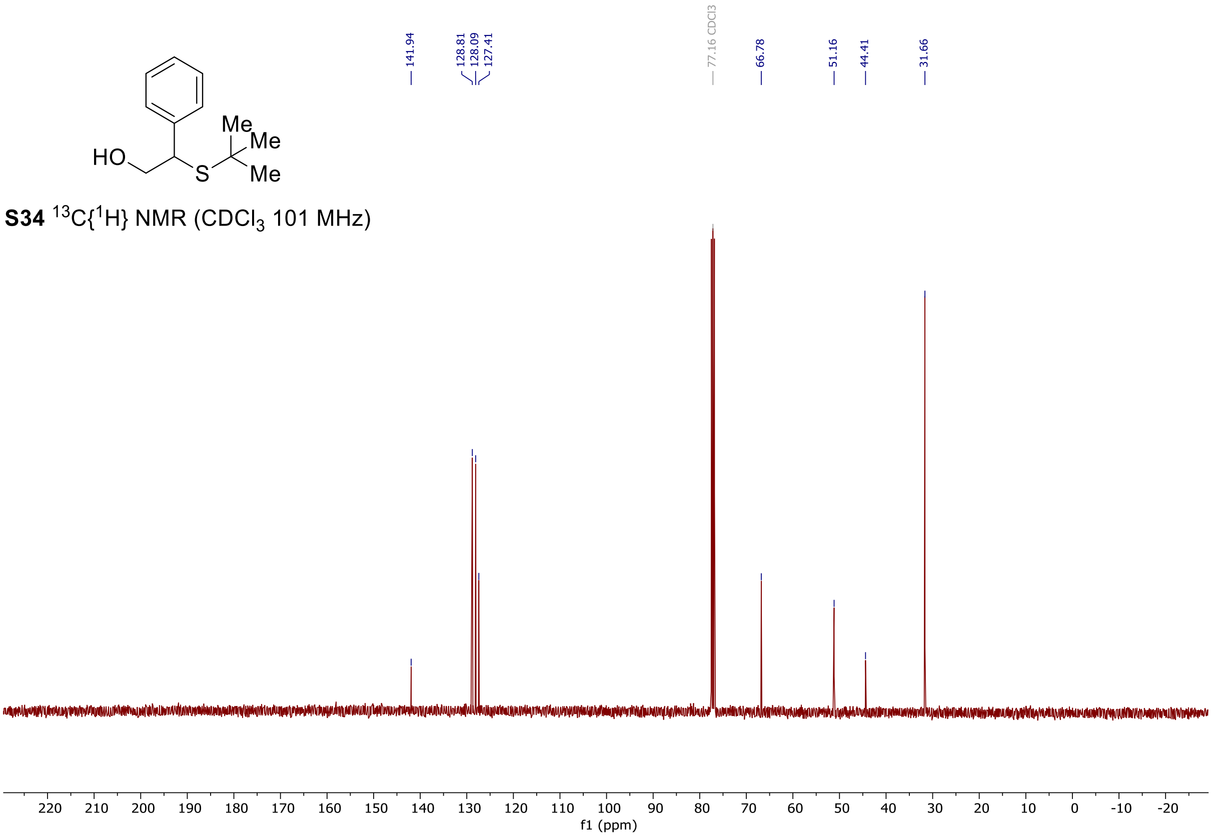

S34 $^{13}C\{^{1}H\}$ NMR ($CDCl_3$ 101 MHz)

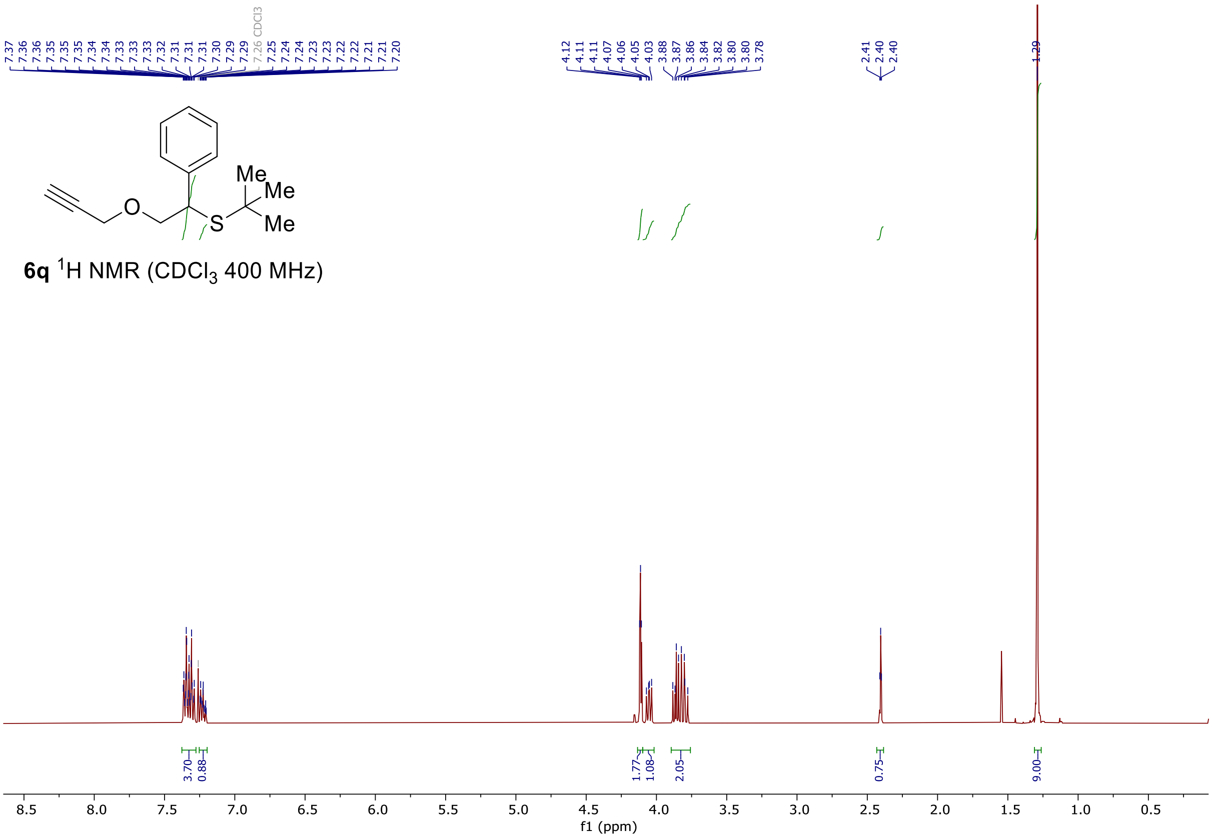
7.37 7.36 7.36 7.35 7.35 7.35 7.34 7.34 7.33 7.33 7.33 7.32 7.31 7.31 7.31 7.30 7.29 7.29 7.26 CDCl3 7.25 7.24 7.24 7.23 7.23 7.22 7.22 7.21 7.21 7.20
4.12 4.11 4.11 4.07 4.06 4.05 4.03 3.88 3.87 3.86 3.84 3.82 3.80 3.80 3.78
2.41 2.40 2.40
1.29
Me
Me
Me
O
S
6q ¹H NMR (CDCl3 400 MHz)
3.70 0.88 1.77 1.08 2.05 0.75 9.00
8.5 8.0 7.5 7.0 6.5 6.0 5.5 5.0 4.5 4.0 3.5 3.0 2.5 2.0 1.5 1.0 0.5
f1 (ppm)

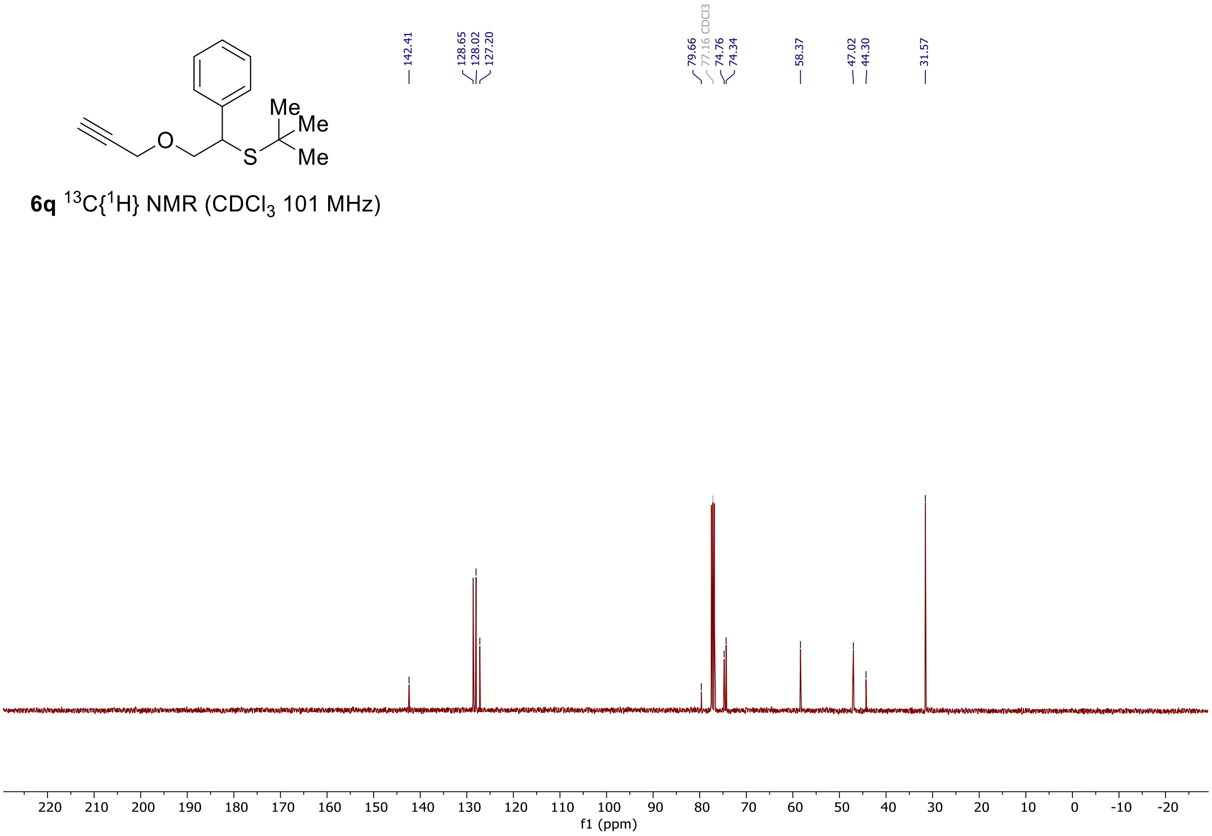
142.41
128.65 128.02 127.20
79.66 77.16 CDCl3 74.76 74.34
58.37
47.02 44.30
31.57
Me
Me
Me
O
S
6q ¹³C{¹H} NMR (CDCl3 101 MHz)
220 210 200 190 180 170 160 150 140 130 120 110 100 90 80 70 60 50 40 30 20 10 0 -10 -20
f1 (ppm)

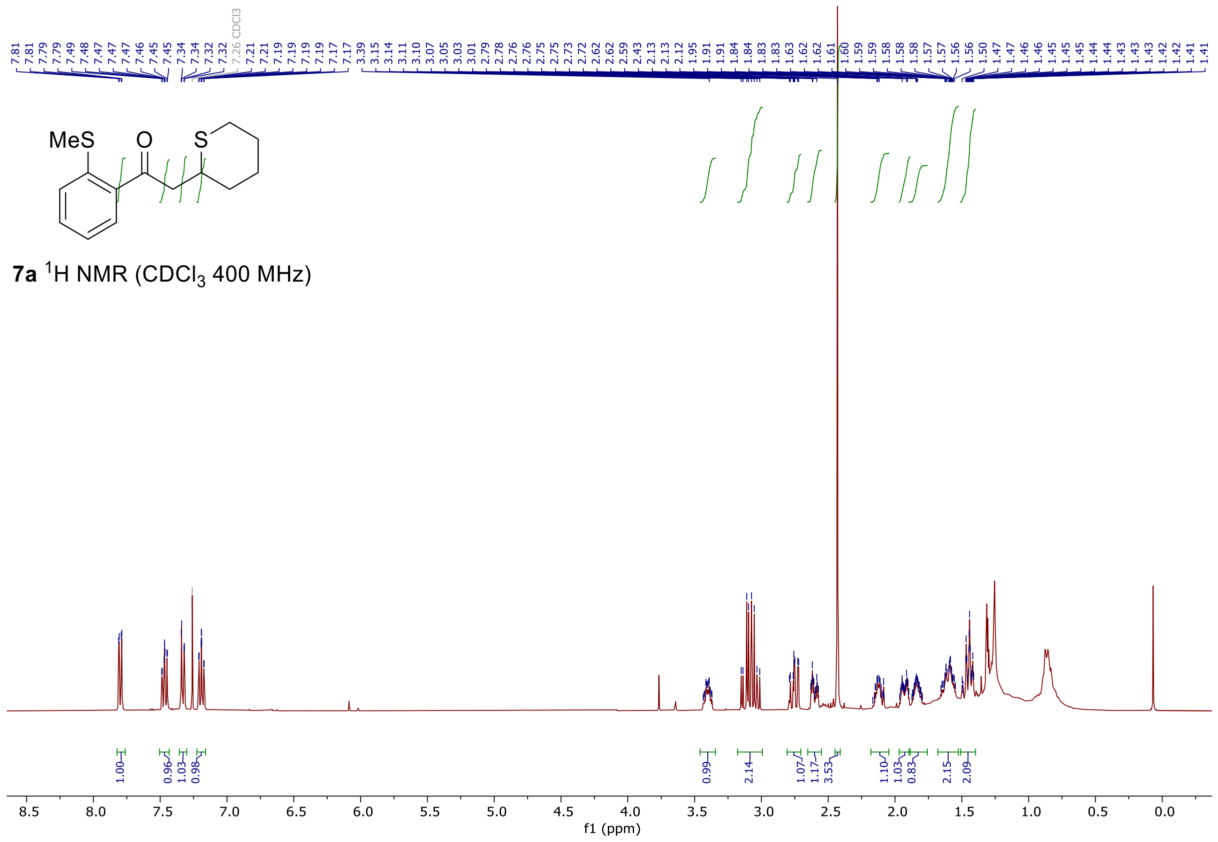

7a ^{1}H NMR ($CDCl_3$ 400 MHz)

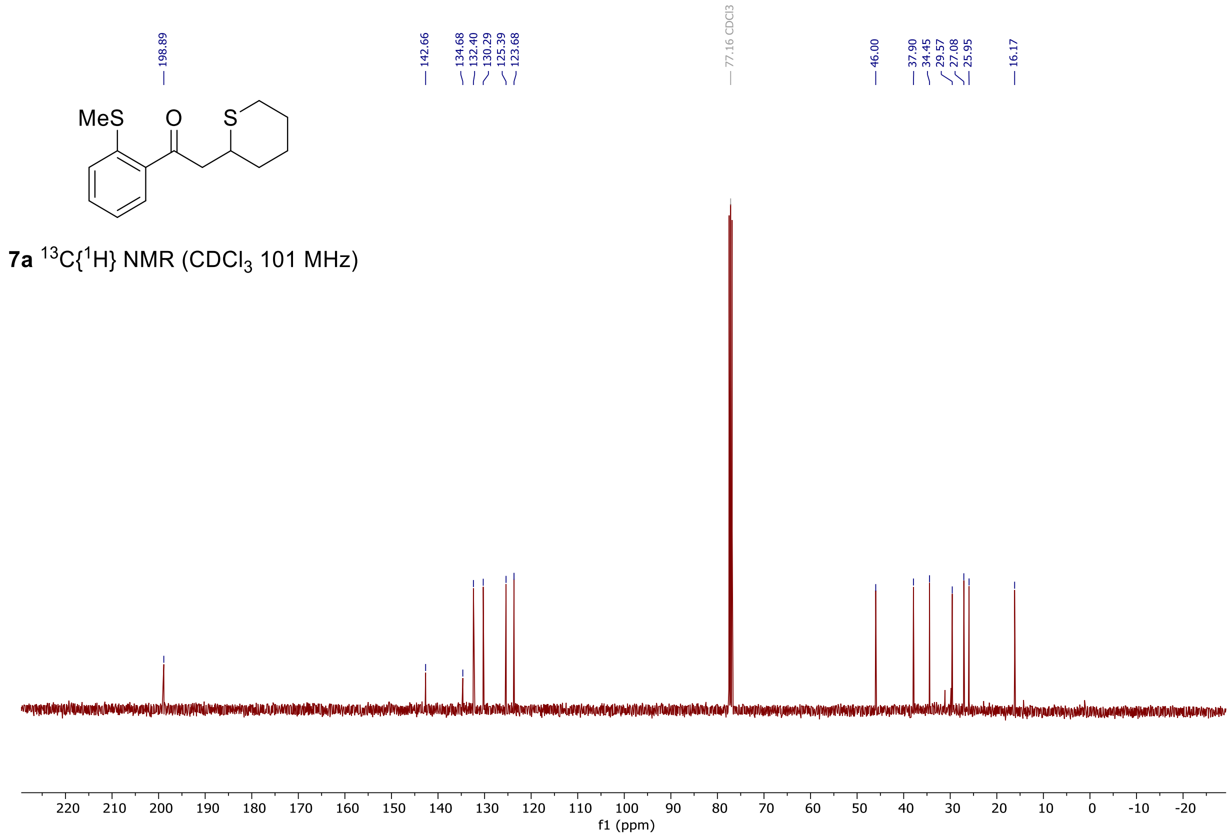

7a $^{13}C\{^{1}H\}$ NMR ($CDCl_3$ 101 MHz)

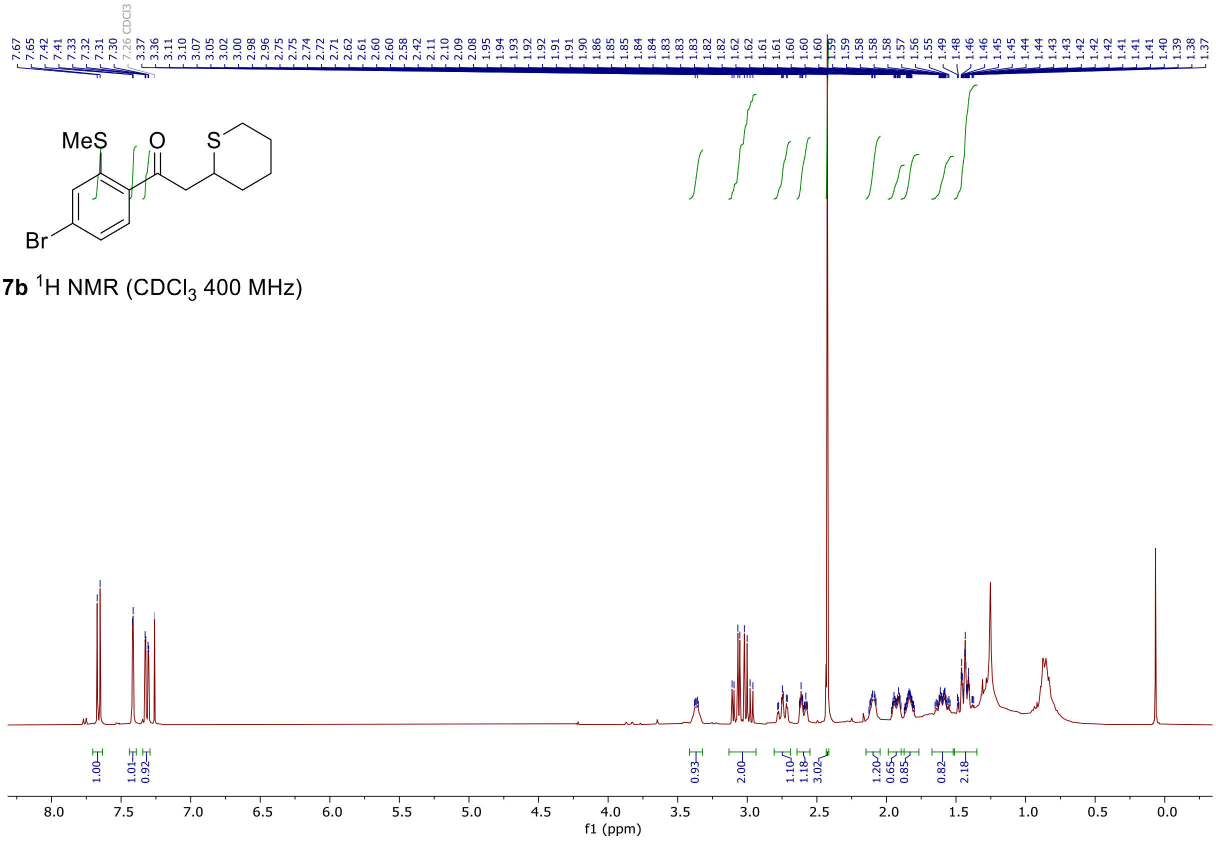

7b ^{1}H NMR ($CDCl_3$ 400 MHz)

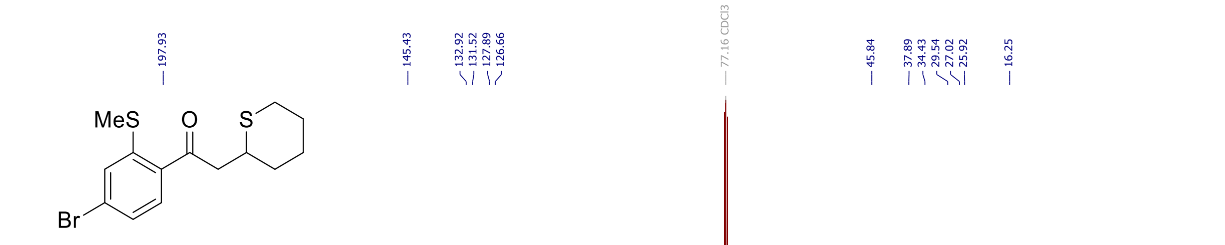

7b ^{13}C{^{1}H} NMR ($CDCl_3$ 101 MHz)

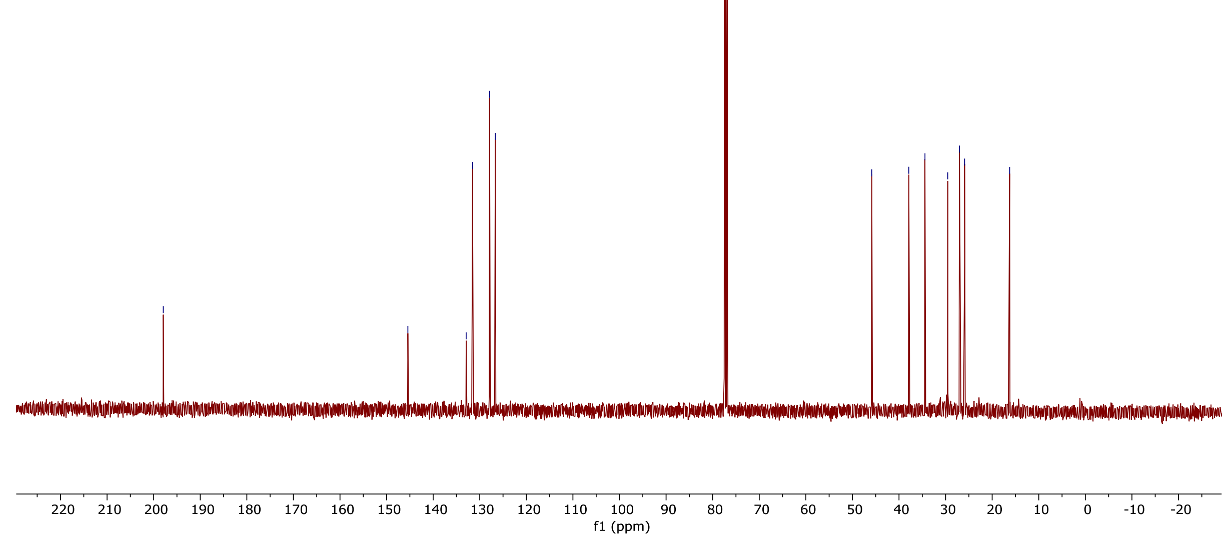

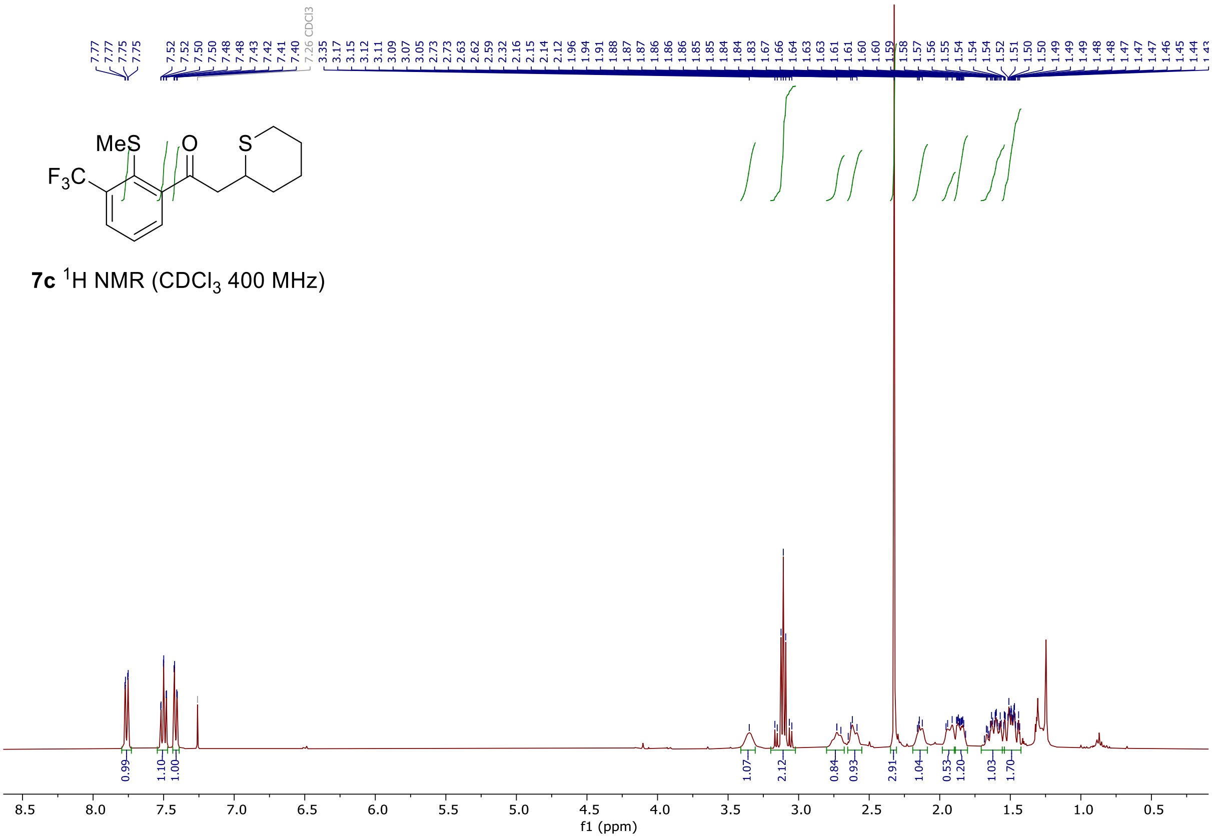
MeS
O
S
F3C
7c 1H NMR (CDCl3 400 MHz)
f1 (ppm)

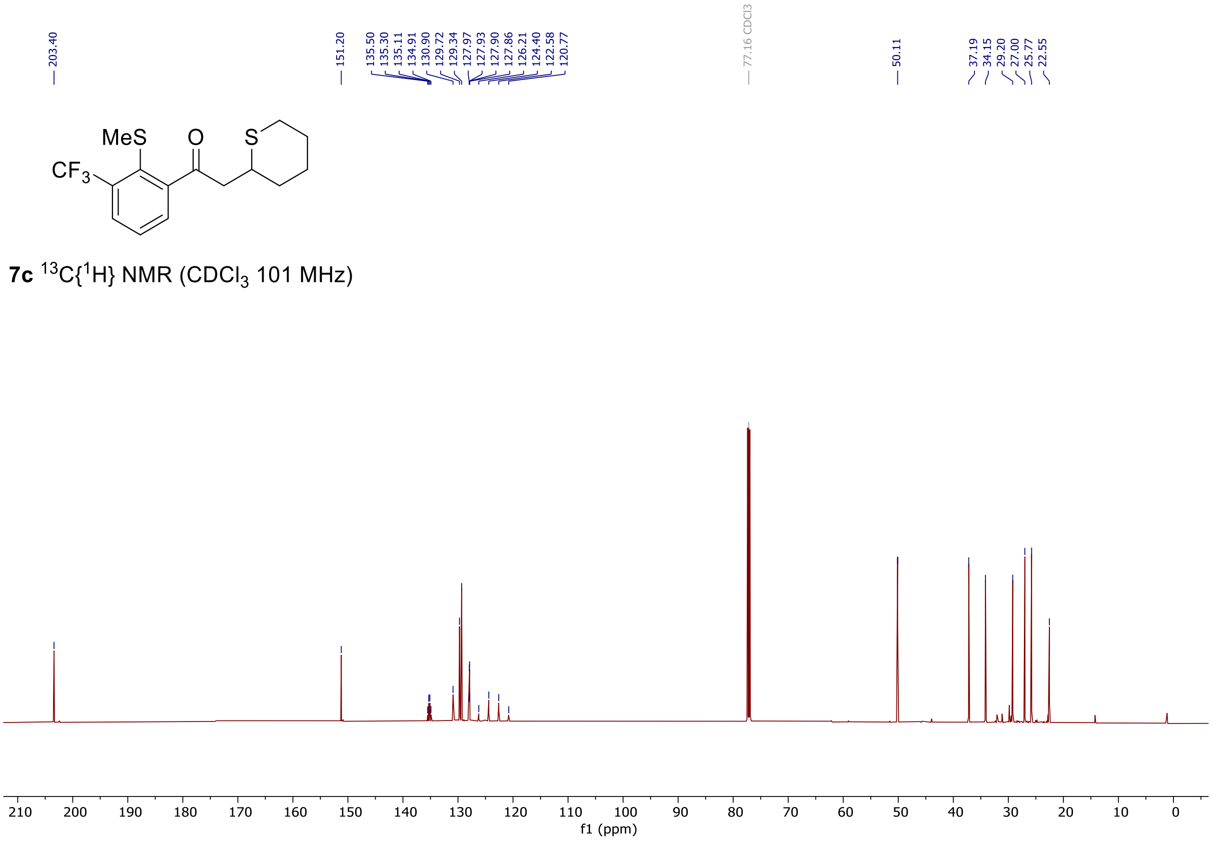
203.40
151.20
135.50
135.30
135.11
134.91
130.90
129.72
129.34
127.97
127.93
127.90
127.86
126.21
124.40
122.58
120.77
77.16 CDCl3
50.11
37.19
34.15
29.20
27.00
25.77
22.55
MeS
O
S
CF3
7c 13C{1H} NMR (CDCl3 101 MHz)
f1 (ppm)

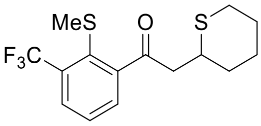

7c $^{19}F\{^{1}H\}$ NMR ($CDCl_3$ 376 MHz)

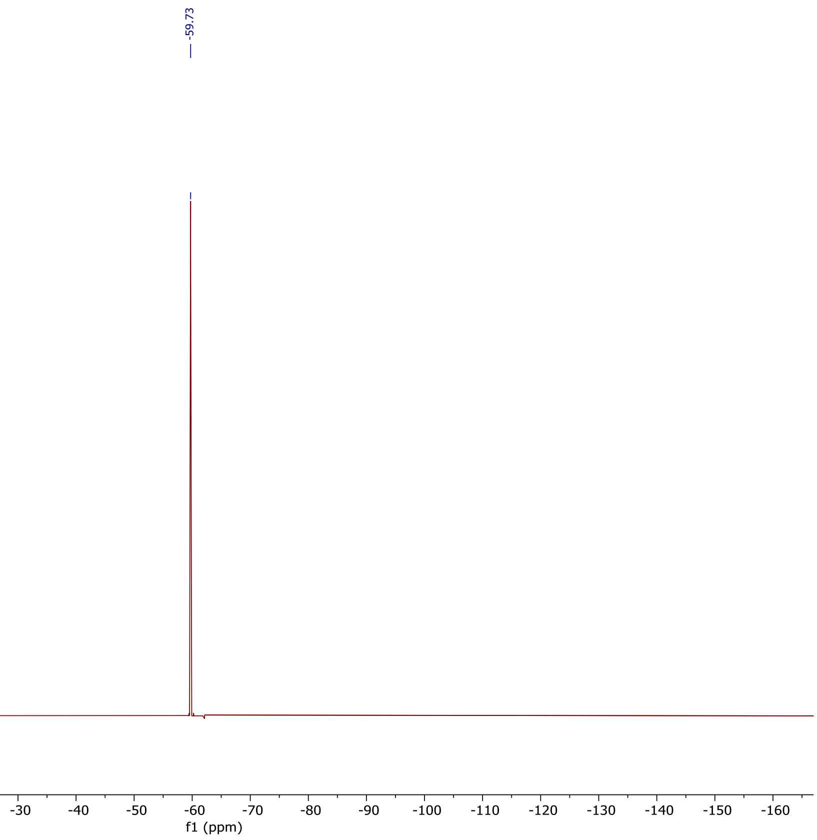

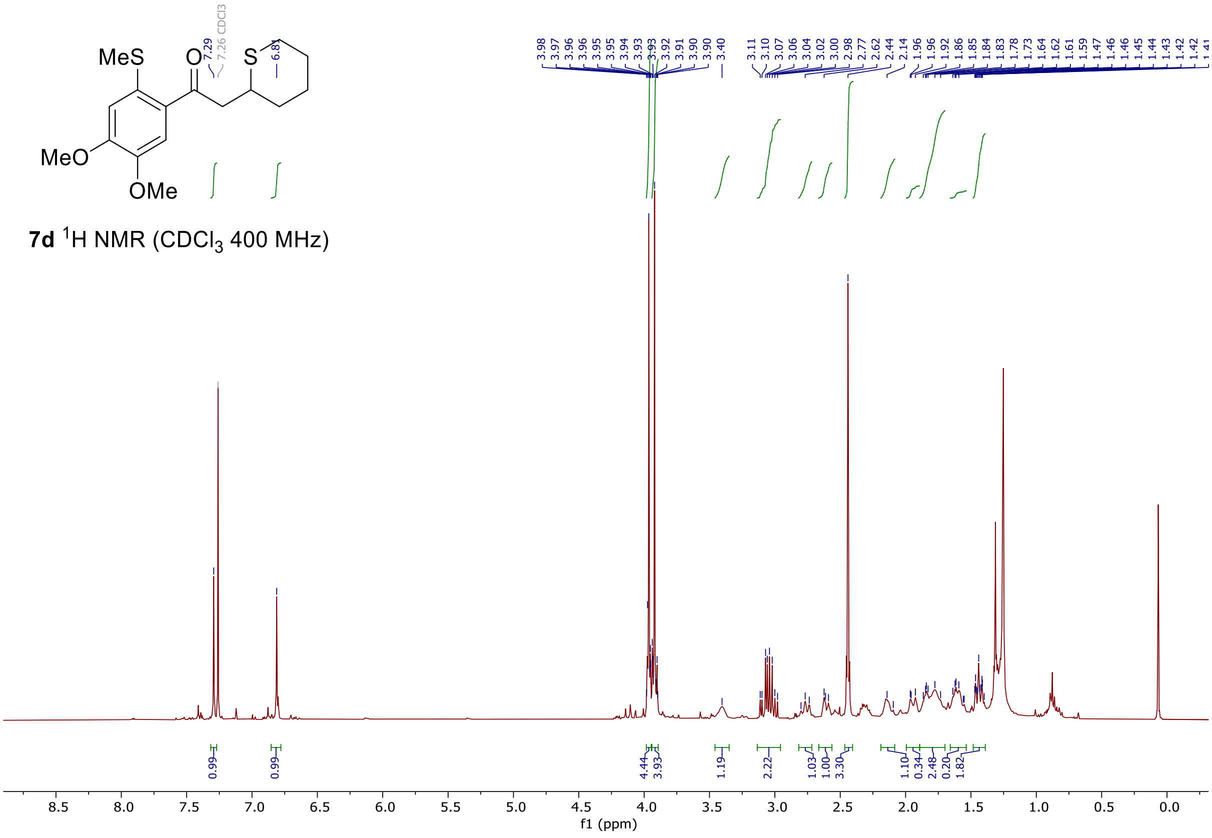

7d ^{1}H NMR ($CDCl_3$ 400 MHz)

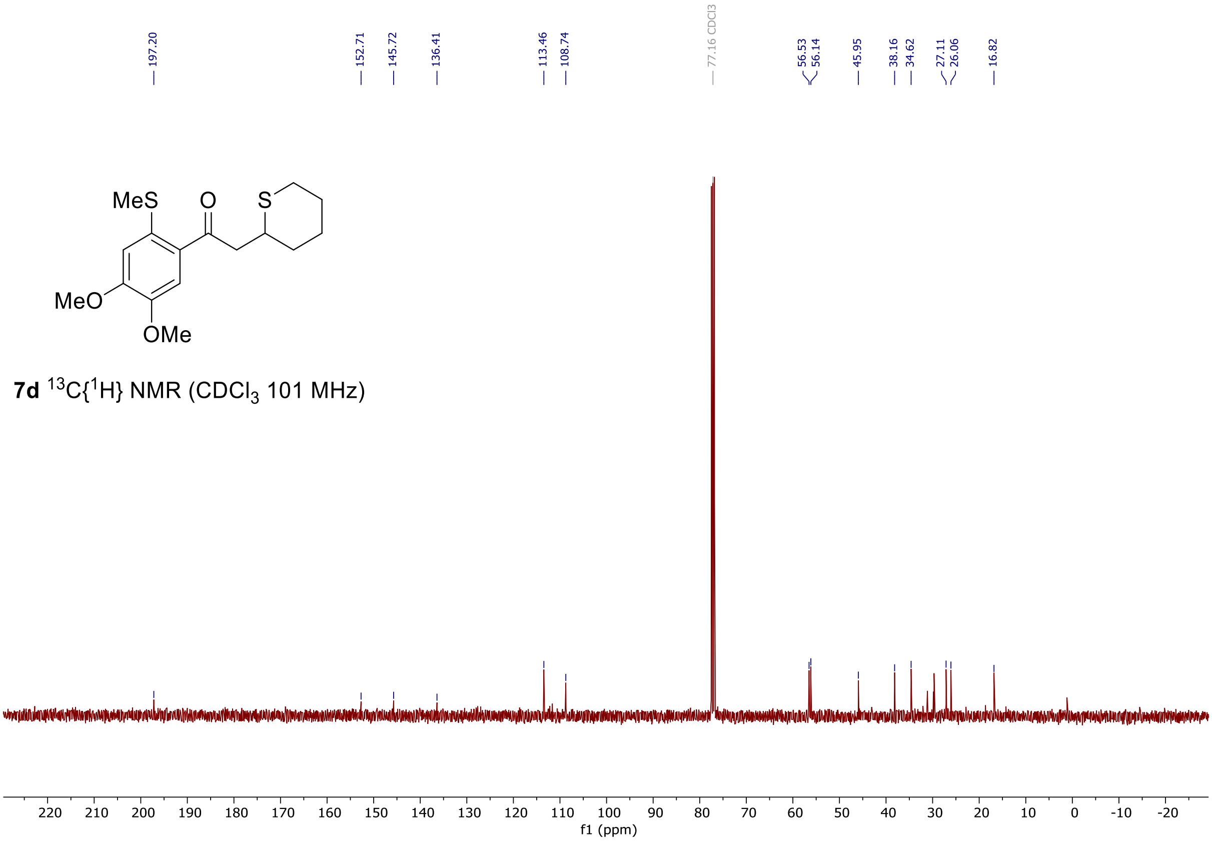

7d $^{13}C\{^{1}H\}$ NMR ($CDCl_3$ 101 MHz)

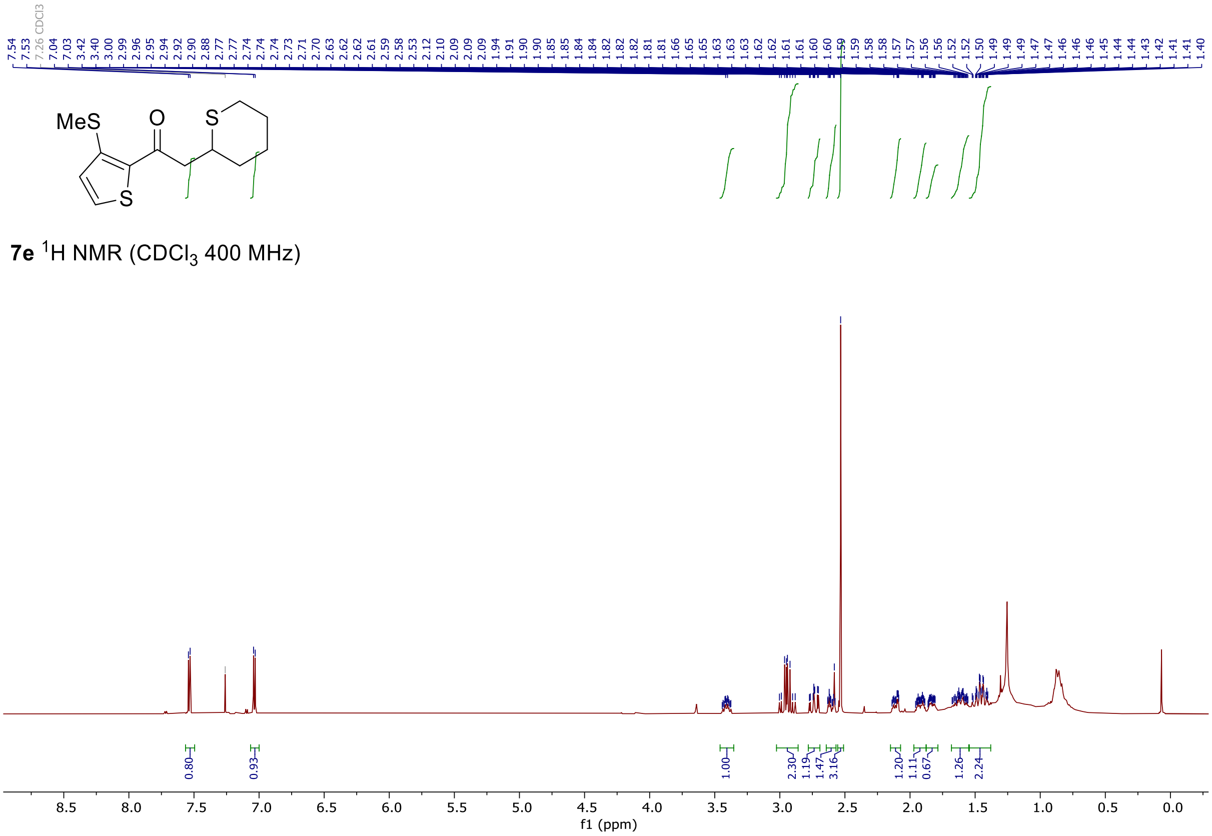

7e ^{1}H NMR ($CDCl_3$ 400 MHz)

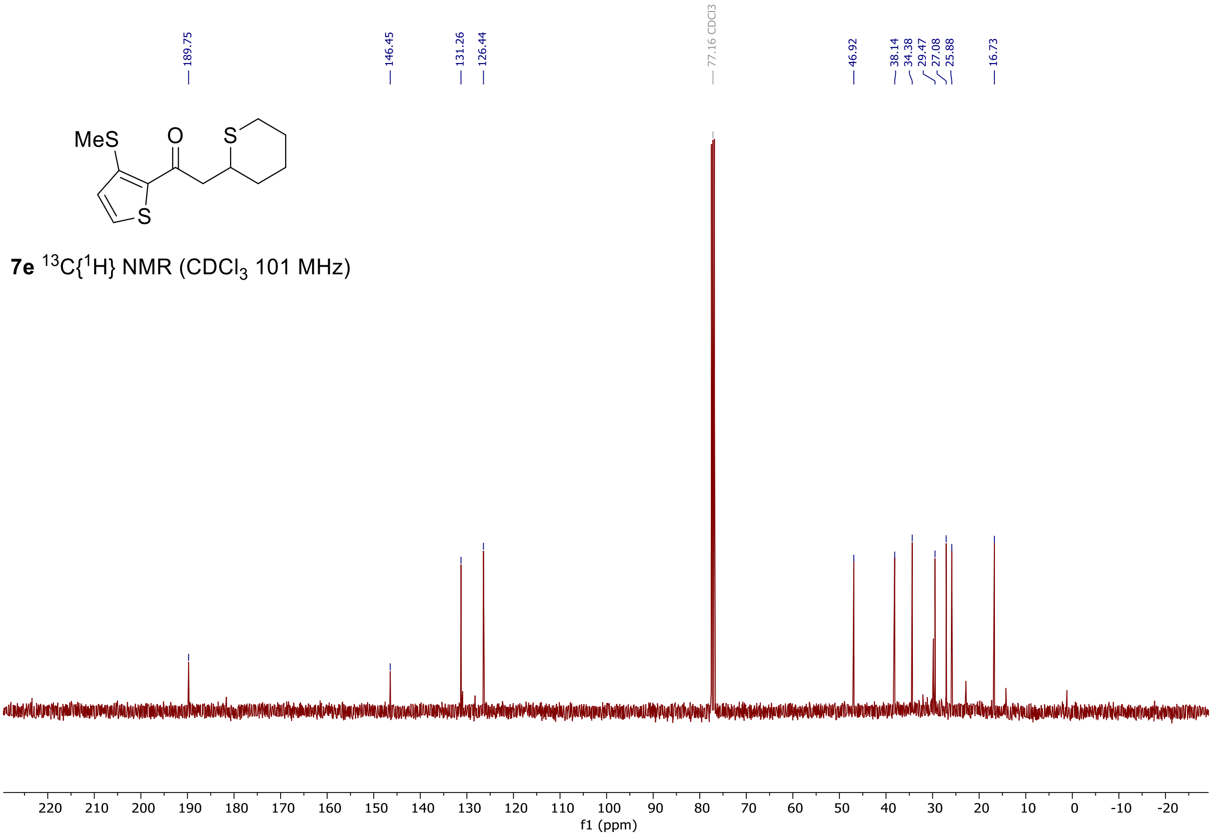

7e $^{13}C\{^{1}H\}$ NMR ($CDCl_3$ 101 MHz)

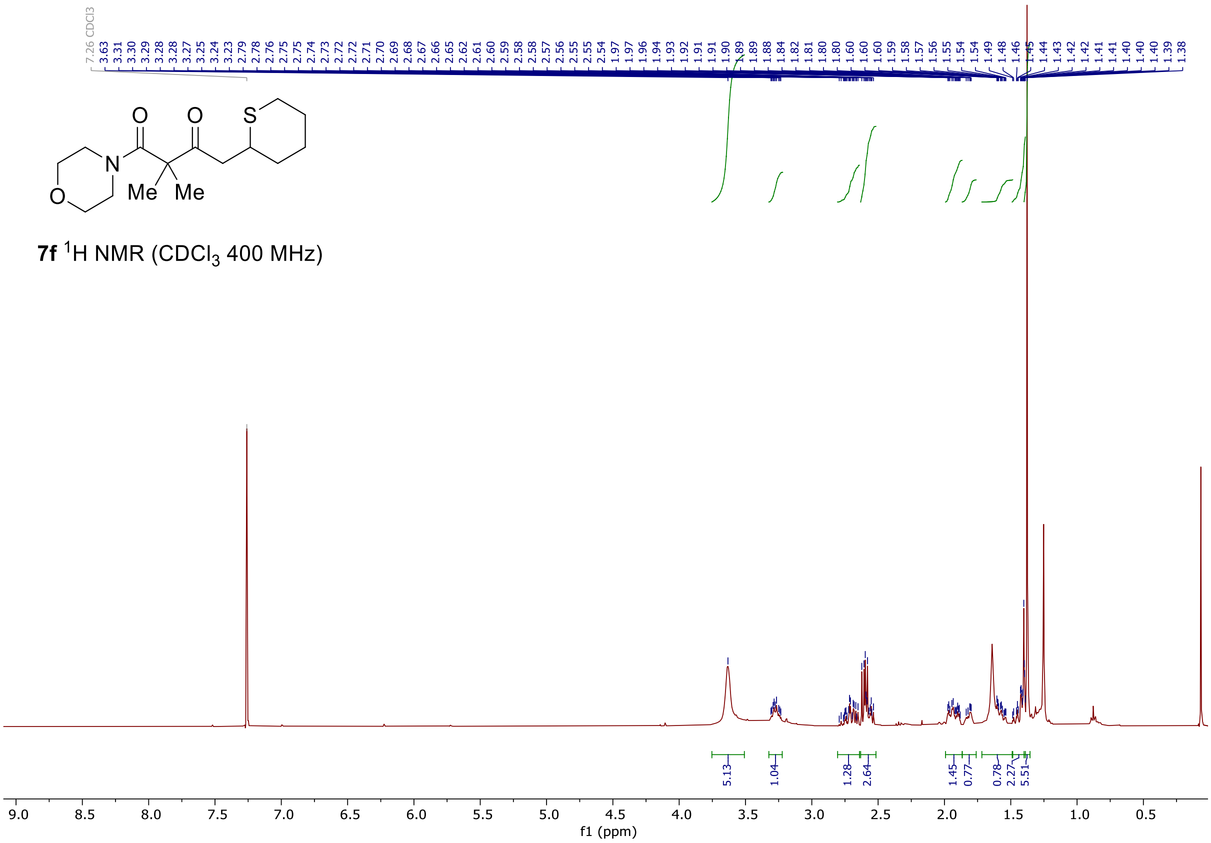

7f ^{1}H NMR ($CDCl_3$ 400 MHz)

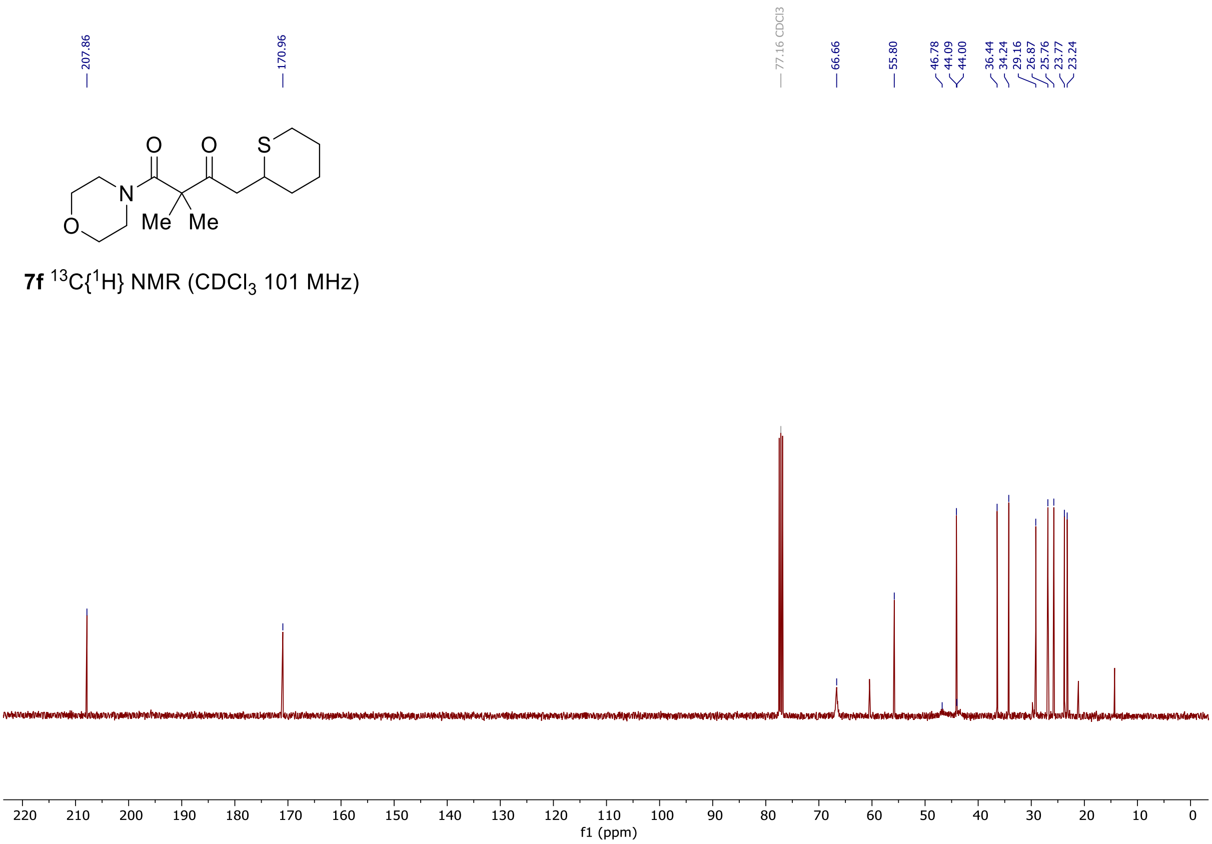

7f $^{13}C\{^{1}H\}$ NMR ($CDCl_3$ 101 MHz)

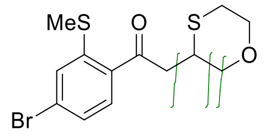

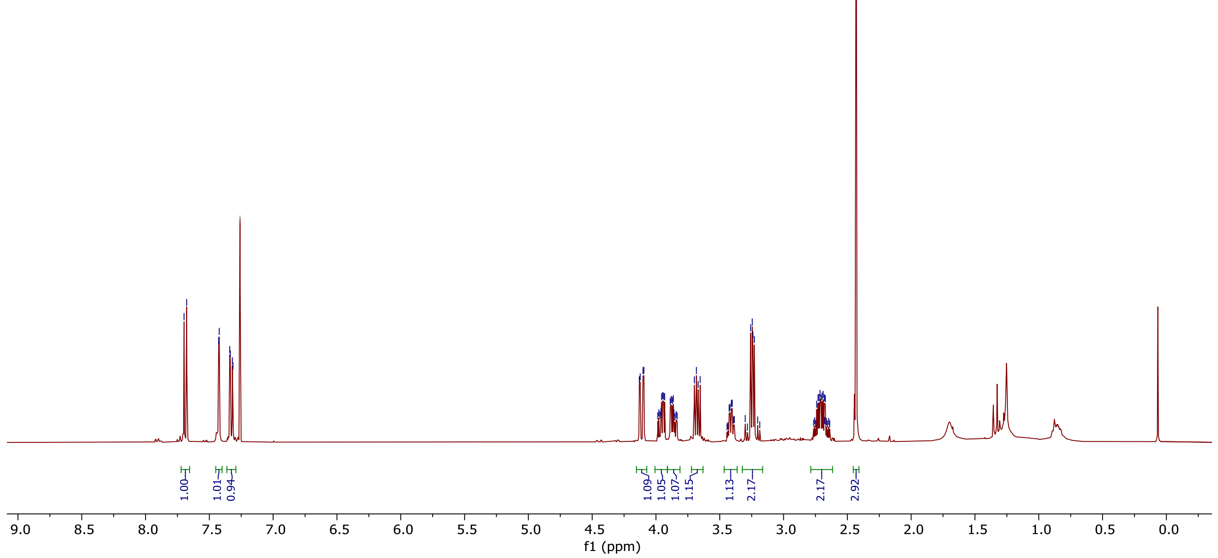

7h ^{1}H NMR ($CDCl_3$ 400 MHz)

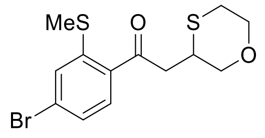

7h $^{13}C\{^{1}H\}$ NMR ($CDCl_3$ 101 MHz)

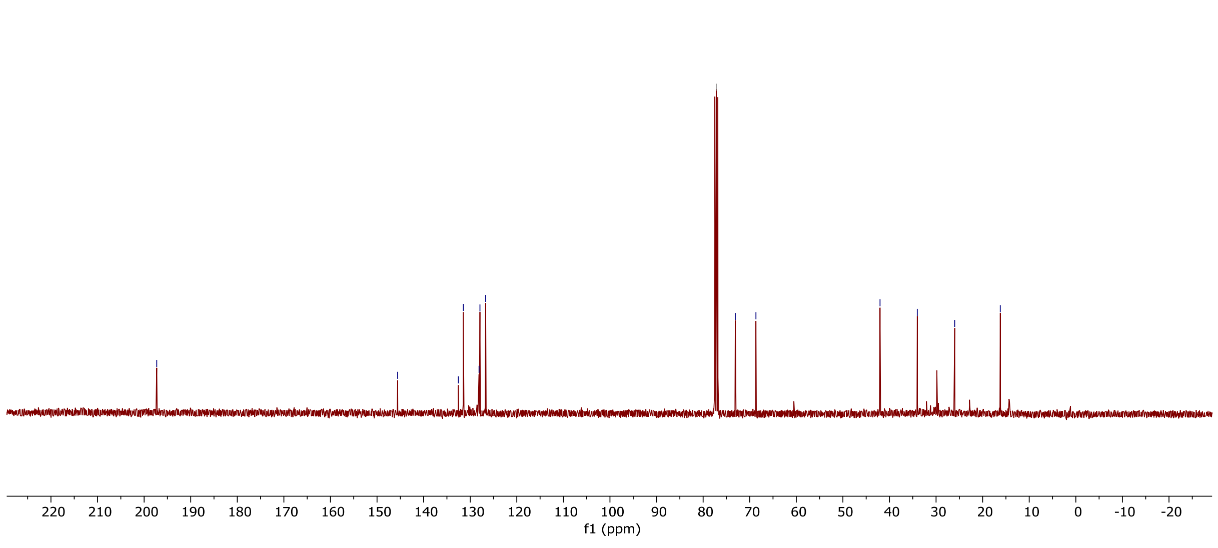

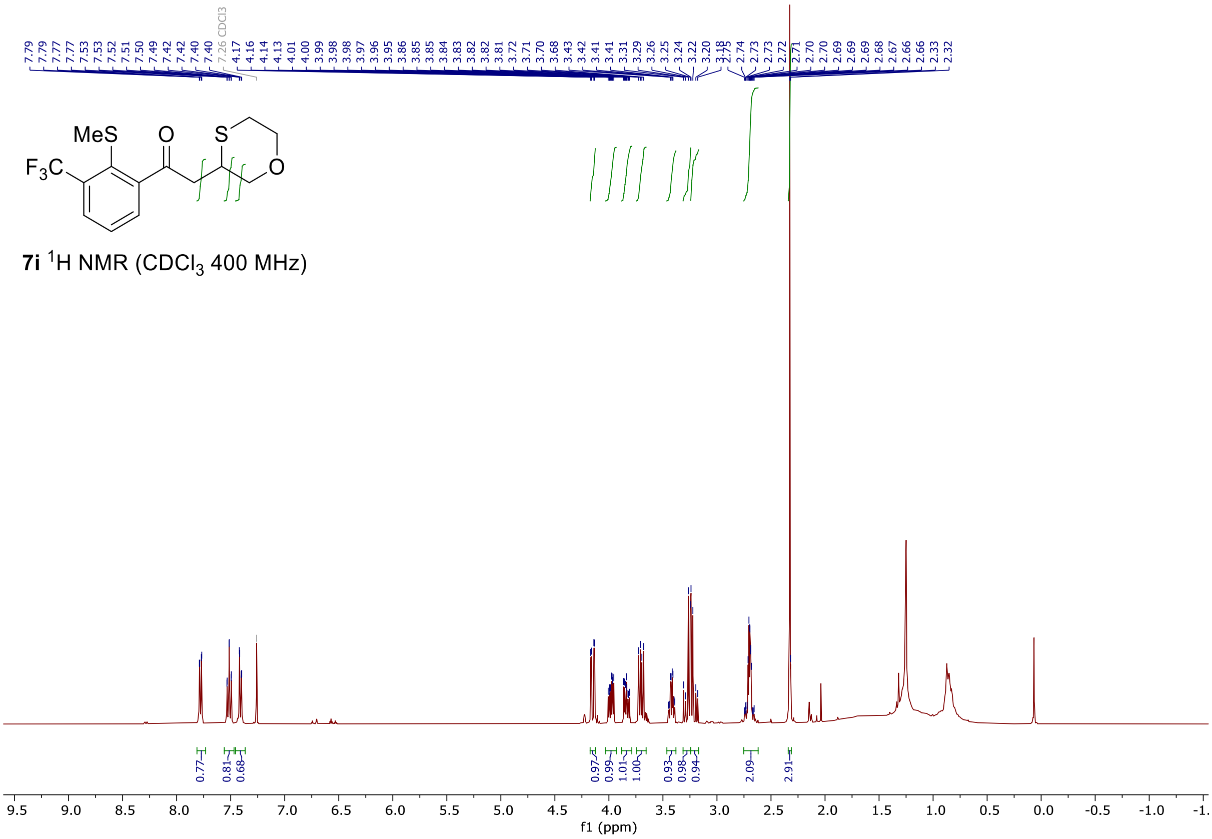

7i ^{1}H NMR ($CDCl_3$ 400 MHz)

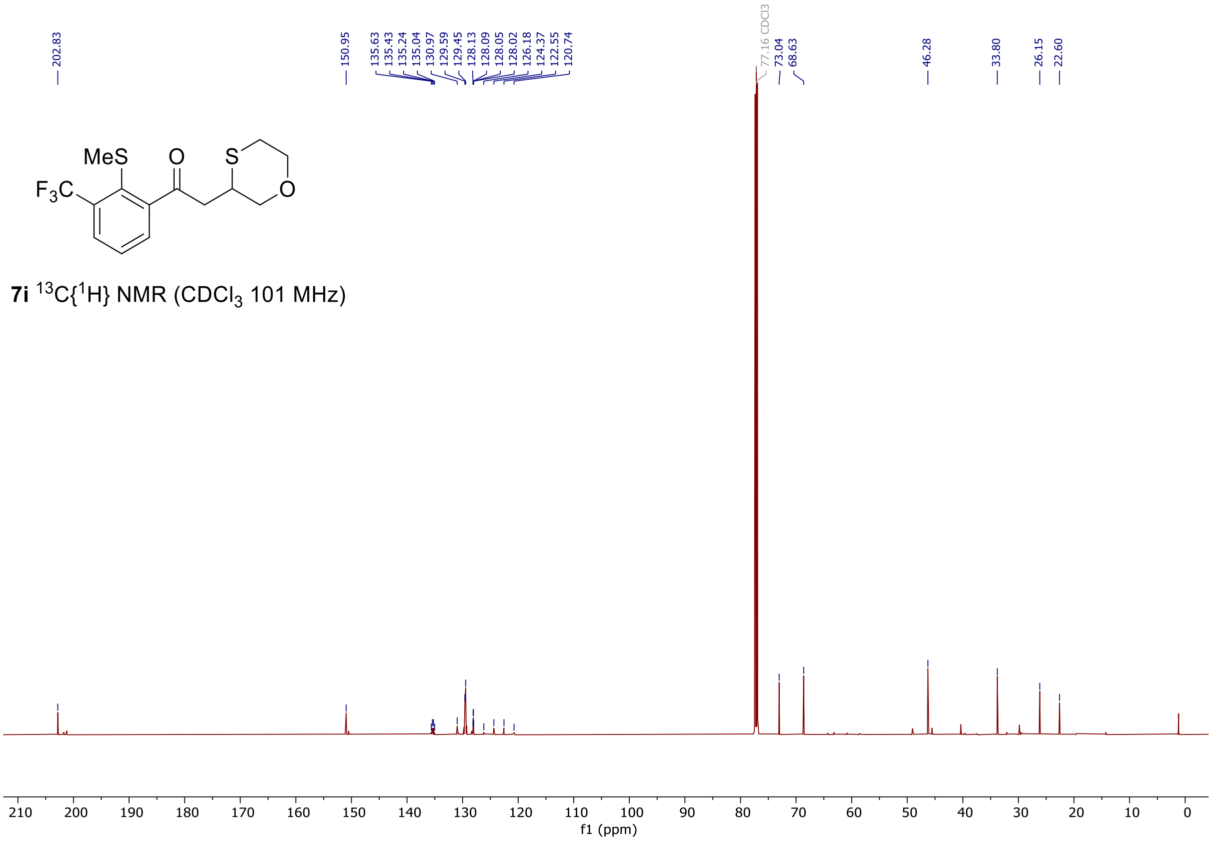

7i ^{13}C{^{1}H} NMR ($CDCl_3$ 101 MHz)

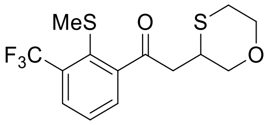

7i $^{19}F\{^{1}H\}$ NMR ($CDCl_3$ 376 MHz)

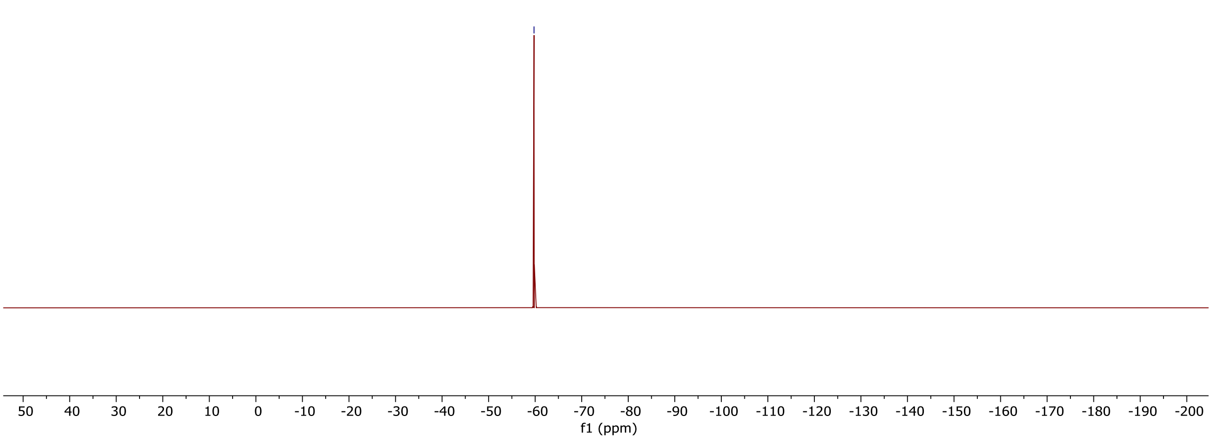

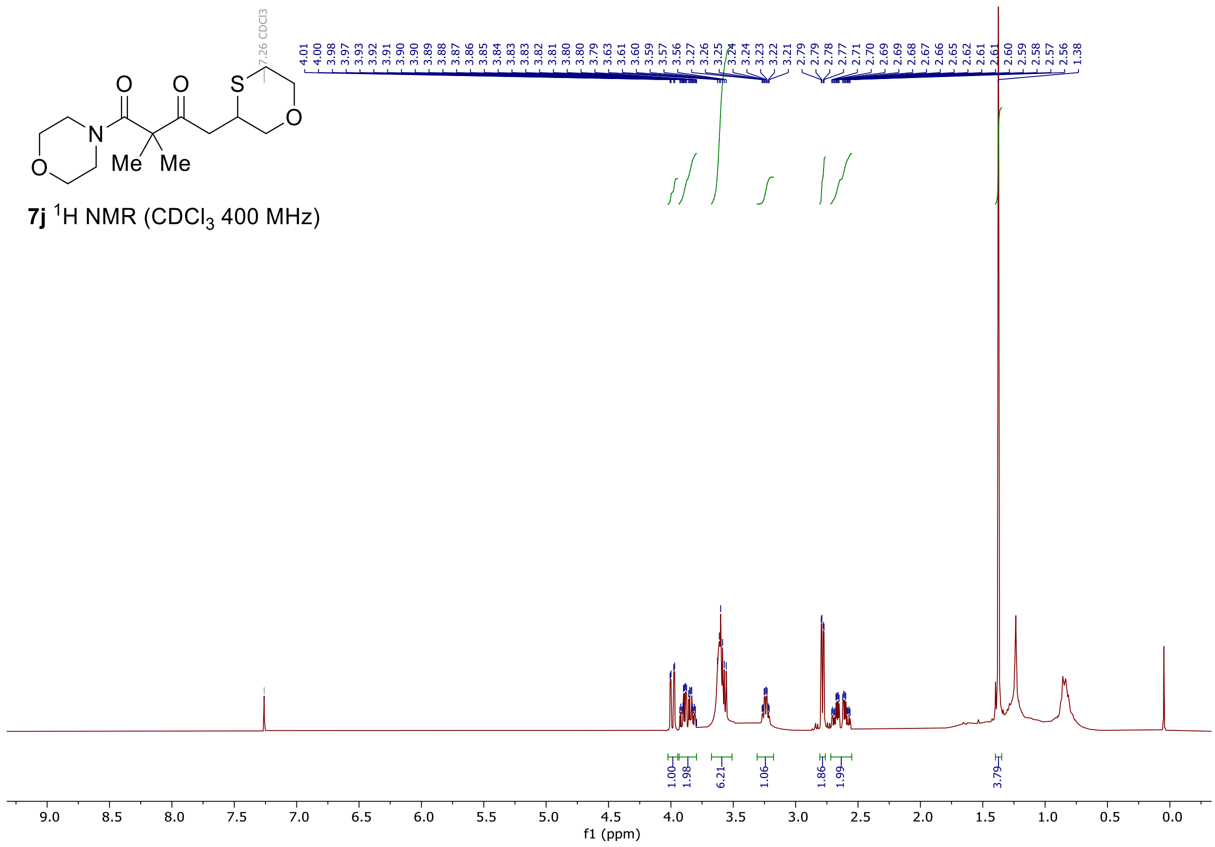
7j 1H NMR (CDCl3 400 MHz)
7.26 CDCl3
4.01 4.00 3.98 3.97 3.93 3.92 3.91 3.90 3.90 3.89 3.88 3.87 3.86 3.85 3.84 3.83 3.83 3.82 3.81 3.80 3.80 3.79 3.63 3.61 3.60 3.59 3.57 3.56 3.27 3.26 3.25 3.24 3.24 3.23 3.22 3.21 2.79 2.79 2.78 2.77 2.71 2.70 2.69 2.69 2.68 2.67 2.66 2.65 2.62 2.61 2.61 2.60 2.59 2.58 2.57 2.56 1.38
1.00 1.98 6.21 1.06 1.86 1.99 3.79
f1 (ppm)

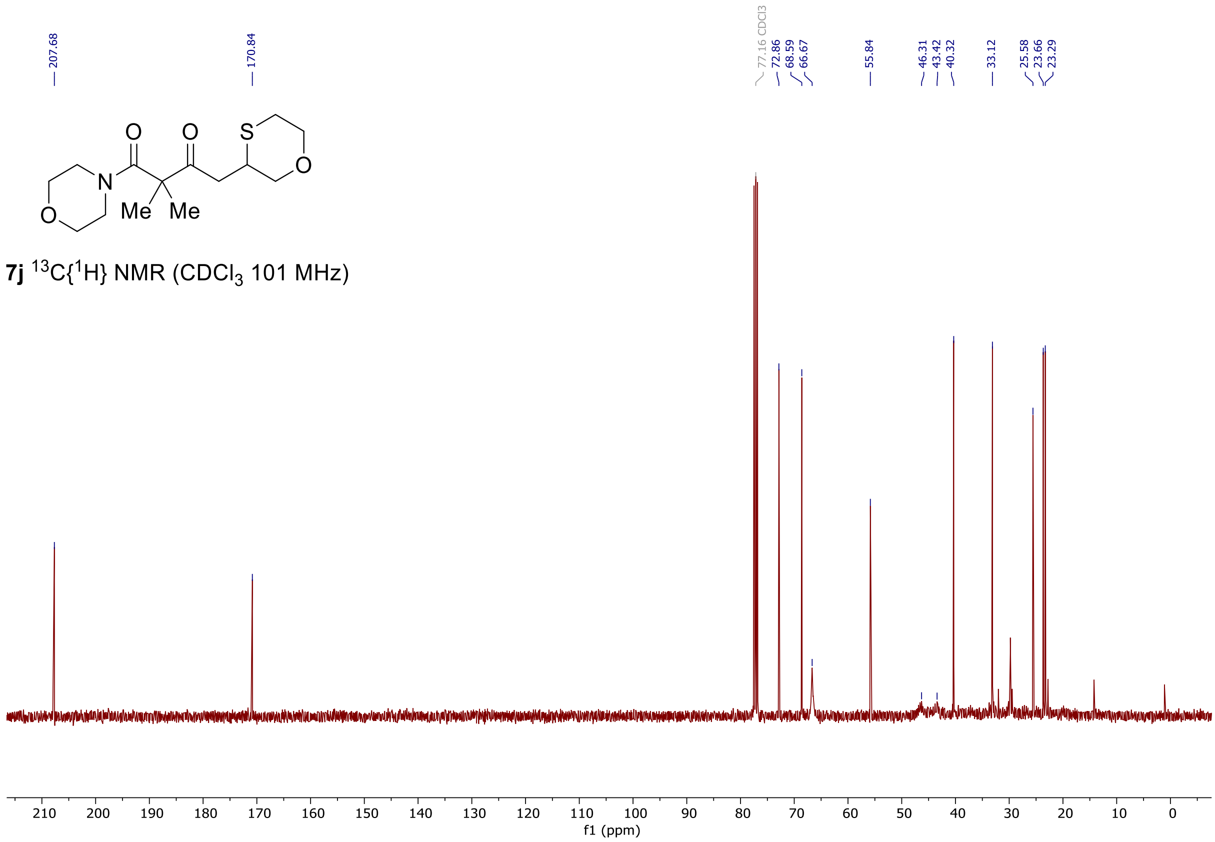
7j 13C{1H} NMR (CDCl3 101 MHz)
207.68
170.84
77.16 CDCl3
72.86 68.59 66.67
55.84
46.31 43.42 40.32
33.12
25.58 23.66 23.29
f1 (ppm)

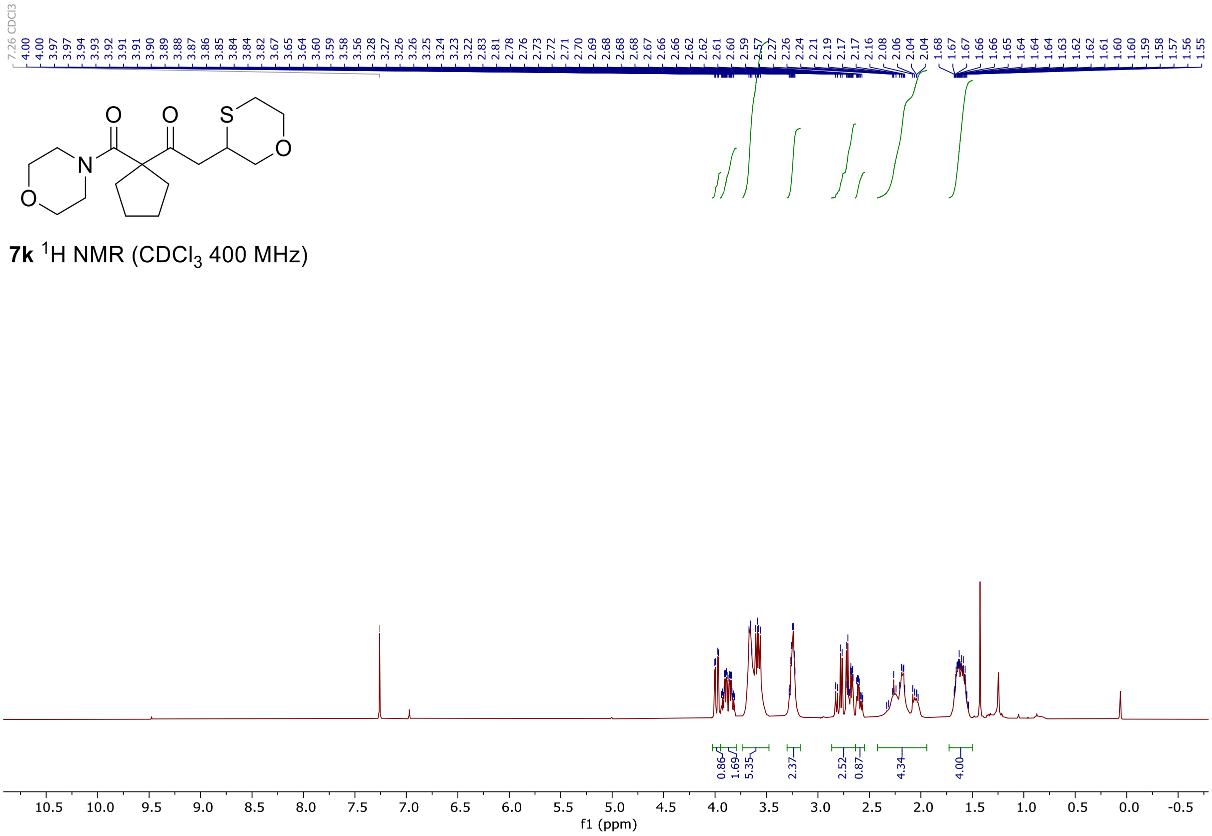

7k ^{1}H NMR ($CDCl_3$ 400 MHz)

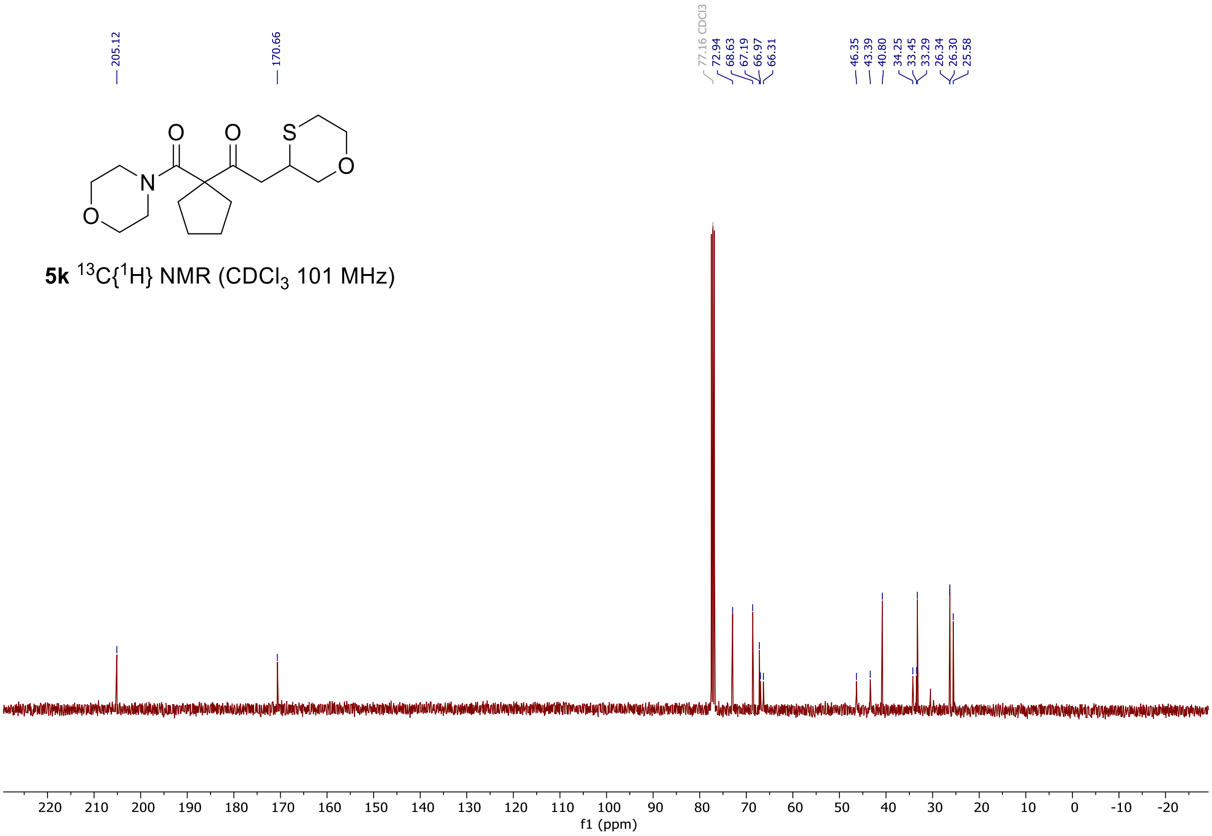

5k ^{13}C{^{1}H} NMR ($CDCl_3$ 101 MHz)

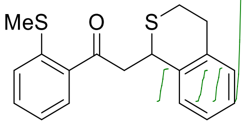

7l ^{1}H NMR ($CDCl_3$ 400 MHz)

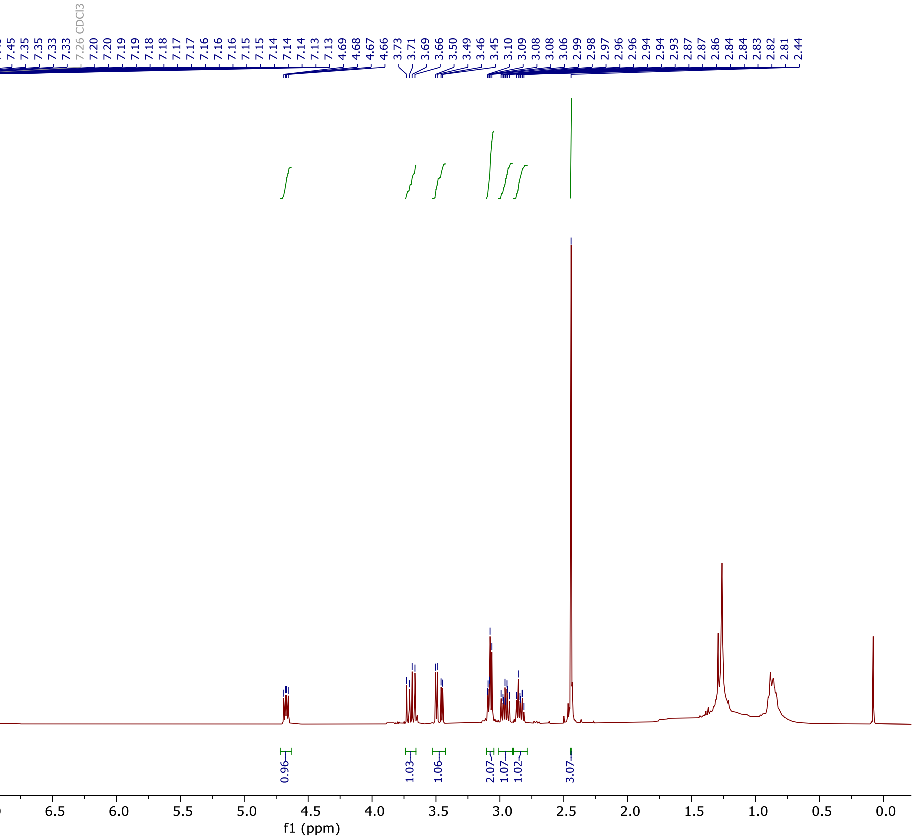

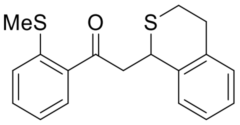

7l ^{13}C{^{1}H} NMR ($CDCl_3$ 101 MHz)

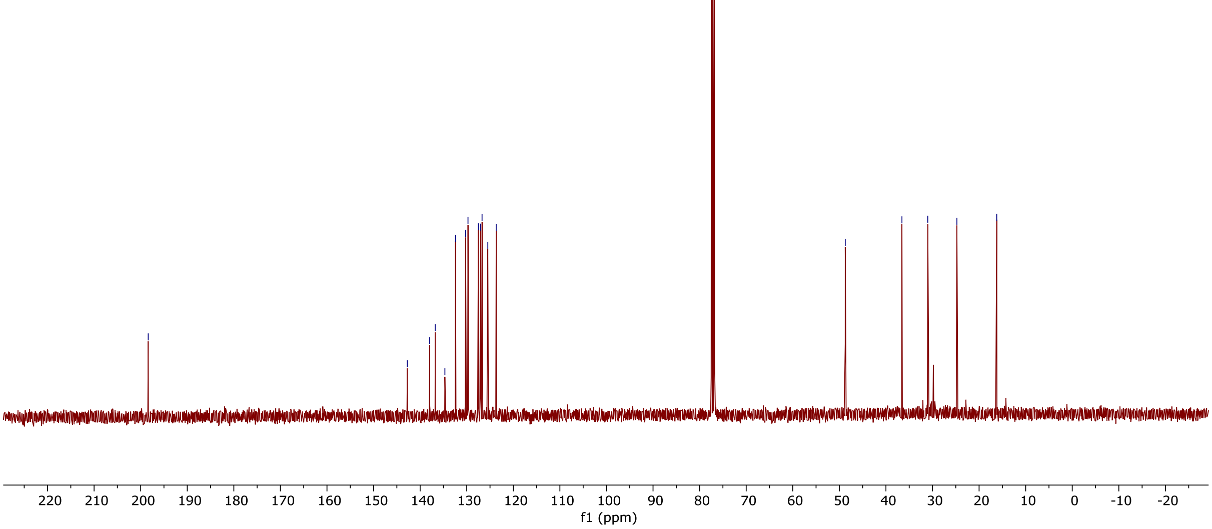

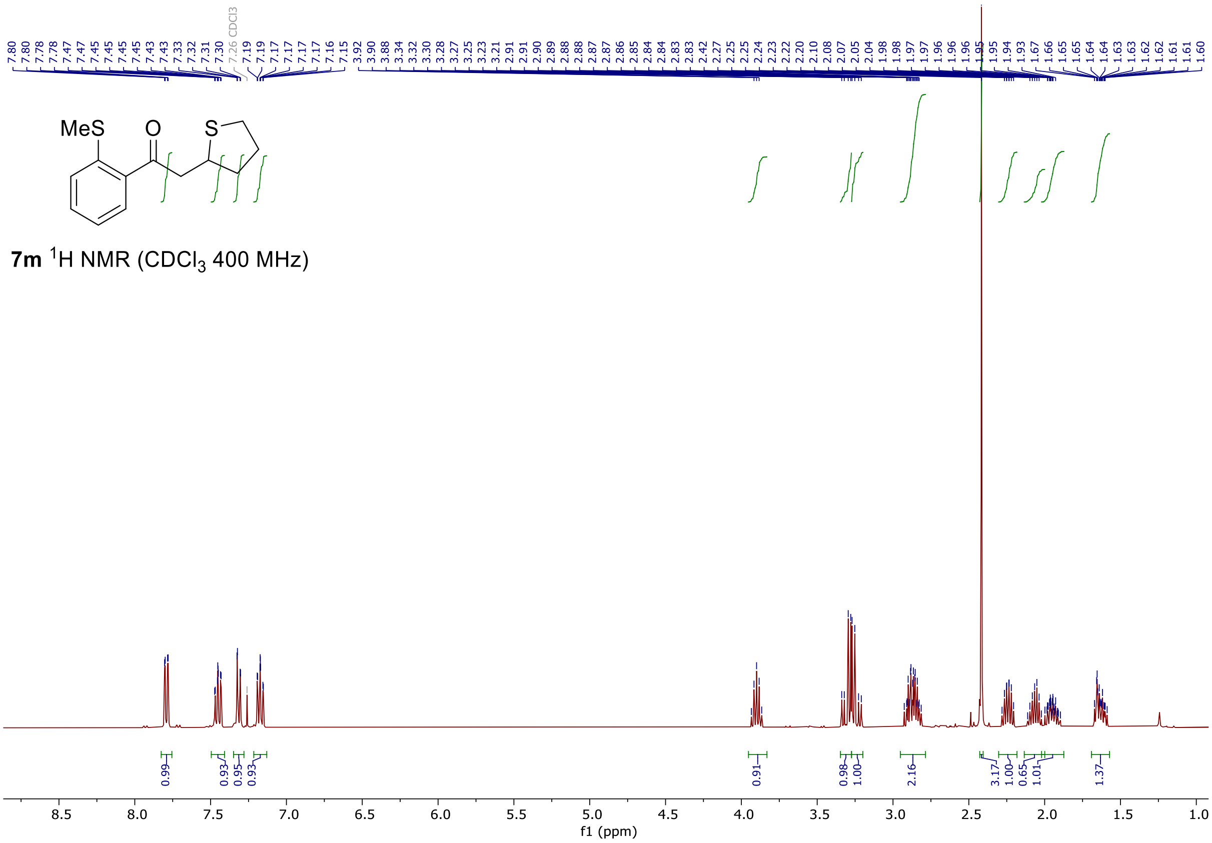

7m ^{1}H NMR (CDCl$_3$ 400 MHz)

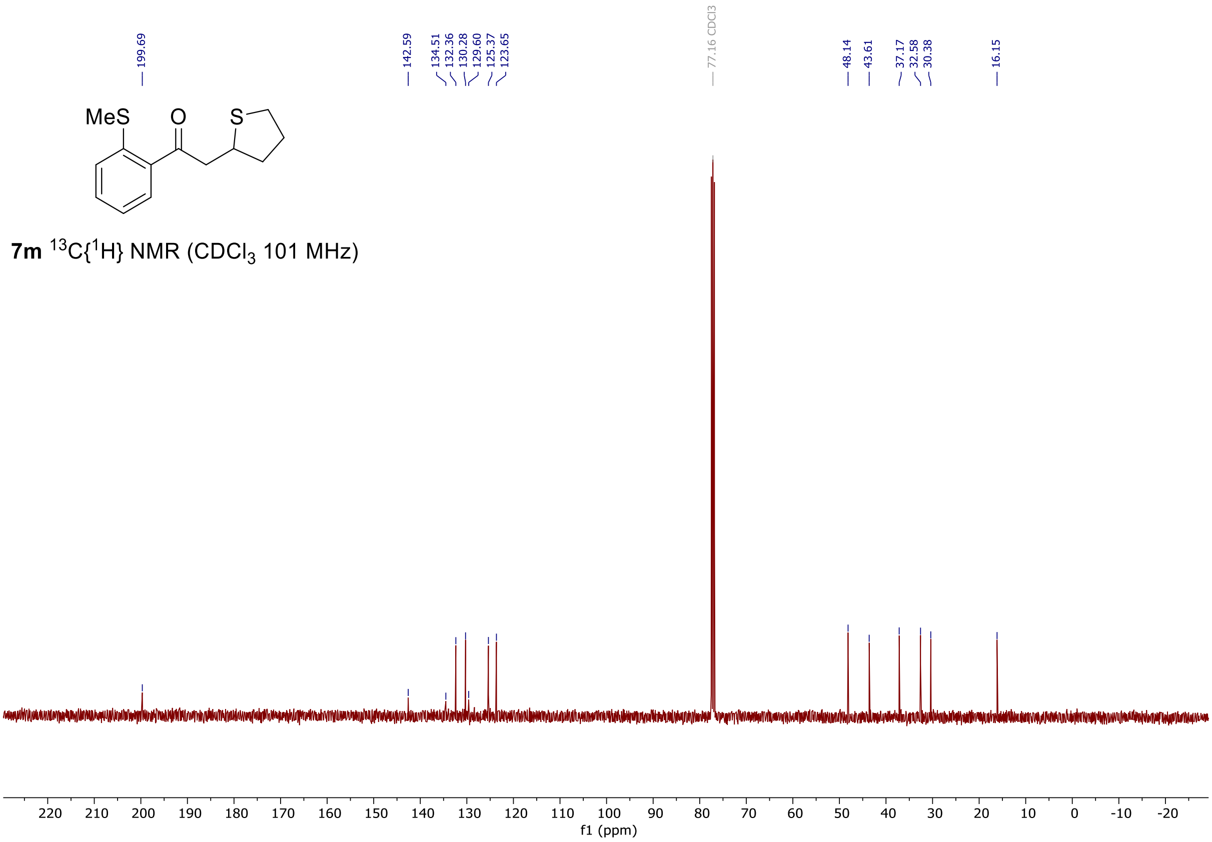

7m ^{13}C{^{1}H} NMR (CDCl$_3$ 101 MHz)

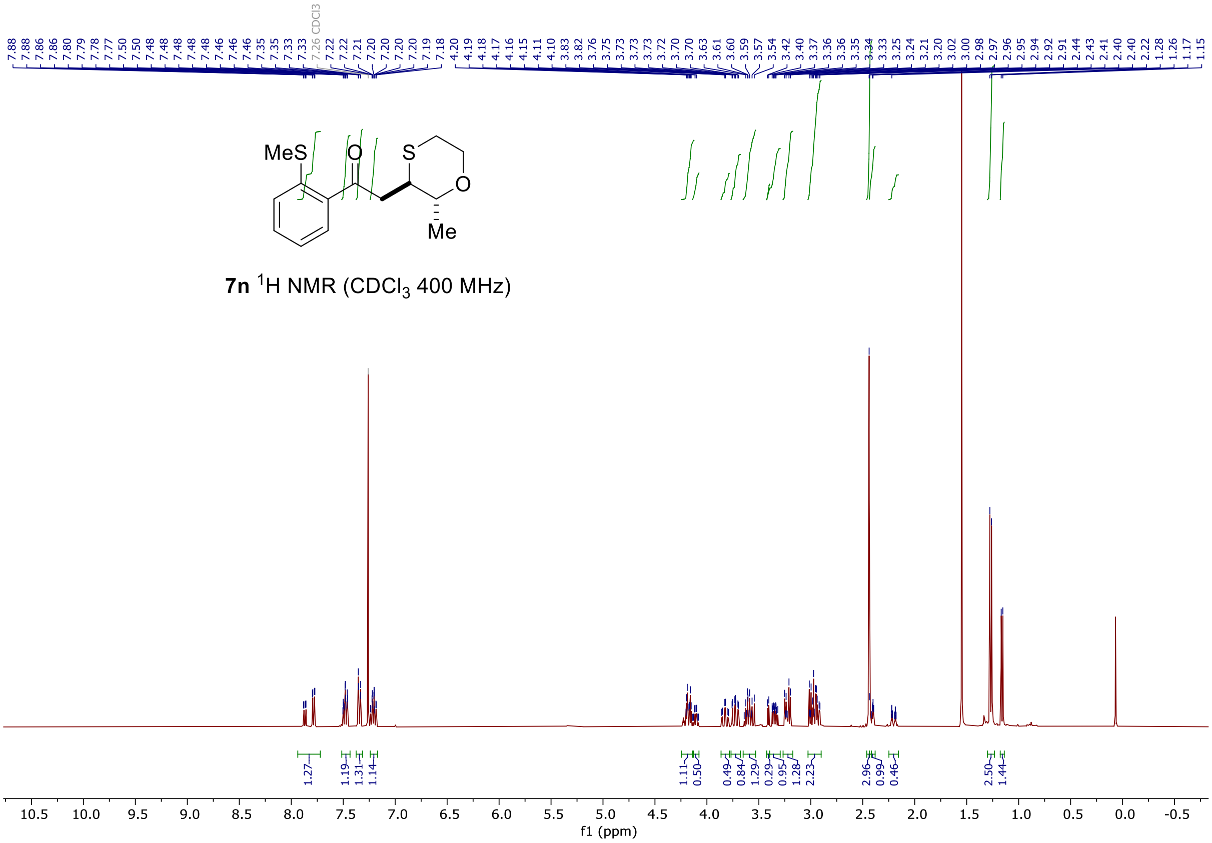
7.88 7.88 7.86 7.80 7.79 7.78 7.77 7.50 7.48 7.48 7.48 7.48 7.46 7.46 7.35 7.35 7.33 7.33 7.26 CDCl3 7.22 7.22 7.21 7.20 7.20 7.20 7.20 7.19 7.18 4.20 4.19 4.18 4.17 4.16 4.15 4.11 4.10 3.83 3.82 3.76 3.75 3.73 3.73 3.73 3.72 3.70 3.70 3.63 3.61 3.60 3.59 3.57 3.54 3.42 3.40 3.37 3.36 3.36 3.35 3.34 3.33 3.25 3.24 3.21 3.20 3.02 3.00 2.98 2.97 2.96 2.95 2.94 2.92 2.91 2.44 2.43 2.41 2.40 2.40 2.22 1.28 1.26 1.17 1.15
MeS
O
S
O
Me
7n 1H NMR (CDCl3 400 MHz)
1.27 1.19 1.31 1.14 1.11 0.50 0.49 0.84 1.29 0.29 0.95 1.28 2.23 2.96 0.99 0.46 2.50 1.44
10.5 10.0 9.5 9.0 8.5 8.0 7.5 7.0 6.5 6.0 5.5 5.0 4.5 4.0 3.5 3.0 2.5 2.0 1.5 1.0 0.5 0.0 -0.5
f1 (ppm)

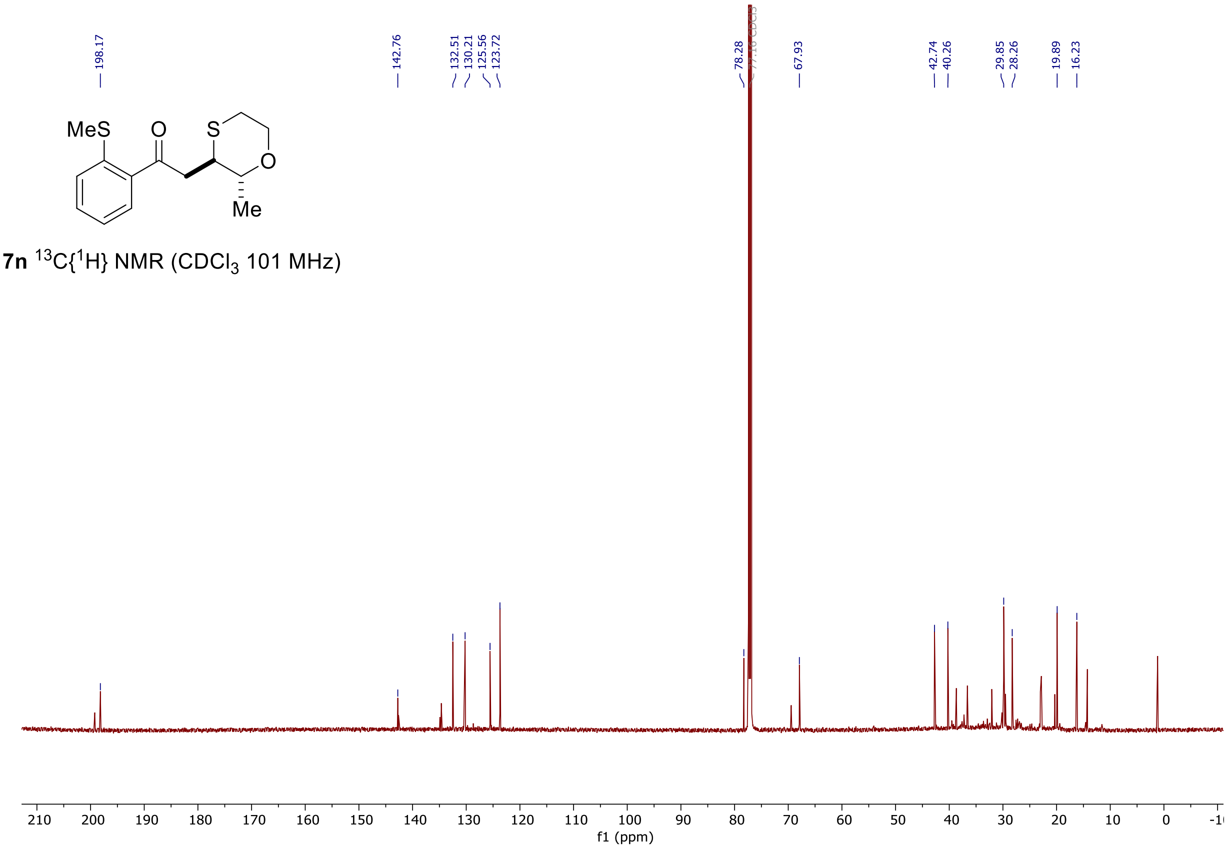
198.17
142.76
132.51 130.21 125.56 123.72
78.28 77.16 CDCl3
67.93
42.74 40.26
29.85 28.26
19.89 16.23
MeS
O
S
O
Me
7n 13C{1H} NMR (CDCl3 101 MHz)
210 200 190 180 170 160 150 140 130 120 110 100 90 80 70 60 50 40 30 20 10 0
f1 (ppm)

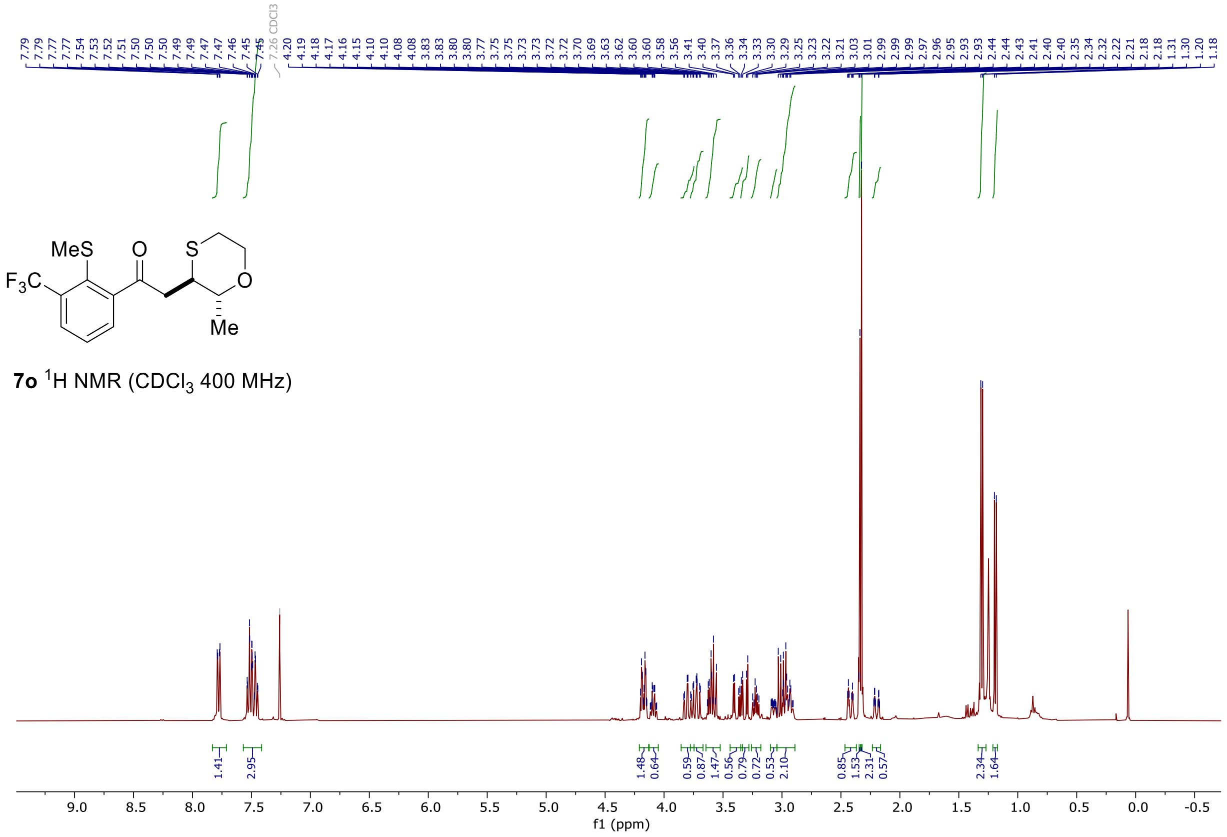

7o ^{1}H NMR ($CDCl_3$ 400 MHz)

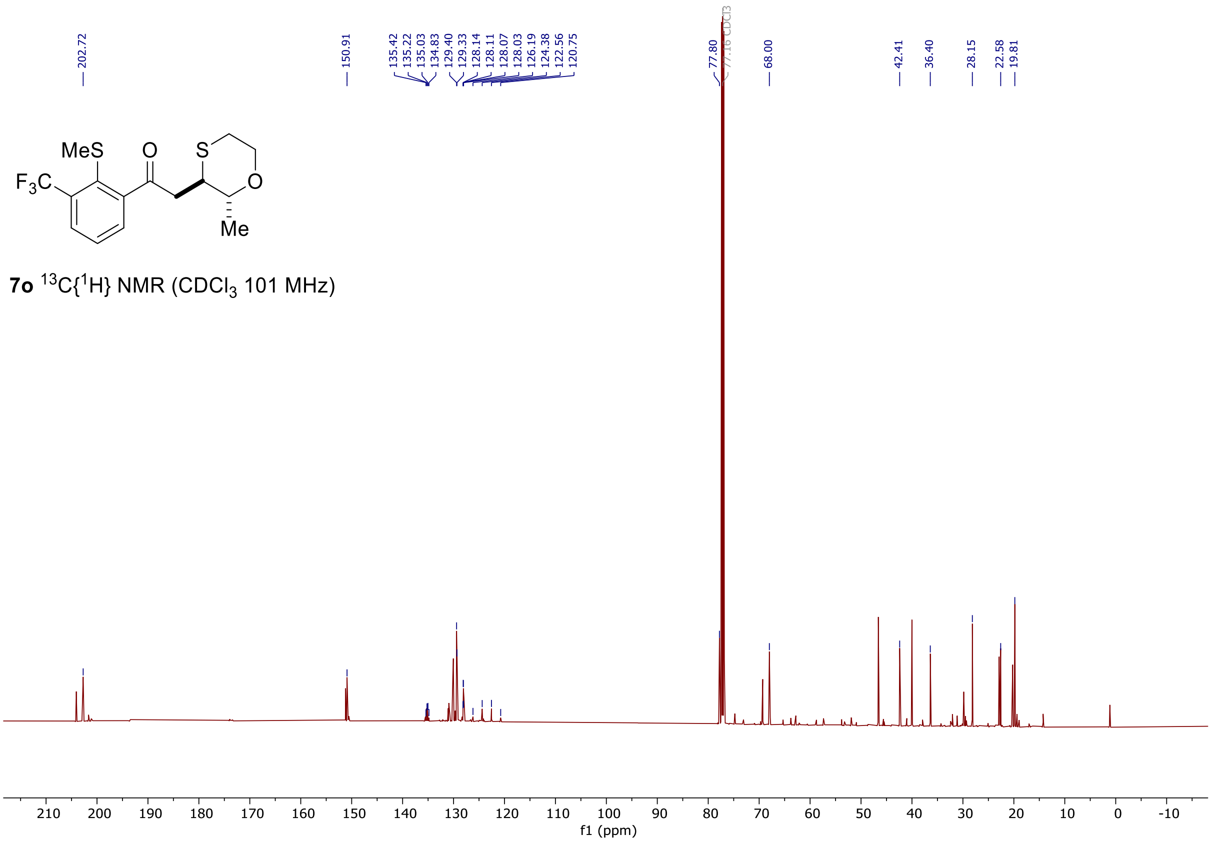

7o ^{13}C{^{1}H} NMR ($CDCl_3$ 101 MHz)

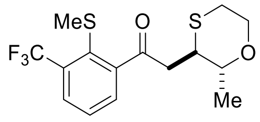

7o $^{19}F\{^1H\}$ NMR (CDCl$_3$ 376 MHz)

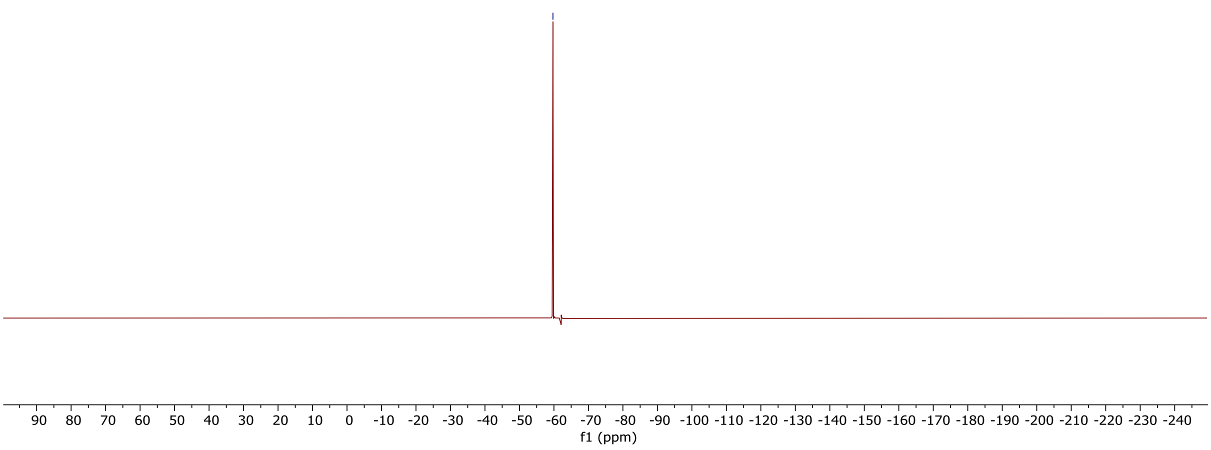

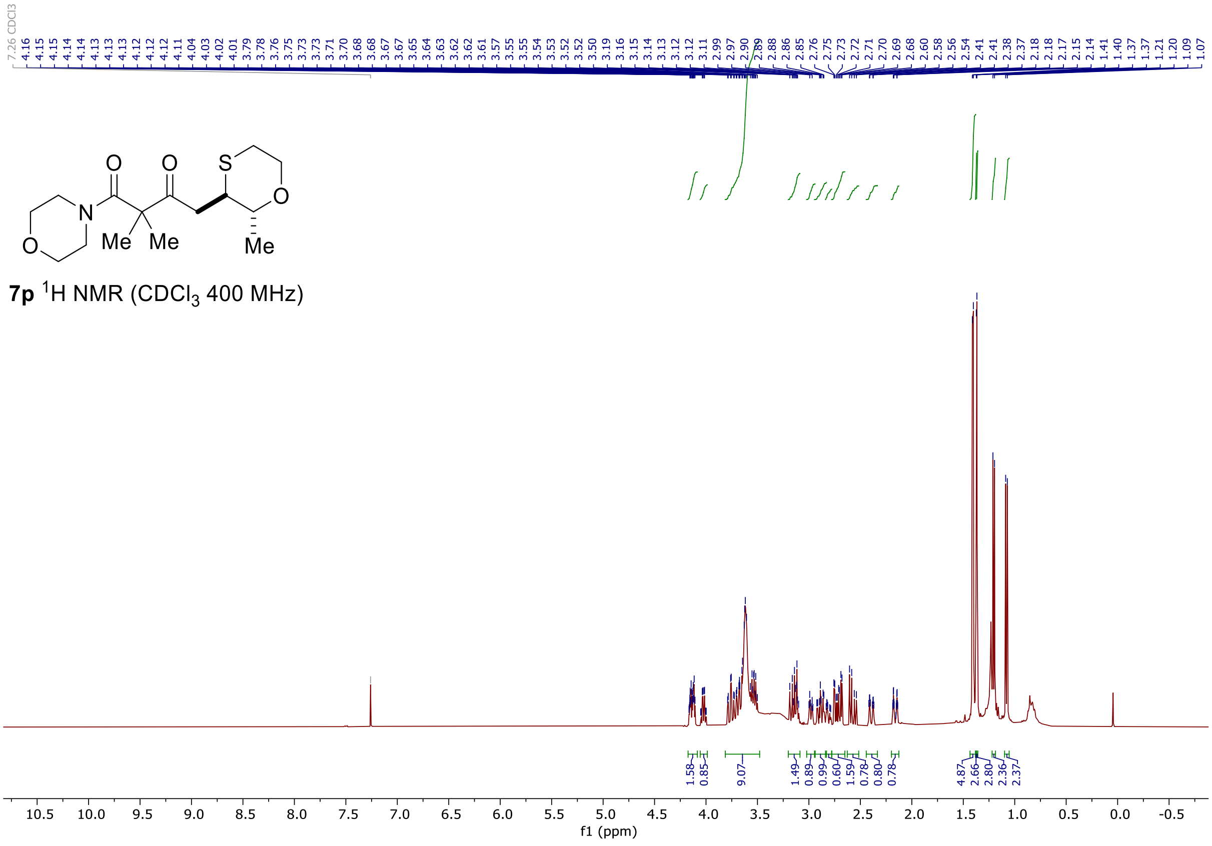

7p ^{1}H NMR ($CDCl_3$ 400 MHz)

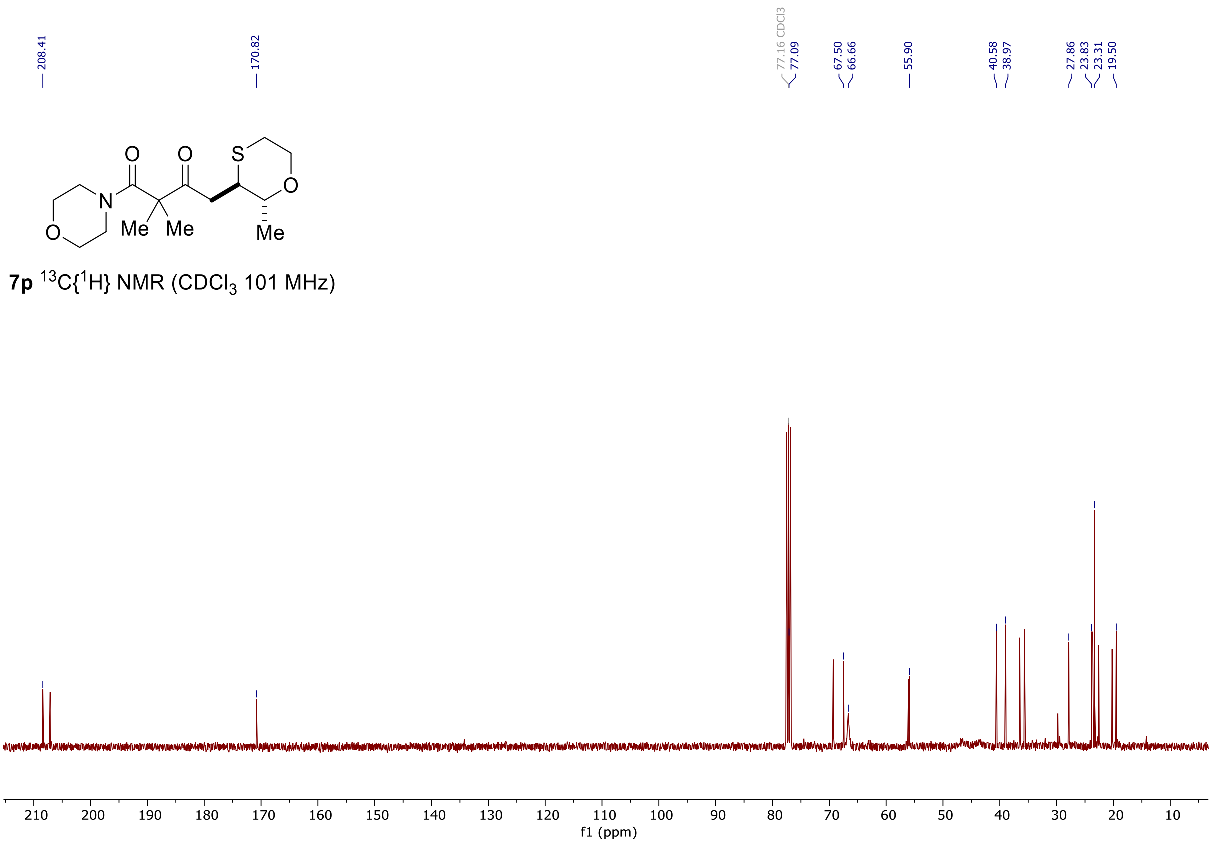

7p $^{13}C\{^{1}H\}$ NMR ($CDCl_3$ 101 MHz)

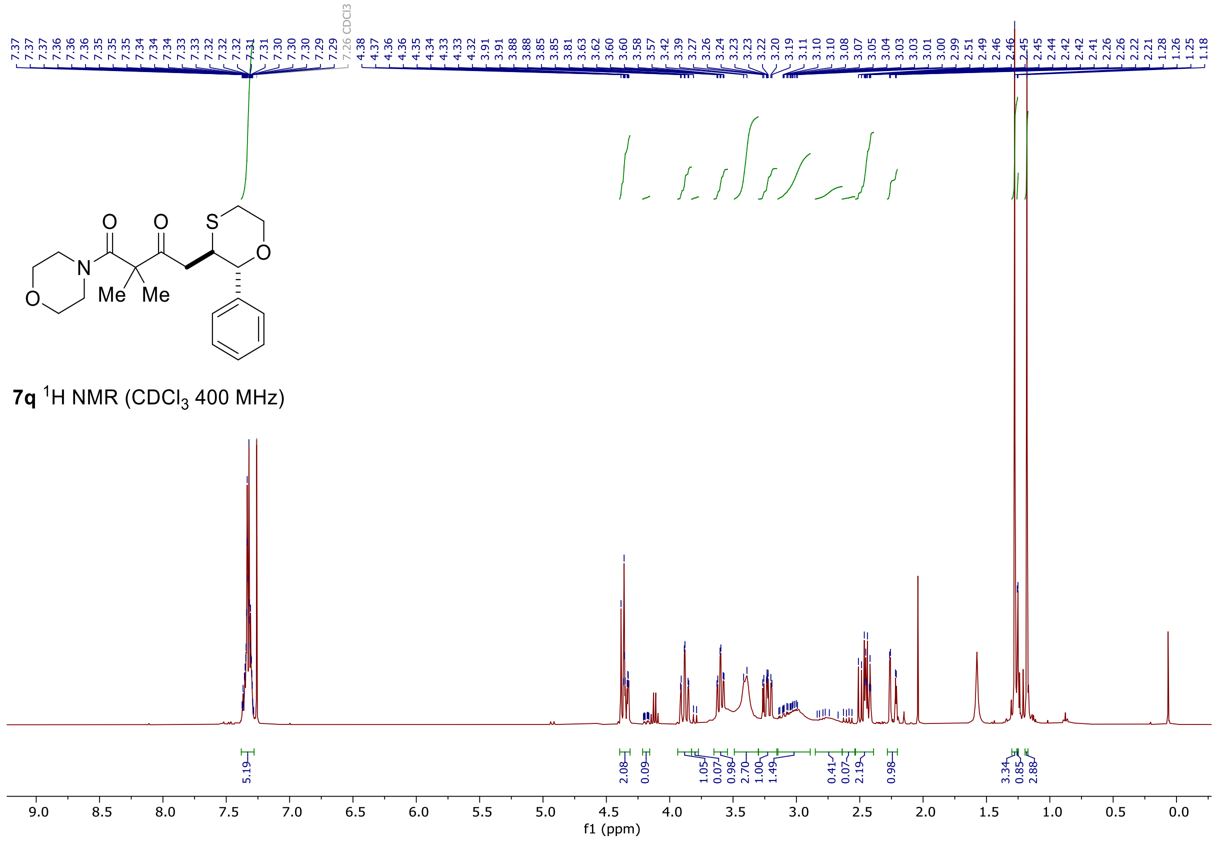

7q ^{1}H NMR ($CDCl_3$ 400 MHz)

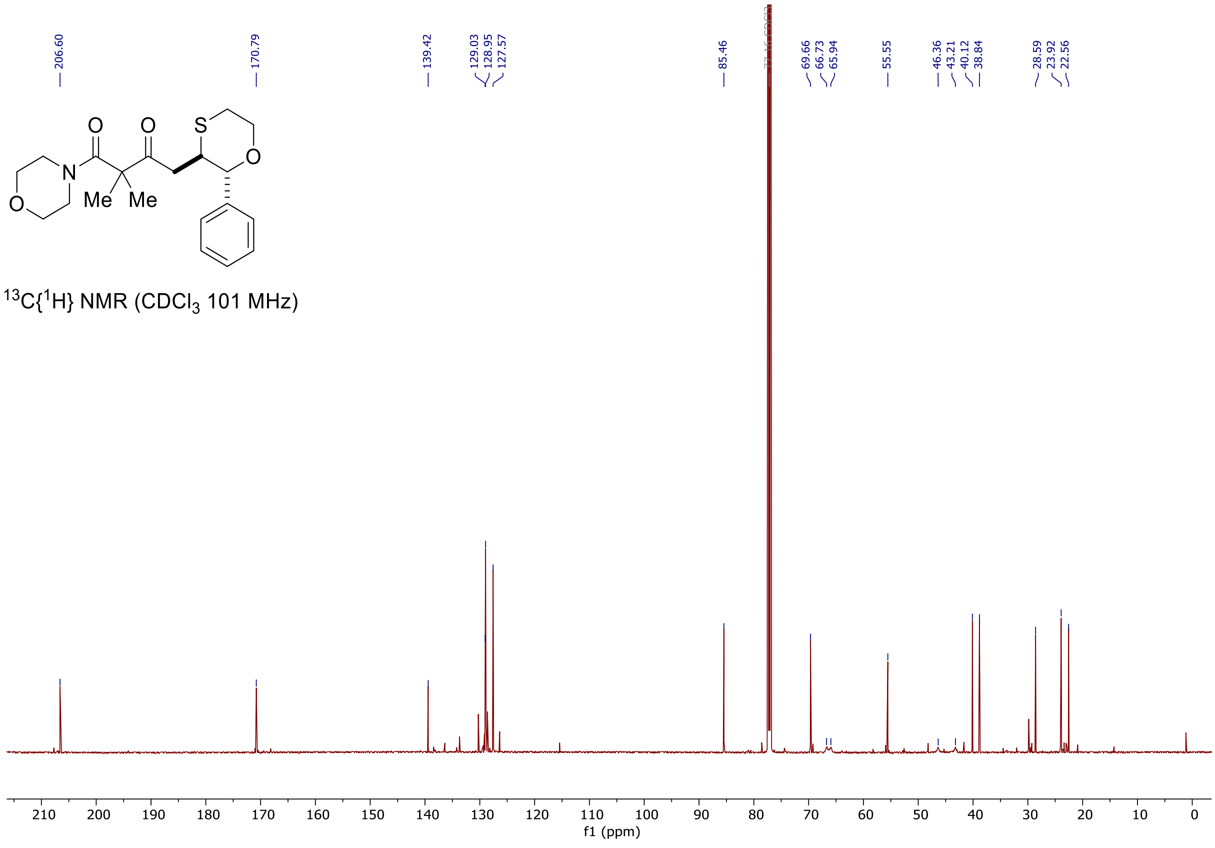

7q $^{13}C\{^{1}H\}$ NMR ($CDCl_3$ 101 MHz)

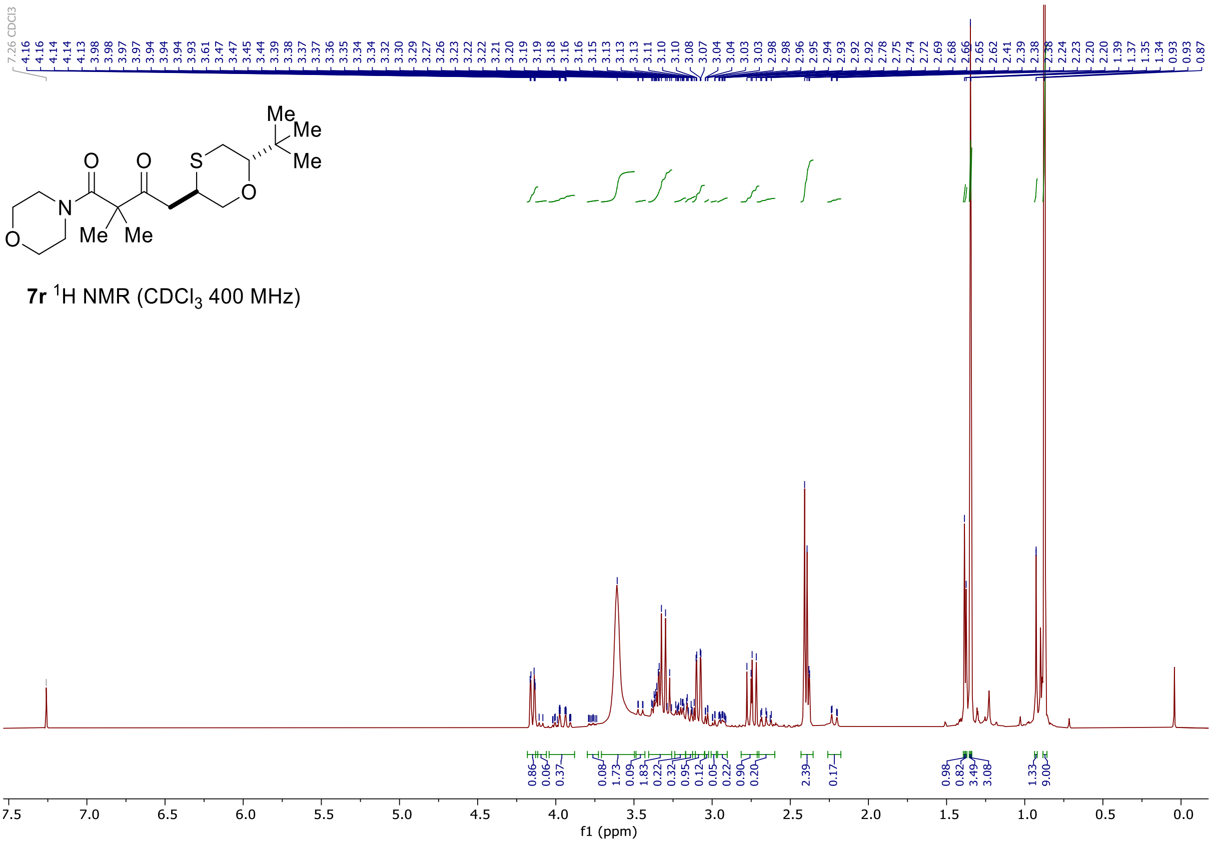

7r ^{1}H NMR ($CDCl_3$ 400 MHz)

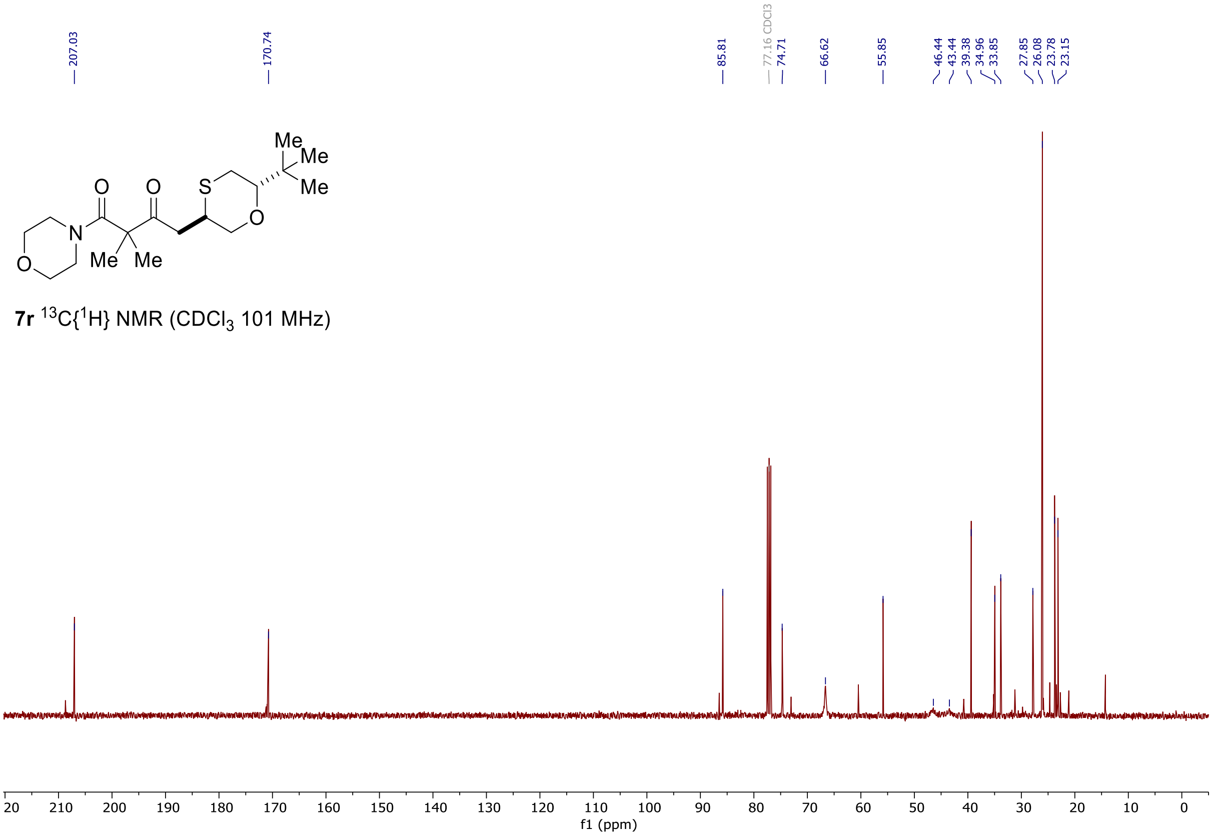

7r $^{13}C\{^{1}H\}$ NMR ($CDCl_3$ 101 MHz)

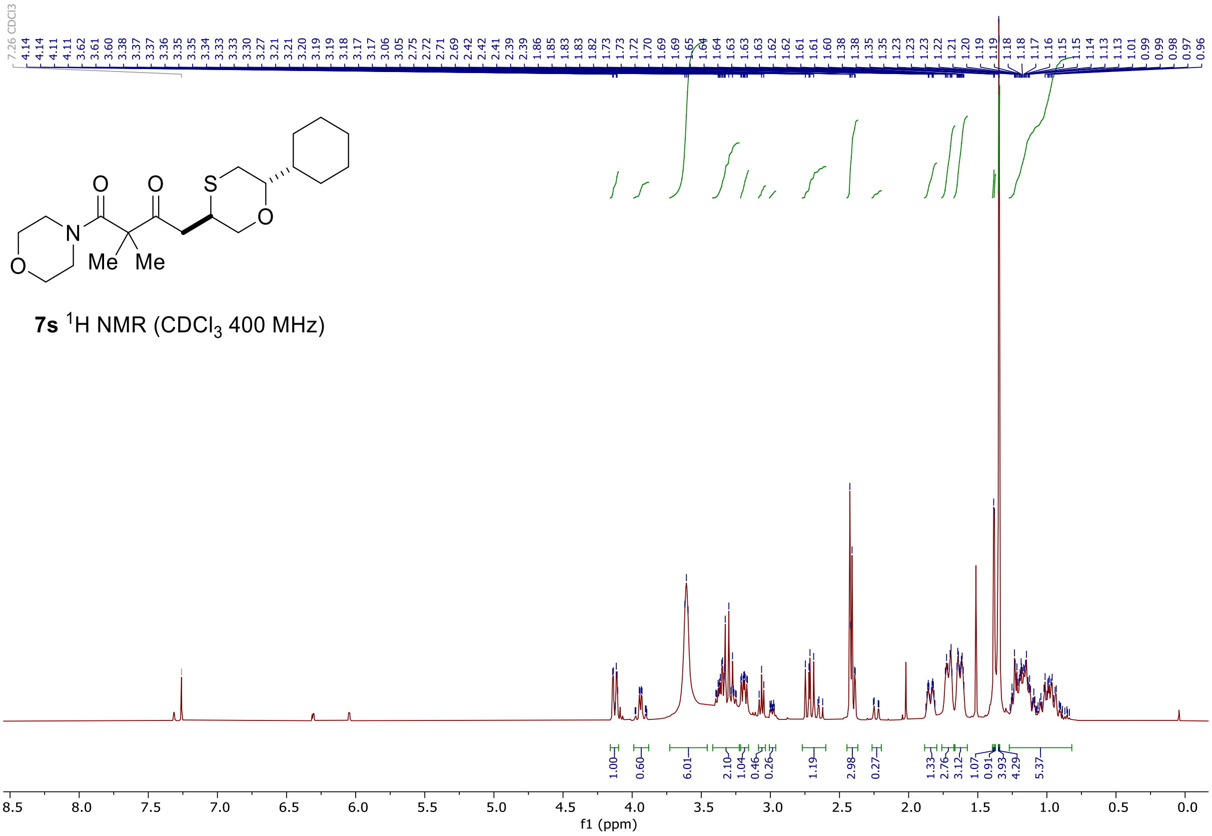

7s ^{1}H NMR ($CDCl_3$ 400 MHz)

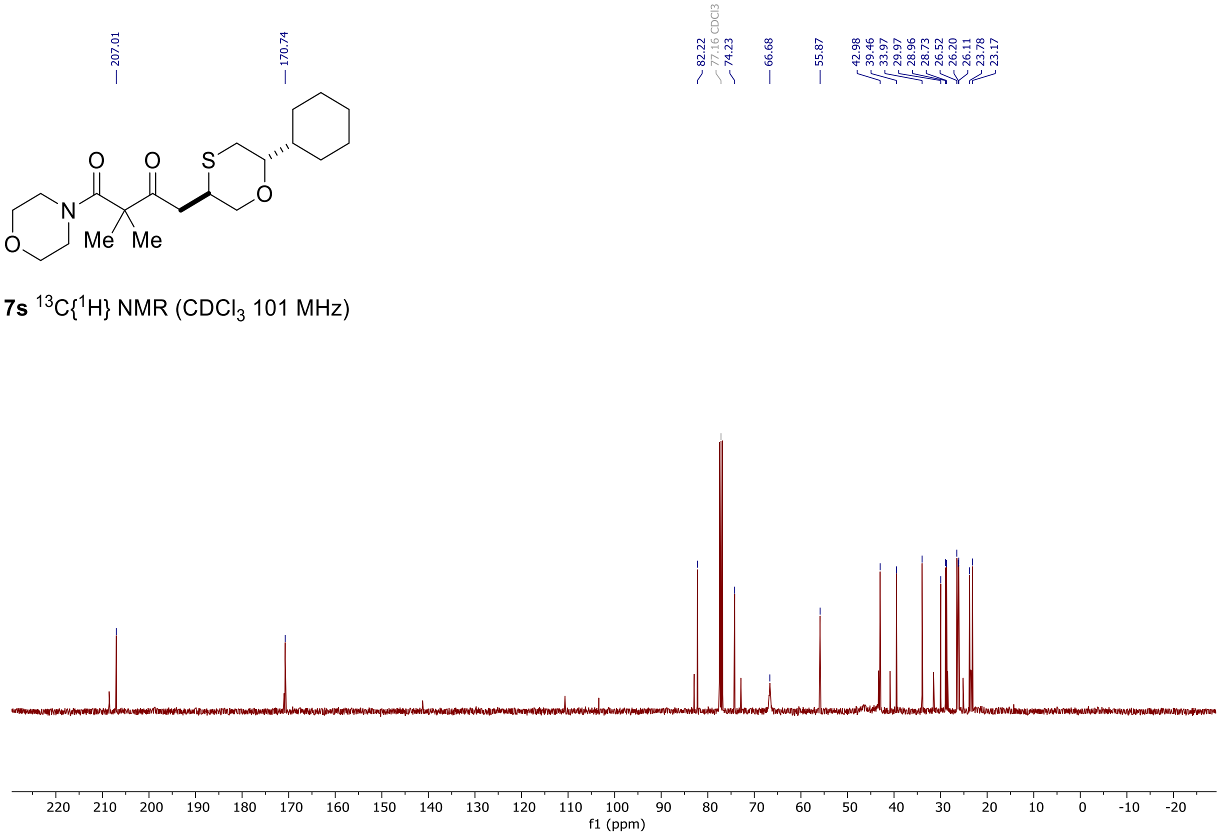

7s $^{13}C\{^{1}H\}$ NMR ($CDCl_3$ 101 MHz)

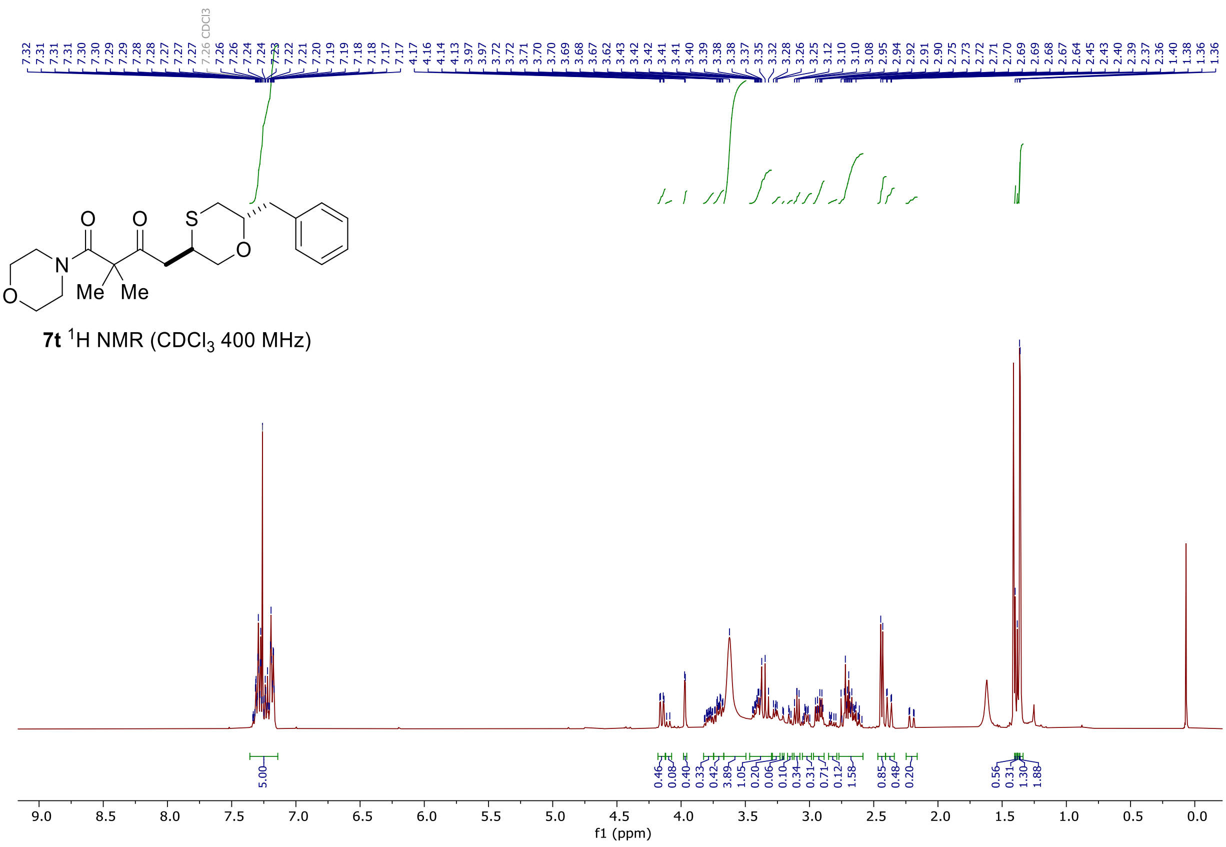

7t ^{1}H NMR ($CDCl_3$ 400 MHz)

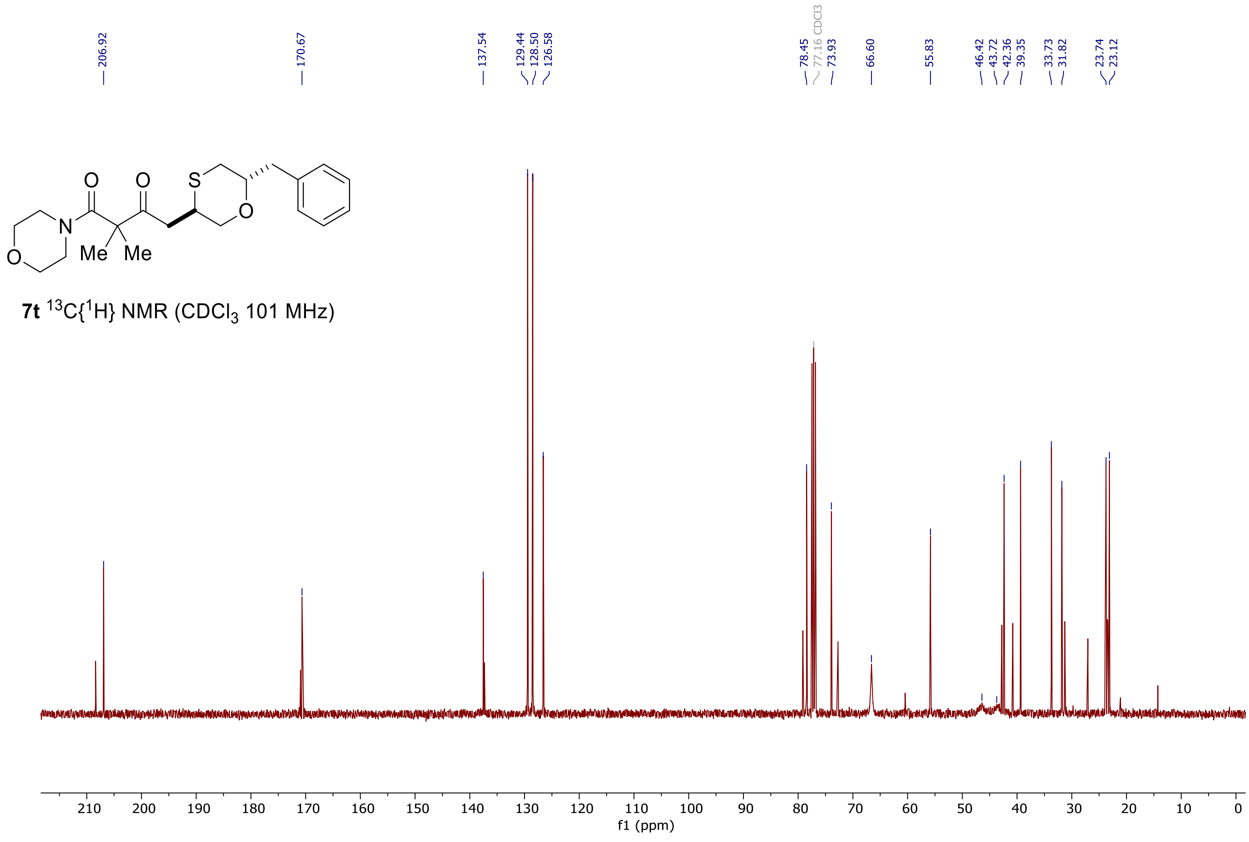

7t $^{13}C\{^{1}H\}$ NMR ($CDCl_3$ 101 MHz)

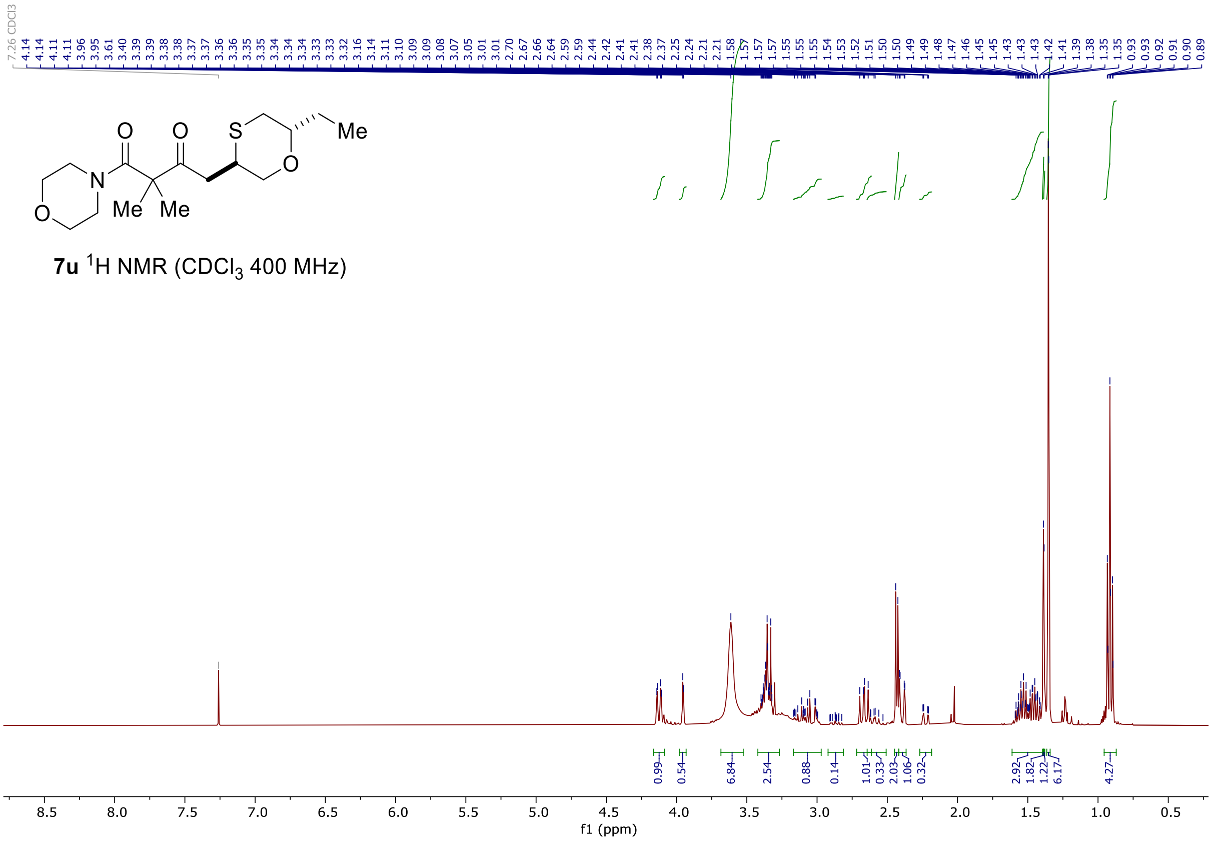

7u ^{1}H NMR ($CDCl_3$ 400 MHz)

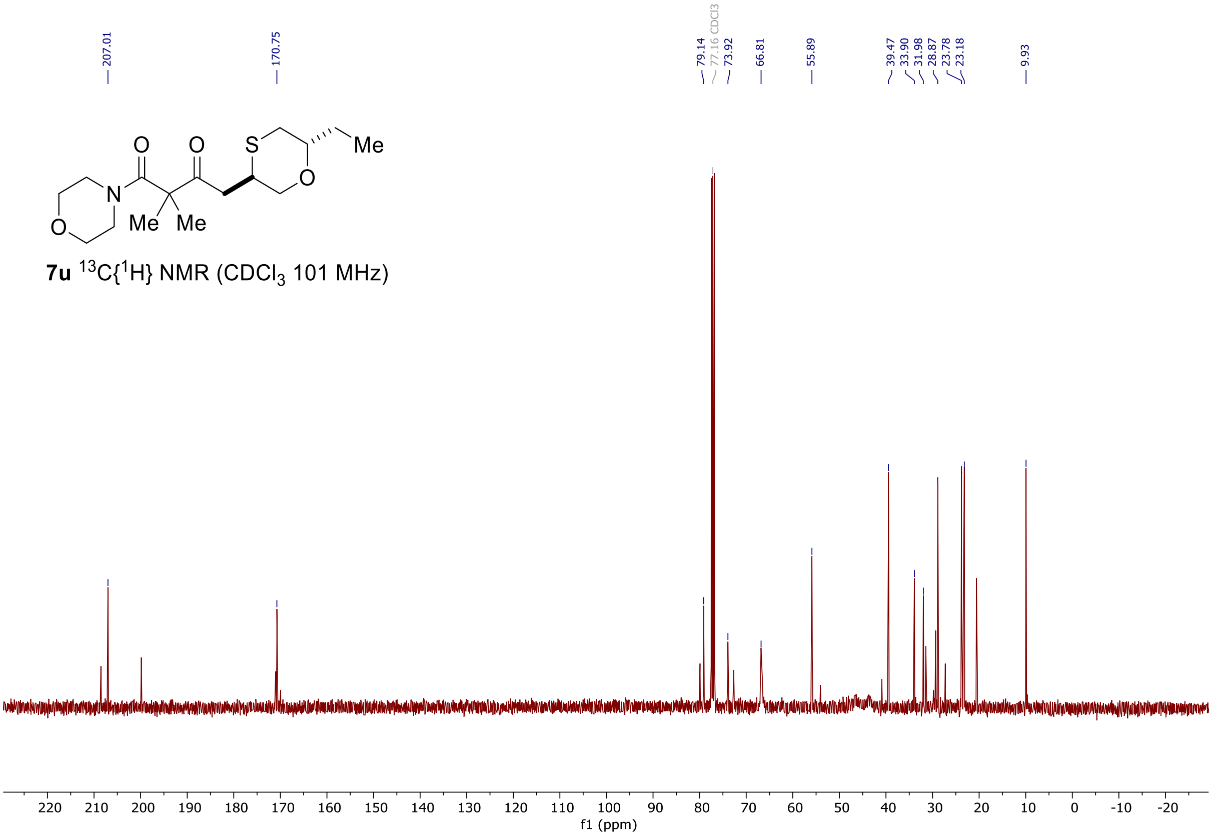

7u ^{13}C{^{1}H} NMR ($CDCl_3$ 101 MHz)

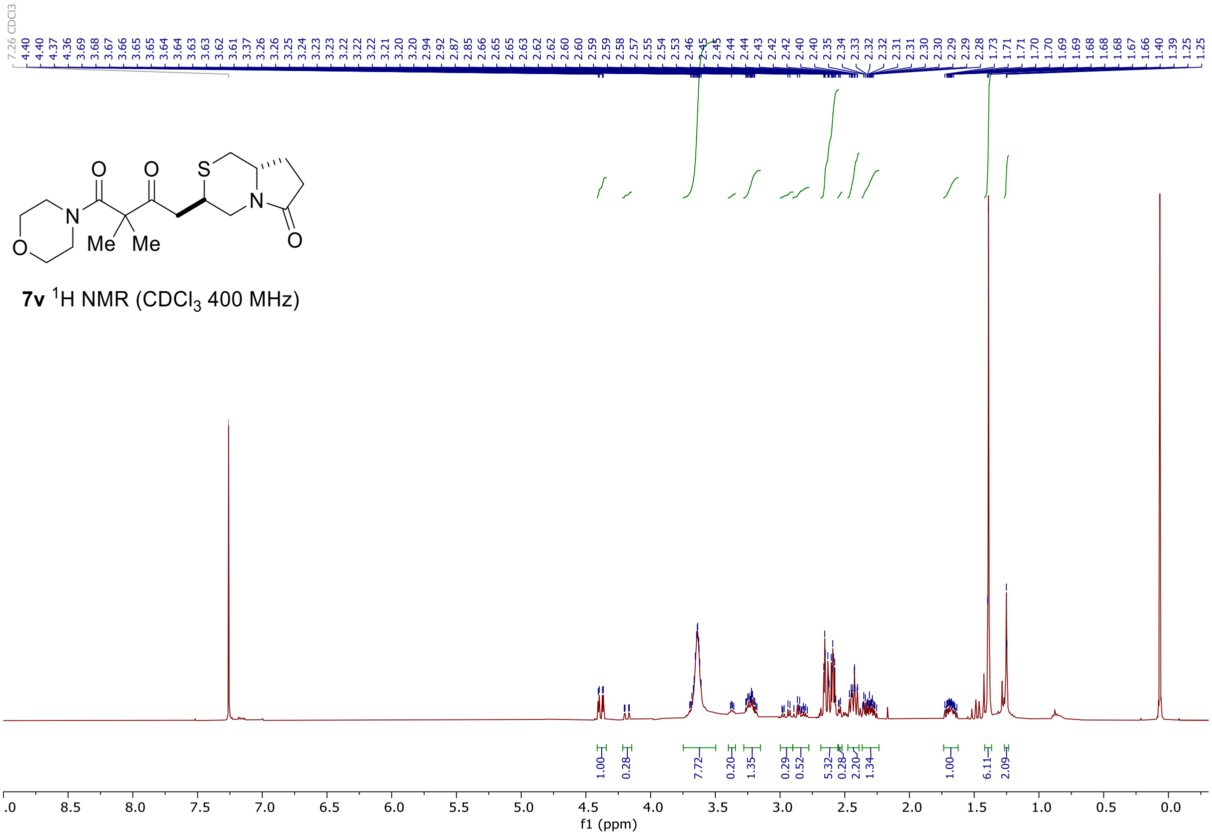

7v ^{1}H NMR ($CDCl_3$ 400 MHz)

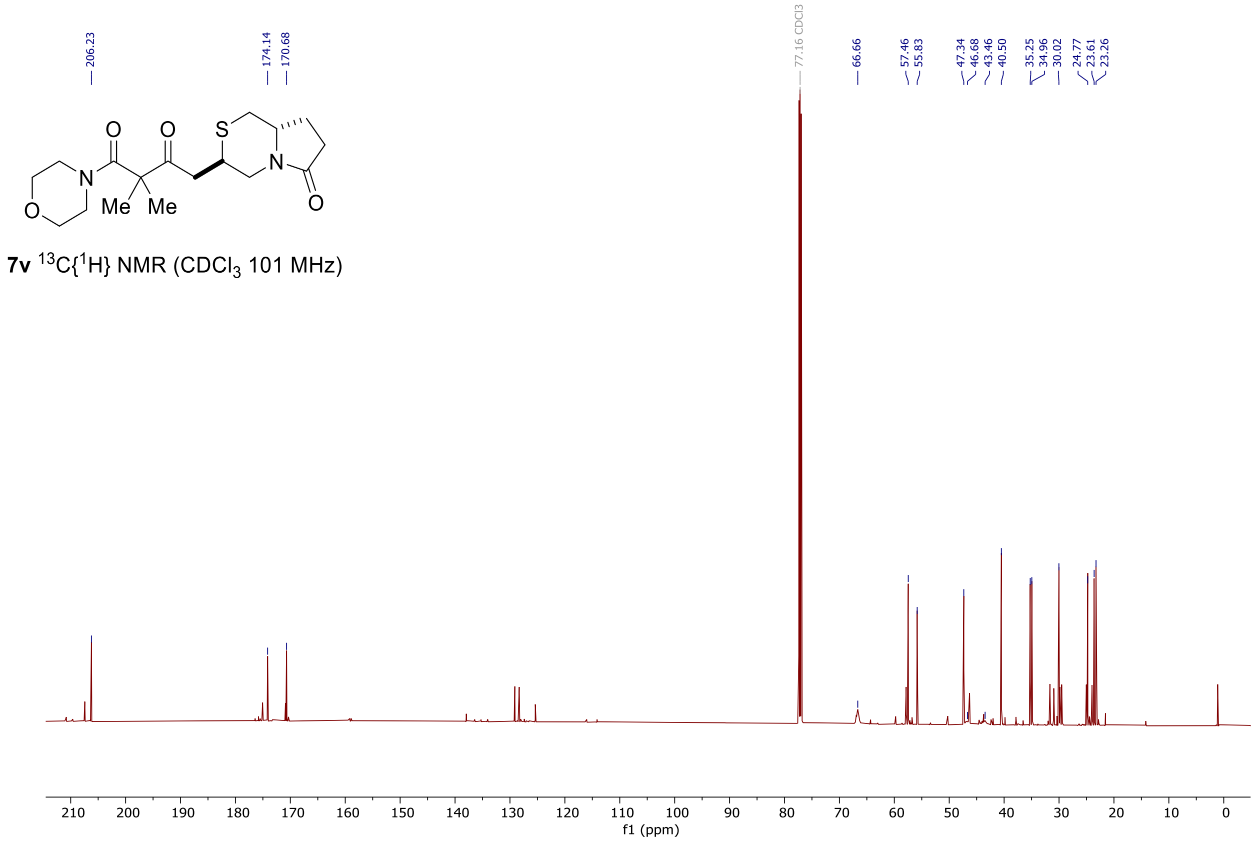

7v ^{13}C{^{1}H} NMR ($CDCl_3$ 101 MHz)

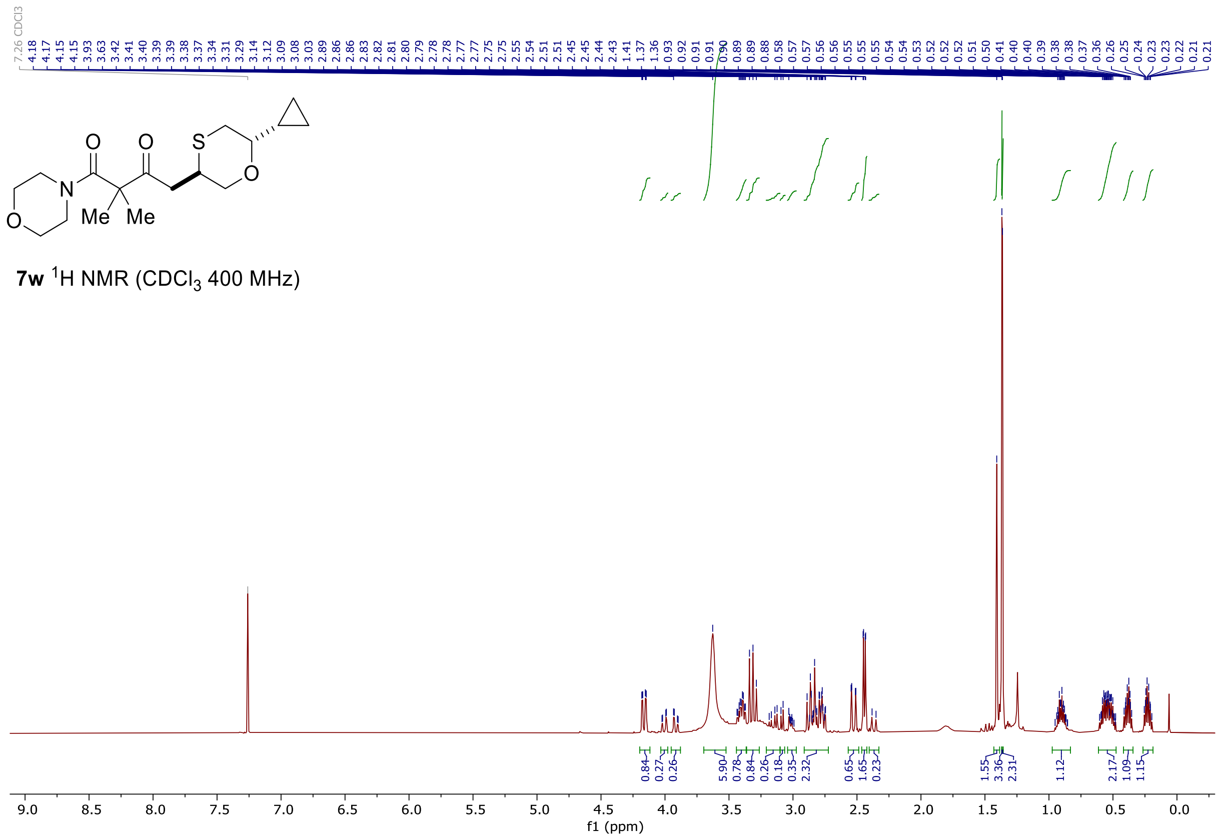

7w ^{1}H NMR ($CDCl_3$ 400 MHz)

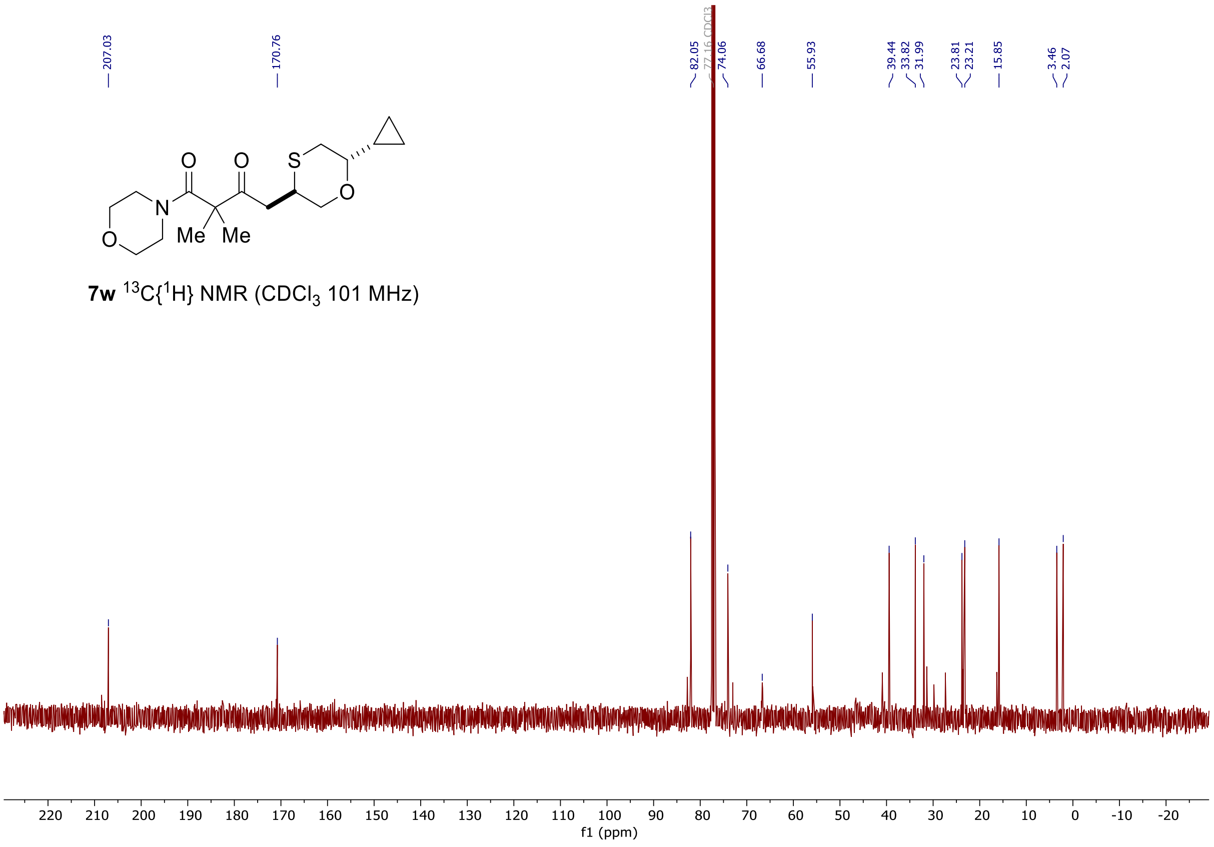

7w ^{13}C{^{1}H} NMR ($CDCl_3$ 101 MHz)

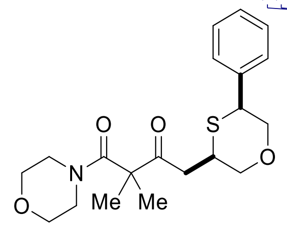

7y ^{1}H NMR ($CDCl_3$ 400 MHz)

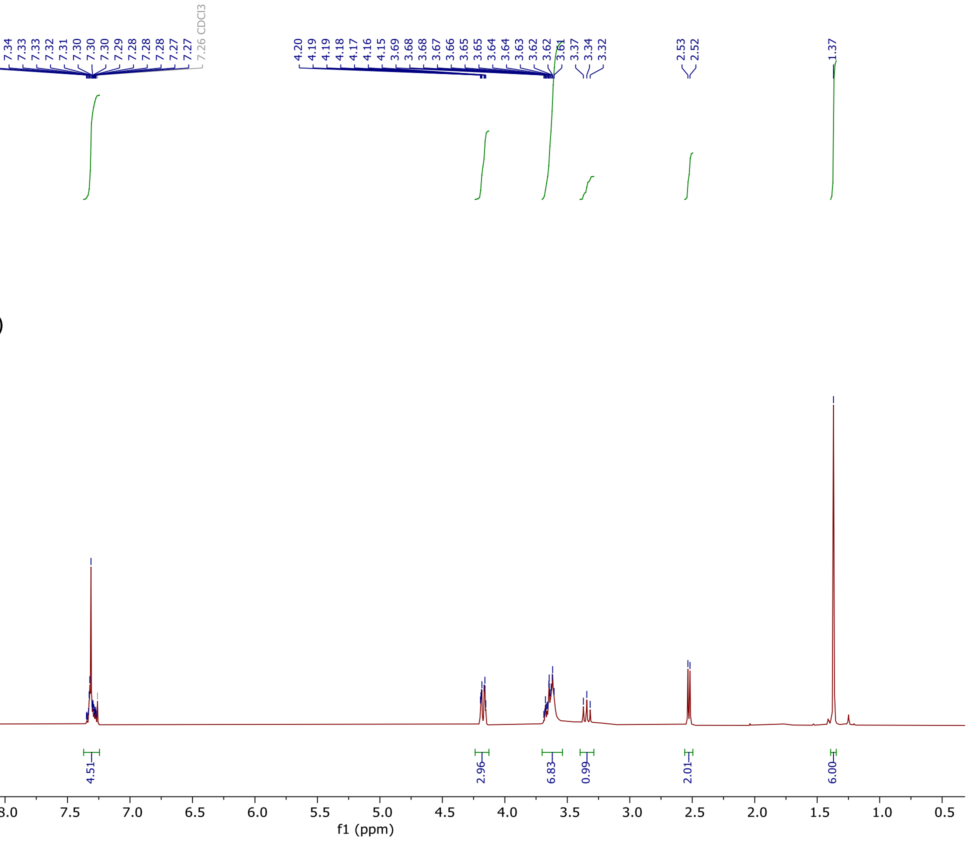

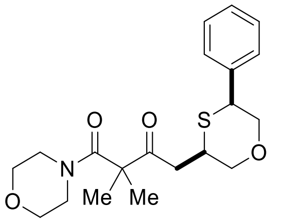

7y ^{13}C{^{1}H} NMR ($CDCl_3$ 101 MHz)

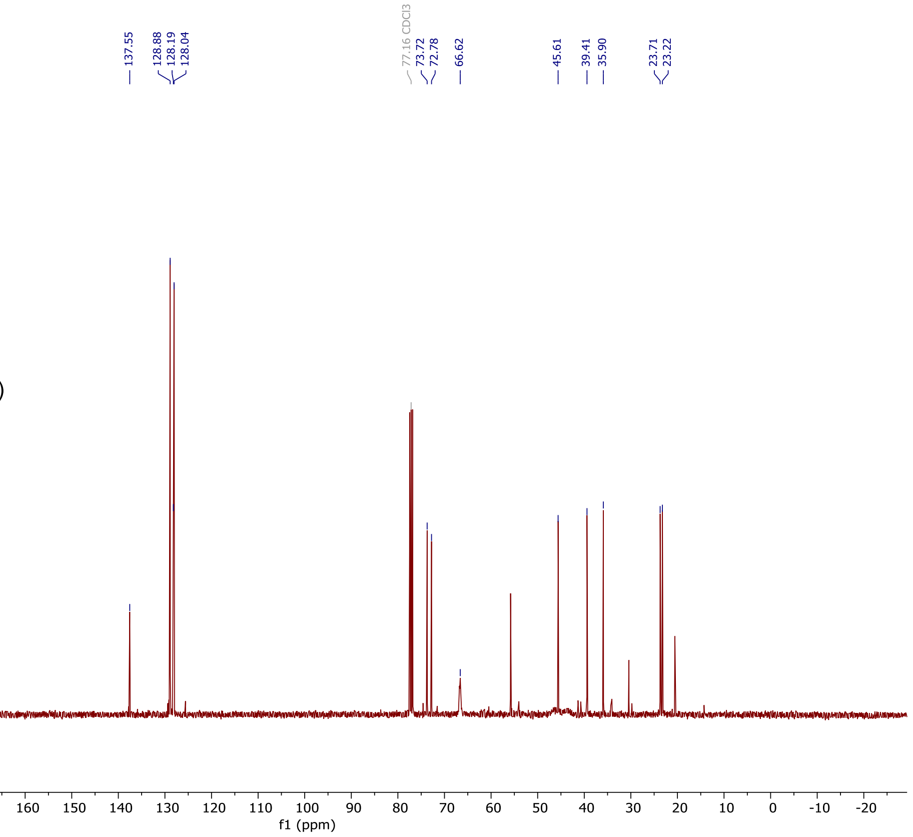

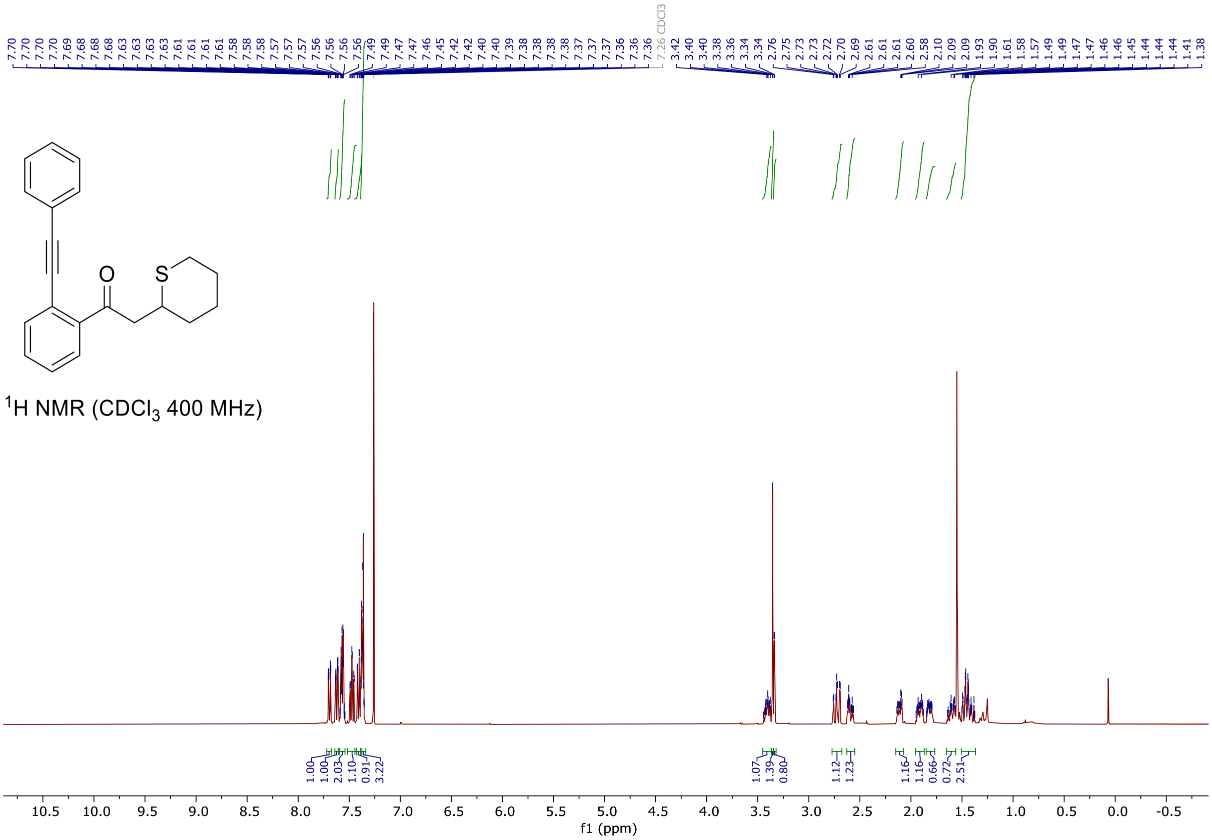

11h ^{1}H NMR ($CDCl_3$ 400 MHz)

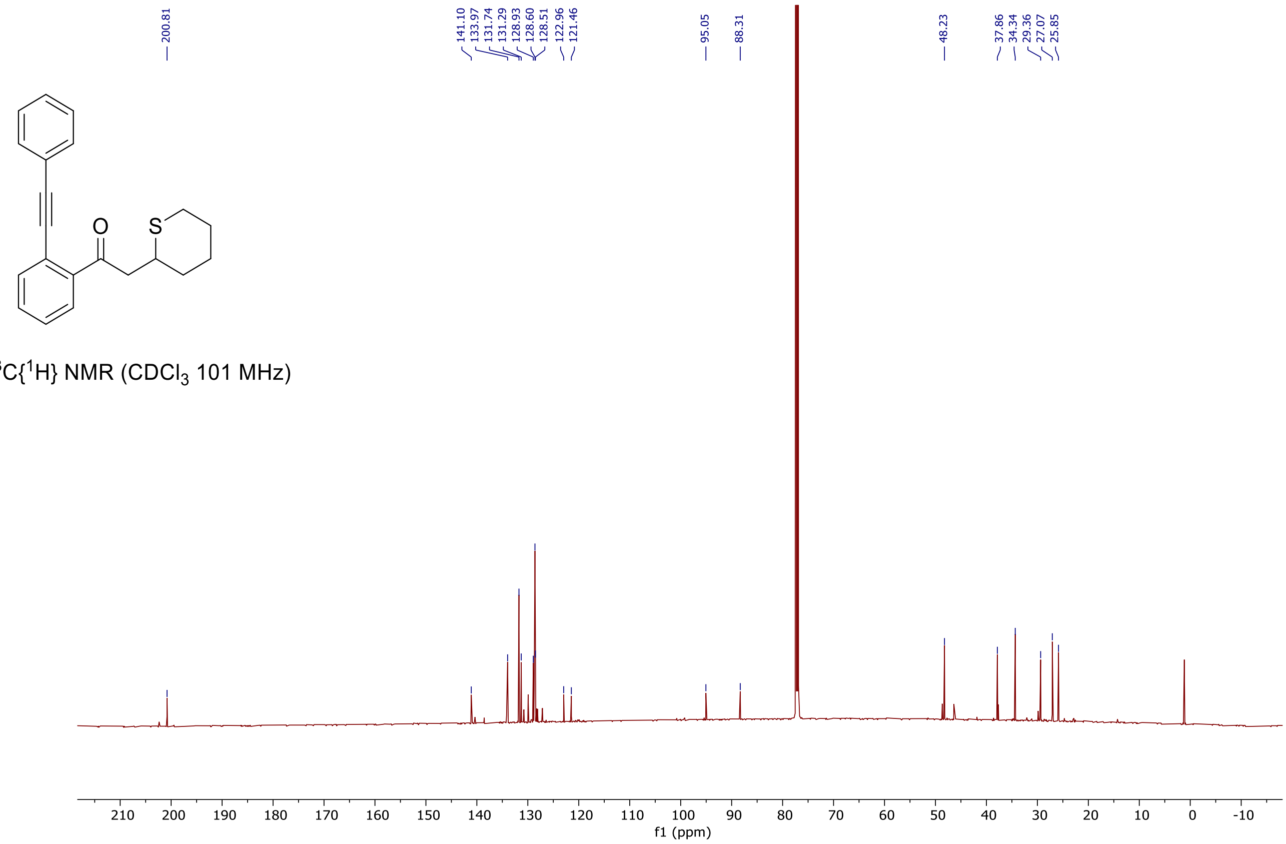

11h $^{13}C\{^{1}H\}$ NMR ($CDCl_3$ 101 MHz)

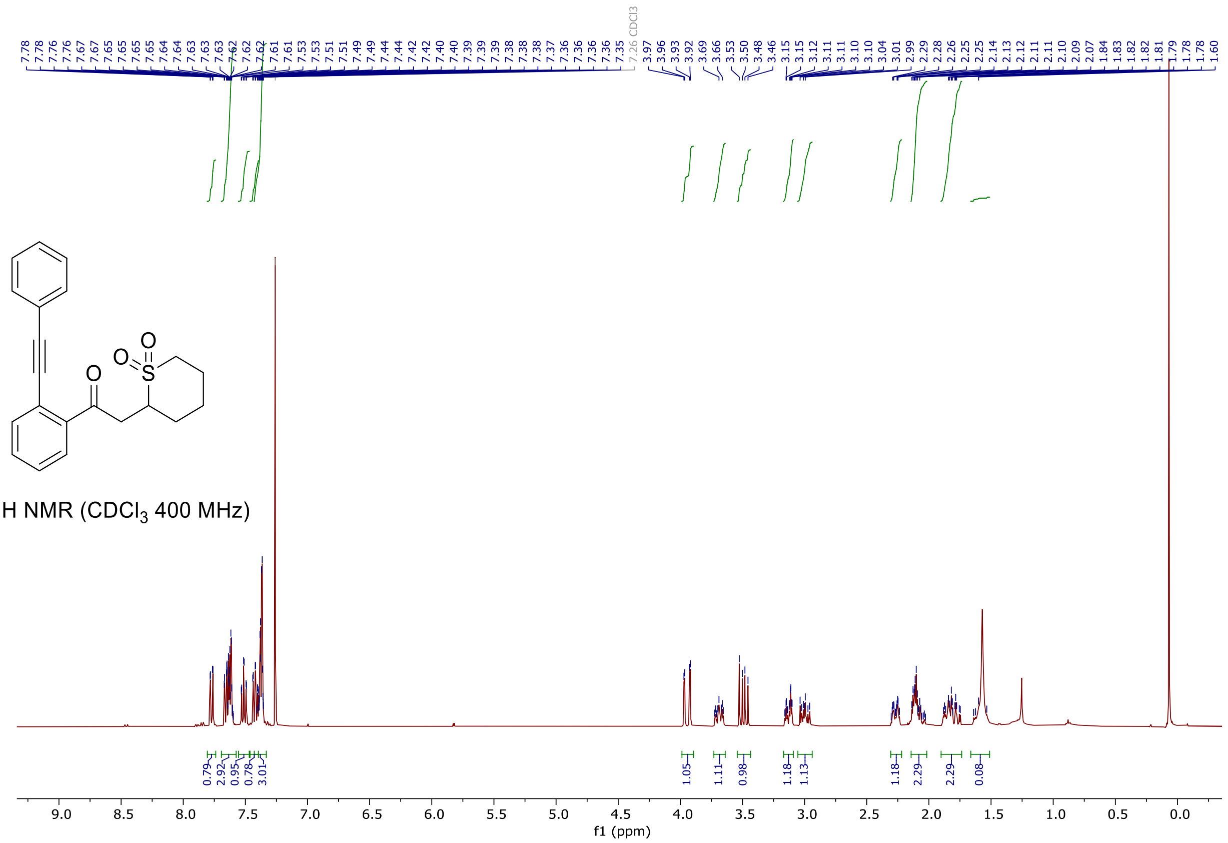

11i ^{1}H NMR ($CDCl_3$ 400 MHz)

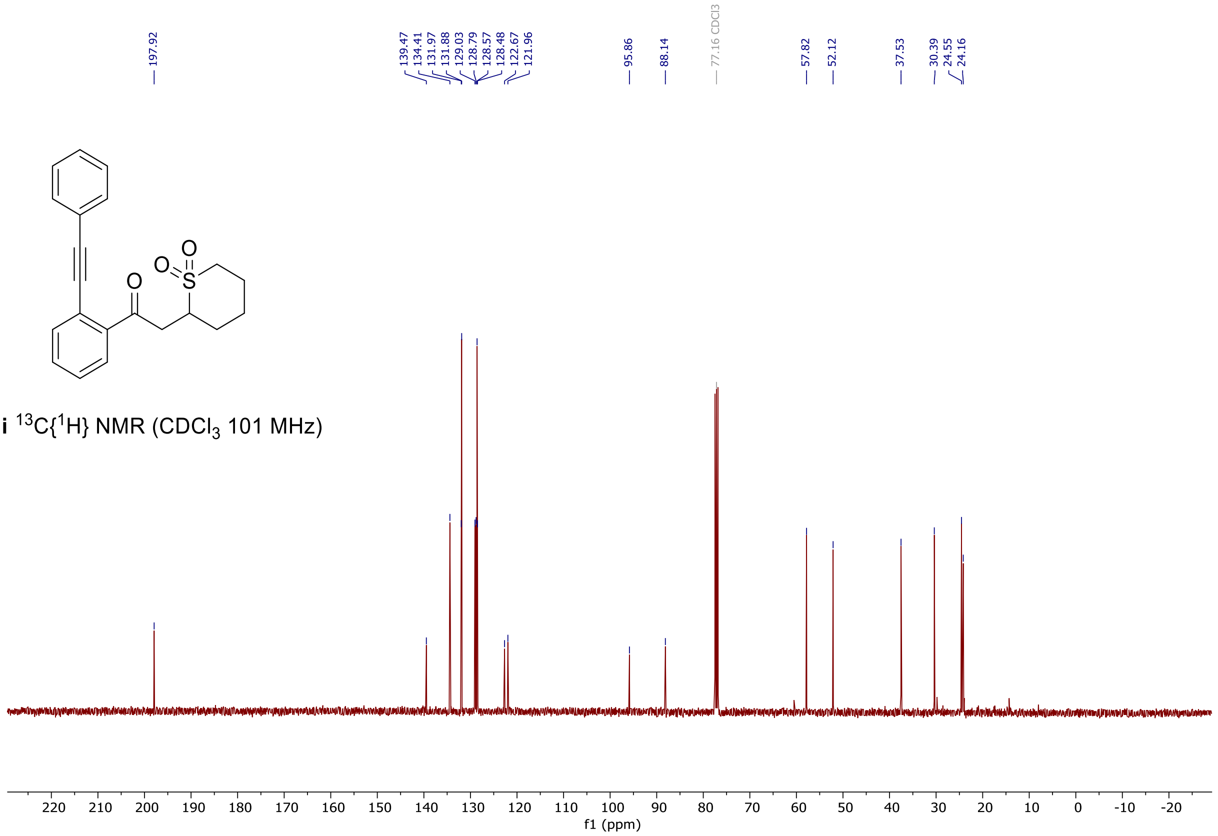

11i ^{13}C{^{1}H} NMR ($CDCl_3$ 101 MHz)

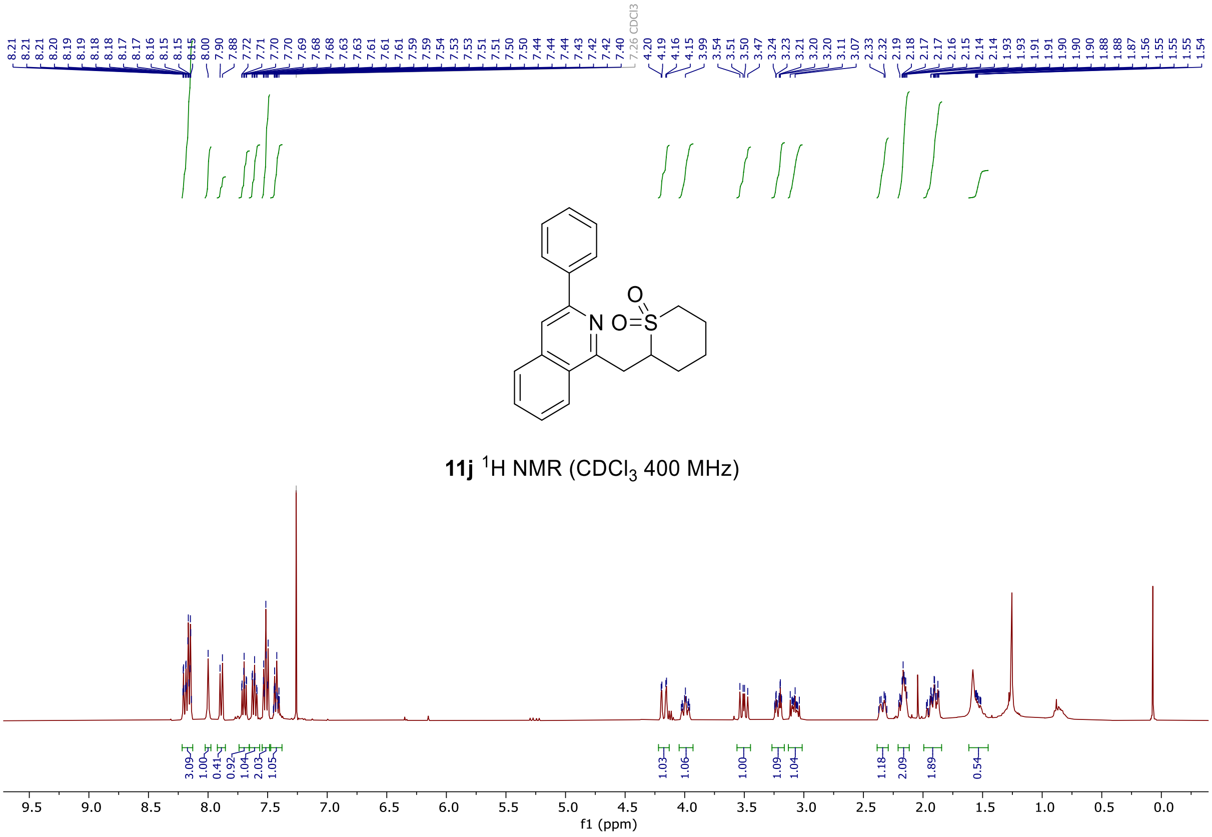

11j ^{1}H NMR ($CDCl_3$ 400 MHz)

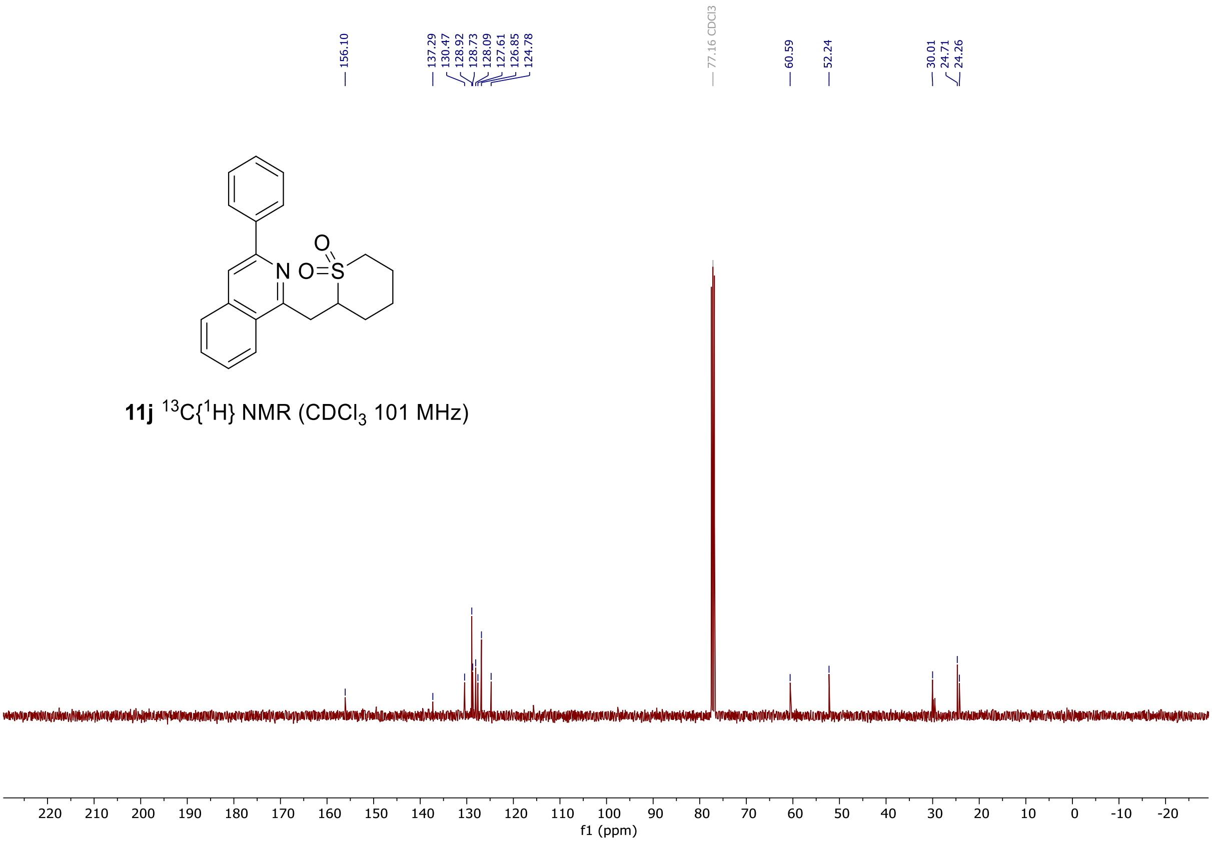

11j $^{13}C\{^{1}H\}$ NMR ($CDCl_3$ 101 MHz)
